# Coupling of unactivated alkyl electrophiles using frustrated ion pairs

**DOI:** 10.1038/s41586-024-08195-1

**Published:** 2024-11-20

**Authors:** Sven Roediger, Emilien Le Saux, Philip Boehm, Bill Morandi

**Affiliations:** Laboratorium für Organische Chemie, ETH Zürich, Zürich, Switzerland

**Keywords:** Synthetic chemistry methodology, Reaction mechanisms

## Abstract

Cross-electrophile coupling reactions have evolved into a major strategy for rapidly assembling important organic molecules^1^. Two readily accessible electrophiles are coupled to form new C–C bonds, providing a key advantage over traditional cross-coupling strategies that require the preformation of reactive organometallic species. Yet, the formation of C(*sp*^3^)–C(*sp*^3^) bonds that form the core of nearly all organic compounds remains highly challenging with current approaches, calling for the design of innovative new strategies. Here we report a distinct, transition-metal-free platform to form such bonds without the need for activating or stabilizing groups on the coupling partners. The reaction is enabled by an unusual single-electron transfer in a frustrated ion pair, and it can couple fragments containing functional groups that would be challenging in related transition-metal-catalysed processes. Moreover, we could further leverage this new mechanistic manifold in the design of other reactions, showing the broad potential of this type of reactivity. We anticipate that our results will provide a framework for further exploration of this reactivity pattern to tackle challenging problems in organic synthesis.

## Abstract (web summary)

A transition-metal-free platform enables the formation of challenging C(*sp*^3^)–C(*sp*^3^) bonds in organic compounds via single-electron transfer, facilitating the coupling of functionalized fragments and expanding possibilities for efficient organic synthesis and reaction design.

## Main

**Fig. 1:**
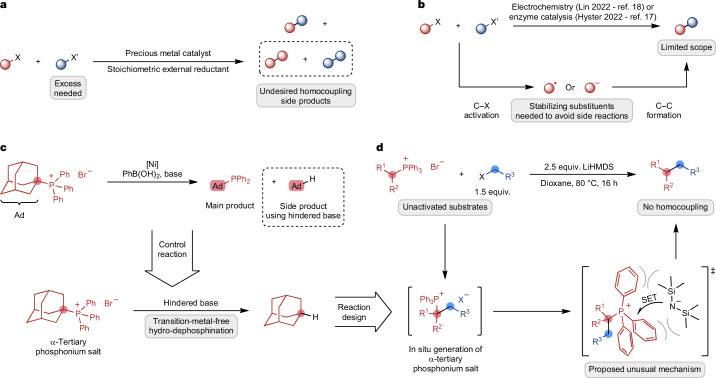
Context of this work. **a**, Limitations of transition-metal-catalysed C(*sp*^3^)–C(*sp*^3^) XECs. **b**, Current methods for transition-metal-free C(*sp*^3^)–C(*sp*^3^) XECs. **c**, Discovery of C(*sp*^3^)–P bond activation in phosphonium salts. **d**, This work: transition-metal-free C(*sp*^3^)–C(*sp*^3^) coupling of unactivated substrates enabled by a frustrated ion pair.

Carbon-based frameworks constitute the backbone of organic molecules that are used in applications ranging from pharmaceuticals to materials. Thus, the modular and efficient construction of C–C bonds is one of the ultimate goals in organic synthesis. Traditionally, cross-coupling reactions between an organometallic reagent and an electrophile have been used for this task, leading to the synthesis of numerous essential molecules^2,3^. However, one of the coupling partners usually needs to be pre-functionalized as an organometallic nucleophilic reagent, increasing the step count and requiring the handling of potentially sensitive intermediates. By contrast, recently discovered cross-electrophile coupling reactions (XECs) bypass the use of organometallic reagents by directly coupling two electrophilic coupling partners^1,4,5^. XECs are most often catalysed by transition metals and rely on an external stoichiometric reductant for catalytic turnover. Often, these reactions require at least one coupling partner with C(*sp*^2^) hybridization at the reactive site to control chemoselectivity^1,6^. Forming C(*sp*^3^)–C(*sp*^3^) bonds by XECs is notably more difficult^7–11^, but highly desirable owing to their ubiquity in organic molecules. For example, the fraction of C(*sp*^3^) atoms in molecules has been positively correlated with the clinical success of drug candidates, creating an even stronger demand for C(*sp*^3^)–C(*sp*^3^) bond-forming reactions^12–14^. A challenge in transition-metal-catalysed C(*sp*^3^)–C(*sp*^3^) XECs is the propensity to form homocoupling side products instead of the desired heterocoupling products^15^ (Fig. 1a). This unwanted reactivity can be partially reduced by using a large excess of one of the coupling partners, leading to additional waste^16^. Combined with the negative economic and environmental impact of transition metals, the development of alternative, transition-metal-free strategies is highly desirable. This would not only avoid potential issues with metal contamination for subsequent applications but could also improve the scope of C(*sp*^3^)–C(*sp*^3^) XECs to include functional groups that are reactive under many metal-catalysed conditions or that could poison a metal catalyst. In this context, two seminal reports emerged recently that used either electrochemical or enzymatic platforms to enable this highly challenging reactivity^17,18^ (Fig. 1b). These approaches rely on the formation of radical or anionic intermediates from one of the substrates that can then be engaged in C–C bond formation with the second coupling partner. To avoid undesired side reactions of these highly reactive intermediates, stabilizing substituents (for example, amides or aromatics) are required, inherently restricting these approaches in their reaction scope and preventing the use of fully unactivated substrates. Therefore, a transition-metal-free XEC of two fully unactivated and unstabilized C(*sp*^3^) coupling partners has so far remained elusive, clearly highlighting the limitations of current approaches. There is, thus, an urgent need for the design of strategically distinct mechanistic manifolds that can address these challenges and thereby unlock the immense potential of XECs for the assembly of C(*sp*^3^)–C(*sp*^3^) linkages ubiquitously found across the molecular sciences.

Here, we report the discovery and investigation of an unprecedented coupling related to XEC in which two completely unactivated and non-organometallic C(*sp*^3^) fragments are combined in a transition-metal-free protocol. Alkylphosphonium salts are reacted with alkyl halides in the presence of a sterically hindered base as the sole reagent to give rise to the coupled products (Fig. 1d). Mechanistic studies suggest that the reaction is enabled by an unusual single-electron transfer (SET) in a frustrated ion pair that is reminiscent of frustrated Lewis pair radical reactivity^19–26^.

Phosphonium salts are key reagents in organic synthesis. They are readily available and widely used, for example, in the Wittig reaction to construct alkenes^27^. Recently, their intriguing reactivity has also been harnessed in unconventional manners, including the selective functionalization of heteroarenes^28,29^. Moreover, the use of phosphonium salts as C(*sp*^2^) electrophiles in transition-metal-catalysed cross-coupling reactions has been investigated^30–32^. Our group has a longstanding interest in diverting the reactivity of phosphonium salts towards unusual transformations, such as C–P metathesis and related transformations for late-stage phosphine functionalization^33,34^. During our recent studies^35^, we discovered conditions under which the P–C(*sp*^3^) bond of the phosphonium salt is activated in addition to the more reactive P–C(*sp*^2^) bond if the phosphonium salt contained a tertiary alkyl group (Fig. 1c). This peculiar hydro-dephosphination sparked our curiosity as it occurred in the absence of any conventional reductant. We reasoned that this unexpected bond activation likely arose from a different mechanism than oxidative addition to the metal catalyst. Our hypothesis was confirmed by subsequent control experiments, which demonstrated that the reaction does not require a transition metal catalyst to yield alkane products and is mediated by a sterically hindered base. Although the activation of C–P bonds in phosphonium salts is well known to occur by basic hydrolysis, it is documented to favour the cleavage of aromatic substituents over alkyl substituents if the alkyl group is not activated^36^. Indeed, subjecting *tert*-alkyltriphenylphosphonium salts to known hydrolysis conditions^37^ did not lead to the formation of the desired alkane products (Supplementary Information section 5.8), implying that the discovered hydro-dephosphination process occurred by a different reaction pathway with distinct selectivity. Although the nature of this bond activation was unclear at this stage, we aimed to leverage this new reactivity to design an innovative approach to challenging C(*sp*^3^)–C(*sp*^3^) bond formation. We hypothesized that an α-tertiary alkylphosphonium salt could be formed in situ by the alkylation of a phosphorus ylide, which can be generated through the deprotonation of a phosphonium salt (Fig. 1d). Subsequent base-mediated hydro-dephosphination would enable an overall coupling between these two C(*sp*^3^) electrophiles that does not require the presence of a transition metal catalyst.

Initial experiments using phosphonium salts and alkyl iodides as the alkylating agent indeed confirmed our hypothesis, as the alkane product was obtained in moderate yields using sterically hindered bases. After careful optimization (Supplementary Tables 1–5), we developed a highly efficient transition-metal-free coupling between unactivated *sec*-alkylphosphonium salts and unactivated alkyl halides. This reaction is mediated by lithium hexamethyldisilazide (LiHMDS) as the only added reagent.

**Fig. 2:**
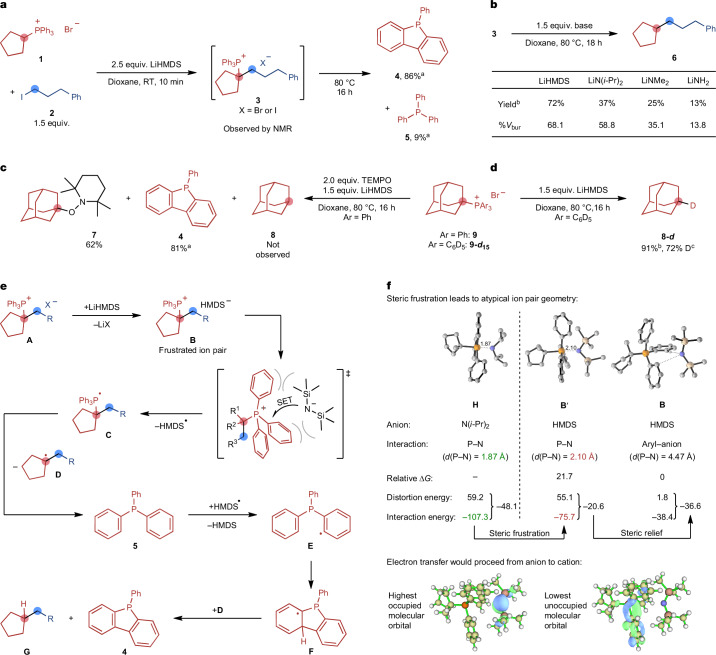
Mechanistic studies. **a**, Investigation of the C–C bond formation and identification of the main byproduct. RT, room temperature. **b**, Effect of the base size. **c**, Reaction inhibition by TEMPO. **d**, Deuteration experiment. **e**, Proposed mechanism of the C–P cleavage. **f**, Computed properties of the frustrated ion pair **B**. Energies are in kcal mol^–1^. ^a^^31^P{^1^H} NMR yield using triphenyl phosphate as the internal standard. ^b^Gas chromatography yield using *n*-dodecane as the internal standard. ^c^Determined by ^1^H NMR spectroscopy of the isolated material. *G*, Gibbs free energy; %*V*_bur_, percent buried volume.

As the reaction does not contain an obvious reductant and the mode of C–P bond activation is unconventional, we became interested in understanding the origin of this unique reactivity. As a first finding, we noticed that triphenylphosphine (**5**), the expected byproduct from a traditional reductive C–P bond activation, was formed only in small amounts (9% nuclear magnetic resonance (NMR) yield), whereas dibenzophosphole **4** was the main byproduct of the reaction in 86% NMR yield. This suggested that the phosphonium moiety serves as a reductant because of the oxidation of two phenyl C–H bonds to a C–C bond in dibenzophosphole **4** (Fig. 2a). Reaction monitoring by NMR spectroscopy also showed that the phosphonium salt **1** is rapidly deprotonated to its ylide in the presence of LiHMDS (Supplementary Fig. 8). When the phosphonium salt **1**, LiHMDS, and alkyl iodide **2** are reacted at room temperature, the *tert*-alkylphosphonium salt **3** is formed as the main species after a few minutes. The identity of **3** was confirmed by an independent synthesis (Supplementary Information, section 6.3). The C–C bond formation is, thus, fast and proceeds through the alkylation of a phosphorus ylide, as set out in our reaction design. These results also highlight that the unique reactivity of the phosphonium electrophile is crucial for the coupling reactivity as it enables the C–C bond formation through a deprotonation-mediated philicity switch to the nucleophilic ylide and also acts as an internal reductant for the unusual oxidation to the dibenzophosphole **4** (refs. ^24,25^). Subjecting the *tert*-alkylphosphonium salt **3** to different bases demonstrated that product formation is strongly dependent on the size of the base (Fig. 2b). Although LiHMDS provided a high yield of product, smaller amide bases gave much lower yields that decreased with the base size. This demonstrates that the size of the base is not only important for limiting undesired S_N_2 reactions of the base with the alkyl halide substrate but also has an important role in the cleavage of the C–P bond. To further study this key step, which is at the core of this new reaction, we used adamantyltriphenylphosphonium bromide (**9**), a phosphonium salt that already contains a tertiary alkyl group, as a model substrate. When **9** was reacted with LiHMDS, adamantane (**8**) was formed in a similar yield as the coupling products under the model reaction conditions (Supplementary Information, section 6.5). However, adding 2,2,6,6-tetramethylpiperidinyloxyl (TEMPO) as a radical trap to this reaction shut down the formation of adamantane (**8**). Instead, the TEMPO adduct **7** was isolated in 62% yield, suggesting the formation of *tert*-alkyl radicals in the reaction (Fig. 2c). Interestingly, dibenzophosphole **4** was formed in a similar yield compared with the standard reaction conditions, and its formation was, therefore, not influenced by the presence of TEMPO. As the formation of dibenzophosphole **4** from the PPh_3_ moiety in the starting phosphonium salts requires the loss of two aryl hydrogen atoms, we next subjected phosphonium salt **9-*d***_**15**_, containing perdeuterated phenyl groups, to the reaction conditions (Fig. 2d). NMR analysis indicated that the adamantane isolated from this reaction contained 72% deuterium at one of the tertiary positions (**8-*d***).

With these results in hand, we propose a plausible reaction mechanism (Fig. 2e). The starting phosphonium salt is deprotonated by LiHMDS, and the resulting ylide is rapidly alkylated by the alkyl halide to yield the α-tertiary phosphonium salt **A**. Next, salt metathesis between **A** and LiHMDS takes place first, leading to a sterically encumbered ion pair **B**. Experiments using different alkali HMDS salts showed that the presence of lithium ions is important for the reaction, possibly because the formation of LiX (X = Br or I) serves as a driving force for the salt metathesis step. Similarly, the addition of 12-crown-4 to the reaction, which selectively binds lithium ions^38^, shut down the reactivity (Supplementary Table 12). The key step enabling the coupling reaction is a SET from the HMDS anion to the phosphonium cation in the frustrated ion pair **B**. Such SET processes in phosphonium halide salts are well known to occur photochemically by ultraviolet irradiation or also by visible-light activation in the case of phosphonium salts containing strongly electron-withdrawing substituents^39–43^. By contrast, the thermal process has only been postulated for the reaction of tetraphenylphosphonium chloride with lithium amides based on preliminary data^24,25^. At the time this study was carried out, no applications or detailed studies of such a process existed. We however would like to point out a complementary report^44^, using a SET step in a frustrated ion pair for C–H activation, that appeared shortly after an initial version of our work was deposited as a preprint.

In the lowest-energy isomer of the salt **B**, the HMDS anion forms a C–H⋯anion interaction with a phenyl group of the phosphonium ion and an additional π–anion interaction with a second phenyl group (Fig. 2f). Isomers with the direct interaction of the phosphonium P atom and the anion in a phosphorane-type geometry (isomer **B**′), which are typical for smaller nucleophiles (Supplementary Information section 7.3), are much higher in energy^28^. We performed distortion–interaction analyses on the different phosphonium HMDS ion pair geometries and a related smaller ion pair containing the N(*i*-Pr)_2_ anion (structure **H**) to understand the origin of the unusual isomer preference (Fig. 2f). The analysis showed that the phosphorane-like isomer **B**′ of the HMDS ion pair is destabilized because the interaction energy is substantially lower than in the case of the smaller N(*i*-Pr)_2_ ion pair **H** (−75.7 kcal mol^–1^ and −107.3 kcal mol^–1^, respectively). This is caused by the large steric bulk of the two ions, which leads to a longer P–N distance in the HMDS ion pair **B**′ (2.10 Å) than in the N(*i*-Pr)_2_ ion pair **H** (1.87 Å). Because of this steric frustration, the formation of the atypical phosphonium amide ion pair **B** containing interactions between the HMDS anion and the phosphonium phenyl groups becomes favoured. Analysis of the frontier orbitals of **B** shows that the highest occupied molecular orbital is located on the HMDS anion whereas the lowest unoccupied molecular orbital is delocalized on the phosphonium cation, in line with the direction of the proposed electron transfer. Calculations of the redox potentials of the ions in **B** show that the proposed SET is accessible under the experimental conditions (Supplementary Table 14). The SET step would be preceded by the formation of a charge-transfer complex^45,46^. Indeed, strong charge-transfer bands at around 380 nm and 515 nm, potentially arising from such an interaction, were visible by ultraviolet–visible spectroscopy when the phosphonium salt **9** was mixed with LiHMDS (Supplementary Fig. 9). A calculated ultraviolet–visible spectrum of **B** shows a charge-transfer band at 348 nm, closely matching one of the experimentally observed bands. By contrast, conformer **B**′ is predicted to have little charge-transfer character (272 nm). This suggests that **B** is more likely to be the active ion pair leading to SET, and its formation is enabled by the steric frustration in the phosphonium HMDS ion pair.

The SET step leads to the formation of a phosphoranyl radical **C** and an HMDS radical. The α-scission of **C** forms the tertiary alkyl radical **D** and triphenylphosphine (**5**). The highly reactive HMDS radical can abstract a hydrogen atom from triphenylphosphine (**5**), resulting in the aryl radical **E**, which can undergo cyclization to the cyclic radical **F**. The weak alkyl C–H bond in **F** can be abstracted by the previously generated alkyl radical **D** to aromatize the phosphorus-containing ring system to the experimentally observed byproduct **4** and leading to the alkane product **G**. The proposed pathway is supported by additional density functional theory calculations (Supplementary Information, section 7).

**Fig. 3:**
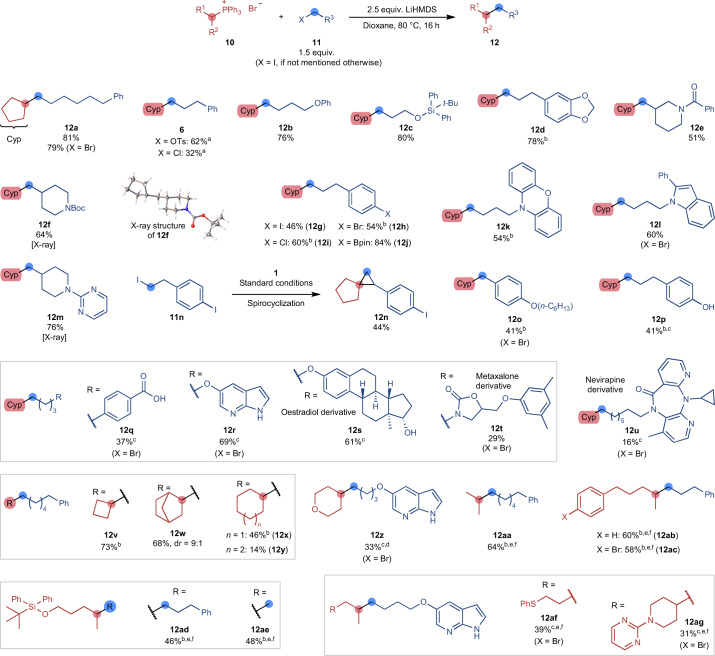
Scope of the coupling reaction. Yields refer to isolated compounds if not stated otherwise. ^a^Gas chromatography yield using *n*-dodecane as the internal standard. ^b^Isolated after hydroboration–oxidation treatment of the crude reaction mixture. ^c^4.0 equiv. LiHMDS. ^d^The product contains a small impurity. The yield has been adjusted accordingly. ^e^Phosphonium iodide as the starting material. ^f^Reaction conducted at 120 °C.

Having gained an increased understanding of the reaction mechanism, we explored the scope of the reaction (Fig. 3). Reacting phosphonium salts with an almost equimolar amount of alkyl halides in the presence of LiHMDS provided a large variety of C(*sp*^3^)–C(*sp*^3^)-coupled products without forming homocoupling side products that are problematic under transition-metal-catalysed conditions. Although the reaction works best using alkyl iodides as coupling partners, alkyl bromides provide the product in a similar yield (79% versus 81%, respectively, for **12a**). Alkyl tosylates could also be engaged and gave the product in a fair yield (**6**, 62%), providing an opportunity to use alcohol starting materials in the reaction after facile derivatization to the corresponding tosylate. Alkyl chlorides participated in the reaction as well, although in lower yields (**6**, 32%). Ethers, silyl-protected alcohols, and acetal moieties were well tolerated (**12b**–**d**). Substrates containing amides or *tert*-butyloxycarbonyl (Boc)-protected amines provided the desired coupled products in moderate yields (**12e** and **12f**). Groups that would be reactive under many transition-metal-catalysed conditions, namely, aryl halides (**12g**–**i**) and aryl boronate esters (**12j**), gave rise to the desired products. The reaction also tolerates the presence of heterocycles such as phenoxazines (**12k**), indoles (**12l**), and pyrimidines (**12m**). When the homobenzylic iodide **11n** was engaged as a coupling partner, the spirocyclopropane **12n** was isolated in 44% yield instead of the coupling product. We hypothesize that the corresponding styrene of **11n** is formed by a fast E2 elimination to which the in situ formed phosphorus ylide can be added. This would give rise to a benzylic carbanion that can undergo 1,3-elimination with the phosphonium moiety to form the cyclopropane ring^47^. In contrast to **11n**, the benzylic bromide **11o** formed the desired coupling product **12o** in a moderate yield, demonstrating that the reaction can be used for the coupling of electron-rich activated alkyl halides in addition to the unactivated ones. The reaction can furthermore be carried out in the presence of acidic moieties such as alcohols (**12p** and **12s**), carboxylic acids (**12q**), and unprotected azaindoles (**12r**) when additional LiHMDS is used in the reaction to account for the deprotonation of the acidic groups. Moreover, substrates containing the hormone oestradiol (**11s**) and the drug molecules nevirapine and metaxalone (**11t** and **11u**) were successfully engaged in the coupling reaction. Phosphonium salts containing differently sized cyclic alkyl groups afforded the alkane products (**12v**–**z**). Smaller-ring phosphonium salts provided higher yields (**12v** and **12w**) than larger-ring ones (**12x**–**z**). We noticed increased levels of alkene side products in these reactions that might arise from a Hofmann-type elimination of the alkylated phosphonium salt intermediate. A range of acyclic phosphonium salts containing functional groups such as aryl halides or heterocycles provided the corresponding products (**12aa**–**12ag**). Notably, the phosphonium salt **11ad** was also engaged with iodomethane to afford the methylated product **12ae** in the same yield as product **12ad**, which contained a longer-chain alkyl group.

To further evaluate the functional group tolerance of the reaction, we carried out a compatibility screen with different additives^48^ (Supplementary Fig. 7). Several functional groups that are known to be reactive or inhibitory under many transition-metal-catalysed reactions, such as terminal alkenes, tertiary amines, pyridines, and epoxides, did not interfere with the reaction. Limitations of the reaction include nitro compounds and most carbonyl groups such as ketones, which can undergo Wittig-type side reactions. The addition of an enol ether, however, did not noticeably inhibit the reaction, indicating that protected ketones may be viable functionalities in substrates.

**Fig. 4:**
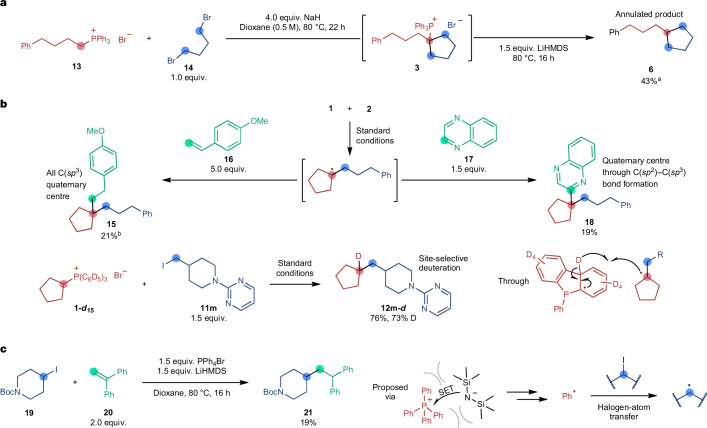
Mechanistically informed extensions of the reaction scope. **a**, Annulation reaction through a double ylide alkylation. **b**, Difunctionalization and deuteration reactions by interception of the alkyl radical intermediate. **c**, Frustrated ion pairs as halogen-atom transfer reagents. ^a^Gas chromatography yield using *n*-dodecane as the internal standard. ^b^Isolated after hydroboration–oxidation treatment of the crude reaction mixture. Boc, *tert*-butyloxycarbonyl.

Using the insights gained from the mechanistic study, we were able to leverage this new reactivity beyond the coupling reaction in a series of preliminary results (Fig. 4). On the basis of the knowledge that the C–C bond is formed by the alkylation of a phosphorus ylide, we also devised a formal [1+*n*] annulation of *n*-alkylphosphonium salt **13** and 1,ω-dibromoalkane **14** (Fig. 4a). In this reaction, NaH-mediated phosphorus ylide alkylation occurs twice^49^, leading to the same *tert*-alkylphosphonium salt intermediate **3** as in the standard reaction that can then undergo C–P fragmentation in the presence of LiHMDS. Besides this two-electron process leveraging the phosphorus ylide reactivity, we also used the one-electron reactivity of the alkyl radical intermediate for further functionalization. First, we were able to intercept it with styrene **16** to construct a quaternary centre in **15**, forming two new C(*sp*^3^)–C(*sp*^3^) bonds in a single step (Fig. 4b). Similarly, the addition of heterocycle **17** to the standard conditions led to the formation of an additional C(*sp*^3^)–C(*sp*^2^) bond in product **18**. Leveraging the hydrogen-atom transfer step of the *tert*-alkyl radical with the phosphonium phenyl groups, the coupling of a phosphonium salt containing perdeuterated phenyl groups (**1-*d***_**15**_) led to the formation of the monodeuterated product **12m-*d***. The deuteration is regioselective for the position at which the phosphorus was bound in the starting material over multiple other sites that would be prone to deuteration reactions using conventional approaches^50^. In the described reactions, the radical arising from the key frustrated ion pair ultimately engages in product formation. We envisioned that this radical could also be used to mediate the formation of other open-shell intermediates through atom transfer processes, thereby extending the applicability of this chemistry. When the alkyl iodide **19** and alkene **20** were reacted in the presence of tetraphenylphosphonium bromide and LiHMDS, C–C bond formation occurred and product **21** was formed. We believe that this reaction could proceed through the formation of a frustrated ion pair between the PPh_4_ cation and the HMDS anion, which leads to a phenyl radical that can react in a halogen-atom transfer step with alkyl iodide **19**.

## Online content

Any methods, additional references, Nature Portfolio reporting summaries, source data, extended data, supplementary information, acknowledgements, peer review information; details of author contributions and competing interests; and statements of data and code availability are available at 10.1038/s41586-024-08195-1.

## Supplementary information

Supplementary Information Supplementary Information sections 1–8, Figs. 1–17, Tables 1–16 and references.

## Data Availability

All experimental, spectroscopic and computational data are available in the Supplementary Information. Crystallographic data are available from the Cambridge Crystallographic Data Centre (CCDC) with the following codes: CCDC 2254109 (**12f**) and CCDC 2254108 (**12m**).
