# Supplementary material for: Coupling of unactivated alkyl electrophiles using frustrated ion pairs
Source: Nature. 2024 Nov 20;636(8041):108–14. doi: 10.1038/s41586-024-08195-1 (PMC11618088; doi:10.1038/s41586-024-08195-1)
Supplement: Supplementary file 1 — Supplementary Information Supplementary Information sections 1–8, Figs. 1–17, Tables 1–16 and references. [file 41586_2024_8195_MOESM1_ESM.pdf]

nature portfolio

https://doi.org/10.1038/s41586-024-08195-1

Supplementary information

Coupling of unactivated alkyl electrophiles using frustrated ion pairs

In the format provided by the authors and unedited

Supplementary Information for

Coupling of Unactivated Alkyl Electrophiles Using Frustrated Ion Pairs

Sven Roediger, Emilien Le Saux, Philip Boehm, Bill Morandi

Corresponding author: bill.morandi@org.chem.ethz.ch

Table of contents

1. General Information 1
2. Reaction Optimization 3
2.1. Optimization of the coupling of unactivated alkyl halides with phosphonium salts 3
2.1.1. Evaluation of different bases and solvents 3
2.1.2. Evaluation of different reagent stoichiometry 4
2.1.3. Evaluation of different phosphonium halides and alkyl halides 5
2.1.4. Evaluation of different reaction temperatures and concentrations 6
2.1.5. Time profile of the reaction 7
2.1.6. Deviations from the optimized conditions 8
2.1.7. Effect of LiHMDS purity 9
3. Synthesis of Starting Materials 10
Commercially available starting materials 10
Cyclopentyltris(phenyl-d_5)phosphonium bromide (**1-d_{15}**) 10
(3-Iodopropyl)benzene (**2**) 11
Adamant-1-yltriphenylphosphonium bromide (**9**) 11
Adamant-1-yltris(phenyl-d_5)phosphonium bromide (**9-d_{15}**) 12
Cyclobutyltriphenylphosphonium bromide (**10v**) 12
(2-Norbornyl)triphenylphosphonium bromide (**10w**) 13
Cycloheptyltriphenylphosphonium bromide (**10y**) 14
Triphenyl(tetrahydro-2H-pyran-4-yl)phosphonium bromide (**10z**) 14
Triphenyl(5-phenylpentan-2-yl)phosphonium iodide (**10ab**) 15
(5-(4-Bromophenyl)pentan-2-yl)triphenylphosphonium iodide (**10ac**) 15
(3-Bromopropoxy)(*tert*-butyl)diphenylsilane (**S16**) 16
(5-((*tert*-Butyldiphenylsilyl)oxy)pentan-2-yl)triphenylphosphonium bromide (**10ad**) 17
(3-Iodoropropyl)(phenyl)sulfane (**S18**) 17
Triphenyl(5-(phenylthio)pentan-2-yl)phosphonium iodide (**10af**) 18
Phosphonium iodide **10ag** 18
(6-Iodohexyl)benzene (**11a**) 19
(4-Iodobutoxy)benzene (**11b**) 20
tert-Butyl(3-iodopropoxy)diphenylsilane (**11c**) 20
5-(3-iodopropyl)benzo[d][1,3]dioxole (**11d**) 21
(3-(Hydroxymethyl)piperidin-1-yl)(phenyl)methanone (**S25**) 21
(3-(Iodomethyl)piperidin-1-yl)(phenyl)methanone (**11e**) 22
3-(4-Iodophenyl)propan-1-ol (**S27**) 23
1-Iodo-4-(3-iodopropyl)benzene (**11g**) 23
1-Bromo-4-(3-iodopropyl)benzene (**11h**) 23
1-Chloro-4-(3-iodopropyl)benzene (**11i**) 24
(3-(4-Bromophenyl)propoxy)(*tert*-butyl)dimethylsilane (**S30**) 24

Silylether **S31** ... 25
Alcohol **S32** ... 25
2-(4-(3-iodopropyl)phenyl)-4,4,5,5-tetramethyl-1,3,2-dioxaborolane (**11j**) ... 26
10-(4-Chlorobutyl)-10*H*-phenoxazine (**S34**) ... 26
10-(4-Iodobutyl)-10*H*-phenoxazine (**11k**) ... 27
1-(4-Bromobutyl)-2-phenyl-1H-indole (**11l**) ... 27
2-(4-(Iodomethyl)piperidin-1-yl)pyrimidine (**11m**) ... 28
1-Iodo-4-(2-iodoethyl)benzene (**11n**) ... 29
(4-(Hexyloxy)phenyl)methanol (**S39**) ... 29
1-(Bromomethyl)-4-(hexyloxy)benzene (**11o**) ... 30
4-(3-iodopropyl)phenol (**11p**) ... 30
4-(4-Bromobutyl)benzoic acid (**11q**) ... 31
5-(4-Bromobutoxy)-1*H*-pyrrolo[2,3-b]pyridine (**11r**) ... 31
Alkyl chloride **S44** ... 32
Alkyl iodide **11s** ... 32
Alkyl bromide **11t** ... 33
Alkyl bromide **11u** ... 34
Triphenyl(4-phenylbutyl)phosphonium bromide (**13**) ... 34
Cyclopentyltriphenylphosphonium iodide (**S1**) ... 35
3-Phenylpropyl 4-methylbenzenesulfonate (**S3**) ... 35
4. Product characterization ... 37
General procedure for the reaction of phosphonium salts with alkyl halides (GP1) ... 37
General procedure for the reaction of phosphonium salts with alkyl halides including a hydroboration-oxidation post-treatment (GP2) ... 37
1-(3-Cyclopentylpropyl)-4-benzene (**6**) ... 38
(6-Cyclopentylhexyl)benzene (**12a**) ... 38
(4-Cyclopentylbutoxy)benzene (**12b**) ... 39
tert-Butyl(3-cyclopentylpropoxy)diphenylsilane (**12c**) ... 39
5-(3-cyclopentylpropyl)benzo[d][1,3]dioxole (**12d**) ... 39
Amide **12e** ... 40
tert-Butyl 4-(cyclopentylmethyl)piperidine-1-carboxylate (**12f**) ... 40
1-(3-Cyclopentylpropyl)-4-iodobenzene (**12g**) ... 43
1-Bromo-4-(3-cyclopentylpropyl)benzene (**12h**) ... 43
1-Chloro-4-(3-cyclopentylpropyl)benzene (**12i**) ... 43
Boronic ester **12j** ... 44
10-(4-Cyclopentylbutyl)-10H-phenoxazine (**12k**) ... 44
1-(4-Cyclopentylbutyl)-2-phenyl-1H-indole (**12l**) ... 45
2-(4-(Cyclopentylmethyl)piperidin-1-yl)pyrimidine (**12m**) ... 45
1-(4-Iodophenyl)spiro[2.4]heptane (**12n**) ... 47

1-(Cyclopentylmethyl)-4-(hexyloxy)benzene (**12o**) 47
4-(3-Cyclopentylpropyl)phenol (**12p**) 47
4-(4-Cyclopentylbutyl)benzoic acid (**12q**) 48
5-(4-Cyclopentylbutoxy)-1*H*-pyrrolo[2,3-b]pyridine (**12r**) 48
Alcohol **12s** 49
Oxazolidinone **12t** 50
Pyridine **12u** 50
(6-Cyclobutylhexyl)benzene (**12v**) 51
2-(6-Phenylhexyl)norbornane (**12w**) 51
(6-Cyclohexylhexyl)benzene (**12x**) 52
(6-Phenylhexyl)cycloheptane (**12y**) 52
Ether **12z** 52
(7-Methyloctyl)benzene (**12aa**) 53
(4-Methylheptane-1,7-diyl)dibenzene (**12ab**) 53
1-Bromo-4-(4-methyl-7-phenylheptyl)benzene **(12ac)** 54
tert-Butyl((4-methyl-7-phenylheptyl)oxy)diphenylsilane (**12ad**) 54
tert-Butyl((4-methylpentyl)oxy)diphenylsilane (**12ae**) 55
5-((5-Methyl-8-(phenylthio)octyl)oxy)-1H-pyrrolo[2,3-b]pyridine (**12af**) 55
Pyrimidine **12ag** 56
5. Further experiments 57
5.1. Cyclization reaction of phosphonium salts and alkyl bis-halides 57
5.2. Formation of quaternary centers 57
5.3. Formation of a selectively deuterated product 58
5.4. Frustrated ion pairs as halogen-atom abstraction reagents 59
5.4.1. Observation of dehalogenation of an alkyl iodide 59
5.4.2. Yield of the dehydrohalogenation 59
5.4.3. Dehalogenative coupling 60
5.5. Reaction using a LiHMDS solution 61
5.6. Catalytic reaction 61
5.7. Reactions of further substrates 62
5.8. Attempted hydrolysis of *tert*-alkylphosphonium salts 62
5.9. Additive compatibility screen 63
6. Mechanistic experiments 65
6.1. Identification of the major phosphorus-containing byproduct 65
6.2. Test for base-mediated cyclization of triphenylphosphine (**5**) 66
6.3. Synthesis and reactivity of the alpha-tertiary phosphonium salt **3** 66
6.3.1. Preparation of phosphonium salt **3** 66
6.3.2. Reactivity of phosphonium salt **3** 67
6.4. NMR studies 67

6.5. Reaction of a phosphonium salt containing a tertiary alkyl group 68
6.6. Reaction inhibition by TEMPO 69
6.7. Isolation of TEMPO adduct **7** 69
6.8. Deuteration experiments 70
6.8.1. Reaction using a deuterated substrate 70
6.8.2. Reaction using deuterated solvent 71
6.9. Investigation of ion effects 71
6.10. UV/Vis spectroscopy 72
7. Computational studies 73
7.1. Computational details 73
7.2. Discussion of the free energy diagram of the reaction 74
7.2.1. Free energy diagram of the reaction 74
7.2.2. D<iscussion of the SET step in the frustrated ion pair **B** 75
7.2.3. Discussion of the regioselectivity of the HAT step 78
7.2.4. Discussion of the cyclization step 79
7.3. Effect of the base 79
7.4. Energies of calculated structures 83
7.5. Coordinates of calculated structures 84
NMR Spectra 94
8. References 195

1. General Information

All air-sensitive manipulations were performed under an inert atmosphere in an argon-filled glovebox (LABmaster Pro SP, MBraun) or by standard Schlenk techniques. LiHMDS was purchased from Acros Organics. It was used as received and stored in an argon-filled glovebox. Commercial phosphonium salts were dried in an oven (120 °C overnight) and stored under argon in a glovebox. All mentions of dioxane refer to 1,4-dioxane. Dioxane used in the coupling reactions was degassed, passed through an activated alumina column, and stored over molecular sieves in an argon-filled glovebox. Other compounds were used as supplied from commercial sources if not stated otherwise.

Proton nuclear magnetic resonance (^{1}H NMR) spectra were acquired on commercial 400 MHz and 500 MHz instruments at the NMR facility of ETH Zürich. Quantitative ^{1}H NMR (^{1}H qNMR) spectra were acquired using a D1 time of 60 s. Carbon-13 nuclear magnetic resonance ($^{13}C\{^{1}H\}$ NMR) spectra were acquired at 101 or 126 MHz. Fluorine-19 nuclear magnetic resonance ($^{19}F\{^{1}H\}$ NMR) spectra were acquired at 376 MHz. Phosphorus-31 nuclear magnetic resonance ($^{31}P\{^{1}H\}$ NMR) spectra were acquired at 162 MHz. Quantitative $^{31}P\{^{1}H\}$ NMR ($^{31}P\{^{1}H\}$ qNMR) were acquired with inverse-gated decoupling (O1P = 0 ppm, D1 = 20 s) at 162 MHz. Boron-11 nuclear magnetic resonance (^{11}B NMR) spectra were acquired at 128 MHz. The proton signal for the residual non-deuterated solvent (δ 7.26 ppm for $CDCl_3$) was used as an internal reference for ^{1}H NMR spectra. For $^{13}C\{^{1}H\}$ NMR spectra, chemical shifts are reported relative to the δ 77.16 ppm resonance of $CDCl_3$. Coupling constants are reported in Hz. Multiplicities are indicated by s (singlet), d (doublet), t (triplet), q (quartet), pent (quintet), h (sextet), hept (heptet), m (multiplet), and br (broad) or a combination thereof.

Gas chromatography (GC) measurements were conducted on a Shimadzu GC-2025 Series GC system. GC-MS spectra were obtained on an instrument containing a Shimadzu GC-2010 Plus GC system and a Shimadzu GC-MS QP 2020 system. Calibration curves using *n*-dodecane as internal standard were used to determine GC yields. GC-MS spectra were obtained on an instrument containing a Shimadzu GC-2010 Plus GC system and a Shimadzu GC-MS QP 2020 system.

High-resolution mass spectra were provided by the mass spectrometry service facility in the Laboratories of Organic Chemistry at ETH Zürich. The data was obtained using electron ionisation (EI) on a Thermo scientific Q Exactive GC Orbitrap with direct Probe or electrospray ionisation (ESI) on a Bruker maXis – ESI-Qq-TOF-MS and are reported in m/z.

Analytical thin-layer chromatography was performed on pre-coated, glass-backed silica gel plates (Merck, 0.25 mm silica gel Si 60, F254). Visualization of the developed chromatogram was either performed by UV absorbance at a wavelength of λ = 254 nm or $KMnO_4$ stain. Flash column chromatography was performed on a Biotage Isolera One system with Sfär columns or manually using silica gel 60 (particle size 40 – 63 μm, Silicycle). $AgNO_3$-impregnated silica was prepared by suspending silica gel 60 (250 g) in a solution of $AgNO_3$ (27.5 g) in acetonitrile (600 mL). The suspension was shaken for mixing, and acetonitrile was removed under reduced pressure to yield the $AgNO_3$-impregnated silica (ca. 10 wt%) as an off-white solid.

Preparative HPLC separations were carried out on an Agilent 1260 Infinity II system (C18 5u, 250x21 mm) with reverse phase conditions using gradients of acetonitrile in water (0.1% trifluoroacetic acid).

Single crystalline samples were measured on a Rigaku Oxford Diffraction XtaLAB Synergy-S Dualflex kappa diffractometer equipped with a Dectris Pilatus 300 HPAD detector and using microfocus sealed tube Cu-Kα radiation with mirror optics (λ = 1.54178 Å). All measurements were carried out at 100 K (unless otherwise noted) using an Oxford Cryosystems Cryostream 800 sample cryostat. Data collected on the

Rigaku instrument were integrated using CrysAlisPro and corrected for absorption effects using a combination of empirical (ABSPACK) and numerical corrections. The structures were solved using SHELXT[1] or SHELXS[2] and refined by full-matrix least-squares analysis (SHELXL),[3] using the program package OLEX2.[4] Unless otherwise indicated below, all non-hydrogen atoms were refined anisotropically and hydrogen atoms were constrained to ideal geometries and refined with fixed isotropic displacement parameters (in terms of a riding model).

Ultraviolet-visible (UV/Vis) spectroscopy was measured on an Agilent Cary 60 instrument (instrument version 2.00) in dual beam mode with a scan rate of 300 nm/min and a data interval of 0.5 nm.

2. Reaction Optimization

2.1. Optimization of the coupling of unactivated alkyl halides with phosphonium salts

2.1.1. Evaluation of different bases and solvents

Inside a glovebox filled with argon, a 4-mL screw-cap vial was subsequently charged with cyclopentyltriphenylphosphonium bromide (**1**) (41.1 mg, 0.1 mmol, 1.0 equiv.), base (0.2 mmol, 2.0 equiv.), 1-iodo-3-phenylpropane (**2**) (16.0 µL, 0.1 mmol, 1.0 equiv.), and solvent (0.5 mL). The vial was capped, taken out of the glovebox, and stirred in a pre-heated heating block at 80 °C for 22 hours. After cooling to room temperature, *n*-dodecane (15 µL) was added as an internal standard, the mixture was shaken, and a sample was taken for GC analysis.

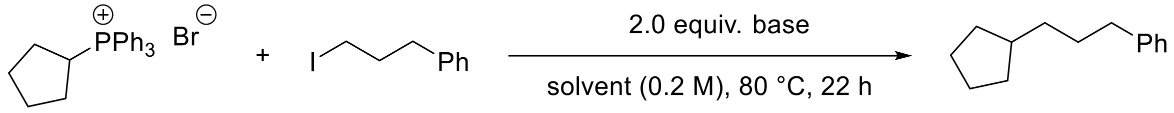

1 **2** 1.0 equiv. **6**

Table S1. Evaluation of different bases and solvents.

Entry	base	solvent	Yield (%)[a]
1	DABCO	dioxane	0
2	NaOMe	dioxane	trace
3	NaOAc	dioxane	0
4	K_3PO_4	dioxane	0
5	KO*t*Bu	dioxane	18
6	LDA	dioxane	19
7	$LiNMe_2$	dioxane	24
8	KHMDS	dioxane	17
**9**	**LiHMDS**	**dioxane**	**48**
10	LiHMDS	toluene	16
11	LiHMDS	DMA	14
12	LiHMDS	ACN	trace

a: Yield determined by GC using *n*-dodecane as an internal standard.

2.1.2. Evaluation of different reagent stoichiometry

Inside a glovebox filled with argon, a 4-mL screw-cap vial was subsequently charged with cyclopentyltriphenylphosphonium bromide (**1**) (X equiv.), LiHMDS (Z equiv.), 1-iodo-3-phenylpropane (**2**) (Y equiv.), and dioxane (0.5 mL). The vial was capped, taken out of the glovebox, and stirred in a pre-heated heating block at 80 °C for 22 hours. After cooling to room temperature, *n*-dodecane (15 µL) was added as an internal standard, the mixture was shaken, and a sample was taken for GC analysis.

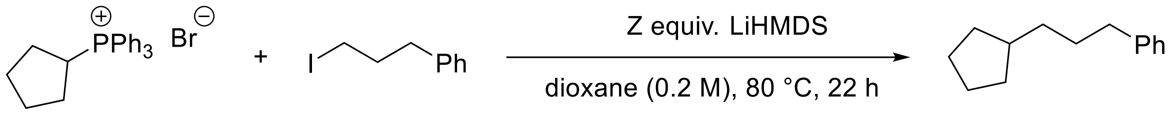

1
X equiv.

2
Y equiv.

6

Table S2. Evaluation of different reagent stoichiometry.

Entry	X	Y	Z	Yield (%)[a]
1	1.0	1.0	2.0	48
2	1.0	1.1	2.0	53
3	1.0	1.5	2.0	46
4	1.0	1.0	2.5	53
5	1.0	1.1	2.5	68
**6**	**1.0**	**1.5**	**2.5**	**85**
7	1.5	1.0	2.5	44
8	1.5	1.0	3.0	46

a: Yield determined by GC using *n*-dodecane as an internal standard.

2.1.3. Evaluation of different phosphonium halides and alkyl halides

Inside a glovebox filled with argon, a 4-mL screw-cap vial was subsequently charged with cyclopentyltriphenylphosphonium halide (0.1 mmol, 1.0 equiv.), LiHMDS (41.8 mg, 0.25 mmol, 2.5 equiv.), alkyl halide (0.15 mmol, 1.5 equiv.), and dioxane (0.5 mL). The vial was capped, taken out of the glovebox, and stirred in a pre-heated heating block at 80 °C for 22 hours. After cooling to room temperature, *n*-dodecane (15 μL) was added as an internal standard, the mixture was shaken, and a sample was taken for GC analysis.

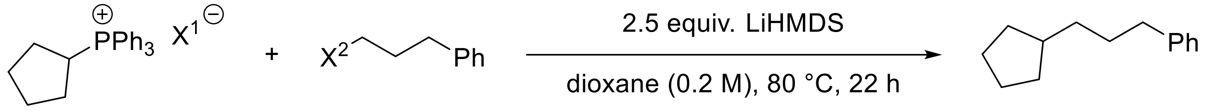

1.5 equiv.

6

1: X^1 = Br
S1: X^1 = I

2: X^2 = I
S2: X^2 = Br
S3: X^2 = OTs
S4: X^2 = Cl

Table S3. Evaluation of different phosphonium halides and alkyl halides.

Entry	X^1	X^2	Yield (%)[a]
**1**	**Br**	**I**	**85**
2	Br	Br	76
3	Br	OTs	62
4	Br	Cl	32
5	I	I	75
6	I	Br	81
7	I	OTs	69
8	I	Cl	44

a: Yield determined by GC using *n*-dodecane as an internal standard.

2.1.4. Evaluation of different reaction temperatures and concentrations

Inside a glovebox filled with argon, a 4-mL screw-cap vial was subsequently charged with cyclopentyltriphenylphosphonium bromide (**1**) (41.1 mg, 0.1 mmol, 1.0 equiv.), LiHMDS (41.8 mg, 0.25 mmol, 2.5 equiv.), 1-iodo-3-phenylpropane (**2**) (24.1 μL, 0.15 mmol, 1.5 equiv.), and dioxane. The vial was capped, taken out of the glovebox, and stirred in a pre-heated heating block at the indicated temperature for 22 hours. After cooling to room temperature, *n*-dodecane (15 μL) was added as an internal standard, the mixture was shaken, and a sample was taken for GC analysis.

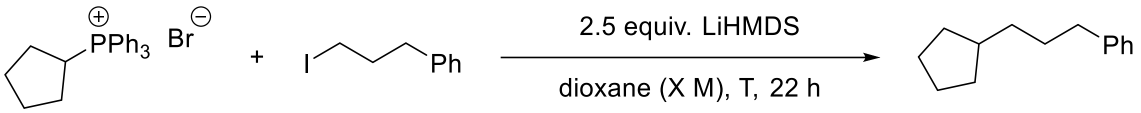

1 **2** 1.5 equiv. **6**

Table S4. Evaluation of different reaction temperatures and concentrations.

Entry	X	T	Yield (%)[a]
1	0.2	80	85
2	0.2	60	15
3	0.2	100	83
4	1.0	80	17
5	0.5	80	36
**6**	**0.1**	**80**	**96**
7	0.05	80	93

a: Yield determined by GC using *n*-dodecane as an internal standard.

2.1.5. <u>Time profile of the reaction</u>

Inside a glovebox filled with argon, a 4-mL screw-cap vial was subsequently charged with cyclopentyltriphenylphosphonium bromide (**1**) (41.1 mg, 0.1 mmol, 1.0 equiv.), LiHMDS (41.8 mg, 0.25 mmol, 2.5 equiv.), 1-iodo-3-phenylpropane (**2**) (24.1 μL, 0.15 mmol, 1.5 equiv.), and dioxane (1.0 mL). The vial was capped, taken out of the glovebox, and stirred in a pre-heated heating block at 80 °C for the indicated time. After cooling to room temperature, *n*-dodecane (15 μL) was added as an internal standard, the mixture was shaken, and a sample was taken for GC analysis. The different time points were obtained from individual reactions.

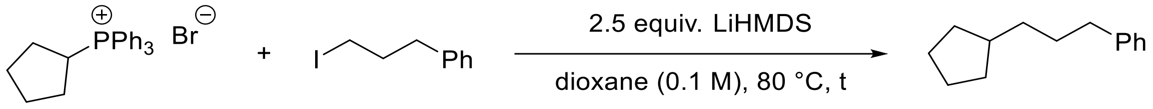

1 **2** **6**

1.5 equiv.

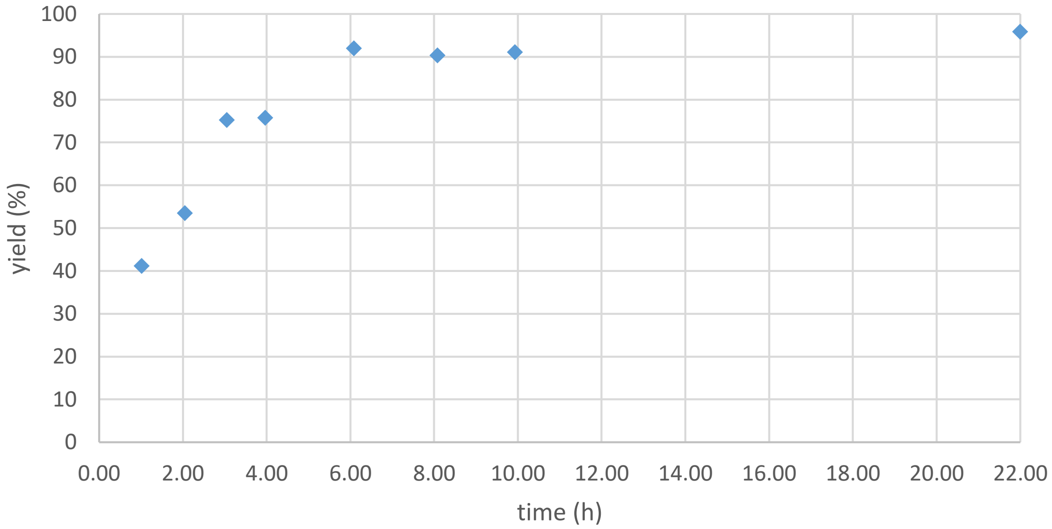

Figure S1. Time profile of the reaction.

The model reaction is completed after ca. six hours. To account for substrates that might react more slowly, it was decided to heat the reactions for 16 hours under the standard conditions.

2.1.6. Deviations from the optimized conditions

Inside a glovebox filled with argon, a 4-mL screw-cap vial was subsequently charged with cyclopentyltriphenylphosphonium bromide (**1**) (41.1 mg, 0.1 mmol, 1.0 equiv.), LiHMDS (41.8 mg, 0.25 mmol, 2.5 equiv.), 1-iodo-3-phenylpropane (**2**) (24.1 µL, 0.15 mmol, 1.5 equiv.), and dioxane (1.0 mL). The vial was capped, taken out of the glovebox, and stirred in a pre-heated heating block at 80 °C for 16 hours. After cooling to room temperature, *n*-dodecane (15 µL) was added as an internal standard, the mixture was shaken, and a sample was taken for GC analysis.

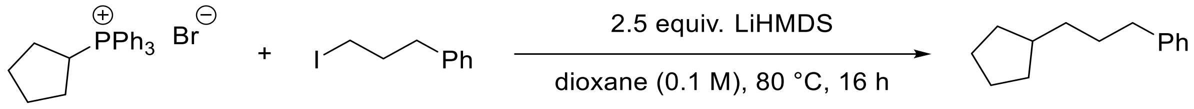

1 **2** 1.5 equiv. **6**

Table S5. Deviations from the optimized conditions.

Entry	Deviation from above	Yield (%)[a]
1	none	97
2	**S2** (alkyl bromide) instead of **2**	79
3	**S3** (alkyl tosylate) instead of **2**	62
4	**S4** (alkyl chloride) instead of **2**	25
5	**S1** (phosphonium iodide) instead of **1**	96
6	2.0 equiv. LiHMDS	53
7	1.1 equiv. **2**	78
8	1.0 equiv. **2**	58
9	60 °C	22
10	1.5 equiv. **1**, 1.0 equiv. **2**	44
11	dioxane (0.2 M)	87
12	KHMDS instead of LiHMDS	18
13	NaHMDS instead of LiHMDS	43
14	LDA instead of LiHMDS	20
15	$LiNMe_2$ instead of LiHMDS	24
16	NaH instead of LiHMDS	traces
17	*n*-BuLi (1.6 M in hexanes) instead of LiHMDS	traces
18	Reaction stirred in the dark[b]	quant.
19	Reaction run under air[c]	17

a: Yield determined by GC using *n*-dodecane as an internal standard. b: The reaction was covered with aluminum foil to avoid exposure to light. c: The reaction was set up under inert atmosphere and then opened to air for 5 minutes before the vial was closed and heated.

2.1.7. <u>Effect of LiHMDS purity</u>

Inside a glovebox filled with argon, a 100-mL Schlenk flask was charged with commercial LiHMDS (95% grade). The flask was capped with a cold finger trap and removed from the glovebox. The cold finger trap was filled with crushed dry ice, and the flask was put under dynamic vacuum (ca. 1 mbar). The bottom of the flask was gently heated with a heat gun until the LiHMDS sublimed on the cold finger as a white solid.

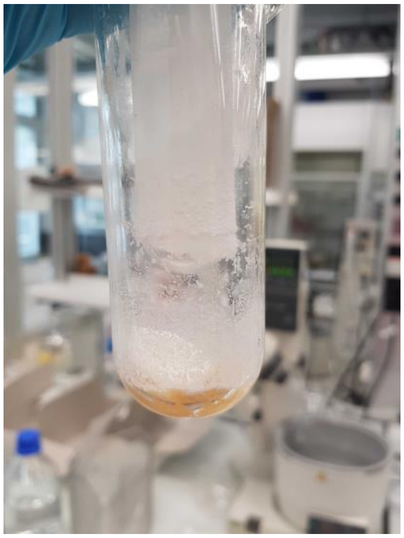

Figure S2. Sublimation of LiHMDS showing the commercial grade material (yellow) at the bottom and the sublimed material (white) at the top.

Inside a glovebox filled with argon, a 4-mL screw-cap vial was subsequently charged with cyclopentyltriphenylphosphonium bromide (**1**) (41.1 mg, 0.1 mmol, 1.0 equiv.), LiHMDS (41.8 mg, 0.25 mmol, 2.5 equiv.), 1-iodo-3-phenylpropane (**2**) (24.1 µL, 0.15 mmol, 1.5 equiv.), and dioxane (1.0 mL). The vial was capped, taken out of the glovebox, and stirred in a pre-heated heating block at 80 °C for the indicated time. After cooling to room temperature, *n*-dodecane (15 µL) was added as an internal standard, the mixture was shaken, and a sample was taken for GC analysis. The different time points were obtained from individual reactions. Reactions at the same time points were set up in parallel.

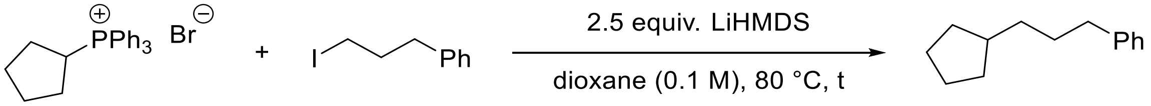

1 **2** **6**

1.5 equiv.

Table S6. Effect of LiHMDS purity.

Entry	LiHMDS purity	Time (h)	Yield (%)[a]
1	sublimed	0.25	25
2	commercial (95% grade)	0.25	28
3	sublimed	6	91
4	commercial (95% grade)	6	94
5	sublimed	16	99[b]
6	commercial (95% grade)	16	97

a: Yield determined by GC using *n*-dodecane as an internal standard. b: A new, previously unused stir bar was used for the reaction.

3. Synthesis of Starting Materials

Commercially available starting materials

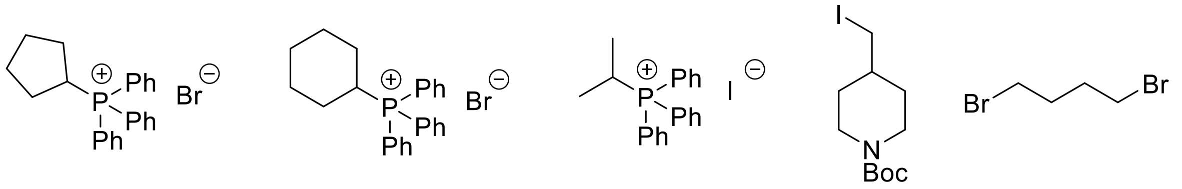

1 **10x** **10aa** **11f** **14**

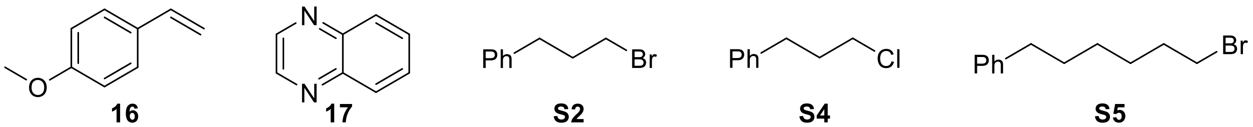

16 **17** **S2** **S4** **S5**

Figure S3. Commercially available starting materials.

The phosphonium salts **1**, **10x**, and **10aa** are commercially available. They were dried at 120 °C overnight and stored under inert atmosphere before use. The alkyl halides **11f**, **14**, **S2**, **S4**, and **S5** as well as the alkene **16** and quinoxaline (**17**) are commercially available and were used as received.

Cyclopentyltris(phenyl-*d*₅)phosphonium bromide (1-*d*₁₅)

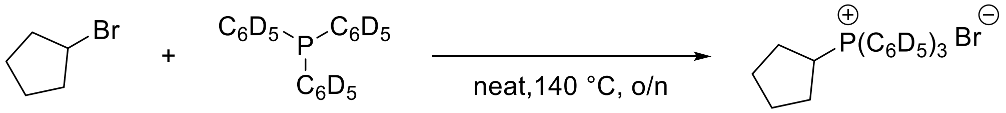

S6 **5-d_{15}** **1-d_{15}**

Under an atmosphere of nitrogen, an 8-mL screw-cap vial equipped was charged with bromocyclopentane (**S6**) (320 µL, 3.0 mmol, 3.0 equiv.) and triphenylphosphine-d_{15} (**5-d_{15}**) (277 mg, 1.0 mmol, 1.0 equiv.). The mixture was heated to 140 °C overnight (ca. 14 h). After cooling to room temperature, the reaction mixture had solidified and was triturated three times with toluene to yield the title compound as a white solid (419 mg, 98%).

^{1}H NMR (400 MHz, $CDCl_3$) δ = 5.29 – 5.12 (*pseudo*-h, *J* = 8.7 Hz, 1H), 2.56 – 2.39 (m, 2H), 1.87 – 1.69 (m, 2H), 1.66 – 1.47 (m, 2H), 1.30 – 1.13 (m, 2H) ppm.

^{13}C{^{1}H} NMR (101 MHz, $CDCl_3$) δ = 134.7 – 133.9 (m), 133.4 (td, *J* = 24.5, 8.4 Hz), 129.9 (td, *J* = 25.1, 12.6 Hz), 118.6 (d, *J* = 84.3 Hz), 29.4 (d, *J* = 49.2 Hz), 28.1, 26.5 (d, *J* = 8.8 Hz) ppm.

^{31}P{^{1}H} NMR (162 MHz, $CDCl_3$) δ = 30.9 ppm.

HRMS (ESI+): m/z for $C_{23}H_9D_{15}P$ [M-Br]$^+$ calcd.: 346.2552, found: 346.2554.

(3-Iodopropyl)benzene (2)

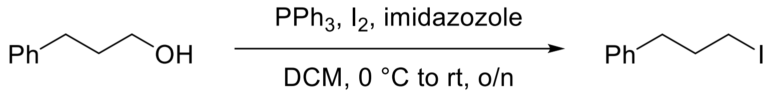

S7 **2**

To a 250-mL two-neck flask under nitrogen were added triphenylphosphine (**5**) (9.5 g, 36 mmol, 1.2 equiv.) and imidazole (2.7 g, 39 mmol, 1.3 equiv.). DCM (100 mL) was added, and the mixture was cooled to 0 °C. Then, iodine (9.1 g, 36 mmol, 1.2 equiv.) was added, and the mixture was stirred at 0 °C for 15 minutes. 3-Phenylpropan-1-ol (**S7**) (4.1 mL, 30 mmol, 1.0 equiv.) was added at 0 °C. The reaction mixture was allowed to warm to room temperature overnight. Then, a saturated aqueous solution of sodium thiosulfate was added, and the phases were separated. The aqueous layer was extracted with DCM, and the combined organic layers were dried with magnesium sulfate. Volatile materials were evaporated under reduced pressure, and the crude product was purified by column chromatography (cyclohexane) to yield the title compound as a colorless oil (6.1 g, 83%).

^{1}H NMR (400 MHz, $CDCl_3$) δ = 7.33 – 7.27 (m, 2H), 7.24 – 7.18 (m, 3H), 3.18 (t, *J* = 6.8 Hz, 2H), 2.74 (t, *J* = 7.3 Hz, 2H), 2.14 (p, *J* = 7.1 Hz, 2H) ppm.

^{13}C{^{1}H} NMR (101 MHz, $CDCl_3$) δ = 140.6, 128.7, 128.6, 126.3, 36.4, 35.0, 6.5 ppm.

The spectroscopic data matched those reported in the literature.[5]

Adamant-1-yltriphenylphosphonium bromide (9)

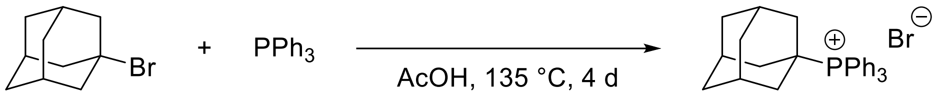

S8 **5** **9**

Under an atmosphere of nitrogen, a 250-mL round-bottom flask equipped with a reflux condenser was charged with 1-bromoadamantane (**S8**) (12.0 g, 56.0 mmol, 1.4 equiv.) and triphenylphosphine (**5**) (10.4 g, 40.0 mmol, 1.0 equiv.). Glacial acetic acid (80 mL) was added, and the mixture was heated under reflux (oil bath set to 135 °C) for 4 days. After cooling to room temperature, diethyl ether (ca. 150 mL) was added. The suspension was filtered, and the residue was washed with diethyl ether. The crude product was recrystallized from hot ethanol to yield the title compound[6] as a white solid (15.4 g, 81%) after prolonged drying at elevated temperature.

^{1}H NMR (400 MHz, $CDCl_3$) δ = 7.82 – 7.74 (m, 3H), 7.69 (td, *J* = 7.8, 3.5 Hz, 6H), 7.61 – 7.51 (m, 6H), 2.06 (d, *J* = 5.6 Hz, 9H), 1.71 (*pseudo*-q, *J* = 11.9 Hz, 6H) ppm.

^{13}C{^{1}H} NMR (101 MHz, $CDCl_3$) δ = 135.2 (d, *J* = 3.0 Hz), 134.4 (d, *J* = 8.6 Hz), 130.7 (d, *J* = 11.7 Hz), 116.2 (d, *J* = 79.7 Hz), 39.1 (d, *J* = 40.2 Hz), 37.6 (d, *J* = 2.5 Hz), 35.3 (d, *J* = 2.1 Hz), 27.7 (d, *J* = 10.3 Hz) ppm.

^{31}P{^{1}H} NMR (162 MHz, $CDCl_3$) δ = 28.8 ppm.

HRMS (ESI+): m/z for $C_{28}H_{30}P$ $[M-Br]^+$ calcd.: 397.2080, found: 397.2072.

Adamant-1-yltris(phenyl-d_5)phosphonium bromide (9-d_{15})

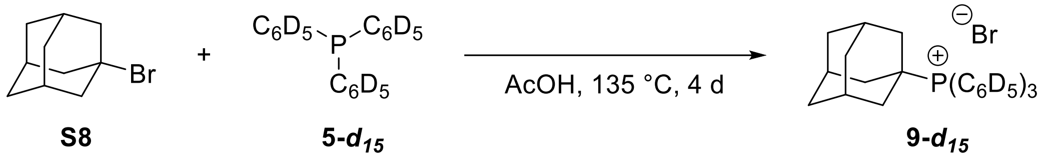

Under an atmosphere of nitrogen, a 25-mL round-bottom flask equipped with a reflux condenser was charged with 1-bromoadamantane (**S8**) (602 mg, 2.8 mmol, 1.4 equiv.) and triphenylphosphine-d_{15} (**5-d_{15}**) (555 mg, 2.0 mmol, 1.0 equiv.). Glacial acetic acid (4.0 mL) was added, and the mixture was heated under reflux (oil bath set to 135 °C) for 4 days. After cooling to room temperature, ethyl acetate (ca. 20 mL) was added. The suspension was filtered, and the residue was washed with ethyl acetate. The title compound was obtained as a white solid (865 g, 88%) after prolonged drying at elevated temperature.

^{1}H NMR (400 MHz, $CDCl_3$) δ = 2.15 – 1.88 (d, *J* = 5.5 Hz, 9H), 1.86 – 1.74 (m, 6H) ppm.

^{13}C{^{1}H} NMR (101 MHz, $CDCl_3$) δ = 135.2 – 134.4 (m), 134.1 (td, *J* = 24.4, 8.1 Hz), 130.3 (td, *J* = 25.1, 11.8 Hz), 116.0 (d, *J* = 79.5 Hz), 39.2 (d, *J* = 40.5 Hz), 37.7 (d, *J* = 2.5 Hz), 35.4 (d, *J* = 2.0 Hz), 27.8 (d, *J* = 10.3 Hz) ppm.

^{31}P{^{1}H} NMR (162 MHz, $CDCl_3$) δ = 28.7 ppm.

HRMS (ESI+): m/z for $C_{28}H_{15}D_{15}P$ [M-Br]$^+$ calcd.: 412.3021, found: 412.3017.

Cyclobutyltriphenylphosphonium bromide (10v)

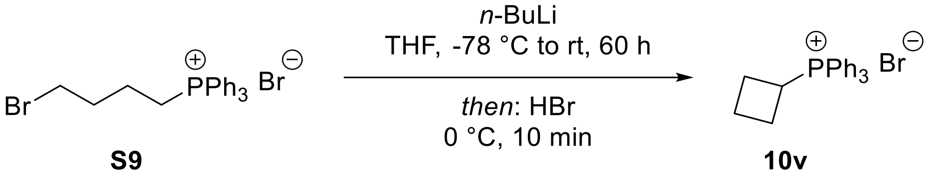

A 100-mL round-bottom flask under nitrogen was charged with (4-bromobutyll)triphenylphosphonium bromide (**S9**) (4.8 g, 10.0 mmol, 1.0 equiv.) and THF (20 mL). The mixture was cooled to -78 °C, and *n*-butyllithium (1.6 M in hexanes, 13.8 mL, 22.0 mmol, 2.2 equiv.) was added slowly. The reaction mixture was allowed to slowly warm to room temperature and stirred for 60 hours (over weekend). The dark red solution was treated with concentrated aqueous HBr (ca. 6 mL) at 0 °C until discoloration was observed. The layers were separated, and the aqueous layer was extracted three times with DCM. The combined organic layers were washed with saturated aqueous $NaHCO_3$ and then dried with magnesium sulfate. Volatile materials were removed under reduced pressure, and the crude product was recrystallized from hot acetonitrile to yield the product as a white solid (490 mg, 12%).

^{1}H NMR (400 MHz, $CDCl_3$) δ = 7.76 – 7.67 (m, 9H), 7.66 – 7.60 (m, 6H), 5.67 – 5.46 (m, 1H), 2.79 (dtd, *J* = 10.5, 8.7, 5.5 Hz, 2H), 2.40 (dq, *J* = 17.6, 8.7 Hz, 1H), 2.17 – 1.97 (m, 2H), 1.64 – 15.1 (m, 1H) ppm.

^{13}C{^{1}H} NMR (101 MHz, $CDCl_3$) δ = 135.0 (d, *J* = 3.0 Hz), 133.7 (d, *J* = 9.5 Hz), 130.5 (d, *J* = 12.3 Hz), 117.9 (d, *J* = 85.2 Hz), 25.4 (d, *J* = 45.0 Hz), 23.2 (d, *J* = 5.5 Hz), 20.4 (d, *J* = 16.9 Hz) ppm.

$^{31}P\{^1H\}$ NMR (162 MHz, $CDCl_3$) δ = 24.8 ppm.

HRMS (ESI+): m/z for $C_{22}H_{22}P$ [M-Br]$^+$ calcd.: 317.1454, found: 317.1453.

(2-Norbornyl)triphenylphosphonium bromide (10w)

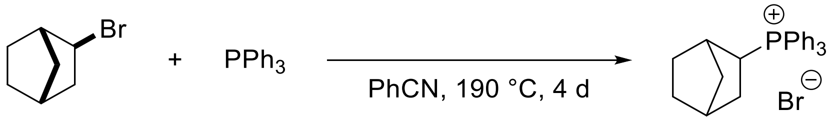

S10 **5** **10w**

A 16-mL screw-cap vial was charged with triphenylphosphine (**5**) (790 mg, 3.0 mmol, 1.0 equiv.), *exo*-2-bromonorbornane (**S10**) (460 µL, 3.6 mmol, 1.2 equiv.), and benzonitrile (0.6 mL). The vial was capped, and the reaction mixture was stirred at 190 °C for 4 days. After cooling to room temperature, the reaction mixture was triturated with diethyl ether until the solution was colorless (5 times). The residue was dissolved in DCM and filtered through a silica plug, first eluting with DCM and then with 15% MeOH in DCM. The latter fraction was collected and dried to yield the title compound as a 9:1 mixture of diastereomers (766 mg, 58%).

The characterization data for the major isomer are reported here:

^1H NMR (400 MHz, $CDCl_3$) δ = 7.94 – 7.86 (m, 6H), 7.74 – 7.68 (m, 3H), 7.68 – 7.62 (m, 6H), 4.84 (q, *J* = 9.0 Hz, 1H), 2.59 – 2.50 (m, 1H), 2.37 – 2.27 (m, 2H), 2.20 (br, 1H), 1.78 – 1.69 (m, 1H), 1.68 – 1.50 (m, 3H), 0.86 (d, *J* = 10.3 Hz, 1H), -0.02 (d, *J* = 10.4 Hz, 1H) ppm.

$^{13}C\{^1H\}$ NMR (101 MHz, $CDCl_3$) δ = 134.8 (d, *J* = 3.0 Hz), 134.0 (d, *J* = 9.5 Hz), 130.5 (d, *J* = 12.1 Hz), 118.9 (d, *J* = 84.1 Hz), 39.5, 37.4, 36.4 (d, *J* = 4.0 Hz), 33.1 (d, *J* = 4.8 Hz), 31.4 (d, *J* = 3.8 Hz), 31.3 (d, *J* = 57.0 Hz), 27.8 ppm.

$^{31}P\{^1H\}$ NMR (162 MHz, $CDCl_3$) δ = 27.0 ppm.

HRMS (ESI+): m/z for $C_{25}H_{26}P$ [M-Br]$^+$ calcd.: 357.1767, found: 357.1766.

The minor isomer has a $^{31}P\{^1H\}$ NMR shift of 25.3 ppm. Its ^1H NMR signals largely overlap with the major isomer. Distinct peaks are visible at 7.85–7.79 ppm (m), 3.01 ppm (br), 1.44–1.19 ppm (m), and 0.61–0.53 ppm (m).

The diastereomeric ratio was determined by $^{31}P\{^1H\}$ qNMR spectroscopy. Comparison of the spectroscopic data with literature values indicated that the major isomer is the *exo*-isomer.[7]

Cycloheptyltriphenylphosphonium bromide (10y)

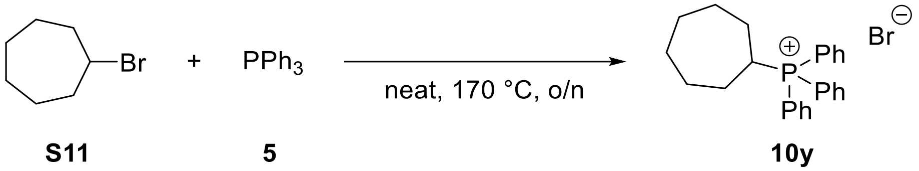

Under an atmosphere of nitrogen, a 16-mL vial was charged with triphenylphosphine (**5**) (790 mg, 3.0 mmol, 1.0 equiv.) and bromocycloheptane (**S11**) (1.2 mL, 9.0 mmol, 3.0 equiv.). The mixture was heated to 170 °C overnight. After cooling to room temperature, the reaction mixture was triturated three times with diethyl ether to yield the product as a white solid (1.09 g, 83%).

^{1}H NMR (400 MHz, $CDCl_3$) δ = 7.94 – 7.86 (m, 6H), 7.75 – 7.69 (m, 3H), 7.69 – 7.63 (m, 6H), 4.81 (dtt, *J* = 13.6, 10.0, 3.6 Hz, 1H), 2.29 – 2.14 (m, 2H), 1.99 – 1.87 (m, 2H), 1.88 – 1.74 (m, 2H), 1.67 – 1.50 (m, 2H), 1.46 – 1.28 (m, 4H) ppm.

^{13}C{^{1}H} NMR (101 MHz, $CDCl_3$) δ = 134.8 (d, *J* = 3.0 Hz), 133.9 (d, *J* = 9.2 Hz), 130.6 (d, *J* = 12.0 Hz), 118.1 (d, *J* = 82.7 Hz), 29.9 (d, *J* = 42.9 Hz), 28.4 (d, *J* = 1.8 Hz), 27.6, 27.2 (d, *J* = 15.7 Hz) ppm.

^{31}P{^{1}H} NMR (162 MHz, $CDCl_3$) δ = 30.9 ppm.

HRMS (ESI+): m/z for $C_{25}H_{28}P$ [M-Br]+ calcd.: 359.1923, found: 359.1922.

Triphenyl(tetrahydro-2H-pyran-4-yl)phosphonium bromide (10z)

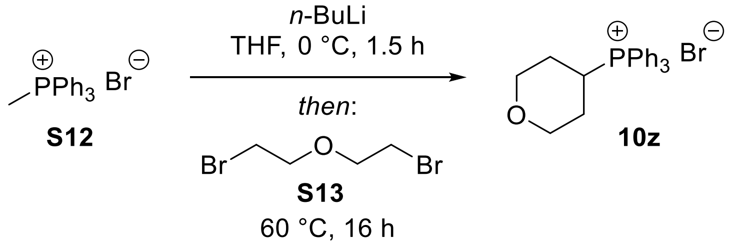

A 250-mL round-bottom flask under nitrogen was charged with methyltriphenylphosphonium bromide (**S12**) (3.21 g, 9.0 mmol, 1.8 equiv.) and THF (10 mL). The flask was cooled to 0 °C, and *n*-butyllithium (1.6 M in hexanes, 5.9 mL, 9.4 mmol, 1.9 equiv.) was slowly added. The mixture was stirred at 0 °C for 1.5 hours. In a separate flask under nitrogen, ether **S13** (630 µL, 5.0 mmol, 1.0 equiv.) was dissolved in THF (8 mL). The solution of **S13** was added to the reaction mixture at 0 °C. The reaction mixture was heated to 60 °C and stirred at that temperature for 16 h. After cooling to room temperature, the off-white suspension was filtered, and the residue was washed with ethyl acetate. The residue was recrystallized from hot water, yielding the title compound as an off-white solid (1.34 g, 63%).

^{1}H NMR (400 MHz, $CDCl_3$) δ = 8.00 (dd, *J* = 12.0, 7.2 Hz, 6H), 7.76 – 7.67 (m, 3H), 7.69 – 7.60 (m, 6H), 6.07 (q, *J* = 11.9 Hz, 1H), 4.26 (t, *J* = 11.6 Hz, 2H), 3.82 (dt, *J* = 10.9, 5.0 Hz, 2H), 1.94 (d, *J* = 12.7 Hz, 2H), 1.47 – 1.32 (m, 2H) ppm.

^{13}C{^{1}H} NMR (101 MHz, $CDCl_3$) δ = 134.8 (d, *J* = 3.0 Hz), 134.1 (d, *J* = 9.4 Hz), 130.6 (d, *J* = 12.3 Hz), 116.9 (d, *J* = 83.6 Hz), 66.2 (d, *J* = 14.0 Hz), 26.6 (d, *J* = 48.5 Hz), 25.7 (d, *J* = 4.0 Hz) ppm.

^{31}**P{^{1}H} NMR** (162 MHz, $CDCl_3$) δ = 26.7 ppm.

HRMS (ESI+): m/z for $C_{23}H_{24}OP$ [M-Br]$^+$ calcd.: 347.1559, found: 347.1555.

Triphenyl(5-phenylpentan-2-yl)phosphonium iodide (10ab)

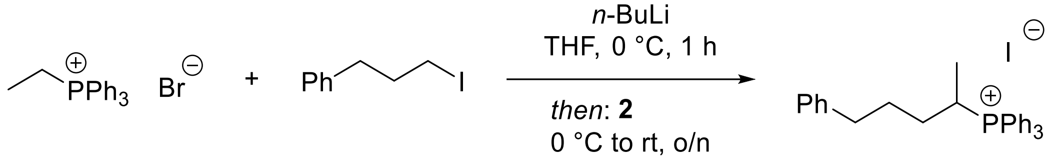

S14 **2** **10ab**

Under an atmosphere of nitrogen, a 50-mL round-bottom flask was charged with ethyltriphenylphosphonium bromide (**S14**) (1.11 g, 3.0 mmol, 1.0 equiv.) and THF (12 mL). The mixture was cooled to 0 °C. Then, *n*-butyllithium (1.6 M in hexanes, 2.1 mL, 3.3 mmol, 1.1 equiv.) was added dropwise, and the mixture was stirred at 0 °C for one hour. Subsequently, a solution of 1-iodo-3-phenylpropane (**2**) (886 mg, 3.6 mmol, 1.2 equiv.) in THF (2 mL) was added. The reaction mixture was allowed to warm to room temperature overnight. Then, a saturated aqueous solution of ammonium chloride was added, and the phases were separated. The aqueous layer was extracted with DCM three times. The combined organic layers were washed with brine and then dried with magnesium sulfate. Volatile materials were removed under reduced pressure, and the crude product was purified by column chromatography to yield the title compound as an off-white solid (877 mg, 54%).

^{1}**H NMR** (400 MHz, $CDCl_3$) δ = 7.90 – 7.81 (m, 6H), 7.76 – 7.70 (m, 3H), 7.68 – 7.60 (m, 6H), 7.22 – 7.07 (m, 5H), 4.94 – 4.79 (m, 1H), 2.71 – 2.52 (m, 2H), 2.26 – 2.12 (m, 1H), 1.95 – 1.79 (m, 2H), 1.33 (dd, *J* = 19.6, 6.9 Hz, 3H), 1.18 – 1.04 (m, 1H) ppm.

^{13}**C{^{1}H} NMR** (101 MHz, $CDCl_3$) δ = 141.1, 134.8 (d, *J* = 3.1 Hz), 134.0 (d, *J* = 9.3 Hz), 130.6 (d, *J* = 12.1 Hz), 128.6, 128.4, 125.9, 117.6 (d, *J* = 83.1 Hz), 35.2, 29.5, 27.8 (d, *J* = 14.0 Hz), 25.9 (d, *J* = 45.8 Hz), 13.5 (d, *J* = 2.3 Hz) ppm.

^{31}**P{^{1}H} NMR** (162 MHz, $CDCl_3$) δ = 31.2 ppm.

HRMS (ESI+): m/z for $C_{29}H_{30}P$ [M-I]$^+$ calcd.: 409.2080, found: 409.2073.

(5-(4-Bromophenyl)pentan-2-yl)triphenylphosphonium iodide (10ac)

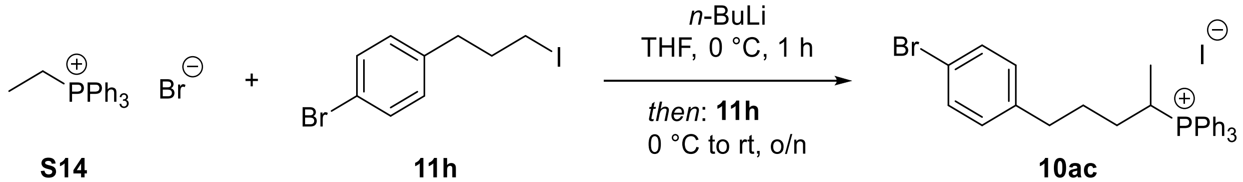

S14 **11h** **10ac**

Under an atmosphere of nitrogen, a 50-mL round-bottom flask was charged with ethyltriphenylphosphonium bromide (**S14**) (1.11 g, 4.0 mmol, 1.0 equiv.) and THF (12 mL). The mixture was cooled to 0 °C. Then, *n*-butyllithium (1.6 M in hexanes, 2.1 mL, 3.3 mmol, 1.1 equiv.) was added dropwise, and the mixture was stirred at 0 °C for one hour. Subsequently, a solution of 1-bromo-4-(3-iodopropyl)benzene (**11h**) (1.17 g, 3.6 mmol, 1.2 equiv.) in THF (3 mL) was added. The reaction mixture

was allowed to warm to room temperature overnight. Then, the reaction mixture was filtered. The residue was washed with ethyl acetate and then dissolved in a mixture of DCM and water. The layers were separated, and the aqueous layer was extracted three times with DCM. The combined organic layers were washed with brine and then dried with magnesium sulfate. Volatile materials were removed under reduced pressure to yield the title compound as a white solid (1.44 g, 78%).

^{1}H NMR (400 MHz, $CDCl_3$) δ = 7.97 – 7.87 (m, 6H), 7.80 – 7.72 (m, 3H), 7.69 – 7.61 (m, 6H), 7.31 (d, *J* = 8.3 Hz, 2H), 7.07 (d, *J* = 8.4 Hz, 2H), 5.32 – 4.94 (m, 1H), 2.71 – 2.49 (m, 2H), 2.34 – 2.15 (m, 1H), 1.99 – 1.79 (m, 2H), 1.34 (dd, *J* = 19.6, 6.8 Hz, 3H), 1.14 – 0.88 (m, 1H) ppm.

^{13}C{^{1}H} NMR (101 MHz, $CDCl_3$) δ = 140.3, 134.8 (d, *J* = 2.2 Hz), 134.1 (d, *J* = 9.3 Hz), 131.4, 130.7, 130.6, 130.5, 119.7, 117.7 (d, *J* = 82.5 Hz), 34.6, 29.3, 27.6 (d, *J* = 14.5 Hz), 25.8 (d, *J* = 45.6 Hz), 13.6 ppm (more signals than expected as signals are reported as singlets when multiplet assignment was ambiguous).

^{31}P{^{1}H} NMR (162 MHz, $CDCl_3$) δ = 31.5 ppm.

HRMS (ESI+): m/z for $C_{29}H_{29}BrP$ [M-I]+ calcd.: 487.1185, found: 487.1177.

(3-Bromopropoxy)(*tert*-butyl)diphenylsilane (S16)

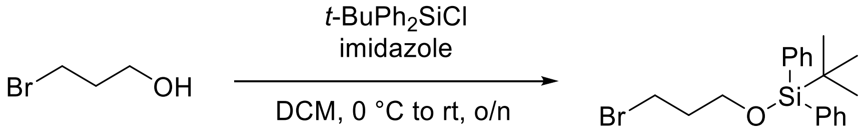

S15 **S16**

To a 100-mL round-bottom flask were added 3-bromopropan-1-ol (**S15**) (900 µL, 10.0 mmol, 1.0 equiv.) and DCM (50 mL). The mixture was cooled to 0 °C. Subsequently, *tert*-butylchlorodiphenylsilane (2.8 mL, 11.0 mmol, 1.1 equiv.) was added. Then, imidazole (1.0 g, 15 mmol, 1.5 equiv.) was added portionwise. The reaction mixture was allowed to warm to room temperature overnight (ca. 14 h). Then, a saturated aqueous solution of sodium hydrogen carbonate was added, phases were separated, and the organic layer was extracted with DCM. The combined organic layers were washed with water and brine before they were dried with magnesium sulfate. Volatile materials were removed under reduced pressure, and the residue was purified by column chromatography (cyclohexane) to yield the product as a colorless oil (2.26 g, 60%).

^{1}H NMR (400 MHz, $CDCl_3$) δ = 7.70 – 7.65 (m, 4H), 7.47 – 7.37 (m, 6H), 3.79 (t, *J* = 5.7 Hz, 2H), 3.59 (t, *J* = 6.5 Hz, 2H), 2.18 – 1.92 (m, 2H), 1.06 (s, 9H) ppm.

^{13}C{^{1}H} NMR (101 MHz, $CDCl_3$) δ = 135.7, 133.7, 129.8, 127.8, 61.5, 35.6, 30.7, 27.0, 19.4 ppm.

The spectroscopic data matched those reported in the literature.[8]

(5-((*tert*-Butyldiphenylsilyl)oxy)pentan-2-yl)triphenylphosphonium bromide (10ad)

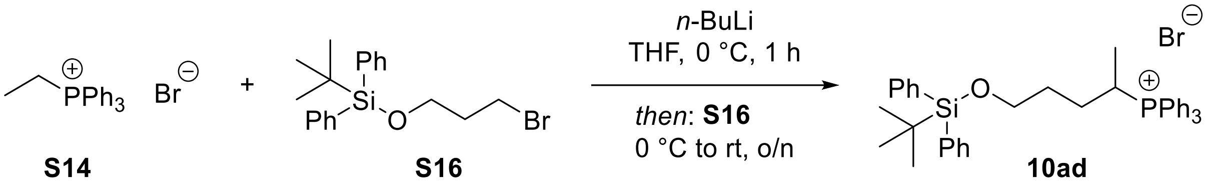

Under an atmosphere of nitrogen, a 50-mL round-bottom flask was charged with ethyltriphenylphosphonium bromide (**S14**) (1.11 g, 4.0 mmol, 1.0 equiv.) and THF (12 mL). The mixture was cooled to 0 °C. Then, *n*-butyllithium (1.6 M in hexanes, 2.1 mL, 3.3 mmol, 1.1 equiv.) was added dropwise, and the mixture was stirred at 0 °C for one hour. Subsequently, a solution of (3-bromopropoxy)(*tert*-butyl)diphenylsilane (**S16**) (1.36 g, 3.6 mmol, 1.2 equiv.) in THF (3 mL) was added. The reaction mixture was allowed to warm to room temperature overnight. Then, the reaction mixture was dissolved by addition of DCM and loaded on silica. Purification by column chromatography yielded the title compound as a white solid (506 mg, 25%).

^{1}H NMR (400 MHz, $CDCl_3$) δ = 8.02 – 7.92 (m, 6H), 7.78 – 7.71 (m, 3H), 7.69 – 7.63 (m, 6H), 7.53 – 7.46 (m, 4H), 7.41 – 7.34 (m, 2H), 7.32 – 7.25 (m, 4H), 5.50 – 5.37 (m, 1H), 3.73 – 3.59 (m, 2H), 2.25 – 2.13 (m, 1H), 2.10 – 1.98 (m, 1H), 1.88 – 1.76 (m, 1H), 1.34 (dd, *J* = 19.8, 6.8 Hz, 3H), 1.26 – 1.12 (m, 1H), 0.89 (s, 9H) ppm.

^{13}C{^{1}H} NMR (101 MHz, $CDCl_3$) δ = 135.4 (d, *J* = 2.2 Hz), 134.6 (d, *J* = 3.0 Hz), 134.0 (d, *J* = 9.3 Hz), 133.6 (d, *J* = 14.3 Hz), 130.4 (d, *J* = 12.1 Hz), 129.6, 127.6 (d, *J* = 2.9 Hz), 117.9 (d, *J* = 82.9 Hz), 63.3, 29.4 (d, *J* = 14.6 Hz), 27.4, 26.8, 25.2 (d, *J* = 45.1 Hz), 19.2, 13.5 ppm.

^{31}P{^{1}H} NMR (162 MHz, $CDCl_3$) δ = 31.6 ppm.

HRMS (ESI+): m/z for $C_{39}H_{44}OPSi$ $[M-Br]^+$ calcd.: 587.2894, found: 587.2886.

(3-Iodoropropyl)(phenyl)sulfane (S18)

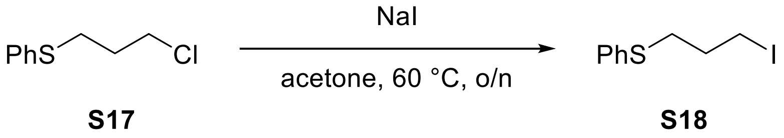

Under an atmosphere of nitrogen, a 100-mL round-bottom flask was charged with (3-chloropropyl)(phenyl)sulfane (**S17**) (1.87 g, 10.0 mmol, 1.0 equiv.), sodium iodide (6.7 g, 45 mmol, 4.5 equiv.), and acetone (20 mL). The mixture was stirred at 60 °C overnight (ca. 14 h). After cooling to room temperature, volatile materials were removed under reduced pressure, and the residue was dissolved in a mixture of DCM and a saturated aqueous solution of sodium thiosulfate. The phases were separated, and the aqueous layer was extracted twice with DCM. The combined organic layers were washed with brine and then dried with magnesium sulfate. Volatile materials were removed under reduced pressure to yield the title compound as a yellow oil (2.79 g, quant.).

^{1}H NMR (400 MHz, $CDCl_3$) δ = 7.40 – 7.32 (m, 2H), 7.34 – 7.25 (m, 2H), 7.25 – 7.16 (m, 1H), 3.30 (t, *J* = 6.7 Hz, 2H), 3.02 (t, *J* = 6.9 Hz, 2H), 2.10 (p, *J* = 6.7 Hz, 2H) ppm.

^{13}C{^{1}H} NMR (101 MHz, $CDCl_3$) δ = 135.7, 129.8, 129.1, 126.5, 34.4, 32.5, 5.0 ppm.

The spectroscopic data matched those reported in the literature.[9]

Triphenyl(5-(phenylthio)pentan-2-yl)phosphonium iodide (10af)

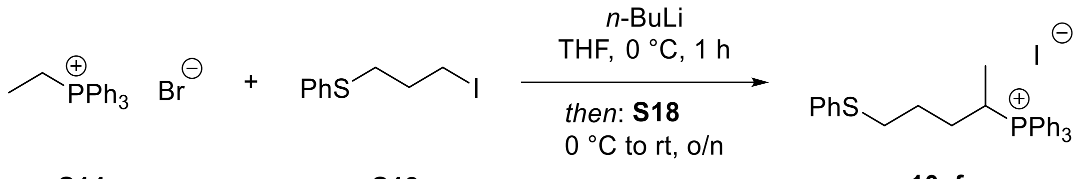

Under an atmosphere of nitrogen, a 50-mL round-bottom flask was charged with ethyltriphenylphosphonium bromide (**S14**) (1.11 g, 4.0 mmol, 1.0 equiv.) and THF (12 mL). The mixture was cooled to 0 °C. Then, *n*-butyllithium (1.6 M in hexanes, 2.1 mL, 3.3 mmol, 1.1 equiv.) was added dropwise, and the mixture was stirred at 0 °C for one hour. Subsequently, a solution of (3-iodopropyl)(phenyl)sulfane (**S18**) (1.00 g, 3.6 mmol, 1.2 equiv.) in THF (3 mL) was added. The reaction mixture was allowed to warm to room temperature overnight. Then, the reaction mixture was filtered, and the residue was washed with ethyl acetate. The residue was dissolved in a mixture of DCM and water. The layers were separated, and the aqueous phase was extracted three times with DCM. The combined organic layers were dried with magnesium sulfate, and volatile materials were removed under reduced pressure to yield the title compound as a white solid (1.34 g, 79%).

^{1}H NMR (400 MHz, $CDCl_3$) δ = 7.95 – 7.85 (m, 6H), 7.79 – 7.72 (m, 3H), 7.71 – 7.63 (m, 6H), 7.18 (d, *J* = 4.3 Hz, 4H), 7.15 – 7.07 (m, 1H), 5.10 – 4.92 (m, 1H), 3.10 – 2.91 (m, 2H), 2.37 – 2.22 (m, 1H), 2.08 – 1.93 (m, 1H), 1.94 – 1.83 (m, 1H), 1.36 – 1.20 (m, 4H (overlapping singles including dd, *J* = 19.6, 6.9 Hz) ppm.

^{13}C{^{1}H} NMR (101 MHz, $CDCl_3$) δ = 136.2, 134.9 (d, *J* = 3.0 Hz), 134.0 (d, *J* = 9.3 Hz), 130.6 (d, *J* = 12.2 Hz), 129.3, 129.0, 126.0, 117.6 (d, *J* = 83.2 Hz), 33.6, 28.9, 26.1 (d, *J* = 14.6 Hz), 25.6 (d, *J* = 46.2 Hz), 13.5 (d, *J* = 2.2 Hz) ppm.

^{31}P{^{1}H} NMR (162 MHz, $CDCl_3$) δ = 31.4 ppm.

HRMS (ESI+): m/z for $C_{29}H_{30}PS$ $[M-I]^+$ calcd.: 441.1800, found: 441.1797.

Phosphonium iodide 10ag

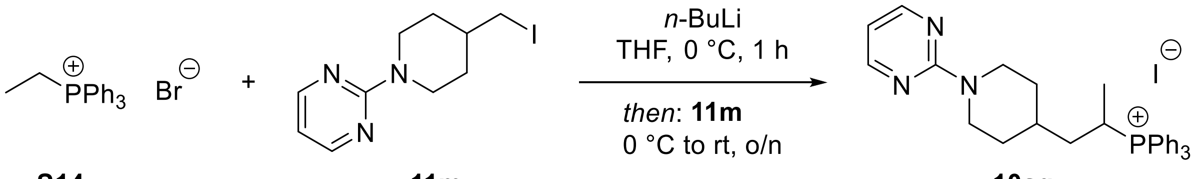

Under an atmosphere of nitrogen, a 50-mL round-bottom flask was charged with ethyltriphenylphosphonium bromide (**S14**) (1.11 g, 4.0 mmol, 1.0 equiv.) and THF (12 mL). The mixture was cooled to 0 °C. Then, *n*-butyllithium (1.6 M in hexanes, 2.1 mL, 3.3 mmol, 1.1 equiv.) was added dropwise, and the mixture was stirred at 0 °C for one hour. Subsequently, a solution of 2-(4-(Iodomethyl)piperidin-1-yl)pyrimidine (**11m**) (1.09 g, 3.6 mmol, 1.2 equiv.) in THF (3 mL) was added. The

reaction mixture was allowed to warm to room temperature overnight. Then, the reaction was quenched with methanol. Volatile materials were removed under reduced pressure, and the residue was purified by column chromatography (0% to 6% methanol in DCM) to yield the title compound as a white solid (1.08 g, 61%, ca. 92% pure (remaining **S14**)).

^{1}H NMR (400 MHz, $CDCl_3$) δ = 8.20 (d, *J* = 4.7 Hz, 2H), 7.99 – 7.86 (m, 6H), 7.80 – 7.71 (m, 3H), 7.73 – 7.63 (m, 6H), 6.37 (t, *J* = 4.7 Hz, 1H), 4.86 – 4.53 (m, 3H), 3.02 – 2.71 (m, 2H), 2.53 (br-d, *J* = 12.8 Hz, 1H), 2.05 – 1.92 (m, 1H), 1.71 – 1.55 (m, 2H), 1.44 (dd, *J* = 19.7, 6.8 Hz, 3H), 1.20 – 0.98 (m, 3H) ppm.

^{13}C{^{1}H} NMR (101 MHz, $CDCl_3$) δ = 161.6, 157.7, 135.0 (d, *J* = 3.0 Hz), 134.0 (d, *J* = 9.3 Hz), 130.7 (d, *J* = 12.0 Hz), 117.4 (d, *J* = 83.1 Hz), 109.3, 43.8 (d, *J* = 1.6 Hz), 37.6 (d, *J* = 1.8 Hz), 33.7 (d, *J* = 13.9 Hz), 32.6 (d, *J* = 120.9 Hz), 24.2 (d, *J* = 46.4 Hz), 14.6 (d, *J* = 2.3 Hz) ppm.

^{31}P{^{1}H} NMR (162 MHz, $CDCl_3$) δ = 31.5 ppm.

HRMS (ESI+): m/z for $C_{30}H_{33}N_3P$ [M-I]$^+$ calcd.: 466.2407, found: 466.2396.

(6-Iodohexyl)benzene (11a)

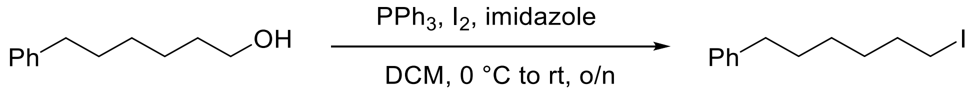

S19 **11a**

To a 250-mL two-neck flask under nitrogen were added triphenylphosphine (**5**) (4.7 g, 17.9 mmol, 3.2 equiv.) and imidazole (1.3 g, 19.6 mmol, 3.5 equiv.). DCM (50 mL) was added, and the mixture was cooled to 0 °C. Then, iodine (4.6 g, 17.9 mmol, 3.2 equiv.) was added, and the mixture was stirred at 0 °C for 15 minutes. 6-Phenylhexan-1-ol (**S19**) (1.0 g, 5.6 mmol, 1.0 equiv.) was added at 0 °C. The reaction mixture was allowed to warm to room temperature overnight. Then, a saturated aqueous solution of sodium thiosulfate was added, and the phases were separated. The aqueous layer was extracted with DCM, and the combined organic layers were dried with magnesium sulfate. Volatile materials were evaporated under reduced pressure, and the crude product was purified by column chromatography (cyclohexane) to yield the title compound as a colorless oil (0.98 g, 61%).

^{1}H NMR (400 MHz, $CDCl_3$) δ = 7.31 – 7.25 (m, 2H), 7.21 – 7.16 (m, 3H), 3.19 (t, *J* = 7.0 Hz, 2H), 2.62 (t, *J* = 7.7 Hz, 2H), 1.83 (p, *J* = 7.0 Hz, 2H), 1.64 (p, *J* = 7.5 Hz, 2H), 1.49 – 1.32 (m, 4H) ppm.

^{13}C{^{1}H} NMR (101 MHz, $CDCl_3$) δ = 142.7, 128.5, 128.4, 125.8, 36.0, 33.6, 31.4, 30.5, 28.3, 7.3 ppm.

The spectroscopic data matched those reported in the literature.[10]

(4-Iodobutoxy)benzene (11b)

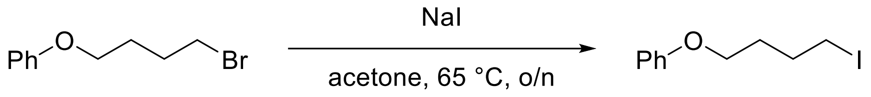

S20 **11b**

To a 50-mL round-bottom flask under nitrogen were added (4-bromobutoxy)benzene (**S20**) (1.83 g, 8.0 mmol, 1.0 equiv.), sodium iodide (3.6 g, 24 mmol, 3.0 equiv.), and dry acetone (16 mL). The reaction mixture was stirred in the dark at 65 °C overnight. After cooling to room temperature, volatile materials were evaporated under reduced pressure. Then, a saturated aqueous solution of sodium thiosulfate and DCM were added, and the phases were separated. The aqueous layer was extracted with DCM three times. The combined organic layers were washed with brine and dried with magnesium sulfate. Volatile materials were evaporated under reduced pressure, and the crude product was purified by column chromatography (*n*-hexane) to yield the title compound as a colorless oil that solidified upon standing (1.98 g, 90%).

^{1}H NMR (400 MHz, $CDCl_3$) δ = 7.33 – 7.25 (m, 2H), 6.95 (tt, *J* = 7.3, 1.1 Hz, 1H), 6.92 – 6.86 (m, 2H), 3.99 (t, *J* = 6.0 Hz, 2H), 3.27 (t, *J* = 6.9 Hz, 2H), 2.13 – 1.99 (m, 2H), 1.95 – 1.85 (m, 2H) ppm.

^{13}C{^{1}H} NMR (101 MHz, $CDCl_3$) δ = 159.0, 129.6, 120.9, 114.6, 66.6, 30.4, 30.3, 6.6 ppm.

The spectroscopic data matched those reported in the literature.[11]

***tert*-Butyl(3-iodopropoxy)diphenylsilane (11c)**

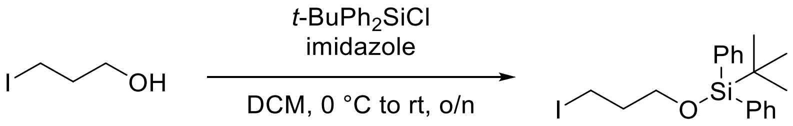

S21 **11c**

To a 250-mL round-bottom flask were added 3-iodopropan-1-ol (**S21**) (480 µL, 5.0 mmol, 1.0 equiv.) and DCM (50 mL). The mixture was cooled to 0 °C. Subsequently, *tert*-butylchlorodiphenylsilane (1.4 mL, 5.5 mmol, 1.1 equiv.) and imidazole (510 mg, 7.5 mmol, 1.5 equiv.) were added. The cooling bath was removed, and the reaction mixture was stirred for room temperature overnight (ca. 14 h). Then, a saturated aqueous solution of sodium hydrogencarbonate was added, phases were separated, and the organic layer was extracted with DCM. The combined organic layers were washed with water and brine before they were dried with magnesium sulfate. Volatile materials were removed under reduced pressure, and the residue was purified by column chromatography (cyclohexane) to yield the product as a colorless oil (558 mg, 26%).

^{1}H NMR (400 MHz, $CDCl_3$) δ = 7.70 – 7.65 (m, 4H), 7.47 – 7.37 (m, 6H), 3.72 (t, *J* = 5.7 Hz, 2H), 3.35 (t, *J* = 6.8 Hz, 2H), 2.04 (tt, *J* = 6.8, 5.7 Hz, 2H), 1.06 (s, 9H) ppm.

^{13}C{^{1}H} NMR (101 MHz, $CDCl_3$) δ = 135.7, 133.7, 129.8, 127.9, 63.4, 36.3, 27.0, 19.4, 3.6 ppm.

The spectroscopic data matched those reported in the literature.[12]

5-(3-iodopropyl)benzo[d][1,3]dioxole (11d)

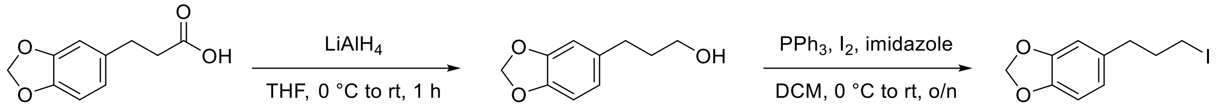

S22 **S23** **11d**

A 50-mL two-neck round-bottom flask placed under an atmosphere of nitrogen was charged with a solution of 3-(benzo[d][1,3]dioxol-5-yl)propanoic acid (**S22**) (500 mg, 2.6 mmol, 1.0 equiv.) in anhydrous THF (2 mL). A solution of $LiAlH_4$ (150 mg, 3.9 mmol, 1.5 equiv.) in anhydrous THF (2 mL) was added dropwise at 0 °C. The reaction mixture was allowed to warm to 25 °C and stirred for 4 h. The reaction mixture was then cooled to 0 °C and diluted with Et_2O (10 mL), and treated sequentially with water (0.2 mL), NaOH (2 M, 0.4 mL), water (0.6 mL), warmed to 25 °C, and stirred for 15 min. $MgSO_4$ was added and the mixture was stirred for further 15 min. The slurry was then filtered, and the filtrate concentrated under reduced pressure to afford the crude alcohol **S23** as a colorless oil (366 mg, 79%). The crude alcohol **S23** was used in the next step without further purification.

To a 50-mL two-neck round-bottom flask under nitrogen were added triphenylphosphine (**5**) (639 mg, 2.4 mmol, 1.2 equiv.) and imidazole (207 mg, 3 mmol, 1.5 equiv.). DCM (10 mL) was added, and the mixture was cooled to 0 °C. Then, iodine (619 mg, 2.4 mmol, 1.2 equiv.) was added, and the mixture was stirred at 0 °C for 15 minutes. 3-(benzo[d][1,3]dioxol-5-yl)propan-1-ol (**S23**) (366 mg, 2 mmol, 1.0 equiv.) was added at 0 °C. The reaction mixture was allowed to warm to room temperature overnight. Then, a saturated aqueous solution of sodium thiosulfate was added, and the phases were separated. The aqueous layer was extracted with DCM, and the combined organic layers were dried with magnesium sulfate. Volatile materials were evaporated under reduced pressure, and the crude product was purified by column chromatography (cyclohexane) to yield the title compound as a yellow oil (467 mg, 79%).

^{1}H NMR (400 MHz, $CDCl_3$) δ = 6.73 (d, *J* = 7.8 Hz, 1H), 6.69 – 6.63 (m, 2H), 5.93 (s, 2H), 3.16 (t, *J* = 6.8 Hz, 2H), 2.65 (t, *J* = 7.2 Hz, 2H), 2.08 (p, *J* = 7.2 Hz, 2H) ppm.

^{13}C{^{1}H} NMR (101 MHz, $CDCl_3$) δ = 147.8, 146.0, 134.3, 121.5, 109.1, 108.4, 101.0, 36.0, 35.2, 6.4 ppm.

The spectroscopic data matched those reported in the literature.[13]

(3-(Hydroxymethyl)piperidin-1-yl)(phenyl)methanone (S25)

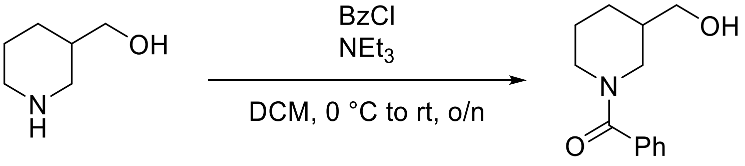

S24 **S25**

A 100-mL round-bottom flask was charged with piperidin-3-ylmethanol (**S24**) (633 mg, 5.5 mmol, 1.1 equiv.), triethylamine (1,4 mL, 10.0 mmol, 2.0 equiv.), and DCM (20 mL). The mixture was cooled to 0 °C. Then, a solution of benzoyl chloride (580 µL, 5.0 mmol, 1.0 equiv.) in DCM (10 mL) was added dropwise, and the mixture was allowed to warm to room temperature overnight. Then, aqueous hydrochloric acid (1 M) was added, and the phases were separated. The aqueous layer was extracted with DCM three

times, and the combined organic layers were dried with magnesium sulfate. Volatile materials were removed under reduced pressure, and the crude product was purified by column chromatography (ethyl acetate) to yield the title compound as a white solid (985 mg, 82%).

The product forms a mixture of rotamers at ambient temperature. Signals were attributed to the major rotamer (major) and the minor rotamer (minor) when possible.

^{1}H NMR (400 MHz, $CDCl_3$) δ = 7.35 (s, 5H), 4.43 (br, 0.4H, minor), 4.13 (br, 0.6H, major), 3.74 (br, 0.4H, minor), 3.48 (br, 2H), 3.90 – 3.05 (m, 3H), 2.79 (br, 1H), 1.92 – 1.20 (m, 5H) ppm.

^{13}C{^{1}H} NMR (101 MHz, $CDCl_3$) δ = 171.1 (major), 170.7 (minor), 136.0, 129.7, 128.5, 127.0, 64.5 (minor), 64.0 (major), 51.0 (minor), 48.9 (major), 45.2 (major), 43.1 (minor), 39.5 (minor), 38.0 (major), 27.3 (minor), 26.8 (major), 24.7 (major), 24.4 (minor) ppm.

HRMS (ESI+): m/z for $C_{13}H_{18}NO_2$ $[M+H]^+$ calcd.: 220.1332, found: 220.1330.

(3-(Iodomethyl)piperidin-1-yl)(phenyl)methanone (11e)

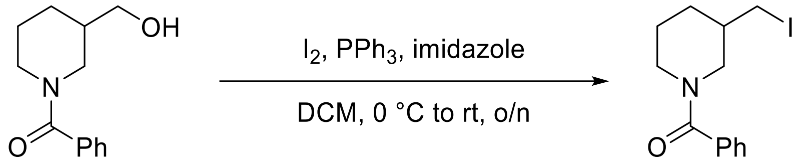

S25 **11e**

Under an atmosphere of nitrogen, a 50-mL round-bottom flask was charged with triphenylphosphine (944 mg, 3.6 mmol, 1.2 equiv.), imidazole (265 mg, 3.9 mmol, 1.3 equiv.), and DCM (9 mL). After cooling the mixture to 0 °C, iodine (914 mg, 3.6 mmol, 1.2 equiv.) was added. The mixture was stirred 15 min at 0 °C. Then, a solution of alcohol **S25** (658 mg, 3.0 mmol, 1.0 equiv.) in DCM (3 mL) was added, and the reaction mixture was allowed to warm to room temperature overnight. Subsequently, an aqueous saturated solution of sodium thiosulfate was added, and the phases were separated. The aqueous layer was extracted with DCM three times, and the combined organic layers were dried with magnesium sulfate. Volatile materials were removed under reduced pressure, and the crude product was purified by column chromatography (20% to 34% ethyl acetate in cyclohexane) to yield the title compound as an off-white solid (842 mg, 85%).

^{1}H NMR (400 MHz, $CDCl_3$) δ = 7.43 – 7.35 (m, 5H), 4.57 (br-*pseudo*-d, *J* = 55.9 Hz, 1H), 3.75 (br-*pseudo*-d, *J* = 90.2 Hz, 1H), 3.30 – 2.53 (m, 4H), 2.11 – 1.89 (m, 1H), 1.60 (*pseudo*-d, *J* = 76.1 Hz, 3H), 1.41 – 1.26 (m, 1H) ppm.

^{13}C{^{1}H} NMR (101 MHz, $CDCl_3$) δ = 170.6, 136.1 (br), 129.7, 128.6, 127.0, 53.7 (br), 48.1 (br), 42.8 (br), 39.2 (br), 37.6 (br), 31.6 (br), 25.5 (br), 24.4 (br), 10.0 (br), 8.9 (br) ppm (additional signals due to rotamers).

HRMS (ESI+): m/z for C_{13}H17NOI [M+H]+ calcd.: 330.0349, found: 330.0342.

3-(4-Iodophenyl)propan-1-ol (S27)

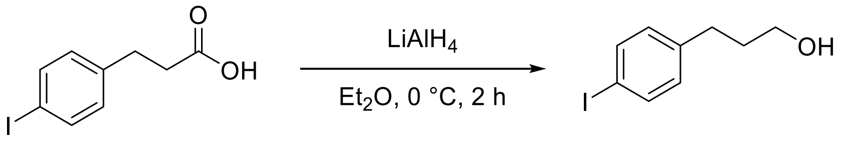

S26 **S27**

In a 50-mL round-bottom flask to a solution of carboxylic acid **S26** (966 mg, 3.5 mmol, 1.0 equiv.) in Et_2O (11 mL) cooled to 0 °C, $LiAlH_4$ (136 mg, 3.57 mmol, 1.05 equiv.) was added portionwise. The resulting suspension was stirred at 0 °C for 2 hours. After Fieser work-up, all solids were removed by filtration and the filtrate was evaporated under reduced pressure. The obtained crude alcohol was used for the subsequent reaction without prior purification or characterization.

1-Iodo-4-(3-iodopropyl)benzene (11g)

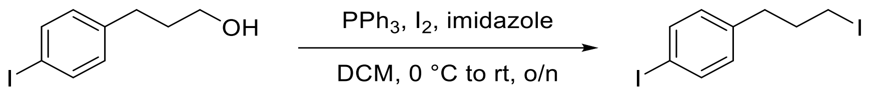

S27 **11g**

To a 50-mL two-neck flask under nitrogen were added triphenylphosphine (**5**) (1.10 g, 4.20 mmol, 1.2 equiv.) and imidazole (310 mg, 4.55 mmol, 1.3 equiv.). DCM (14 mL) was added, and the mixture was cooled to 0 °C. Then, iodine (1.07 g, 4.20 mmol, 1.2 equiv.) was added, and the mixture was stirred at 0 °C for 15 minutes. 3-(4-Iodophenyl)propan-1-ol (**S27**) (917 mg, 3.50 mmol, 1.0 equiv.) was added at 0 °C. The reaction mixture was allowed to warm to room temperature overnight. Then, a saturated aqueous solution of sodium thiosulfate was added, and the phases were separated. The aqueous layer was extracted with DCM, and the combined organic layers were dried with sodium sulfate. Volatile materials were evaporated under reduced pressure, and the crude product was purified by column chromatography (pentane) to yield the title compound as a white solid (270 mg, 0.73 mmol, 21%)

^{1}H NMR (400 MHz, $CDCl_3$) δ = 7.65 – 7.57 (m, 2H), 7.01 – 6.90 (m, 2H), 3.15 (t, *J* = 6.8 Hz, 2H), 2.68 (t, *J* = 7.3 Hz, 2H), 2.09 (dq, *J* = 7.9, 6.8 Hz, 2H) ppm.

^{13}C{^{1}H} NMR (101 MHz, $CDCl_3$) δ = 140.1, 137.7, 130.8, 91.4, 35.8, 34.6, 6.1 ppm.

The spectroscopic data matched those reported in the literature.[12]

1-Bromo-4-(3-iodopropyl)benzene (11h)

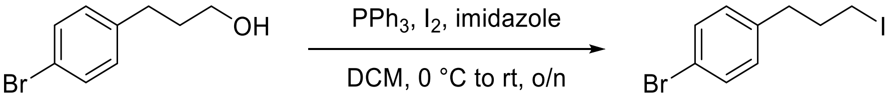

S28 **11h**

To a 100-mL two-neck flask under nitrogen were added triphenylphosphine (**5**) (3.15 g, 12.0 mmol, 1.2 equiv.) and imidazole (885 mg, 13.0 mmol, 1.3 equiv.). DCM (35 mL) was added, and the mixture was cooled to 0 °C. Then, iodine (3.05 g, 12.0 mmol, 1.2 equiv.) was added, and the mixture was stirred at 0 °C for 15 minutes. 3-(4-Bromophenyl)propan-1-ol (**S28**) (2.15 g, 10.0 mmol, 1.0 equiv.) was added at 0 °C. The reaction mixture was allowed to warm to room temperature overnight. Then, a saturated aqueous

solution of sodium thiosulfate was added, and the phases were separated. The aqueous layer was extracted with DCM, and the combined organic layers were dried with sodium sulfate. Volatile materials were evaporated under reduced pressure, and the crude product was purified by column chromatography (pentane) to yield the title compound as a colorless oil (3.16 g, 9.7 mmol, 97%).

^{1}H NMR (400 MHz, $CDCl_3$) δ = 7.47 – 7.36 (m, 2H), 7.12 – 7.02 (m, 2H), 3.15 (t, *J* = 6.8 Hz, 2H), 2.69 (t, *J* = 7.3 Hz, 2H), 2.10 (dq, *J* = 8.1, 6.8 Hz, 2H) ppm.

^{13}C{^{1}H} NMR (101 MHz, $CDCl_3$) δ = 139.5, 131.7, 130.5, 120.1, 35.7, 34.7, 6.1 ppm.

The spectroscopic data matched those reported in the literature.[14]

1-Chloro-4-(3-iodopropyl)benzene (11i)

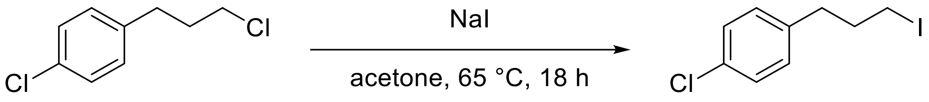

S29 **11i**

To a 50-mL round-bottom flask under nitrogen were added alkyl chloride **S29** (756 mg, 4.0 mmol, 1.0 equiv.) and sodium iodide (2.7 g, 18.0 mmol, 4.5 equiv.). Acetone (8 mL) was added, and the mixture was stirred at 65 °C for 18 hours. Then, volatile materials were removed under reduced pressure. An aqueous saturated solution of NaS_2O_3 and DCM were added. The phases were separated, and the aqueous layer was extracted with DCM three times. The combined organic layers were dried with magnesium sulfate. Volatile materials were evaporated under reduced pressure, and the crude product was purified by column chromatography (cyclohexane) to yield the title compound as a yellow oil (1.08 g, 96%).

^{1}H NMR (400 MHz, $CDCl_3$) δ = 7.30 – 7.22 (m, 2H), 7.13 (d, *J* = 8.6 Hz, 2H), 3.15 (t, *J* = 6.8 Hz, 2H), 2.71 (s, *J* = 7.3 Hz, 2H), 2.16 – 2.05 (m, 2H) ppm.

^{13}C{^{1}H} NMR (101 MHz, $CDCl_3$) δ = 138.9, 132.1, 130.0, 128.8, 35.6, 34.8, 6.1 ppm.

The spectroscopic data matched those reported in the literature.[15]

(3-(4-Bromophenyl)propoxy)(*tert*-butyl)dimethylsilane (S30)

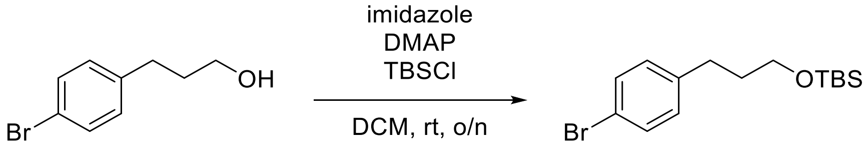

S28 **S30**

To a 250-mL round-bottom flask were added alcohol **S28** (4.30 g, 20.0 mmol, 1.0 equiv.), imidazole (1.91 g, 28.0 mmol, 1.4 equiv.), DMAP (342 mg, 28.0 mmol, 1.4 equiv.), and DCM (80 mL). The solution was stirred at room temperature and TBSCl (3.62 g, 24.0 mmol, 1.2 equiv.) dissolved in DCM (20 mL) was added dropwise. The mixture was stirred at room temperature overnight. Then, water (100 mL) was added and the aqueous phase was extracted with DCM three times. The combined organic layers were dried with sodium sulfate. Volatile materials were evaporated under reduced pressure, and the crude product was

purified by column chromatography (2% ethyl acetate in pentane) to yield the title compound as a colorless oil (6.57 g, 19.9 mmol, quant.).

^{1}H NMR (400 MHz, $CDCl_3$) δ = 7.43 – 7.35 (m, 2H), 7.10 – 7.02 (m, 2H), 3.61 (t, J = 6.2 Hz, 2H), 2.67 – 2.59 (m, 2H), 1.88 – 1.71 (m, 2H), 0.91 (s, 9H), 0.05 (s, 6H) ppm.

^{13}C{^{1}H} NMR (101 MHz, $CDCl_3$) δ = 141.4, 131.5, 130.4, 119.5, 62.2, 34.4, 31.7, 26.1, 18.5, -5.1 ppm.

The spectroscopic data matched those reported in the literature.[16]

Silylether S31

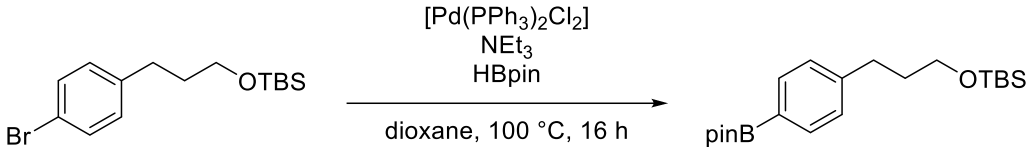

S30 **S31**

Inside a glovebox filled with argon, to a 50 mL Schlenk flask were added [Pd(PPh_3)$_2$$Cl_2$] (393 mg, 0.56 mmol, 0.07 equiv.), dioxane (13 mL), aryl bromide **S30** (2.64 g, 8.00 mmol, 1.0 equiv.), and HBpin (1.74 mL, 1.54 g, 12.0 mmol, 1.5 equiv.). The flask was tightly closed with a septa cap, removed from the glovebox, and NEt_3 (3.35 mL, 2.43 g, 24.0 mmol, 3.0 equiv.) was added. The reaction was heated to 100 °C in an oil bath for 16 hours. After cooling the reaction to room temperature, it was filtered through a Celite plug with ethyl acetate. The organic phase was washed with sat. aq. $NaHCO_3$ solution and dried over sodium sulfate. Volatile materials were evaporated under reduced pressure, and the obtained crude boronic ester was used for the subsequent deprotection without prior purification or characterization.

Alcohol S32

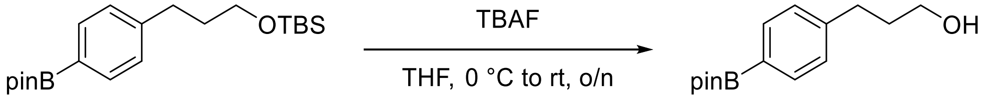

S31 **S32**

To a 100-mL round-bottom flask were added crude boronic ester **S31** (1.45 g, 3.84 mmol, 1.0 equiv.) and THF (38 mL). The solution was cooled to 0 °C, and TBAF (1 M in THF, 5.76 mL, 5.76 mmol, 1.5 equiv.) was added dropwise. The reaction was stirred overnight while allowing slow warming to room temperature. To the reaction mixture was added sat. aq. NH_4Cl solution and the aqueous phase was extracted with ethyl acetate. The combined organic phases were dried over sodium sulfate. Volatile materials were evaporated under reduced pressure, and the crude product was purified by column chromatography (20% ethyl acetate in pentane) to yield the title compound as a colorless oil (314 mg, 1.20 mmol, 31%).

^{1}H NMR (400 MHz, $CDCl_3$) δ = 7.77 – 7.69 (m, 2H), 7.25 – 7.17 (m, 2H), 3.67 (t, J = 6.4 Hz, 2H), 2.77 – 2.67 (m, 2H), 1.95 – 1.85 (m, 2H), 1.34 (s, 12H), 0.91 (s, 1H) ppm.

^{13}C{^{1}H} NMR (101 MHz, $CDCl_3$) δ = 145.4, 135.1, 128.1, 83.8, 62.4, 34.2, 32.4, 25.0 ppm. The carbon atom bound to boron is not observed due to quadrupolar relaxation.

The spectroscopic data matched those reported in the literature.[17]

2-(4-(3-iodopropyl)phenyl)-4,4,5,5-tetramethyl-1,3,2-dioxaborolane (11j)

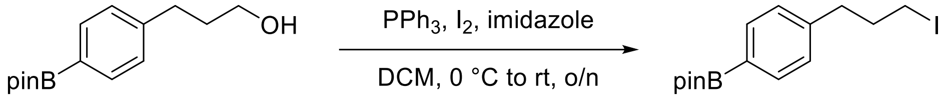

S32 **11j**

To a 25-mL two-neck flask under nitrogen were added triphenylphosphine (**5**) (378 mg, 1.44 mmol, 1.2 equiv.) and imidazole (106 mg, 1.56 mmol, 1.3 equiv.). DCM (5.0 mL) was added, and the mixture was cooled to 0 °C. Then, iodine (366 mg, 1.44 mmol, 1.2 equiv.) was added, and the mixture was stirred at 0 °C for 15 minutes. Alcohol **S32** (314 mg, 1.20 mmol, 1.0 equiv.) was added at 0 °C. The reaction mixture was allowed to warm to room temperature overnight. Then, a saturated aqueous solution of sodium thiosulfate was added, and the phases were separated. The aqueous layer was extracted with DCM, and the combined organic layers were dried with sodium sulfate. Volatile materials were evaporated under reduced pressure, and the crude product was purified by column chromatography (3% ethyl acetate in pentane) to yield the title compound as a colorless oil (285 mg, 0.77 mmol, 64%).

^{1}H NMR (400 MHz, $CDCl_3$) δ = 7.83 – 7.66 (m, 2H), 7.25 – 7.18 (m, 2H), 3.16 (t, *J* = 6.8 Hz, 2H), 2.74 (t, *J* = 7.3 Hz, 2H), 2.21 – 2.06 (m, 2H), 1.34 (s, 12H) ppm.

^{11}B NMR (128 MHz, $CDCl_3$) δ = 30.9 (br) ppm.

^{13}C{^{1}H} NMR (101 MHz, $CDCl_3$) δ = 143.9, 135.2, 128.2, 83.8, 36.6, 34.8, 25.0, 6.4 ppm. The carbon atom bound to boron is not observed due to quadrupolar relaxation.

HRMS (ESI+): m/z for $C_{15}H_{22}BINaO_2$ $[M+Na]^+$ calcd.: 395.0650, found: 395.0652.

10-(4-Chlorobutyl)-10*H*-phenoxazine (S34)

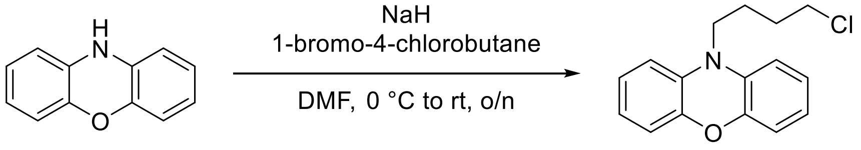

S33 **S34**

To 50-mL round-bottom flask were added phenoxazine (**S33**) (1.19 g, 6.50 mmol, 1.0 equiv.) and DMF (10 mL). The solution was cooled to 0 °C and sodium hydride (60% in mineral oil, 286 mg, 7.15 mmol, 1.1 equiv.) was added. The resulting suspension was stirred at 0 °C for 30 minutes, before 1-bromo-4-chlorobutane (0.83 mL, 1.23 g, 7.15 mmol, 1.1 equiv.) was added dropwise. The reaction mixture was slowly warmed to room temperature and stirred overnight. Water was added and the aqueous phase was extracted three times with diethyl ether. The combined organic layers were dried with sodium sulfate. Volatile materials were evaporated under reduced pressure, and the crude product was purified by column chromatography (4% ethyl acetate in pentane) to yield the title compound as a yellow oil (1.61 g, 5.88 mmol 90%).

^{1}H NMR (400 MHz, $CDCl_3$) δ = 6.80 (ddd, *J* = 8.0, 6.4, 2.6 Hz, 2H), 6.70 – 6.60 (m, 4H), 6.51 – 6.43 (m, 2H), 3.61 (t, *J* = 6.2 Hz, 2H), 3.57 – 3.50 (m, 2H), 1.96 – 1.77 (m, 4H) ppm.

^{13}C{^{1}H} NMR (101 MHz, $CDCl_3$) δ = 145.2, 133.3, 123.8, 121.1, 115.6, 111.4, 44.6, 43.4, 29.9, 22.7 ppm.

The spectroscopic data matched those reported in the literature.[18]

10-(4-Iodobutyl)-10*H*-phenoxazine (11k)

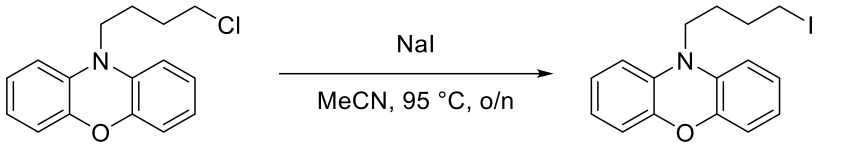

S34 **11k**

Under nitrogen, to a 100-mL two-neck flask equipped with a reflux condenser were added alkyl chloride **S34** (1.61 g, 5.86 mmol, 1.0 equiv.) and sodium iodide (2.64 g, 17.6 mmol, 3.0 equiv.). Acetonitrile (30 mL) was added, and the mixture was stirred at 95 °C overnight. Then, water (30 mL) was added and the aqueous phase was extracted three times with diethyl ether. The combined organic layers were dried with sodium sulfate. Volatile materials were evaporated under reduced pressure, and the crude product was purified by column chromatography (2% ethyl acetate in pentane) to yield the title compound as a yellow solid (1.66 g, 4.54 mmol, 78%, 89% purity (remaining **S34**)).

^{1}H NMR (400 MHz, $CDCl_3$) δ = 6.89 – 6.77 (m, 2H), 6.72 – 6.61 (m, 4H), 6.52 – 6.46 (m, 2H), 3.56 – 3.44 (m, 2H), 3.25 (t, *J* = 6.8 Hz, 2H), 2.02 – 1.88 (m, 2H), 1.88 – 1.73 (m, 2H) ppm.

^{13}C{^{1}H} NMR (101 MHz, $CDCl_3$) δ = 145.1, 133.3, 123.8, 121.0, 115.6, 111.4, 43.0, 30.8, 26.1, 5.9 ppm.

HRMS (ESI+): m/z for $C_{16}H_{17}NOI$ $[M+H]^+$ calcd.: 366.0349, found: 366.0339.

1-(4-Bromobutyl)-2-phenyl-1H-indole (11l)

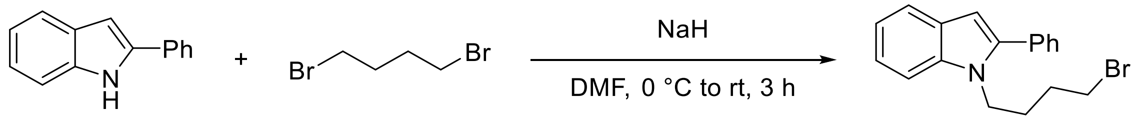

S35 **14** **11l**

To a 50-mL two-neck round-bottom flask were added 2-phenyl-1H-indole (**S35**) (966 mg, 5.0 mmol, 1.0 equiv.) and DMF (20 mL). The mixture was cooled to 0 °C. Then, sodium hydride (240 mg, 60% dispersion in mineral oil, 6.0 mmol, 1.2 equiv.) was added portionwise. The reaction mixture was stirred at 0 °C for 30 minutes. Then, 1,4-dibromobutane (**14**) (1.79 mL, 15.0 mmol, 3.0 equiv.) was added slowly. The reaction mixture was stirred at room temperature for 3 hours. Then, water was added slowly. The aqueous layer was extracted two times with ethyl acetate. The combined organic layers were washed three times with water and two times with aqueous lithium chloride solution (1 M). The organic layers were dried with magnesium sulfate, and volatile materials were removed under reduced pressure. The residue was purified by column chromatography (0% to 3% ethyl acetate in cyclohexane) to yield the product as a light yellow oil (699 mg, 43%).

^{1}H NMR (400 MHz, $CDCl_3$) δ = 7.68 (dt, *J* = 7.7, 1.0 Hz, 1H), 7.54 – 7.43 (m, 5H), 7.42 (dd, *J* = 8.2, 0.9 Hz, 1H), 7.27 (ddd, *J* = 8.2, 7.1, 1.2 Hz, 1H), 7.18 (ddd, *J* = 7.9, 7.0, 1.0 Hz, 1H), 6.57 (d, *J* = 0.8 Hz, 1H), 4.23 (t, *J* = 7.2 Hz, 2H), 3.21 (t, *J* = 6.6 Hz, 2H), 1.86 (tt, *J* = 7.9, 6.8 Hz, 2H), 1.75 – 1.62 (m, 2H) ppm.

^{13}C{^{1}H} NMR (101 MHz, $CDCl_3$) δ = 141.4, 137.4, 133.2, 129.6, 128.7, 128.4, 128.2, 121.8, 120.8, 120.1, 110.1, 102.5, 43.0, 32.9, 29.8, 28.5 ppm.

The spectroscopic data matched those reported in the literature.[19]

2-(4-(Iodomethyl)piperidin-1-yl)pyrimidine (11m)

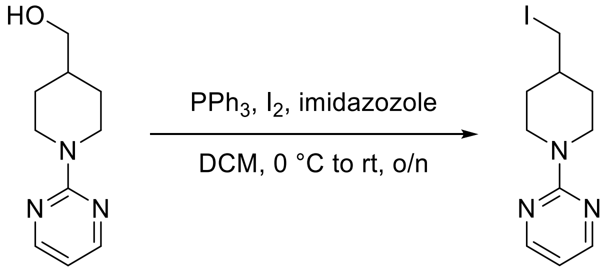

S36 **11m**

To a 100-mL two-neck flask under nitrogen were added triphenylphosphine (**5**) (1.26 g, 4.8 mmol, 1.2 equiv.) and imidazole (350 mg, 5.2 mmol, 1.3 equiv.). DCM (11 mL) was added, and the mixture was cooled to 0 °C. Then, iodine (1.22 g, 4.8 mmol, 1.2 equiv.) was added, and the mixture was stirred at 0 °C for 15 minutes. (1-(Pyrimidin-2-yl)piperidin-4-yl)methanol (**S36**) (770 mg, 4.0 mmol, 1.0 equiv.) was added as a solution in DCM (1 mL) at 0 °C. The reaction mixture stirred for 30 min at 0 °C and then allowed to warm to room temperature overnight. Then, water was added, and the phases were separated. The aqueous layer was extracted with DCM twice. The combined organic layers were washed with a saturated solution of sodium thiosulfate and then dried with magnesium sulfate. Volatile materials were evaporated under reduced pressure, and the crude product was purified by column chromatography (20% to 30% ethyl acetate in cyclohexane) to yield the title compound as a white solid (1.10 g, 91%).

^{1}H NMR (400 MHz, $CDCl_3$) δ = 8.28 (d, *J* = 4.7 Hz, 2H), 6.44 (t, *J* = 4.7 Hz, 1H), 4.78 (dt, *J* = 13.3, 2.3 Hz, 2H), 3.11 (d, *J* = 6.6 Hz, 2H), 2.89 – 2.79 (m, 2H), 2.02 – 1.87 (m, 2H), 1.83 – 1.66 (m, 1H), 1.20 (qd, *J* = 12.4, 4.1 Hz, 2H) ppm.

^{13}C{^{1}H} NMR (101 MHz, $CDCl_3$) δ = 161.6, 157.9, 109.7, 43.8, 39.2, 32.7, 13.8 ppm.

HRMS (ESI+): m/z for $C_{10}H_{15}IN_3$ $[M+H]^+$ calcd.: 304.0305, found: 304.0307.

1-Iodo-4-(2-iodoethyl)benzene (11n)

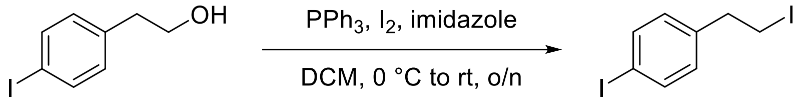

S37 **11n**

To a 50-mL two-neck flask under nitrogen were added triphenylphosphine (**5**) (1.26 g, 4.80 mmol, 1.2 equiv.) and imidazole (361 mg, 5.20 mmol, 1.3 equiv.). DCM (15 mL) was added, and the mixture was cooled to 0 °C. Then, iodine (1.22 g, 4.80 mmol, 1.2 equiv.) was added, and the mixture was stirred at 0 °C for 15 minutes. 2-(4-Iodophenyl)ethan-1-ol (**S37**) (992 mg, 4.00 mmol, 1.0 equiv.) was added at 0 °C. The reaction mixture was allowed to warm to room temperature overnight. Then, a saturated aqueous solution of sodium thiosulfate was added, and the phases were separated. The aqueous layer was extracted with DCM, and the combined organic layers were dried with sodium sulfate. Volatile materials were evaporated under reduced pressure, and the crude product was purified by column chromatography (pentane) to yield the title compound as a white solid (1.44 g, 4.00 mmol, quant.).

^{1}H NMR (400 MHz, $CDCl_3$) δ = 7.78 – 7.49 (m, 2H), 7.11 – 6.80 (m, 2H), 3.32 (td, *J* = 7.6, 0.7 Hz, 2H), 3.12 (t, *J* = 7.6 Hz, 2H) pm.

^{13}C{^{1}H} NMR (101 MHz, $CDCl_3$) δ = 140.2, 137.8, 130.5, 92.3, 39.7, 5.1 ppm.

The spectroscopic data matched those reported in the literature.[20]

(4-(Hexyloxy)phenyl)methanol (S39)

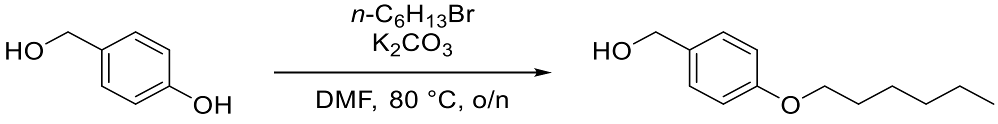

S38 **S39**

A 100-mL round-bottom flask was charged with 4-(hydroxymethyl)phenol (**S38**) (992 mg, 8.0 mmol, 1.0 equiv.), potassium carbonate (1.66 g, 12.0 mmol, 1.5 equiv, 1-bromohexane (1.35 mL, 9.6 mmol, 1.2 equiv.), and DMF (16 mL). The reaction mixture was stirred at 80 °C overnight. After cooling to room temperature, water was added, and the mixture was extracted three times with DCM. The combined organic layers were washed two times with a saturated aqueous solution of $NaHCO_3$ and three times with an aqueous solution of lithium chloride (1 M) and subsequently dried with magnesium sulfate. Volatile materials were removed under reduced pressure, and the crude product was purified by column chromatography (XX) to yield the title compound as a white solid (1.36 g, 82%).

^{1}H NMR (400 MHz, $CDCl_3$) δ = 7.30 – 7.24 (m, 2H), 6.90 – 6.85 (m, 2H), 4.60 (s, 2H), 3.95 (t, *J* = 6.6 Hz, 2H), 1.84 – 1.72 (m, 2H), 1.66 (br, 1H), 1.52 – 1.41 (m, 2H), 1.40 – 1.30 (m, 4H), 0.96 – 0.84 (m, 3H) ppm.

^{13}C{^{1}H} NMR (101 MHz, $CDCl_3$) δ = 159.0, 133.1, 128.8, 114.7, 68.2, 65.2, 31.7, 29.4, 25.9, 22.7, 14.2 ppm.

HRMS (ESI+): m/z for $C_{13}H_{20}NaO_2$ $[M+Na]^+$ calcd.: 231.1356, found: 231.1352.

1-(Bromomethyl)-4-(hexyloxy)benzene (11o)

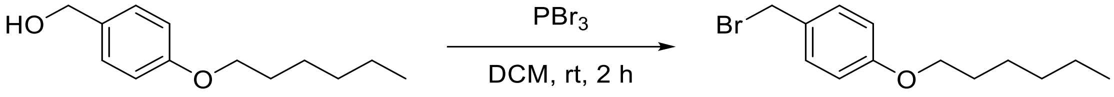

S39 **11o**

Under an atmosphere of nitrogen, a 25-mL round-bottom flask was charged with 4-(hexyloxy)phenyl)methanol (**S39**) (833 mg, 4.0 mmol, 1.0 equiv.) and DCM (8 mL). The mixture was cooled to 0 °C, and PBr_3 (570 µL, 6.0 mmol, 1.5 equiv.) was added dropwise. The reaction mixture was stirred at room temperature for two hours. Then, a saturated aqueous solution of $NaHCO_3$ was added slowly (*Note*: gas evolution). The layers were separated, and the aqueous layer was extracted with DCM. The combined organic layers were washed with water and brine before they were dried with magnesium sulfate. Volatile materials were removed under reduced pressure to yield the title compound as a yellow oil (1.0 g, 92%).

^{1}H NMR (400 MHz, $CDCl_3$) δ = 7.34 – 7.28 (m, 2H), 6.89 – 6.81 (m, 2H), 4.50 (s, 2H), 3.95 (t, *J* = 6.6 Hz, 2H), 1.77 (ddt, *J* = 9.0, 7.9, 6.6 Hz, 2H), 1.52 – 1.40 (m, 2H), 1.38 – 1.29 (m, 4H), 0.99 – 0.87 (m, 3H) ppm.

^{13}C{^{1}H} NMR (101 MHz, $CDCl_3$) δ = 159.4, 130.6, 129.8, 114.9, 68.2, 34.3, 31.7, 29.3, 25.8, 22.7, 14.2 ppm.

HRMS (ESI+): m/z for $C_{13}H_{19}O$ $[M-Br]^+$ calcd.: 191.1430, found: 191.1428.

4-(3-iodopropyl)phenol (11p)

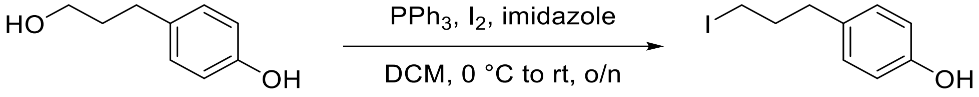

S40 **11p**

Under an atmosphere of nitrogen, a 100-mL two-neck round-bottom flask was charged with triphenylphosphine (**5**) (2.52 g, 9.6 mmol, 1.2 equiv.), imidazole (710 mg, 10.4 mmol, 1.3 equiv.), and DCM (32 mL). The mixture was cooled to 0 °C. Then, iodine (2.44 g, 9.6 mmol, 1.2 equiv.) was added, and the mixture was stirred at 0 °C for 15 min. Subsequently, primary alcohol **S40** (1.22 g, 8.0 mmol, 1.0 equiv.) was added. The reaction mixture was allowed to warm to room temperature overnight (ca. 21 h). Then, a saturated aqueous solution of sodium thiosulfate was added, and the phases were separated. The aqueous layer was extracted with DCM three times, and the combined organic layers were dried with magnesium sulfate. Volatile materials were evaporated under reduced pressure, and the crude product was purified by column chromatography (0% to 20% ethyl acetate in cyclohexane) to yield the title compound as a colorless oil that solidified upon cooling (2.06 g, 98%).

^{1}H NMR (400 MHz, $CDCl_3$) δ = 7.06 (d, *J* = 8.6 Hz, 2H), 6.76 (d, *J* = 8.5 Hz, 2H), 4.78 (s, 1H), 3.16 (t, *J* = 6.8 Hz, 2H), 2.66 (t, *J* = 7.3 Hz, 2H), 2.22 – 2.02 (m, 2H) ppm.

^{13}C{^{1}H} NMR (101 MHz, $CDCl_3$) δ = 154.0, 132.8, 129.8, 115.5, 35.4, 35.2, 6.6 ppm.

The spectroscopic data matched those reported in the literature.[21]

4-(4-Bromobutyl)benzoic acid (11q)

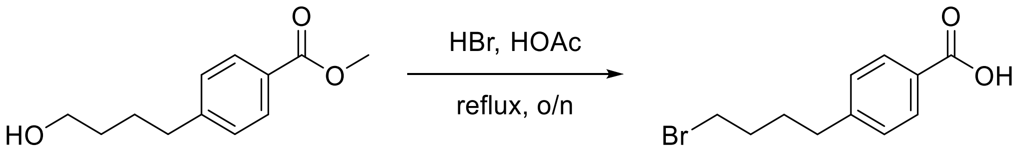

S41 **11q**

A 50-mL round-bottom flask with a reflux condenser was charged with alcohol **S41** (680 mg, 3.27 mmol, 1.0 equiv.), glacial acetic acid (6 mL), and hydrobromic acid (40% in water, 6 mL). The mixture was heated under reflux overnight (heating block set to 125 °C). After cooling to room temperature, the mixture was diluted with water, leading to precipitation. The suspension was filtered, and the residue was washed with water three times to yield the title compound as an off-white solid (700 mg, 83%).

^{1}H NMR (400 MHz, $CDCl_3$) δ = 10.80 (br, 1H), 8.04 (d, *J* = 8.3 Hz, 2H), 7.29 (d, *J* = 8.3 Hz, 2H), 3.43 (t, *J* = 6.5 Hz, 2H), 2.73 (t, *J* = 7.5 Hz, 2H), 1.95 – 1.87 (m, 2H), 1.86 – 1.77 (m, 2H) ppm.

^{13}C{^{1}H} NMR (101 MHz, $CDCl_3$) δ = 172.3, 148.5, 130.6, 128.7, 127.3, 35.2, 33.5, 32.3, 29.6 ppm.

HRMS (ESI+): m/z for $C_{11}H_{14}O_2Br$ $[M+H]^+$ calcd.: 257.0172, found: 257.0170.

5-(4-Bromobutoxy)-1*H*-pyrrolo[2,3-b]pyridine (11r)

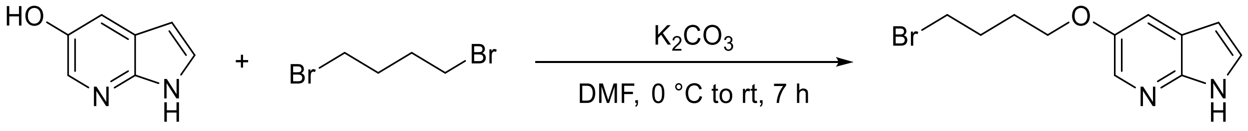

S42 **14** **11r**

A 50-mL round-bottom flask was charged with phenol **S42** (537 mg, 4.0 mmol, 1.0 equiv.), potassium carbonate (1.1 g, 8.0 mmol, 2.0 equiv.), and DMF (12 mL). Then, the mixture was cooled to 0 °C, and 1,4-dibromobutane (**14**) (0.52 mL, 4.4 mmol, 1.1 equiv.) was added. The reaction mixture was allowed to warm to room temperature over a course of seven hours. Then, water was added, and the aqueous layer was extracted three times with DCM. The combined organic layer was washed with aqueous LiCl solution (1 M) four times and then dried with magnesium sulfate. Volatile materials were removed under reduced pressure, and the residue was purified by column chromatography (20% to 40% ethyl acetate in cyclohexane) to yield the title compound as a white solid (340 mg, 32%).

^{1}H NMR (400 MHz, $CDCl_3$) δ = 9.94 (s, 1H), 8.09 (d, *J* = 2.7 Hz, 1H), 7.47 (d, *J* = 2.7 Hz, 1H), 7.33 (dd, *J* = 3.5, 1.9 Hz, 1H), 6.43 (dd, *J* = 3.6, 1.5 Hz, 1H), 4.07 (t, *J* = 6.0 Hz, 2H), 3.51 (t, *J* = 6.6 Hz, 2H), 2.16 – 2.07 (m, 2H), 2.03 – 1.93 (m, 2H) ppm.

^{13}C{^{1}H} NMR (101 MHz, $CDCl_3$) δ = 150.7, 144.2, 133.9, 126.1, 120.4, 113.2, 100.7, 68.6, 33.6, 29.6, 28.2 ppm.

HRMS (ESI+): m/z for $C_{11}H_{14}N_2OBr$ $[M+H]^+$ calcd.: 269.0284, found: 269.0279.

Alkyl chloride S44

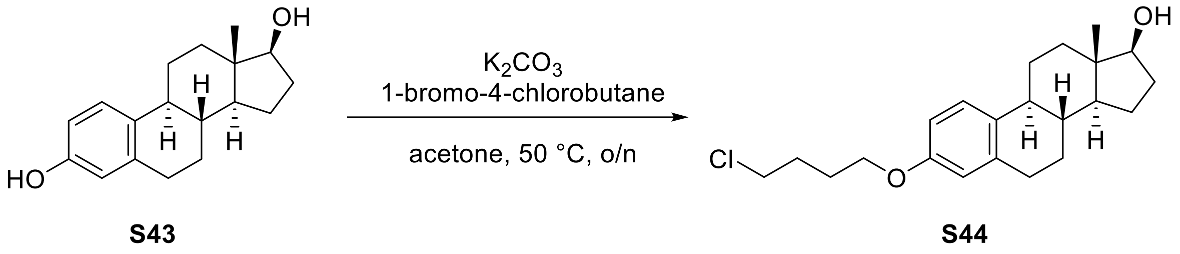

To a 100-mL round-bottom flask equipped with a reflux condenser were added β-oestradiol (**S43**) (1.09 g, 4 mmol, 1.0 equiv.), potassium carbonate (1.11 g, 8 mmol, 2.0 equiv.) and 1-bromo-4-chlorobutane (0.69 mL, 6 mmol, 1.5 equiv.), followed by acetone (20 mL). The mixture was heated under reflux (oil bath set to 50 °C). After cooling to room temperature, water was added, and the phases were separated. The aqueous layer was extracted with ethyl acetate three times. The combined organic layers were washed with brine and dried with magnesium sulfate. Volatile materials were evaporated under reduced pressure, and the crude product was purified by column chromatography (0% to 10% ethyl acetate in cyclohexane) to yield the title compound as a white solid (1.06 g, 73%).

^{1}H NMR (400 MHz, $CDCl_3$) δ = 7.20 (d, *J* = 8.5 Hz, 1H), 6.69 (dd, *J* = 8.6, 2.8 Hz, 1H), 6.62 (d, *J* = 2.5 Hz, 1H), 3.97 (t, *J* = 5.7 Hz, 2H), 3.73 (t, *J* = 8.6 Hz, 1H), 3.61 (t, *J* = 6.3 Hz, 2H), 2.92 – 2.77 (m, 2H), 2.35 – 2.25 (m, 1H), 2.23 – 2.01 (m, 2H), 2.02 – 1.83 (m, 6H), 1.75 – 1.64 (m, 1H), 1.56 – 1.37 (m, 5H), 1.37 – 1.24 (m, 2H), 1.24 – 1.14 (m, 1H), 0.78 (s, 3H) ppm.

^{13}C{^{1}H} NMR (101 MHz, $CDCl_3$) δ = 156.9, 138.2, 132.9, 126.5, 114.6, 112.1, 82.0, 67.0, 50.2, 44.9, 44.1, 43.4, 39.0, 36.9, 30.8, 29.9, 29.5, 27.4, 26.9, 26.5, 23.3, 11.2 ppm.

HRMS (ESI+): m/z for $C_{22}H_{31}O_2NaCl$ $[M+Na]^+$ calcd: 385.1905 found: 385.1907.

Alkyl iodide 11s

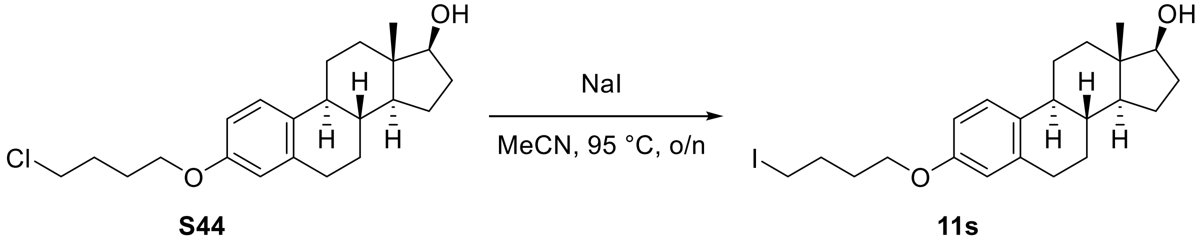

Under nitrogen, to a 25-mL two-neck flask equipped with a reflux condenser were added alkyl chloride **S44** (400 mg, 1.1 mmol, 1.0 equiv.) and sodium iodide (826 mg, 5.5 mmol, 5.0 equiv.). Acetonitrile (5 mL) was added, and the mixture was stirred at 95 °C overnight. Then, water (10 mL) was added, and the aqueous phase was extracted three times with diethyl ether. The combined organic layers were dried with sodium sulfate. Volatile materials were evaporated under reduced pressure, and the crude product was purified by column chromatography (0% to 10% ethyl acetate in cyclohexane) to yield the title compound as a white solid (451 mg, 90%).

^{1}H NMR (400 MHz, $CDCl_3$) δ = 7.20 (d, *J* = 8.6 Hz, 1H), 6.69 (dd, *J* = 8.6, 2.8 Hz, 1H), 6.62 (d, *J* = 2.8 Hz, 1H), 3.96 (t, *J* = 6.0 Hz, 2H), 3.75 (t, *J* = 8.3 Hz, 1H), 3.26 (t, *J* = 6.9 Hz, 2H), 2.88– 2.81 (m, 2H), 2.35 – 2.27 (m, 1H), 2.23 – 2.16 (m, 1H), 2.16 – 2.05 (m, 1H), 2.05 – 1.98 (m, 2H), 1.98 – 1.92 (m, 1H), 1.91 –

1.84 (m, 2H), 1.74 – 1.65 (m, 1H), 1.56 – 1.37 (m, 6H), 1.37 – 1.27 (m, 2H), 1.24 – 1.15 (m, 1H), 0.78 (s, 3H) ppm.

^{13}C{^{1}H} NMR (101 MHz, $CDCl_3$) δ = 156.9, 138.2, 132.9, 126.5, 114.6, 112.1, 82.0, 66.7, 50.2, 44.1, 43.4, 39.0, 36.9, 30.7, 30.4, 30.4, 30.00, 27.4, 26.5, 23.3, 11.2, 6.7 ppm.

HRMS (ESI+): m/z for $C_{22}H_{31}O_2NaI$ $[M+Na]^+$ calcd: 477.1261 found: 477.1262.

Alkyl bromide 11t

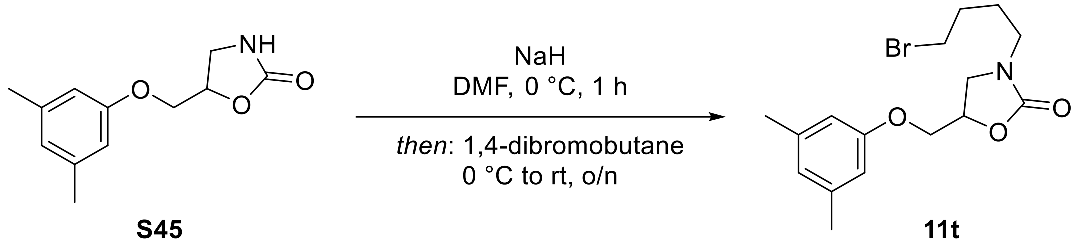

S45 **11t**

To a 50-mL two-neck round-bottom flask were added metaxalone (**S45**) (885 mg, 4.0 mmol, 1.0 equiv.) and DMF (12 mL). The mixture was cooled to 0 °C, and sodium hydride (60% in mineral oil, 192 mg, 4.8 mmol, 1.2 equiv.) was added. After stirring at 0 °C for one hour, 1,8-dibromobutane (1.4 mL, 12.0 mmol, 3.0 equiv.) was added, and the reaction mixture was allowed to warm to room temperature overnight. Then, water was added slowly and subsequently ethyl acetate as well. The layers were separated, and the organic layer was washed with an aqueous solution of lithium chloride (1 M) five times. The organic layer was dried with magnesium sulfate, and volatile materials were removed under reduced pressure. The crude product was purified by column chromatography (20% to 40% ethyl acetate in cyclohexane) to yield the title compound as a colorless oil (1.09 g, 76%) that solidified to a white solid upon standing.

^{1}H NMR (400 MHz, $CDCl_3$) δ = 6.64 (tt, *J* = 1.4, 0.7 Hz, 1H), 6.53 (dq, *J* = 1.2, 0.6 Hz, 2H), 4.87 – 4.76 (m, 1H), 4.16 – 4.02 (m, 2H), 3.70 (t, *J* = 8.8 Hz, 1H), 3.56 (dd, *J* = 8.6, 5.8 Hz, 1H), 3.46 (t, *J* = 6.4 Hz, 2H), 3.43 – 3.25 (m, 2H), 2.29 (q, *J* = 0.6 Hz, 6H), 1.98 – 1.87 (m, 2H), 1.81 – 1.69 (m, 2H) ppm.

^{13}C{^{1}H} NMR (101 MHz, $CDCl_3$) δ = 158.3, 157.8, 139.6, 123.6, 112.5, 70.9, 68.1, 46.7, 43.3, 33.3, 29.6, 25.9, 21.5 ppm.

HRMS (ESI+): m/z for $C_{16}H_{23}NO_3Br$ $[M+H]^+$ calcd: 356.0856 found: 356.0862.

Alkyl bromide 11u

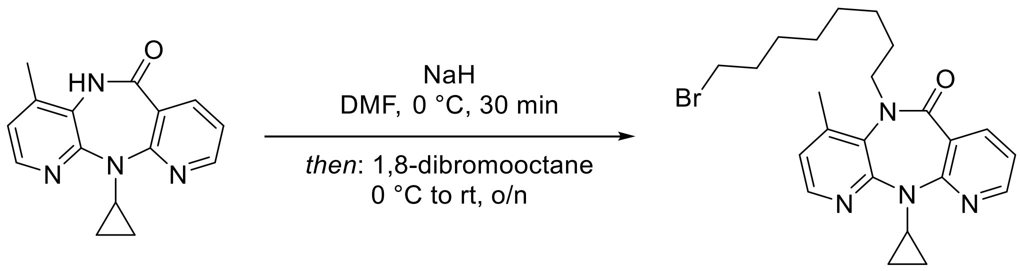

S46 **11u**

To a 50-mL two-neck round-bottom flask were added nevirapine (**S46**) (1.07 g, 4.0 mmol, 1.0 equiv.) and DMF (6 mL). The mixture was cooled to 0 °C, and sodium hydride (60% in mineral oil, 192 mg, 4.8 mmol, 1.2 equiv.) was added. After stirring at 0 °C for 30 min, 1,8-dibromooctane (2.2 mL, 12.0 mmol, 3.0 equiv.) was added, and the reaction mixture was allowed to warm to room temperature overnight. Then, water was added slowly and subsequently ethyl acetate as well. The layers were separated, and the organic layer was washed with an aqueous solution of lithium chloride (1 M) five times. The organic layer was dried with magnesium sulfate, and volatile materials were removed under reduced pressure. The crude product was purified by column chromatography (0% to 5% methanol in DCM) to yield the title compound as a light yellow oil (1.2 g, 66% (containing a minor impurity and residual cyclohexane which was used during the transfer process after the column chromatography)).

^{1}H NMR (400 MHz, $CDCl_3$) δ = 8.42 (dd, *J* = 4.8, 2.0 Hz, 1H), 8.16 (d, *J* = 4.9 Hz, 1H), 8.05 (dd, *J* = 7.6, 2.0 Hz, 1H), 7.04 (dd, *J* = 7.6, 4.8 Hz, 1H), 6.95 (dd, *J* = 4.9, 0.7 Hz, 1H), 4.57 (ddd, *J* = 13.4, 7.9, 6.5 Hz, 1H), 3.73 (tt, *J* = 6.7, 3.8 Hz, 1H), 3.36 (t, *J* = 6.8 Hz, 2H), 2.95 (ddd, *J* = 13.4, 7.9, 6.1 Hz, 1H), 2.32 (s, 3H), 1.86 – 1.76 (m, 2H), 1.49 – 1.32 (m, 8H), 1.25 – 1.18 (m,5H), 1.07 – 1.01 (m, 2H), 0.56 – 0.50 (m, 2H) ppm (more signals than expected due to overlap with residual cyclohexane).

^{13}C{^{1}H} NMR (101 MHz, $CDCl_3$) δ = 168.4, 161.1, 159.6, 150.7, 145.2, 144.2, 140.3, 128.2, 122.8, 121.9, 119.2, 49.9, 34.0, 32.8, 29.2, 28.7, 28.6, 28.2, 28.1, 26.7, 18.8, 8.9, 8.3 ppm.

HRMS (ESI+): m/z for $C_{23}H_{30}N_4OBr$ $[M+H]^+$ calcd: 457.1598 found: 457.1594.

Triphenyl(4-phenylbutyl)phosphonium bromide (13)

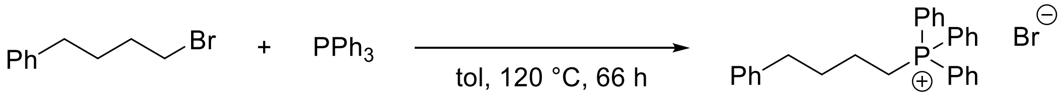

S47 **5** **13**

Under an atmosphere of nitrogen, a 16-mL vial was charged with triphenylphosphine (**5**) (1.03 g, 4.0 mmol, 1.0 equiv.), alkyl bromide **S47** (1.1 mL, 6.0 mmol, 1.5 equiv.), and toluene (1.3 mL). The mixture was heated to 120 °C for 66 hours (over weekend). After cooling to room temperature, the reaction mixture had solidified. It was dissolved with dichloromethane and loaded on silica. Purification by column chromatography (0% to 10% methanol in dichloromethane) yielded the title compound as a tan solid (1.25 g, 66%).

^{1}H NMR (400 MHz, $CDCl_3$) δ = 7.71 – 7.61 (m, 9H), 7.59 – 7.53 (m, 6H), 7.11 – 7.04 (m, 2H), 7.03 – 6.97 (m, 3H), 3.64 – 3.52 (m, 2H), 2.54 (t, *J* = 7.4 Hz, 2H), 1.87 (p, *J* = 7.4 Hz, 2H), 1.53 (h, *J* = 8.8 Hz, 2H) ppm.

^{13}C{^{1}H} NMR (101 MHz, $CDCl_3$) δ = 140.9, 134.9 (d, *J* = 3.0 Hz), 133.4 (d, *J* = 10.0 Hz), 130.3 (d, *J* = 12.5 Hz), 128.2 (d, *J* = 15.7 Hz), 125.6, 117.9 (d, *J* = 86.0 Hz), 34.5, 31.0 (d, *J* = 15.7 Hz), 22.3 (d, *J* = 50.3 Hz), 21.4 (d, *J* = 4.3 Hz) ppm (1 less signal than expected due to overlapping signals or assignment as doublet instead of two singlets).

^{31}P{^{1}H} NMR (162 MHz, $CDCl_3$) δ = 24.1 ppm.
The spectroscopic data matched those reported in the literature.[22]

Cyclopentyltriphenylphosphonium iodide (S1)

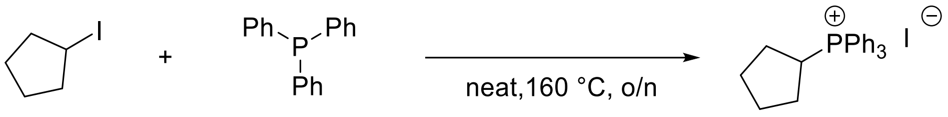

S48 **5-*d*$_{15}$** **S1**

Under an atmosphere of nitrogen, a 16-mL screw-cap vial equipped was charged with iodocyclopentane (**S48**) (1.27 mL, 11.0 mmol, 1.1 equiv.) and triphenylphosphine (**5**) (2.62 g, 10.0 mmol, 1.0 equiv.). The mixture was heated to 160 °C overnight (ca. 14 h). After cooling to room temperature, the reaction mixture had solidified. The solid was crushed and washed with diethyl ether. The resulting crude material was purified by column chromatography (5% to 10% methanol in DCM) to yield the title compound as a light yellow solid (3.27 g, 71%).

^{1}H NMR (400 MHz, $CDCl_3$) δ = 7.89 – 7.78 (m, 6H), 7.78 – 7.71 (m, 3H), 7.70 – 7.61 (m, 6H), 5.08 (h, *J* = 9.3 Hz, 1H), 2.56 – 2.42 (m, 2H), 1.89 – 1.75 (m, 2H), 1.69 – 1.52 (m, 2H), 1.34 – 1.22 (m, 2H) ppm.

^{13}C{^{1}H} NMR (101 MHz, $CDCl_3$) δ = 134.9 (d, *J* = 3.0 Hz), 134.0 (d, *J* = 9.5 Hz), 130.5 (d, *J* = 12.2 Hz), 118.8 (d, *J* = 84.5 Hz), 29.9 (d, *J* = 49.1 Hz), 28.2, 26.6 (d, *J* = 8.8 Hz) ppm.

^{31}P{^{1}H} NMR (162 MHz, $CDCl_3$) δ = 31.2 ppm.

HRMS (ESI+): m/z for $C_{23}H_{24}P$ $[M-I]^+$ calcd.: 331.1610, found: 331.16102.

3-Phenylpropyl 4-methylbenzenesulfonate (S3)

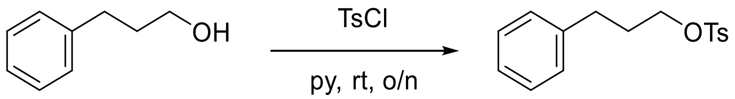

S7 **S3**

To a 25-mL round-bottom flask were added 3-phenylpropanol (**S7**) (1.36 mL, 10.0 mmol, 1.0 equiv.) and pyridine (5 mL). Then, tosyl chloride (2.86 g, 15.0 mmol, 1.5 equiv.) was added. The reaction mixture was stirred at room temperature for overnight (ca. 14 h). Then, aqueous hydrochloric acid (15%) was added until the pH was acidic. The aqueous layer was extracted three times with ethyl acetate. The combined organic layers were washed with water and brine and then dried with magnesium sulfate. Volatile materials

were removed under reduced pressure, and the residue was purified by column chromatography (0% to 10% ethyl acetate in cyclohexane) to yield the title compound as a colorless oil (440 mg, 15%).

^{1}H NMR (400 MHz, $CDCl_3$) δ = 7.82 (d, *J* = 8.3 Hz, 2H), 7.37 (d, *J* = 8.0 Hz, 2H), 7.26 (tt, *J* = 8.0, 1.6 Hz, 2H), 7.22 – 7.17 (m, 1H), 7.09 (d, *J* = 7.1 Hz, 2H), 4.06 (t, *J* = 6.2 Hz, 2H), 2.67 (t, *J* = 7.6 Hz, 2H), 2.48 (s, 3H), 2.02 – 1.94 (m, 2H) ppm.

^{13}C{^{1}H} NMR (101 MHz, $CDCl_3$) δ = 144.9, 140.5, 133.2, 130.0, 128.6, 128.5, 128.0, 126.3, 69.7, 31.6, 30.6, 21.8 ppm.

The spectroscopic data matched those reported in the literature.[19]

4. Product characterization

General procedure for the reaction of phosphonium salts with alkyl halides (GP1)

Inside a glovebox filled with argon, an 8-mL screw-cap vial was charged with phosphonium salt (0.4 mmol, 1.0 equiv.), alkyl halide (0.6 mmol, 1.5 equiv.), LiHMDS (167.3 mg, 1.0 mmol, 2.5 equiv.), and dioxane (4.0 mL). The vial was capped, taken out of the glovebox, and stirred in a pre-heated heating block at 80 °C for 16 hours. After cooling to room temperature, the reaction mixture was loaded on silica and purified by column chromatography.

General procedure for the reaction of phosphonium salts with alkyl halides including a hydroboration-oxidation post-treatment (GP2)

Inside a glovebox filled with argon, a 16-mL screw-cap vial was charged with phosphonium salt (0.3 mmol, 1.0 equiv.), alkyl halide (0.45 mmol, 1.5 equiv.), LiHMDS (125.5 mg, 0.75 mmol, 2.5 equiv.), and dioxane (3.0 mL). The vial was capped with a septum cap, taken out of the glovebox, and stirred in a pre-heated heating block at 80 °C for 16 hours. After cooling to room temperature, borane THF adduct (0.75 mL, 1.0 M in THF, 0.75 mmol, 2.5 equiv.) was added via the septum, and the reaction mixture was stirred at room temperature for two hours. Then, the reaction mixture was cooled to 0 °C and connected to a Schlenk manifold via a needle in the septum. A solution of NaOH (0.25 mL, 3.0 M in water, 0.75 mmol, 2.5 equiv.) was slowly added (*Note:* gas evolution). Subsequently, aqueous hydrogen peroxide (0.23 mL, 30% in water, 2.25 mmol, 7.5 equiv.) was slowly added (*Note:* slight exotherm). The reaction mixture was stirred at 65 °C for two hours. After cooling to room temperature, the reaction was quenched with aqueous $NaHSO_3$ (40%) and shaken. After dilution with ethyl acetate, the organic layer was removed. The remaining aqueous layer was extracted three times with ethyl acetate directly in the reaction vial. The combined organic layers were loaded on silica and purified by column chromatography.

Note: Alkene analogues of the alkane coupling products were observed as minor side products with a range of substrates. For convenience of separation, a hydroboration-oxidation treatment of the crude reaction mixture was conducted, transforming the alkenes into alcohols that show a higher difference in polarity to the alkane coupling products than the initial alkene side products.

1-(3-Cyclopentylpropyl)-4-benzene (6)

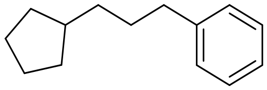

The title compound was prepared following a slightly modified general procedure GP1 (dioxane (0.2 M) instead of dioxane (0.1 M)), using cyclopentyltriphenylphosphonium bromide (**1**) (410 mg, 1.0 mmol, 1.0 equiv.) and (3-iodopropyl)benzene (**2**) (230 µL, 1.5 mmol, 1.5 equiv.). Purification by column chromatography (cyclohexane) yielded the title compound as a colorless oil (61.9 mg, 33%).

Note: The deviation from the optimized conditions is because the compound was isolated in order to generate a calibration curve before the conditions were fully optimized. The product was notably volatile under high vacuum leading to mass losses.

^{1}H NMR (400 MHz, $CDCl_3$) δ = 7.33 – 7.28 (m, 2H), 7.23 – 7.17 (m, 3H), 2.63 (t, *J* = 7.8 Hz, 2H), 1.86 – 1.73 (m, 3H), 1.70 – 1.47 (m, 6H), 1.41 – 1.34 (m, 2H), 1.16 – 1.04 (m, 2H) ppm.

^{13}C{^{1}H} NMR (101 MHz, $CDCl_3$) δ = 143.1, 128.5, 128.4, 125.7, 40.2, 36.4, 36.1, 32.8, 30.9, 25.4 ppm.

The spectroscopic data matched those reported in the literature.[23]

(6-Cyclopentylhexyl)benzene (12a)

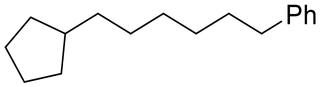

The title compound was prepared following general procedure GP1, using cyclopentyltriphenylphosphonium bromide (**1**) (164.5 mg, 0.4 mmol, 1.0 equiv.) and (6-iodohexyl)benzene (**11a**) (172.9 mg, 0.6 mmol, 1.5 equiv.). Purification by column chromatography (cyclohexane) yielded the title compound as a colorless oil (74.4 mg, 81%).

Similarly, the title compound was also prepared following general procedure GP2, using cyclopentyltriphenylphosphonium bromide (**1**) (164.5 mg, 0.4 mmol, 1.0 equiv.) and (6-bromohexyl)benzene (**S5**) (144.7 mg, 0.6 mmol, 1.5 equiv.) to yield the product as a colorless oil (72.6 mg, 79%).

^{1}H NMR (400 MHz, $CDCl_3$) δ = 7.36 – 7.28 (m, 2H), 7.25 – 7.17 (m, 3H), 2.64 (t, *J* = 7.8 Hz, 2H), 1.85 – 1.73 (m, 3H), 1.71 – 1.49 (m, 6H), 1.41 – 1.29 (m, 8H), 1.17 – 1.04 (m, 2H) ppm.

^{13}C{^{1}H} NMR (101 MHz, $CDCl_3$) δ = 143.1, 128.5, 128.4, 125.7, 40.3, 36.4, 36.2, 32.9, 31.7, 30.0, 29.5, 28.9, 25.4 ppm.

HRMS (EI+): m/z for $C_{17}H_{26}$ $[M]^+$ calcd.: 230.2029, found: 230.2028.

(4-Cyclopentylbutoxy)benzene (12b)

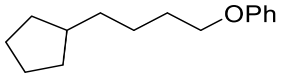

The title compound was prepared following general procedure GP1, using cyclopentyltriphenylphosphonium bromide (**1**) (164.5 mg, 0.4 mmol, 1.0 equiv.) and (4-iodobutoxy)benzene (**11b**) (165.7 mg, 0.6 mmol, 1.5 equiv.). Purification by column chromatography with $AgNO_3$-impregnated silica (cyclohexane) yielded the title compound as a colorless oil (66.0 mg, 76%).

^{1}H NMR (400 MHz, $CDCl_3$) δ = 7.34 – 7.27 (m, 2H), 6.99 – 6.91 (m, 3H), 3.98 (t, J = 6.5 Hz, 2H), 1.87 – 1.75 (m, 5H), 1.69 – 1.45 (m, 6H), 1.44 – 1.36 (m, 2H), 1.18 – 1.06 (m, 2H) ppm.

^{13}C{^{1}H} NMR (101 MHz, $CDCl_3$) δ = 159.3, 129.5, 120.6, 114.6, 68.0, 40.3, 36.1, 32.8, 29.7, 25.4, 25.3 ppm.

HRMS (EI+): m/z for $C_{15}H_{22}O$ [M]$^+$ calcd.: 218.1665, found: 218.1664.

***tert*-Butyl(3-cyclopentylpropoxy)diphenylsilane (12c)**

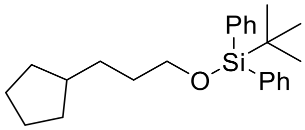

The title compound was prepared following general procedure GP1, using cyclopentyltriphenylphosphonium bromide (**1**) (164.5 mg, 0.4 mmol, 1.0 equiv.) and *tert*-butyl(3-iodopropoxy)diphenylsilane (**11c**) (254.6 mg, 0.6 mmol, 1.5 equiv.). Purification by column chromatography with $AgNO_3$-impregnated silica (cyclohexane) yielded the title compound as a colorless oil (117.1 mg, 80%).

^{1}H NMR (400 MHz, $CDCl_3$) δ = 7.71 – 7.67 (m, 4H), 7.46 – 7.36 (m, 6H), 3.67 (t, J = 6.6 Hz, 2H), 1.78 – 1.68 (m, 3H), 1.65 – 1.45 (m, 6H), 1.39 – 1.32 (m, 2H), 1.12 – 1.01 (m, 11H, overlapping signals) ppm.

^{13}C{^{1}H} NMR (101 MHz, $CDCl_3$) δ = 135.7, 134.4, 129.6, 127.7, 64.4, 40.0, 32.9, 32.4, 32.0, 27.0, 25.3, 19.4 ppm.

HRMS (ESI+): m/z for $C_{24}H_{34}NaOSi$ [M+Na]$^+$ calcd.: 389.2271, found: 389.2272.

5-(3-cyclopentylpropyl)benzo[d][1,3]dioxole (12d)

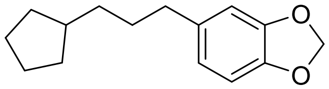

The title compound was prepared following general procedure GP2, using cyclopentyltriphenylphosphonium bromide (**1**) (123.4 mg, 0.3 mmol, 1.0 equiv.) and alkyl iodide **11d** (130.5 mg, 0.45 mmol, 1.5 equiv.). Purification by column chromatography (0% to 3% toluene in cyclohexane) yielded the title compound as a colorless oil (54.2 mg, 78%).

^{1}H NMR (400 MHz, $CDCl_3$) δ = 6.72 (d, *J* = 7.9 Hz, 1H), 6.68 – 6.60 (m, 2H), 5.91 (s, 2H), 2.56 – 2.48 (m, 2H), 1.80 – 1.69 (m, 3H), 1.64 – 1.45 (m, 6H), 1.32 (ddd, *J* = 11.7, 9.0, 5.6 Hz, 2H), 1.11 – 1.01 (m, 2H) ppm.

^{13}C{^{1}H} NMR (101 MHz, $CDCl_3$) δ = 147.6, 145.5, 137.0, 121.2, 109.0, 108.2, 100.8, 40.2, 36.1, 35.9, 32.8, 31.1, 25.3 ppm.

HRMS (ESI+): m/z for $C_{15}H_{21}O_2$ $[M+H]^+$ calcd.: 233.1536, found: 233.1531.

Amide 12e

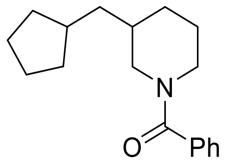

The title compound was prepared following general procedure GP1, using cyclopentyltriphenylphosphonium bromide (**1**) (82.3 mg, 0.2 mmol, 1.0 equiv.) and amide **11e** (98.8 mg, 0.3 mmol, 1.5 equiv.). Purification by column chromatography (0% to 20% ethyl acetate in cyclohexane) and subsequently reverse-phase HPLC (10% to 100% acetonitrile in water (0.1% trifluoroacetic acid)) yielded the title compound as a colorless oil (27.6 mg, 51%).

^{1}H NMR (400 MHz, $CDCl_3$, measured at ca. 293 K) δ = 7.38 (s, 5H), 4.55 (br, 1H), 3.65 (br, 1H), 2.88 (br-*pseudo*-d, *J* = 36.5 Hz, 1H), 2.54 (br-*pseudo*-d, *J* = 51.8 Hz, 1H), 2.01 –1.32 (m, 12H), 1.21 – 0.86 (m, 4H) ppm.

^{13}C{^{1}H} NMR (101 MHz, $CDCl_3$, measured at ca. 293 K) δ = 170.4, 136.7, 129.5, 128.5, 126.9, 54.0, 48.7, 48.6, 43.2, 40.5, 40.1, 37.1, 36.2, 35.2, 33.2, 33.0, 32.8, 31.8, 31.5, 26.1, 25.2 ppm.

The compound forms a mixture of rotamers at ambient temperature, leading to complex spectra. Spectra were also measured at 55 °C at which temperature most signals were better resolved.

^{1}H NMR (400 MHz, $CDCl_3$, measured at 328 K) δ = 7.38 (s, 5H), 4.46 (very br, 1H), 3.67 (very br, 1H), 2.97 – 2.83 (m, 1H), 2.63 – 2.49 (m, 1H), 1.94 – 1.87 (m, 1H), 1.84 – 1.40 (m, 10H), 1.24 – 1.11(m, 3H), 1.04 (br, 2H) ppm.

^{13}C{^{1}H} NMR (101 MHz, $CDCl_3$, measured at 328 K) δ = 170.4, 137.0, 129.4, 128.5, 127.0, 53.9 (very br), 48.7 (very br), 40.4, 37.3, 35.9 (br), 33.1, 31.8, 25.7 (br), 25.2 ppm.

HRMS (ESI+): m/z for C18H26NO [M+H]+ calcd.: 272.2009, found: 272.2003.

***tert*-Butyl 4-(cyclopentylmethyl)piperidine-1-carboxylate (12f)**

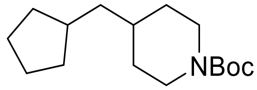

The title compound was prepared following general procedure GP1, using cyclopentyltriphenylphosphonium bromide (**1**) (164.5 mg, 0.4 mmol, 1.0 equiv.) and *tert*-butyl 4-

(iodomethyl)piperidine-1-carboxylate (**11f**) (195.1 mg, 0.6 mmol, 1.5 equiv.). Purification by column chromatography with $AgNO_3$-impregnated silica (2% to 3% ethyl acetate in cyclohexane) yielded the title compound as a colorless oil that solidified upon standing (68.7 mg, 64%).

X-Ray quality crystals were obtained by slow evaporation of dichloromethane at room temperature.

^{1}H NMR (400 MHz, $CDCl_3$) δ = 4.02 (br, 2H), 2.63 (br-t, J = 12.8 Hz, 2H), 1.89 – 1.76 (m, 1H), 1.75 – 1.66 (m, 2H), 1.65 – 1.52 (m, 4H), 1.52 – 1.44 (m, 2H), 1.42 (s, 9H), 1.40 – 1.32 (m, 1H), 1.20 (t, J = 7.1 Hz, 2H), 1.09 – 0.94 (m, 4H) ppm.

^{13}C{^{1}H} NMR (101 MHz, $CDCl_3$) δ = 155.0, 79.1, 44.2 (br), 43.3, 37.1, 35.1, 33.0, 32.6 (br), 28.6, 25.2 ppm.

HRMS (ESI+): m/z for $C_{16}H_{29}NNaO_2$ $[M+Na]^+$ calcd.: 290.2090, found: 290.2092.

Table S7. Crystal data and structure refinement for **12f**.

Deposition number	2254109
Empirical formula	$C_{16}H_{29}NO_2$
Formula weight	267.40
Temperature/K	100.0(1)
Crystal system	triclinic
Space group	P-1
a/Å	10.66780(10)
b/Å	11.94370(10)
c/Å	21.1542(2)
α/°	97.2240(10)
β/°	97.9480(10)
γ/°	115.2600(10)
Volume/Å^3	2362.26(4)
Z	6
ρ_{calc}g/cm^3	1.128
μ/mm^{-1}	0.569
F(000)	888.0
Crystal size/mm^3	0.333 × 0.181 × 0.146
Radiation	Cu Kα (λ = 1.54184)
2Θ range for data collection/°	8.366 to 159.374
Index ranges	-13 ≤ h ≤ 13, -14 ≤ k ≤ 15, -25 ≤ l ≤ 26
Reflections collected	81682
Independent reflections	10129 [R_{int} = 0.0326, R_{sigma} = 0.0172]
Data/restraints/parameters	10129/0/523
Goodness-of-fit on F^2	1.052
Final R indexes [I>=2σ (I)]	R_1 = 0.0379, wR_2 = 0.0991
Final R indexes [all data]	R_1 = 0.0407, wR_2 = 0.1010
Largest diff. peak/hole / e Å^{-3}	0.29/-0.34

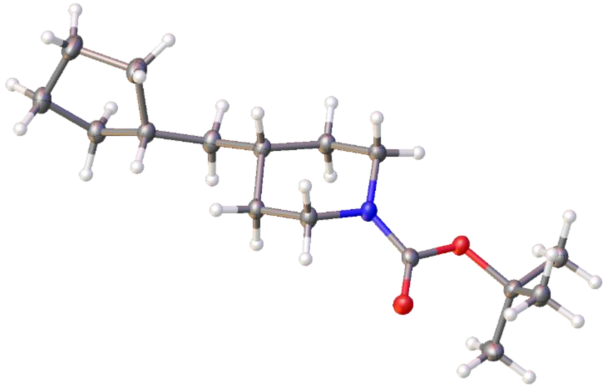

Figure S4. X-ray diffraction structure of **12f**.

1-(3-Cyclopentylpropyl)-4-iodobenzene (12g)

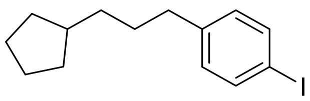

The title compound was prepared following general procedure GP1, using cyclopentyltriphenylphosphonium bromide (**1**) (123.4 mg, 0.3 mmol, 1.0 equiv.) and 1-iodo-4-(3-iodopropyl)benzene (**11g**) (167.4 mg, 0.45 mmol, 1.5 equiv.). Purification by column chromatography with $AgNO_3$-impregnated silica (cyclohexane) yielded the title compound as a colorless oil (43.6 mg, 46%).

^{1}H NMR (400 MHz, $CDCl_3$) δ = 7.73 – 7.41 (*pseudo*-td, *J* = 8.4 Hz, 2,1 Hz, 2H), 7.11 – 6.72 (*pseudo*-td, *J* = 8.4 Hz, 2,1 Hz, 2H), 2.55 (t, *J* = 7.7 Hz, 2H), 1.83 – 1.68 (m, 3H), 1.67 – 1.44 (m, 6H), 1.39 – 1.27 (m, 2H), 1.14 – 0.99 (m, 2H) ppm.

^{13}C{^{1}H} NMR (101 MHz, $CDCl_3$) δ = 142.7, 137.4, 130.7, 90.7, 40.1, 35.9, 35.9, 32.8, 30.6, 25.3 ppm.

HRMS (EI+): m/z for $C_{14}H_{19}I$ $[M]^+$ calcd.: 314.0526, found: 314.0520.

1-Bromo-4-(3-cyclopentylpropyl)benzene (12h)

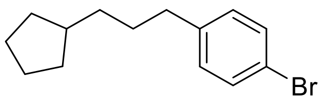

The title compound was prepared following general procedure GP2, using cyclopentyltriphenylphosphonium bromide (**1**) (123.4 mg, 0.3 mmol, 1.0 equiv.) and 1-bromo-4-(3-iodopropyl)benzene (**11h**) (146.2 mg, 0.45 mmol, 1.5 equiv.). Purification by column chromatography (cyclohexane) yielded the title compound as a colorless oil (43.6 mg, 54%).

^{1}H NMR (400 MHz, $CDCl_3$) δ = 7.38 (d, *J* = 8.4 Hz, 2H), 7.05 (d, *J* = 8.2 Hz, 2H), 2.55 (t, *J* = 7.7 Hz, 2H), 1.81 – 1.69 (m, 3H), 1.66 – 1.45 (m, 6H), 1.37 – 1.28 (m, 2H), 1.14 – 0.99 (m, 2H) ppm.

^{13}C{^{1}H} NMR (101 MHz, $CDCl_3$) δ = 142.0, 131.4, 130.3, 119.4, 40.2, 35.9, 35.8, 32.8, 30.7, 25.3 ppm.

HRMS (EI+): m/z for $C_{14}H_{19}Br$ $[M]^+$ calcd.: 266.0665, found: 266.0664.

1-Chloro-4-(3-cyclopentylpropyl)benzene (12i)

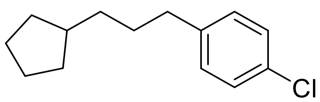

The title compound was prepared following general procedure GP2, using cyclopentyltriphenylphosphonium bromide (**1**) (123.4 mg, 0.3 mmol, 1.0 equiv.) and 1-chloro-4-(3-iodopropyl)benzene (**11i**) (126.2 mg, 0.45 mmol, 1.5 equiv.). Purification by column chromatography (cyclohexane) yielded the title compound as a colorless oil (40.0 mg, 60%).

^{1}H NMR (400 MHz, $CDCl_3$) δ = 7.24 (d, *J* = 8.4 Hz, 2H), 7.10 (d, *J* = 8.5 Hz, 2H), 2.57 (t, *J* = 7.7 Hz, 2H), 1.81 – 1.69 (m, 3H), 1.65 – 1.46 (m, 6H), 1.37 – 1.29 (m, 2H), 1.13 – 0.99 (m, 2H) ppm.

^{13}**C{^{1}H} NMR** (101 MHz, $CDCl_3$) δ = 141.5, 131.4, 129.9, 128.4, 40.2, 35.9, 35.7, 32.8, 30.7, 25.3 ppm.

HRMS (EI+): m/z for $C_{14}H_{19}Cl$ [M]$^+$ calcd.: 222.1170, found: 222.1168.

Boronic ester 12j

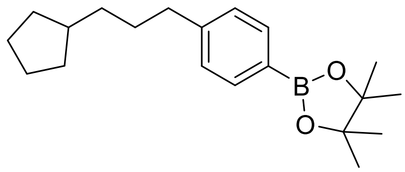

The title compound was prepared following general procedure GP1, using cyclopentyltriphenylphosphonium bromide (**1**) (123.4 mg, 0.3 mmol, 1.0 equiv.) and alkyl iodide **11j** (167.4 mg, 0.45 mmol, 1.5 equiv.). Purification by column chromatography with $AgNO_3$-impregnated silica (0% to 1% ethyl acetate in cyclohexane) yielded the title compound as a colorless oil (79.2 mg, 84%).

^{1}**H NMR** (400 MHz, $CDCl_3$) δ = 7.76 (d, *J* = 7.9 Hz, 2H), 7.22 (d, *J* = 7.9 Hz, 2H), 2.63 (t, *J* = 7.7 Hz, 2H), 1.81 – 1.71 (m, 3H), 1.71 – 1.47 (m, 6H), 1.38 – 1.33 (m, 14H), 1.13 – 1.00 (m, 2H) ppm.

^{11}**B NMR** (128 MHz, $CDCl_3$) δ = 31.3 (br) ppm.

^{13}**C{^{1}H} NMR** (101 MHz, $CDCl_3$) δ = 146.6, 135.0, 128.0, 83.7, 40.2, 36.6, 36.0, 32.8, 30.7, 25.3, 25.0 ppm. The carbon atom bound to boron is not observed due to quadrupolar relaxation.

HRMS (ESI+): m/z for $C_{20}H_{31}BNaO_2$ [M+Na]$^+$ calcd.: 337.2309, found: 337.2310.

10-(4-Cyclopentylbutyl)-10H-phenoxazine (12k)

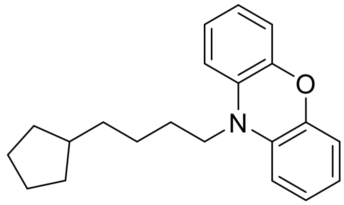

The title compound was prepared following general procedure GP2, using cyclopentyltriphenylphosphonium bromide (**1**) (123.4 mg, 0.3 mmol, 1.0 equiv.) and 10-(4-iodobutyl)-10H-phenoxazine (**11k**) (164.3 mg, 0.45 mmol, 1.5 equiv.). Purification by column chromatography with $AgNO_3$-impregnated silica (cyclohexane) yielded the title compound as a colorless oil (49.5 mg, 54%).

^{1}**H NMR** (500 MHz, $CDCl_3$) δ = 6.80 (t, *J* = 8.4 Hz, 2H), 6.64 (d, *J* = 6.3 Hz, 4H), 6.47 (d, *J* = 8.0 Hz, 2H), 3.48 (br, 2H), 1.85 – 1.73 (m, 3H), 1.72 – 1.59 (m, 4H), 1.58 – 1.50 (m, 2H), 1.47 – 1.38 (m, 4H), 1.19 – 1.06 (m, 2H) ppm.

^{13}**C{^{1}H} NMR** (126 MHz, $CDCl_3$) δ = 145.2 (br), 133.5 (br), 123.7, 120.8 (br), 115.4 (br), 111.4, 44.2 (br), 40.3, 36.0, 32.8, 26.2, 25.3 (2 overlapping signals: s and br) ppm.

HRMS (ESI+): m/z for $C_{21}H_{26}NO$ [M+H]$^+$ calcd.: 308.2009, found: 308.2001.

1-(4-Cyclopentylbutyl)-2-phenyl-1H-indole (12l)

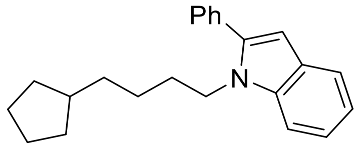

The title compound was prepared following general procedure GP1, using cyclopentyltriphenylphosphonium bromide (**1**) (123.4 mg, 0.3 mmol, 1.0 equiv.) and 1-(4-bromobutyl)-2-phenyl-1H-indole (**11l**) (147.7 mg, 0.45 mmol, 1.5 equiv.). Purification by column chromatography with $AgNO_3$-impregnated silica (cyclohexane) yielded the title compound as a brown oil (57.6 mg, 60%).

^{1}H NMR (400 MHz, $CDCl_3$) δ = 7.69 (d, *J* = 7.8 Hz, 1H), 7.57 – 7.48 (m, 4H), 7.48 – 7.41 (m, 2H), 7.28 (ddd, *J* = 8.2, 7.0, 1.2 Hz, 1H), 7.18 (ddd, *J* = 8.0, 7.0, 1.0 Hz, 1H), 6.57 (d, *J* = 0.8 Hz, 1H), 4.18 (t, *J* = 7.6 Hz, 2H), 1.78 – 1.45 (m, 9H), 1.22 (q, *J* = 4.3, 3.8 Hz, 4H), 1.07 – 0.97 (m, 2H) ppm.

^{13}C{^{1}H} NMR (101 MHz, $CDCl_3$) δ = 141.5, 137.5, 133.5, 129.6, 128.6, 128.3, 128.0, 121.6, 120.7, 119.8, 110.2, 102.1, 44.1, 40.0, 35.6, 32.7, 30.3, 26.1, 25.3 ppm.

HRMS (ESI+): m/z for $C_{23}H_{28}N$ $[M+H]^+$ calcd.: 318.2216 found: 318.2212.

2-(4-(Cyclopentylmethyl)piperidin-1-yl)pyrimidine (12m)

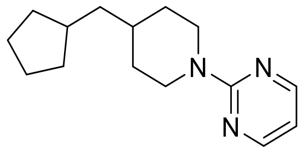

The title compound was prepared following general procedure GP1, using cyclopentyltriphenylphosphonium bromide (**1**) (164.5 mg, 0.4 mmol, 1.0 equiv.) and 2-(4-(iodomethyl)piperidin-1-yl)pyrimidine (**11m**) (181.9 mg, 0.6 mmol, 1.5 equiv.). Purification by column chromatography with $AgNO_3$-impregnated silica (3% ethyl acetate in cyclohexane) yielded the title compound as a colorless oil that solidified upon standing (74.3 mg, 76%).

X-Ray quality crystals were obtained by slow evaporation of *n*-pentane at room temperature.

^{1}H NMR (400 MHz, $CDCl_3$) δ = 8.26 (dd, *J* = 4.8, 0.9 Hz, 2H), 6.39 (td, *J* = 4.7, 0.9 Hz, 1H), 4.70 (dt, *J* = 13.3, 2.5 Hz, 2H), 2.83 (td, *J* = 12.8, 2.7 Hz, 2H), 1.87 (hept, *J* = 7.1 Hz, 1H), 1.81 – 1.69 (m, 4H), 1.64 – 1.43 (m, 5H), 1.25 (t, *J* = 7.1 Hz, 2H), 1.14 (td, *J* = 12.3, 11.7, 4.1 Hz, 2H), 1.10 – 0.98 (m, 2H) ppm.

^{13}C{^{1}H} NMR (101 MHz, $CDCl_3$) δ = 161.8, 157.8, 109.2, 44.3, 43.4, 37.1, 35.5, 33.1, 32.6, 25.2 ppm.

HRMS (ESI+): m/z for $C_{15}H_{24}N_3$ $[M+H]^+$ calcd.: 246.1965, found: 246.1966.

Table S8. Crystal data and structure refinement for **12m**.

Deposition number	2254108
Empirical formula	$C_{15}H_{23}N_3$
Formula weight	245.36
Temperature/K	100.0(1)
Crystal system	monoclinic
Space group	$P2_1/c$
a/Å	12.8746(2)
b/Å	9.41390(10)
c/Å	11.8486(2)
α/°	90
β/°	110.463(2)
γ/°	90
Volume/Å^3	1345.43(4)
Z	4
ρ_{calc}g/cm^3	1.211
μ/mm^{-1}	0.560
F(000)	536.0
Crystal size/mm^3	0.242 × 0.192 × 0.05
Radiation	Cu Kα (λ = 1.54184)
2Θ range for data collection/°	7.328 to 159.056
Index ranges	-15 ≤ h ≤ 16, -12 ≤ k ≤ 11, -14 ≤ l ≤ 14
Reflections collected	27862
Independent reflections	2883 [R_{int} = 0.0475, R_{sigma} = 0.0258]
Data/restraints/parameters	2883/0/163
Goodness-of-fit on F^2	1.065
Final R indexes [I>=2σ (I)]	R_1 = 0.0375, wR_2 = 0.0975
Final R indexes [all data]	R_1 = 0.0402, wR_2 = 0.0993
Largest diff. peak/hole / e Å^{-3}	0.25/-0.21

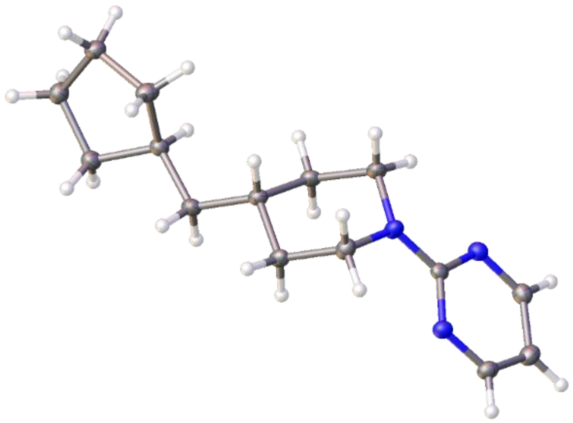

Figure S5. X-ray diffraction structure of **12m**.

1-(4-Iodophenyl)spiro[2.4]heptane (12n)

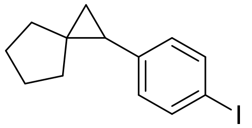

The title compound was prepared following general procedure GP1, using cyclopentyltriphenylphosphonium bromide (**1**) (164.5 mg, 0.4 mmol, 1.0 equiv.) and 1-iodo-4-(2-iodoethyl)benzene (**11n**) (214.8 mg, 0.6 mmol, 1.5 equiv.). Purification by column chromatography with $AgNO_3$-impregnated silica (cyclohexane) yielded the title compound as a colorless oil (52.2 mg, 44%).

^{1}H NMR (400 MHz, $CDCl_3$) δ = 7.57 (td, *J* = 8.4, 2.1 Hz, 2H), 6.86 – 6.82 (m, 2H), 1.93 (dd, *J* = 8.6, 6.0 Hz, 1H), 1.76 – 1.60 (m, 5H), 1.60 – 1.49 (m, 1H), 1.37 – 1.29 (m, 1H), 1.25 – 1.16 (m, 1H), 1.06 (dd, *J* = 8.6, 5.0 Hz, 1H), 0.95 (*pseudo*-t, *J* = 5.5 Hz, 1H) ppm.

^{13}C{^{1}H} NMR (101 MHz, $CDCl_3$) δ = 140.9, 137.0, 130.2, 90.2, 38.1, 31.6, 26.5, 26.4, 18.7 ppm.

HRMS (EI+): m/z for $C_{13}H_{15}I$ $[M]^+$ calcd.: 298.0213, found: 298.0210.

1-(Cyclopentylmethyl)-4-(hexyloxy)benzene (12o)

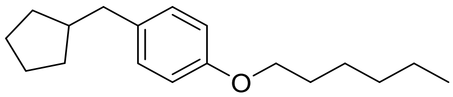

The title compound was prepared following general procedure GP2, using cyclopentyltriphenylphosphonium bromide (**1**) (123.4 mg, 0.3 mmol, 1.0 equiv.) and 1-(Bromomethyl)-4-(hexyloxy)benzene (**11o**) (122.0 mg, 0.45 mmol, 1.5 equiv.). Purification by column chromatography ($AgNO_3/SiO_2$, cyclohexane) yielded the title compound as a colorless oil (32.1 mg, 41%).

^{1}H NMR (400 MHz, $CDCl_3$) δ = 7.11 – 7.04 (m, 2H), 6.83 – 6.78 (m, 2H), 3.93 (t, *J* = 6.6 Hz, 2H), 2.54 (d, *J* = 7.4 Hz, 2H), 2.04 (hept, *J* = 7.2 Hz, 1H), 1.77 (dq, *J* = 8.0, 6.7 Hz, 2H), 1.72 – 1.58 (m, 4H), 1.57 – 1.41 (m, 4H), 1.39 – 1.29 (m, 4H), 1.24 – 1.12 (m, 2H), 0.96 – 0.87 (m, 3H) ppm.

^{13}C{^{1}H} NMR (101 MHz, $CDCl_3$) δ = 157.3, 134.5, 129.7, 114.3, 68.1, 42.3, 41.3, 32.6, 31.8, 29.5, 25.9, 25.1, 22.8, 14.2 ppm.

HRMS (ESI+): m/z for $C_{18}H_{28}ONa$ $[M+Na]^+$ calcd.: 283.2032, found: 283.2031.

4-(3-Cyclopentylpropyl)phenol (12p)

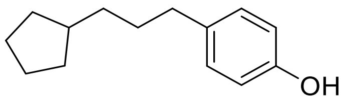

The title compound was prepared following a modified general procedure GP2 (16-mL vial instead of 8-mL vial; 4.0 equiv. LiHMDS (200.8 mg, 1.2 mmol) instead of 2.5 equiv. LiHMDS; after quenching the reaction with sodium sulfite, the reaction mixture was acidified with aqueous HCl (2 M) before the extraction step), using cyclopentyltriphenylphosphonium bromide (**1**) (123.4 mg, 0.3 mmol, 1.0 equiv.) and 4-(3-

iodopropyl)phenol (**11p**) (117.9 mg, 0.45 mmol, 1.5 equiv.). Purification by column chromatography (4% to 5% ethyl acetate in cyclohexane) yielded the title compound as a colorless oil (24.9 mg, 41%).

^{1}H NMR (400 MHz, $CDCl_3$) δ = 7.04 (dt, *J* = 8.4 , 2.5 Hz, 2H), 6.75 (dt, *J* = 8.4, 2.5 Hz, 2H), 4.76 (br, 1H), 2.53 (t, *J* = 7.7 Hz, 2H), 1.80 – 1.68 (m, 3H), 1.65 – 1.53 (m, 4H), 1.52 – 1.46 (m, 2H), 1.37 – 1.28 (m, 2H), 1.11 – 1.00 (m, 2H) ppm.

^{13}C{^{1}H} NMR (101 MHz, $CDCl_3$) δ = 153.5, 135.4, 129.6, 115.2, 40.2, 36.0, 35.5, 32.8, 31.1, 25.3 ppm.

HRMS (EI+): m/z for $C_{14}H_{20}O$ $[M]^+$ calcd.: 204.1509, found: 204.1507.

4-(4-Cyclopentylbutyl)benzoic acid (12q)

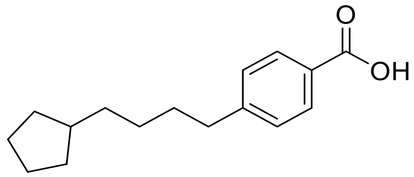

The title compound was prepared following a modified general procedure GP1 (4.0 equiv. LiHMDS (133.9.8 mg, 0.8 mmol) instead of 2.5 equiv. LiHMDS), using cyclopentyltriphenylphosphonium bromide (**1**) (82.3 mg, 0.2 mmol, 1.0 equiv.) and 4-(4-bromobutyl)benzoic acid (**11q**) (77.1 mg, 0.3 mmol, 1.5 equiv.). A different workup procedure than in GP1 was used:
After cooling to room temperature, the reaction mixture was acidified with aqueous HCl (2 M, 2 mL) and then diluted with ethyl acetate. The phases were separated, and the aqueous phase was extracted three times with ethyl acetate. The combined organic layers were loaded on silica. Purification by column chromatography (0% to 18% ethyl acetate in cyclohexane) and subsequently by reverse-phase HPLC (10% to 100% acetonitrile in water (0.1% trifluoroacetic acid)) yielded the title compound as a white solid (18.2 mg, 37%).

^{1}H NMR (400 MHz, $CDCl_3$) δ = 8.03 (d, *J* = 8.3 Hz, 2H), 7.28 (d, *J* = 8.2 Hz, 2H), 2.74 – 2.62 (t, *J* = 7.8 Hz, 2H), 1.80 – 1.68 (m, 3H), 1.68 – 1.54 (m, 4H), 1.53 – 1.44 (m, 2H), 1.40 – 1.29 (m, 4H), 1.16 – 1.00 (m, 2H) ppm.

^{13}C{^{1}H} NMR (101 MHz, $CDCl_3$) δ = 172.3, 149.7, 130.4, 128.7, 126.9, 40.2, 36.3, 36.1, 32.9, 31.5, 28.6, 25.3 ppm.

HRMS (ESI+): m/z for $C_{16}H_{23}O_2$ $[M+H]^+$ calcd.: 247.1693, found: 247.1693.

5-(4-Cyclopentylbutoxy)-1*H*-pyrrolo[2,3-b]pyridine (12r)

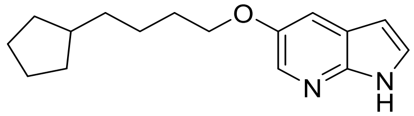

The title compound was prepared following a modified general procedure GP1 (4.0 equiv. of LiHMDS (133.9 mg, 0.8 mmol, 4.0 equiv.) instead of 2.5 equiv. LiHMDS; reaction quenched with methanol before loading on silica), using cyclopentyltriphenylphosphonium bromide (**1**) (82.3 mg, 0.2 mmol, 1.0 equiv.) and azaindole **11r** (80.7 mg, 0.3 mmol, 1.5 equiv.). Purification by column chromatography (5% to 30% ethyl

acetate in cyclohexane) and subsequently reverse-phase HPLC (10% to 100% acetonitrile in water (0.1% trifluoroacetic acid)) yielded the title compound as a lightbrown solid (35.9 mg, 69%).

^{1}H NMR (400 MHz, $CDCl_3$) δ = 11.75 (br, 1H), 8.01 (s, 1H), 7.70 (d, *J* = 2.3 Hz, 1H), 7.40 (d, *J* = 3.4 Hz, 1H), 6.50 (d, *J* = 3.4 Hz, 1H), 4.03 (t, *J* = 6.5 Hz, 2H), 1.87 – 1.72 (m, 5H), 1.67 – 1.56 (m, 2H), 1.56 – 1.44 (m, 4H), 1.40 – 1.33 (m, 2H), 1.15 – 1.03 (m, 2H) ppm.

^{13}C{^{1}H} NMR (101 MHz, $CDCl_3$) δ = 150.6, 141.2, 128.2, 127.9, 123.2, 117.8, 101.0, 70.2, 40.2, 36.0, 32.8, 29.6, 25.3, 25.3 ppm.

HRMS (ESI+): m/z for $C_{16}H_{23}N_2O$ $[M+H]^+$ calcd.: 259.1805, found: 259.1802.

Alcohol 12s

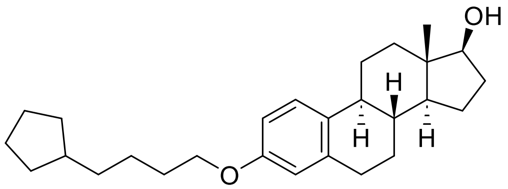

The title compound was prepared following a modified general procedure GP1 (4.0 equiv. of LiHMDS (133.9 mg, 0.8 mmol, 4.0 equiv.) instead of 2.5 equiv. LiHMDS), using cyclopentyltriphenylphosphonium bromide (**1**) (82.3 mg, 0.2 mmol, 1.0 equiv.) and alkyl iodide **11s** (136.3 mg, 0.3 mmol, 1.5 equiv.).
A different workup procedure than in GP1 was used:
After cooling to room temperature, the reaction mixture was acidified with aqueous HCl (2 M, 2 mL) and then diluted with ethyl acetate. The phases were separated, and the aqueous phase was extracted three times with ethyl acetate. The combined organic layers were loaded on silica. Purification by column chromatography (10% to 20% EtOAc in *n*-hexane) yielded the title compound as a colorless oil (48.5 mg, 61%).

^{1}H NMR (400 MHz, $CDCl_3$) δ = 7.20 (d, *J* = 9.3 Hz, 1H), 6.71 (dd, *J* = 8.6, 2.7 Hz, 1H), 6.63 (d, *J* = 2.7 Hz, 1H), 3.93 (t, *J* = 6.6 Hz, 2H), 3.73 (t, *J* = 8.3 Hz, 1H), 2.93 – 2.79 (m, 2H), 2.36 – 2.28 (m, 1H), 2.25 – 2.07 (m, 2H), 1.99 – 1.92 (m, 1H), 1.91 – 1.85 (m, 1H), 1.82 – 1.67 (m, 6H), 1.64 – 1.57 (m, 2H), 1.57 – 1.41 (m, 6H), 1.42 – 1.15 (m, 8H), 1.14 – 1.03 (m, 2H), 0.79 (s, 3H) ppm.

^{13}C{^{1}H} NMR (101 MHz, $CDCl_3$) δ = 157.2, 138.0, 132.5, 126.4, 114.6, 112.2, 82.0, 68.0, 50.2, 44.1, 43.4, 40.3, 39.0, 36.9, 36.1, 32.8, 30.7, 29.9, 29.8, 29.7, 27.4, 26.5, 25.4, 25.3, 23.3, 14.3, 11.2 ppm.

HRMS (ESI+): m/z for $C_{27}H_{41}O_2$ $[M+H]^+$ calcd: 397.3101, found: 397.31.

Oxazolidinone 12t

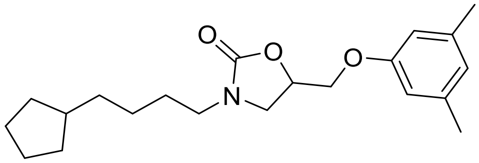

The title compound was prepared following general procedure GP1, using cyclopentyltriphenylphosphonium bromide (**1**) (61.7 mg, 0.15 mmol, 1.0 equiv.) and alkyl bromide **11t** (80.2 mg, 0.225 mmol, 1.5 equiv.). Purification by column chromatography (5% to 50% ethyl acetate in cyclohexane) and subsequently reverse-phase HPLC (50% to 100% acetonitrile in water (0.1% trifluoroacetic acid)) yielded the title compound as a colorless oil (15.1 mg, 29%).

^{1}H NMR (400 MHz, $CDCl_3$) δ = 6.67 – 6.62 (m, 1H), 6.54 – 6.50 (m, 2H), 4.80 (dtd, *J* = 8.8, 5.7, 4.2 Hz, 1H), 4.22 – 3.98 (m, 2H), 3.68 (t, *J* = 8.8 Hz, 1H), 3.53 (dd, *J* = 8.7, 5.8 Hz, 1H), 3.27 (ddt, *J* = 21.0, 13.9, 6.8 Hz, 2H), 2.28 (*pseudo*-d, *J* = 0.7 Hz, 6H), 1.84 – 1.65 (m, 3H), 1.62 – 1.53 (m, 4H), 1.53 – 1.45 (m, 2H), 1.43 – 1.29 (m, 4H), 1.18 – 0.97 (m, 2H) ppm.

^{13}C{^{1}H} NMR (101 MHz, $CDCl_3$) δ = 158.3, 157.7, 139.6, 123.5, 112.5, 70.8, 68.1, 46.9, 44.3, 40.1, 35.9, 32.8, 32.8, 27.7, 25.9, 25.3, 21.5 ppm.

HRMS (ESI+): m/z for $C_{21}H_{32}NO_3$ $[M+H]^+$ calcd: 346.2377, found: 346.2380.

Pyridine 12u

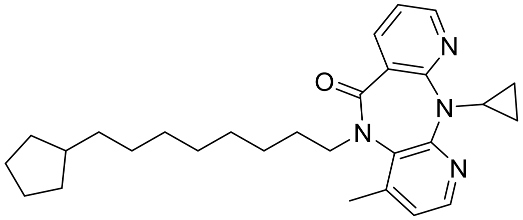

The title compound was prepared following a modified general procedure GP1 (4.0 equiv. of LiHMDS (100.4 mg, 0.6 mmol, 4.0 equiv.) instead of 2.5 equiv. LiHMDS; quenched with methanol before loading on silica), using cyclopentyltriphenylphosphonium bromide (**1**) (61.7 mg, 0.15 mmol, 1.0 equiv.) and alkyl bromide **11u** (102.9 mg, 0.225 mmol, 1.5 equiv.). Purification by column chromatography (0% to 5% methanol in DCM) and subsequently reverse-phase HPLC (30% to 100% acetonitrile in water (0.1% trifluoroacetic acid)) yielded the title compound as a white solid (10.4 mg, 16%).

^{1}H NMR (500 MHz, $CDCl_3$) δ = 8.43 (dd, *J* = 4.8, 2.0 Hz, 1H), 8.17 (d, *J* = 4.9 Hz, 1H), 8.07 (dd, *J* = 7.6, 2.0 Hz, 1H), 7.05 (dd, *J* = 7.6, 4.8 Hz, 1H), 6.96 (dd, *J* = 4.9, 0.7 Hz, 1H), 4.57 (ddd, *J* = 13.4, 8.1, 6.4 Hz, 1H), 3.73 (tt, *J* = 6.7, 3.8 Hz, 1H), 2.96 (ddd, *J* = 13.4, 8.1, 6.0 Hz, 1H), 2.33 (s, 3H), 1.77 – 1.65 (m, 2H), 1.62 – 1.51 (m, 6H), 1.50 – 1.42 (m, 2H), 1.24 – 1.17 (m, 12H), 1.08 – 0.98 (m, 4H), 0.58 – 0.51 (m, 2H) ppm (one more H than expected due to overlap with residual grease).

^{13}C{^{1}H} NMR (126 MHz, $CDCl_3$) δ = 168.4, 161.1, 159.6, 150.7, 145.1, 144.3, 140.3, 128.2, 122.8, 121.9, 119.2, 50.0, 40.3, 36.3, 32.9, 30.0, 29.6, 29.4, 28.8, 28.8, 28.2, 26.9, 25.3, 18.9, 8.9, 8.3 ppm.

HRMS (ESI+): m/z for $C_{28}H_{39}N_4O$ $[M+H]^+$ calcd: 447.3118, found: 447.3115.

(6-Cyclobutylhexyl)benzene (12v)

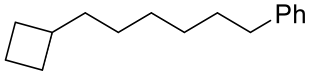

The title compound was prepared following general procedure GP2, using cyclobutyltriphenylphosphonium bromide (**10v**) (119.2 mg, 0.3 mmol, 1.0 equiv.) and (6-iodohexyl)benzene (**11a**) (129.7 mg, 0.45 mmol, 1.5 equiv.). Purification by column chromatography (cyclohexane) yielded the title compound as a light yellow oil (47.2 mg, 73%).

^{1}H NMR (400 MHz, $CDCl_3$) δ = 7.28 (t, *J* = 7.5 Hz, 2H), 7.21 – 7.15 (d, *J* = 7.0 Hz, 3H), 2.61 (t, *J* = 7.7 Hz, 2H), 2.24 (hept, *J* = 7.8 Hz, 1H), 2.09 – 1.96 (m, 2H), 1.92 – 1.73 (m, 2H), 1.67 – 1.51 (m, 4H), 1.41 – 1.25 (m, 6H), 1.24 – 1.13 (m, 2H) ppm.

^{13}C{^{1}H} NMR (101 MHz, $CDCl_3$) δ = 143.1, 128.5, 128.4, 125.7, 37.2, 36.4, 36.2, 31.7, 29.7, 29.5, 28.6, 27.3, 18.6 ppm.

HRMS (EI+): m/z for $C_{16}H_{24}$ $[M]^+$ calcd.: 216.1871, found: 216.1873.

2-(6-Phenylhexyl)norbornane (12w)

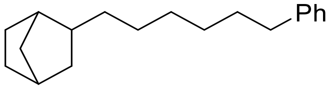

The title compound was prepared following general procedure GP1, using (2-norbornyl)triphenylphosphonium bromide (**10w**) (174.9 mg, 0.4 mmol, 1.0 equiv.) and (6-iodohexyl)benzene (**11a**) (172.9 mg, 0.6 mmol, 1.5 equiv.). Purification by column chromatography with $AgNO_3$-impregnated silica (cyclohexane) yielded the title compound as a colorless oil (69.8 mg, 68%, *dr* = 9:1).

NMR data for the major diastereomer:

^{1}H NMR (400 MHz, $CDCl_3$) δ = 7.34 – 7.29 (m, 2H), 7.22 (d, *J* = 7.4 Hz, 3H), 2.65 (t, *J* = 7.7 Hz, 2H), 2.17 (br, 1H), 2.12 (br, 1H), 1.77 (br, 2H), 1.66 (p, *J* = 7.0 Hz, 2H), 1.58 – 1.47 (m, 2H), 1.42 – 1.26 (m, 11H), 1.17 – 1.08 (m, 1H), 0.62 (dd, *J* = 6.7, 2.4 Hz, 1H) ppm.

^{13}C{^{1}H} NMR (101 MHz, $CDCl_3$) δ = 143.1, 128.5, 128.4, 125.7, 40.2, 40.1, 40.0, 37.4, 37.3, 36.2, 33.1, 31.7, 30.4, 30.0, 29.6, 29.0, 22.5 ppm.

HRMS (EI+): m/z for $C_{19}H_{28}$ $[M]^+$ calcd.: 256.2186, found: 256.2184.

The diastereoselectivity was determined by comparison of the ^{1}H NMR signals at 2.12 ppm (major isomer) and 1.98 ppm (minor isomer). The stereochemistry is tentatively assigned as *endo* based on comparison to published data for 2-methylnorbornane isomers.[24]

(6-Cyclohexylhexyl)benzene (12x)

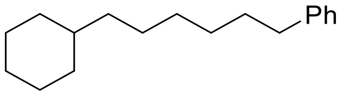

The title compound was prepared following general procedure GP2, using cyclohexyltriphenylphosphonium bromide (**10x**) (127.6 mg, 0.3 mmol, 1.0 equiv.) and (6-iodohexyl)benzene (**11a**) (129.7 mg, 0.45 mmol, 1.5 equiv.). Purification by column chromatography (cyclohexane) yielded the title compound as a colorless oil (33.5 mg, 46%).

^{1}H NMR (400 MHz, $CDCl_3$) δ = 7.31 – 7.26 (m, 2H), 7.21 – 7.15 (3H, m), 2.61 (t, *J* = 7.7 Hz, 2H), 1.73 – 1.57 (m, 7H), 1.39 – 1.08 (m, 13H), 0.93 – 0.79 (m, 2H) (one proton signal too much; presumably because of overlap with small amounts of grease) ppm.

^{13}C{^{1}H} NMR (101 MHz, $CDCl_3$) δ = 143.2, 128.7, 128.5, 125.8, 37.9, 37.8, 36.3, 33.7, 31.8, 30.1, 29.6, 27.0, 27.0, 26.7 ppm.

HRMS (EI+): m/z for $C_{18}H_{28}$ $[M]^+$ calcd.: 244.2186, found: 244.2185.

(6-Phenylhexyl)cycloheptane (12y)

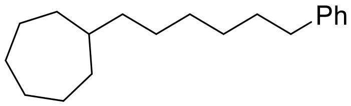

The title compound was prepared following general procedure GP1, using cycloheptyltriphenylphosphonium bromide (**10y**) (175.8 mg, 0.4 mmol, 1.0 equiv.) and (6-iodohexyl)benzene (**11a**) (172.9 mg, 0.6 mmol, 1.5 equiv.). Purification by column chromatography with $AgNO_3$-impregnated silica (cyclohexane) yielded the title compound as a colorless oil (14.7 mg, 14%).

^{1}H NMR (400 MHz, $CDCl_3$) δ = 7.31 – 7.25 (m, 2H), 7.21 – 7.15 (m, 3H), 2.61 (t, *J* = 7.7 Hz, 2H), 1.73 – 1.55 (m, 7H), 1.53 – 1.23 (m, 12H), 1.23 – 1.09 (m, 4H) ppm.

^{13}C{^{1}H} NMR (101 MHz, $CDCl_3$) δ = 143.1, 128.6, 128.4, 125.7, 39.4, 38.4, 36.2, 34.8, 31.7, 30.0, 29.6, 28.7, 27.5, 26.7 ppm.

HRMS (EI+): m/z for $C_{19}H_{30}$ $[M]^+$ calcd.: 258.2342, found: 258.2342.

Ether 12z

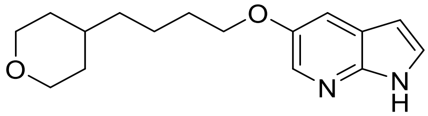

The title compound was prepared following a modified general procedure GP1 (4.0 equiv. of LiHMDS (100.4 mg, 0.6 mmol, 4.0 equiv.) instead of 2.5 equiv. LiHMDS; reaction quenched with methanol before loading on silica), using triphenyl(tetrahydro-2H-pyran-4-yl)phosphonium bromide (**10z**) (64.1 mg, 0.15 mmol, 1.0 equiv.) and azaindole **11r** (60.6 mg, 0.225 mmol, 1.5 equiv.). Purification by column

chromatography (5% to 40% ethyl acetate in cyclohexane) and subsequently reverse-phase HPLC (5% to 50% acetonitrile in water (0.1% trifluoroacetic acid)) yielded the title compound as a white solid (19.7 mg, 36%).
The product contains a small impurity (8% by ^{1}H NMR) that likely corresponds to the title compound containing an additional double bond in the molecule. The actual yield of the title compound is therefore 33%.

^{1}H NMR (400 MHz, $CDCl_3$) δ = 10.19 (s, 1H), 8.11 (s, 1H), 7.46 (d, *J* = 2.2 Hz, 1H), 7.32 (s, 1H), 6.42 (d, *J* = 3.4 Hz, 1H), 4.03 (t, *J* = 6.4 Hz, 2H), 3.96 (dd, *J* = 11.1, 4.1 Hz, 2H), 3.37 (td, *J* = 11.7, 2.1 Hz, 2H), 1.81 (p, *J* = 6.8 Hz, 2H), 1.62 (d, *J* = 12.9 Hz, 2H), 1.57 – 1.46 (m, 2H), 1.37 – 1.22 (m, 5H) ppm.

^{13}C{^{1}H} NMR (101 MHz, $CDCl_3$) δ = 150.9, 144.3, 134.0, 126.0, 120.4, 113.2, 100.5, 69.7, 68.3, 36.8, 35.1, 33.3, 29.7, 23.0 ppm.

HRMS (ESI+): m/z for $C_{13}H_{23}N_2O_2$ $[M+H]^+$ calcd.: 275.1754, found: 275.1746.

(7-Methyloctyl)benzene (12aa)

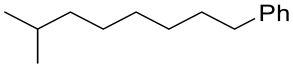

The title compound was prepared following a modified general procedure GP2 (reaction conducted at 120 °C), using *iso*-propyltriphenylphosphonium iodide (**10aa**) (129.7 mg, 0.3 mmol, 1.0 equiv.) and (6-iodohexyl)benzene (**11a**) (129.7 mg, 0.45 mmol, 1.5 equiv.). Purification by column chromatography (cyclohexane) yielded the title compound as a colorless oil (22.7 mg, 64%).

^{1}H NMR (400 MHz, $CDCl_3$) δ = 7.31 – 7.26 (m, 2H), 7.21 – 7.16 (m, 3H), 2.61 (t, *J* = 7.7 Hz, 2H), 1.67 – 1.58 (m, 2H), 1.58 – 1.47 (hept, *J* = 6.7 Hz, 1H), 1.37 – 1.25 (m, 7H), 1.20 – 1.13 (m, 2H), 0.87 (d, *J* = 6.6 Hz, 6H) ppm (one proton signal too much; presumably because of overlap with small amounts of grease).

^{13}C{^{1}H} NMR (101 MHz, $CDCl_3$) δ = 143.1, 128.6, 128.4, 125.7, 39.2, 36.2, 31.7, 29.9, 29.5, 28.1, 27.5, 22.8 ppm.

HRMS (EI+): m/z for $C_{15}H_{24}$ $[M]^+$ calcd.: 204.1873, found: 204.1870.

(4-Methylheptane-1,7-diyl)dibenzene (12ab)

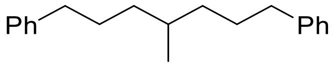

The title compound was prepared following a modified general procedure GP2 (reaction conducted at 120 °C), using phosphonium salt **10ab** (160.9 mg, 0.3 mmol, 1.0 equiv.) and 1-iodo-3-phenylpropane (**2**) (110.7 mg, 0.45 mmol, 1.5 equiv.). Purification by column chromatography ($AgNO_3/SiO_2$, cyclohexane) yielded the title compound as a colorless oil (47.8 mg, 60%).

^{1}H NMR (400 MHz, $CDCl_3$) δ = 7.31 – 7.25 (m, 4H), 7.22 – 7.14 (m, 6H), 2.65 – 2.49 (m, 4H), 1.72 – 1.50 (m, 4H), 1.50 – 1.41 (m, 1H), 1.40 – 1.30 (m, 2H), 1.24 – 1.10 (m, 2H), 0.87 (d, *J* = 6.6 Hz, 3H) ppm.

^{13}C{^{1}H} NMR (101 MHz, CDCl$_3$) δ = 143.1, 128.5, 128.4, 125.7, 36.8, 36.4, 32.7, 29.1, 19.8 ppm.

HRMS (EI+): m/z for $C_{20}H_{26}$ [M]$^+$ calcd.: 266.2029, found: 266.2029.

1-Bromo-4-(4-methyl-7-phenylheptyl)benzene (12ac)

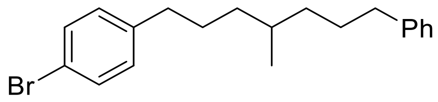

The title compound was prepared following a modified general procedure GP2 (reaction conducted at 120 °C), using (5-(4-bromophenyl)pentan-2-yl)triphenylphosphonium iodide (**10ac**) (184.6 mg, 0.3 mmol, 1.0 equiv.) and 1-iodo-3-phenylpropane (**2**) (110.7 mg, 0.45 mmol, 1.5 equiv.). Purification by column chromatography ($AgNO_3/SiO_2$, cyclohexane) yielded the title compound as a colorless oil (60.1 mg, 58%).

^{1}H NMR (400 MHz, CDCl$_3$) δ = 7.43 – 7.35 (m, 2H), 7.32 – 7.25 (m, 2H), 7.22 – 7.14 (m, 3H), 7.04 (d, *J* = 8.3 Hz, 2H), 2.68 – 2.42 (m, 4H), 1.70 – 1.49 (m, 4H), 1.48 – 1.40 (m, 1H), 1.39 – 1.25 (m, 2H), 1.22 – 1.09 (m, 2H), 0.86 (d, *J* = 6.6 Hz, 3H) ppm.

^{13}C{^{1}H} NMR (101 MHz, CDCl$_3$) δ = 143.0, 141.9, 131.4, 130.3, 128.5, 128.4, 125.8, 119.4, 36.7, 36.6, 36.4, 35.8, 32.7, 29.1, 28.9, 19.7 ppm.

HRMS (EI+): m/z for $C_{20}H_{25}Br$ [M]$^+$ calcd.: 344.1134, found: 344.1127.

***tert*-Butyl((4-methyl-7-phenylheptyl)oxy)diphenylsilane (12ad)**

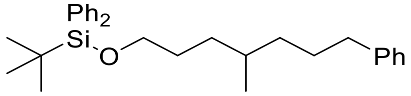

The title compound was prepared following a modified general procedure GP2 (reaction conducted at 120 °C), using phosphonium salt **10ad** (200.3 mg, 0.3 mmol, 1.0 equiv.) and 1-iodo-3-phenylpropane (**2**) (110.7 mg, 0.45 mmol, 1.5 equiv.). Purification by column chromatography ($AgNO_3/SiO_2$, cyclohexane) yielded the title compound as a colorless oil (60.8 mg, 46%).

^{1}H NMR (400 MHz, CDCl$_3$) δ = 7.69 – 7.65 (m, 4H), 7.46 – 7.34 (m, 6H), 7.31 – 7.25 (m, 2H), 7.21 – 7.14 (m, 3H), 3.64 (t, *J* = 6.6 Hz, 2H), 2.64 – 2.50 (m, 2H), 1.69 – 1.47 (m, 4H), 1.46 – 1.25 (m, 3H), 1.22 – 1.09 (m, 2H), 1.05 (s, 9H), 0.85 (d, *J* = 6.5 Hz, 3H) ppm.

^{13}C{^{1}H} NMR (101 MHz, CDCl$_3$) δ = 143.1, 135.7, 134.3, 129.6, 128.5, 128.4, 127.7, 125.7, 64.5, 36.8, 36.4, 33.0, 32.6, 30.2, 29.1, 27.0, 19.8, 19.4 ppm.

HRMS (ESI+): m/z for $C_{30}H_{41}OSi$ [M+H]$^+$ calcd.: 445.2921, found: 445.2924.

tert-Butyl((4-methylpentyl)oxy)diphenylsilane (12ae)

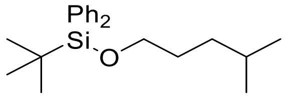

The title compound was prepared following a modified general procedure GP2 (stirred at room temperature for 15 minutes before heating; reaction conducted at 120 °C), using phosphonium salt **10ad** (200.3 mg, 0.3 mmol, 1.0 equiv.) and iodomethane (28 µL, 0.45 mmol, 1.5 equiv.). Purification by column chromatography ($AgNO_3/SiO_2$, cyclohexane) yielded the title compound as a colorless oil (49.4 mg, 48%).

^{1}H NMR (400 MHz, $CDCl_3$) δ = 7.70 – 7.66 (m, 4H), 7.45 – 7.35 (m, 6H), 3.65 (t, *J* = 6.6 Hz, 2H), 1.63 – 1.47 (m, 3H), 1.27 – 1.17 (m, 2H), 1.05 (s, 9H), 0.86 (d, *J* = 6.6 Hz, 6H) ppm.

^{13}C{^{1}H} NMR (101 MHz, $CDCl_3$) δ = 135.7, 134.4, 129.6, 127.7, 64.5, 35.1, 30.6, 27.9, 27.0, 22.8, 19.4 ppm.

HRMS (ESI+): m/z for $C_{22}H_{32}NaOSi$ $[M+Na]^+$ calcd.: 363.2115, found: 363.2115.

5-((5-Methyl-8-(phenylthio)octyl)oxy)-1H-pyrrolo[2,3-b]pyridine (12af)

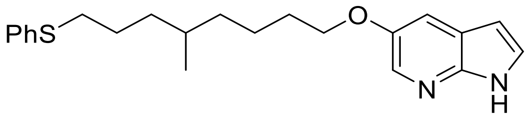

The title compound was prepared following a modified general procedure GP1 (reaction conducted at 120 °C; 4.0 equiv. LiHMDS (100.4 mg, 0.6 mmol) instead of 2.5 equiv. LiHMDS; reaction quenched with methanol before loading on silica), using phosphonium salt **10af** (85.3 mg, 0.15 mmol, 1.0 equiv.) and azaindole **11r** (60.6 mg, 0.225 mmol, 1.5 equiv.). Purification by column chromatography (5% to 40% ethyl acetate in cyclohexane) and subsequently reverse-phase HPLC (30% to 100% acetonitrile in water (0.1% trifluoroacetic acid)) yielded the title compound as a light-yellow oil (21.7 mg, 39%).

^{1}H NMR (400 MHz, $CDCl_3$) δ = 10.16 – 9.81 (br, 1H), 8.10 (d, *J* = 2.7 Hz, 1H), 7.47 (d, *J* = 2.6 Hz, 1H), 7.35 – 7.24 (m, 5H), 7.19 – 7.12 (m, 1H), 6.42 (dd, *J* = 3.5, 1.9 Hz, 1H), 4.02 (t, *J* = 6.5 Hz, 2H), 3.03 – 2.80 (m, 2H), 1.85 – 1.73 (m, 2H), 1.74 – 1.59 (m, 2H), 1.58 – 1.39 (m, 4H), 1.39 – 1.14 (m, 3H), 0.88 (d, *J* = 6.3 Hz, 3H) ppm.

^{13}C{^{1}H} NMR (101 MHz, $CDCl_3$) δ = 150.9, 144.1, 137.1, 134.0, 129.0, 129.0, 126.0, 125.8, 120.4, 113.3, 100.6, 69.7, 36.8, 36.2, 34.1, 32.6, 29.8, 26.8, 23.6, 19.7 ppm.

HRMS (ESI+): m/z for $C_{22}H_{29}N_2OS$ $[M+H]^+$ calcd.: 369.1995, found: 369.1989.

Pyrimidine 12ag

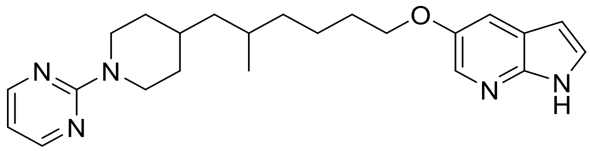

The title compound was prepared following a modified general procedure GP1 (reaction conducted at 120 °C; 4.0 equiv. LiHMDS (100.4 mg, 0.6 mmol) instead of 2.5 equiv. LiHMDS; reaction quenched with methanol before loading on silica), using phosphonium salt **10ag** (89.0 mg, 0.15 mmol, 1.0 equiv.) and azaindole **11r** (60.6 mg, 0.225 mmol, 1.5 equiv.). Purification by column chromatography (5% to 50% ethyl acetate in cyclohexane) and subsequently reverse-phase HPLC (20% to 70% acetonitrile in water (0.1% trifluoroacetic acid)) yielded the title compound as a colorless oil (18.5 mg, 31%).

^{1}H NMR (400 MHz, $CDCl_3$) δ = 10.03 (br, 1H), 8.31 (d, *J* = 4.7 Hz, 2H), 8.13 (d, *J* = 2.6 Hz, 1H), 7.49 (d, *J* = 2.5 Hz, 1H), 7.33 (dd, *J* = 3.5, 2.0 Hz, 1H), 6.44 (t, *J* = 4.7 Hz, 2H), 4.82 – 4.68 (m, 2H), 4.05 (t, *J* = 6.5 Hz, 2H), 2.95 – 2.81 (m, 2H), 1.89 – 1.70 (m, 4H), 1.69 – 1.45 (m, 4H), 1.46 – 1.33 (m, 1H), 1.30 – 1.06 (m, 5H), 0.92 (d, *J* = 6.6 Hz, 3H) ppm.

^{13}C{^{1}H} NMR (101 MHz, $CDCl_3$) δ = 161.8, 157.8, 150.9, 144.2, 134.1, 125.9, 120.4, 113.2, 109.3, 100.6, 69.8, 44.4, 44.3, 44.3, 37.3, 33.8, 33.1, 32.1, 29.9, 29.5, 23.6, 20.0 ppm.

HRMS (ESI+): m/z for $C_{23}H_{32}N_5O$ $[M+H]^+$ calcd.: 394.2601, found: 394.2590.

5. Further experiments

5.1. Cyclization reaction of phosphonium salts and alkyl bis-halides

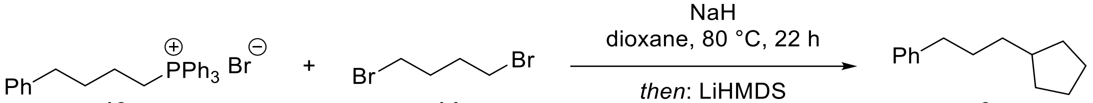

13 **14** 1.0 equiv. **6**

In a glovebox filled with argon, a 4-mL screw-cap vial was charged with triphenyl(4-phenylbutyl)phosphonium bromide (**13**) (142.6 mg, 0.3 mmol, 1.0 equiv.), sodium hydride (28.8 mg, 1.2 mmol, 4.0 equiv.), 1,4-dibromobutane (**14**) (64.8 mg, 0.3 mmol, 1.0 equiv.), and dioxane (0.6 mL). The vial was capped and stirred outside the glovebox at 80 °C for 22 h. After cooling to room temperature, the vial was taken in the glovebox again and LiHMDS (75.3 mg, 0.45 mmol, 1.5 equiv.) was added. The vial was capped again and stirred at 80 °C for 16 h. After cooling to room temperature, *n*-dodecane (45 µL) was added as internal standard, the vial was shaken, and a sample was taken for GC analysis. The yield of 1-(3-cyclopentylpropyl)-4-benzene (**6**) was determined to be 43%.

5.2. Formation of quaternary centers

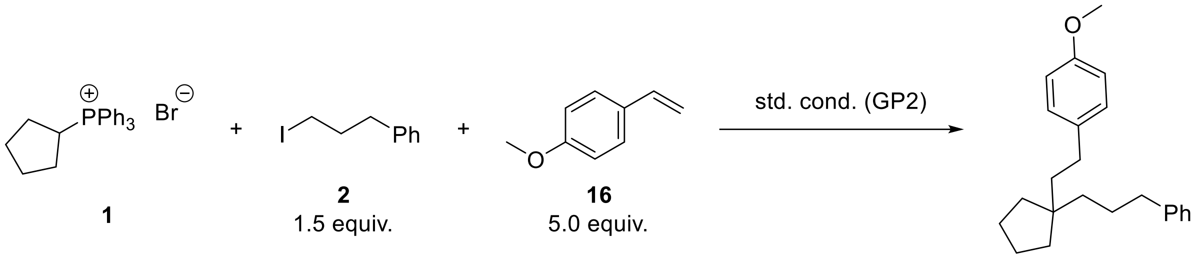

1 **2** 1.5 equiv. **16** 5.0 equiv. **15**

The product was prepared following general procedure GP2, using cyclopentyltriphenylphosphonium bromide (**1**) (123.4 mg, 0.3 mmol, 1.0 equiv.) and 1-iodo-3-phenylpropane (**2**) (100.7 mg, 0.45 mmol, 1.5 equiv.). Additionally, 1-methoxy-4-vinylbenzene (**16**) (201.3 mg, 1.5 mmol, 5.0 equiv.) was added before heating the reaction mixture. Purification by column chromatography with $AgNO_3$-impregnated silica (cyclohexane) yielded 1-methoxy-4-(2-(1-(3-phenylpropyl)cyclopentyl)ethyl)benzene (**15**) as a colorless oil (20.6 mg, 21%).

^{1}H NMR (400 MHz, $CDCl_3$) δ = 7.34 – 7.27 (m, 2H), 7.24 – 7.17 (m, 3H), 7.08 (d, *J* = 8.4 Hz, 2H), 6.83 (d, *J* = 8.7 Hz, 2H), 3.79 (s, 3H), 2.62 (t, *J* = 7.6 Hz, 2H), 2.46 – 2.40 (m, 2H), 1.69 – 1.50 (m, 8H), 1.47 – 1.36 (m, 6H) ppm.

^{13}C{^{1}H} NMR (101 MHz, $CDCl_3$) δ = 157.7, 143.0, 135.7, 129.2, 128.5, 128.4, 125.8, 113.9, 55.4, 45.1, 41.3, 38.4, 38.0, 37.0, 30.6, 27.0, 24.9 ppm.

HRMS (ESI+): m/z for $C_{23}H_{31}O$ [M+H]$^+$ calcd.: 323.2369, found: 323.2372.

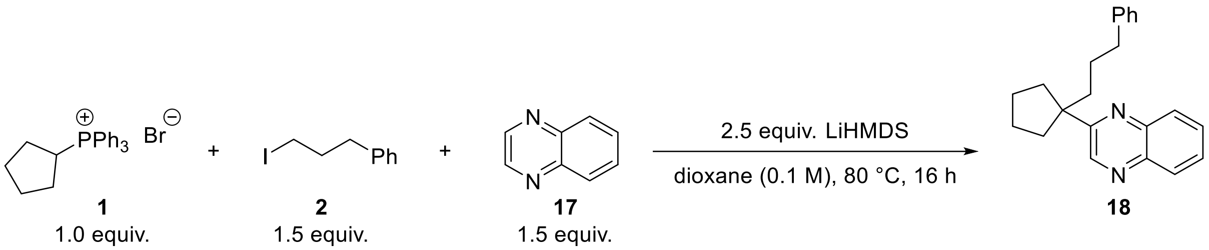

Inside a glovebox filled with argon, a 4-mL screw-cap vial was charged with cyclopentyltriphenylphosphonium bromide (**1**) (82.3 mg, 0.2 mmol, 1.0 equiv.), LiHMDS (83.7 mg, 0.5 mmol, 2.5 equiv.), 1-iodo-3-phenylpropane (**2**) (48.3 µL, 0.3 mmol, 1.5 equiv.), quinoxaline (**17**) (39.0 mg, 0.3 mmol, 1.5 equiv.), and dioxane (2.0 mL). The vial was capped, taken out of the glovebox, and stirred in a pre-heated heating block at 80 °C for 16 hours. The crude mixture was purified by column chromatography (SiO_2, 0% to 20% EtOAc in hexanes) to yield 2-(1-(3-phenylpropyl)cyclopentyl)quinoxaline (**18**) as a brown solid (12.2 mg, 19%).

^{1}H NMR (400 MHz, $CDCl_3$) δ = 8.85 (s, 1H), 8.12 – 8.02 (m, 2H), 7.81 – 7.66 (m, 2H), 7.22 – 7.17 (m, 2H), 7.15 – 7.10 (m, 1H), 7.05 – 7.01 (m, 2H), 2.50 (t, *J* = 7.7 Hz, 2H), 2.45 – 2.35 (m, 2H), 1.97 – 1.90 (m, 2H), 1.89 – 1.82 (m, 2H), 1.80 – 1.71 (m, 2H), 1.69 – 1.58 (m, 2H), 1.45 – 1.33 (m, 2H) ppm.

^{13}C{^{1}H} NMR (101 MHz, $CDCl_3$) δ = 162.6, 144.6, 142.2, 141.7, 140.5, 129.8, 129.5, 129.1, 128.9, 128.4, 128.4, 125.8, 53.1, 41.3, 37.2, 36.4, 27.5, 24.4 ppm.

HRMS (ESI+): m/z for $C_{22}H_{25}N_2$ $[M+H]^+$ calcd.: 317.2012, found: 317.2005.

5.3. Formation of a selectively deuterated product

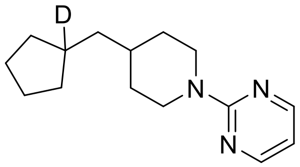

2-(4-((Cyclopentyl-1-*d*)methyl)piperidin-1-yl)pyrimidine (**12m-*d***) was prepared following general procedure GP1, using cyclopentyltris(phenyl-d_5)phosphonium bromide (**1-*d*$_{15}$**) (127.9 mg, 0.3 mmol, 1.0 equiv.) and 2-(4-(iodomethyl)piperidin-1-yl)pyrimidine (**11m**) (136.4 mg, 0.45 mmol, 1.5 equiv.). Purification by column chromatography with $AgNO_3$-impregnated silica (3% ethyl acetate in cyclohexane) yielded the product **12m-*d*** as a white solid (56.0 mg, 76%, 73% D).

The degree of deuteration was determined by comparing the integration of the peak at 1.87 ppm in the ^{1}H qNMR spectra of the protiated compound **12m** and the deuterated compound **12m-*d***. It was determined to be 73% D. The position of deuteration was determined by 2D NMR spectroscopy using spectra of the protiated compound **12m**. Due to the partial deuteration, signals belonging to **12m-*d*** and **12m** are visible in the ^{13}C{^{1}H} NMR spectrum (see overlay of spectra in the NMR section of this SI). Only signals belonging to **12m-*d*** are reported here.

^{1}H NMR (400 MHz, $CDCl_3$) δ = 8.26 (d, *J* = 4.7 Hz, 2H), 6.39 (t, *J* = 4.7 Hz, 1H), 4.70 (dt, *J* = 13.2, 2.6 Hz, 2H), 2.83 (td, *J* = 12.9, 2.6 Hz, 2H), 1.87 (hept, *J* = 7.3 Hz, 0.27H), 1.79 – 1.69 (m, 4H), 1.64 – 1.42 (m, 5H), 1.25 (m, 2H), 1.13 (td, *J* = 12.4, 4.2 Hz, 2H), 1.08 – 0.98 (m, 2H) ppm.

^{13}C{^{1}H} NMR (101 MHz, $CDCl_3$) δ = 161.8, 157.8, 109.2, 44.3, 43.3, 36.6 (t, *J* = 19.3 Hz), 35.4, 33.0, 32.6, 25.2 ppm.

HRMS (ESI+): m/z for $C_{15}H_{23}DN_3$ $[M+H]^+$ calcd.: 247.2028, found: 247.2031.

5.4. Frustrated ion pairs as halogen-atom abstraction reagents

5.4.1. Observation of dehalogenation of an alkyl iodide

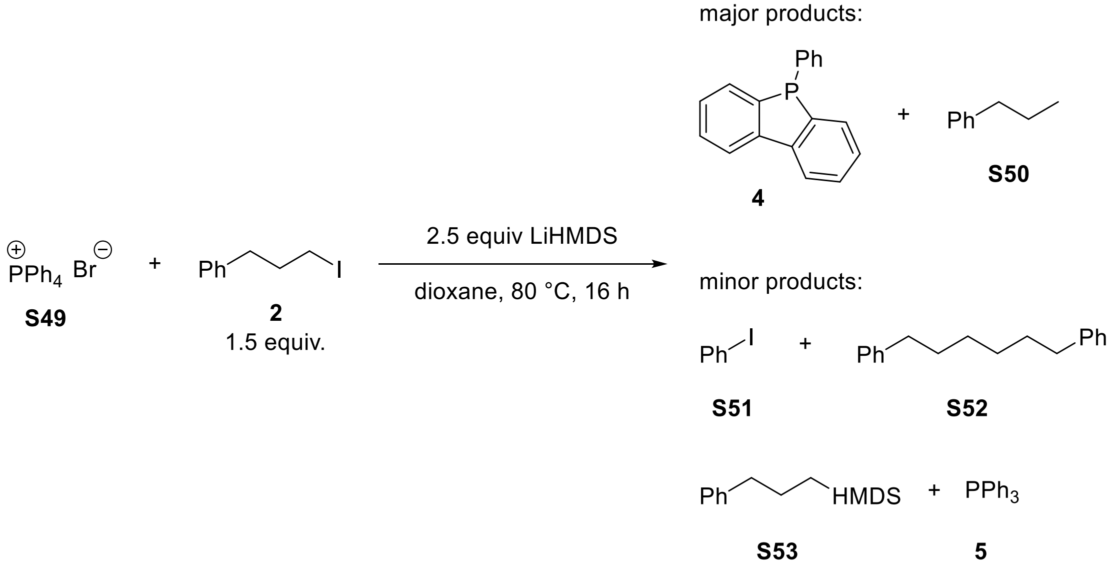

Inside a glovebox filled with argon, a 4-mL screw-cap vial was subsequently charged with tetraphenylphosphonium bromide (**S49**) (41.9 mg, 0.1 mmol, 1.0 equiv.), LiHMDS (41.8 mg, 0.25 mmol, 2.5 equiv.), 1-iodo-3-phenylpropane (**2**) (24.1 µL, 0.15 mmol, 1.5 equiv.), and dioxane (1.0 mL). The vial was taken out of the glovebox and stirred in a pre-heated heating block at 80 °C for 16 hours. After cooling to room temperature, a sample was taken for GC/MS analysis.

The observation of the dehalogenated compound **S50** as a major product in this reaction might be explained by the formation of a phenyl radical from phosphonium salt **S49** via a frustrated ion pair with LiHMDS that can undergo direct halogen-atom transfer with alkyl iodide **2** to give the corresponding alkyl radical. Alternatively, the phenyl radical can abstract a hydrogen atom from the dioxane solvent and the resulting radical can undergo XAT with substrate **2**. The formed alkyl radical can then either dimerize, leading to **S52,** or undergo hydrogen-atom transfer, leading to **S50**. Notably, iodobenzene (**S51**), potentially formed as a by-product in the aforementioned halogen-atom transfer step, was also observed in the reaction.

5.4.2. Yield of the dehydrohalogenation

Inside a glovebox filled with argon, a 4-mL screw-cap vial was subsequently charged with tetraphenylphosphonium bromide (**S49**) (62.9 mg, 0.15 mmol, 1.5 equiv.), LiHMDS (41.8 mg, 0.25 mmol, 1.5 equiv.), alkyl halide (0.1 mmol, 1.0 equiv.), and dioxane (1.0 mL). The vial was taken out of the glovebox and stirred in a pre-heated heating block at 80 °C for 16 hours. After cooling to room temperature, *n*-dodecane (15 µL) was added as an internal standard, the mixture was shaken, and a sample was taken for GC analysis.

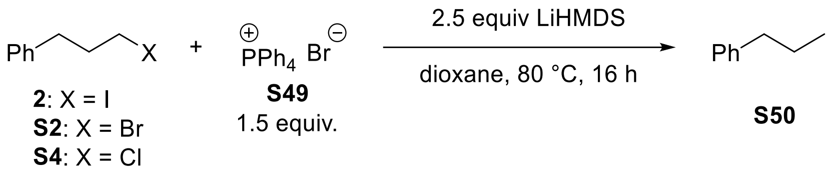

Table S9. Yield of the dehydrohalogenation of different alkyl halides.

Entry	X	Yield (%)[a]
1	I	27
2	Br	trace
3	Cl	not observed

a: Yield determined by GC using *n*-dodecane as an internal standard.

The yield of the hydrodehalogenated product **S50** was significantly lowered going from the heavy halogen iodine in **2** to the lighter halogens (**S2** and **S4**). This is in line with the propensity of the different alkyl halides to undergo halogen-atom transfer,[25] further supporting the generation of phenyl radicals from the combination of tetraphenylphosphonium bromide (**S49**) and LiHMDS.

5.4.3. Dehalogenative coupling

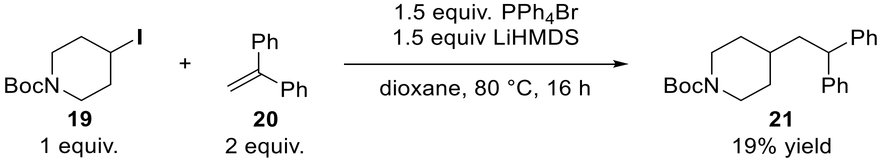

A 4-mL screw-cap vial was charged with *tert*-butyl 4-iodopiperidine-1-carboxylate (**19**) (62.2 mg, 0.2 mmol, 1.0 equiv.) before being transferred inside a glovebox filled with argon. Tetraphenylphosphonium bromide (126 mg, 0.3 mmol, 1.5 equiv.), LiHMDS (50.2 mg, 0.3 mmol, 1.5 equiv.), 1,1-diphenylethylene (**20**) (70.5 µL, 0.4 mmol, 2.0 equiv.), and dioxane (2.0 mL) were subsequently added. The vial was capped, taken out of the glovebox, and stirred in a pre-heated heating block at 80 °C for 16 hours. The crude mixture was purified by column chromatography (SiO_2, 5% EtOAc in hexanes) and subsequently reverse-phase HPLC (10% to 100% acetonitrile in water (0.1% trifluoroacetic acid)) to yield *tert*-butyl 4-(2,2-diphenylethyl)piperidine-1-carboxylate (**21**) as a light-yellow oil (14.1 mg, 19%).

^{1}H NMR (400 MHz, $CDCl_3$) δ = 7.31 – 7.21 (m, 8H), 7.20 – 7.15 (m, 2H), 4.10 – 3.97 (m, 3H), 2.62 – 2.52 (m, 2H), 2.06 – 1.92 (m, 2H), 1.75 – 1.65 (m, 2H), 1.44 (s, 9H), 1.37 – 1.24 (m, 1H), 1.14 (qd, *J* = 12.4, 4.3 Hz, 2H) ppm.

^{13}C{^{1}H} NMR (101 MHz, $CDCl_3$) δ = 155.0, 145.0, 128.6, 127.9, 126.3, 79.4, 48.0, 44.0, 42.8, 33.6, 32.3, 28.6 ppm.

The spectroscopic data matched those reported in the literature.[26]

5.5. Reaction using a LiHMDS solution

Inside a glovebox filled with argon, a 4-mL screw-cap vial was subsequently charged with cyclopentyltriphenylphosphonium bromide (**1**) (41.1 mg, 0.1 mmol, 1.0 equiv.), 1-iodo-3-phenylpropane (**2**) (24.1 µL, 0.15 mmol, 1.5 equiv.), and dioxane (0.75 mL). The vial was capped with a septum cap and taken out of the glovebox. LiHMDS (1.0 M solution in THF, 0.25 mL, 0.25 mmol, 2.5 equiv.) was added through the septum cap by syringe, and the vial was stirred in a pre-heated heating block at 80 °C for 16 hours. After cooling to room temperature, *n*-dodecane (15 µL) was added as an internal standard, the mixture was shaken, and a sample was taken for GC analysis. The product **6** was formed in 87% GC yield.

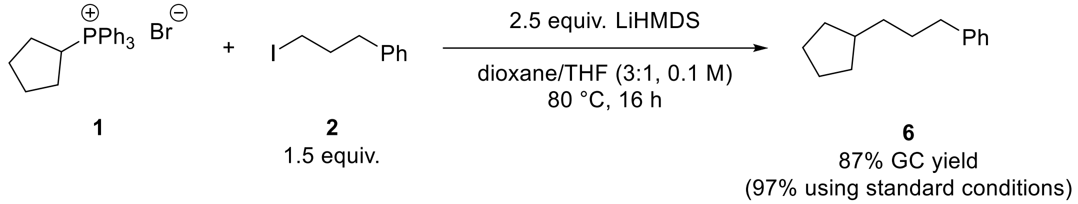

5.6. Catalytic reaction

Inside a glovebox filled with argon, a 4-mL screw-cap vial was subsequently charged with cyclopentyltriphenylphosphonium bromide (**1**) (41.1 mg, 0.1 mmol, 1.0 equiv.), LiHMDS (3.3 mg, 0.02 mmol, 0.2 equiv.), base (0.25 mmol, 2.5 equiv.), 1-iodo-3-phenylpropane (**2**) (24.1 µL, 0.15 mmol, 1.5 equiv.), and dioxane (1.0 mL). The vial was taken out of the glovebox and stirred in a pre-heated heating block at 80 °C for 16 hours. After cooling to room temperature, *n*-dodecane (15 µL) was added as an internal standard, the mixture was shaken, and a sample was taken for GC analysis.

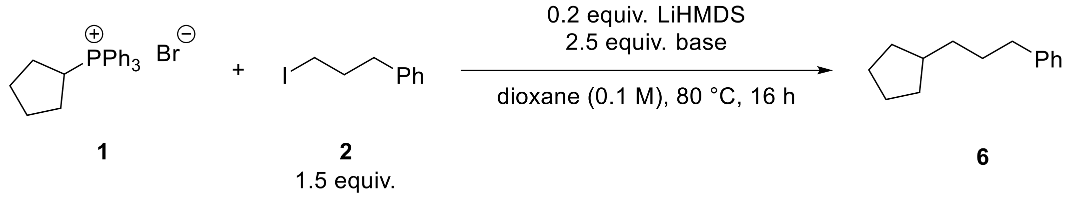

Table S10. Attempted catalytic versions of the reaction.

Entry	base	Other deviation	Yield (%)[a]
1	KHMDS	-	24
**2**	**NaH**	**-**	**51**
3	LDA	-	27
4	KO*t*Bu	-	27
5	NaH	No LiHMDS	traces

a: Yield determined by GC using *n*-dodecane as an internal standard. b: A new, previously unused stir bar was used for the reaction.

These unoptimized conditions (entry 2) show that the reaction can in principle also be conducted in catalytic fashion using NaH as the stoichiometric base. A control experiment (entry 5) demonstrates that NaH is not effective at forming the product **6** and that the reaction is therefore enabled by the catalytic amount of LiHMDS.

5.7. Reactions of further substrates

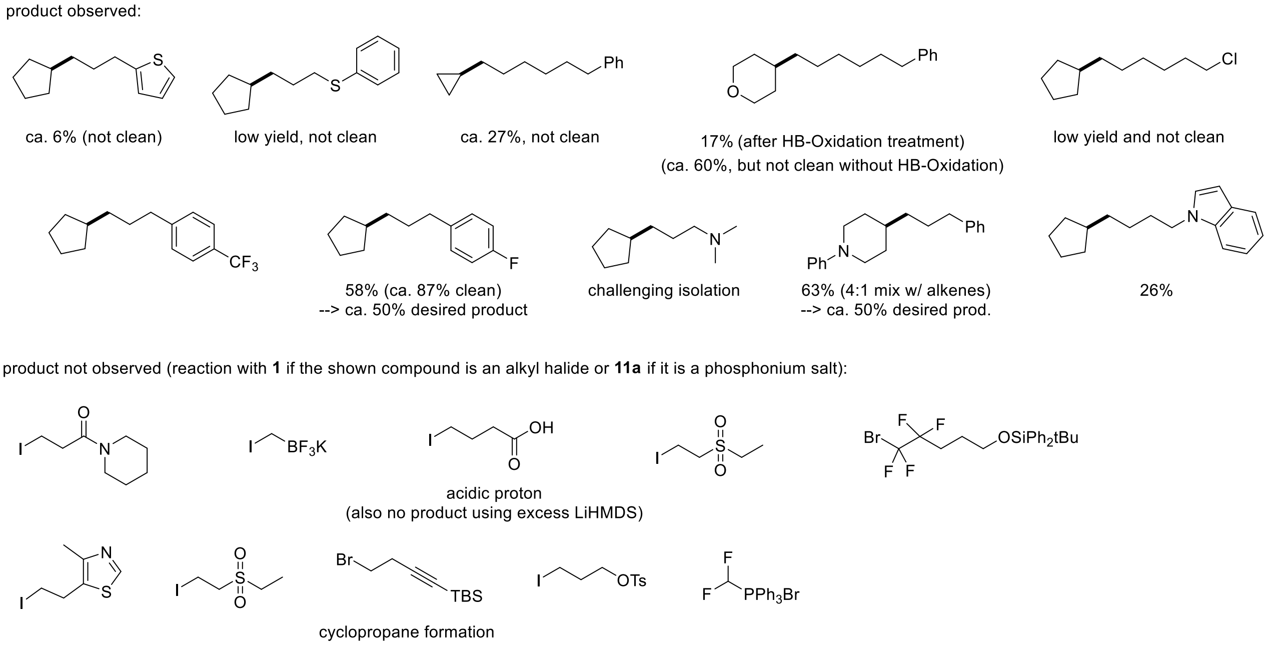

Figure S6. Further substrates tested under the conditions of GP1.

5.8. Attempted hydrolysis of *tert*-alkylphosphonium salts

A 4-mL screw-cap vial was charged with phosphonium salt (0.25 mmol, 1.0 equiv.), acetonitrile (1.25 mL), and aqueous sodium hydroxide solution (2 M, 0.25 mL, 0.5 mmol, 2.0 equiv.). The vial was capped and heated to 100 °C for 20 hours.

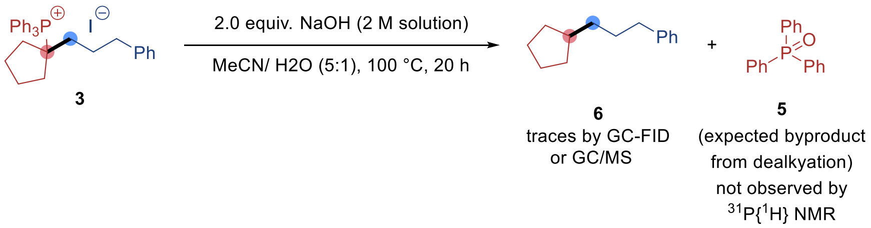

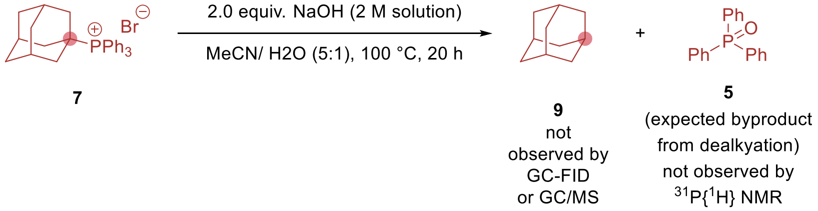

The data shows that the hydrolysis of the phosphonium salts in this work does not lead to the desired alkane products.

5.9. Additive compatibility screen

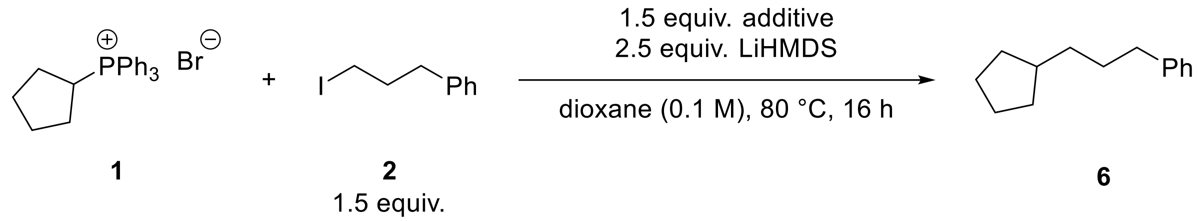

Inside a glovebox filled with argon, a 4-mL screw-cap vial was subsequently charged with cyclopentyltriphenylphosphonium bromide (**1**) (41.1 mg, 0.1 mmol, 1.0 equiv.), LiHMDS (41.8 mg, 0.25 mmol, 2.5 equiv. or 66.9 mg, 0.4 mmol, 4.0 equiv.), an additive (0.15 mmol, 1.5 equiv.), dioxane (1.0 mL), and 1-iodo-3-phenylpropane (**2**) (24.1 µL, 0.15 mmol, 1.5 equiv.). The vial was capped, taken out of the glovebox, and stirred in a pre-heated heating block at 80 °C for 16 hours. After cooling to room temperature, *n*-dodecane (15 µL) was added as an internal standard. The mixture was shaken, and a sample was taken for GC analysis.

For some of the additives, the conversion of the additive was determined. If the additive was volatile enough, a calibration curve against *n*-dodecane was constructed, and the additive conversion was determined by GC analysis. Otherwise, the additive conversion was determined by ^{1}H NMR spectroscopy (D1 = 30 s) using 1,3,5-trimethoxybenzene as internal standard.
To determine the conversion of additives containing acidic sites that could potentially be deprotonated under the reaction conditions, a different workup procedure was used:
After cooling to room temperature, *n*-dodecane (15 µL) (and 1,3,5-trimethoxybenzene if required) was added as an internal standard. Then, hydrochloric acid (2 M, 1 mL) was added, and the aqueous layer was extracted three times with ethyl acetate. A sample was taken for GC analysis. For additives that require NMR analysis instead of GC analysis, volatile materials were removed under reduced pressure, and the residue was dissolved in $CDCl_3$ or CD_3OD for ^{1}H qNMR analysis (D1 = 30 s).

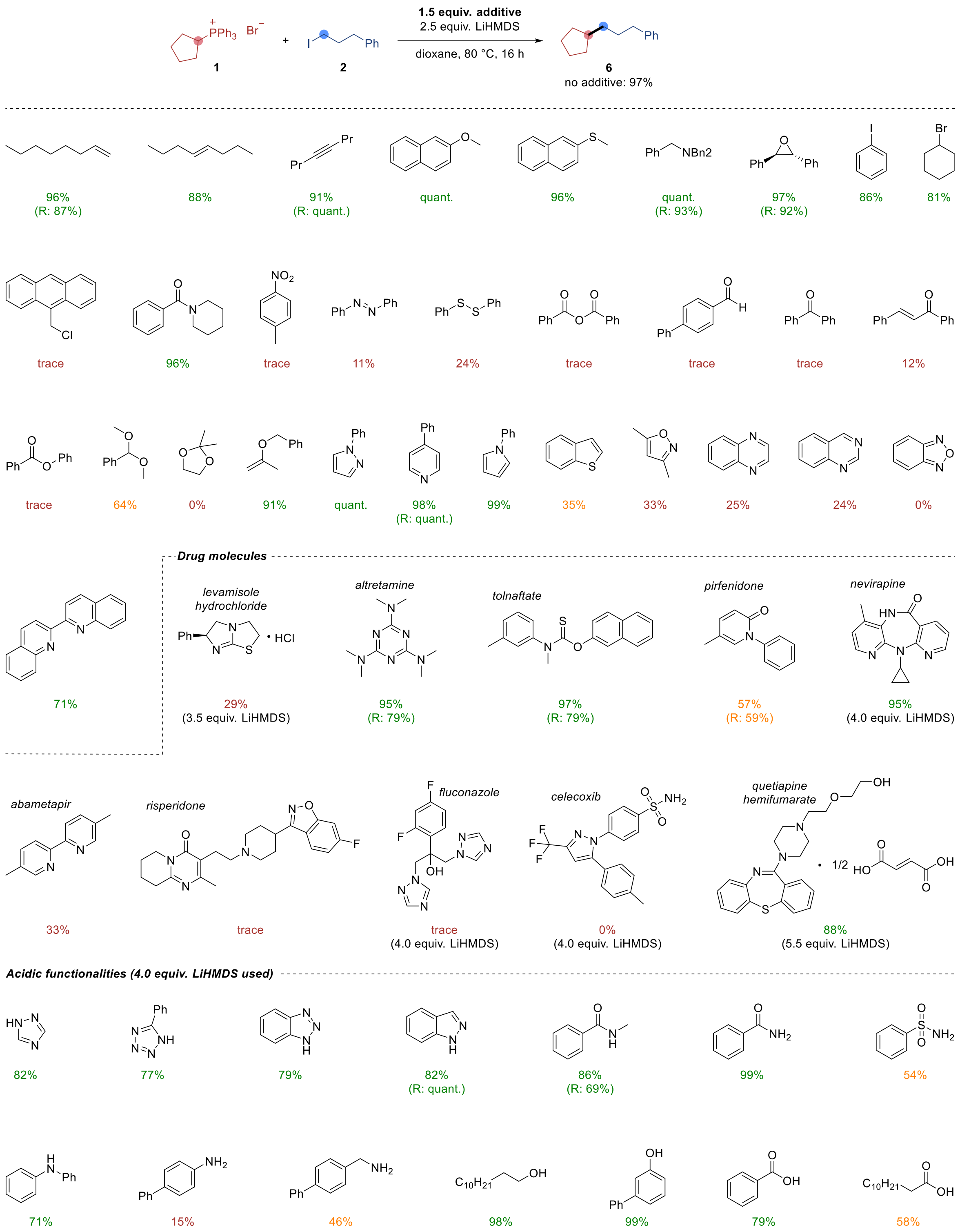

Figure S7. Additive compatibility screen. Yields were determined by GC using *n*-dodecane as internal standard and correspond to those of the coupling product when the reaction was conducted in the presence of the shown additive. Additive recovery (R) was determined for certain additives and is given in parentheses. It is normalized to account for 1.5 equiv. of the additive being used.

6. Mechanistic experiments

6.1. Identification of the major phosphorus-containing byproduct

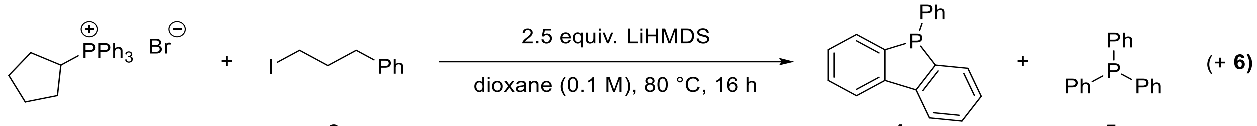

1 **2** 1.5 equiv. **4** 86% NMR yield **5** 9% NMR yield

Inside a glovebox filled with argon, a 4-mL screw-cap vial was subsequently charged with cyclopentyltriphenylphosphonium bromide (**1**) (41.1 mg, 0.1 mmol, 1.0 equiv.), LiHMDS (41.8 mg, 0.25 mmol, 2.5 equiv.), 1-iodo-3-phenylpropane (**2**) (24.1 µL, 0.15 mmol, 1.5 equiv.), and dioxane (1.0 mL). The vial was capped, taken out of the glovebox, and stirred in a pre-heated heating block at 80 °C for 16 hours. After cooling to room temperature, triphenyl phosphate was added as an internal standard, the mixture was shaken, and a sample was taken for $^{31}P\{^1H\}$ qNMR analysis. Dibenzophosphole **4** was formed in 86% and triphenylphosphine (**5**) was formed in 9%.

The identity of **4** was confirmed by comparison with an authentic sample prepared according to a literature procedure[27]:

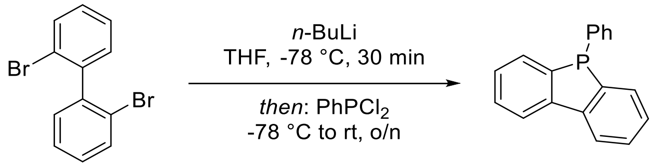

S54 **4**

Under an atmosphere of nitrogen, a 10-mL Schlenk flask was charged with 2,2'-dibromobiphenyl (**S54**) (156 mg, 0.5 mmol, 1.0 equiv.) and THF (1.0 mL). After cooling to -78 °C, *n*-buthyllithium (1.6 M in hexanes, 0.66 mL, 1.05 mmol, 2.1 equiv.) was slowly added. After stirring at -78 °C for 30 minutes, dichlorophenylphosphine (68 µL, 0.5 mmol, 1.0 equiv.) was added. The reaction mixture was stirred overnight, slowly warming from -78 °C to room temperature. Then, saturated aqueous ammonium chloride was added and the reaction mixture was stirred for 5 min. The aqueous layer was extracted three times with ethyl acetate. The combined organic layers were dried with magnesium sulfate, and volatile materials were evaporated under reduced pressure. The crude material was purified by column chromatography (cyclohexane) to yield dibenzophosphole **4** as a white solid (68.4 mg, 53%).

^{1}H NMR (400 MHz, $CDCl_3$) δ = 7.99 (dd, *J* = 7.8, 1.0 Hz, 2H), 7.79 – 7.71 (m, 2H), 7.50 (td, *J* = 7.6, 1.2 Hz, 2H), 7.41 – 7.31 (m, 4H), 7.31 – 7.23 (m, 3H) ppm.

$^{13}C\{^1H\}$ NMR (101 MHz, $CDCl_3$) δ = 143.8 (d, *J* = 3.0 Hz), 142.7 (d, *J* = 2.7 Hz), 136.3 (d, *J* = 18.9 Hz), 132.8 (d, *J* = 20.1 Hz), 130.6 (d, *J* = 21.9 Hz), 129.4 (d, *J* = 1.1 Hz), 128.8, 128.8 (d, *J* = 7.6 Hz), 127.7 (d, *J* = 7.7 Hz), 121.5 ppm.

$^{31}P\{^1H\}$ NMR (162 MHz, $CDCl_3$) δ = -10.1 ppm.

The spectroscopic data matched those reported in the literature.[27]

6.2. Test for base-mediated cyclization of triphenylphosphine (5)

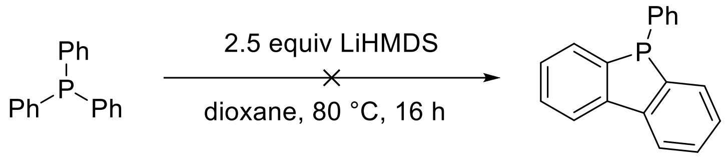

In a glovebox filled with argon, a 4-mL screw-cap vial was subsequently charged with triphenylphosphine (**5**) (26.2 mg, 0.1 mmol, 1.0 equiv.), LiHMDS (41.8 mg, 0.25 mmol, 2.5 equiv.), and dioxane (1.0 mL). The vial was taken out of the glovebox and stirred in a pre-heated heating block at 80 °C for 16 hours. After cooling to room temperature, the reaction mixture was analyzed by GC and NMR. Dibenzophosphole **4** was not observed.

6.3. Synthesis and reactivity of the alpha-tertiary phosphonium salt 3

6.3.1. Preparation of phosphonium salt **3**

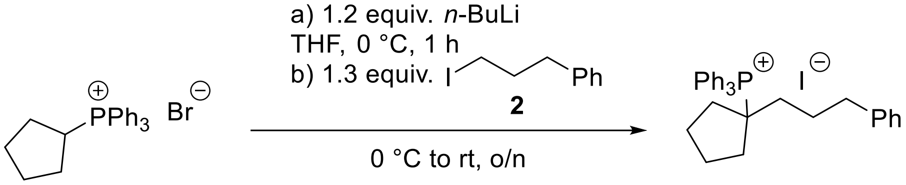

Under an atmosphere of nitrogen, a 25-mL round-bottom flask was charged with cyclopentyltriphenylphosphonium bromide (**1**) (617 mg, 1.5 mmol, 1.0 equiv.) and THF (6 mL). The flask was cooled to 0 °C and *n*-BuLi (1.6 M in hexanes, 1.1 mL, 1.8 mmol, 1.2 equiv.) was added dropwise. The reaction mixture was stirred at 0 °C for one hour. Then, 1-iodo-3-phenylpropane (**2**) (320 µL, 2.0 mmol, 1.3 equiv.) was added dropwise. The reaction mixture was allowed to warm to room temperature overnight (ca. 14 h). Afterwards, the reaction mixture was quenched with a saturated aqueous solution of ammonium chloride and stirred an additional 10 minutes. The phases were separated and the aqueous layer was extracted three times with DCM. The combined organic layers were washed two times with water and then dried with magnesium sulfate. Volatile materials were removed under reduced pressure, and the crude product was purified by column chromatography (0% to 4% methanol in DCM) to give the title compound as a light yellow solid (656 mg, 76%, 92% purity (remaining **1**)). The purity of **3** was determined by ^{31}P{^{1}H} qNMR analysis.

^{1}H NMR (400 MHz, $CDCl_3$) δ = 7.86 – 7.80 (m, 3H), 7.75 – 7.69 (m, 6H), 7.64 – 7.57 (m, 6H), 7.24 – 7.14 (m, 3H), 7.07 – 7.03 (m, 2H), 2.57 (t, *J* = 6.7 Hz, 2H), 2.44 – 2.19 (m, 4H), 1.90 – 1.66 (m, 6H), 1.56 – 1.43 (m, 2H) ppm.

^{13}C{^{1}H} NMR (101 MHz, $CDCl_3$) δ = 141.1, 135.2 (d, *J* = 3.0 Hz), 134.5 (d, *J* = 8.7 Hz), 130.7 (d, *J* = 11.8 Hz), 128.5 (d, *J* = 7.8 Hz), 126.1, 118.0 (d, *J* = 80.5 Hz), 45.4 (d, *J* = 42.2 Hz), 38.3 (d, *J* = 2.7 Hz), 36.0, 35.4, 27.2 (d, *J* = 6.9 Hz), 26.2 (d, *J* = 12.3 Hz) ppm (1 less signal than expected due to overlapping signals or assignment as doublet instead of two singlets).

^{31}P{^{1}H} NMR (162 MHz, $CDCl_3$) δ = 38.1 ppm.

HRMS (ESI+): m/z for $C_{32}H_{34}P$ [M-I]$^+$ calcd.: 449.2393, found: 449.2392.

6.3.2. Reactivity of phosphonium salt **3**

Inside a glovebox filled with argon, a 4-mL screw-cap vial was subsequently charged with phosphonium salt **3** (57.7 mg, 0.1 mmol, 1.0 equiv.), base (0.15 mmol, 1.5 equiv.), and dioxane (1.0 mL). The vial was taken out of the glovebox and stirred in a pre-heated heating block at 80 °C for 18 hours. After cooling to room temperature, *n*-dodecane (15 µL) was added as an internal standard, the mixture was shaken, and a sample was taken for GC analysis.

Note: Only 1.5 equiv. instead of 2.5 equiv. of base were used in reactions of phosphonium salt **3** as 1 equiv. of base is used in the alkylation step of the standard reaction (general procedure GP1). As **3** already contains a tertiary alkyl group, the amount of base was reduced for reactions using **3**.

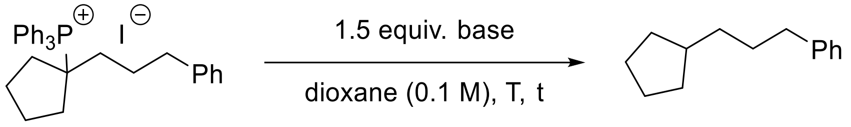

3
(92% purity)

6

Table S11. Reactivity of phosphonium salt **3**.

Entry	Base	Temperature (°C)	Time (h)	Yield (%)[a]
1	LiHMDS	80	18	72
2	LiHMDS	80	2	43
3	LiHMDS	60	18	22
4	LiHMDS	rt	18	trace
5	-	80	18	trace
6	-	80	18	trace
7	NaH	80	18	trace
8	DABCO	80	18	not detected
9	NaOMe	80	18	not detected
10	LDA	80	18	37
11	$LiNMe_2$	80	18	25
12	$LiNH_2$	80	18	13

a: Yield determined by GC using *n*-dodecane as an internal standard.

The reactivity of salt **3** correlates well with the reactivity of the overall reaction (table S1 and table S5), indicating that the identity of the base is important for the C–P bond cleavage. Notably, other amide bases (entries 10-12) performed worse than LiHMDS (entry 1), and the yield decreased with decreasing size of the base. This highlights that a large amide base is important to facilitate the reactivity.

6.4. NMR studies

Inside a glovebox filled with argon, a 4-mL screw-cap vial was subsequently charged with cyclopentyltriphenylphosphonium bromide (**1**) (20.6 mg, 0.05 mmol, 1.0 equiv.), LiHMDS (20.9 mg, 0.125 mmol, 2.5 equiv.), 1-iodo-3-phenylpropane (**2**) (12.1 µL, 0.075 mmol, 1.5 equiv.), and dioxane (0.5 mL). The vial was capped, taken out of the glovebox, and stirred in a pre-heated heating block at 80 °C for 16 hours. After cooling to room temperature, the vial was transferred into an argon-filled glovebox. It

was spiked with THF-d_8 (0.1 mL), shaken, and transferred to an NMR tube for measurement. The measurements were conducted at room temperature.

Certain reagents were omitted for some of the measurements and reaction time and temperature were also deviated in certain cases as indicated in figure S8.

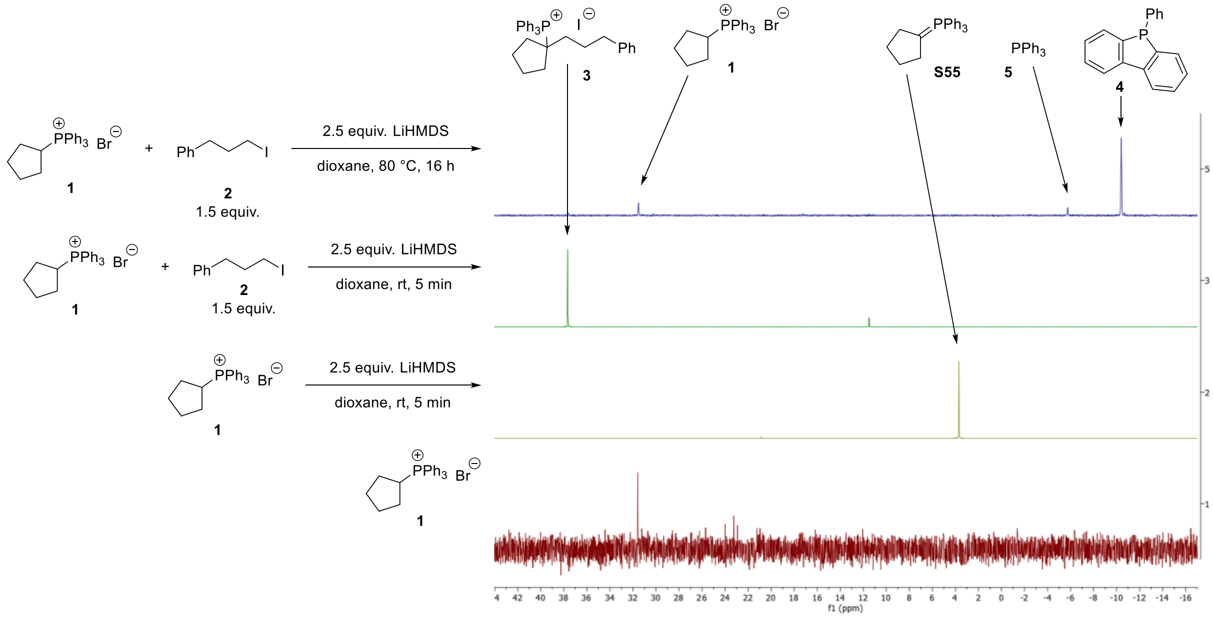

Figure S8. $^{31}P\{^1H\}$ NMR spectra. Top: After heating the reaction for 16 hours. Second from top: Full reaction mixture after stirring at room temperature for 5 min. Third from top: Reaction mixture without alkyl iodide **2** after stirring for 5 min at room temperature. Bottom: phosphonium salt **1**.

The NMR spectra show that the starting phosphonium salt **1** is very quickly consumed even at room temperature in the presence of LiHMDS to give rise to a new species that was tentatively assigned as the corresponding ylide **S55**. In contrast, mixing the phosphonium salt **1**, alkyl iodide **2**, and LiHMDS at room temperature lead to a different signal corresponding to the alkylated phosphonium salt **3**. This indicates that the C–C bond formation is rapid and proceeds by alkylation of the phosphorus ylide. An NMR spectrum of the reaction after heating shows that the phosphonium salts are largely consumed, and the dibenzophosphole **4** is formed as the main phosphorus-containing byproduct of the reaction.

6.5. Reaction of a phosphonium salt containing a tertiary alkyl group

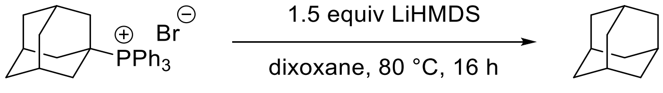

9 **8** 89% GC yield

Inside a glovebox filled with argon, a 4-mL screw-cap vial was subsequently charged with adamant-1-yltriphenylphosphonium bromide (**9**) (47.7 mg, 0.1 mmol, 1.0 equiv.), LiHMDS (25.1 mg, 0.15 mmol, 1.5 equiv.), and dioxane (1.0 mL). The vial was taken out of the glovebox and stirred in a pre-heated heating block at 80 °C for 16 hours. After cooling to room temperature, *n*-dodecane (15 µL) was added as an internal standard, the mixture was shaken, and a sample was taken for GC analysis. Adamantane (**8**) was detected in 89% yield.

Note: Only 1.5 equiv. instead of 2.5 equiv. of LiHMDS were used in reactions of phosphonium salt **9** as 1 equiv. of base is used in the alkylation step of the standard reaction (general procedure GP1). As salt **9** already contains a tertiary alkyl group, the amount of base was reduced for reactions using **9**.

As the adamantylphosphonium salt **9** contains a well-defined tertiary alkyl group and provided the C–P cleaved product under the same conditions as the standard reaction, we used it as a model substrate for the C–P cleavage part of the mechanism.

6.6. Reaction inhibition by TEMPO

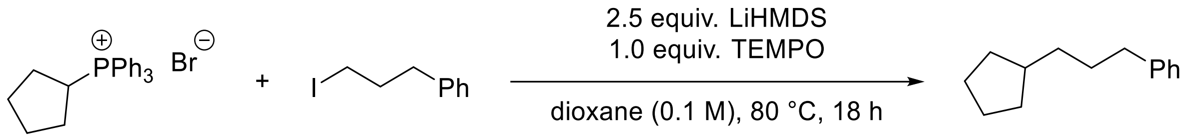

1 1.0 equiv. **2** 1.5 equiv. **6** < 5%

Inside a glovebox filled with argon, a 4-mL screw-cap vial was subsequently charged with cyclopentyltriphenylphosphonium bromide (**1**) (41.1 mg, 0.1 mmol, 1.0 equiv.), LiHMDS (41.8 mg, 0.25 mmol, 2.5 equiv.), 1-iodo-3-phenylpropane (**2**) (24.1 µL, 0.15 mmol, 1.5 equiv.), TEMPO (15.6 mg, 0.1 mmol, 1.0 equiv.), and dioxane (1.0 mL). The vial was taken out of the glovebox and stirred in a pre-heated heating block at 80 °C for 16 hours. After cooling to room temperature, *n*-dodecane (15 µL) was added as an internal standard, the mixture was shaken, and a sample was taken for GC analysis. Only traces of the coupling product **6** were detected.

Inhibition by TEMPO might indicate that the reaction is proceeding through a radical pathway.

6.7. Isolation of TEMPO adduct 7

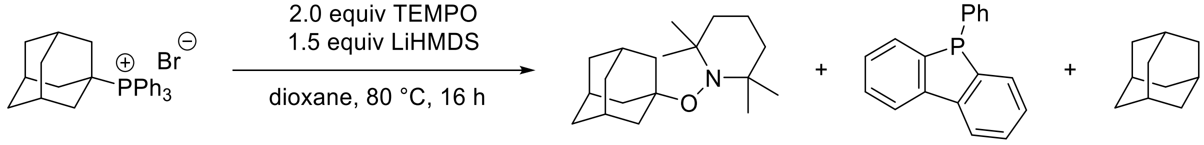

9 **7** 62% isolated **4** 81% NMR yield **8** not observed

Inside a glovebox filled with argon, an 8-mL screw-cap vial was subsequently charged with adamant-1-yltriphenylphosphonium bromide (**9**) (143.2 mg, 0.3 mmol, 1.0 equiv.), LiHMDS (75.3 mg, 0.45 mmol, 1.5 equiv.), TEMPO (93.7 mg, 0.6 mmol, 2.0 equiv.), and dioxane (3.0 mL). The vial was taken out of the glovebox and stirred in a pre-heated heating block at 80 °C for 16 hours. After cooling to room temperature, the reaction mixture was loaded on silica and purified by column chromatography with $AgNO_3$-impregnated silica (cyclohexane) to yield TEMPO adduct **7** as a white solid (54.0 mg, 62%).

Note: $AgNO_3$-impregnated silica was used in this separation as **4** and **7** have similar polarity and the modified silica strongly binds **4**, leading to an easier separation.

^{1}H NMR (400 MHz, $CDCl_3$) δ = 2.12 (br, 3H), 1.90 (d *J* = 3.2 Hz, 6H), 1.60 – 1.57 (m, 6H), 1.56 – 1.41 (m, 5H), 1.30 – 1.27 (m, 1H), 1.18 (s, 6H), 1.08 (s, 6H) ppm.

$^{13}C\{^1H\}$ NMR (101 MHz, $CDCl_3$) δ = 76.6, 59.2, 42.7, 41.2, 36.8, 35.6, 31.6, 20.8, 17.4 ppm.

The spectroscopic data matched those reported in the literature.[28]

The yield of dibenzophosphole **4** was determined to be 81% from a parallel reaction by $^{31}P\{^1H\}$ NMR using triphenyl phosphate as an internal standard. Adamantane (**8**) was not observed by GC/MS in this reaction.

Isolation of the TEMPO adduct **8** in good yield and the lack of formation of adamantane (**8**) in this reaction suggest that the reaction proceeds through a tertiary alkyl radical.

6.8. Deuteration experiments

6.8.1. Reaction using a deuterated substrate

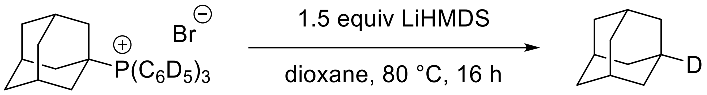

9-*d*₁₅

8-*d*
49% (91% purity, 91% GC yield)
72% D

Inside a glovebox filled with argon, an 8-mL screw-cap vial was subsequently charged with adamantan-1-yltris(phenyl-d_5)phosphonium bromide (**9-*d*$_{15}$**) (246.3 mg, 0.5 mmol, 1.0 equiv.), LiHMDS (125.5 mg, 0.75 mmol, 2.5 equiv.), and dioxane (5.0 mL). The vial was taken out of the glovebox and stirred in a pre-heated heating block at 80 °C for 16 hours. After cooling to room temperature, the reaction mixture was loaded on silica and filtered through a plug filled with $AgNO_3$-impregnated silica (*n*-pentane) to yield adamantane-*d* (**8-*d***) as a white solid (33.2 mg, 49%, 91% purity (remaining *n*-pentane). ^1H qNMR analysis indicated that 72% deuteration had occurred at one of the tertiary carbons. The GC yield was determined to be 91% from a parallel reaction at 0.1 mmol scale using *n*-dodecane as internal standard.

^1H NMR (400 MHz, $CDCl_3$) δ = 1.88 (br, 3.28H), 1.78 – 1.73 (m, 12H) ppm.

$^{13}C\{^1H\}$ NMR (101 MHz, $CDCl_3$) δ = 37.9, 37.8, 28.5, 27.8 (t, *J* = 20.1 Hz) ppm.

The spectroscopic data matched those reported in the literature.[29]

The deuterium incorporation at the tertiary alkyl position indicates that a reactive *tert*-alkyl species abstracts a deuterium from the strong phenyl C–D bond of the phosphonium salt. This *tert*-alkyl species could be a *tert*-alkyl radical (see section 6.6 and 6.7 for radical trapping experiments that invoke such a species). As the *tert*-alkyl radical is less reactive than the aryl radical that would be formed by HAT from the aryl C–D bond, the aryl group would require prior activation to lower the strength of the C–D bond (see Fig. 2e in the manuscript for a proposed pathway that accounts for these considerations).

6.8.2. Reaction using deuterated solvent

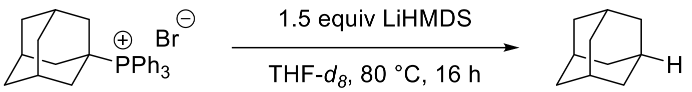

9

8
28% (98% GC yield)
0% D

Inside a glovebox filled with argon, an 8-mL screw-cap vial was subsequently charged with adamant-1-yltriphenylphosphonium bromide (**9**) (238.7 mg, 0.5 mmol, 1.0 equiv.), LiHMDS (125.5 mg, 0.75 mmol, 2.5 equiv.), and THF-d_8 (5.0 mL). The vial was taken out of the glovebox and stirred in a pre-heated heating block at 80 °C for 16 hours. After cooling to room temperature, the reaction mixture was loaded on silica and filtered through a plug filled with $AgNO_3$-impregnated silica (*n*-pentane) to yield adamantane (**8**) as a white solid (37.6 mg, 28%). ^{1}H qNMR analysis indicated that no deuterium had occurred. The GC yield was determined to be 98% from a parallel reaction at 0.1 mmol scale using *n*-dodecane as internal standard.

^{1}H NMR (400 MHz, $CDCl_3$) δ = 1.88 (br, 4H), 1.76 (t, *J* = 3.4 Hz, 12H) ppm.

The spectroscopic data matched those reported in the literature.[30]

6.9. Investigation of ion effects

Inside a glovebox filled with argon, a 4-mL screw-cap vial was subsequently charged with cyclopentyltriphenylphosphonium bromide (**1**) (41.1 mg, 0.1 mmol, 1.0 equiv.), base (0.25 mmol, 2.5 equiv.), an additive (0.25 mmol, 2.5 equiv.), 1-iodo-3-phenylpropane (**2**) (24.1 µL, 0.15 mmol, 1.5 equiv.), and dioxane (1.0 mL). The vial was capped, taken out of the glovebox, and stirred in a pre-heated heating block at 80 °C for 16 hours. After cooling to room temperature, *n*-dodecane (15 µL) was added as an internal standard, the mixture was shaken, and a sample was taken for GC analysis.

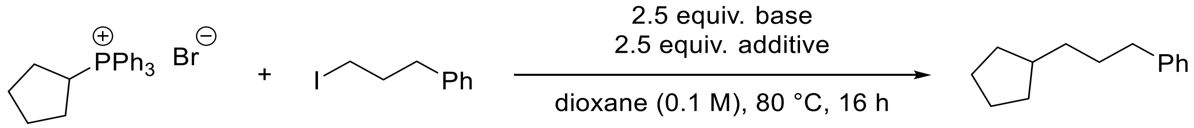

1 **2** 1.5 equiv. **6**

Table S12. Effect of additives on the reaction yield.

Entry	base	additive	Yield (%)[a]
1	LiHMDS	-	96
2	KHMDS	-	18
3	NaHMDS	-	43
4	LiHMDS	KCl	quant.
5	LiHMDS	12-crown-4	0
6	LiHMDS	12-crown-4 (1.0 equiv.)	13
7	LiHMDS	18-crown-6	75

a: Yield determined by GC using *n*-dodecane as an internal standard.

The reaction only gives high yield when LiHMDS is used (entry 1) while congeners containing other alkali cations (entries 2-3) gave significantly lowered yield. The addition of an alkali halide additive to the reaction using LiHMDS also did not change the yield much (entry 4). In contrast, addition of 12-crown-4, which selectively binds lithium cations, shuts down the reaction (entries 5-6). As a control reaction, 18-crown-6, which selectively binds potassium cations, only lowered the yield a bit (entry 11). These results suggest that lithium bound to HMDS is important for the reactivity. We hypothesize that lithium cations are important for the facilitation of a metathesis step between the phosphonium salt after alkylation (containing the *tert*-alkyl group) and LiHMDS. The formation of LiX (X =Br or I) might be a driving force for this step that leads to the formation a hindered *tert*-alkylphosphonium HMDS ion pair.

6.10. UV/Vis spectroscopy

Inside a glovebox filled with argon, a 4-mL screw-cap vial was charged with adamantyltriphenylphosphonium bromide (**9**) (7.2 mg, 0.015 mmol, 1.0 equiv.) and LiHMDS (2.5 mg, 0.015 mmol, 1.0 equiv.). Dioxane (1 mL) was added, and the vial was shaken for 30 seconds. The supernatant was removed by syringe filtration, and a UV/Vis spectrum of the filtrate was taken in the glovebox.

Note 1: Samples of the two starting materials were taken using the same concentration. Phosphonium salt **9** was measured in *N,N*-dimethylformamide (DMF) because of the poor solubility in dioxane.

Note 2: A background spectrum of the pure solvent (dioxane or DMF, respectively) was subtracted from the samples containing the analytes.

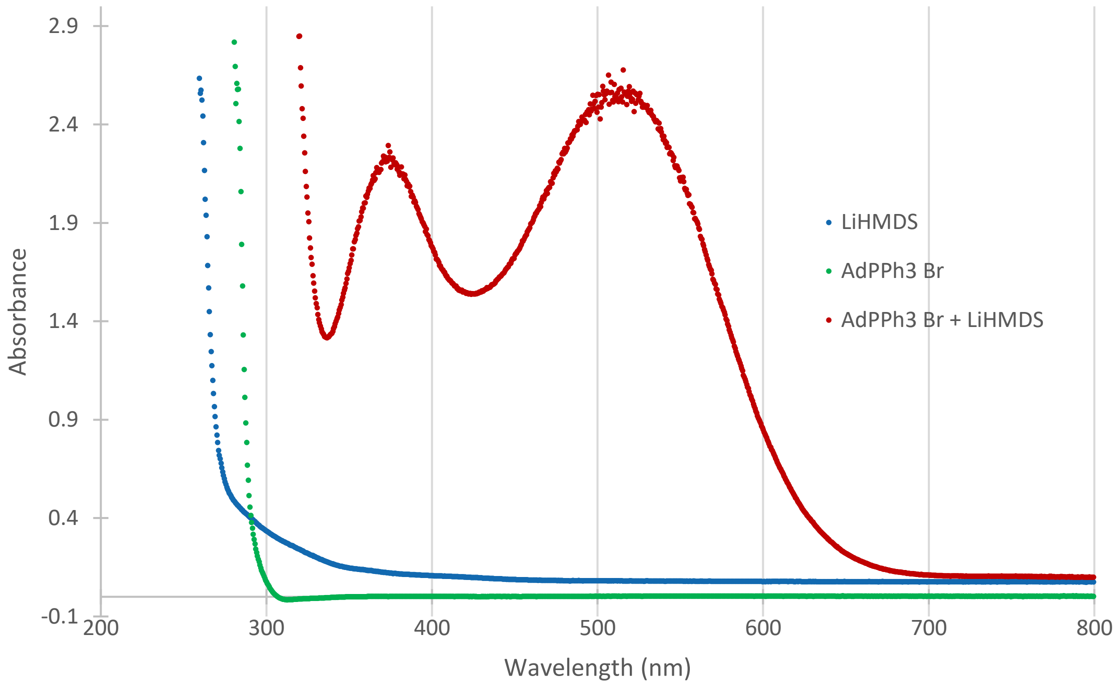

Figure S9. UV/Vis spectra of **9** (AdPPh3 Br), LiHMDS, and the mixture of the two compounds.

The UV/Vis spectrum shows the appearance of two charge-transfer bands when the colorless starting materials **9** and LiHMDS are mixed.

7. Computational studies

7.1. Computational details

Conformer searches for ground state structures were conducted with CREST (version 2.10.2) using default settings.[31] Additional conformers were added manually if necessary. Conformer searches for transition state structures were conducted manually based on the conformer ensembles of the corresponding ground state structures.
DFT-level optimization, frequency, and population analysis calculations of the generated conformer ensembles were conducted using the Gaussian09 suite of programs (Revision D.01).[32] The keyword integral(grid=ultrafine) was used in all calculations to limit grid-based errors. Optimizations were conducted at the PBE0 level of theory[33–36] including Grimme's dispersion correction (D3) with Becke-Johnson damping[37,38] and the SMD solvent model for 1,4-dioxane.[39] Initial optimizations were carried out with the def2svp basis set and then further refined with calculations using the def2tzvp basis set.[40,41] Frequency calculations were carried out with the optimized geometries using the same level of theory, the def2tzvp basis set, and thermal correction to 353 K. Ground states were identified by having zero imaginary frequencies in frequency calculations, whereas all reported transition states have exactly one imaginary frequency with its vibrational mode corresponding to the appropriate reactivity. Quasi-harmonic vibrational corrections were applied using the method proposed by Grimme[42] (cut-off value of 100 cm^{-1}) as implemented in the Goodvibes program.[43]
Bond dissociation enthalpy were calculated in the gas phase at the PBE0-D3(BJ)/def2tzvp level of theory including thermal correction to 298 K.

All other calculations were carried out using ORCA Version 5.0.3.[44–46] Single point energies were calculated at the PW6B95 level of theory[47] including Grimme's dispersion correction (D3) with Becke-Johnson damping[37,38] and the SMD solvent model for 1,4-dioxane.[39] The default RIJCOSX approximation was applied and the def2-qzvpp basis set[40,41] as well as the def2/J auxiliary basis sets were used. Tight convergence criteria (TightSCF keyword) and the default grid (defgrid2) were used. Orbital data was obtained at the same level of theory and visualized using Multiwfn (Version 3.8).[48] NCI plots were also generated from calculations at the same level. The NCI isosurface was generated with Multiwfn (Version 3.8) and visualized with VMD (version 1.9.3).[49] UV/Vis spectra were simulated by TD-DFT using the CAM-B3LYP functional,[50] the def2-qzvpp basis set,[40,41] the def2/J auxiliary basis set, and the SMD solvent model for 1,4-dioxane.[39] The first 10 excitations were calculated (nroots = 10).

Thermochemical corrections were calculated by subtracting the electronic energy of the optimization calculations from the quasi-harmonic free energies obtained using the GoodVibes program. Gibbs free energies were obtained by adding this thermochemical correction to the ORCA single point energy at the PW6B95 level of theory (see paragraph above).

Buried volume calculations were carried out with SambVca 2.1 using standard settings and the structures optimized according to the conditions above (Bondii radii scaled by 1.17, 3.5 sphere radius, H atoms not included).[51] The values were calculated for the amide anions, putting the nitrogen atom at the center for the sphere.

Geometries of structures were visualized with CYLview20.[52] Energy diagrams were created using EveRplot (version 1.1).[53]

7.2. Discussion of the free energy diagram of the reaction

7.2.1. Free energy diagram of the reaction

Anionic and open-shell intermediates are more challenging to model by DFT than closed-shell, neutral species. The proposed reaction mechanism contains such species as well as closed-shell intermediates which could potentially also lead to faulty error cancellation due to the different natures of the species in addition to the above mentioned challenge. We calculated single point energies using a selection of functionals (Table S13) to see if the different methods give similar results. The results show that the barrier heights vary significantly between the different methods. The provided energy diagram should thus only be regarded as a qualitative description of the relative barriers of the reaction rather than a quantitative one.

Table S13. Calculated Gibbs free energies using different functionals in the single point calculation.

	wB97X-V	PBE0-D3(BJ)[a]	PW6B95-D3(BJ)	M06-2X-D3(0)
pre-**B** (solvent-separated ions)	0.0	0.0	0.0	0.0
**B** (contact ion pair)	-18.4	-18.9	-19.1	-19.9
**C**	19.2	11.0	12.5	17.1
**TS1**	26.2	27.5	21.2	24.0
**5**	-4.0	-6.4	-6.8	-3.8
**TS2**	20.4	14.0	16.2	18.8
**E**	0.6	-1.9	-2.0	-2.5
**TS3**	9.4	5.3	5.3	9.0
**F**	-23.1	-25.0	-22.4	-19.7
**4** + **G**	-95.8	-95.6	-97.1	-96.3

a: Electronic energy evaluated at the same conditions as the frequency calculation.

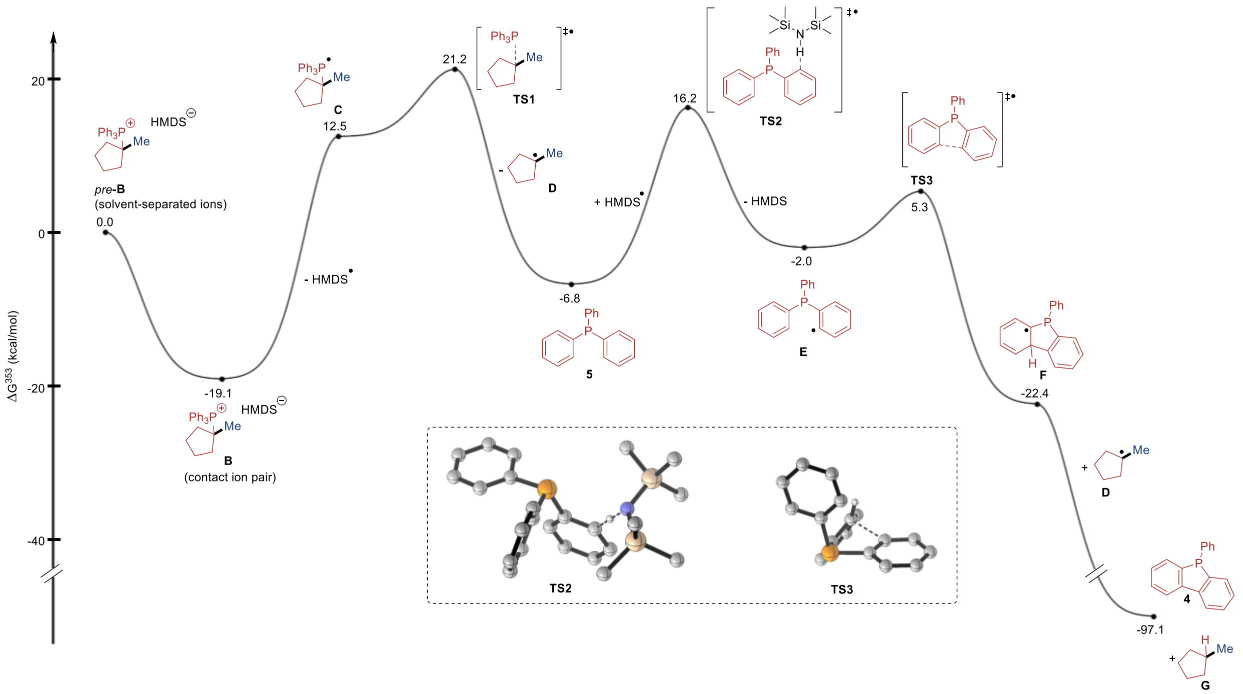

Figure S10. Calculated free energy diagram at the PW6B95-D3(BJ)/SMD(dioxane)-def2qzvpp//PBE0-D3(BJ)/ SMD(dioxane)-def2tzvp level of theory. The optimized geometries of the transition states **TS2** and **TS3** are shown. Most hydrogen atoms have been omitted for clarity.

7.2.2. Discussion of the SET step in the frustrated ion pair **B**

We were able to locate two types of geometries for the ion pair **B** between the phosphonium cation and the HMDS anion (Figure S11A). In structure **B**, the HMDS nitrogen interacts with the phenyl groups of the phosphonium cation in a C–H-anion and a pi-anion interaction. The interactions are also visible in non-covalent interaction plots (Figure S11B). In contrast, structure **B’** features a direct interaction between the phosphonium phosphorus atom and the HMDS nitrogen atom that resembles the structure of a phosphorane. This structure **B’** is 21.7 kcal/mol less favorable than **B**, likely because of the strong steric repulsion that is visible between the ions in the NCI plot (Figure S11C).

A **Structures and relative energies of phosphonium HMDS ion pairs**

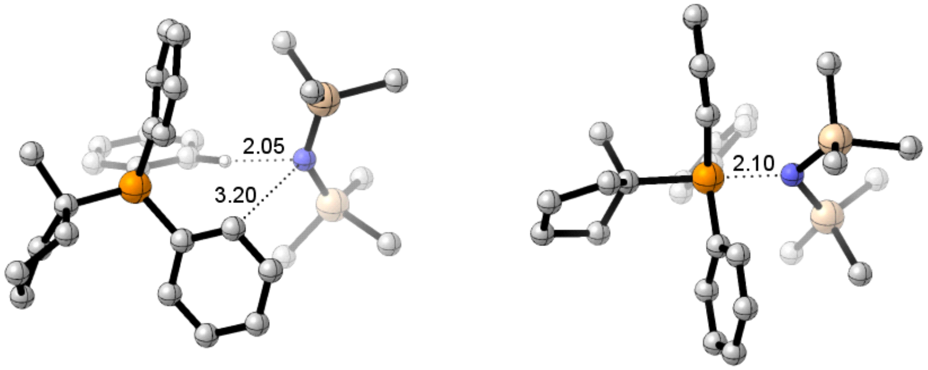

**B**	**B'**
0.0 kcal/mol	21.7 kcal/mol

B **NCI plot of B**

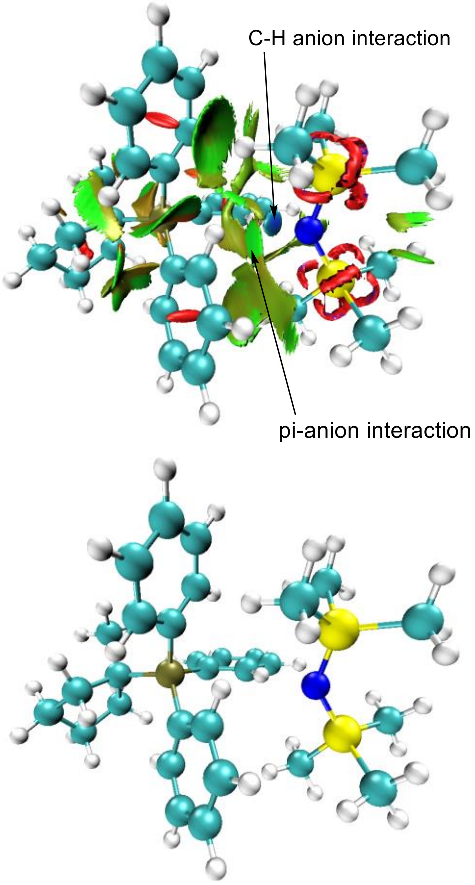

C **NCI plot of B'**

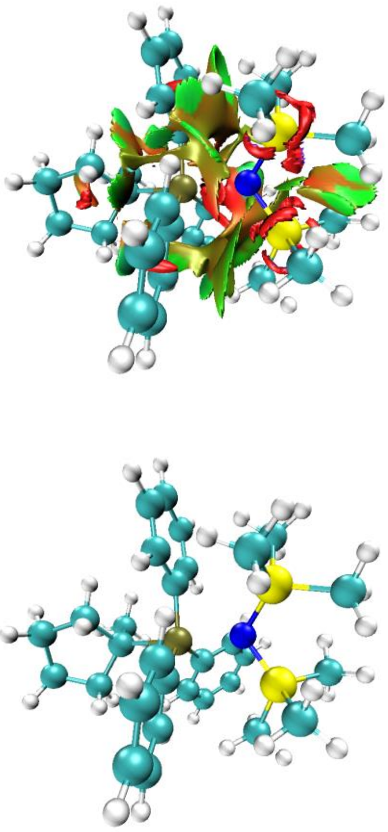

Figure S11. Comparison of phosphonium HMDS ion pair isomers (most hydrogens omitted for clarity). (**A**) Molecular structure and relative energies (in kcal/mol). (**B**) NCI plot of **B** (top). For easier understanding, the structure is shown in the same view below without the NCI isosurface below. (**C**) NCI plot of **B’** (top). For easier understanding, the structure is shown in the same view below without the NCI isosurface. The standard color scale for NCI plots was used (blue – strong interaction, green – van der Waals interactions, yellow to red: repulsion including steric effects in rings)

Frontier orbital analysis of both structures show that the HOMO is located on the HMDS ion, whereas the LUMO is located on the phosphonium part of the ion pair (Figure S12). Thus, SET would lead to the proposed phosphoranyl radical and an HMDS radical.

A Frontier orbitals of B.

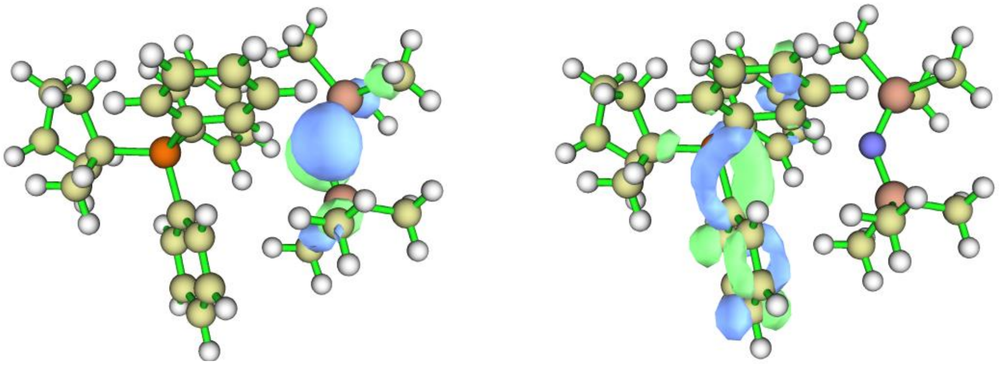

HOMO LUMO

B Frontier orbitals of B'.

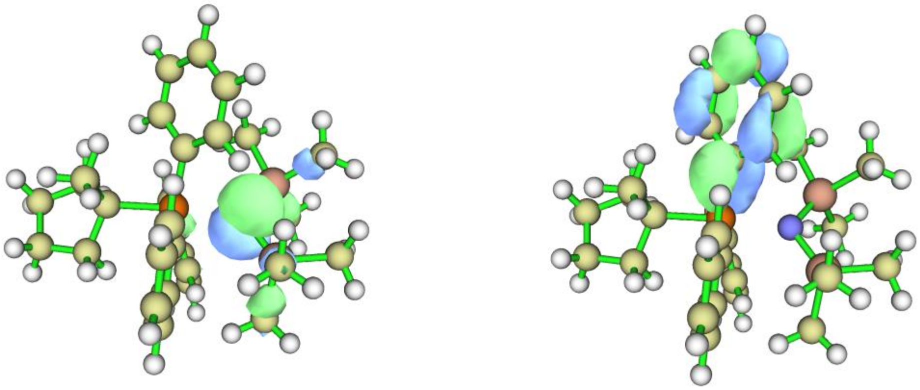

HOMO LUMO

Figure S12. Frontier orbitals of phosphonium HMDS ion pair isomers. (**A**) HOMO and LUMO of **B**. (**B**) HOMO and LUMO of **B’**.

To evaluate the thermodynamic feasibility of the single-electron transfer from the HMDS anion to the phosphonium cation, we calculated the electronic energy for the single-electron oxidation (equivalent to the ionization potential) of the HMDS anion and the single-electron reduction (equivalent to the electron affinity) of the phosphonium cation (Table S14). The results show that the energy difference is thermodynamically accessible at the reaction temperature of 80 °C despite a significant dependence on the employed functional (0.73–1.04 eV; corresponding to 16.8–24.0 kcal/mol).

Table S14. Calculated electronic energies for the single electron transfer step. Energies given in eV.

	**wB97X-V**	**PW6B95-D3(BJ)**	**M06-2X-D3(0)**
1e- reduction of phosphonium cation	-2.43	-2.73	-2.69
1e- oxidation of HMDS anion	3.47	3.46	3.62
Sum	1.04	0.73	0.93

Notably, it is known that the proposed SET step can occur photochemically using phosphonium halide salts.[54–57]. For comparison, we also calculated the difference in electronic energy for the same

phosphonium cation and halide anions given values of 1.83 eV (42.2 kcal/mol) for the iodide salt and 1.93 eV (44.5 kcal/mol) for the bromide salt, showing that the SET for these salts requires photoexcitation in contrast to the case of the HMDS salt.

The structure responsible for the SET step should exhibit notable charge-transfer character. To investigate which structure might be enabling the SET step, we calculated the UV/Vis spectra of **B** and **B'** by TD-DFT. For **B**, the first excitation was calculated to occur at 348 nm. The value was 272 nm for **B'**, which indicates that no significant charge-transfer character is expected for this isomer. The calculated value for **B** is similar to the experimentally determined charge-transfer band for the combination of adamantyltriphenylphosphonium bromide (**9**) and LiHMDS (charge-transfer bands at ca. 380 nm and 515 nm). Deviations might arise from the slightly different alkyl group in the phosphonium salts **B** and **9**.
The results of the UV/Vis calculations thus indicate that it is more likely that **B** rather than **B'** is the active species involved in the SET step.

7.2.3. Discussion of the regioselectivity of the HAT step

In HAT step (compound **5** to structure **E** via **TS2**), the HMDS radical abstracts a hydrogen atom from the *ortho*-position of the phenyl group in triphenylphosphine (**5**). To understand the regioselectivity of this step, we calculated the bond dissociation enthalpies (BDEs) of the different C–H bonds in **5** (Figure S13).

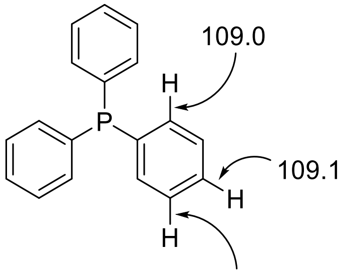

Figure S13. Calculated C–H bond dissociation enthalpies in triphenylphosphine (**5**).

The calculations show that the BDEs are not significantly different. One possible explanation for the observed regioselectivity is that the HAT step is reversible and endergonic (see Figure S10) and that the intermediates formed after activation of the *meta*- and *para*-positions cannot undergo further reaction, whereas the *ortho*-radical is consumed via the cyclization to intermediate **E**. Additionally, the most favorable structure of the frustrated ion pair **B** already contains a C–H-anion interaction between the HMDS anion and the *ortho* C–H bond of a phenyl group of the phosphonium moiety. It is conceivable that this preorientation leads to preferential *ortho* activation of the HMDS radical after the SET step.
Additionally, the alkyl substituent and HMDS anion are situated on opposite ends of the ion pair to minimize steric interactions which might prevent undesired HAT with an alkyl C–H bond.
A potential mechanistic alternative to the proposed HAT step is that the HMDS radical oxidizes triphenylphosphine (**5**) to the corresponding radical cation **E'** which can then cyclize to **E''** (Figure S14). A subsequent deprotonation would lead again to the cyclic radical **F** as in the main mechanistic proposal. The initial electron transfer step is however thermodynamically very unfavorable which makes such a mechanism unlikely.

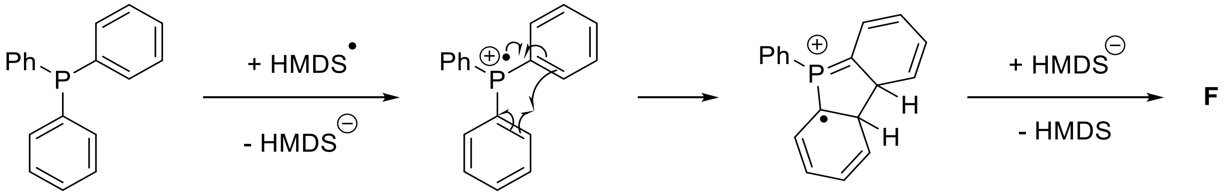

Figure S14. Potential mechanistic alternative to the HAT step.

7.2.4. Discussion of the cyclization step

The lowest barrier transition state **TS3** for the cyclization of the triphenylphosphine-derived radical **E** yields a conformer of the cyclized radical **F** in which the phenyl group is on the same face of the molecule as the weak C–H bond, making the approach of the tertiary alkyl radical **D** for the subsequent HAT step sterically unfavorable (Figure S15). An alternative transition state **TS3’**, that is only 0.5 kcal/mol higher in energy than **TS3**, yields the isomer **F’** in which the C–H bond is well accessible by the tertiary alkyl radical. While HAT from **F** might be possible, we expect that it should be more likely to occur from **F’**. The energy required for the isomerization of **F** to **F’** by inversion at the phosphorus center is 24.4 kcal/mol (**TS4**), allowing access to the more reactive isomer.

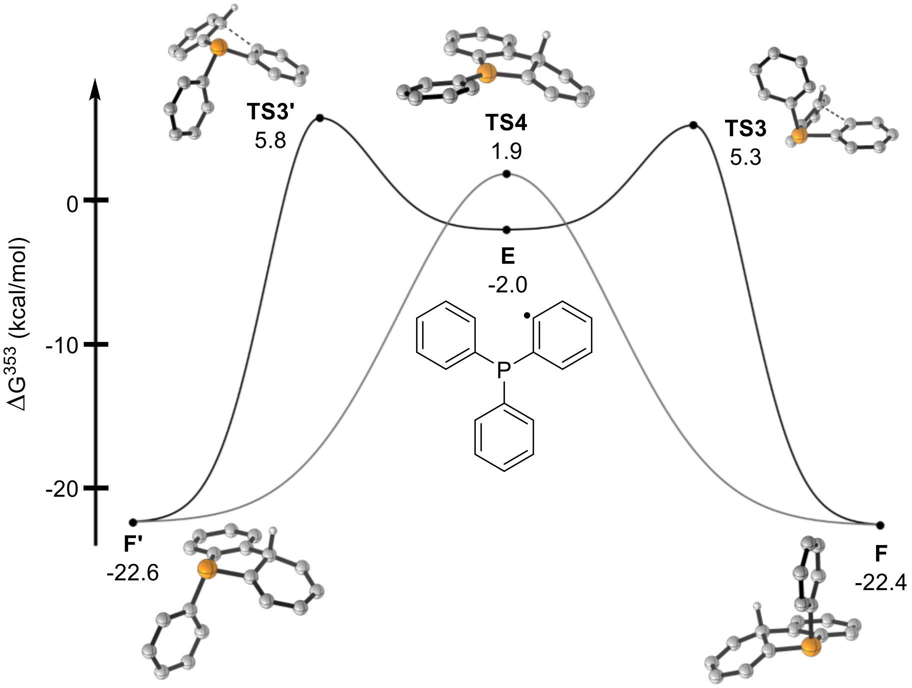

Figure S15. Energetics of the cyclization step and interconversion of the cyclized products. Most hydrogens were omitted in the 3D structures for clarity.

7.3. Effect of the base

Experimentally, strong dependence of the reaction outcome on the size of the amide base was observed. While HMDS bases performed best, smaller amide bases like lithium diisopropylamide or lithium dimethylamide gave significantly lowered yield. To investigate the origin of this effect, we also performed calculations using these two amide bases.
First, we investigated the structure of the phosphonium amide ion pairs (Figure S16).

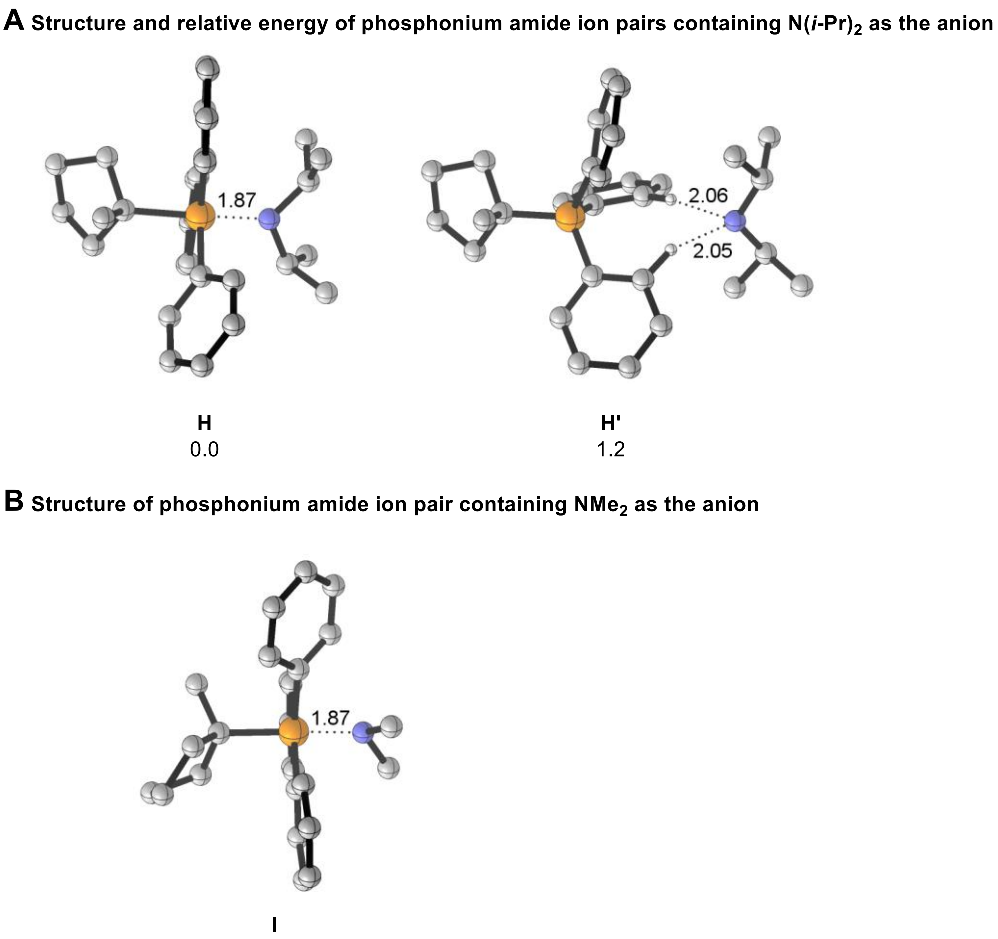

Figure S16. Structures of phosphonium amide ion pairs. (**A**) Molecular structure and relative energies (in kcal/mol) for ion pairs containing the N(*i*-Pr)$_2$ anion. (**B**) Molecular structure for the ion pair containg the NMe$_2$ anion.

While the formation of a direct interaction between the phosphorus of the phosphonium cation and the amide nitrogen, reminiscent of a phosphorane, is not preferred in the case of HMDS (structure **B'**, see also section 7.2.2), it is preferred in the case of diisopropylamide (structure **H**) and dimethylamide (structure **I**). Notably, the P–N distance is significantly shorter in the case of the smaller anions (1.87 Å, respectively) than in the case of HMDS as the anion (2.10 Å). For the case of diisopropylamide, we were able to also locate an ion pair containing weak interactions between the phosphonium phenyl groups and the amide (**H'**). It is 1.2 kcal/mol higher in energy than **H**, whereas the related structure **B**, containing the HMDS anion, was 21.7 kcal/mol preferred to the corresponding P–N interaction isomer **B'**. Distortion-interaction analysis of the ion pairs was performed for the ion pair formation from the free ions to further understand the bonding in these structures (Table S15).

Table S15. Distortion-interaction analysis of different ion pairs. All values in kcal/mol.

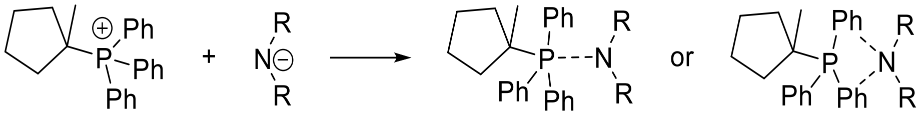

Structure	Anion	Type of interaction	Distortion	Interaction	Distorsion + Interaction
**B**	HMDS	anion-aryl	1.8	-38.4	-36.6
**B'**	HMDS	P–N	55.1	-75.7	-20.6
**H**	N(*i*-Pr)$_2$	P–N	59.2	-107.3	-48.1
**H'**	N(*i*-Pr)$_2$	anion-aryl	3.2	-40.5	-37.3
**I**	NMe$_2$	P–N	45.4	-103.1	-57.8

The structure containing a close P–N interaction (**B'**, **H**, **I**) have a high distortion energy that is balanced by a strong interaction energy. Notably, the interaction energies of the smaller amides (**H**, **I**) are similar but significantly higher than the one in the case of the HMDS salt (**B'**). The difference in interaction energy is also reflected in the longer P–N distance in **B'** (2.10 Å) compared to **H** and **I** (1.87 Å, respectively) and likely originates from the large size of the HMDS anion preventing closer interaction.

The structures containing the anion-aryl interactions (**B** and **H'**) only have a smaller distortion energy, but also a much weaker interaction energy than the P–N-type isomers. For the HMDS ion pair, the anion-aryl isomer **B** with lower interaction energy is preferred to the P–N-type **B'** because the large steric bulk of the HMDS anion prevents the formation of a sufficiently large interaction in the P–N isomer **B'**.

In the case of the smaller amide anions, the steric size of the anions is small enough to allow an increased interaction energy in the P–N-type isomers so that this structure is preferred.

Dimethylamide and diisopropylamide thus prefer to form phosphorane-like P–N-type structures with phosphonium cations instead of forming interactions with phosphonium phenyl groups as in the case of HMDS. On the basis of the above analysis of the charge-transfer character of the different ion pair geometries (section 7.2.2), we propose that this may result in a lowered ability to perform the SET to the phosphonium cation.

The identity of the amide also has a strong effect on the HAT step of the reaction. The calculated energy barriers for this step with the dimethylamide radical (**TS5**) and the diisopropylamide radical (**TS6**) are almost identical and significantly higher than the barrier using the HMDS radical (Figure S17A).

A **Energetics of HAT step using different aminyl radicals**

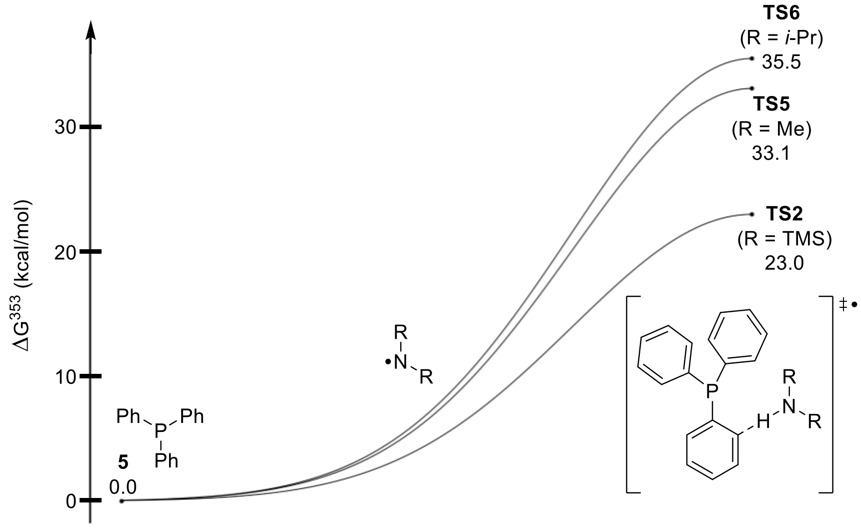

B **Calculated properties of different amines**

amine	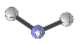 NHMe$_2$	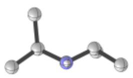 NH(*i*-Pr)$_2$	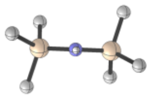 NH(TMS)$_2$
Dihedral angle RRNH	122.2°	124.5°	178°
N–H BDE (kcal/mol)	88.9	89.5	105.1

Explanation of dihedral angle:

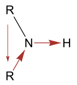

Figure S17. Investigation of the HAT step using different aminyl radicals. (**A**) Energetics of the HAT step. (**B**) Properties of the corresponding amines.

Analysis of the N–H BDEs of the corresponding amines shows that the bond is significantly stronger in the case of HMDS than in the case of the two smaller amines (Figure S17B). The higher BDE correlates with a higher reactivity of the radical. While the geometries of the two smaller amides show the typical trigonal pyramidal arrangements of amines, HMDS features a nearly planar nitrogen (Figure S17B) that is likely enforced by the large silyl substituents. This highlights that the large size of HMDS not only enables the formation of an ion pair interaction suitable for SET from the amide anion to the phosphonium cation, but also enables the subsequent HAT step by generating a more reactive aminyl radical after SET.

7.4. Energies of calculated structures

Table S16. Summary of calculated energy values. E: electronic energy of the optimization (using PBE0-D3(BJ) in Gaussian). ZPE: zero-point energy correction. H: enthalpy of the optimization calculation. T.S: entropy term. T.qh-S: entropy term with quasi-harmonic energy correction. qh-G(T): free energy with quasi-harmonic correction of the optimization reaction, calculated as [qh-G(T)] = [H] – [T.qh-S]. E_SPC: electronic energy of the single point calculation (using PW6B95-D3(BJ) in ORCA). qh-G(T)_SPC: free energy with quasi-harmonic correction, calculated as [qh-G(T)_SPC] = [qh-G(T)] – [E] + [E_SPC]. All values in Hartree.

Structure	E	ZPE	H	T.S	T.qh-S	qh-G(T)	E_SPC	qh-G(T)_SPC
(1-MeCyp)PPh_3^+	-1270.574530	0.437675	-1270.104764	0.099584	0.093487	-1270.198252	-1273.397914	-1273.021636
Br^-	-	-	-	-	-	-	-2575.468083	-
Br-radical	-	-	-	-	-	-	-2575.296853	-
$HMDS^-$	-872.945606	0.224954	-872.697994	0.076076	0.073730	-872.771724	-874.487038	-874.313156
HMDS	-873.501342	0.238195	-873.239942	0.076842	0.074882	-873.314824	-875.042133	-874.855615
HMDS-radical	-872.822829	0.225495	-872.574138	0.079181	0.076428	-872.650566	-874.360017	-874.187754
HN(*i*-Pr)$_2$	-292.187494	0.205526	-291.968231	0.055324	0.054660	-292.022891	-292.932590	-292.767987
$HNMe_2$	-135.052669	0.092478	-134.953351	0.038104	0.038107	-134.991458	-135.393980	-135.332769
H-radical	-	-	-	-	-	-	-0.500906	-
I^-	-	-	-	-	-	-	-298.033698	-
I-radical	-	-	-	-	-	-	-297.866231	-
N(*i*-Pr)$_2^-$	-291.590793	0.188138	-291.388940	0.055591	0.054676	-291.443616	-292.339162	-292.191985
N(*i*-Pr)$_2$-radical	-291.532129	0.191275	-291.326948	0.057187	0.055937	-291.382886	-292.273421	-292.124178
NMe_2^-	-134.454557	0.074453	-134.373542	0.037420	0.037424	-134.410966	-134.798073	-134.754482
NMe_2-radical	-134.397317	0.077477	-134.312825	0.039518	0.039436	-134.352261	-134.734601	-134.689545
**4**[a]	-1034.464655	0.253579	-1034.189842	0.076629	0.072715	-1034.262557	-1036.682536	-1036.480438
**5**	-1035.644590	0.274775	-1035.346865	0.081787	0.076269	-1035.423133	-1037.869051	-1037.647594
**B**	-2143.579283	0.663505	-2142.859957	0.151442	0.140168	-2143.000125	-2147.944397	-2147.365239
**B'**	-2143.551218	0.667670	-2142.829501	0.143326	0.135039	-2142.964539	-2147.917357	-2147.330678
**C**	-1270.672810	0.432806	-1270.207524	0.099104	0.094285	-1270.301810	-1273.498158	-1273.127158
**D**	-235.022485	0.154099	-234.858156	0.048567	0.048263	-234.906419	-235.626313	-235.510247
**E**	-1034.959027	0.261641	-1034.674415	0.083259	0.077380	-1034.751796	-1037.179323	-1036.972092
**E-meta_radical**	-1034.959495	0.261712	-1034.674906	0.082475	0.076955	-1034.751861	-1037.179489	-1036.971855
**E-para_radical**	-1034.958625	0.261729	-1034.674035	0.082209	0.076816	-1034.750851	-1037.178405	-1036.970631
**F**	-1034.998511	0.262343	-1034.714311	0.078228	0.074310	-1034.788621	-1037.214464	-1037.004574
**F'**	-1034.999121	0.262260	-1034.714980	0.077989	0.074220	-1034.789200	-1037.214797	-1037.004876
**G**	-235.677002	0.168786	-235.498254	0.047541	0.046752	-235.545006	-236.285483	-236.153487
**H**	-1562.239245	0.633060	-1561.561370	0.120930	0.116196	-1561.677566	-1565.807078	-1565.245399
**H'**	-1562.228410	0.627366	-1561.554586	0.129957	0.121298	-1561.675884	-1565.795988	-1565.243462
**I**	-1405.125619	0.519242	-1404.568619	0.107705	0.103221	-1404.671839	-1408.287782	-1407.834002
**TS1**	-1270.643831	0.431408	-1270.179831	0.103175	0.095797	-1270.275628	-1273.481432	-1273.113229
**TS2**	-1908.459826	0.495715	-1907.917689	0.133577	0.123505	-1908.041194	-1912.217301	-1911.798669
**TS3**	-1034.948727	0.260869	-1034.666027	0.077961	0.074231	-1034.740259	-1037.168878	-1036.960410
**TS3'**	-1034.948569	0.260939	-1034.665830	0.077292	0.073962	-1034.739792	-1037.168471	-1036.959694
**TS4**	-1034.959136	0.261269	-1034.676558	0.075767	0.073069	-1034.749628	-1037.175444	-1036.965936
**TS5**	-1170.014519	0.349540	-1169.634996	0.098507	0.091238	-1169.726234	-1172.572605	-1172.284320
**TS6**	-1327.148364	0.462514	-1326.648839	0.112855	0.104469	-1326.753307	-1330.110282	-1329.715225

a: The structure showed a small persistent imaginary frequency (-9.2 cm^{-1}). This frequency was inverted in the calculation of the thermochemical correction using the invertifreq functionality of the GoodVibes program.

7.5. Coordinates of calculated structures

(1-MeCyp)PPh_3^+

P	-0.098771	0.022004	0.142670
C	-0.761998	-1.639258	-0.010404
C	-0.896141	-2.165848	-1.296508
C	-1.455838	-3.419066	-1.476903
C	-1.879559	-4.155338	-0.380550
C	-1.745411	-3.636901	0.898974
C	-1.192039	-2.381758	1.088182
H	-1.095937	-1.997940	2.094090
H	-2.071724	-4.212017	1.757212
H	-2.313254	-5.138242	-0.522952
H	-1.556313	-3.821750	-2.477636
H	-0.561501	-1.599152	-2.157378
C	0.703003	0.322599	1.771748
C	1.608838	-0.881622	2.182511
H	1.252471	-1.254468	3.145351
H	1.558023	-1.728452	1.496438
C	3.025181	-0.323330	2.310833
H	3.560323	-0.426922	1.362285
H	3.604553	-0.847254	3.072425
C	2.816246	1.150453	2.617130
H	2.519108	1.285890	3.661323
H	3.705561	1.759027	2.446780
C	1.672527	1.530792	1.685975
H	2.068658	1.656104	0.674796
H	1.172974	2.458597	1.968071
C	-0.364122	0.604459	2.830396
H	0.141855	0.816516	3.775380
H	-1.033821	-0.238928	3.002258
H	-0.974620	1.471737	2.575805
C	-1.438710	1.191616	-0.089787
C	-1.193548	2.564248	0.002579
C	-2.234682	3.461755	-0.148777
C	-3.522820	3.000526	-0.386781
C	-3.769398	1.640000	-0.475049
C	-2.731830	0.732528	-0.326996
H	-2.933305	-0.329535	-0.393587
H	-4.774259	1.278681	-0.657916
H	-4.336436	3.707101	-0.501682
H	-2.040388	4.525358	-0.078704
H	-0.193915	2.935770	0.191420
C	1.100129	0.208182	-1.181672
C	2.160340	-0.698011	-1.255567
C	3.113059	-0.564637	-2.248751
C	3.012236	0.463379	-3.177076
C	1.952020	1.353269	-3.116221
C	0.993879	1.229441	-2.121414
H	0.162894	1.922482	-2.089788
H	1.863863	2.147412	-3.848010
H	3.758973	0.562958	-3.956193
H	3.933203	-1.270572	-2.303548
H	2.236414	-1.517312	-0.551540

Br^-

Br	0.000000	0.000000	0.000000

Br-radical

Br	0.000000	0.000000	0.000000

$HMDS^-$

N	0.000002	0.000133	0.768986
Si	1.525045	-0.001113	0.131374
C	2.835936	-0.487056	1.412620
H	2.672376	-1.511351	1.764878
H	3.855168	-0.428461	1.013379
H	2.780331	0.168016	2.288828
C	1.807279	-1.195045	-1.325425
H	1.175075	-0.928387	-2.179933
H	1.552713	-2.222010	-1.042040
H	2.847083	-1.195424	-1.674713
C	2.104882	1.683760	-0.534903
H	1.445743	2.029375	-1.338974
H	2.074170	2.442284	0.255073
H	3.127290	1.654981	-0.930937
Si	-1.525047	0.001138	0.131384
C	-2.104695	-1.683853	-0.534747
H	-2.074301	-2.442241	0.255373
H	-3.126926	-1.655182	-0.931233
H	-1.445184	-2.029616	-1.338451
C	-2.836000	0.487133	1.412536
H	-3.855195	0.428525	1.013196
H	-2.672471	1.511455	1.764727
H	-2.780526	-0.167855	2.288814
C	-1.807396	1.194924	-1.325500
H	-1.552850	2.221929	-1.042232
H	-2.847237	1.195238	-1.674686
H	-1.175276	0.928254	-2.180067

HMDS

N	0.000748	0.077830	0.792591
H	-0.000966	0.209925	1.794750
Si	-1.579346	0.018744	0.084538
C	-2.774182	0.737783	1.330071
H	-2.529850	1.777992	1.564378
H	-3.800746	0.714605	0.952137
H	-2.761354	0.171075	2.266693
C	-1.609510	1.017491	-1.497694
H	-0.900009	0.634228	-2.236981
H	-1.368813	2.067757	-1.311642
H	-2.602466	0.978141	-1.956719
C	-2.099503	-1.733912	-0.321135
H	-3.112939	-1.760076	-0.734688
H	-1.430522	-2.188952	-1.057514
H	-2.087591	-2.367253	0.571064
Si	1.579962	-0.003139	0.085408
C	2.785642	-0.462317	1.439098
H	3.808103	-0.518893	1.053711
H	2.782175	0.280694	2.242905
H	2.540904	-1.433511	1.878621
C	1.599479	-1.293088	-1.268072
H	2.595109	-1.359628	-1.718348
H	0.899185	-1.054329	-2.073699
H	1.343066	-2.282983	-0.881169
C	2.096794	1.636259	-0.656385
H	1.434994	1.933166	-1.475125
H	3.115373	1.589426	-1.055268
H	2.070167	2.432023	0.094224

HMDS-radical

N	0.000015	0.000160	0.756272
Si	1.599188	-0.010420	0.095421
C	1.922354	-1.780660	-0.433069
H	1.309440	-2.063555	-1.292177
H	2.973972	-1.898423	-0.713481
H	1.713948	-2.480515	0.380288
C	1.748318	1.150208	-1.368164
H	1.099905	0.848629	-2.195165
H	2.777858	1.163884	-1.740610
H	1.489253	2.175785	-1.090241
C	2.828183	0.501656	1.404346
H	2.773979	-0.160712	2.272847
H	3.853519	0.475653	1.022556
H	2.629243	1.519570	1.752492
Si	-1.599162	0.010404	0.095442
C	-1.922816	1.780984	-0.431606
H	-1.714859	2.480190	0.382421

H	-1.309794	2.064825	-1.290325
H	-2.974407	1.898594	-0.712185
C	-1.748284	-1.149104	-1.369023
H	-2.777851	-1.162595	-1.741404
H	-1.489088	-2.174871	-1.091928
H	-1.099993	-0.846785	-2.195854
C	-2.827797	-0.503163	1.404136
H	-2.773608	0.158356	2.273282
H	-2.628533	-1.521368	1.751254
H	-3.853203	-0.477085	1.022538

HN(*i*-Pr)$_2$

C	-1.223330	0.132269	-0.240990
H	-1.093802	0.787383	-1.109673
C	-2.335004	-0.849394	-0.562092
H	-3.275008	-0.331061	-0.764730
H	-2.507057	-1.528188	0.280757
H	-2.076519	-1.454128	-1.433926
C	-1.594909	0.999140	0.957437
H	-1.714491	0.383871	1.855693
H	-2.538964	1.522575	0.782457
H	-0.836364	1.756504	1.168329
N	0.013121	-0.622318	-0.082766
H	-0.131864	-1.338489	0.621475
C	1.226066	0.115990	0.237197
H	1.109329	0.721666	1.152454
C	2.334391	-0.888776	0.490491
H	2.073009	-1.565841	1.309813
H	2.512674	-1.493567	-0.402943
H	3.265216	-0.385278	0.761843
C	1.597262	1.056132	-0.895653
H	0.858093	1.848638	-1.034045
H	2.556520	1.538139	-0.691471
H	1.680527	0.501845	-1.835014

HNMe$_2$

C	-1.201299	-0.221199	0.020071
H	-1.263106	-0.971557	-0.774031
H	-2.081669	0.419851	-0.062744
H	-1.254169	-0.756942	0.983478
N	0.000000	0.563005	-0.149196
H	0.000000	1.330657	0.510116
C	1.201299	-0.221199	0.020071
H	1.263106	-0.971557	-0.774031
H	2.081669	0.419851	-0.062744
H	1.254169	-0.756942	0.983478

H-radical

H	0.000000	0.000000	0.000000

I-

I	0.000000	0.000000	0.000000

I-radical

I	0.000000	0.000000	0.000000

N(*i*-Pr)$_2^-$

N	0.000040	0.000113	0.625478
C	-1.179496	0.197380	-0.137341
C	-2.280074	0.700310	0.789681
H	-1.975071	1.644311	1.250475
H	-3.236427	0.848092	0.272703
H	-2.426622	-0.025547	1.597894
C	-1.687549	-1.067420	-0.860854
H	-2.587724	-0.880401	-1.463893
H	-1.914128	-1.840767	-0.117248
H	-0.920542	-1.469429	-1.530430
H	-1.082978	0.973157	-0.944475
C	1.179473	-0.197405	-0.137438
C	1.687473	1.067177	-0.861367
H	2.587574	0.879969	-1.464457
H	1.914153	1.840730	-0.118005
H	0.920396	1.469012	-1.530969
C	2.280149	-0.700114	0.789588
H	1.975178	-1.643991	1.250657
H	3.236444	-0.848046	0.272544
H	2.426795	0.025946	1.597600
H	1.082821	-0.973396	-0.944347

N(*i*-Pr)$_2$-radical

C	-1.201224	0.108200	-0.231547
H	-0.988351	0.554490	-1.217315
C	-1.738613	-1.304166	-0.467938
H	-1.951957	-1.791902	0.486271
H	-1.018707	-1.918440	-1.013595
H	-2.661718	-1.262490	-1.051377
C	-2.216064	0.968297	0.499504
H	-1.827093	1.976302	0.660683
H	-2.445401	0.536233	1.476880
H	-3.144600	1.046946	-0.072130
N	-0.000010	0.000010	0.551381
C	1.201236	-0.108238	-0.231491
H	0.988408	-0.554654	-1.217211
C	1.738595	1.304115	-0.468035
H	2.661732	1.262390	-1.051420
H	1.018701	1.918299	-1.013809
H	1.951876	1.791978	0.486123
C	2.216077	-0.968218	0.499695
H	3.144629	-1.046918	-0.071906
H	2.445381	-0.536028	1.477023
H	1.827125	-1.976212	0.660992

NMe$_2^-$

N	0.000000	0.658667	0.000007
C	1.139564	-0.165210	-0.000004
H	1.221734	-0.868411	-0.883383
H	1.221924	-0.868285	0.883487
H	2.071180	0.422623	-0.000104
C	-1.139564	-0.165210	-0.000005
H	-1.221739	-0.868406	-0.883387
H	-1.221917	-0.868290	0.883484
H	-2.071180	0.422623	-0.000093

NMe$_2$-radical

C	-1.181143	0.168356	0.002230
H	-2.072896	-0.453911	0.095402
H	-1.258370	0.745860	-0.933025
H	-1.173856	0.908240	0.818011
N	0.000011	-0.631540	0.000187
C	1.181173	0.168292	-0.001997
H	1.254101	0.754773	0.927942
H	2.073402	-0.454561	-0.086230
H	1.177359	0.900491	-0.824805

4

C	2.549511	2.948134	0.590010
C	2.733180	1.576475	0.650235
C	1.793015	0.731436	0.068668
C	0.679741	1.279341	-0.589270
C	0.507459	2.653791	-0.653978
C	1.439043	3.486772	-0.052513
H	1.305190	4.561942	-0.088854
H	-0.350710	3.075703	-1.166236
P	-0.352494	-0.000010	-1.364180
C	0.679821	-1.279307	-0.589293
C	0.507627	-2.653768	-0.654027
C	1.439265	-3.486701	-0.052580
C	2.549702	-2.948004	0.589948
C	2.733284	-1.576335	0.650199
C	1.793062	-0.731345	0.068652

H	3.601758	-1.167098	1.154713
H	3.278088	-3.607792	1.047708
H	1.305481	-4.561879	-0.088941
H	-0.350518	-3.075725	-1.166290
C	-1.861315	-0.000057	-0.326679
C	-1.815837	-0.000173	1.066818
C	-2.987735	-0.000192	1.802370
C	-4.218338	-0.000095	1.155070
C	-4.272244	0.000020	-0.228899
C	-3.095883	0.000037	-0.967654
H	-3.134464	0.000122	-2.052021
H	-5.229690	0.000095	-0.737683
H	-5.135030	-0.000110	1.734222
H	-2.944659	-0.000282	2.885942
H	-0.857708	-0.000249	1.575148
H	3.601678	1.167284	1.154745
H	3.277854	3.607959	1.047785

5

P	-0.000626	-0.000271	-1.239068
C	-1.625883	-0.217061	-0.431269
C	-2.703859	0.476596	-0.982437
C	-3.967038	0.381028	-0.421746
C	-4.175143	-0.427786	0.687183
C	-3.113423	-1.133964	1.232345
C	-1.844951	-1.027100	0.679969
H	-1.020604	-1.577837	1.118128
H	-3.270485	-1.770287	2.096394
H	-5.165193	-0.511996	1.121124
H	-4.793746	0.930799	-0.857729
H	-2.547772	1.098126	-1.858910
C	0.624832	1.515869	-0.431784
C	1.768837	2.097975	-0.978990
C	2.318322	3.239491	-0.418738
C	1.718429	3.828875	0.685735
C	0.571846	3.267285	1.227021
C	0.029403	2.115304	0.675167
H	-0.862763	1.680181	1.110485
H	0.096672	3.725248	2.087665
H	2.141403	4.728001	1.119591
H	3.211324	3.676654	-0.851517
H	2.232243	1.648208	-1.851719
C	1.000175	-1.299084	-0.431242
C	1.810669	-1.083430	0.680271
C	2.537675	-2.128085	1.233012
C	2.457691	-3.400679	0.687917
C	1.653937	-3.625191	-0.421411
C	0.939501	-2.579355	-0.982540
H	0.323613	-2.755354	-1.859144
H	1.591723	-4.616096	-0.857387
H	3.025517	-4.215864	1.122309
H	3.166772	-1.945684	2.097392
H	1.874569	-0.094157	1.118569

B

P	1.736830	0.084433	0.158461
C	1.057646	-0.196781	1.792958
C	-0.334240	-0.267185	1.872411
C	-0.930629	-0.426420	3.113199
C	-0.158993	-0.531842	4.259800
C	1.225679	-0.475830	4.173850
C	1.838123	-0.303420	2.944317
H	2.917135	-0.259649	2.896198
H	1.832815	-0.563820	5.067326
H	-0.635122	-0.660650	5.225452
H	-2.012564	-0.466396	3.165210
H	-0.997528	-0.195642	0.995862
C	1.027161	-1.097083	-0.990085
C	-0.047392	-0.716932	-1.789903
C	-0.599807	-1.628952	-2.676914
C	-0.097634	-2.914825	-2.759749
C	0.949910	-3.308587	-1.934365
C	1.507968	-2.408301	-1.047763
H	2.309710	-2.735076	-0.399820
H	1.324527	-4.324536	-1.974632
H	-0.537717	-3.625292	-3.450035
H	-1.445182	-1.326238	-3.281690
H	-0.483546	0.267860	-1.709196
C	1.267284	1.734922	-0.371120
C	1.504728	2.132592	-1.688472
C	1.229775	3.431810	-2.074526
C	0.710379	4.336226	-1.157460
C	0.464579	3.940725	0.146779
C	0.747873	2.643501	0.545039
H	0.551807	2.337403	1.564752
H	0.037621	4.636783	0.858484
H	0.479798	5.348418	-1.468581
H	1.403083	3.734557	-3.100354
H	1.885560	1.426086	-2.415818
N	-2.714976	-0.137293	-0.123690
Si	-3.277273	1.418353	-0.286459
C	-2.953440	2.478016	1.256066
H	-3.557373	2.117432	2.096062
H	-3.197326	3.535226	1.100760
H	-1.906185	2.420749	1.566712
C	-2.437785	2.336806	-1.716975
H	-1.349656	2.345669	-1.612379
H	-2.673111	1.853053	-2.671608
H	-2.764793	3.380151	-1.788770
C	-5.132437	1.620266	-0.621824
H	-5.410253	2.672860	-0.750332
H	-5.733352	1.222591	0.202151
H	-5.432492	1.088773	-1.531206
Si	-3.444029	-1.624599	0.028166
C	-4.259276	-2.279272	-1.554407
H	-4.785077	-3.227010	-1.391031
H	-3.521247	-2.443865	-2.346588
H	-4.990189	-1.558578	-1.936749
C	-2.208562	-2.967429	0.540438
H	-2.660950	-3.963635	0.476348
H	-1.866271	-2.832952	1.571536
H	-1.322665	-2.969002	-0.101483
C	-4.820220	-1.723729	1.331466
H	-4.470041	-1.365472	2.305871
H	-5.678423	-1.105093	1.049373
H	-5.186650	-2.747725	1.468919
C	3.576470	0.028174	0.140966
C	4.105866	1.293164	0.823158
H	3.791018	1.378825	1.863968
H	3.789332	2.196626	0.300652
H	5.198022	1.262572	0.811432
C	4.163554	-1.251991	0.813998
H	3.404361	-1.939914	1.187447
H	4.743372	-0.942528	1.686561
C	5.072146	-1.907652	-0.230256
C	5.414210	-0.787330	-1.200300
H	6.197261	-0.145122	-0.786329
H	5.766041	-1.149142	-2.167930
C	4.103016	-0.021390	-1.313911
H	3.422036	-0.578777	-1.962166
H	4.217248	0.980270	-1.730060
H	4.531965	-2.695662	-0.762438
H	5.949905	-2.369633	0.224649

B'

P	0.609823	-0.031141	0.138102
C	0.284486	-0.836228	1.748195
C	0.982802	-1.968865	2.150020
C	0.733035	-2.563785	3.377384
C	-0.201449	-2.015468	4.239390

C	-0.897185	-0.877844	3.855011
C	-0.668866	-0.303798	2.616422
H	-1.257689	0.543465	2.297142
H	-1.640293	-0.443776	4.514173
H	-0.395581	-2.476707	5.201048
H	1.272711	-3.462203	3.654050
H	1.716438	-2.416425	1.500012
C	0.513049	-0.963577	-1.433877
C	0.750211	-2.334331	-1.448160
C	0.660824	-3.060461	-2.627929
C	0.369279	-2.422347	-3.820813
C	0.143639	-1.053096	-3.818726
C	0.198751	-0.336409	-2.636593
H	-0.050010	0.712054	-2.656442
H	-0.101170	-0.537761	-4.740672
H	0.306695	-2.986864	-4.743903
H	0.826684	-4.131621	-2.605893
H	0.993721	-2.858175	-0.535188
C	0.670437	1.790277	0.259562
C	0.837272	2.610072	-0.853054
C	1.005014	3.978502	-0.711379
C	1.030852	4.554910	0.548304
C	0.880970	3.747580	1.664320
C	0.698584	2.381471	1.522522
H	0.597027	1.777458	2.412810
H	0.900634	4.179290	2.658383
H	1.166965	5.624435	0.658938
H	1.128731	4.592084	-1.596390
H	0.879750	2.189382	-1.846051
N	-1.477872	0.111333	-0.085496
Si	-2.412339	1.490655	-0.587577
C	-2.336430	2.985159	0.554951
H	-2.412930	2.696257	1.607449
H	-3.203431	3.617161	0.332848
H	-1.448074	3.603207	0.437090
C	-2.106120	2.122982	-2.344859
H	-2.326755	1.345178	-3.082381
H	-2.809707	2.943584	-2.523715
H	-1.112003	2.519834	-2.548629
C	-4.288896	1.210765	-0.659608
H	-4.750028	1.214438	0.328923
H	-4.630949	0.323441	-1.192280
H	-4.685750	2.078225	-1.198986
Si	-2.457519	-1.331274	0.012564
C	-3.063285	-1.848409	-1.699199
H	-3.357318	-1.014799	-2.339237
H	-3.910502	-2.539447	-1.633449
H	-2.253377	-2.376112	-2.213752
C	-1.719292	-2.940685	0.666290
H	-1.502072	-2.915370	1.734586
H	-0.856716	-3.339628	0.139195
H	-2.533116	-3.663574	0.524396
C	-3.899127	-1.142946	1.237074
H	-3.825189	-0.229193	1.830182
H	-4.876607	-1.141210	0.750291
H	-3.885674	-1.982947	1.938383
C	2.625593	-0.109597	0.217849
C	3.151896	0.422995	1.552451
H	2.912211	1.475268	1.702318
H	4.242304	0.344694	1.586745
H	2.766935	-0.137155	2.406298
C	3.353162	-1.446340	-0.067093
H	2.894295	-1.958922	-0.909587
H	3.357345	-2.131414	0.781211
C	4.786821	-1.073905	-0.462400
C	4.690503	0.338577	-1.053688
H	5.331072	1.033584	-0.506365
H	5.014668	0.372827	-2.095757
C	3.218927	0.747298	-0.921378
H	2.695198	0.530919	-1.857857
H	3.122428	1.814160	-0.730543
H	5.192200	-1.796859	-1.173606
H	5.449962	-1.089318	0.405406

C

P	-0.032997	-0.010135	-0.130513
C	0.931140	-0.257403	1.340829
C	0.216382	-0.355242	2.562104
C	0.827908	-0.814701	3.707958
C	2.165495	-1.211505	3.697389
C	2.879085	-1.131010	2.501242
C	2.282239	-0.674138	1.345400
H	2.875133	-0.619882	0.443683
H	3.923976	-1.422679	2.474872
H	2.640082	-1.577007	4.599830
H	0.256593	-0.862602	4.629153
H	-0.823351	-0.050236	2.603332
C	1.015431	0.462900	-1.568533
C	1.875550	1.693953	-1.209014
H	1.314805	2.622686	-1.328278
H	2.212113	1.642657	-0.169879
C	3.078475	1.581136	-2.135678
H	3.912043	2.208526	-1.814219
H	2.809489	1.893043	-3.149120
C	3.406744	0.093791	-2.103401
H	3.984782	-0.235950	-2.968660
H	4.010837	-0.126582	-1.218698
C	2.048190	-0.617122	-2.014915
H	2.087186	-1.479796	-1.349146
H	1.748689	-0.998132	-2.993881
C	0.087369	0.811558	-2.733075
H	0.689764	1.132847	-3.586781
H	-0.591621	1.628002	-2.480965
H	-0.508481	-0.045017	-3.052431
C	-1.108178	1.402571	0.236684
C	-0.645107	2.431808	1.054824
C	-1.415957	3.565375	1.253719
C	-2.651240	3.685621	0.632415
C	-3.113369	2.668393	-0.191408
C	-2.344001	1.534532	-0.394425
H	-2.703132	0.739979	-1.038279
H	-4.078028	2.757663	-0.677959
H	-3.253827	4.573080	0.789998
H	-1.051600	4.357060	1.898534
H	0.316234	2.333685	1.545699
C	-1.107557	-1.352805	-0.557431
C	-2.177818	-1.648073	0.325658
C	-2.884448	-2.825731	0.213596
C	-2.557647	-3.768849	-0.761093
C	-1.497056	-3.498842	-1.627736
C	-0.778558	-2.326847	-1.529398
H	0.037177	-2.157053	-2.219164
H	-1.232753	-4.216141	-2.397858
H	-3.113545	-4.695008	-0.841452
H	-3.707937	-3.014738	0.894419
H	-2.458094	-0.932593	1.090699

D

C	0.770931	0.014269	-0.090929
C	-0.081201	1.219113	0.117052
H	0.231614	2.075592	-0.491631
H	-0.032656	1.562575	1.165738
C	-1.490951	0.726178	-0.212938
H	-1.651532	0.779332	-1.294718
H	-2.278898	1.311608	0.265875
C	-1.474359	-0.734397	0.235542
C	-0.067046	-1.221501	-0.127224
H	-0.055586	-1.679722	-1.129083
H	0.298943	-1.999281	0.555820
H	-1.608477	-0.783156	1.321082

H	-2.268626	-1.331435	-0.217667
C	2.243975	0.006209	0.019748
H	2.685864	0.942274	-0.335478
H	2.690666	-0.815159	-0.550020
H	2.580595	-0.121850	1.062574

E

P	-0.024485	-0.013176	-1.263077
C	-1.652381	-0.077100	-0.433954
C	-2.014844	0.718034	0.650068
C	-3.276724	0.601198	1.215614
C	-4.186964	-0.315119	0.711185
C	-3.834842	-1.111464	-0.370104
C	-2.580741	-0.985259	-0.944331
H	-2.314635	-1.599300	-1.799283
H	-4.543298	-1.826251	-0.773717
H	-5.171555	-0.405855	1.155975
H	-3.546551	1.227505	2.058835
H	-1.308182	1.430885	1.058804
C	0.739673	1.446430	-0.473430
C	0.446154	2.735013	-0.941475
C	1.027356	3.846211	-0.351113
C	1.925687	3.705893	0.700776
C	2.248689	2.436429	1.177912
C	1.632405	1.381208	0.561594
H	2.949828	2.304331	1.994528
H	2.381924	4.582196	1.149003
H	0.784426	4.835855	-0.720957
C	0.883524	-1.361806	-0.424469
C	2.012307	-1.864994	-1.068860
C	2.769371	-2.868389	-0.484321
C	2.394474	-3.393228	0.743915
C	1.263501	-2.908420	1.385236
C	0.512886	-1.896521	0.806685
H	-0.367911	-1.521558	1.315241
H	0.964578	-3.318120	2.343807
H	2.979944	-4.184497	1.198638
H	3.648319	-3.247743	-0.993463
H	2.298724	-1.464992	-2.036567
H	-0.245529	2.856493	-1.770232

E-meta radical

P	0.003340	0.043618	-1.222293
C	-0.618910	1.549580	-0.389761
C	-0.009729	2.124373	0.733415
C	-0.599084	3.247149	1.244103
C	-1.726530	3.860918	0.772151
C	-2.322603	3.276735	-0.346147
C	-1.764891	2.144718	-0.919960
H	-2.228200	1.709218	-1.799401
H	-3.216709	3.718103	-0.773988
H	-2.138987	4.754987	1.226107
H	0.881467	1.686807	1.169146
C	1.627934	-0.185018	-0.418866
C	2.704346	0.522388	-0.955654
C	3.967327	0.418053	-0.396328
C	4.176505	-0.413130	0.695708
C	3.116433	-1.133234	1.225681
C	1.847902	-1.017954	0.675253
H	1.024649	-1.579642	1.101316
H	3.274848	-1.786631	2.076542
H	5.166311	-0.503339	1.128855
H	4.792859	0.978994	-0.819991
H	2.547316	1.161689	-1.818956
C	-1.001020	-1.263765	-0.434372
C	-0.950546	-2.532642	-1.012487
C	-1.668829	-3.585391	-0.469282
C	-2.465156	-3.378905	0.648833
C	-2.533933	-2.117560	1.221152
C	-1.803614	-1.065984	0.686272

H	-1.859296	-0.086117	1.146129
H	-3.156497	-1.949279	2.093009
H	-3.035668	-4.199335	1.069431
H	-1.615174	-4.567283	-0.926095
H	-0.340621	-2.694419	-1.895983

E-para radical

P	-0.000783	0.058585	-1.227527
C	0.101468	1.683612	-0.393759
C	0.857865	1.920326	0.752394
C	0.885649	3.186692	1.336767
C	0.133455	4.156782	0.734347
C	-0.630346	3.996739	-0.389840
C	-0.629188	2.727565	-0.963871
H	-1.205856	2.549424	-1.867023
H	-1.201740	4.806661	-0.829294
H	1.476950	3.374386	2.226232
H	1.433129	1.116905	1.199377
C	1.368436	-0.856427	-0.434151
C	2.642645	-0.681760	-0.975458
C	3.738609	-1.325610	-0.424919
C	3.572610	-2.172030	0.662957
C	2.307652	-2.365553	1.197121
C	1.211479	-1.709298	0.655302
H	0.228244	-1.861843	1.085227
H	2.171029	-3.028630	2.044289
H	4.427762	-2.684502	1.089055
H	4.723607	-1.175177	-0.852605
H	2.773744	-0.032531	-1.835737
C	-1.467621	-0.686323	-0.431762
C	-1.956932	-1.868591	-0.988809
C	-3.058484	-2.504972	-0.440846
C	-3.701427	-1.955874	0.660366
C	-3.232948	-0.772523	1.210641
C	-2.120276	-0.142512	0.671312
H	-1.758482	0.778145	1.114137
H	-3.733047	-0.336379	2.068431
H	-4.570133	-2.447094	1.084138
H	-3.423235	-3.426528	-0.880707
H	-1.466098	-2.293068	-1.859364

F

P	-0.352348	0.126686	-1.430294
C	0.710863	-1.182471	-0.730922
C	0.775345	-2.503326	-1.151730
C	1.661656	-3.376603	-0.536634
C	2.481305	-2.925885	0.489102
C	2.419739	-1.604515	0.913629
C	1.534820	-0.730096	0.304197
H	3.065117	-1.266301	1.717094
H	3.179520	-3.608464	0.960644
H	1.720730	-4.408314	-0.864150
H	0.140080	-2.850058	-1.959912
C	-1.862097	-0.028947	-0.386473
C	-2.037050	-1.000743	0.594457
C	-3.210826	-1.048671	1.333944
C	-4.219255	-0.124892	1.106435
C	-4.054474	0.845334	0.127114
C	-2.888591	0.886040	-0.619676
H	-2.771948	1.638097	-1.394443
H	-4.841280	1.567280	-0.061716
H	-5.134892	-0.163479	1.685675
H	-3.334343	-1.812077	2.094369
H	-1.254467	-1.726695	0.783965
C	0.596957	1.347747	-0.483071
C	0.811804	2.647292	-0.826620
C	1.768647	3.423486	-0.146562
C	2.614771	2.815459	0.808341
C	2.457322	1.518652	1.177787
C	1.303648	0.717771	0.682777

H	0.569341	0.676366	1.517353
H	3.130628	1.067479	1.898283
H	3.426929	3.395748	1.233624
H	1.920456	4.459950	-0.421330
H	0.281826	3.076375	-1.671626

F'

C	2.190842	3.006674	0.764436
C	2.691374	1.776072	0.491105
C	2.001201	0.860525	-0.462707
C	0.583923	1.276771	-0.757768
C	0.136709	2.531109	-0.464010
C	0.944552	3.433068	0.247825
H	0.573270	4.423175	0.481735
H	-0.881545	2.814340	-0.712611
P	-0.362813	-0.114348	-1.415731
C	0.776400	-1.248497	-0.555529
C	0.604365	-2.612173	-0.358532
C	1.591391	-3.338782	0.290032
C	2.752714	-2.709049	0.719893
C	2.935747	-1.349494	0.506887
C	1.946184	-0.614437	-0.127031
H	3.846585	-0.868406	0.846661
H	3.521759	-3.281765	1.225981
H	1.455780	-4.400546	0.462376
H	-0.302179	-3.103833	-0.695475
C	-1.864001	-0.133344	-0.371923
C	-1.808368	-0.083762	1.020763
C	-2.974409	-0.097850	1.765591
C	-4.208862	-0.157925	1.128373
C	-4.273158	-0.206014	-0.254563
C	-3.102615	-0.196010	-1.002231
H	-3.148126	-0.237103	-2.085683
H	-5.234006	-0.253359	-0.754676
H	-5.121001	-0.167739	1.714681
H	-2.924010	-0.061357	2.848256
H	-0.846203	-0.033523	1.518951
H	2.568392	0.924179	-1.416958
H	3.644535	1.464716	0.903932
H	2.744988	3.675159	1.415132

G

C	-1.517174	-0.773196	0.030015
C	-1.516707	0.773421	0.032556
C	-0.054598	1.184768	-0.163836
C	0.752637	0.000370	0.360286
C	-0.054348	-1.185198	-0.161777
H	0.193760	-2.125463	0.336996
H	0.168022	-1.317318	-1.227489
C	2.211169	0.000013	-0.044508
H	2.311805	-0.000543	-1.134925
H	2.733935	-0.882019	0.336201
H	2.734132	0.882298	0.335336
H	0.686352	0.001459	1.457272
H	0.194760	2.126515	0.331530
H	0.165476	1.313369	-1.230485
H	-2.164763	1.189317	-0.741621
H	-1.891014	1.150247	0.987798
H	-1.896665	-1.153217	0.981896
H	-2.161673	-1.185716	-0.748925

H

P	0.029992	0.031486	0.131593
C	1.090282	1.499163	-0.213011
C	0.777687	2.460007	-1.171088
C	1.582839	3.570591	-1.370283
C	2.720510	3.757954	-0.602876
C	3.031676	2.826853	0.374779
C	2.226594	1.715554	0.568363
H	2.500430	1.015659	1.344081
H	3.911387	2.958595	0.994809
H	3.353613	4.623564	-0.760607
H	1.311556	4.292915	-2.132067
H	-0.107232	2.367826	-1.780668
C	0.669832	-1.101937	1.444687
C	-0.130102	-1.674536	2.432167
C	0.417303	-2.477052	3.421990
C	1.785777	-2.685518	3.480252
C	2.596869	-2.109522	2.515591
C	2.039717	-1.354036	1.495844
H	2.673443	-0.974874	0.706825
H	3.669413	-2.268461	2.536813
H	2.215320	-3.296397	4.266129
H	-0.235699	-2.927847	4.160967
H	-1.193057	-1.497577	2.453578
C	-1.568375	-0.380401	-0.686975
C	-2.317344	-1.484966	-0.276929
C	-3.499555	-1.828798	-0.914056
C	-3.971036	-1.073651	-1.975486
C	-3.236610	0.023654	-2.397183
C	-2.049517	0.360557	-1.765694
H	-1.490987	1.197554	-2.158091
H	-3.581452	0.623246	-3.232328
H	-4.895375	-1.341341	-2.474016
H	-4.050041	-2.699872	-0.576397
H	-1.972532	-2.111800	0.535569
N	0.934564	-0.974086	-1.157663
C	1.700095	-0.601894	-2.355307
H	1.964126	-1.564232	-2.800655
C	0.937245	0.122369	-3.464664
H	1.521130	0.092792	-4.390589
H	0.764210	1.172805	-3.233840
H	-0.024864	-0.351341	-3.661137
C	0.563674	-2.393741	-1.199167
H	-0.061440	-2.577690	-0.322695
C	-0.258519	-2.813286	-2.420886
H	0.349565	-2.821894	-3.330172
H	-1.111544	-2.156549	-2.588336
H	-0.640178	-3.829193	-2.282983
C	-0.992159	1.185172	1.502296
C	-0.119080	1.579281	2.694995
H	0.778064	2.121963	2.396074
H	-0.680183	2.244614	3.361688
H	0.188588	0.719117	3.292833
C	-2.318094	0.583338	2.080614
C	-3.454666	1.536943	1.711495
C	-2.765173	2.880045	1.560027
H	-2.544603	3.309192	2.542999
H	-3.356238	3.615418	1.007746
C	-1.479930	2.497406	0.839987
H	-1.729138	2.338581	-0.212046
H	-0.720018	3.279032	0.878194
H	-3.885070	1.238857	0.749176
H	-4.263174	1.533078	2.446966
H	-2.225992	0.512260	3.169005
H	-2.560169	-0.412757	1.723104
C	1.754579	-3.341628	-1.059314
H	2.450940	-3.251384	-1.897848
H	1.407182	-4.378851	-1.042209
H	2.308577	-3.160220	-0.138868
C	3.053813	0.063572	-2.117399
H	3.596073	-0.434233	-1.310248
H	3.658656	-0.027743	-3.025344
H	2.984897	1.124541	-1.882288

H'

P	1.200913	-0.161537	-0.145699
C	0.605441	1.275279	-1.042829
C	0.566465	2.514326	-0.406312
C	0.236191	3.649037	-1.127528

C	-0.057590	3.552374	-2.479634
C	-0.030595	2.318550	-3.112241
C	0.299475	1.178979	-2.398592
H	0.301831	0.214886	-2.892118
H	-0.284333	2.238647	-4.162510
H	-0.328722	4.440429	-3.038726
H	0.192427	4.608770	-0.626667
H	0.776210	2.590833	0.653521
C	0.718568	0.048280	1.573636
C	-0.619191	0.362356	1.823460
C	-1.019267	0.587718	3.132569
C	-0.118751	0.493202	4.181265
C	1.202347	0.149162	3.929392
C	1.622671	-0.072007	2.629711
H	2.656008	-0.330324	2.453987
H	1.910788	0.056799	4.744563
H	-0.445172	0.678004	5.198801
H	-2.059550	0.831731	3.317854
H	-1.415156	0.385399	1.049790
C	0.445312	-1.624583	-0.861268
C	1.196629	-2.762640	-1.161467
C	0.585134	-3.861873	-1.737775
C	-0.774242	-3.830542	-2.018365
C	-1.521013	-2.709671	-1.694030
C	-0.928585	-1.600853	-1.107749
H	-1.605202	-0.780730	-0.800729
H	-2.588888	-2.681230	-1.879701
H	-1.251232	-4.688355	-2.479599
H	1.172982	-4.742392	-1.970096
H	2.256200	-2.802913	-0.951586
N	-3.233634	0.013044	0.162395
C	-3.892615	1.225974	-0.218091
C	-3.983202	2.256016	0.922506
H	-4.577350	3.139100	0.651489
H	-4.445494	1.810909	1.808806
H	-2.978573	2.595410	1.200468
C	-3.171701	1.858951	-1.398574
H	-3.176369	1.180781	-2.256355
H	-2.130137	2.051063	-1.128675
H	-3.621099	2.811185	-1.703061
C	-4.099574	-0.872370	0.882259
C	-5.143448	-1.577146	-0.003445
H	-5.711987	-0.854085	-0.594802
H	-5.863516	-2.163162	0.582819
H	-4.639281	-2.253913	-0.702539
C	3.027174	-0.168695	-0.377369
C	3.324264	-0.241708	-1.877846
H	2.956230	-1.160011	-2.336065
H	4.407053	-0.214998	-2.019518
H	2.899262	0.605928	-2.416568
C	3.637799	1.137822	0.183175
H	3.121625	1.467459	1.087526
H	3.571582	1.948796	-0.543007
C	5.067191	0.743643	0.531712
C	4.918343	-0.640546	1.150993
H	5.836546	-1.228169	1.105196
H	4.662810	-0.543959	2.210031
C	3.772047	-1.311342	0.378008
H	3.123896	-1.890178	1.038066
H	4.165764	-2.014680	-0.358403
H	5.540439	1.461215	1.204039
H	5.678994	0.696016	-0.373177
C	-3.297596	-1.941705	1.614809
H	-4.941614	1.055912	-0.548904
H	-2.629374	-1.500948	2.357789
H	-2.679920	-2.504316	0.907751
H	-3.950718	-2.655719	2.128282
H	-4.691295	-0.343348	1.668448

I

P	-0.135710	0.063089	-0.250540
C	-1.234510	-1.418813	-0.381423
C	-0.776267	-2.688931	-0.729244
C	-1.632633	-3.778248	-0.767054
C	-2.974439	-3.625918	-0.457759
C	-3.446932	-2.369949	-0.114380
C	-2.588399	-1.281017	-0.076960
H	-2.991399	-0.318206	0.204658
H	-4.493881	-2.228044	0.129714
H	-3.645451	-4.476728	-0.486917
H	-1.242513	-4.751117	-1.044540
H	0.259721	-2.851259	-0.990017
C	-1.024965	1.647242	0.097683
C	-0.543222	2.597985	0.993520
C	-1.238648	3.774975	1.231194
C	-2.450397	4.010466	0.603394
C	-2.948138	3.068026	-0.284034
C	-2.233913	1.911105	-0.548567
H	-2.592744	1.216070	-1.295157
H	-3.890759	3.240471	-0.791649
H	-3.000279	4.924353	0.798258
H	-0.829182	4.505108	1.920292
H	0.383302	2.435011	1.521758
C	1.667907	0.111609	-0.621058
C	2.400960	-1.011023	-1.006980
C	3.739111	-0.906520	-1.362351
C	4.378306	0.321200	-1.344475
C	3.658141	1.449593	-0.981746
C	2.321377	1.348029	-0.633056
H	1.785251	2.250893	-0.366035
H	4.137777	2.422148	-0.969552
H	5.424466	0.400551	-1.616491
H	4.281527	-1.799865	-1.651788
H	1.947131	-1.991381	-1.034468
N	-0.436936	0.380595	-2.073414
C	-0.228146	-0.656008	-3.051692
H	-0.603413	-0.316700	-4.025539
H	-0.767804	-1.568614	-2.806538
H	0.837275	-0.914595	-3.201166
C	0.031895	1.625275	-2.638364
H	1.078915	1.574703	-2.981611
H	-0.039846	2.449495	-1.929880
H	-0.580745	1.895450	-3.509461
C	0.201572	-0.357316	1.687001
C	-1.046292	-0.185525	2.554801
H	-0.835251	-0.516042	3.578166
H	-1.377820	0.852070	2.617986
H	-1.884442	-0.785852	2.197806
C	1.371008	0.444348	2.350350
C	2.411081	-0.572038	2.823538
C	1.605606	-1.840168	3.040659
H	1.043058	-1.779349	3.978116
H	2.218210	-2.744316	3.086201
C	0.663330	-1.828250	1.845493
H	1.231210	-2.162892	0.975152
H	-0.183136	-2.507681	1.956358
H	3.147302	-0.740682	2.030569
H	2.955937	-0.235838	3.709075
H	0.976215	1.002669	3.205061
H	1.845214	1.168891	1.692694

TS1

P	-0.044171	-0.063981	-0.194952
C	1.515930	-1.037241	-0.357241
C	2.793992	-0.513361	-0.147557
C	3.921944	-1.264762	-0.440787
C	3.802237	-2.552203	-0.943533
C	2.537089	-3.080510	-1.169024
C	1.410715	-2.323589	-0.900215

H	0.427513	-2.728727	-1.122186
H	2.428887	-4.080502	-1.574334
H	4.686797	-3.135890	-1.169940
H	4.904869	-0.841312	-0.264522
H	2.910162	0.485232	0.255414
C	-1.832346	0.663427	-1.352519
C	-1.288819	0.832398	-2.775491
H	-1.371303	1.866964	-3.120942
H	-0.227019	0.559934	-2.831112
C	-2.089305	-0.145979	-3.638032
H	-1.554046	-0.456350	-4.538396
H	-3.030808	0.313660	-3.957613
C	-2.380891	-1.290086	-2.675979
H	-3.167709	-1.964265	-3.021109
H	-1.473295	-1.886915	-2.531892
C	-2.733790	-0.564894	-1.383062
H	-2.631081	-1.197559	-0.496221
H	-3.782926	-0.240909	-1.413058
C	-2.472645	1.900545	-0.779363
H	-3.184420	2.318439	-1.507827
H	-1.750847	2.687730	-0.550274
H	-3.043466	1.685563	0.125274
C	0.631319	1.554572	0.303793
C	0.921296	2.499418	-0.679383
C	1.490047	3.717819	-0.343730
C	1.773772	4.011555	0.982304
C	1.490788	3.078341	1.967866
C	0.925963	1.856848	1.630745
H	0.717342	1.129030	2.405516
H	1.714262	3.297957	3.005709
H	2.216179	4.965340	1.245996
H	1.711965	4.440051	-1.121028
H	0.704659	2.280210	-1.717732
C	-0.772929	-0.741903	1.321536
C	-0.464073	-2.017492	1.798058
C	-1.103096	-2.530678	2.914060
C	-2.059189	-1.780987	3.586224
C	-2.359340	-0.504478	3.136630
C	-1.723853	0.010379	2.018038
H	-1.950665	1.018422	1.698341
H	-3.089580	0.100779	3.662049
H	-2.557022	-2.185039	4.459926
H	-0.845795	-3.523760	3.264727
H	0.291026	-2.611196	1.299476

TS2

P	-1.336256	-0.170078	-0.680404
C	-0.891266	-0.914771	0.937380
C	0.445193	-0.988639	1.285472
C	0.906428	-1.518368	2.467523
C	-0.025736	-2.025541	3.367903
C	-1.376109	-1.982789	3.054014
C	-1.808678	-1.432439	1.855602
H	-2.868662	-1.401580	1.628938
H	-2.105477	-2.378868	3.751905
H	0.305811	-2.456239	4.306392
H	1.967441	-1.547722	2.689588
H	1.320774	-0.542103	0.415399
C	-3.133973	-0.490235	-0.741847
C	-4.117369	0.474418	-0.542857
C	-5.461074	0.144660	-0.660220
C	-5.839407	-1.152455	-0.968493
C	-4.865807	-2.121300	-1.175092
C	-3.525624	-1.788960	-1.076003
H	-2.768770	-2.546303	-1.257604
H	-5.152141	-3.136659	-1.425932
H	-6.889366	-1.408311	-1.055515
H	-6.215297	0.908498	-0.505690
H	-3.836276	1.491267	-0.295909
C	-1.247786	1.606166	-0.264649
C	-1.580567	2.123131	0.986527
C	-1.486083	3.483344	1.231481
C	-1.051976	4.343014	0.230805
C	-0.712054	3.837865	-1.014998
C	-0.807457	2.475277	-1.259733
H	-0.525029	2.075032	-2.227822
H	-0.362187	4.503748	-1.795854
H	-0.970007	5.406192	0.426945
H	-1.742870	3.874121	2.209812
H	-1.905592	1.455638	1.776872
N	2.220978	-0.240612	-0.394641
Si	2.747047	-1.712261	-1.152480
C	3.934649	-1.231305	-2.519562
H	4.843313	-0.761337	-2.134512
H	4.238821	-2.128068	-3.069663
H	3.467996	-0.546045	-3.231473
C	3.617895	-2.875424	0.032517
H	2.938688	-3.219553	0.818167
H	4.477919	-2.403581	0.515354
H	3.978356	-3.764765	-0.495138
C	1.287226	-2.624640	-1.875066
H	1.622925	-3.544750	-2.364742
H	0.570834	-2.907624	-1.098769
H	0.756882	-2.018064	-2.612536
Si	3.119716	1.122774	0.200341
C	2.084264	2.082027	1.420065
H	1.191420	2.495157	0.946683
H	1.764524	1.473747	2.269798
H	2.668676	2.919823	1.814093
C	4.722586	0.623609	1.038339
H	5.267387	1.506738	1.388313
H	5.387230	0.078565	0.361863
H	4.537449	-0.012282	1.909183
C	3.526993	2.225523	-1.257002
H	2.614785	2.528204	-1.778169
H	4.180136	1.730413	-1.979650
H	4.036998	3.131992	-0.914467

TS3

P	0.264374	-0.130414	-1.404439
C	-0.712807	-1.359197	-0.477145
C	-0.931175	-1.117674	0.902023
C	-1.897176	-1.873903	1.584628
C	-2.667233	-2.797047	0.900307
C	-2.460468	-3.018524	-0.456851
C	-1.488309	-2.290274	-1.140751
H	-1.352940	-2.434693	-2.207708
H	-3.058958	-3.751207	-0.985820
H	-3.421649	-3.368156	1.430196
H	-2.033558	-1.732356	2.650956
H	-0.172632	-0.596421	1.477389
C	-0.672160	1.256519	-0.654188
C	-1.481930	0.979300	0.422223
C	-2.265761	1.893405	1.075680
C	-2.235882	3.203201	0.596320
C	-1.435134	3.534795	-0.490149
C	-0.658330	2.571305	-1.120316
H	-0.042868	2.838993	-1.974027
H	-1.422504	4.555740	-0.854581
H	-2.845375	3.964661	1.071971
H	-2.887051	1.622265	1.922943
C	1.817023	-0.087635	-0.429825
C	2.165466	0.940076	0.441572
C	3.376382	0.905913	1.120338
C	4.248275	-0.156529	0.942303
C	3.909576	-1.185273	0.072836
C	2.708850	-1.145043	-0.615146
H	2.455092	-1.946963	-1.302032
H	4.587686	-2.018450	-0.074869
H	5.191657	-0.183135	1.475835

H	3.634799	1.713989	1.795952
H	1.487439	1.771460	0.597226

TS3'

P	-0.276256	0.036390	-1.403003
C	0.640545	-1.343798	-0.644624
C	2.048219	-1.250066	-0.798814
C	2.875611	-2.151172	-0.108994
C	2.325353	-3.085145	0.748503
C	0.944723	-3.172726	0.892382
C	0.109232	-2.297519	0.200930
H	-0.963217	-2.349156	0.351483
H	0.513329	-3.916755	1.552325
H	2.969809	-3.769081	1.289422
H	3.948403	-2.112969	-0.261198
H	2.458928	-0.724785	-1.655713
C	0.773755	1.265549	-0.535131
C	1.984809	0.833168	-0.046558
C	2.904840	1.623841	0.590542
C	2.570217	2.966428	0.765323
C	1.359172	3.452169	0.285914
C	0.465325	2.616224	-0.369201
H	-0.476609	3.010548	-0.738639
H	1.109395	4.498290	0.423275
H	3.258459	3.632545	1.275155
H	3.847983	1.230925	0.956268
C	-1.820690	0.077743	-0.431250
C	-1.852313	0.351959	0.936009
C	-3.013851	-0.191653	-1.096301
C	-3.055520	0.353187	1.620955
H	-0.929123	0.563997	1.463862
C	-4.219350	-0.198769	-0.407781
H	-2.996039	-0.395983	-2.161967
C	-4.240635	0.075813	0.950387
H	-3.070657	0.569666	2.683425
H	-5.141953	-0.412210	-0.935758
H	-5.181508	0.077718	1.489351

TS4

C	2.917386	-2.896151	-0.434732
C	3.121002	-1.575783	-0.222026
C	2.050459	-0.759894	0.428394
C	0.675666	-1.345045	0.159698
C	0.525982	-2.708636	-0.018178
C	1.646542	-3.502841	-0.227936
H	1.529900	-4.568976	-0.377920
H	-0.462517	-3.148955	-0.083624
P	-0.397533	-0.010300	0.066048
C	0.758876	1.311267	0.061188
C	0.610328	2.690040	-0.060452
C	1.743502	3.488199	-0.074179
C	3.007986	2.926102	0.034784
C	3.150490	1.549419	0.174156
C	2.036828	0.732373	0.187923
H	4.139527	1.116659	0.279232
H	3.886623	3.560039	0.016701
H	1.635972	4.562287	-0.174340
H	-0.369824	3.142112	-0.141857
H	2.201704	-0.857343	1.526865
H	4.077080	-1.112115	-0.434768
H	3.727361	-3.505407	-0.822678
C	-2.148410	0.031111	0.021403
C	-2.840622	1.184126	-0.375231
C	-2.872241	-1.117316	0.375815
C	-4.222510	1.182887	-0.400564
H	-2.298487	2.071273	-0.676994
C	-4.253496	-1.103845	0.324893
H	-2.351456	-2.008886	0.703977
C	-4.938251	0.042736	-0.056917
H	-4.746889	2.081794	-0.704809
H	-4.801892	-1.998299	0.598443
H	-6.021205	0.047822	-0.087176

TS5

P	-0.438004	0.008597	-1.013516
C	0.155208	-1.378610	0.027053
C	1.514412	-1.633024	0.057856
C	2.087117	-2.649954	0.780261
C	1.250041	-3.492994	1.509604
C	-0.119985	-3.279993	1.493898
C	-0.667710	-2.232114	0.765063
H	-1.740369	-2.071914	0.774588
H	-0.774317	-3.932276	2.061669
H	1.670393	-4.311610	2.084048
H	3.161450	-2.806824	0.783004
H	2.365253	-0.741071	-0.753610
C	-2.221081	0.041720	-0.610972
C	-2.795336	0.851946	0.364966
C	-4.164190	0.819813	0.589459
C	-4.975078	-0.027558	-0.150615
C	-4.413408	-0.836544	-1.128695
C	-3.048853	-0.791972	-1.364793
H	-2.615615	-1.411662	-2.144093
H	-5.041564	-1.495073	-1.718215
H	-6.044157	-0.051928	0.028337
H	-4.598182	1.460137	1.349596
H	-2.172193	1.516154	0.952195
C	0.219900	1.432789	-0.076315
C	0.468054	1.406742	1.295518
C	0.978097	2.523639	1.938906
C	1.247876	3.679923	1.219316
C	1.007745	3.714586	-0.146346
C	0.501491	2.595311	-0.790239
H	0.333961	2.615435	-1.862012
H	1.225737	4.611711	-0.714904
H	1.652502	4.551004	1.722517
H	1.169782	2.490090	3.005785
H	0.269909	0.503763	1.862020
N	3.219882	-0.161904	-1.241312
C	3.852456	0.644697	-0.228178
H	4.592337	1.297270	-0.706036
H	3.112427	1.277981	0.262843
H	4.382053	0.058669	0.539767
C	4.119388	-1.126333	-1.821101
H	4.860443	-0.600211	-2.434184
H	4.677126	-1.719797	-1.078541
H	3.567057	-1.807882	-2.470734

TS6

P	-0.864070	-0.122614	-0.761424
C	-0.278433	-1.067421	0.697758
C	1.084974	-1.170201	0.921915
C	1.626150	-1.894740	1.958796
C	0.766450	-2.560727	2.828687
C	-0.604056	-2.479810	2.635389
C	-1.125083	-1.741248	1.582244
H	-2.199206	-1.688976	1.442460
H	-1.278071	-2.996546	3.309655
H	1.170366	-3.143417	3.649848
H	2.699636	-1.963473	2.096669
H	1.942340	-0.515735	-0.049220
C	-2.664107	-0.441102	-0.704089
C	-3.600493	0.423056	-0.142920
C	-4.951835	0.106978	-0.164932
C	-5.383669	-1.078979	-0.738906
C	-4.458384	-1.946259	-1.304230
C	-3.111628	-1.622930	-1.297248
H	-2.393051	-2.295476	-1.755918
H	-4.788718	-2.871958	-1.762316
H	-6.439571	-1.324834	-0.753017

H	-5.669871	0.791834	0.272807
H	-3.274583	1.350222	0.313892
C	-0.755482	1.594180	-0.149108
C	-0.729751	1.937489	1.200626
C	-0.633032	3.265754	1.585921
C	-0.567799	4.267314	0.627602
C	-0.596590	3.935844	-0.719406
C	-0.682212	2.606656	-1.104063
H	-0.680618	2.346126	-2.157638
H	-0.539770	4.713379	-1.473065
H	-0.488780	5.305410	0.930596
H	-0.607012	3.519731	2.639956
H	-0.775007	1.160546	1.955577
N	2.752552	-0.130932	-0.795903
C	3.638667	0.790376	-0.099841
C	3.365984	-1.300089	-1.405240
C	4.608991	0.170336	0.899340
H	5.280692	0.937864	1.292629
H	4.072553	-0.262715	1.747364
H	5.229746	-0.608581	0.451769
C	2.283854	-2.151866	-2.042753
H	1.563935	-2.502317	-1.300214
H	1.738324	-1.576086	-2.793986
H	2.723096	-3.029029	-2.524592
C	4.385971	-0.869614	-2.452232
H	4.767248	-1.748012	-2.978037
H	3.918870	-0.206888	-3.185933
H	5.239678	-0.349026	-2.013999
C	2.808410	1.877658	0.554881
H	3.452169	2.652396	0.978110
H	2.135534	2.341490	-0.167614
H	2.201613	1.467095	1.366158
H	3.892769	-1.915575	-0.657990
H	4.237493	1.264352	-0.890416

NMR Spectra

Cyclopentyltris(phenyl-d_5)phosphonium bromide bromide (**1-*d*$_{15}$**) – ^{1}H NMR spectrum ($CDCl_3$)

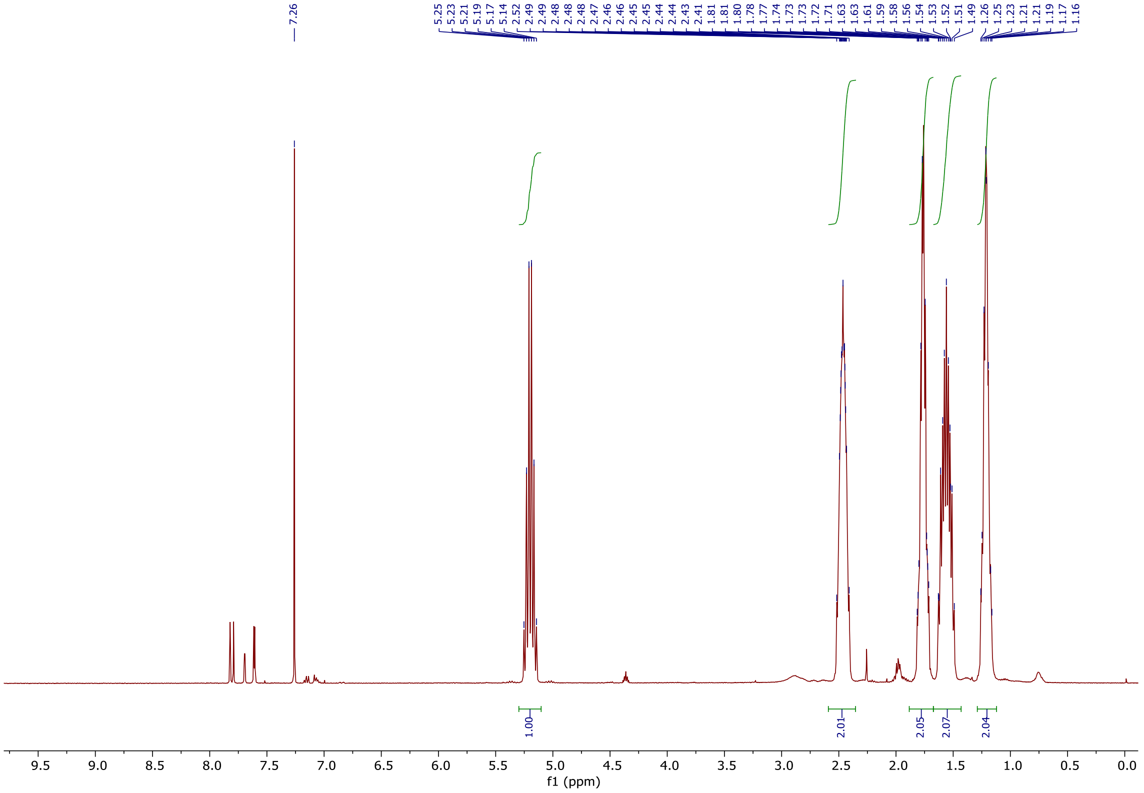

Cyclopentyltris(phenyl-d_5)phosphonium bromide bromide (**1-*d*$_{15}$**) – $^{13}C\{^1H\}$ NMR spectrum ($CDCl_3$)

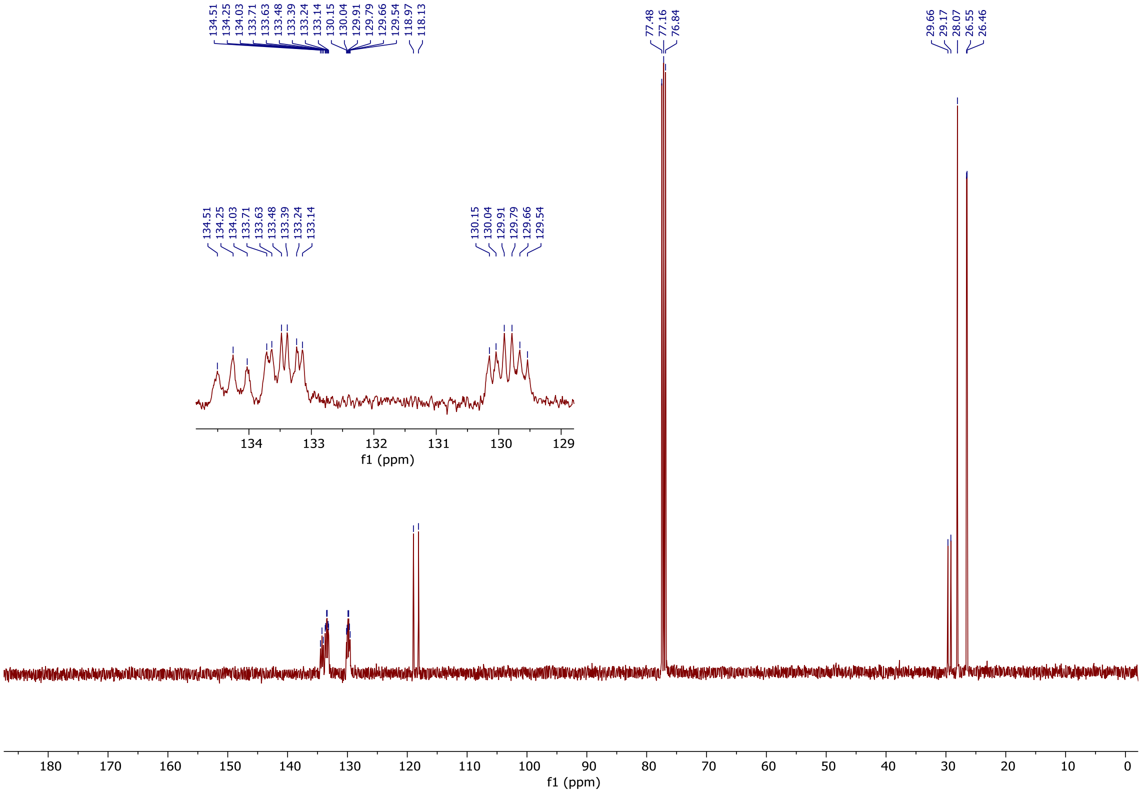

Cyclopentyltris(phenyl-d_5)phosphonium bromide bromide (**1-*d*$_{15}$**) – ^{31}P{^{1}H} NMR spectrum ($CDCl_3$)

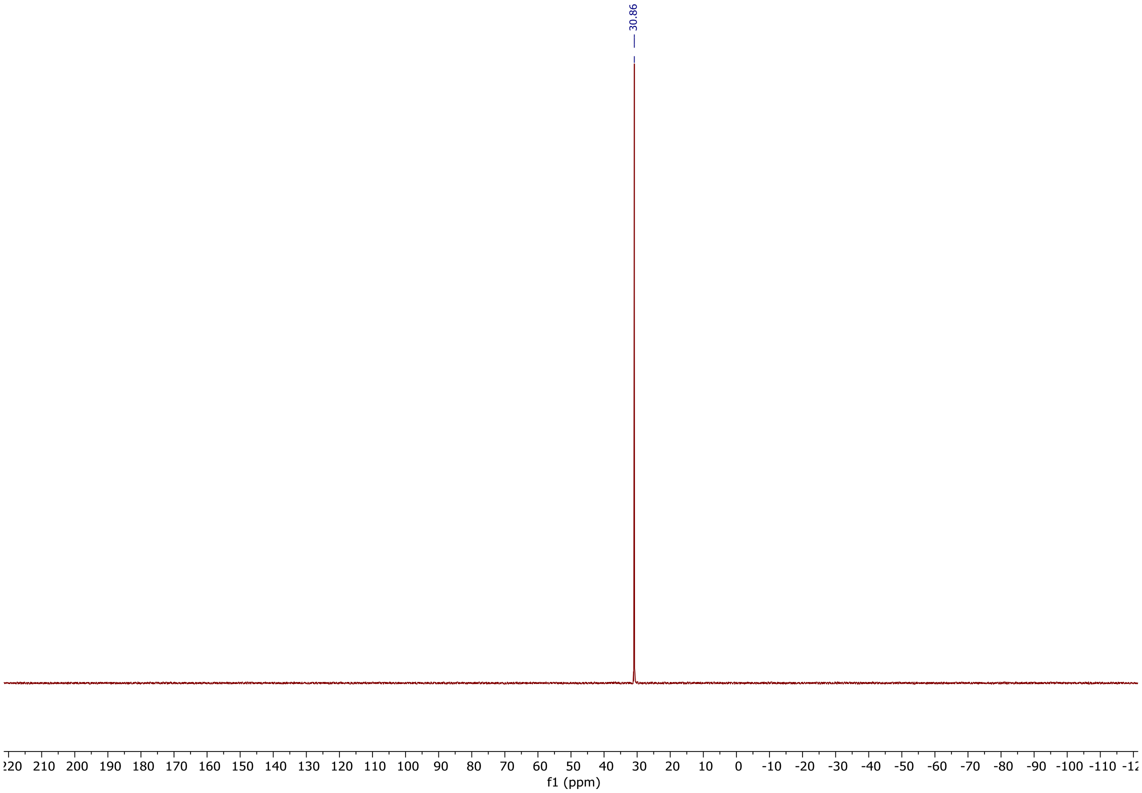

(3-Iodopropyl)benzene (**2**) – ^{1}H NMR spectrum ($CDCl_3$)

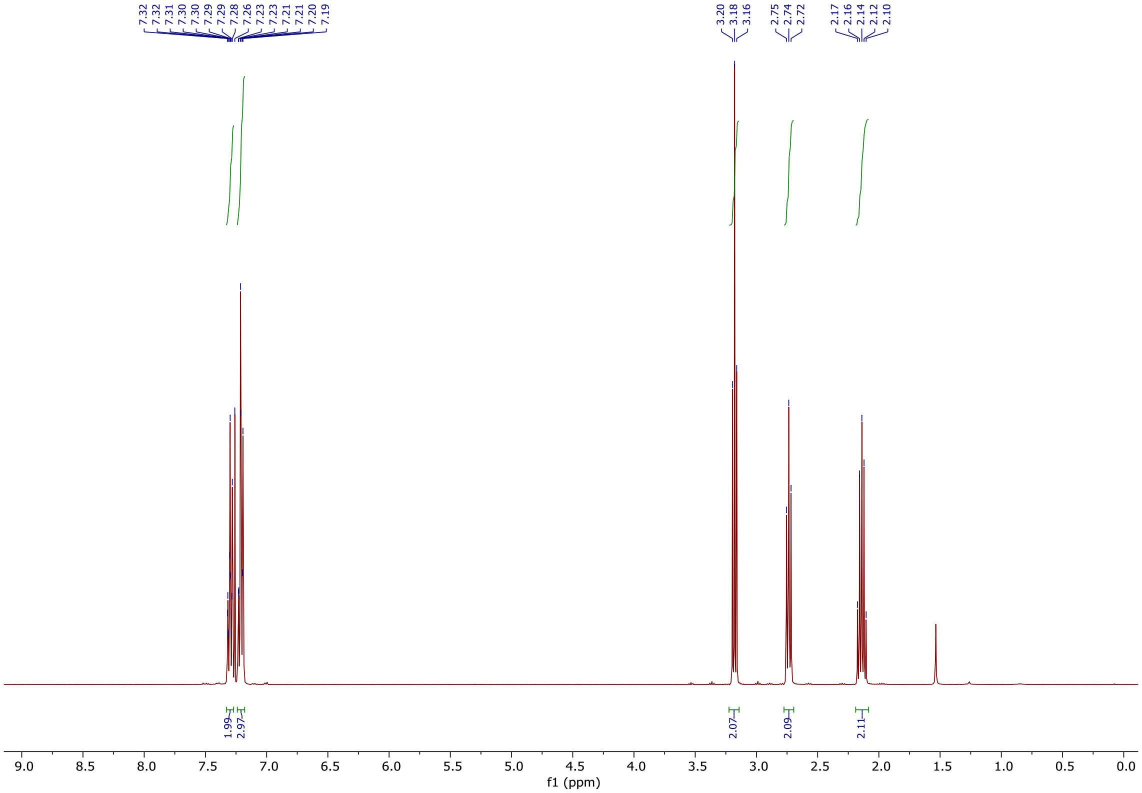

(3-Iodopropyl)benzene (**2**) – $^{13}C\{^1H\}$ NMR spectrum ($CDCl_3$)

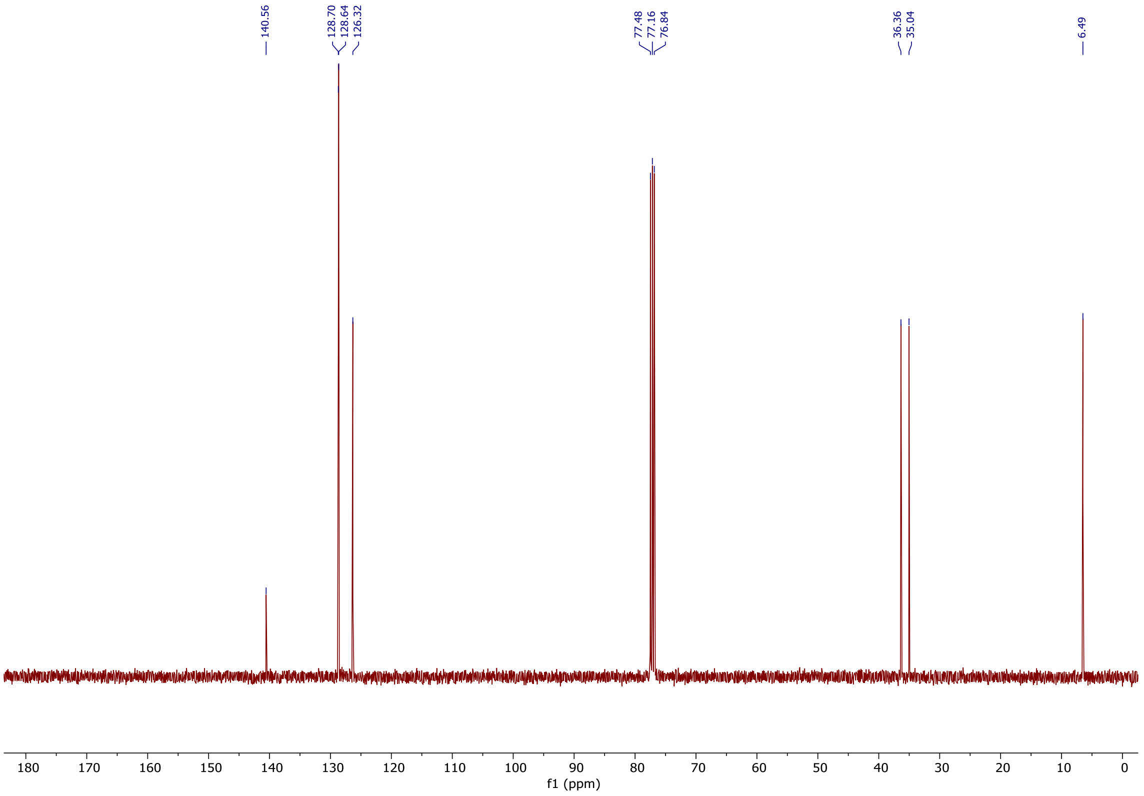

Phosphonium salt **3** – ^1H NMR spectrum ($CDCl_3$)

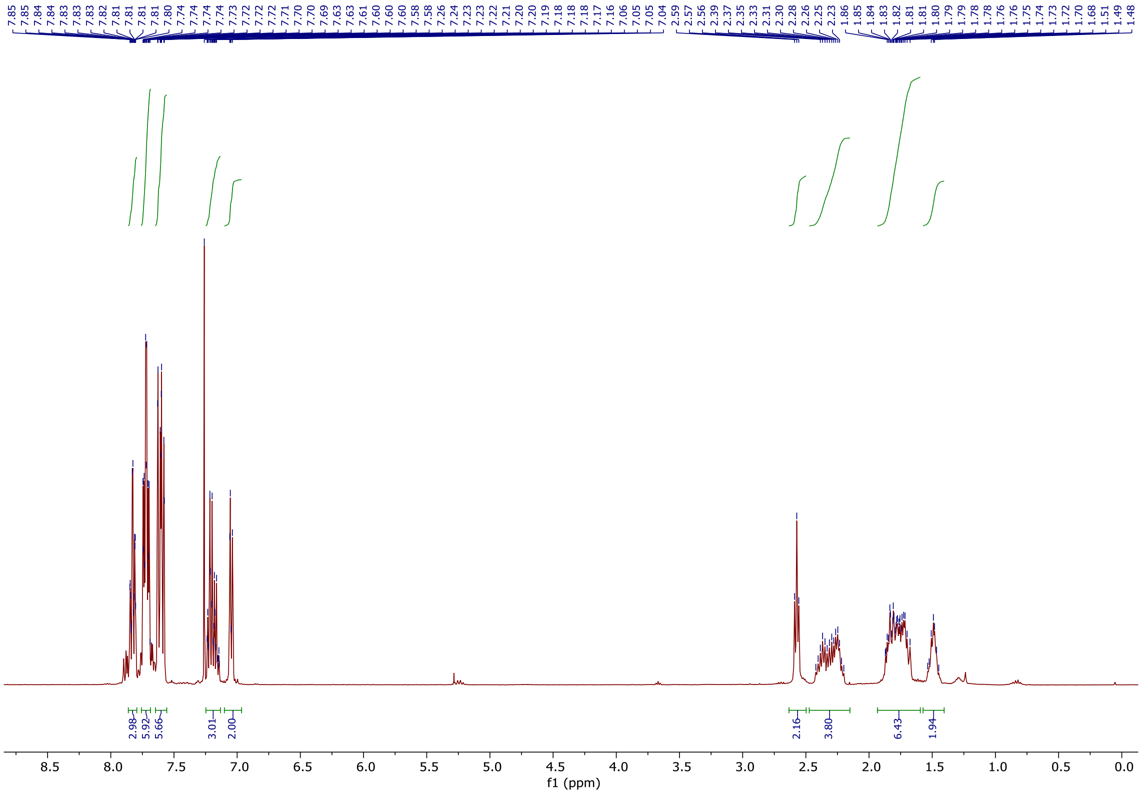

Phosphonium salt **3** – $^{13}C\{^{1}H\}$ NMR spectrum ($CDCl_3$)

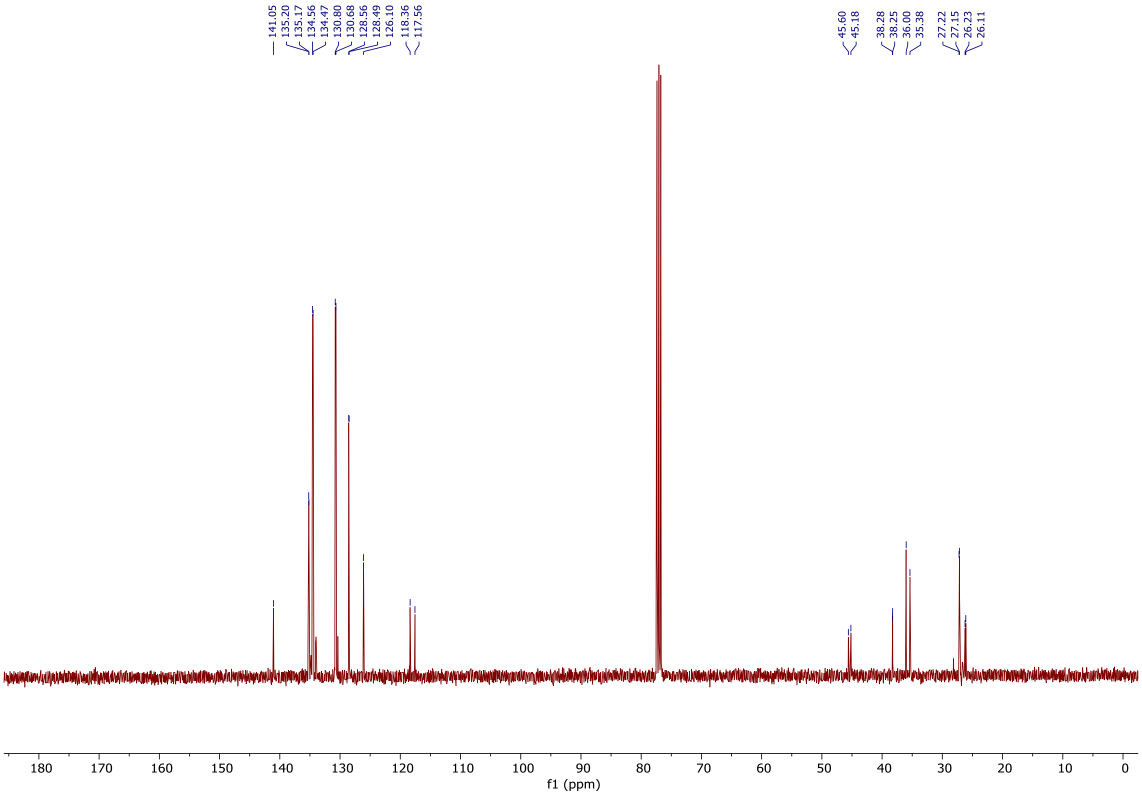

Phosphonium salt **3** – $^{31}P\{^{1}H\}$ qNMR spectrum ($CDCl_3$)

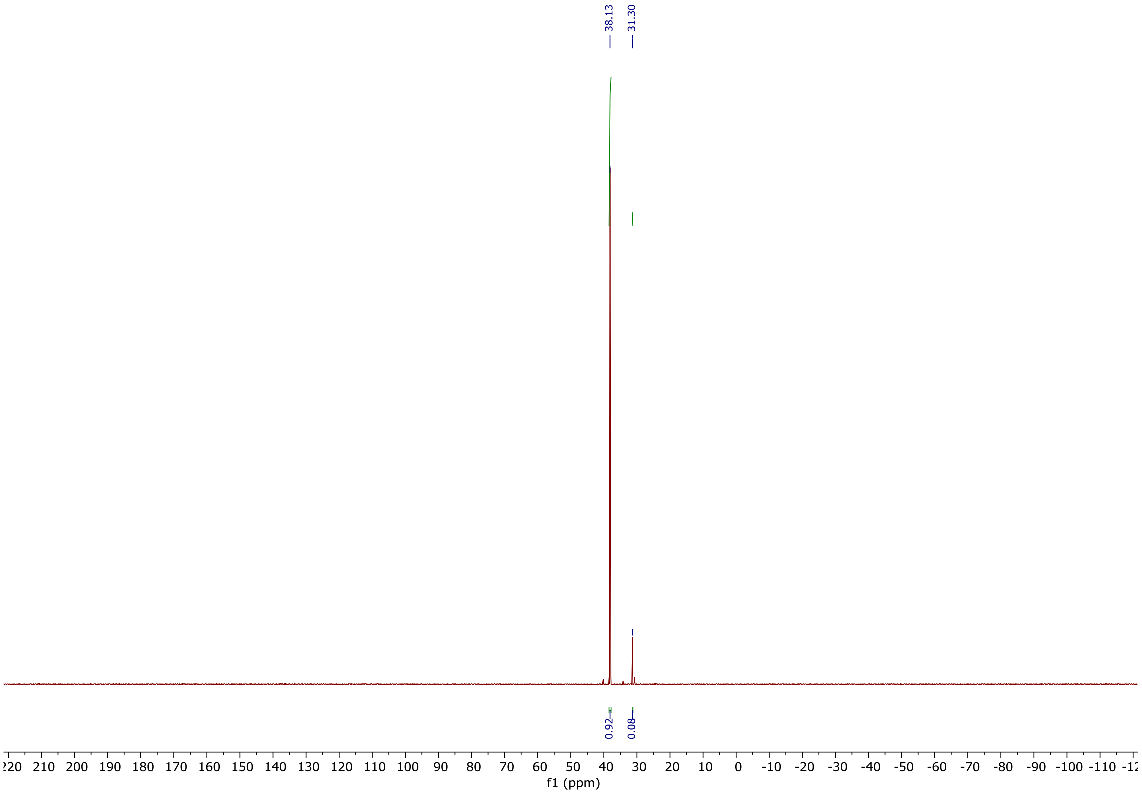

Dibenzophosphole **4** – ^{1}H NMR spectrum ($CDCl_3$)

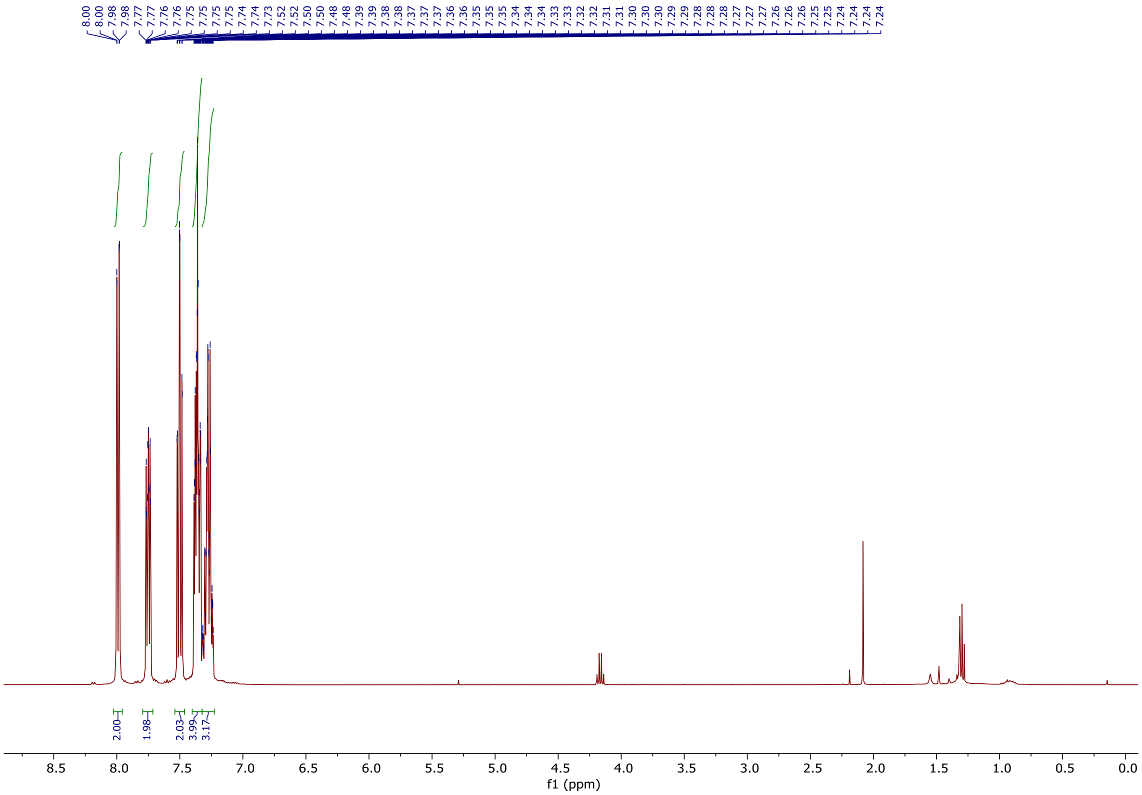

Dibenzophosphole **4** – ^{13}C{^{1}H} NMR spectrum ($CDCl_3$)

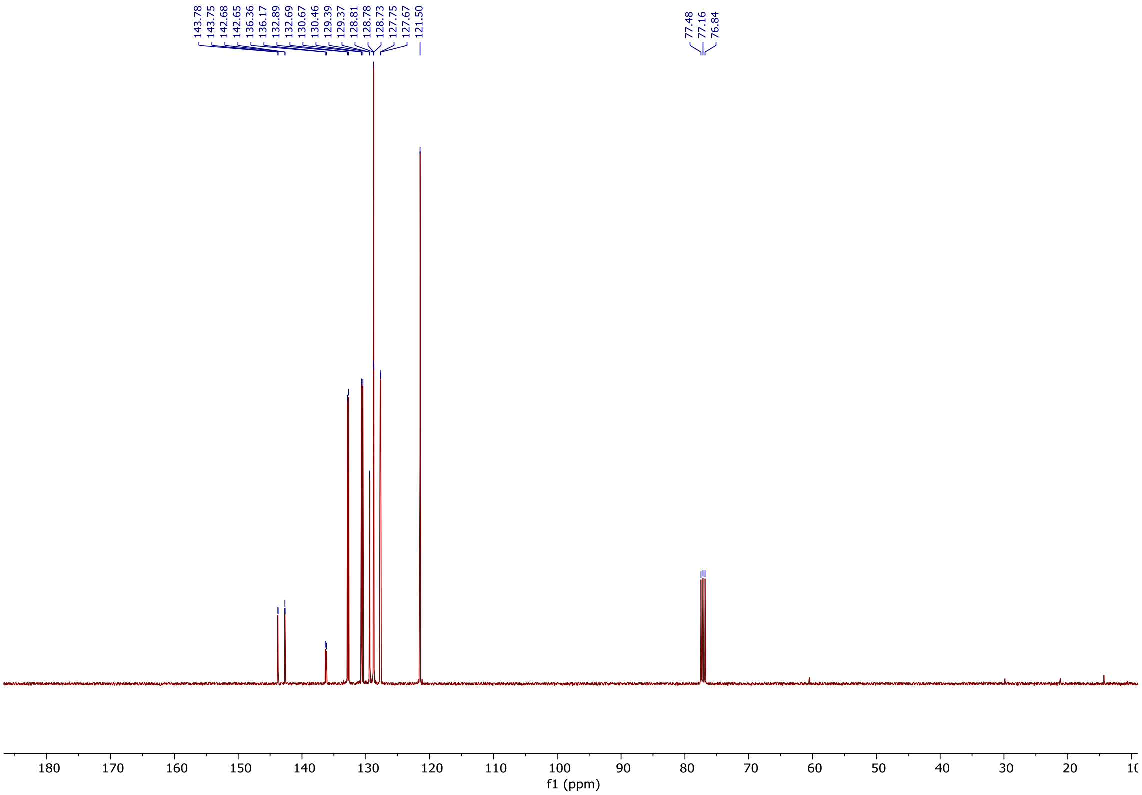

Dibenzophosphole **4** – $^{31}P\{^{1}H\}$ NMR spectrum ($CDCl_3$)

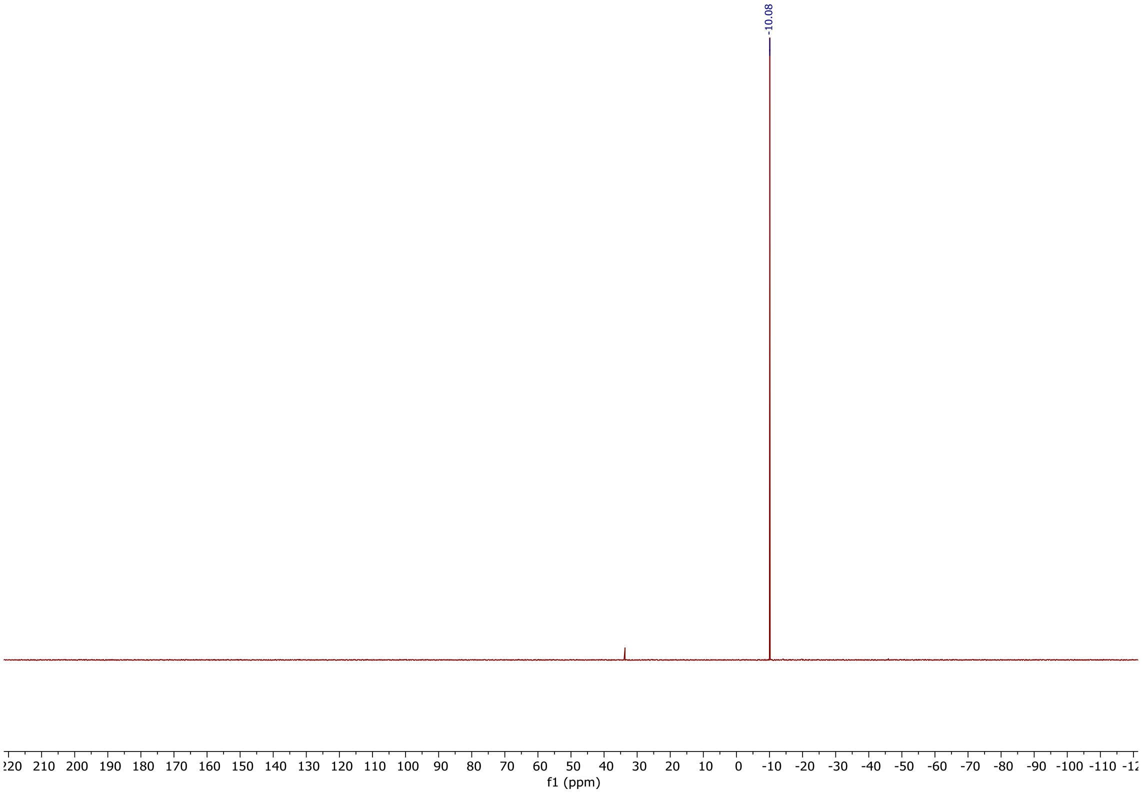

1-(3-Cyclopentylpropyl)-benzene (**6**) – ^{1}H NMR spectrum ($CDCl_3$)

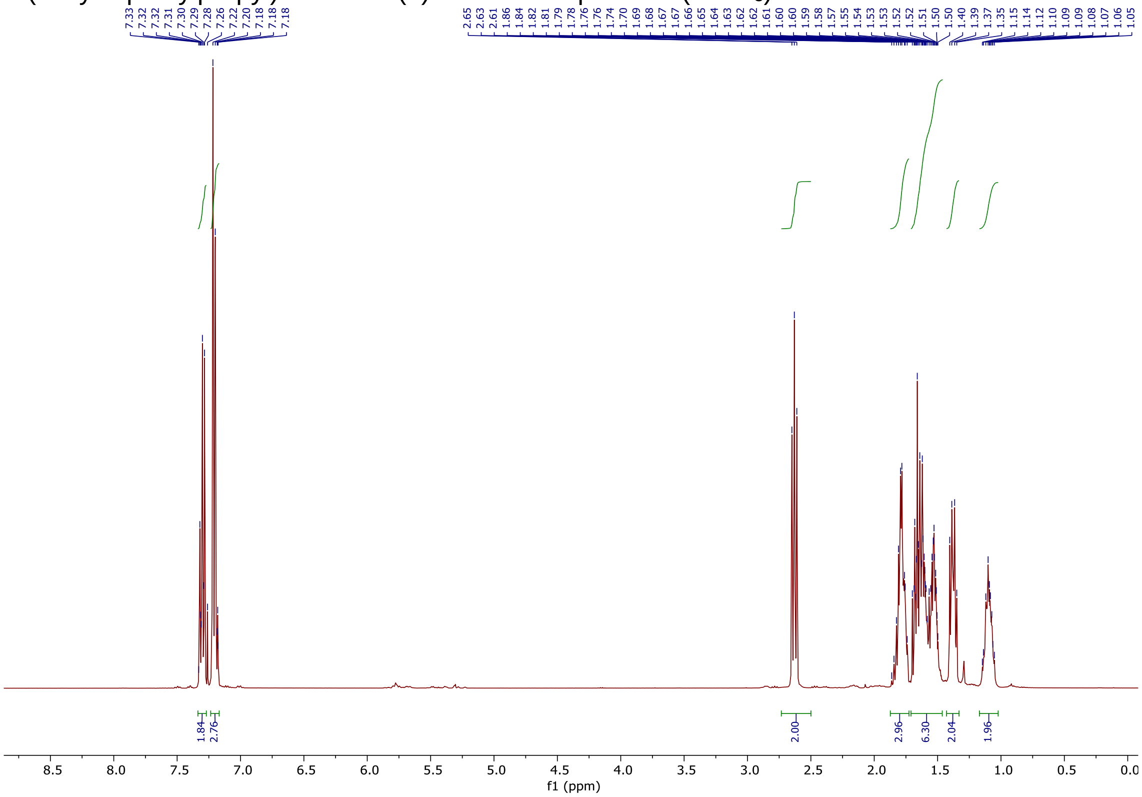

1-(3-Cyclopentylpropyl)-benzene (**6**) – $^{13}C\{^{1}H\}$ NMR spectrum ($CDCl_3$)

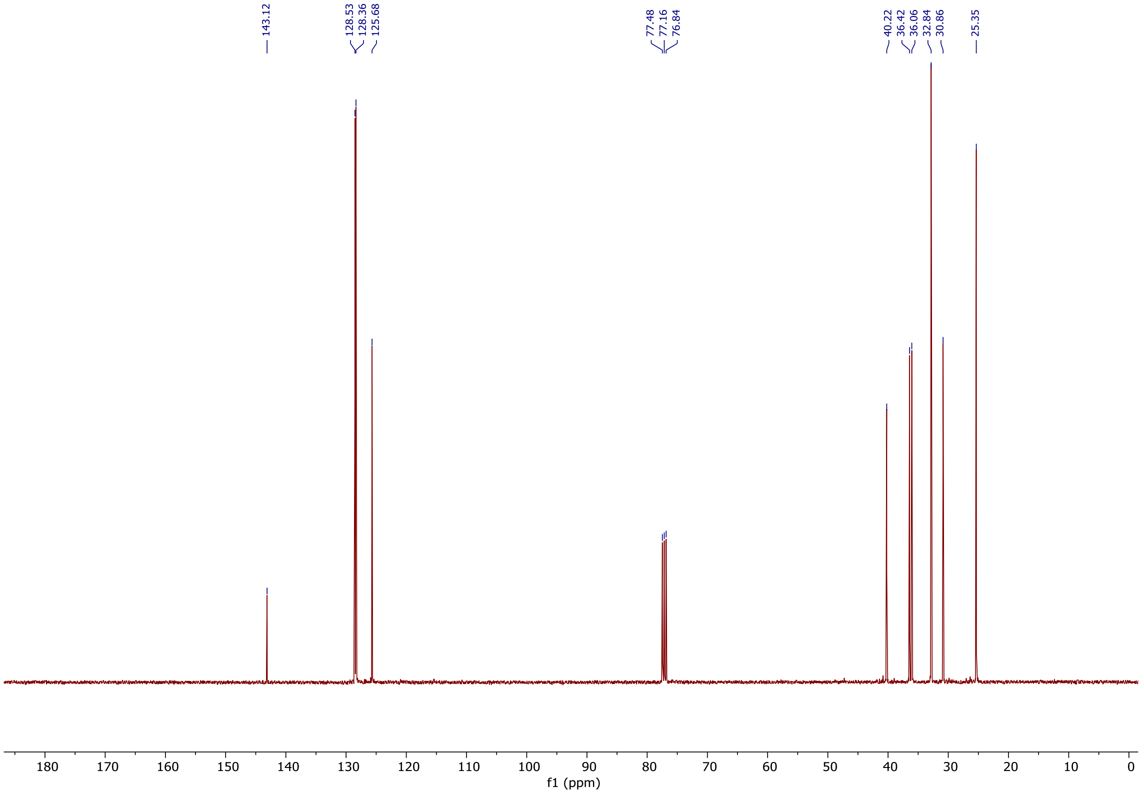

TEMPO adduct **7** – ^{1}H NMR spectrum ($CDCl_3$)

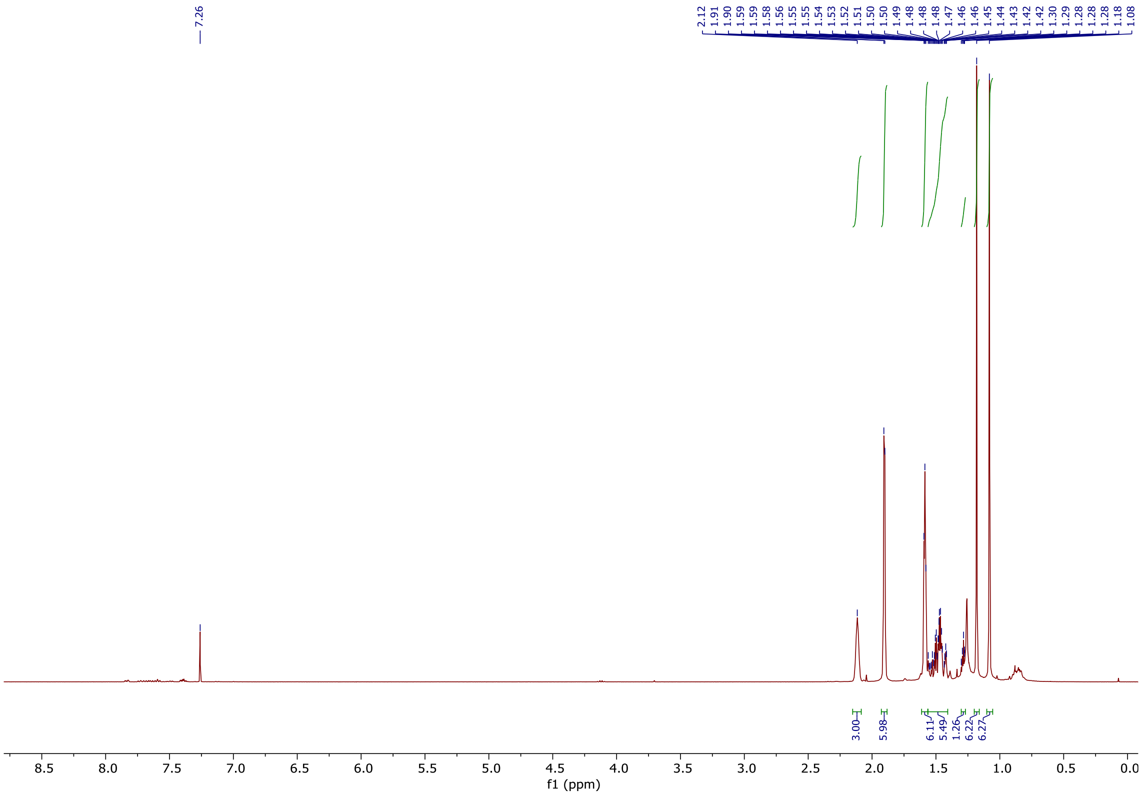

TEMPO adduct **7** – $^{13}C\{^1H\}$ NMR spectrum ($CDCl_3$)

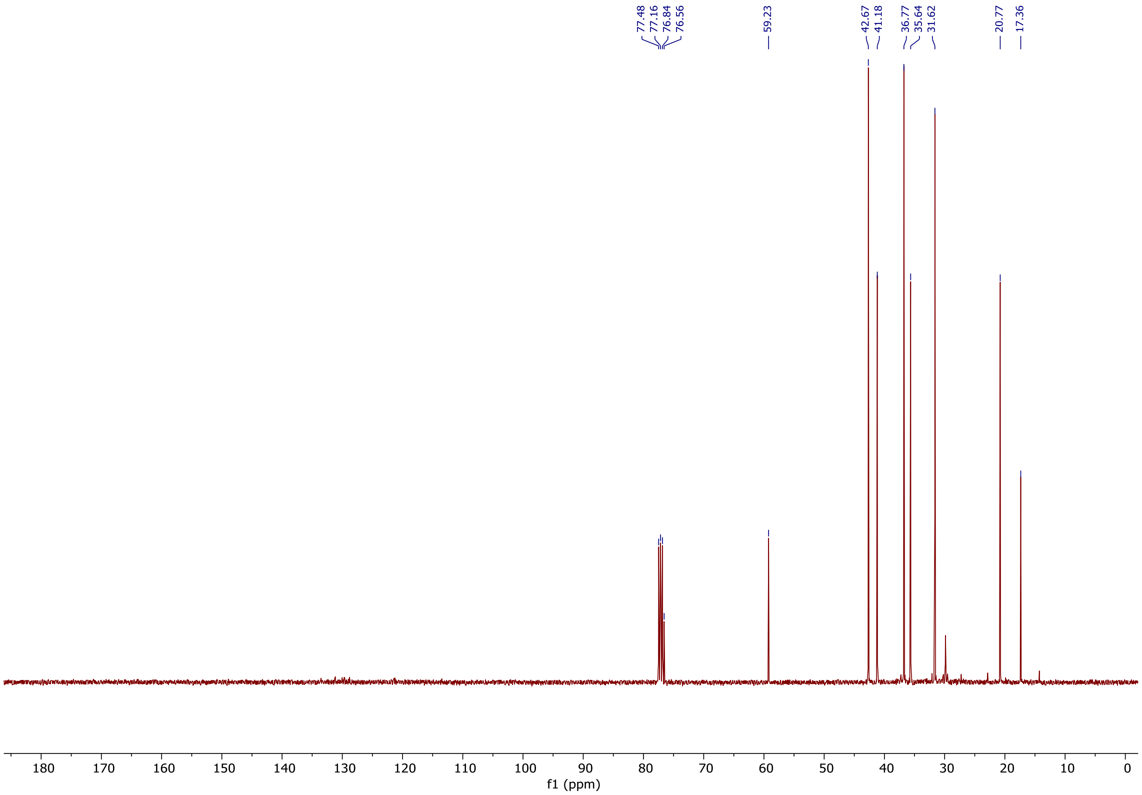

Adamantane (**8**) formed in the reaction using THF-d_8 as solvent – ^1H qNMR spectrum ($CDCl_3$)

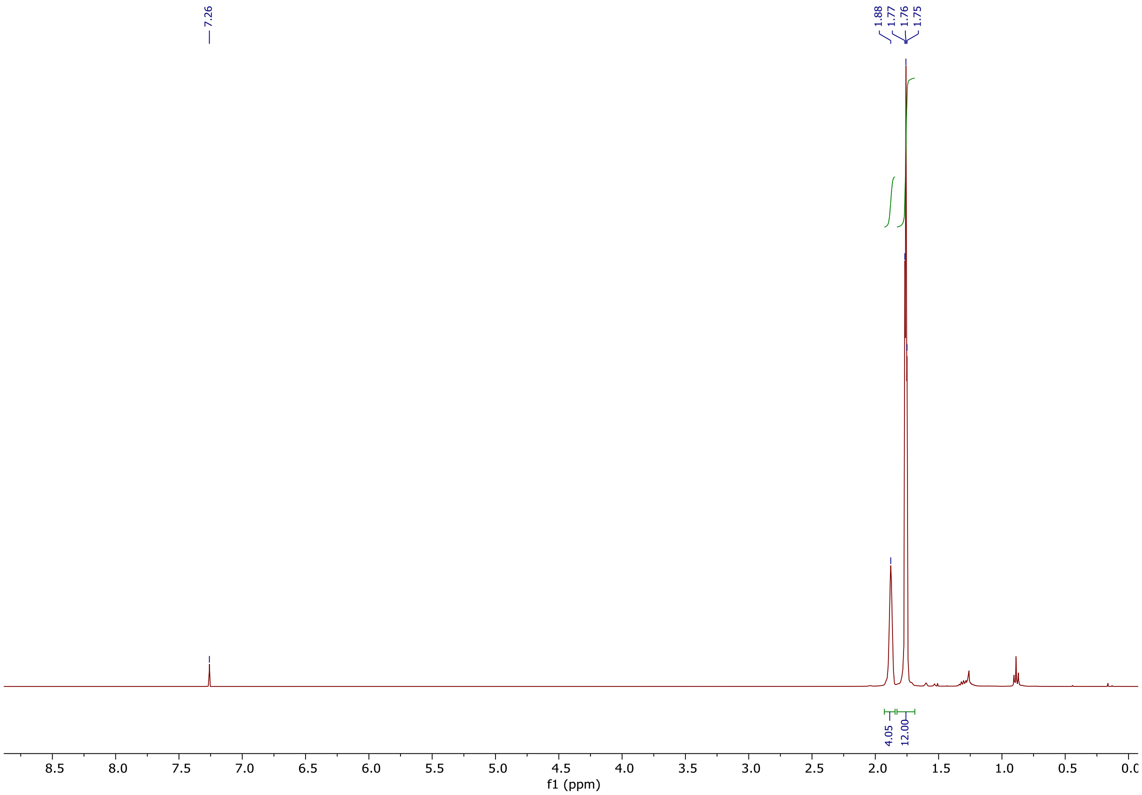

Adamantane-*d* (**8-*d***) formed in the reaction using **9-*d*$_{15}$** as substrate – ^{1}H qNMR spectrum (CDCl$_3$)

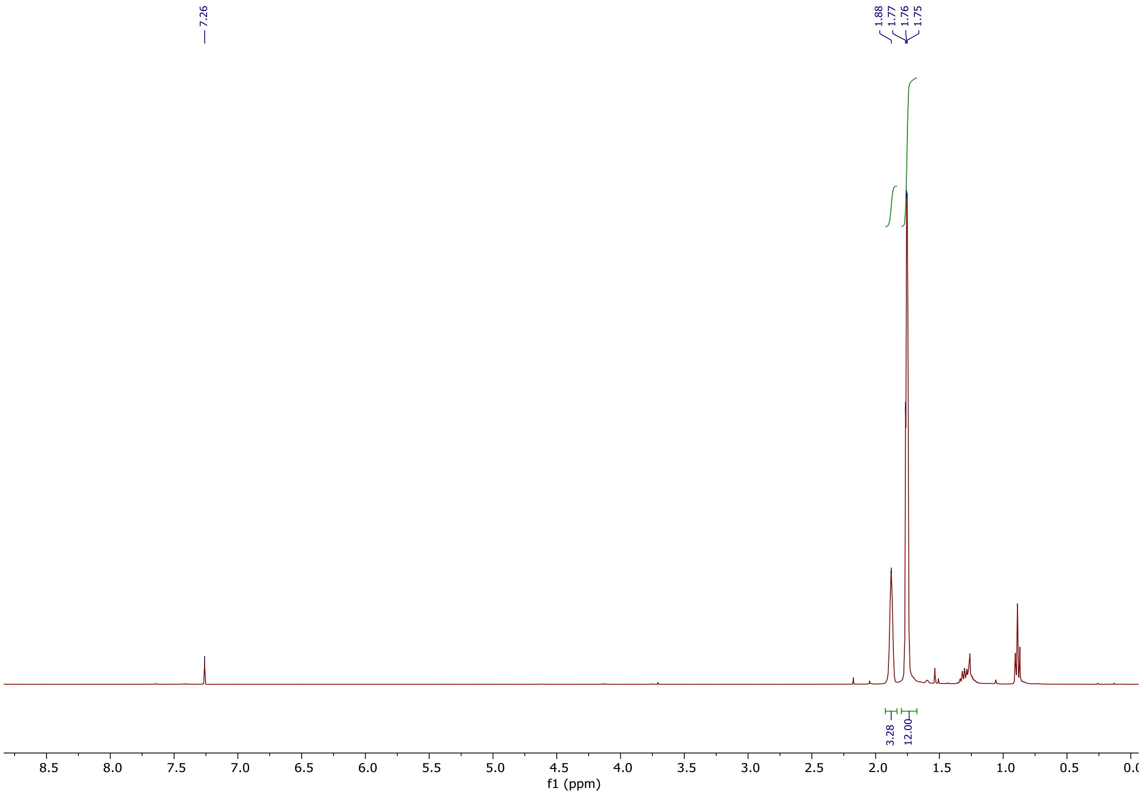

Adamantane-*d* (**8-*d***) formed in the reaction using **9-*d*$_{15}$** as substrate – ^{13}C{^{1}H} NMR spectrum (CDCl$_3$)

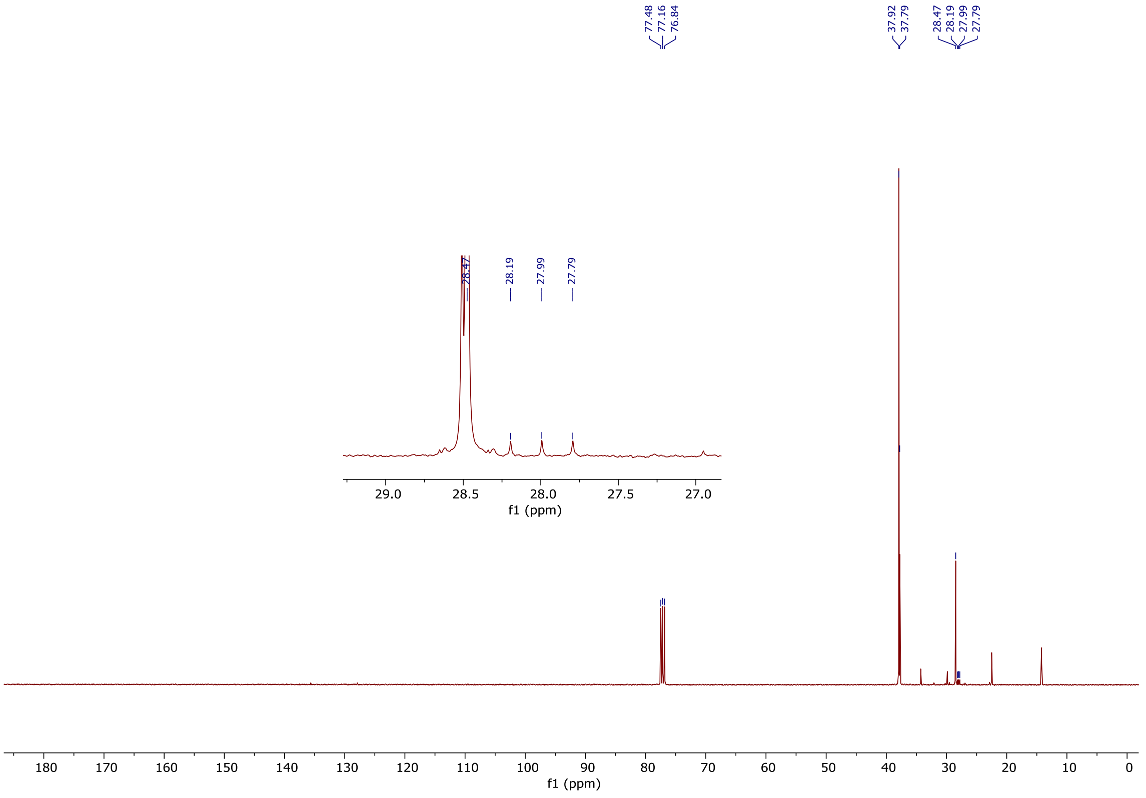

Adamant-1-yltriphenylphosphonium bromide (**9**) – ^{1}H NMR spectrum ($CDCl_3$)

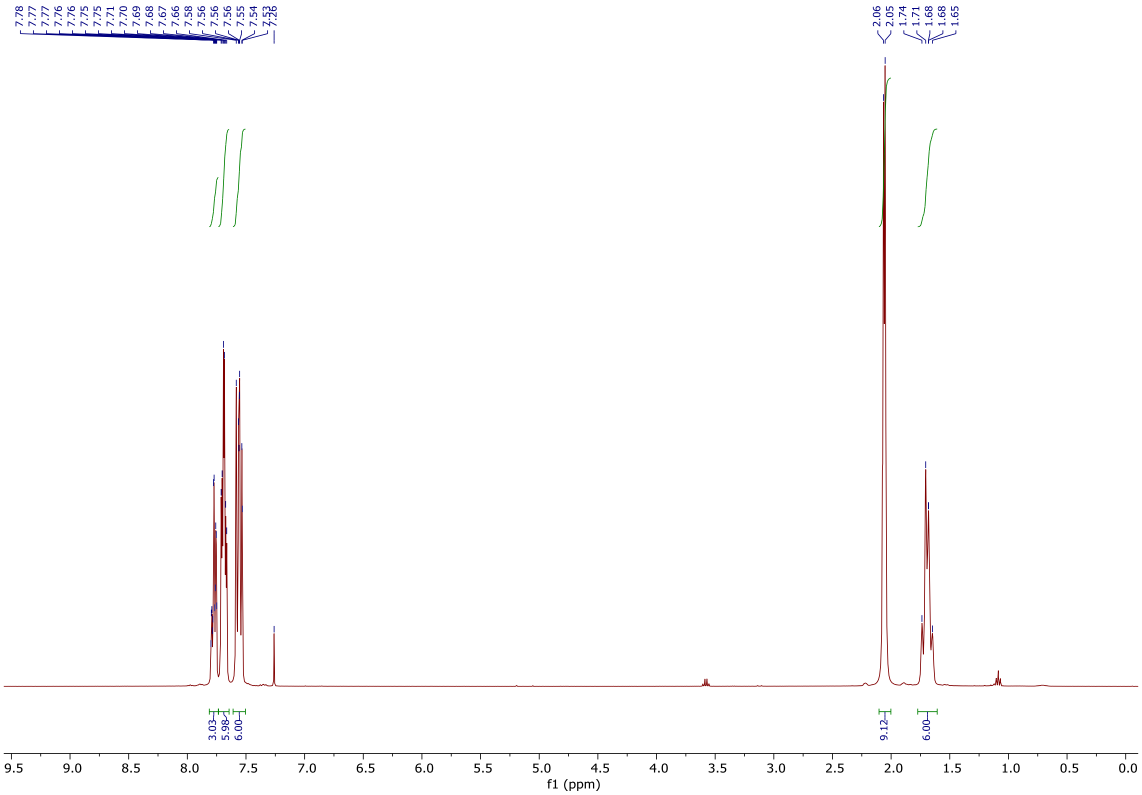

Adamant-1-yltriphenylphosphonium bromide (**9**) – $^{13}C\{^{1}H\}$ NMR spectrum ($CDCl_3$)

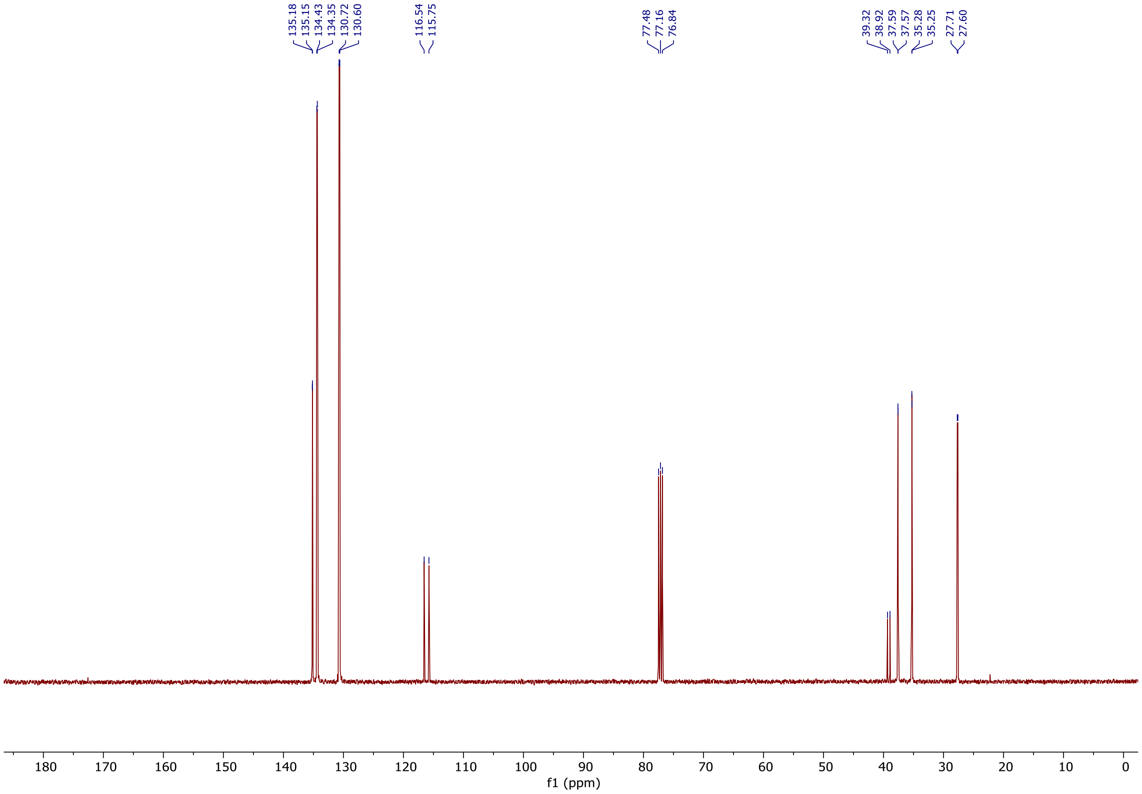

Adamant-1-yltriphenylphosphonium bromide (**9**) – $^{31}P\{^{1}H\}$ NMR spectrum ($CDCl_3$)

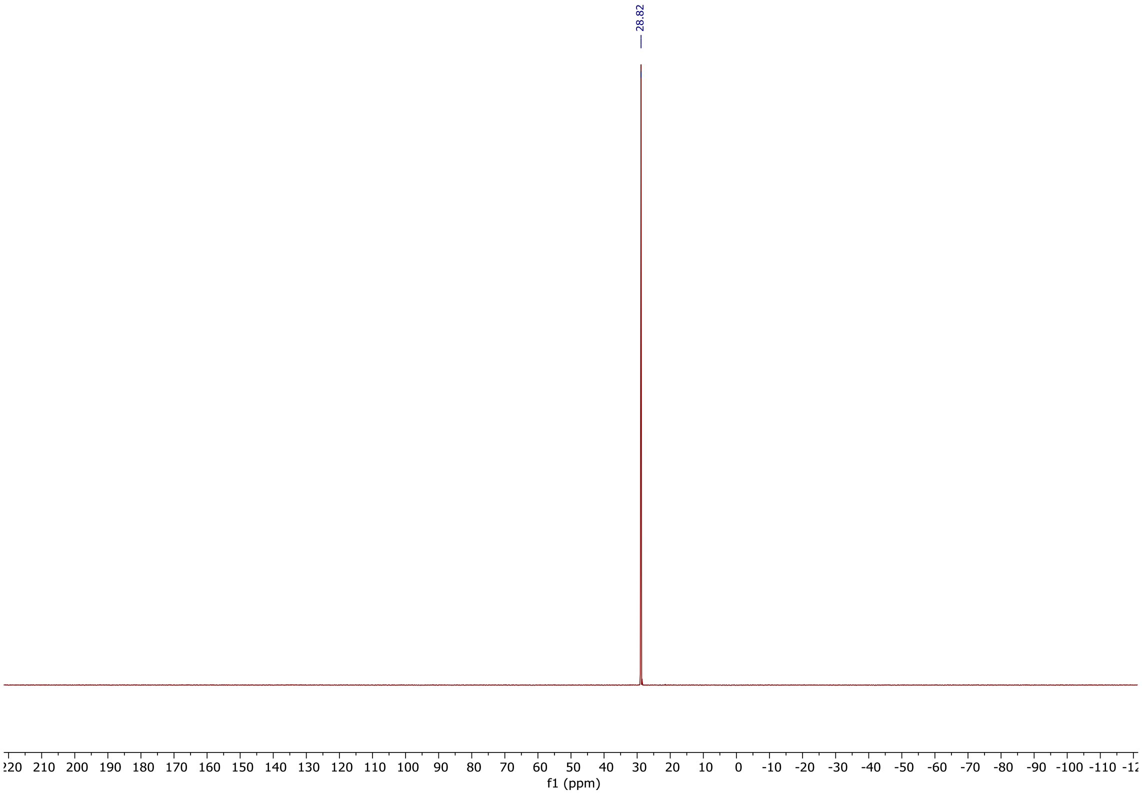

Adamant-1-yltris(phenyl-*d*$_5$)phosphonium bromide (**9-*d*$_{15}$**) – ^{1}H NMR spectrum ($CDCl_3$)

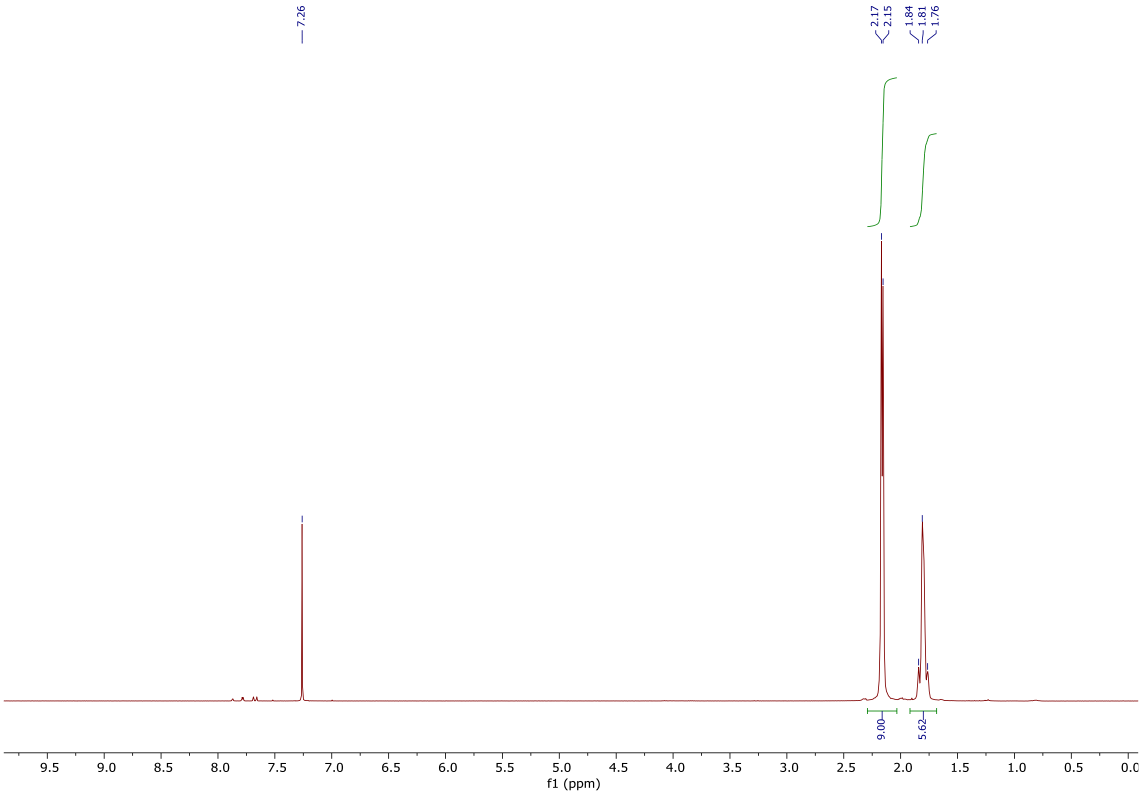

Adamant-1-yltris(phenyl-d_5)phosphonium bromide (**9-*d*$_{15}$**) – $^{13}C\{^{1}H\}$ NMR spectrum ($CDCl_3$)

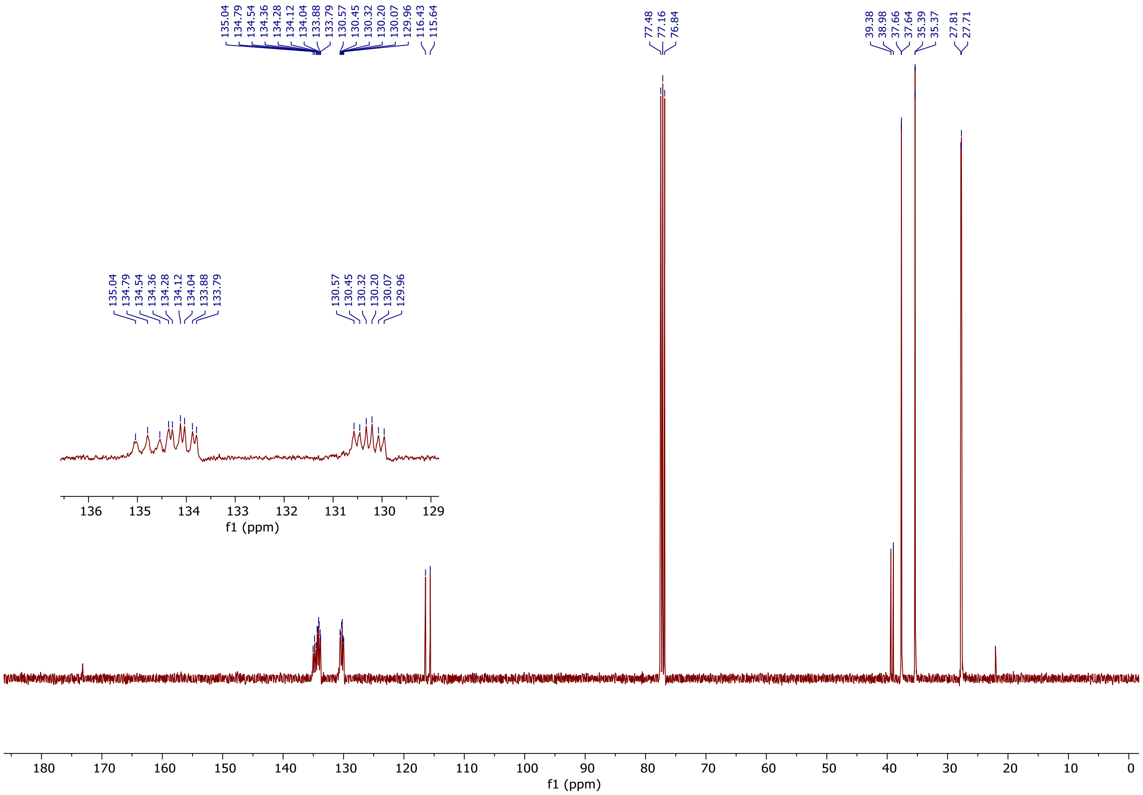

Adamant-1-yltris(phenyl-d_5)phosphonium bromide (**9-*d*$_{15}$**) – $^{31}P\{^{1}H\}$ NMR spectrum ($CDCl_3$)

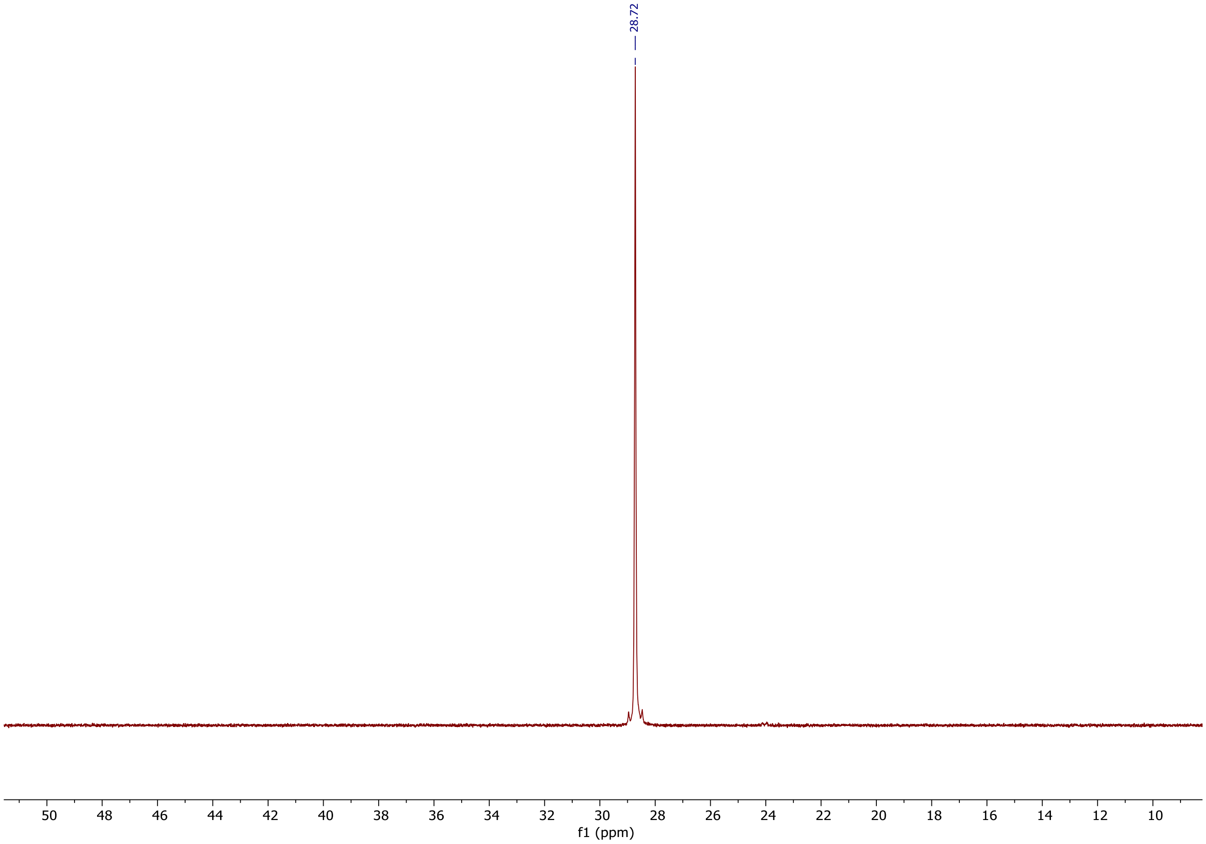

Cyclobutyltriphenylphosphonium bromide (**10v**) – ^{1}H NMR spectrum ($CDCl_3$)

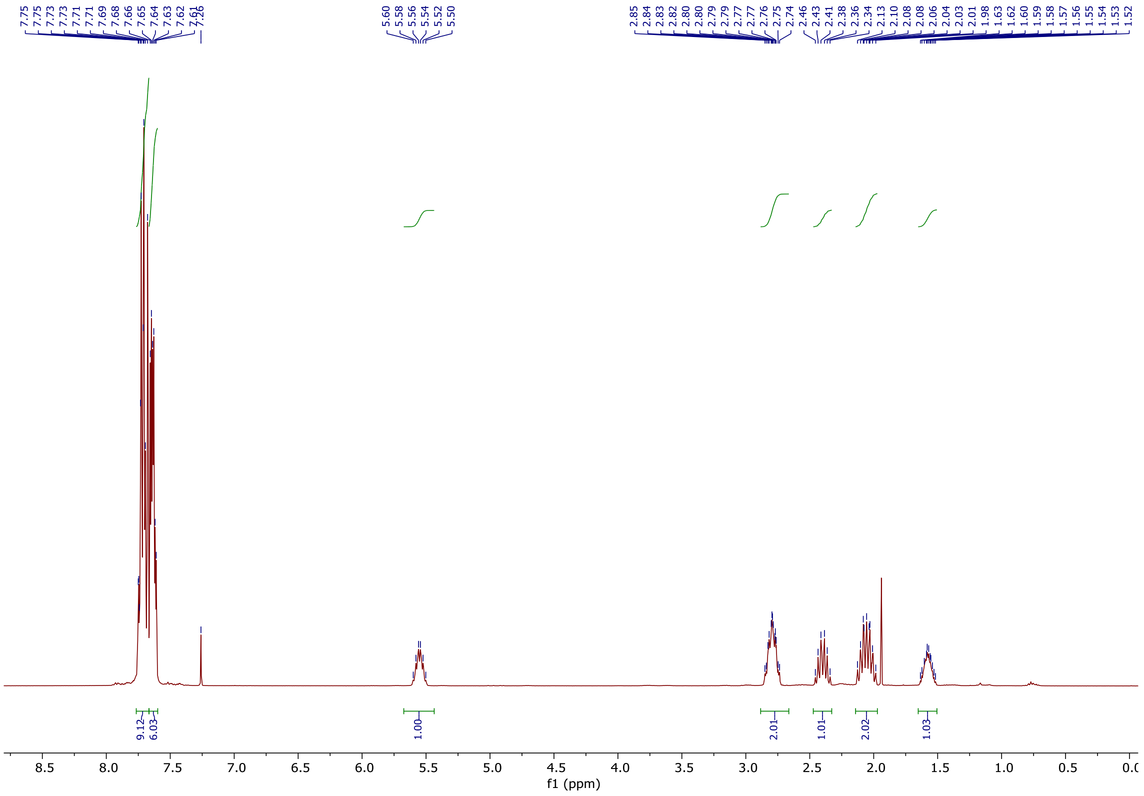

Cyclobutyltriphenylphosphonium bromide (**10v**) – $^{13}C\{^1H\}$ NMR spectrum ($CDCl_3$)

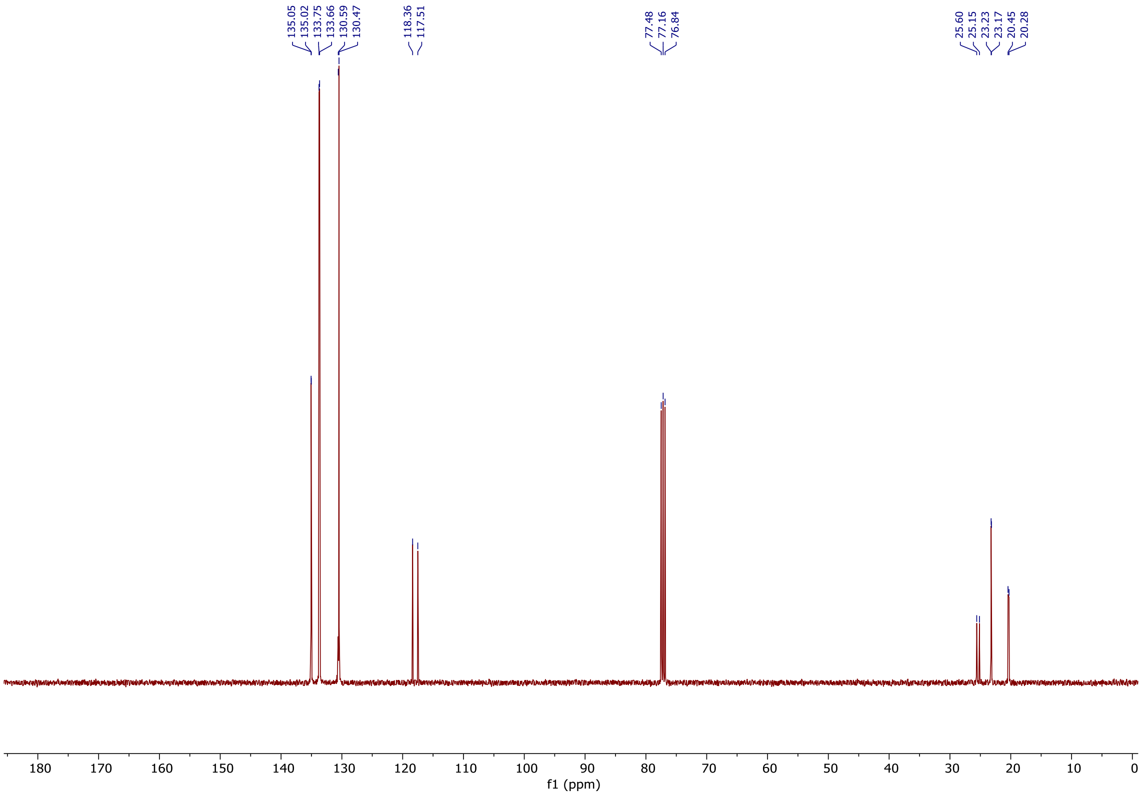

Cyclobutyltriphenylphosphonium bromide (**10v**) – $^{31}P\{^{1}H\}$ NMR spectrum ($CDCl_3$)

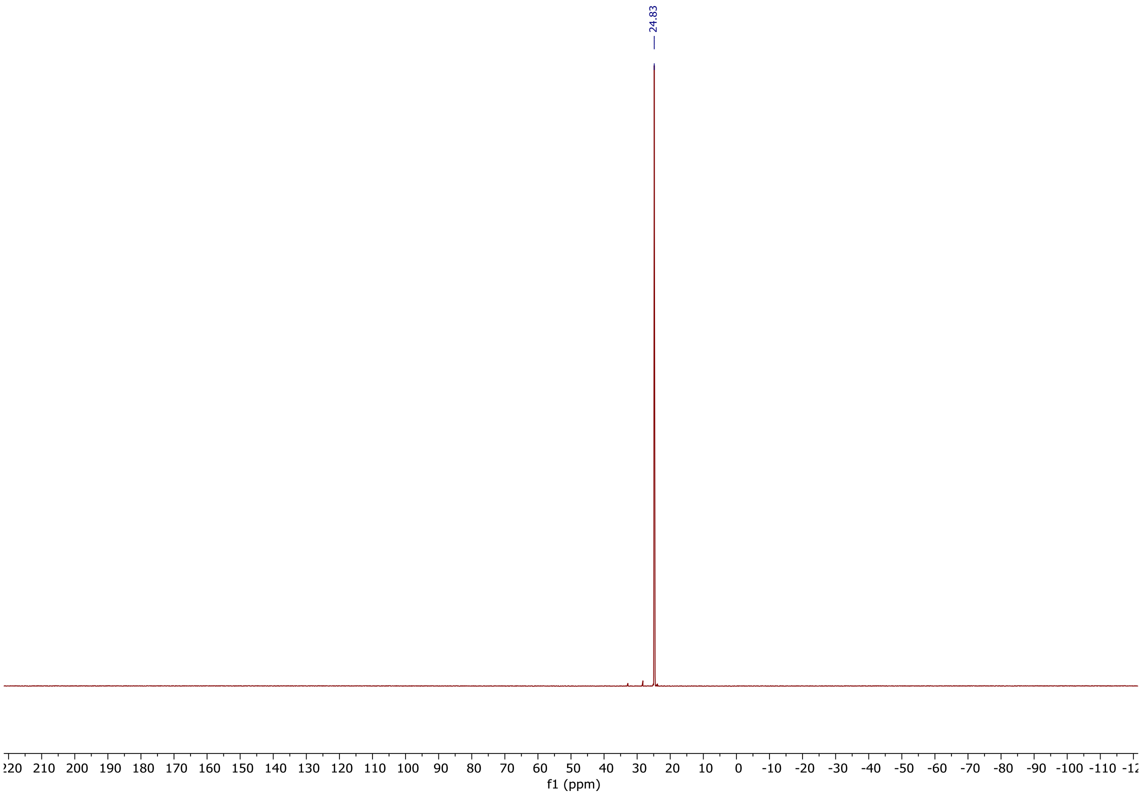

(2-Norbornyl)triphenylphosphonium bromide (**10w**) – ^{1}H NMR spectrum ($CDCl_3$)

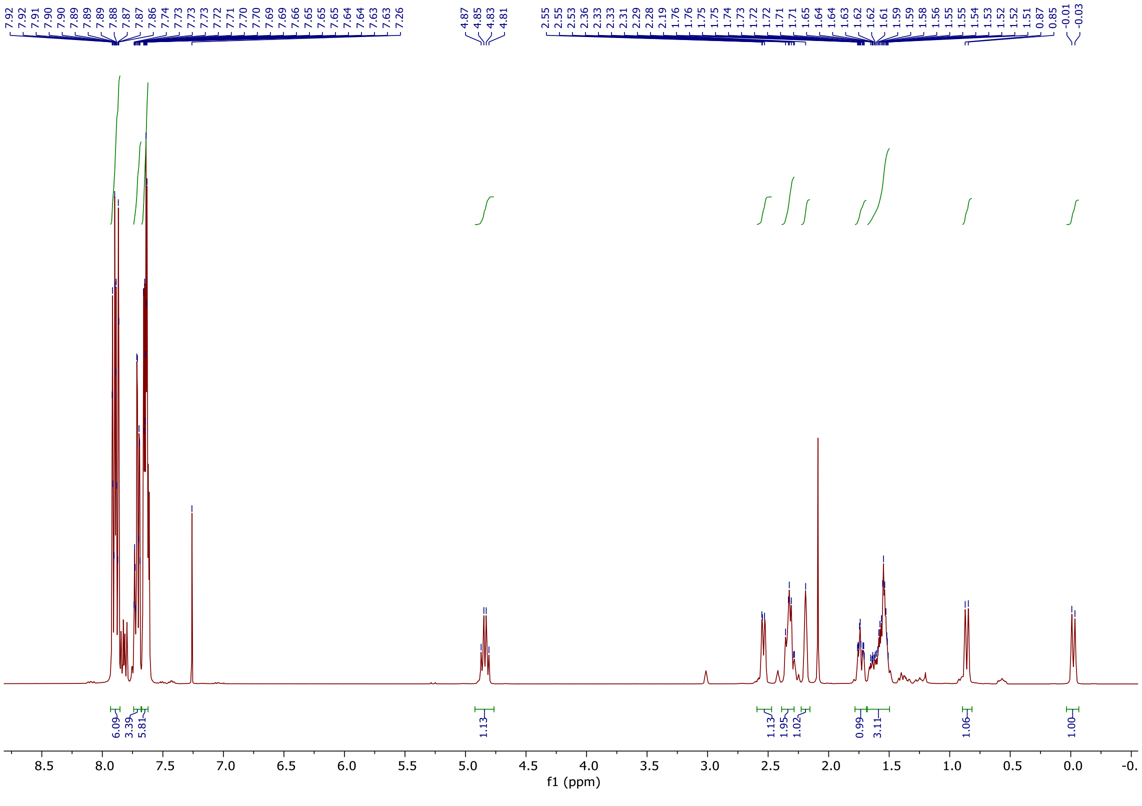

(2-Norbornyl)triphenylphosphonium bromide (**10w**) – $^{13}C\{^1H\}$ NMR spectrum ($CDCl_3$)

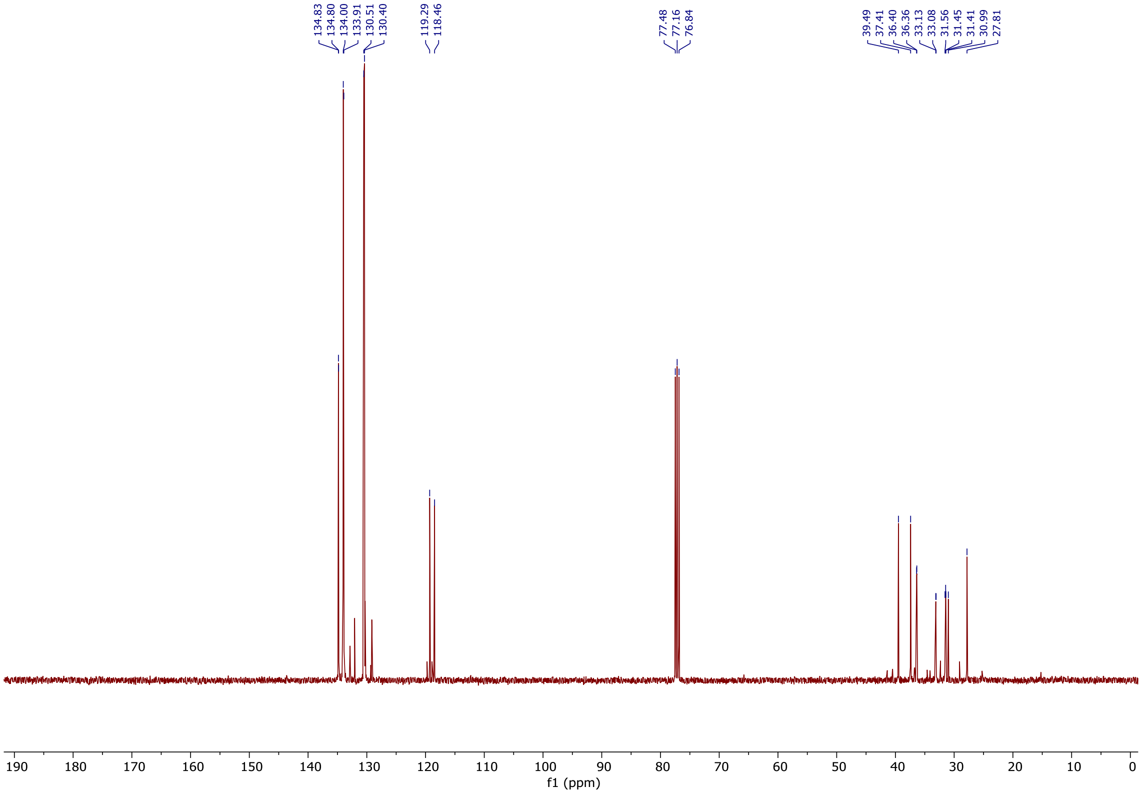

(2-Norbornyl)triphenylphosphonium bromide (**10w**) – $^{31}P\{^1H\}$ qNMR spectrum ($CDCl_3$)

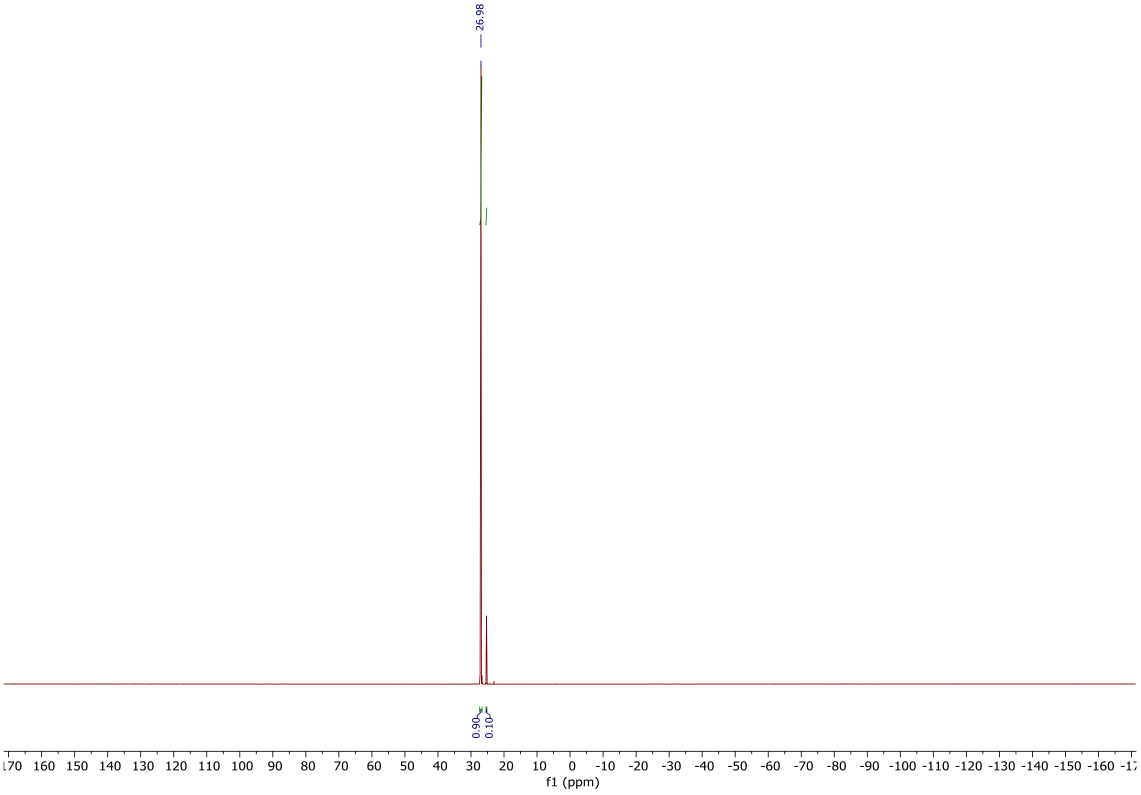

(2-Norbornyl)triphenylphosphonium bromide (**10w**) – COSY NMR spectrum ($CDCl_3$)

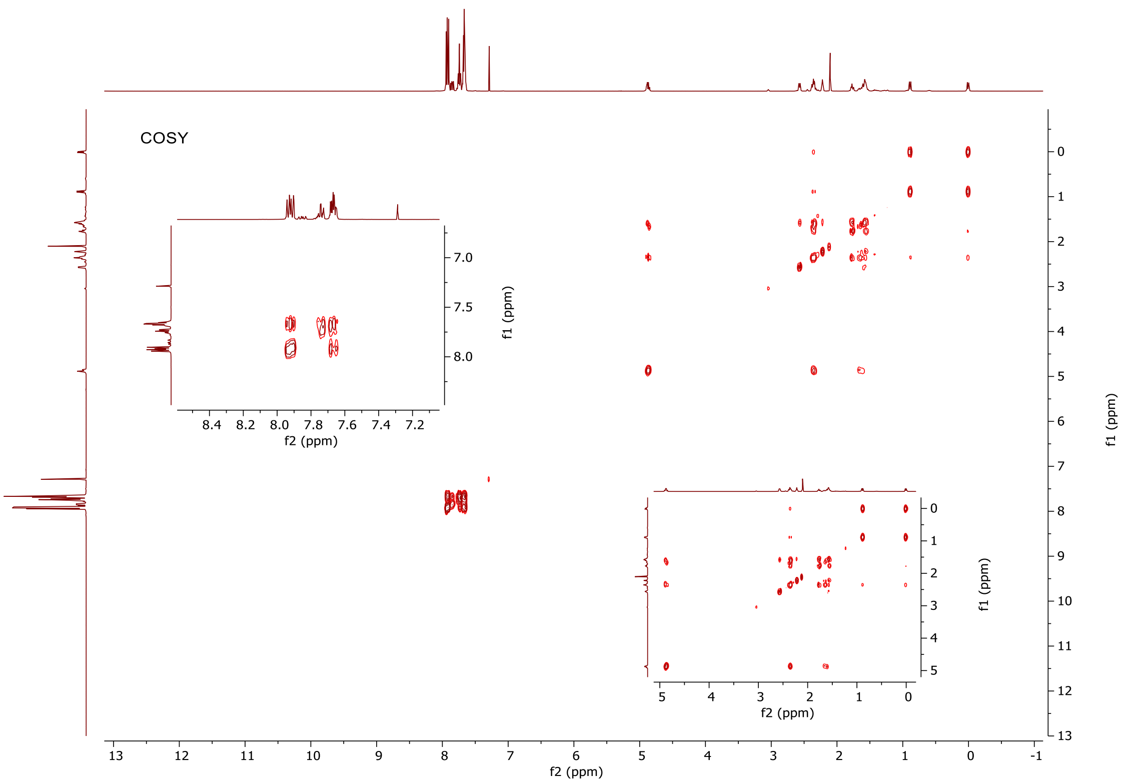

(2-Norbornyl)triphenylphosphonium bromide (**10w**) – HSQC NMR spectrum ($CDCl_3$)

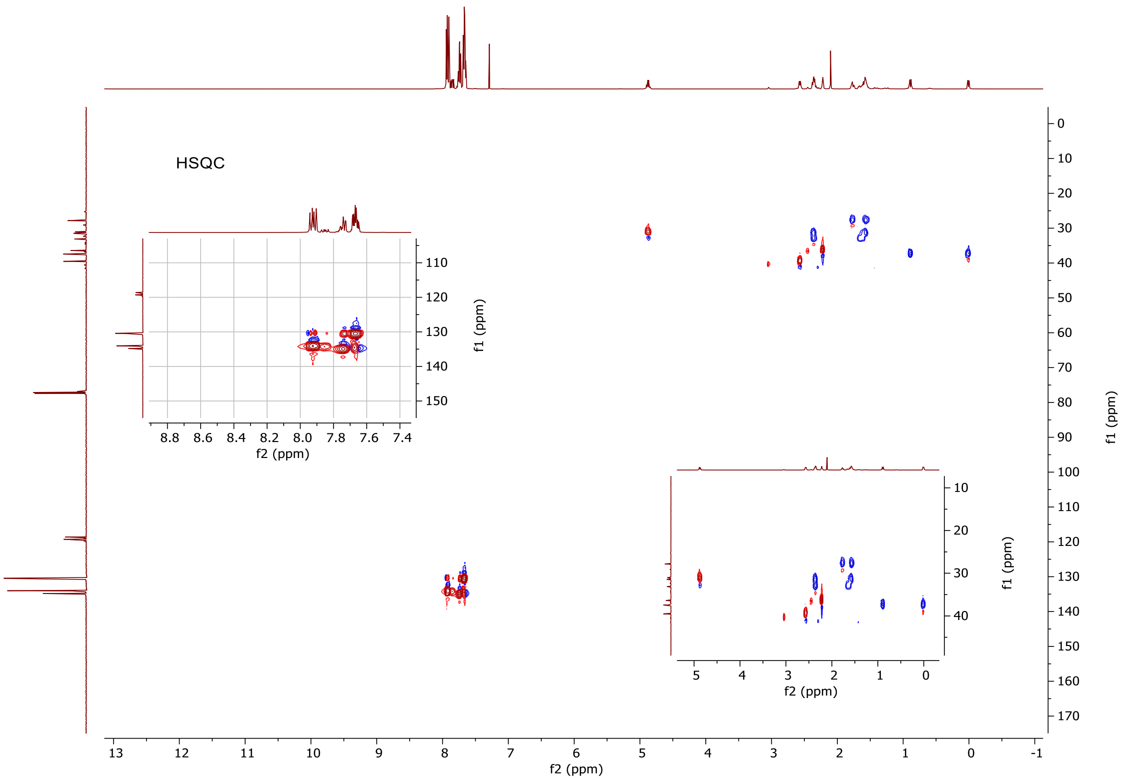

(2-Norbornyl)triphenylphosphonium bromide (**10w**) – HMBC NMR spectrum ($CDCl_3$)

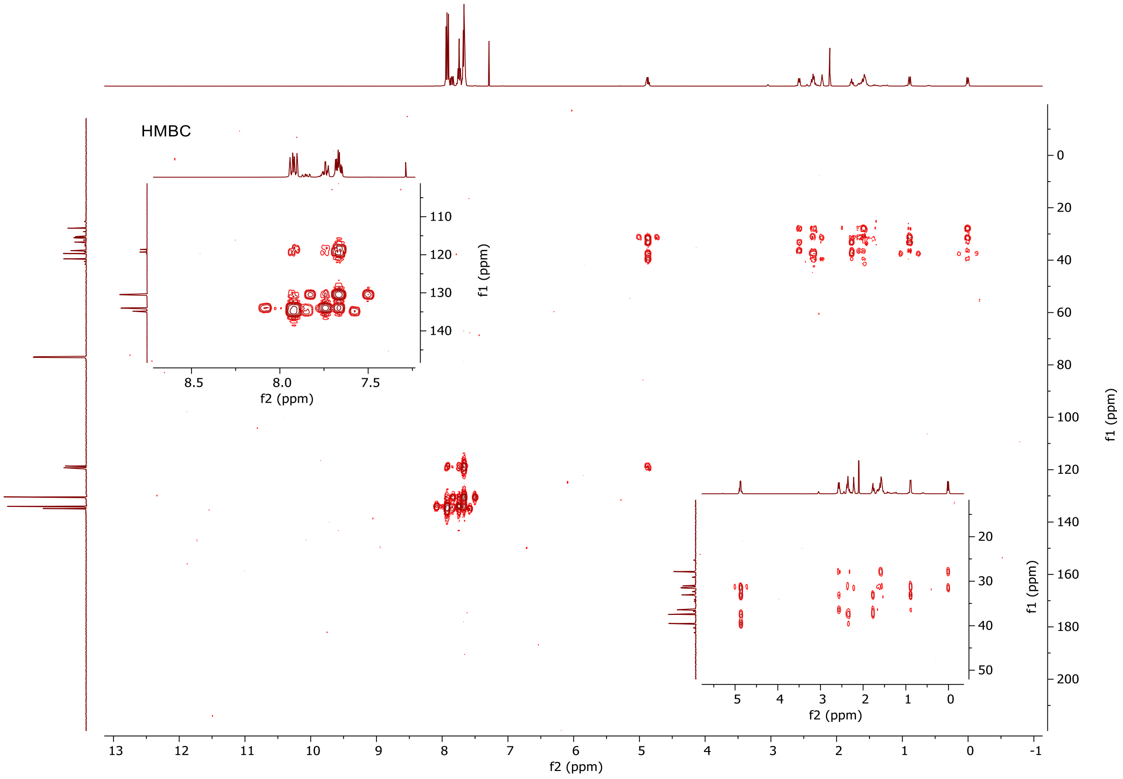

Cycloheptyltriphenylphosphonium bromide (**10y**) – ^1H NMR spectrum ($CDCl_3$)

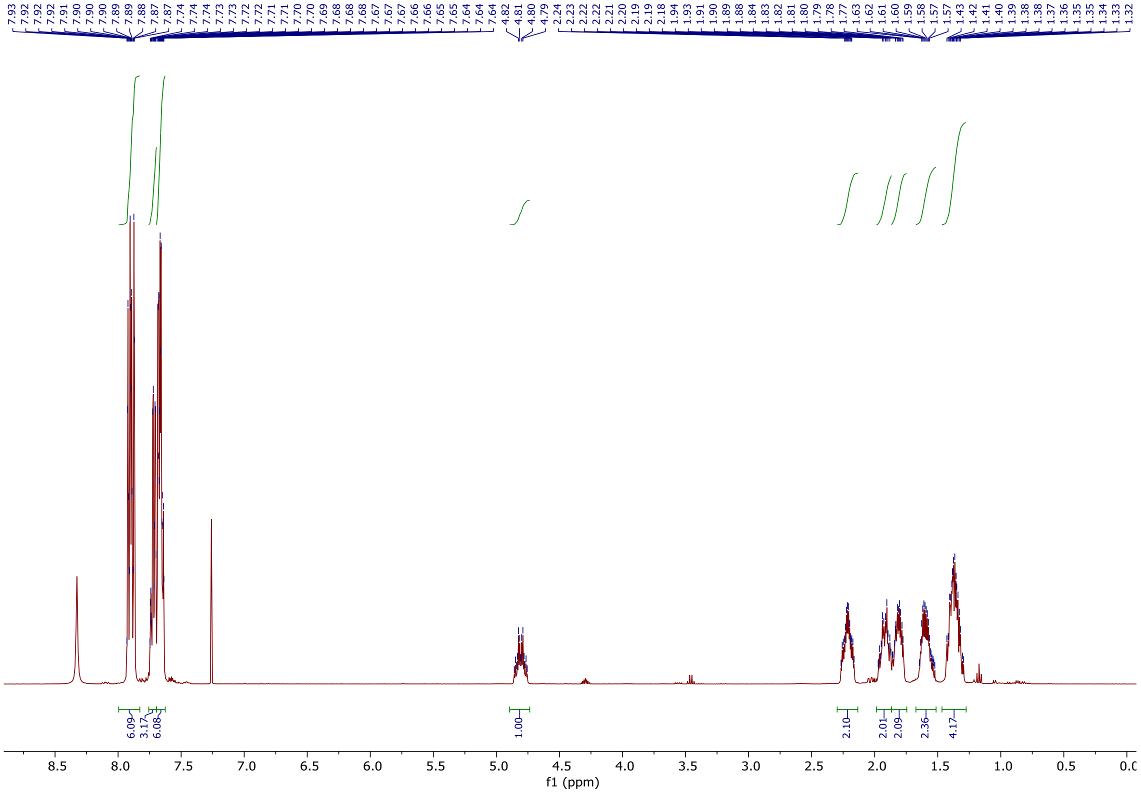

Cycloheptyltriphenylphosphonium bromide (**10y**) – $^{13}C\{^1H\}$ NMR spectrum ($CDCl_3$)

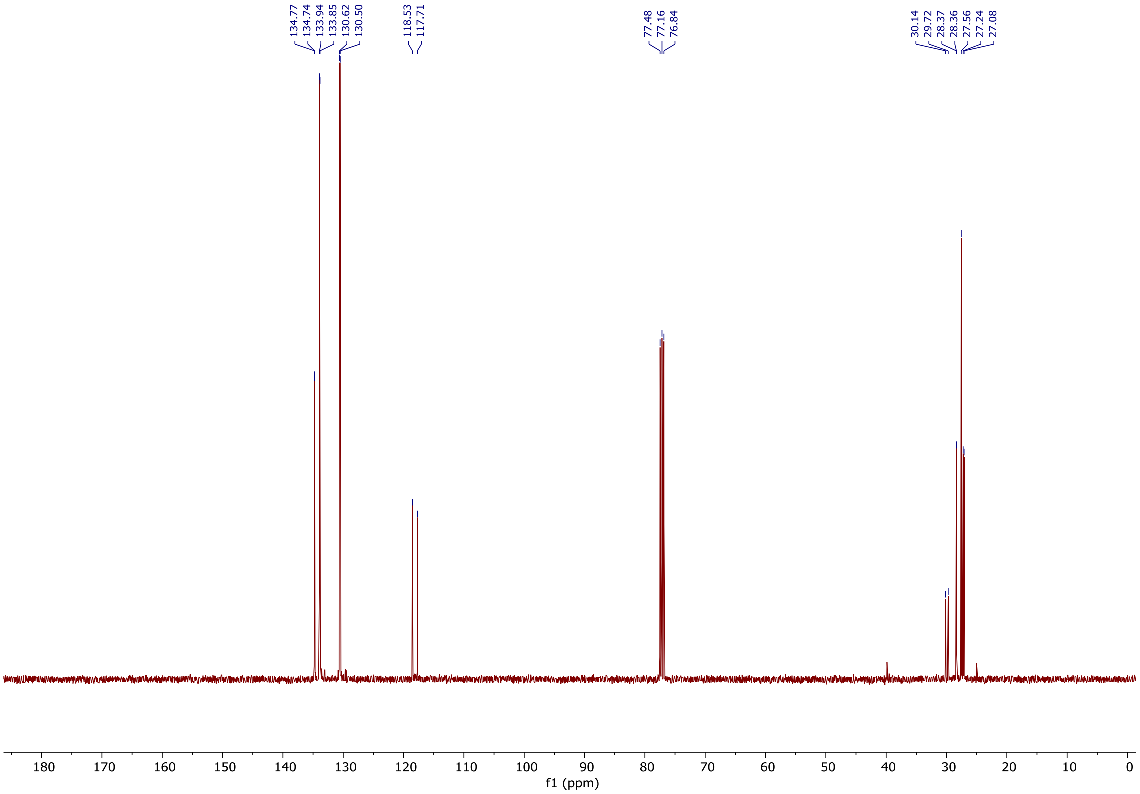

Cycloheptyltriphenylphosphonium bromide (**10y**) – $^{31}P\{^1H\}$ NMR spectrum ($CDCl_3$)

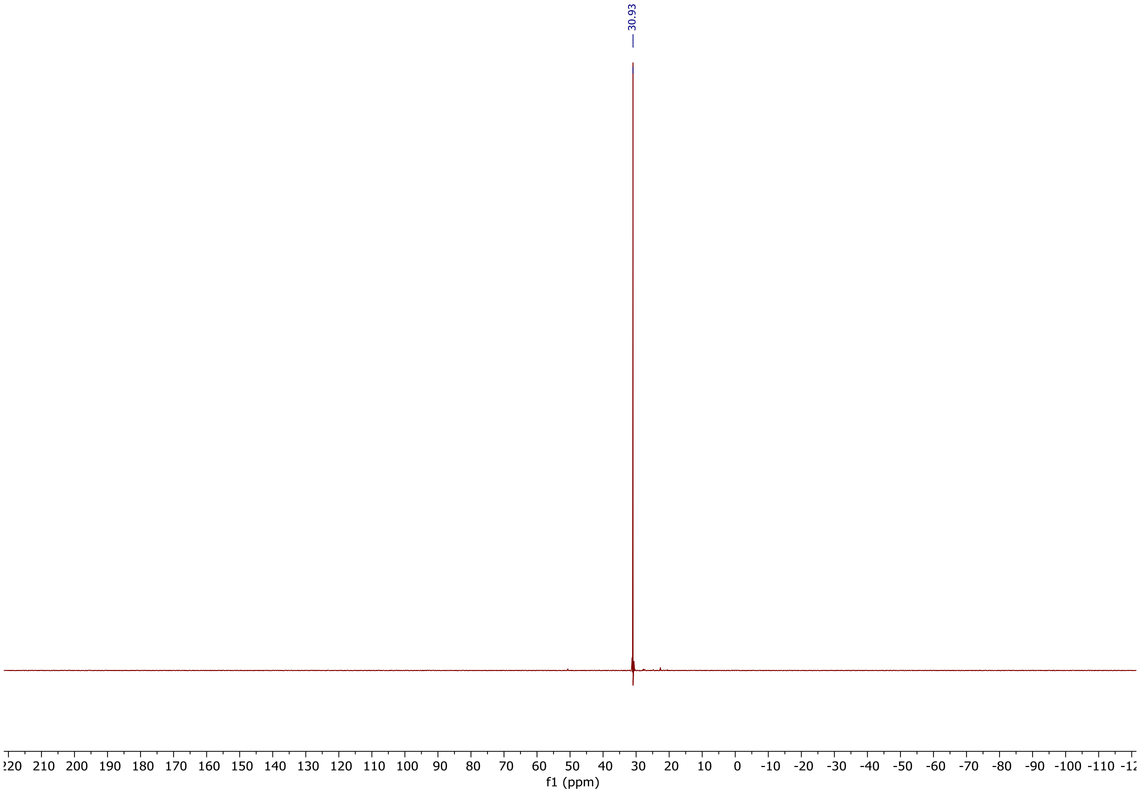

Triphenyl(tetrahydro-2H-pyran-4-yl)phosphonium bromide (**10z**) – ^{1}H NMR spectrum ($CDCl_3$)

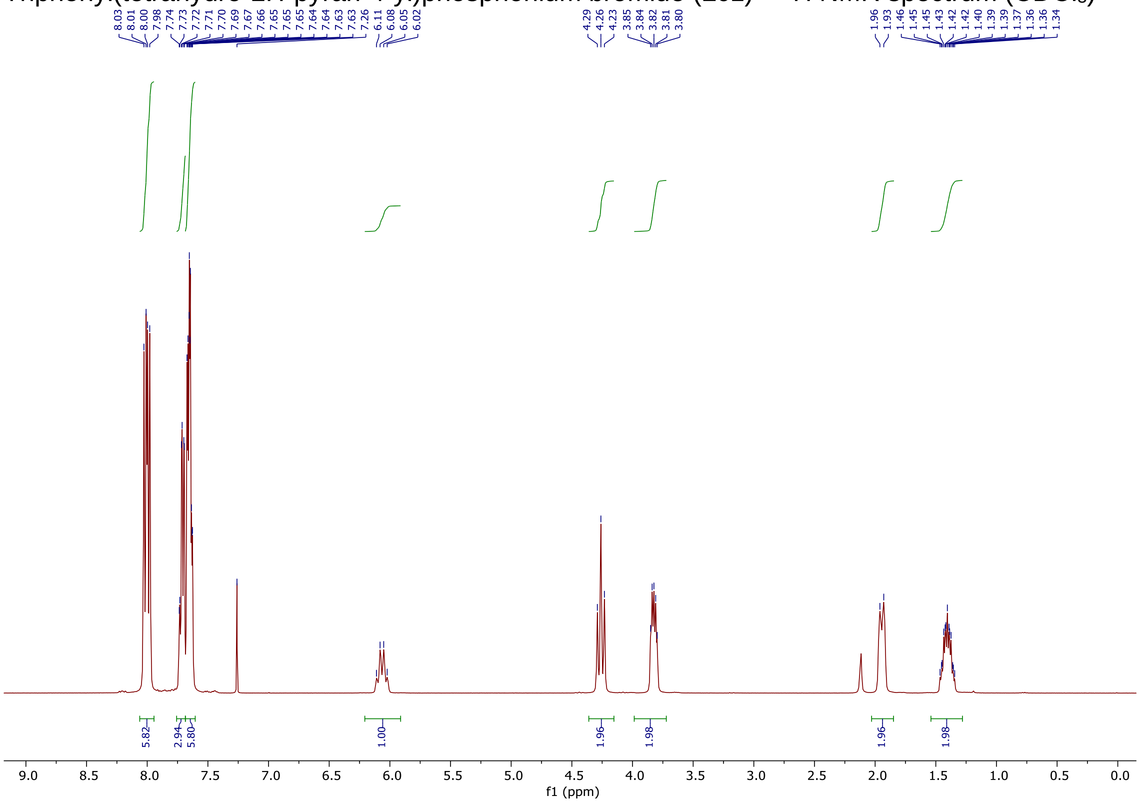

Triphenyl(tetrahydro-2H-pyran-4-yl)phosphonium bromide (**10z**) – ^{13}C{^{1}H} NMR spectrum ($CDCl_3$)

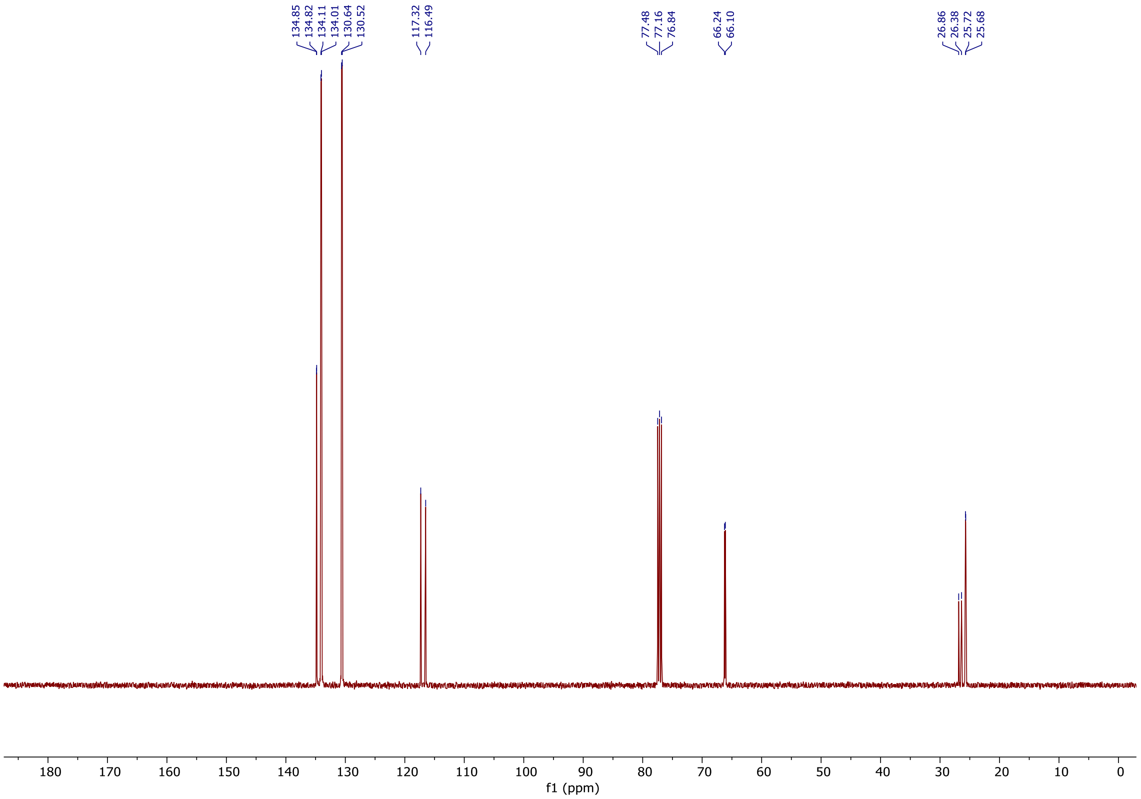

Triphenyl(tetrahydro-2H-pyran-4-yl)phosphonium bromide (**10z**) – $^{31}P\{^{1}H\}$ NMR spectrum ($CDCl_3$)

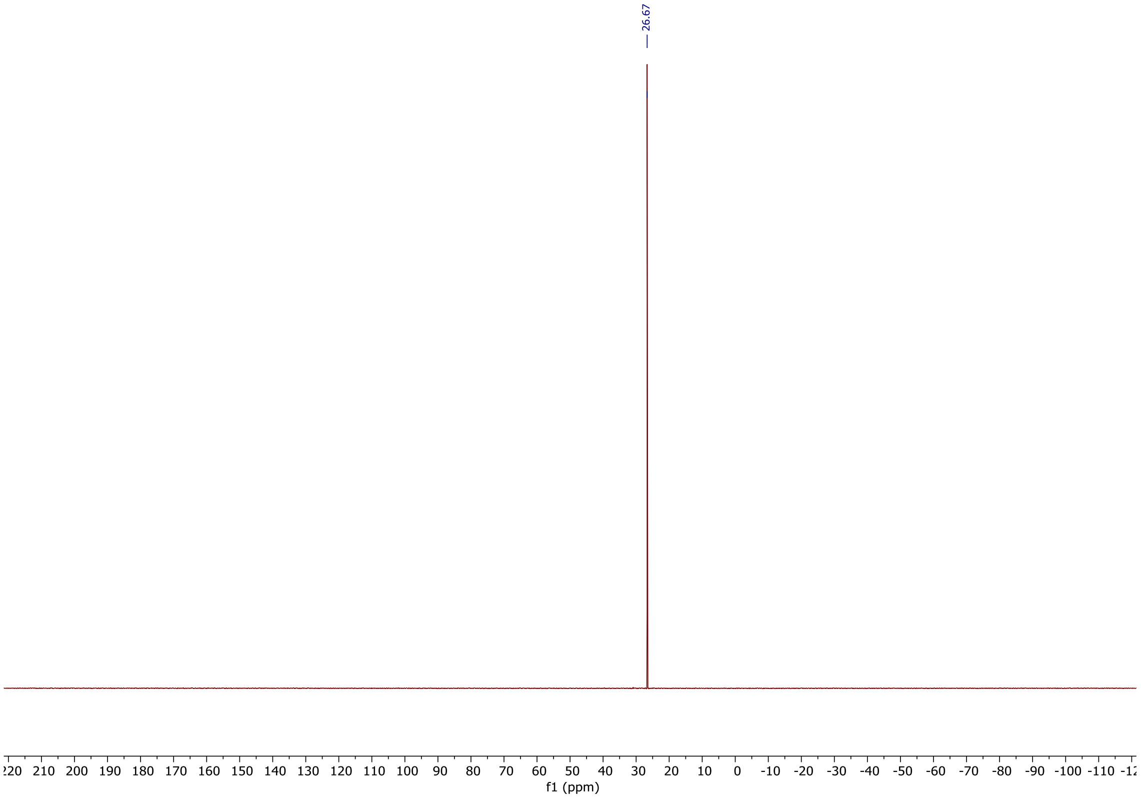

Triphenyl(5-phenylpentan-2-yl)phosphonium iodide (**10ab**) – ^{1}H NMR spectrum ($CDCl_3$)

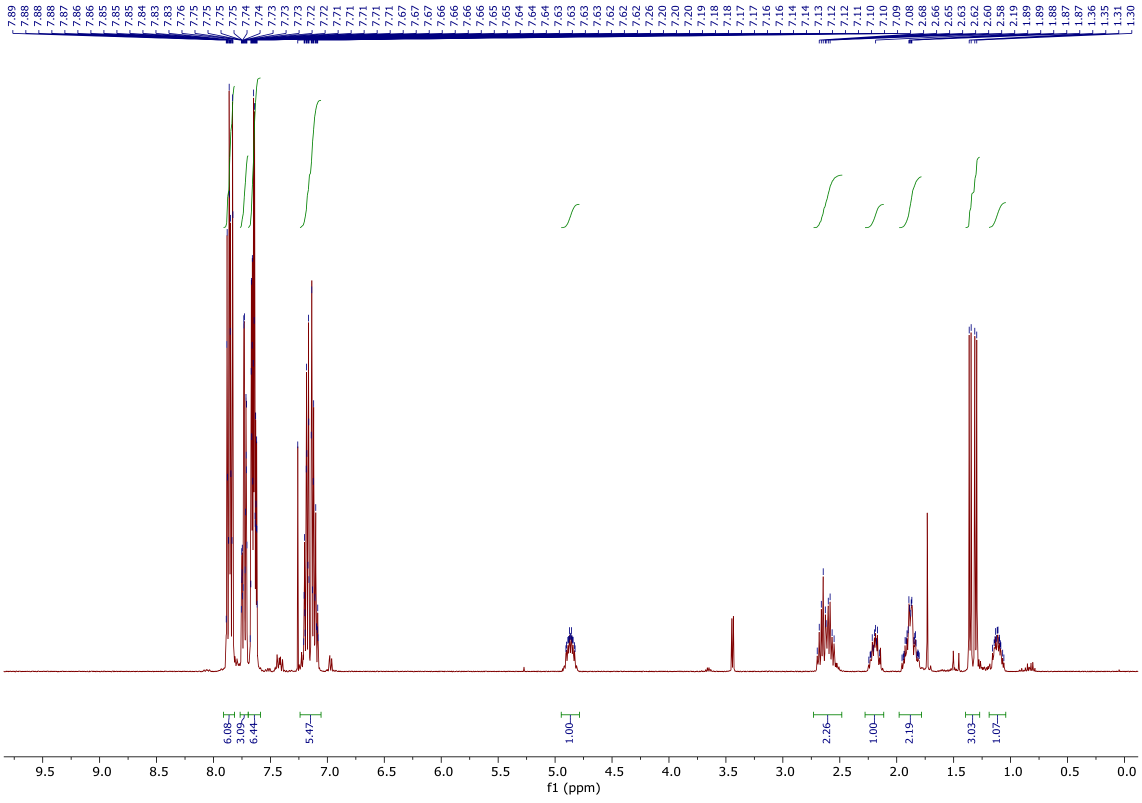

Triphenyl(5-phenylpentan-2-yl)phosphonium iodide (**10ab**) – $^{13}C\{^1H\}$ NMR spectrum ($CDCl_3$)

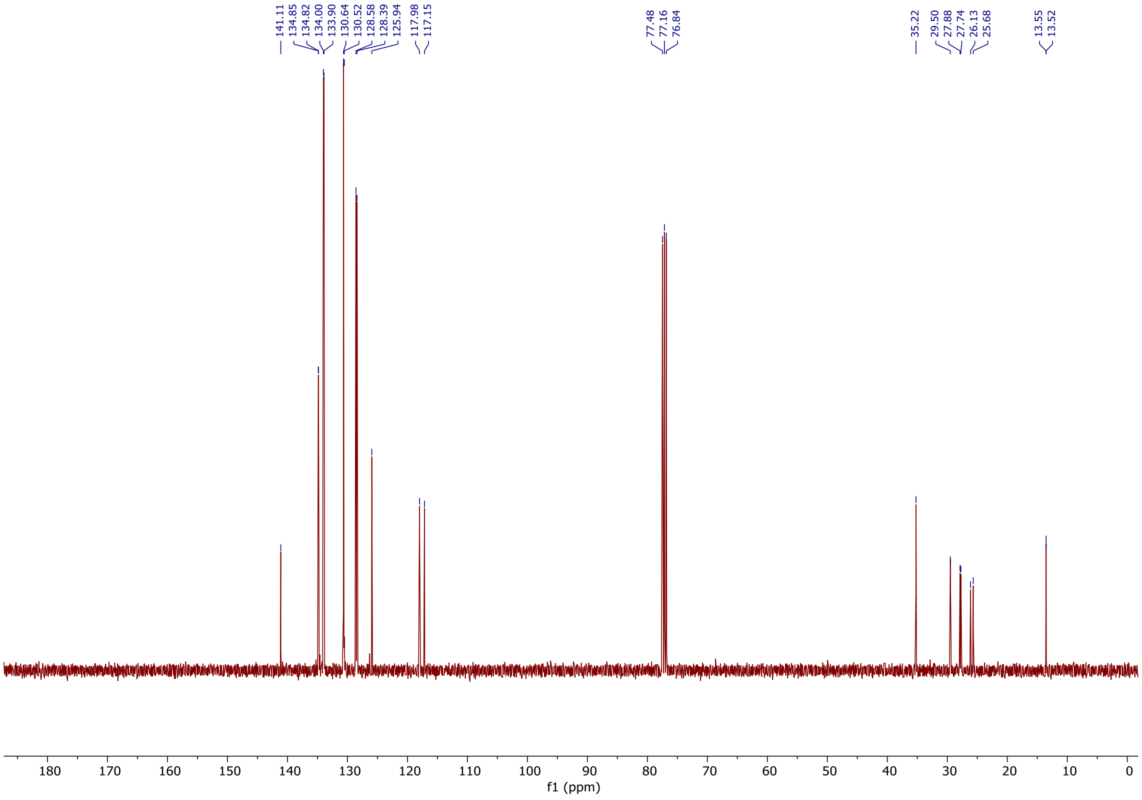

Triphenyl(5-phenylpentan-2-yl)phosphonium iodide (**10ab**) – $^{31}P\{^1H\}$ NMR spectrum ($CDCl_3$)

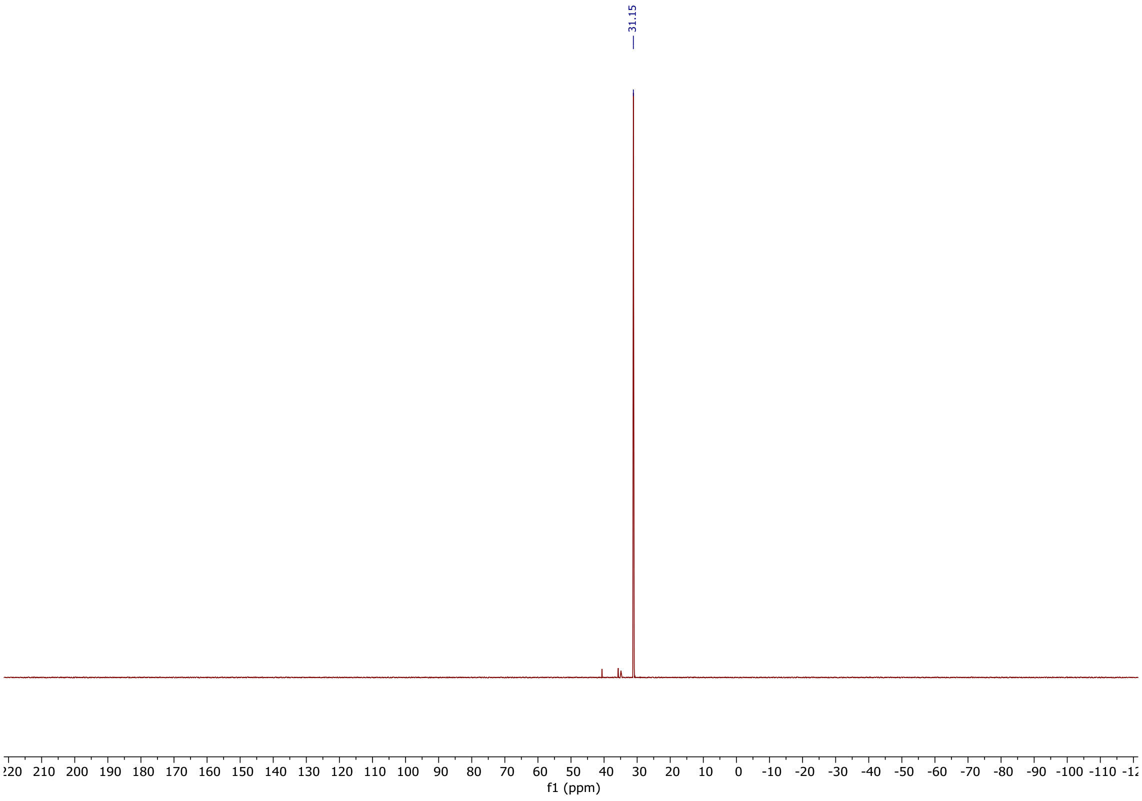

(5-(4-Bromophenyl)pentan-2-yl)triphenylphosphonium iodide (**10ac**) – ^{1}H NMR spectrum ($CDCl_3$)

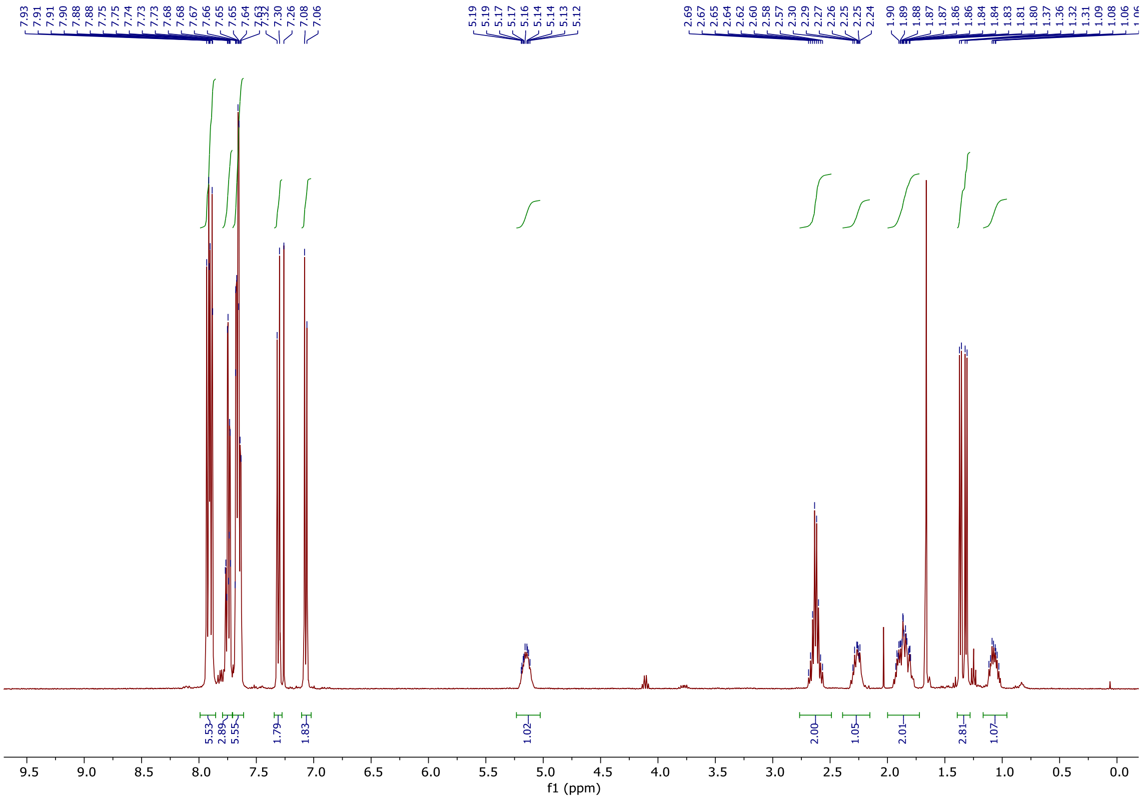

(5-(4-Bromophenyl)pentan-2-yl)triphenylphosphonium iodide (**10ac**) – $^{13}C\{^{1}H\}$ NMR spectrum ($CDCl_3$)

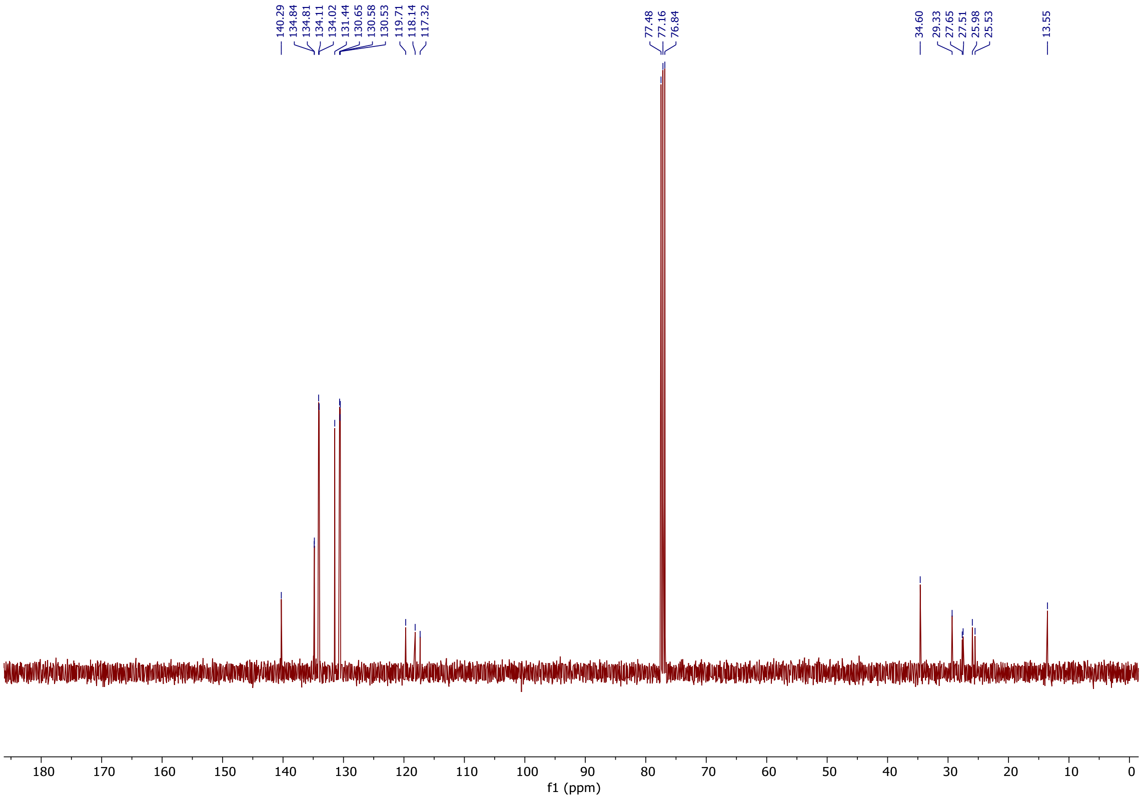

(5-(4-Bromophenyl)pentan-2-yl)triphenylphosphonium iodide (**10ac**) – $^{31}P\{^1H\}$ NMR spectrum ($CDCl_3$)

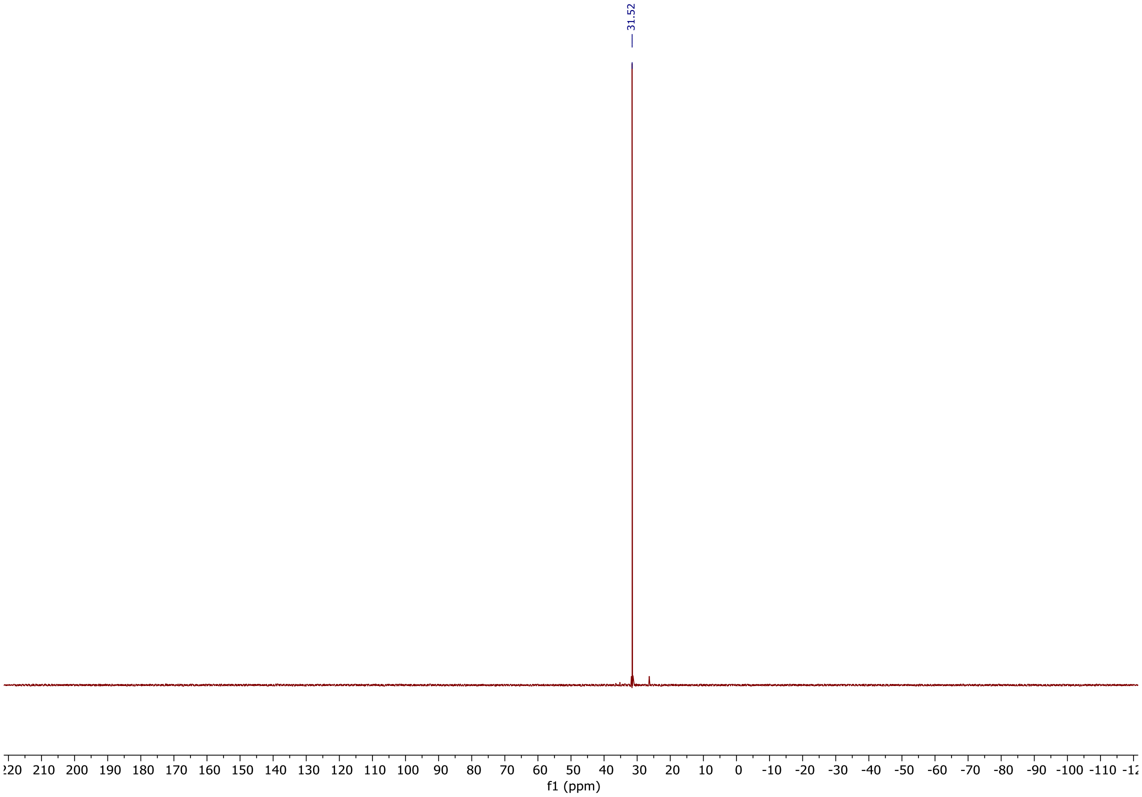

(5-((*tert*-Butyldiphenylsilyl)oxy)pentan-2-yl)triphenylphosphonium bromide (**10ad**) – ^1H NMR spectrum ($CDCl_3$)

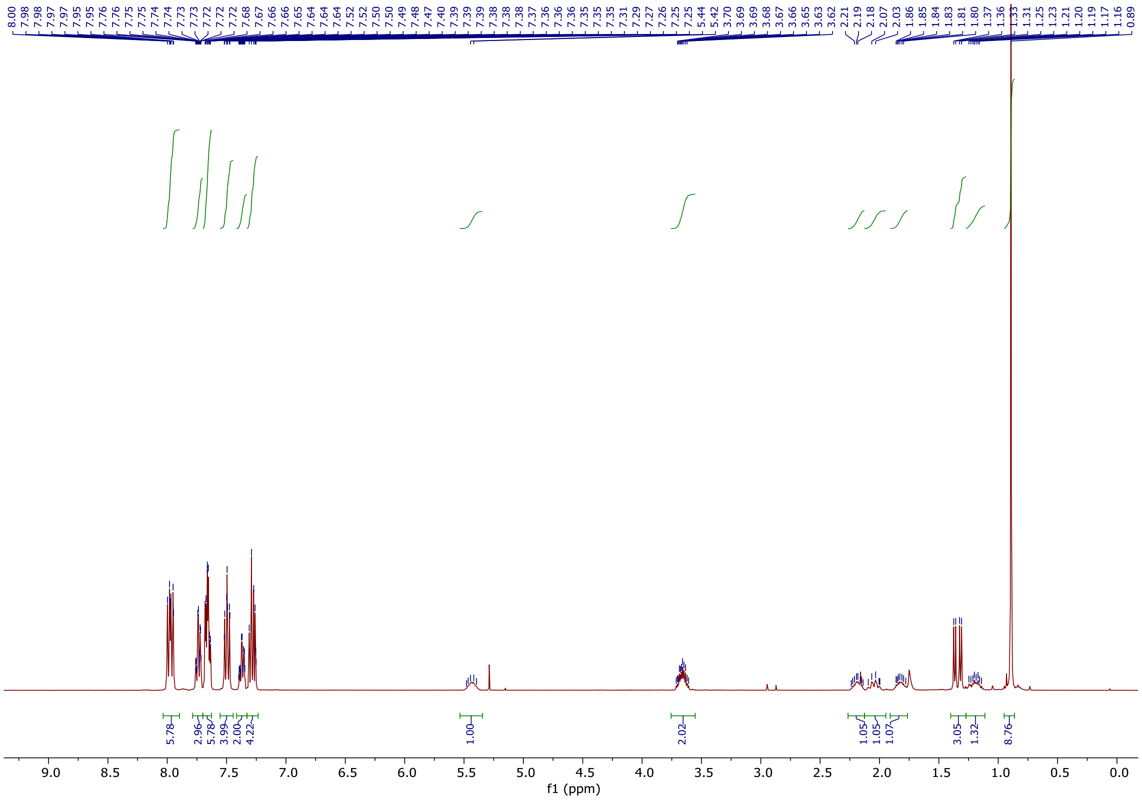

(5-((*tert*-Butyldiphenylsilyl)oxy)pentan-2-yl)triphenylphosphonium bromide (**10ad**) – $^{13}C\{^{1}H\}$ NMR spectrum ($CDCl_3$)

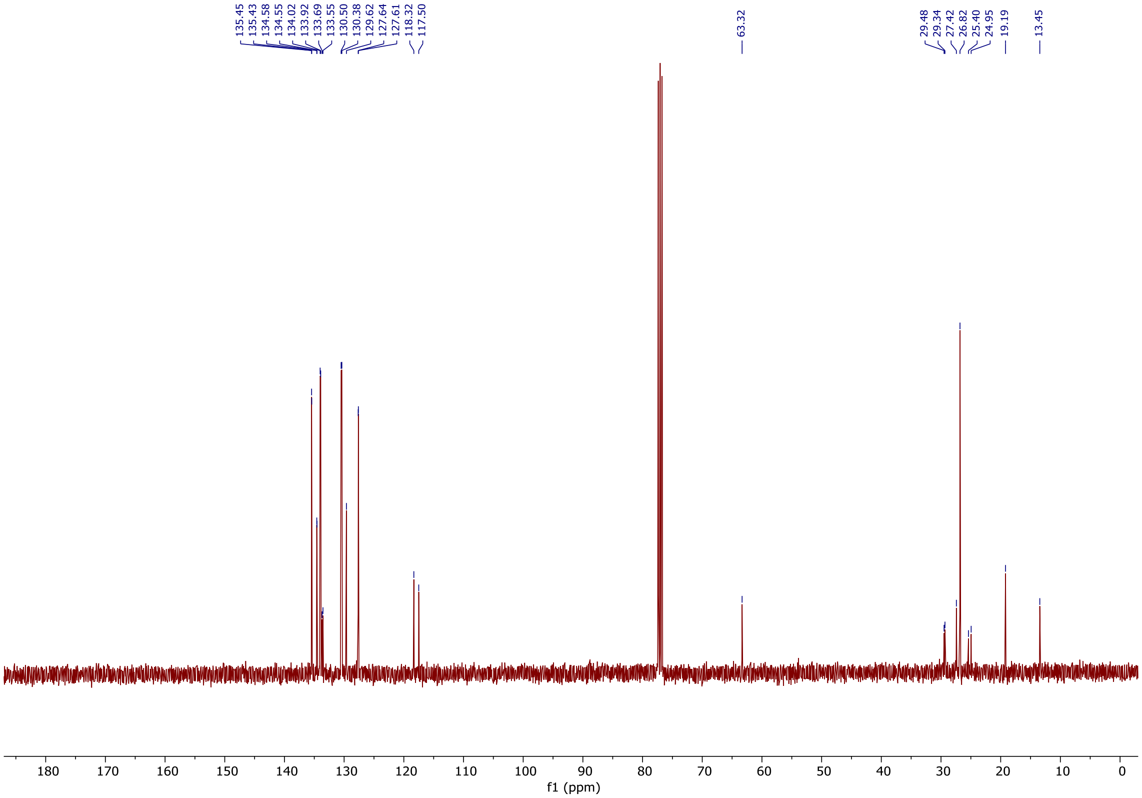

(5-((*tert*-Butyldiphenylsilyl)oxy)pentan-2-yl)triphenylphosphonium bromide (**10ad**) – $^{31}P\{^{1}H\}$ NMR spectrum ($CDCl_3$)

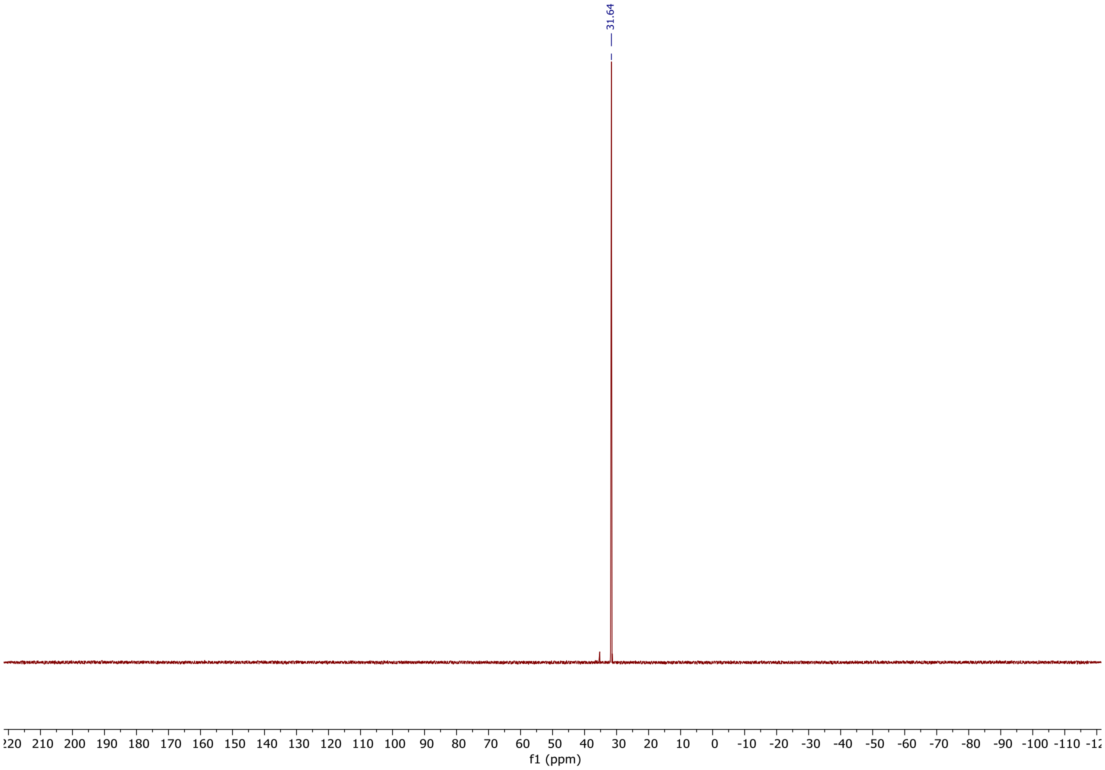

Triphenyl(5-(phenylthio)pentan-2-yl)phosphonium iodide (**10af**) – ^{1}H NMR spectrum ($CDCl_3$)

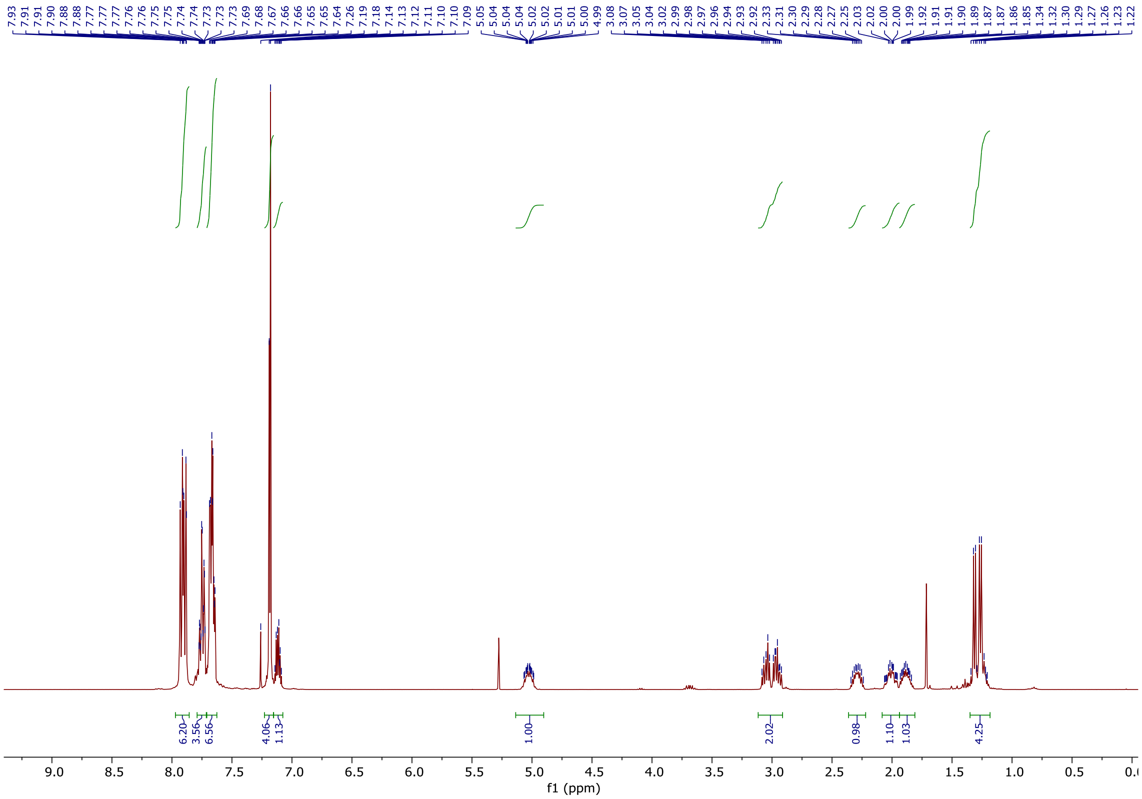

Triphenyl(5-(phenylthio)pentan-2-yl)phosphonium iodide (**10af**) – ^{13}C{^{1}H} NMR spectrum ($CDCl_3$)

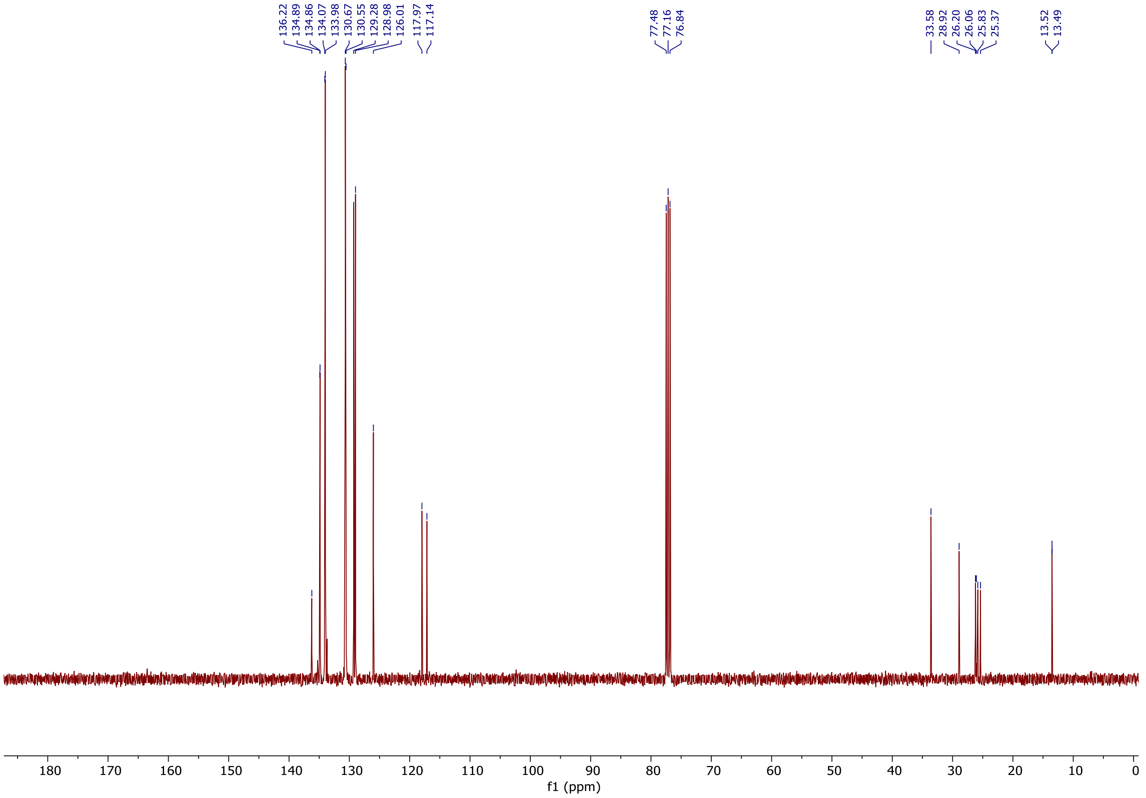

Triphenyl(5-(phenylthio)pentan-2-yl)phosphonium iodide (**10af**) – $^{31}P\{^1H\}$ NMR spectrum ($CDCl_3$)

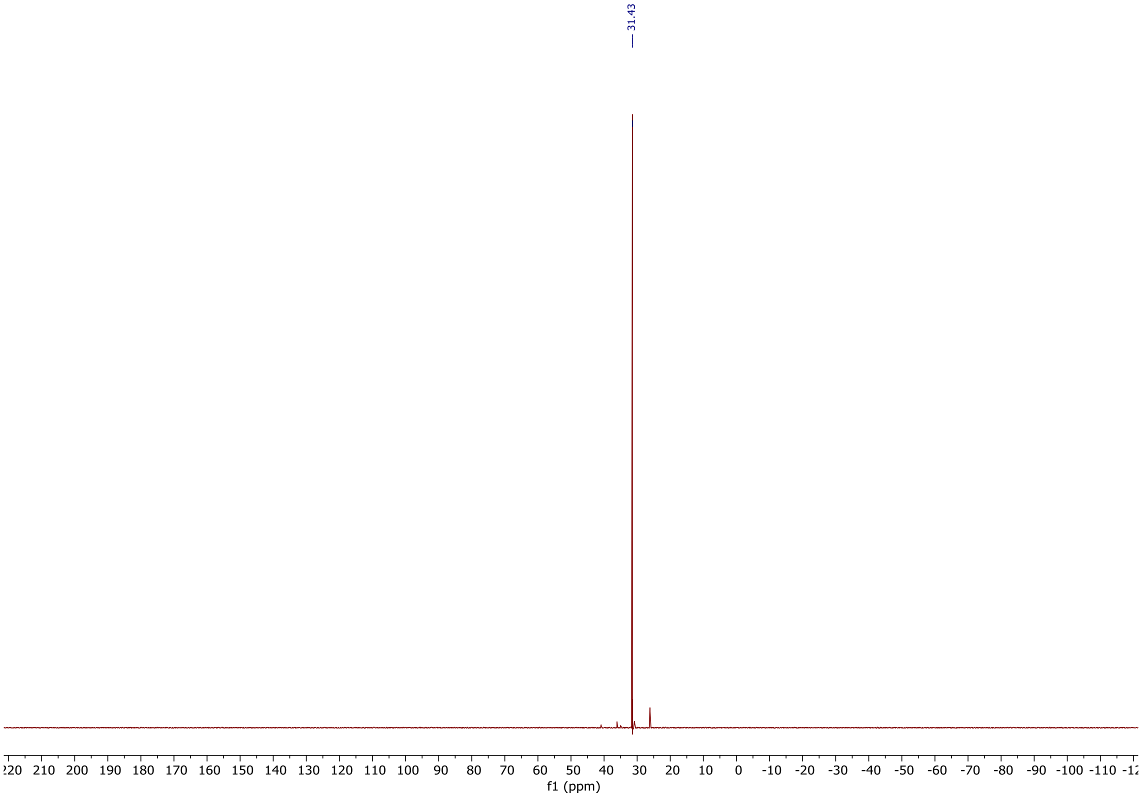

Phosphonium iodide **10ag** – ^1H NMR spectrum ($CDCl_3$)

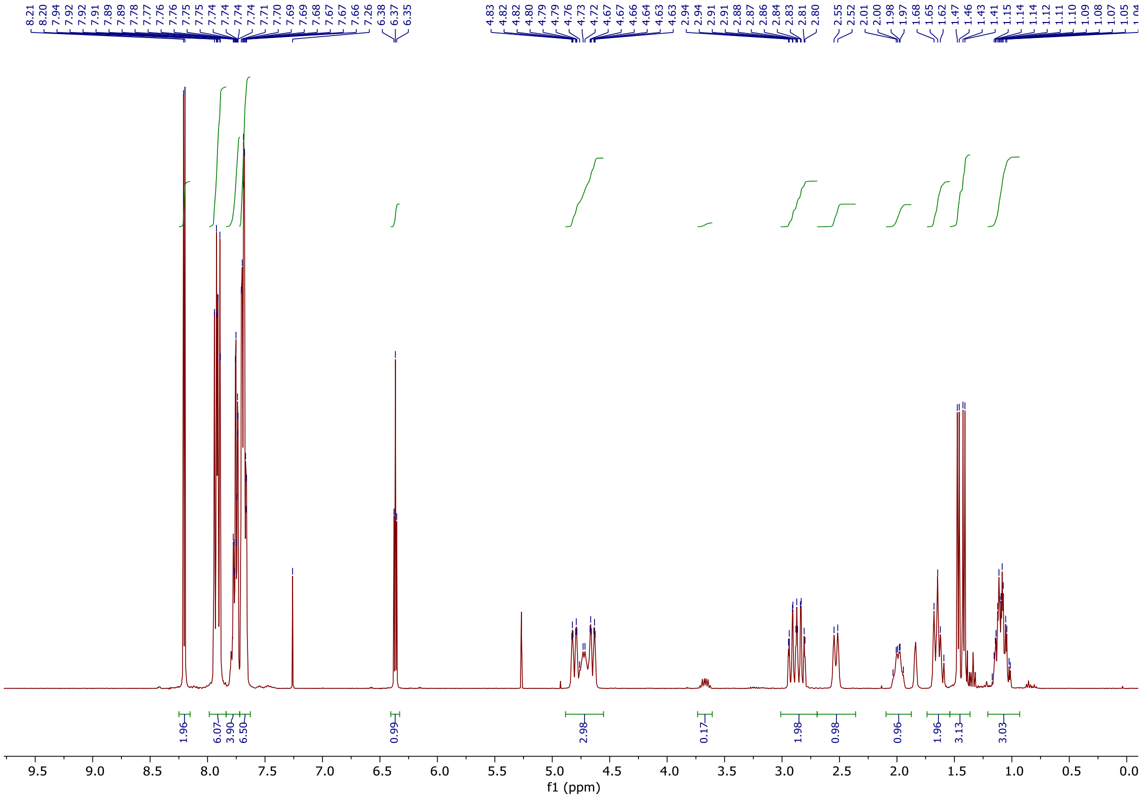

Phosphonium iodide **10ag** – $^{13}C\{^1H\}$ NMR spectrum ($CDCl_3$)

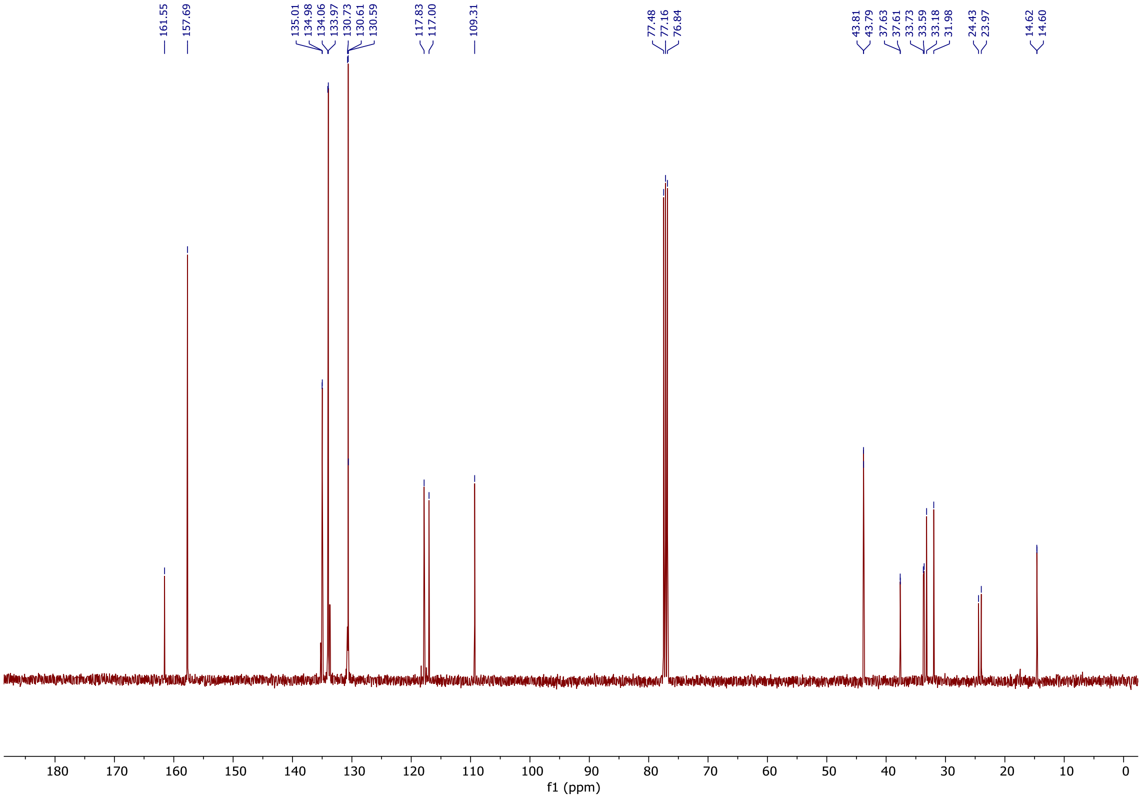

Phosphonium iodide **10ag** – $^{31}P\{^1H\}$ NMR spectrum ($CDCl_3$)

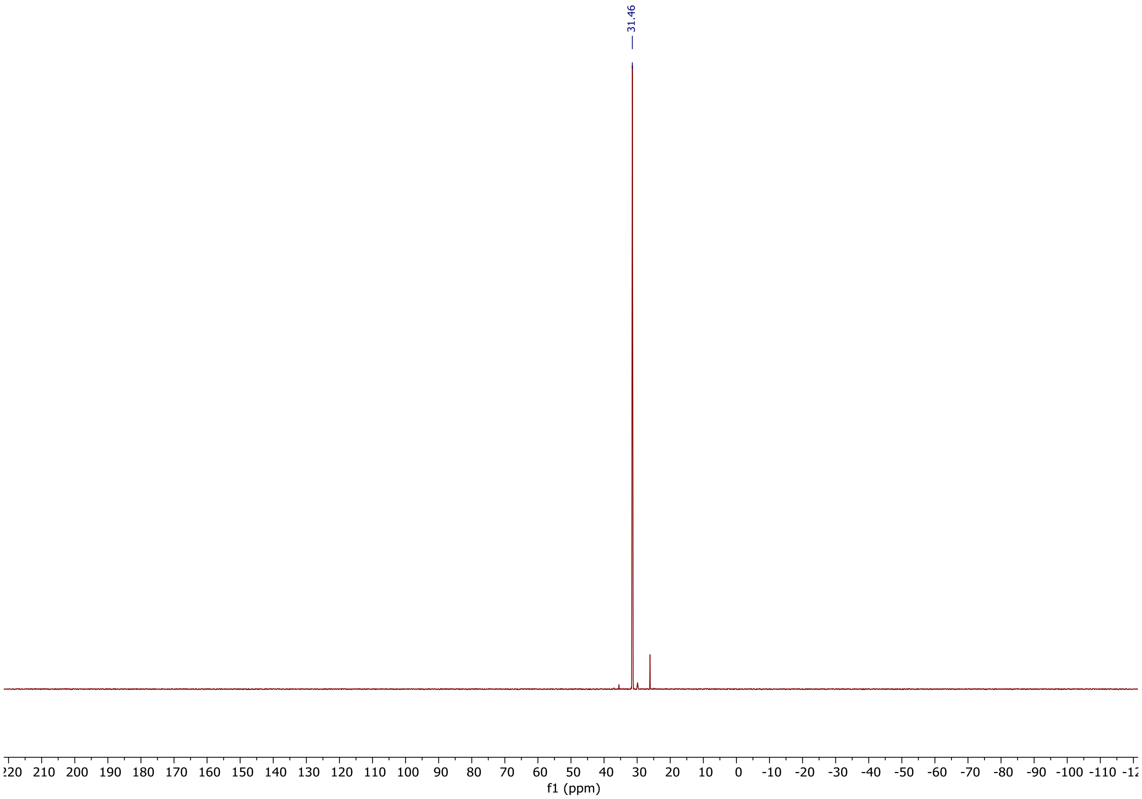

(6-Iodohexyl)benzene (**11a**) – ^{1}H NMR spectrum ($CDCl_3$)

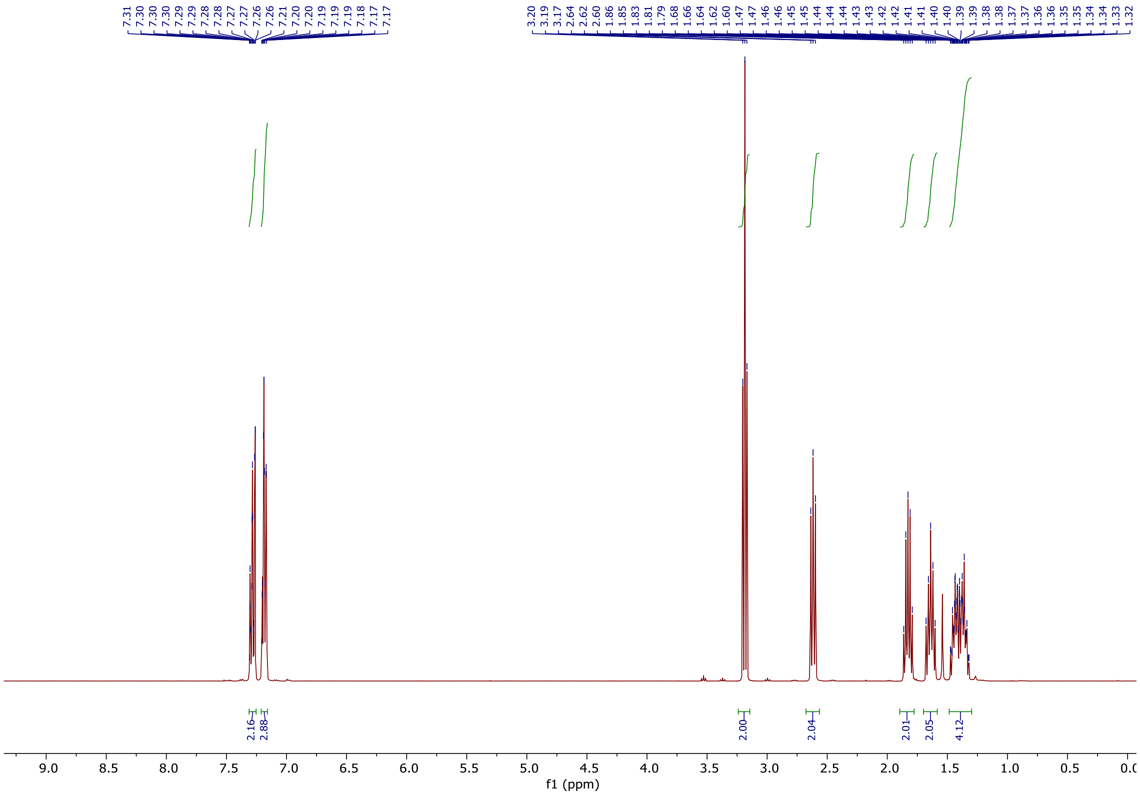

(6-Iodohexyl)benzene (**11a**) – ^{13}C{^{1}H} NMR spectrum ($CDCl_3$)

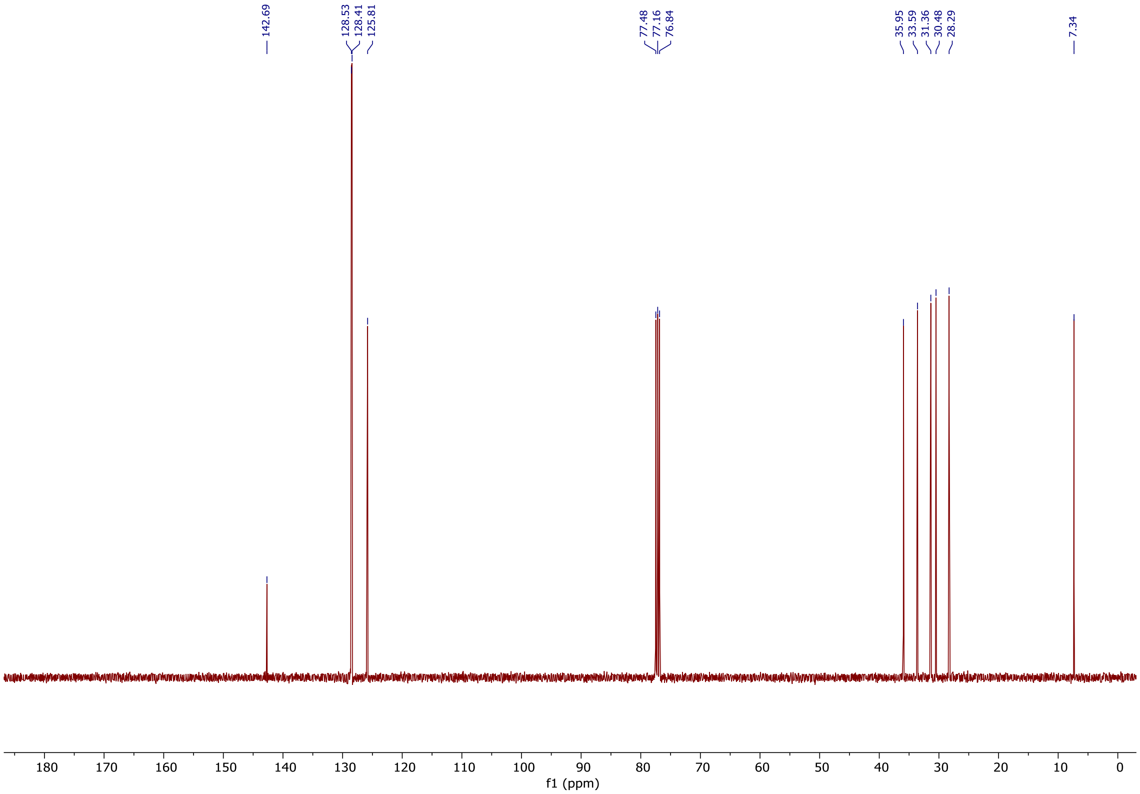

(4-Iodobutoxy)benzene (**11b**) – ^{1}H NMR spectrum ($CDCl_3$)

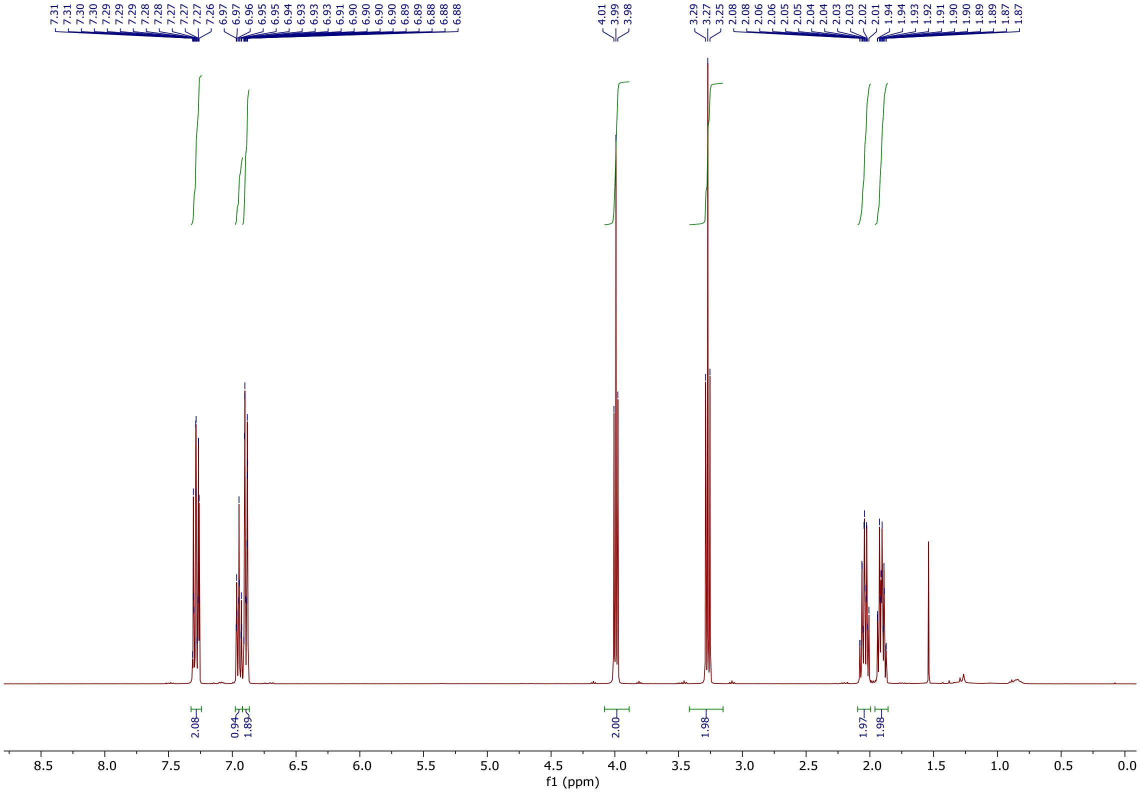

(4-Iodobutoxy)benzene (**11b**) – $^{13}C\{^{1}H\}$ NMR spectrum ($CDCl_3$)

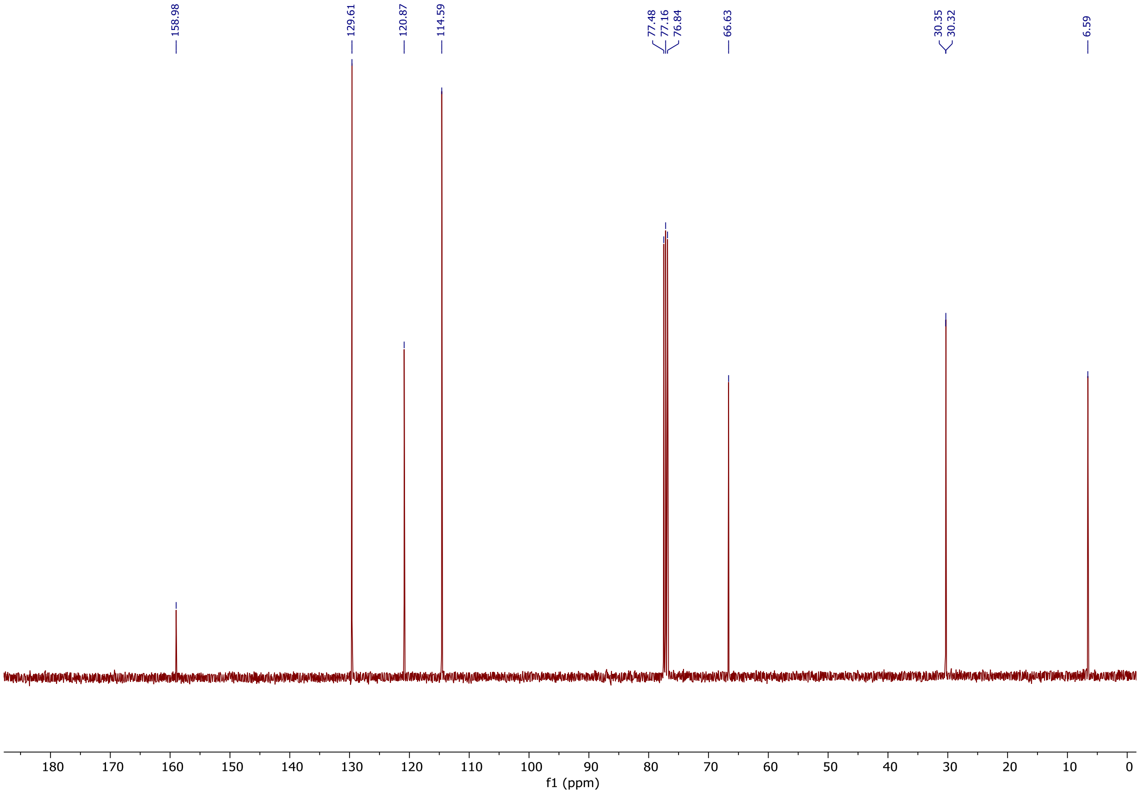

tert-Butyl(3-iodopropoxy)diphenylsilane (**11c**) – ^{1}H NMR spectrum ($CDCl_3$)

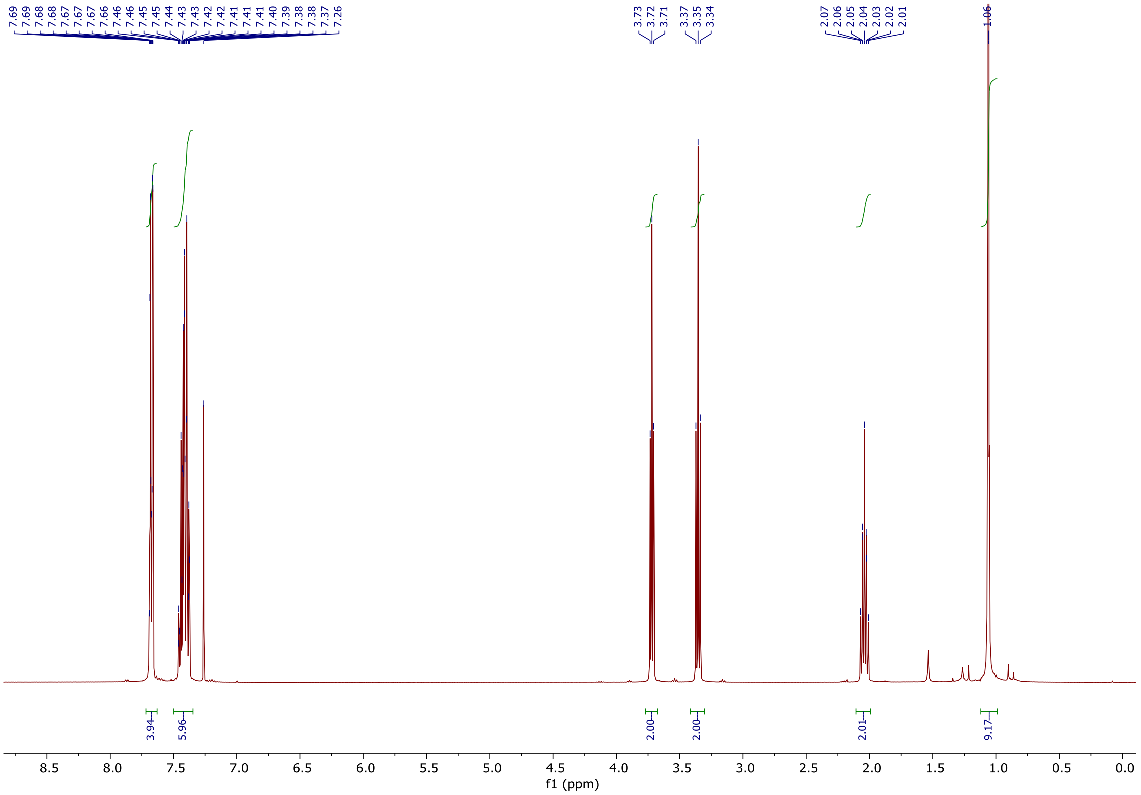

tert-Butyl(3-iodopropoxy)diphenylsilane (**11c**) – $^{13}C\{^1H\}$ NMR spectrum ($CDCl_3$)

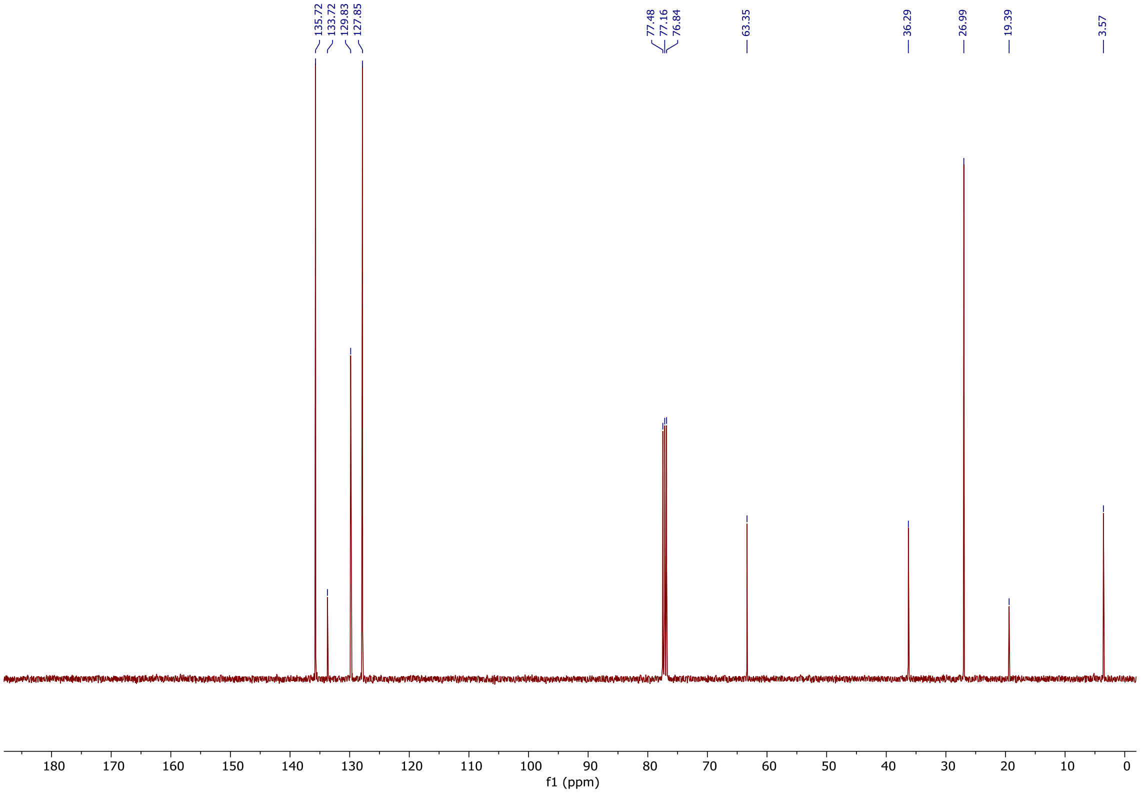

5-(3-iodopropyl)benzo[d][1,3]dioxole (**11d**) – ^{1}H NMR spectrum ($CDCl_3$)

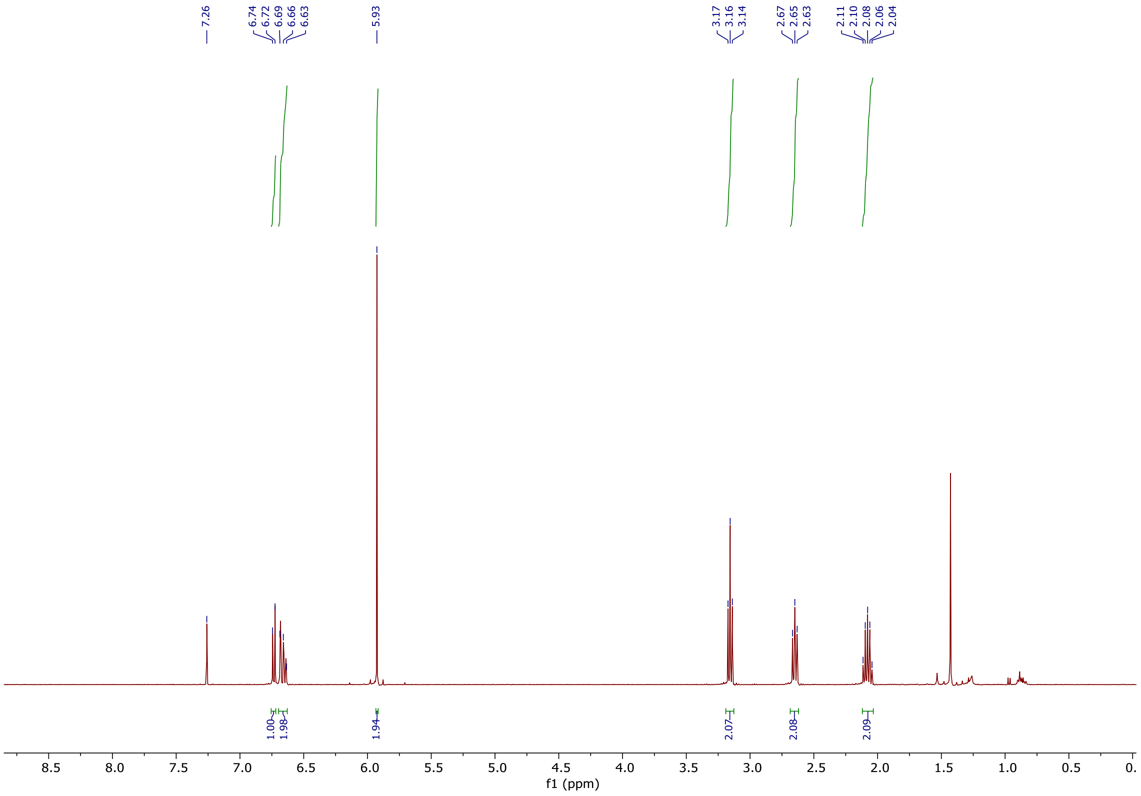

5-(3-iodopropyl)benzo[d][1,3]dioxole (**11d**) – ^{13}C{^{1}H} NMR spectrum ($CDCl_3$)

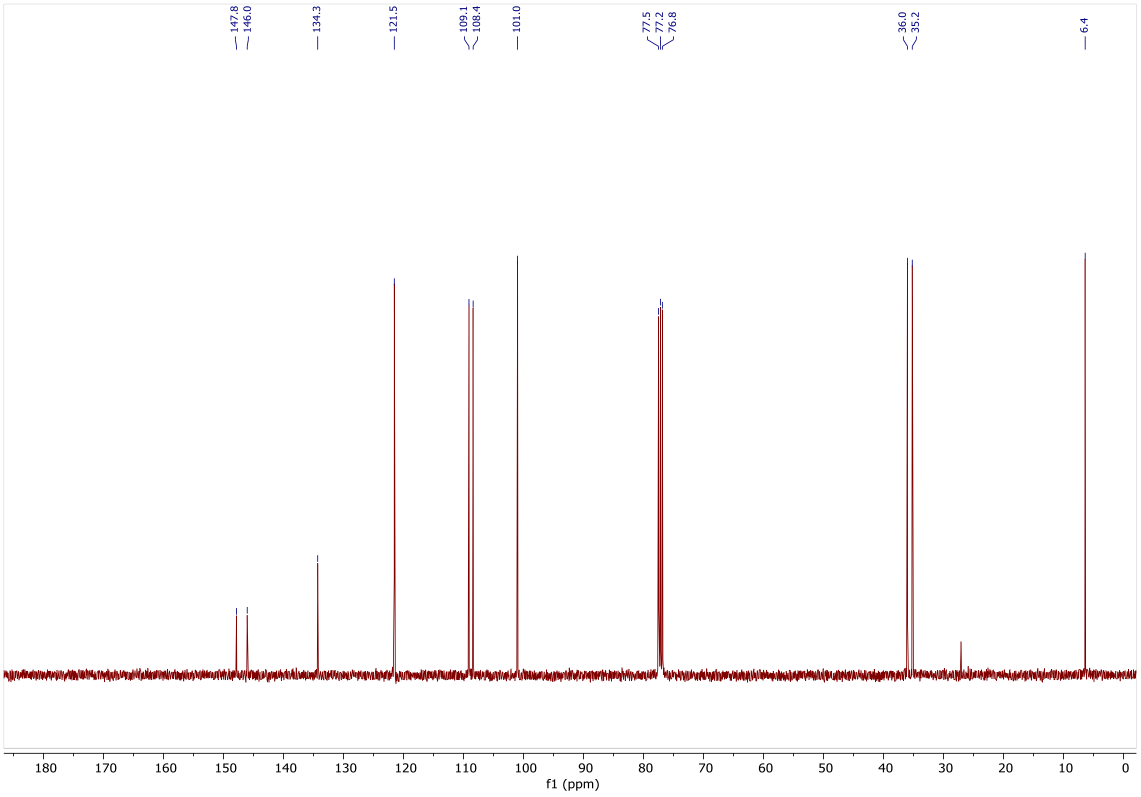

(3-(Iodomethyl)piperidin-1-yl)(phenyl)methanone (**11e**) – ^{1}H NMR spectrum ($CDCl_3$)

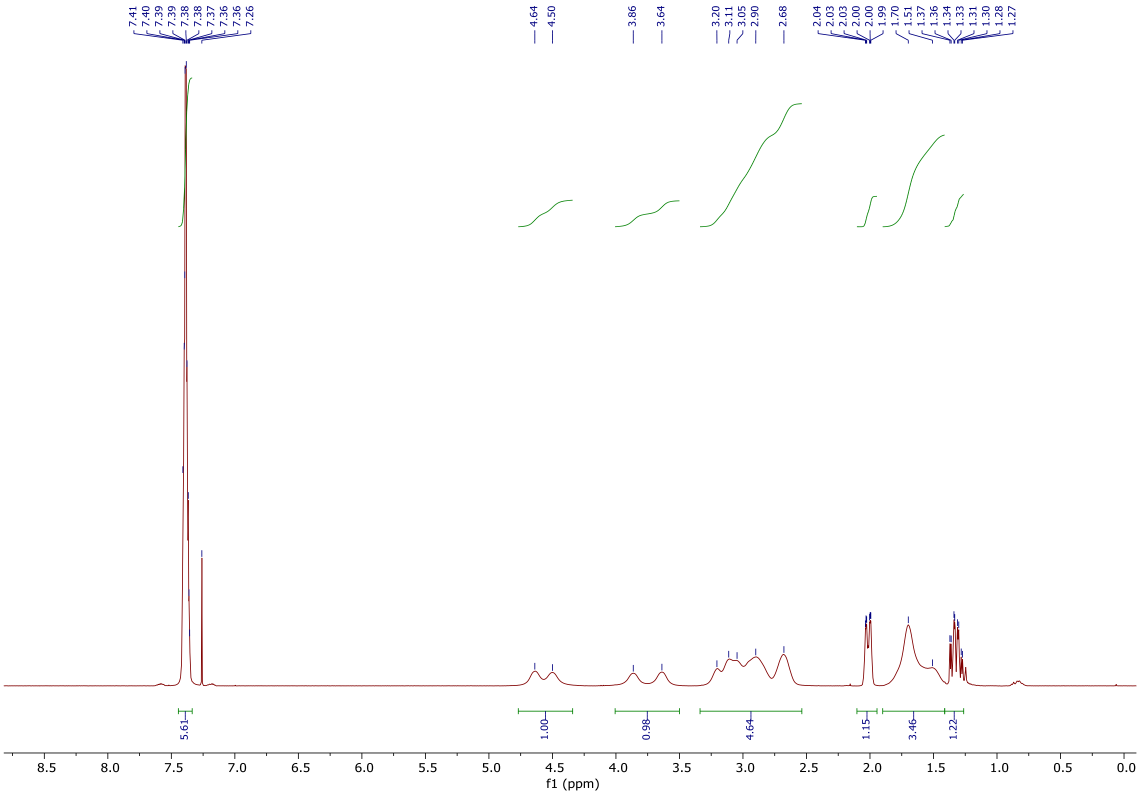

(3-(Iodomethyl)piperidin-1-yl)(phenyl)methanone (**11e**) – $^{13}C\{^{1}H\}$ NMR spectrum ($CDCl_3$)

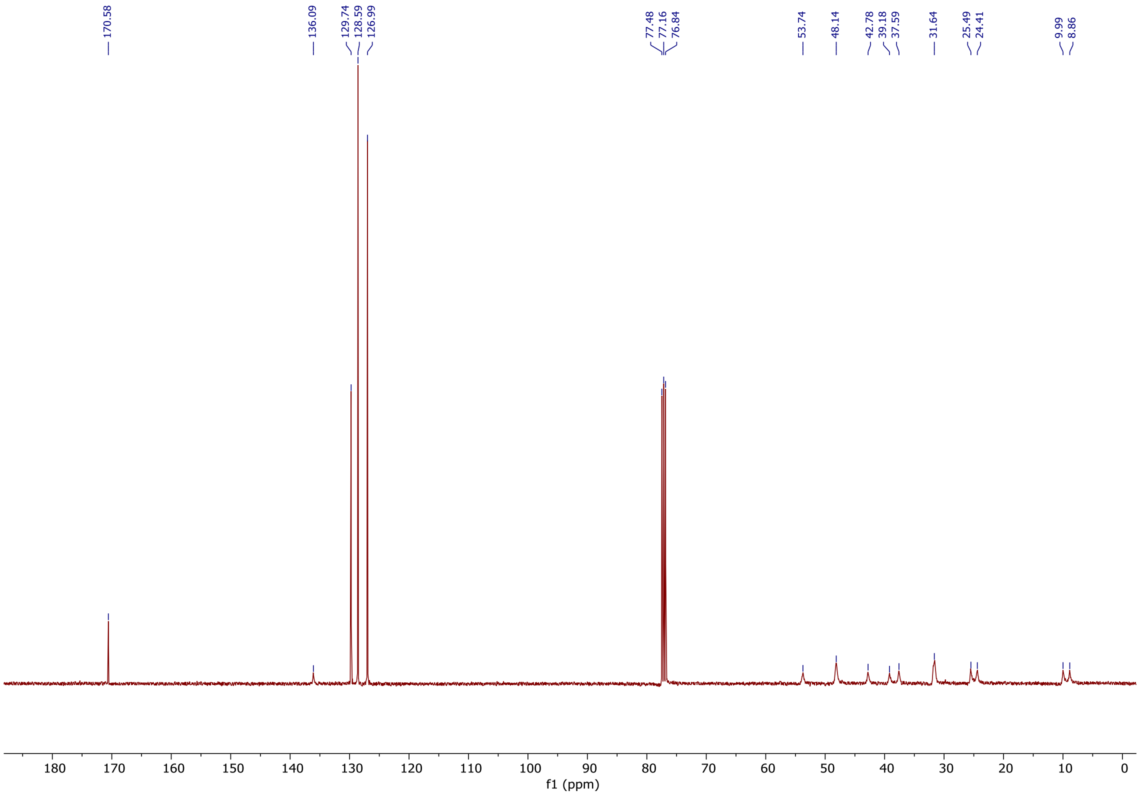

1-Iodo-4-(3-iodopropyl)benzene (**11g**) – ^{1}H NMR spectrum ($CDCl_3$)

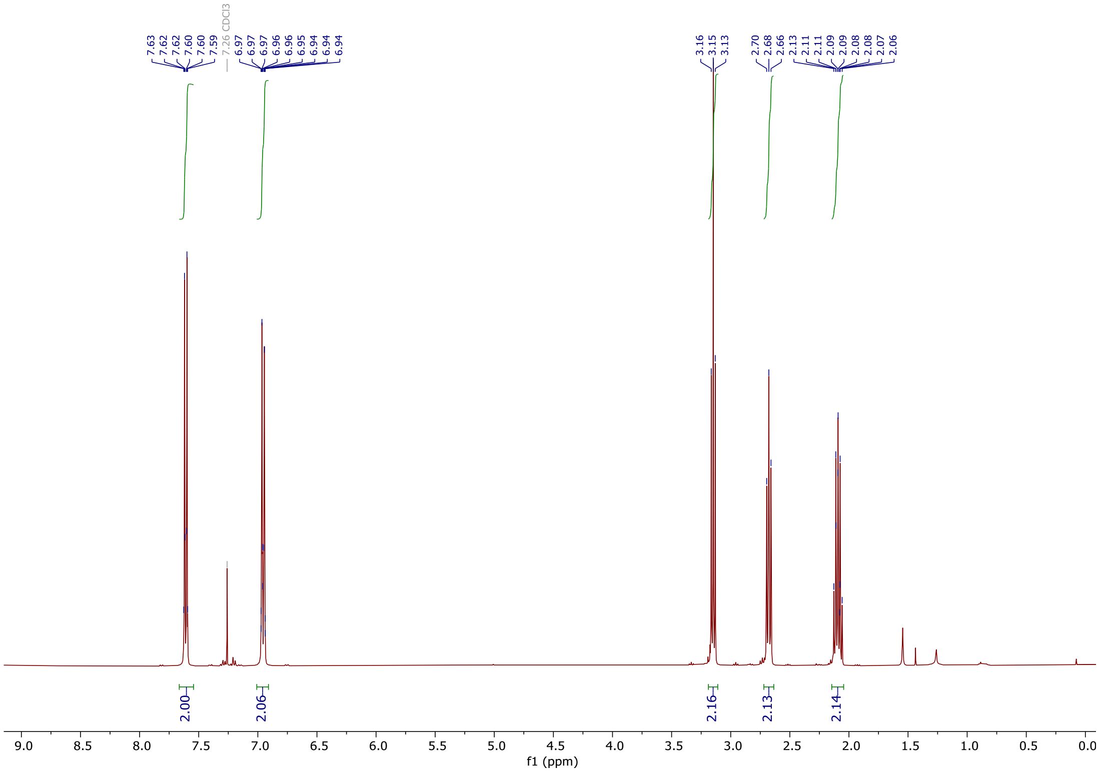

1-Iodo-4-(3-iodopropyl)benzene (**11g**) – $^{13}C\{^{1}H\}$ NMR spectrum ($CDCl_3$)

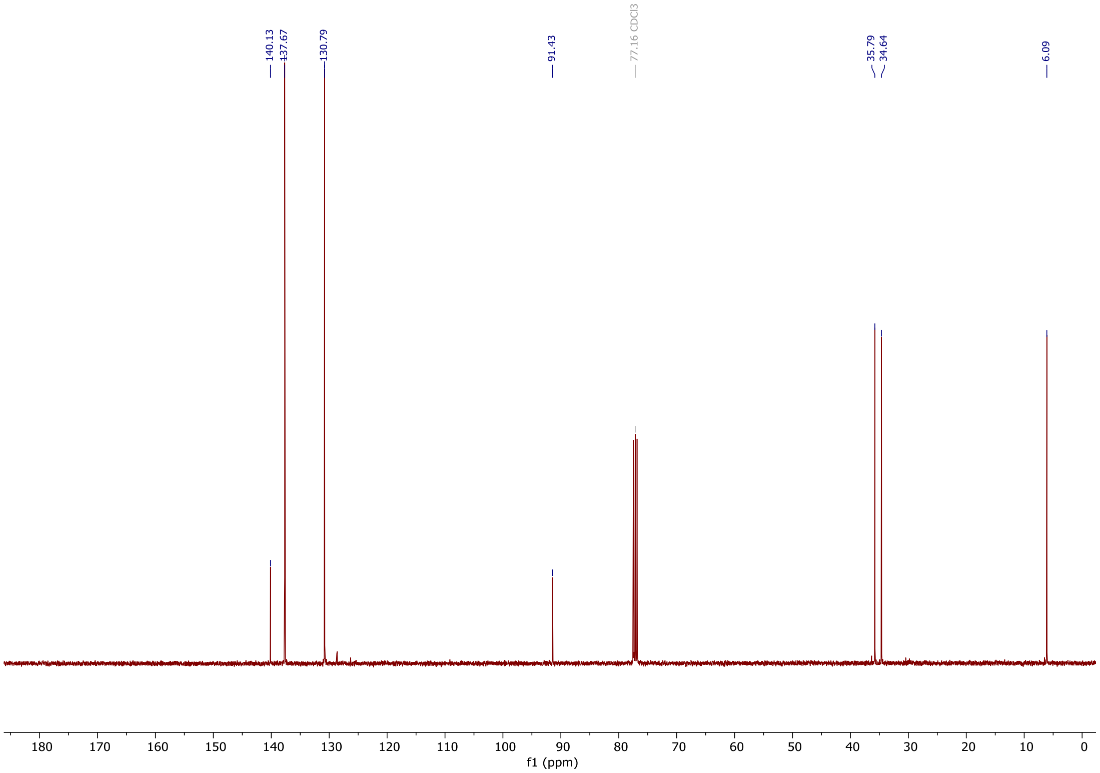

1-Bromo-4-(3-iodopropyl)benzene (**11h**) – ^{1}H NMR spectrum ($CDCl_3$)

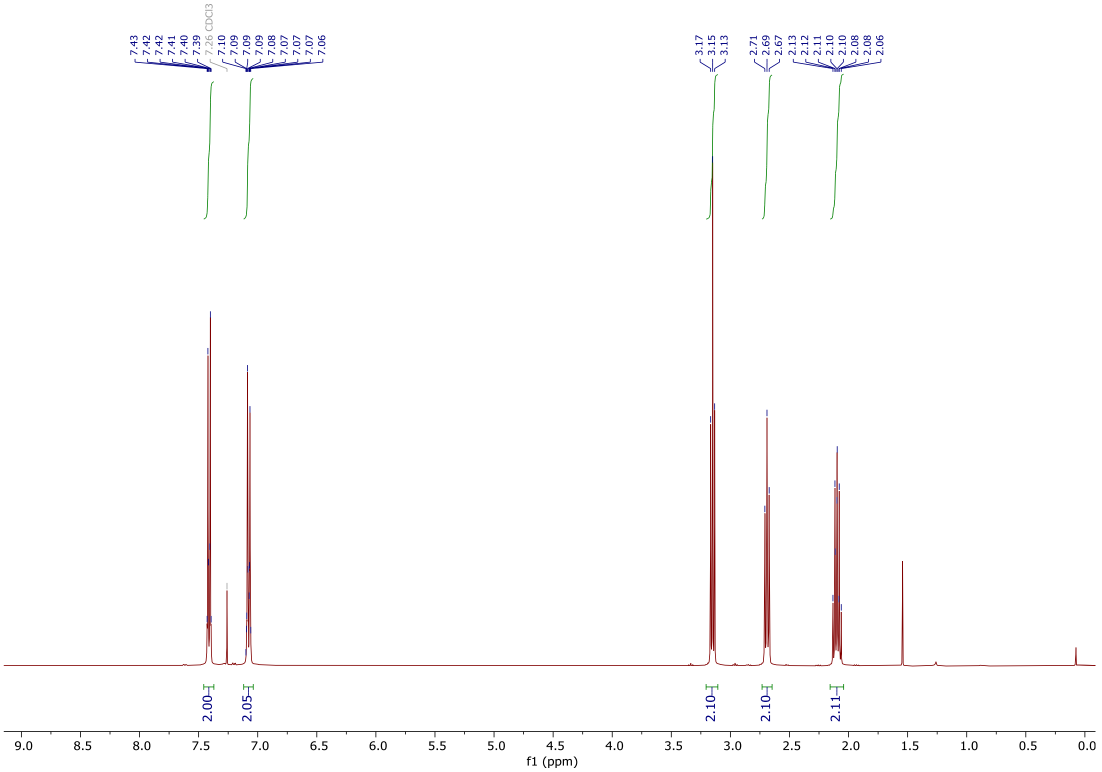

1-Bromo-4-(3-iodopropyl)benzene (**11h**) – $^{13}C\{^{1}H\}$ NMR spectrum ($CDCl_3$)

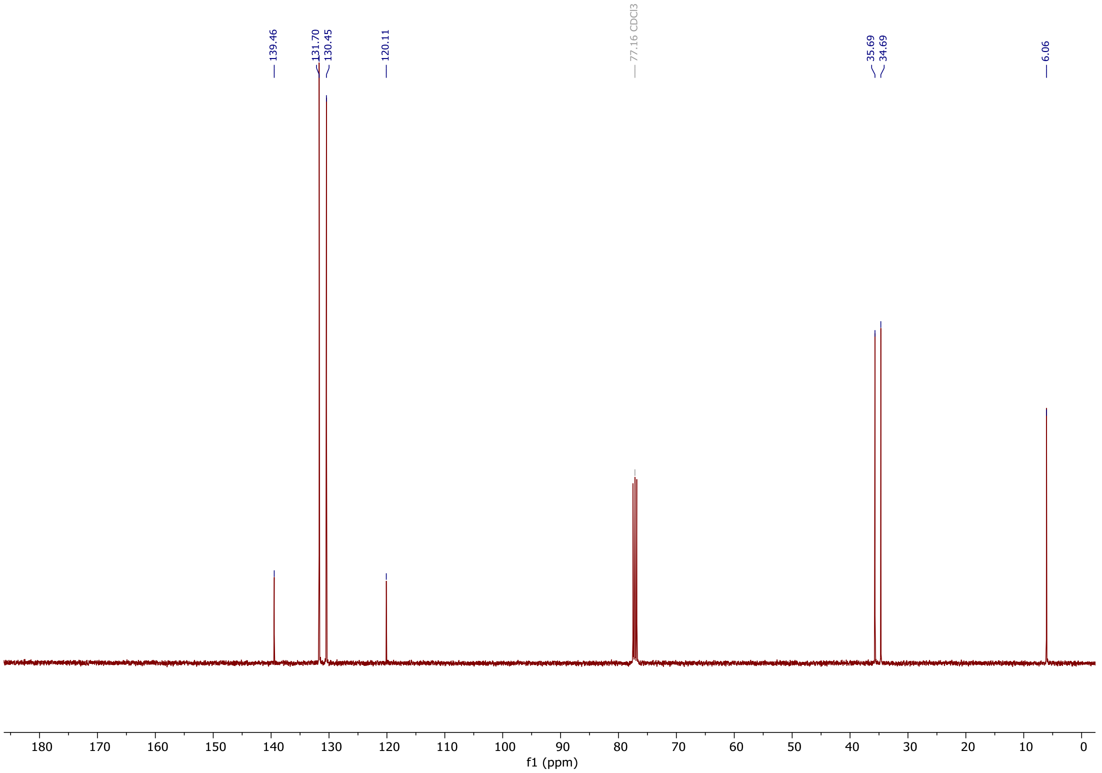

1-Chloro-4-(3-iodopropyl)benzene (**11i**) – ^{1}H NMR spectrum ($CDCl_3$)

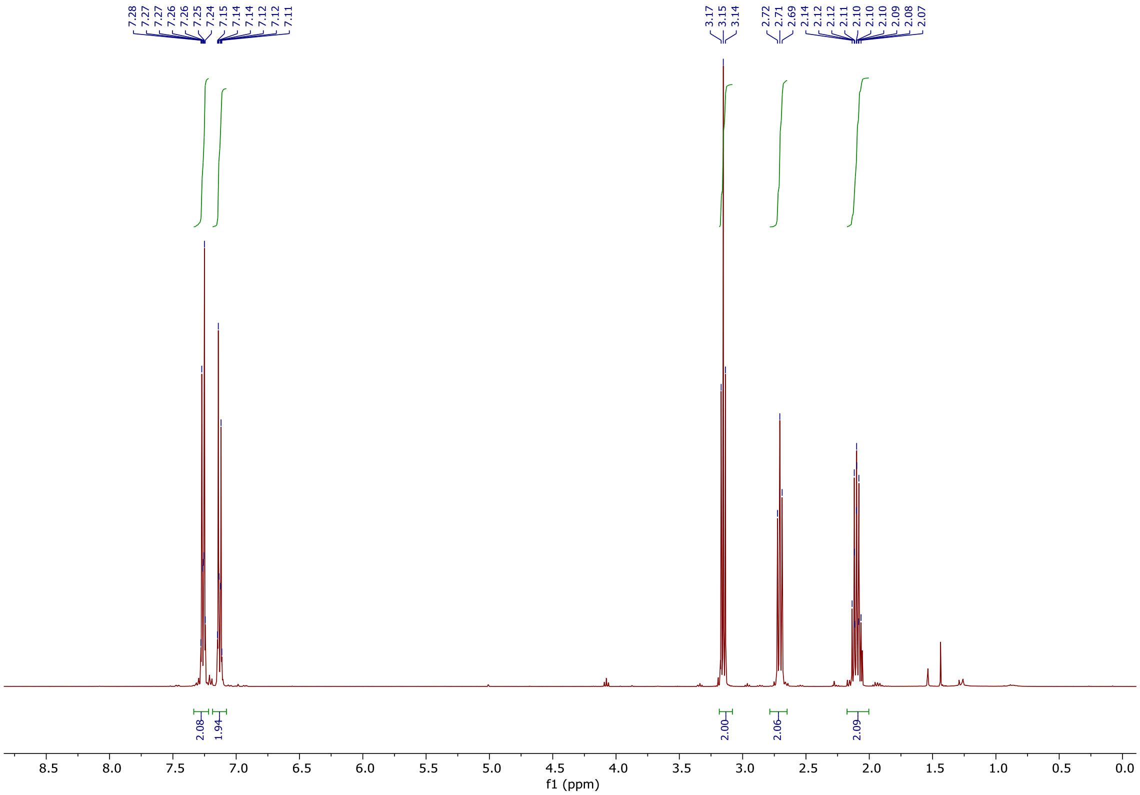

1-Chloro-4-(3-iodopropyl)benzene (**11i**) – ^{13}C{^{1}H} NMR spectrum ($CDCl_3$)

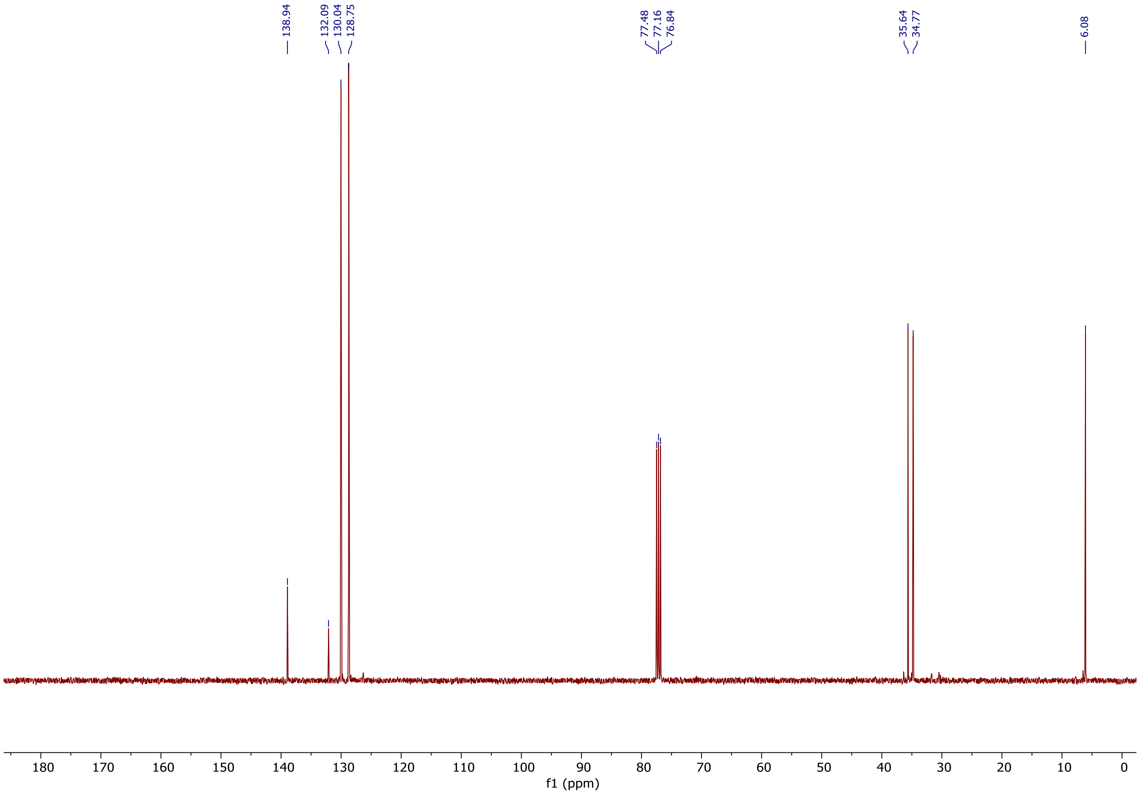

2-(4-(3-iodopropyl)phenyl)-4,4,5,5-tetramethyl-1,3,2-dioxaborolane (**11j**) – ^{1}H NMR spectrum ($CDCl_3$)

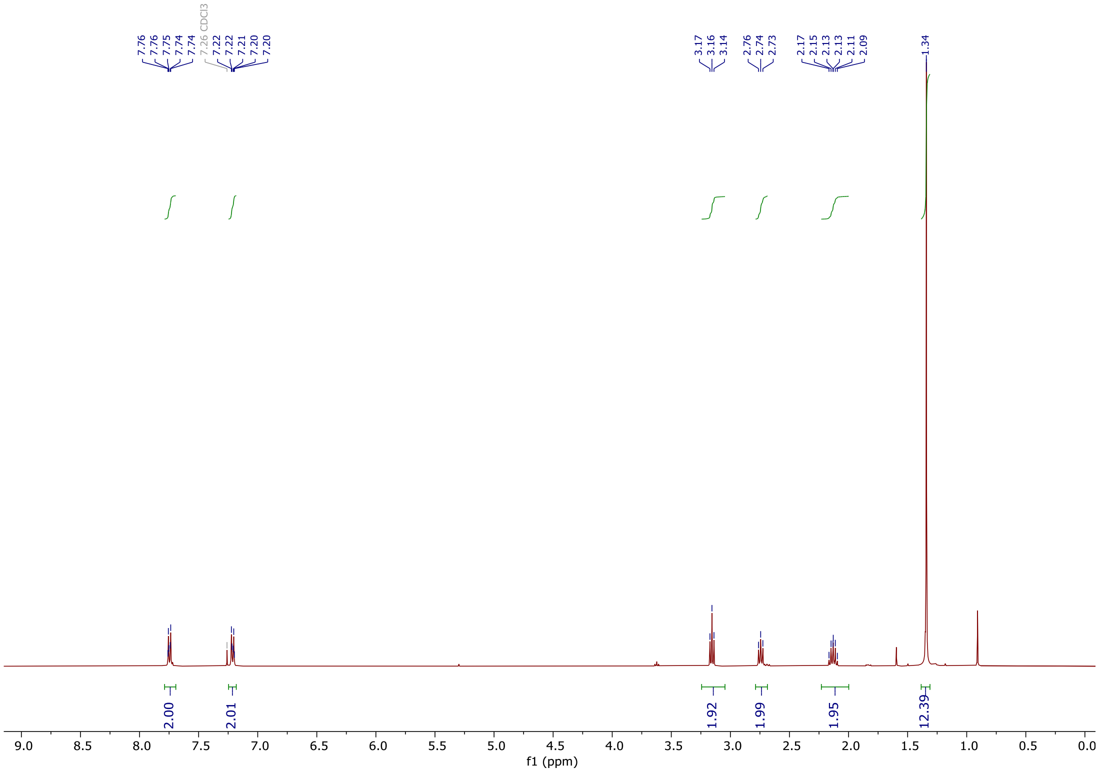

2-(4-(3-iodopropyl)phenyl)-4,4,5,5-tetramethyl-1,3,2-dioxaborolane (**11j**) – ^{11}B NMR spectrum ($CDCl_3$)

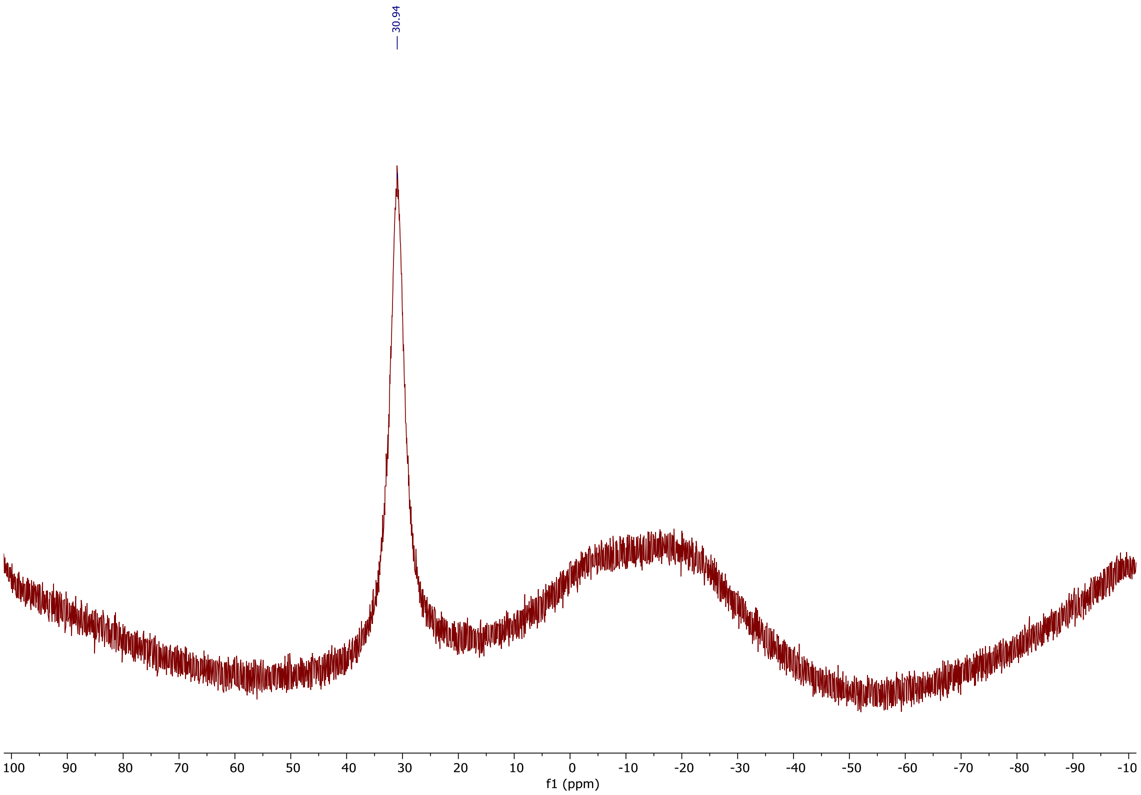

2-(4-(3-iodopropyl)phenyl)-4,4,5,5-tetramethyl-1,3,2-dioxaborolane (**11j**) – $^{13}C\{^{1}H\}$ NMR spectrum ($CDCl_3$)

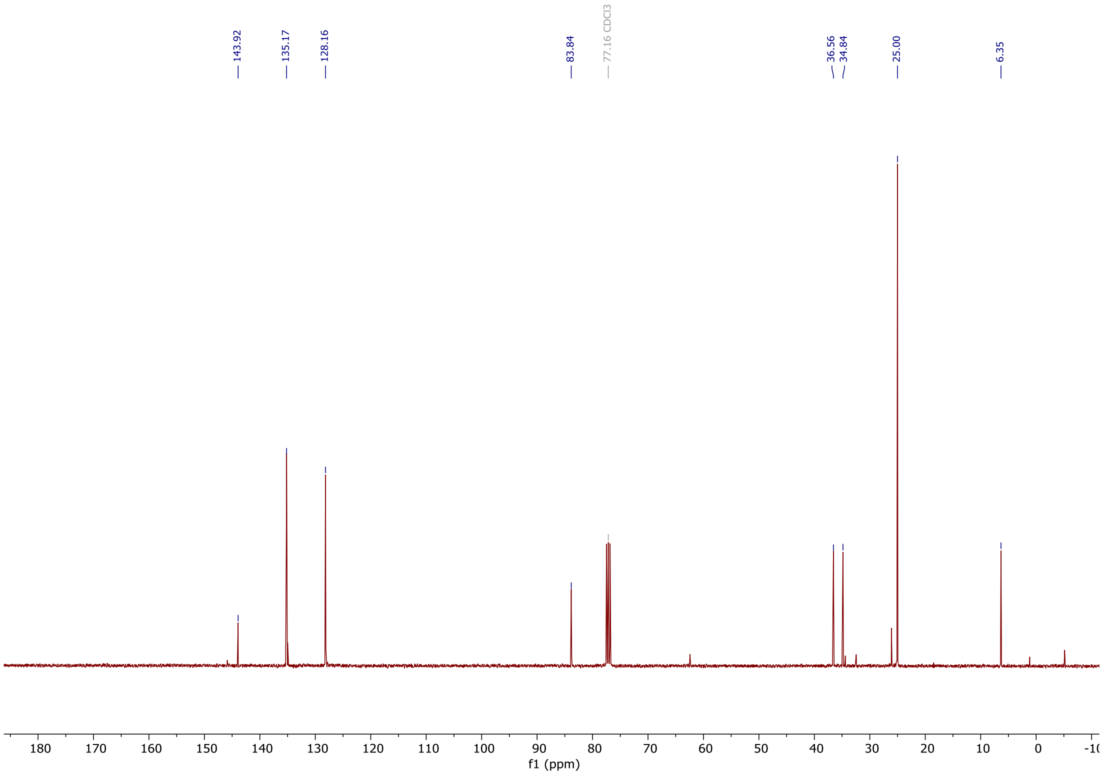

10-(4-Iodobutyl)-10*H*-phenoxazine (**11k**) – ^{1}H NMR spectrum ($CDCl_3$)

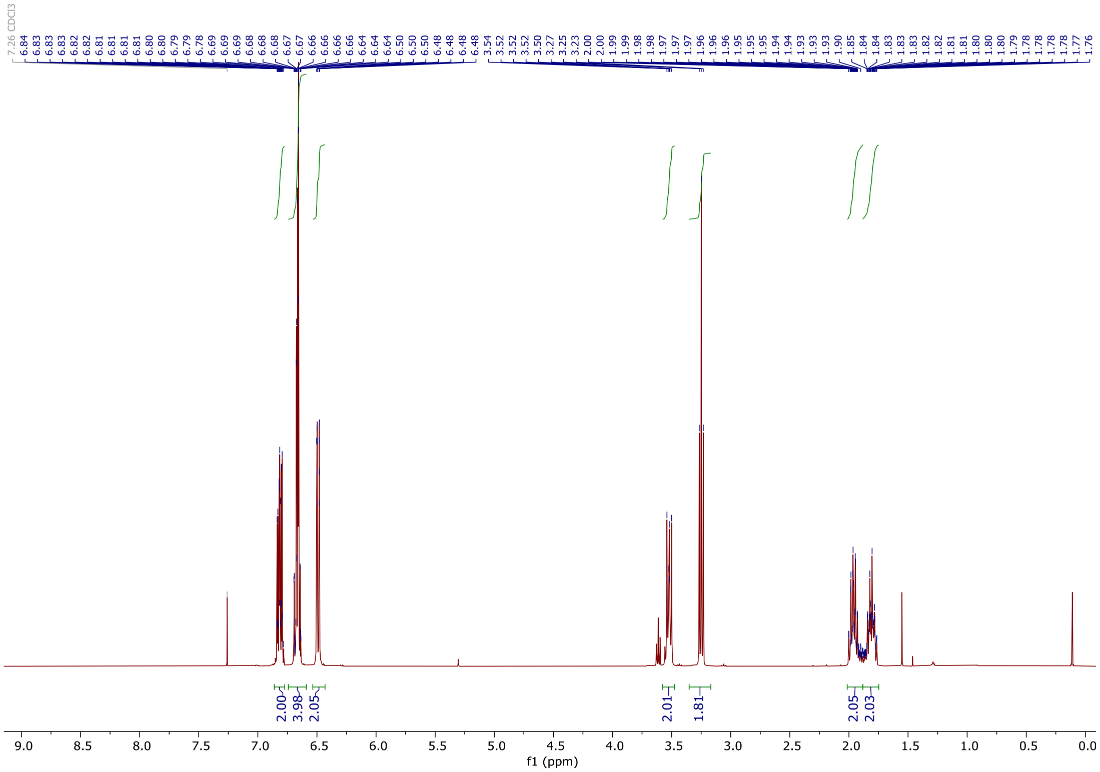

10-(4-Iodobutyl)-10*H*-phenoxazine (**11k**) – $^{13}C\{^{1}H\}$ NMR spectrum ($CDCl_3$)

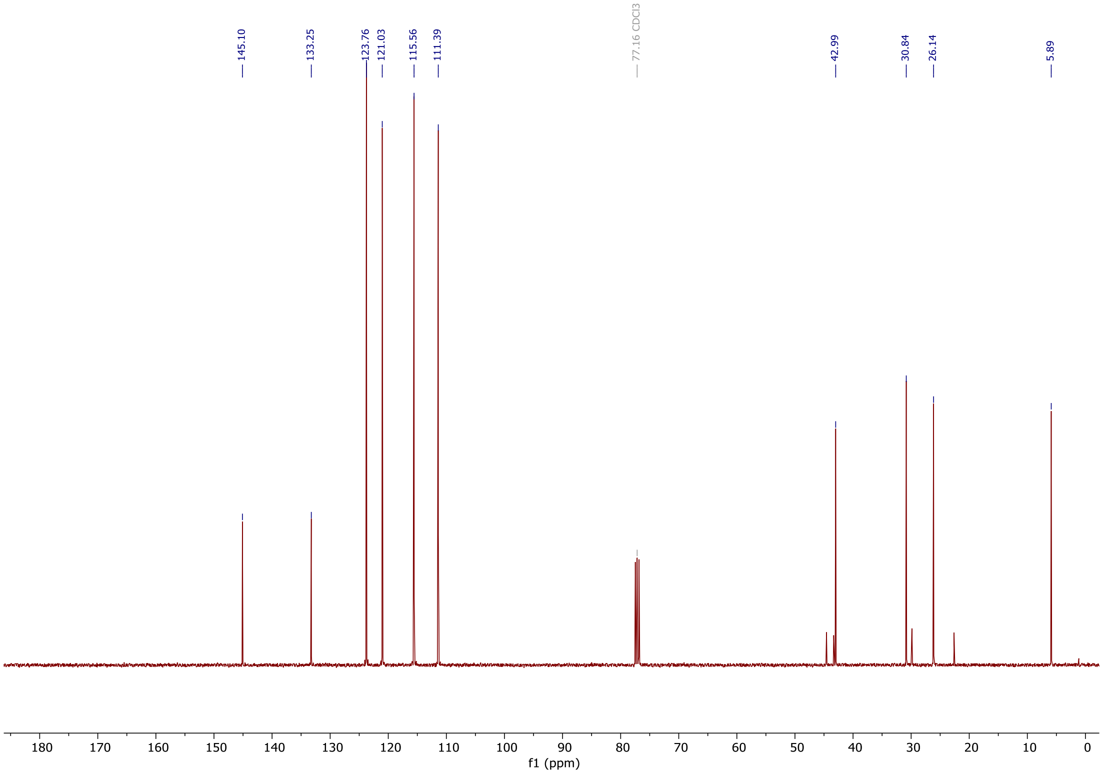

1-(4-Bromobutyl)-2-phenyl-1H-indole (**11l**) – ^{1}H NMR spectrum ($CDCl_3$)

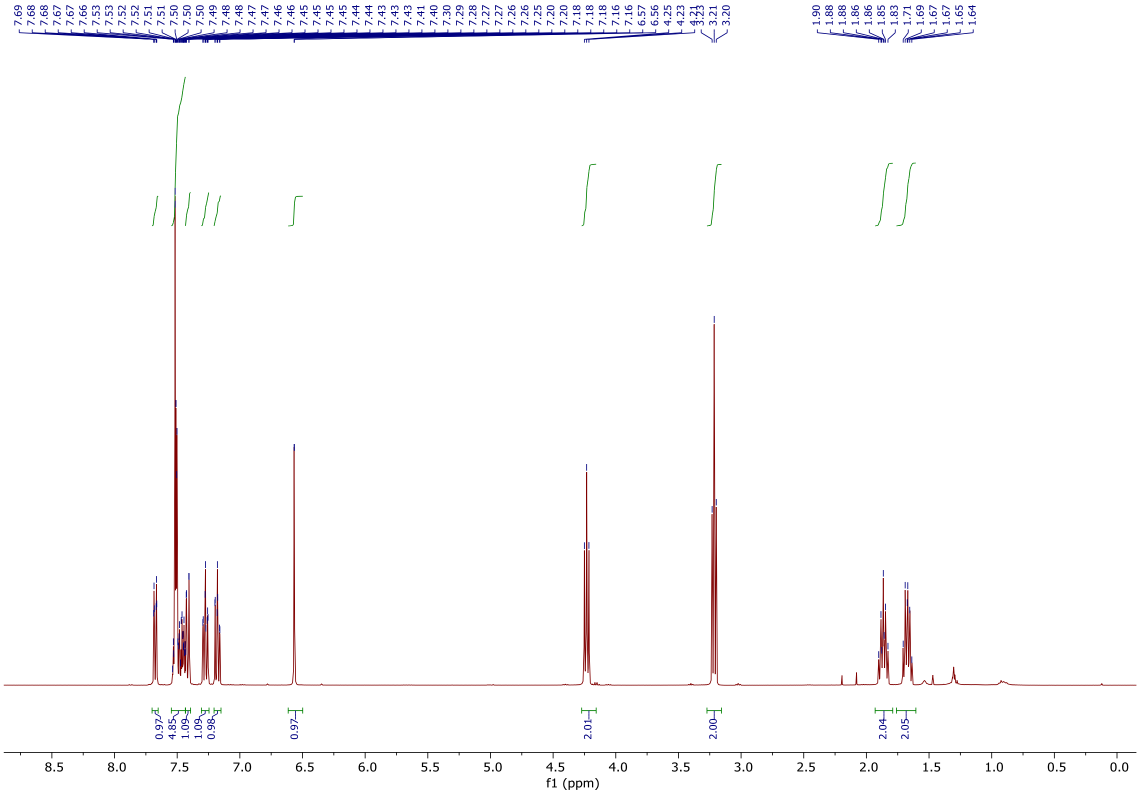

1-(4-Bromobutyl)-2-phenyl-1H-indole (**11l**) – $^{13}C\{^{1}H\}$ NMR spectrum ($CDCl_3$)

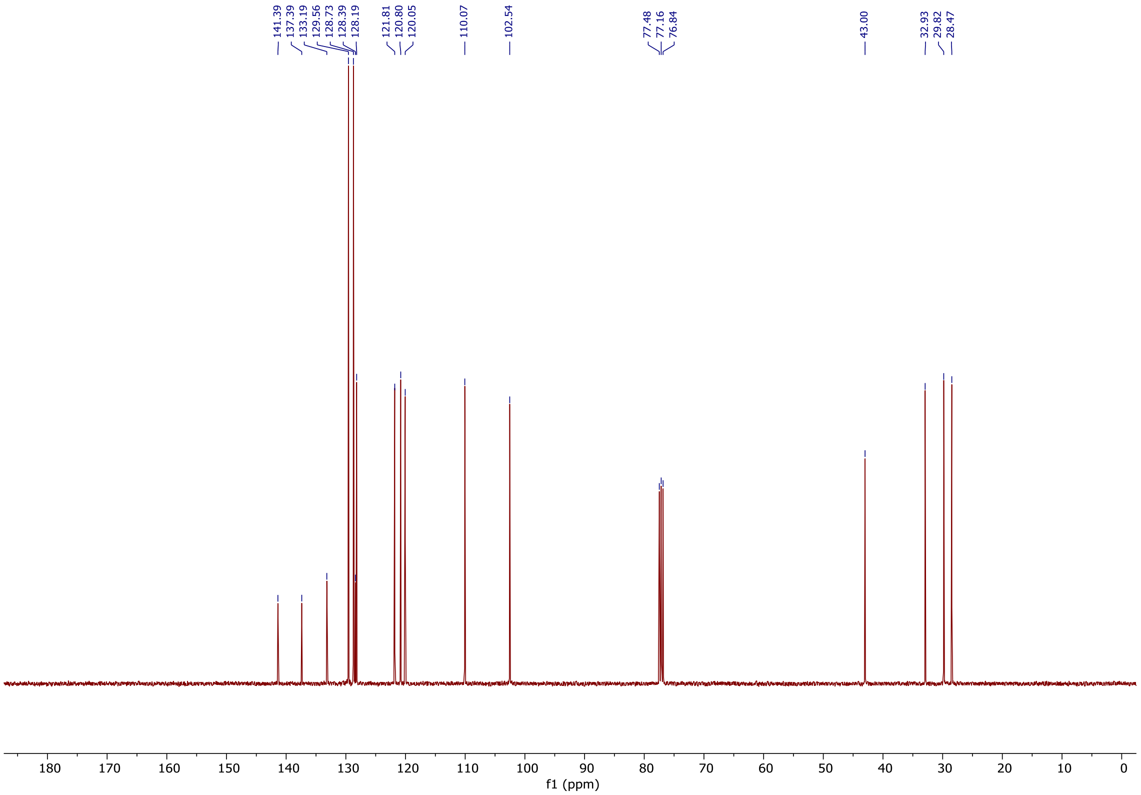

2-(4-(Iodomethyl)piperidin-1-yl)pyrimidine (**11m**) – ^{1}H NMR spectrum ($CDCl_3$)

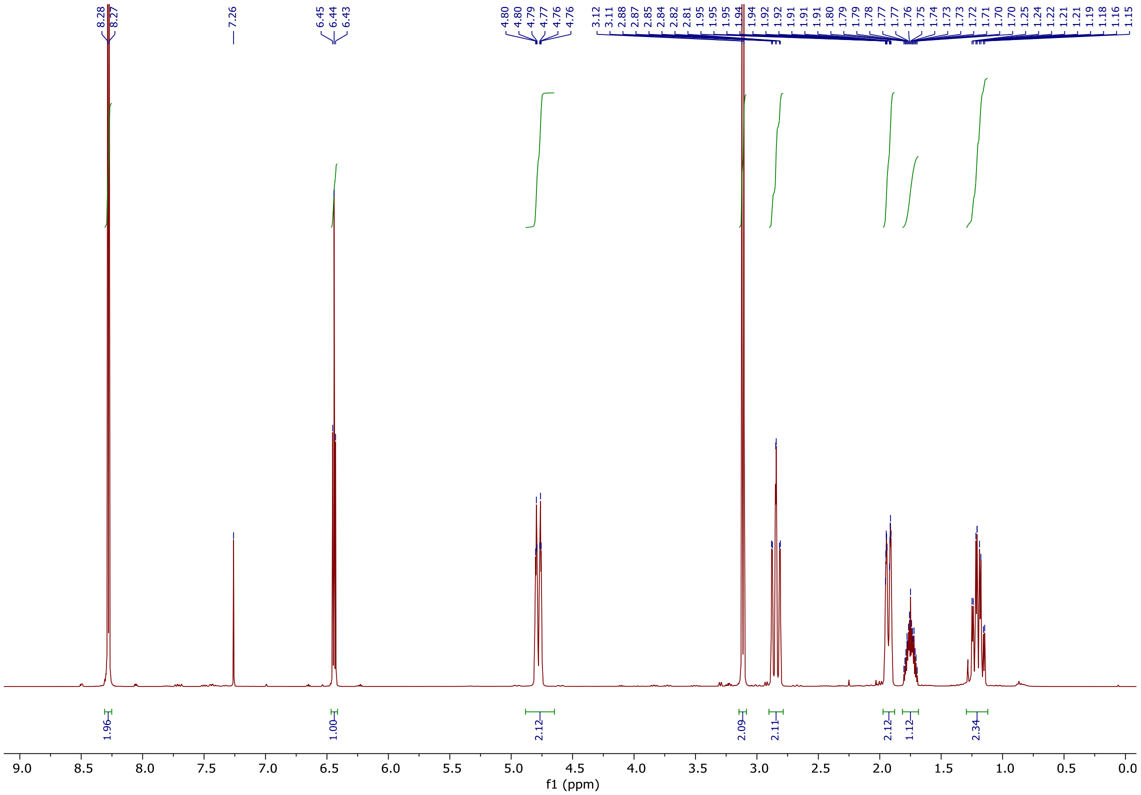

2-(4-(Iodomethyl)piperidin-1-yl)pyrimidine (**11m**) – $^{13}C\{^{1}H\}$ NMR spectrum ($CDCl_3$)

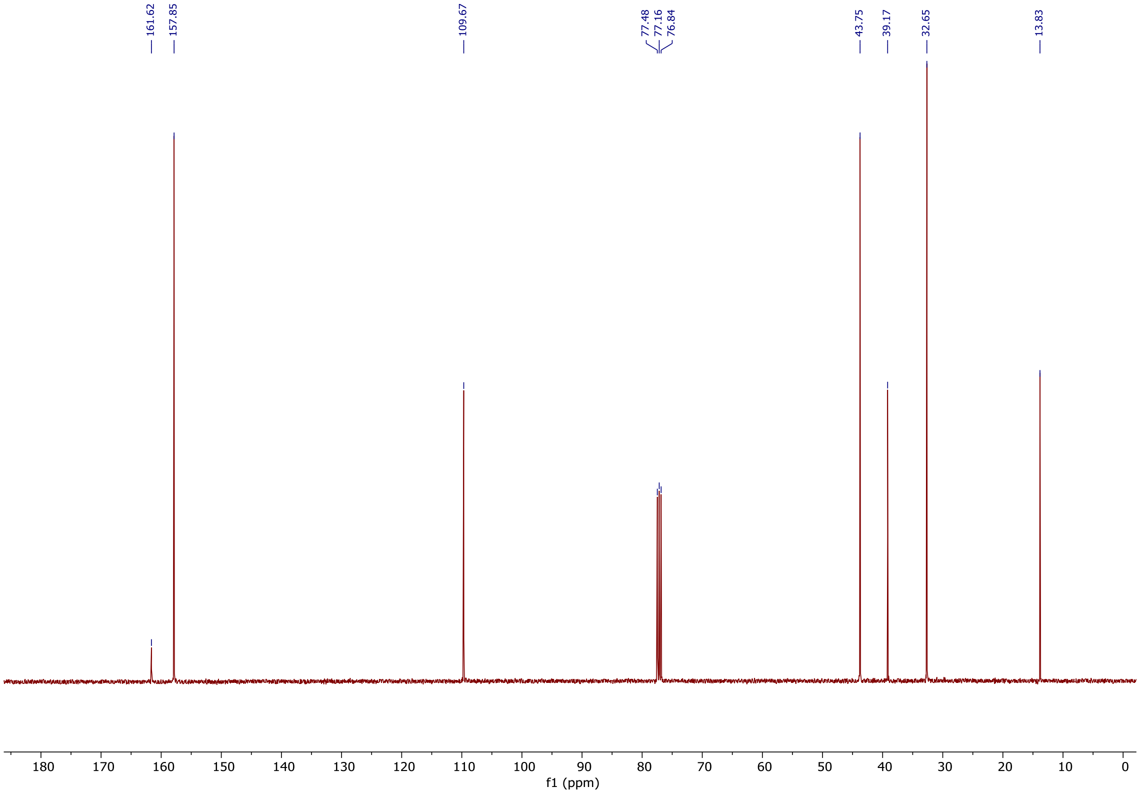

1-Iodo-4-(2-iodoethyl)benzene (**11n**) – ^{1}H NMR spectrum ($CDCl_3$)

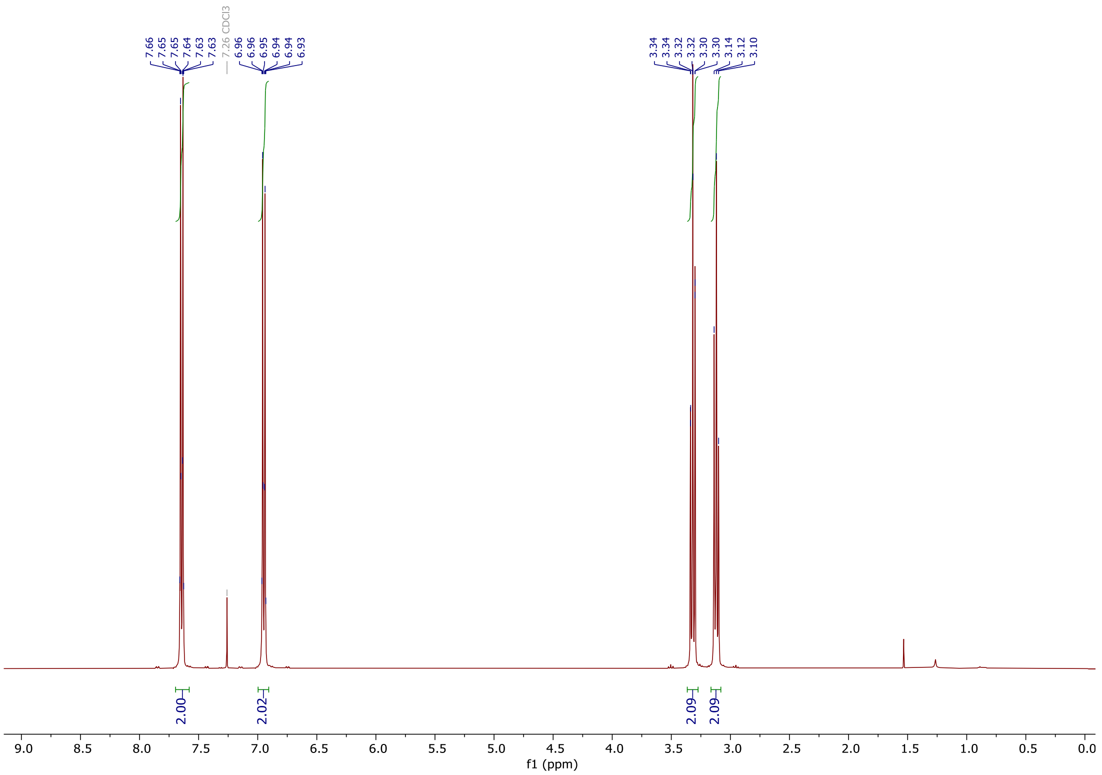

1-Iodo-4-(2-iodoethyl)benzene (**11n**) – $^{13}C\{^1H\}$ NMR spectrum ($CDCl_3$)

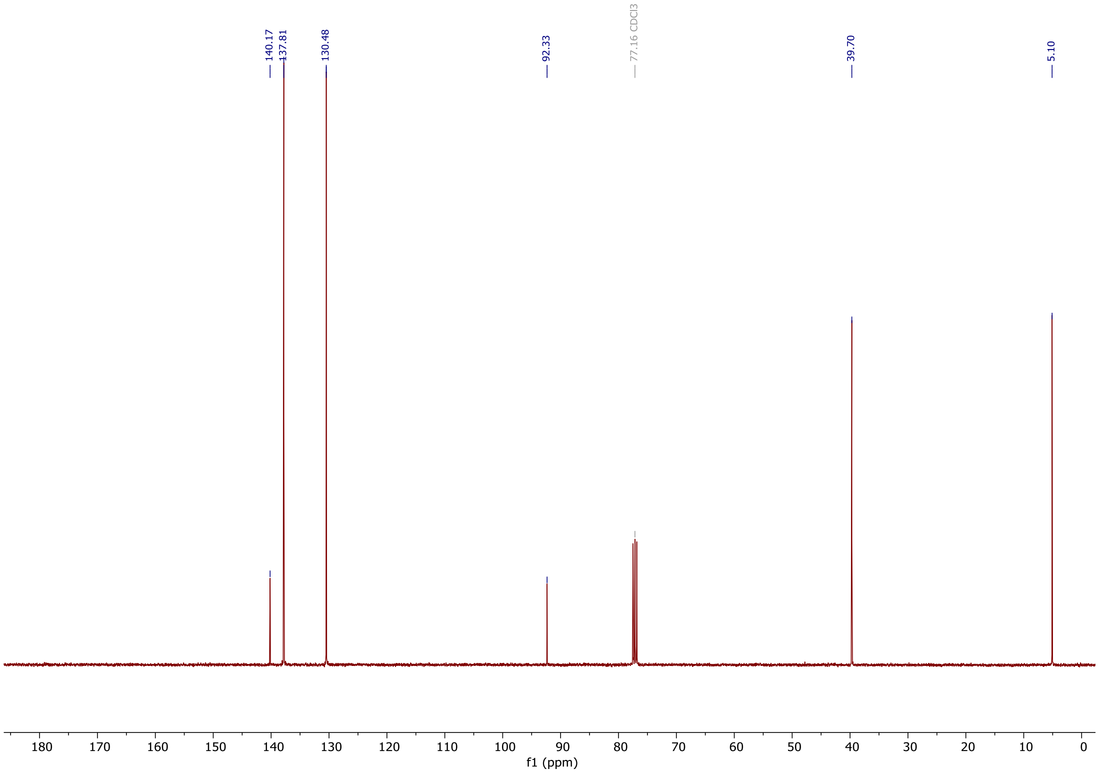

1-(Bromomethyl)-4-(hexyloxy)benzene (**11o**) – ^1H NMR spectrum ($CDCl_3$)

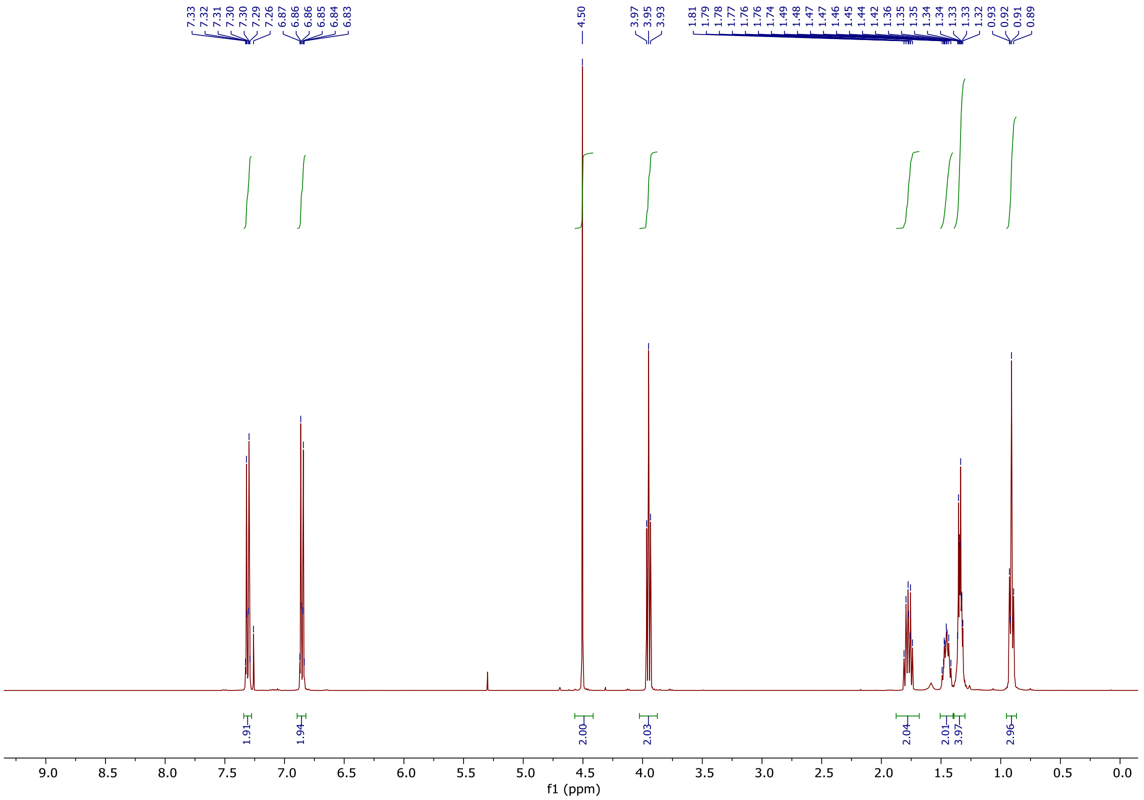

1-(Bromomethyl)-4-(hexyloxy)benzene (**11o**) – $^{13}C\{^1H\}$ NMR spectrum ($CDCl_3$)

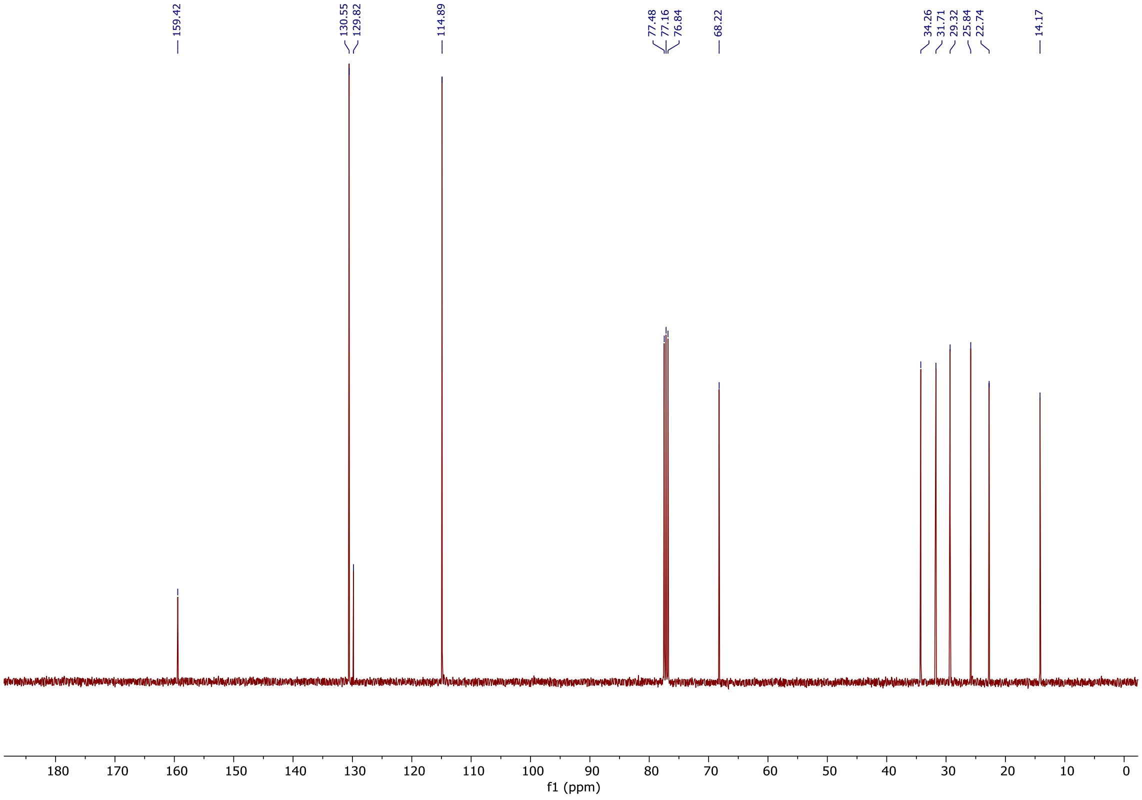

4-(3-iodopropyl)phenol (**11p**) – ^1H NMR spectrum ($CDCl_3$)

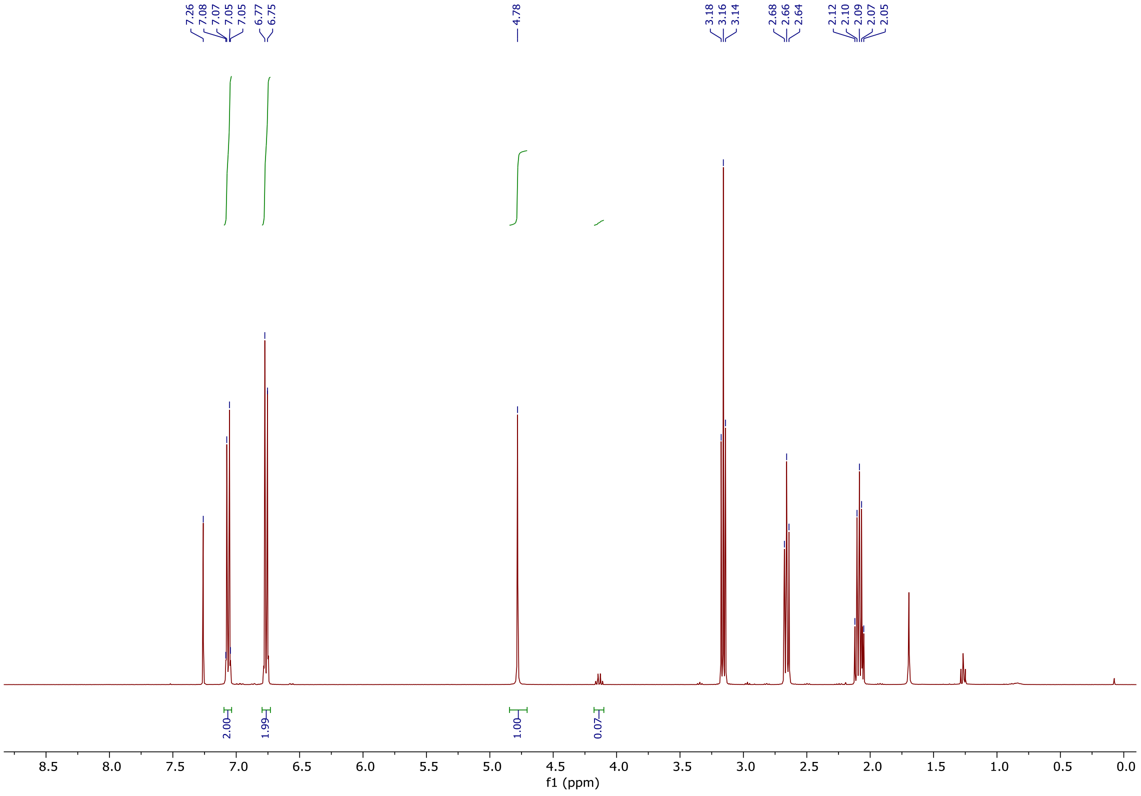

4-(3-iodopropyl)phenol (**11p**) – $^{13}C\{^1H\}$ NMR spectrum ($CDCl_3$)

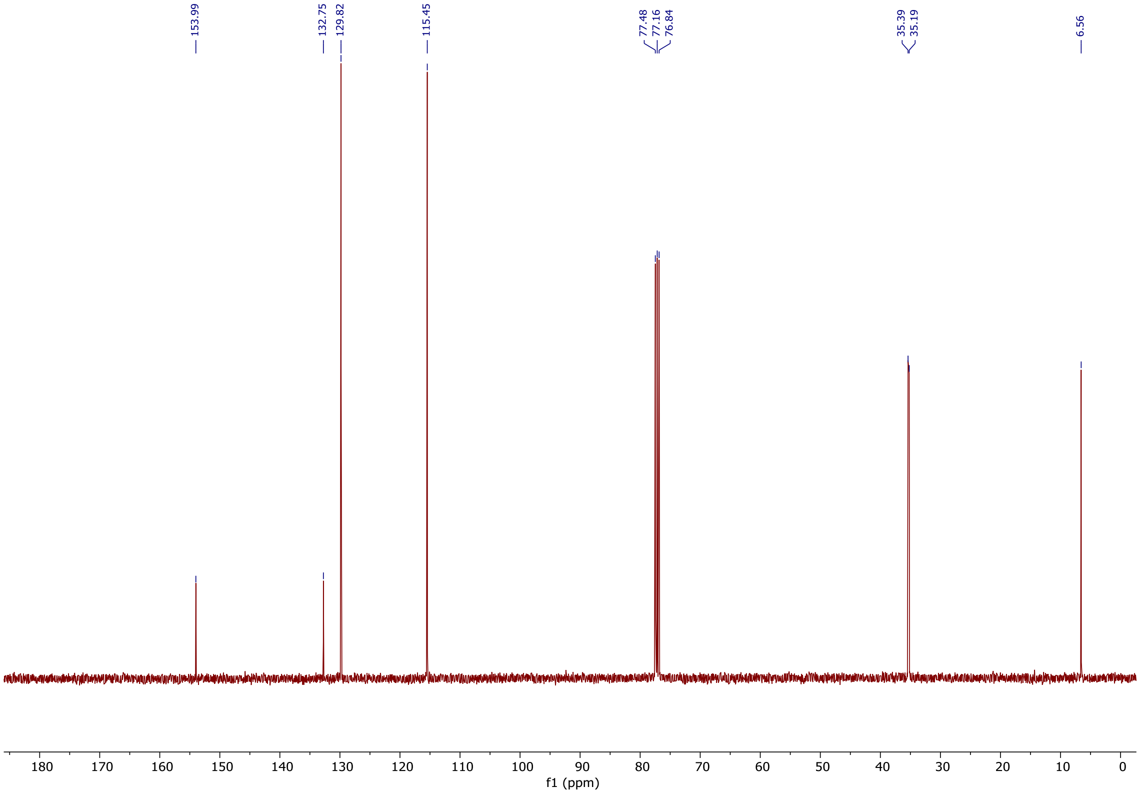

4-(4-Bromobutyl)benzoic acid (**11q**) – ^1H NMR spectrum ($CDCl_3$)

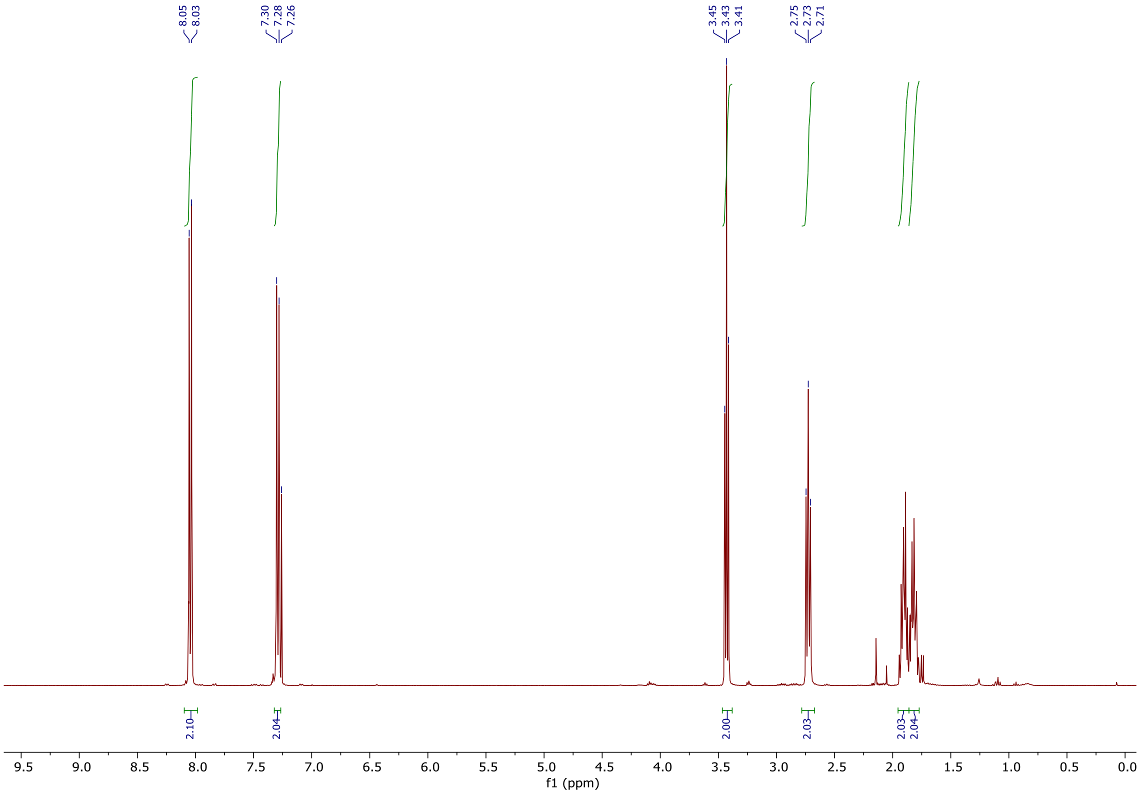

4-(4-Bromobutyl)benzoic acid (**11q**) – $^{13}C\{^{1}H\}$ NMR spectrum ($CDCl_3$)

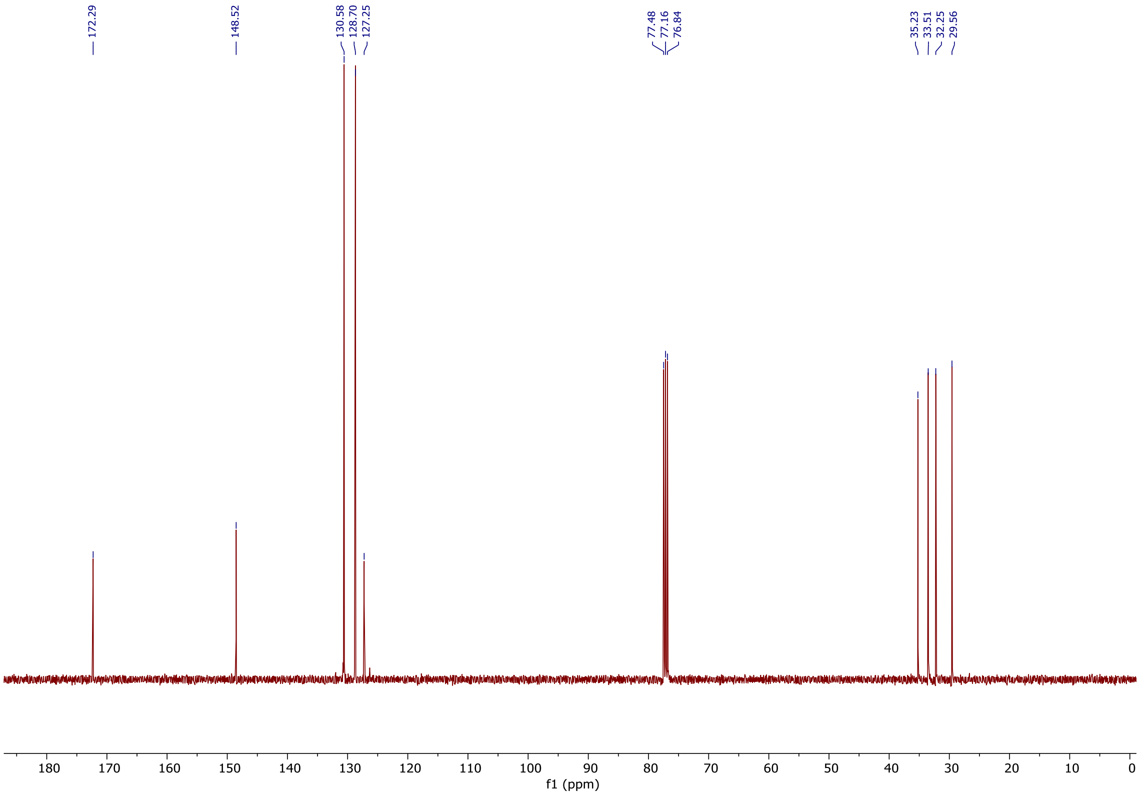

5-(4-Bromobutoxy)-1*H*-pyrrolo[2,3-b]pyridine (**11r**) – ^{1}H NMR spectrum ($CDCl_3$)

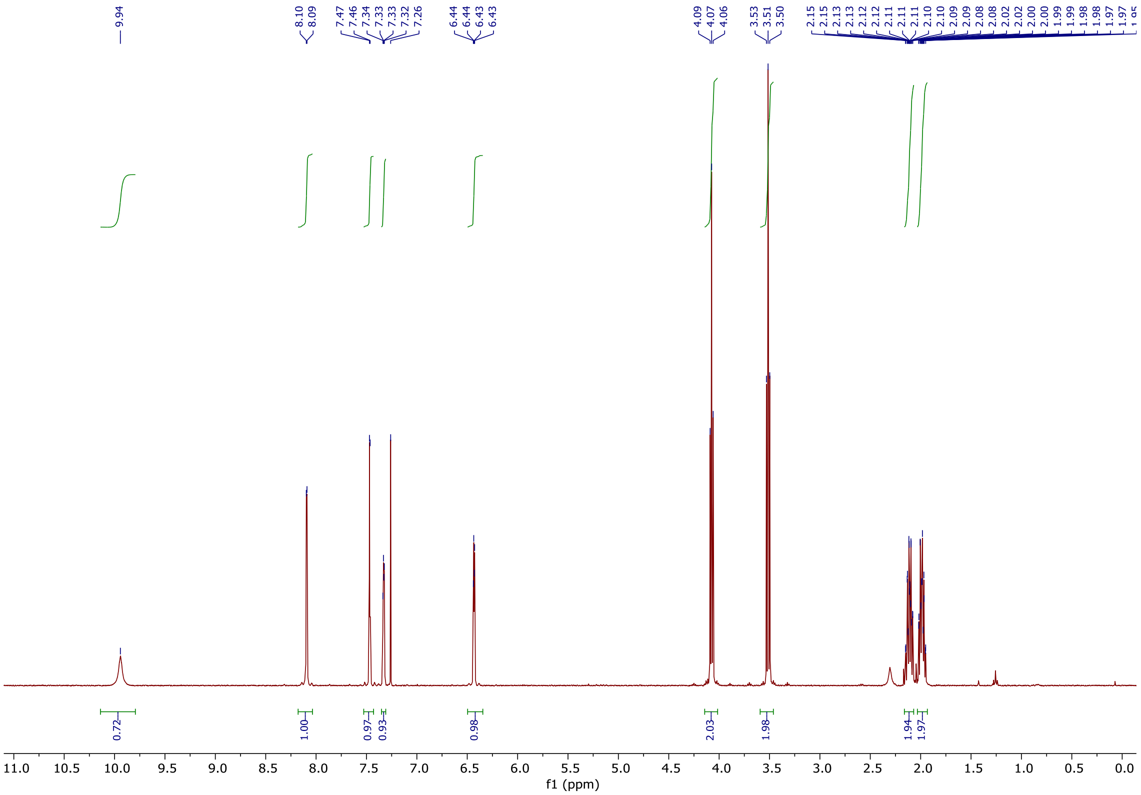

5-(4-Bromobutoxy)-1*H*-pyrrolo[2,3-b]pyridine (**11r**) – $^{13}C\{^{1}H\}$ NMR spectrum ($CDCl_3$)

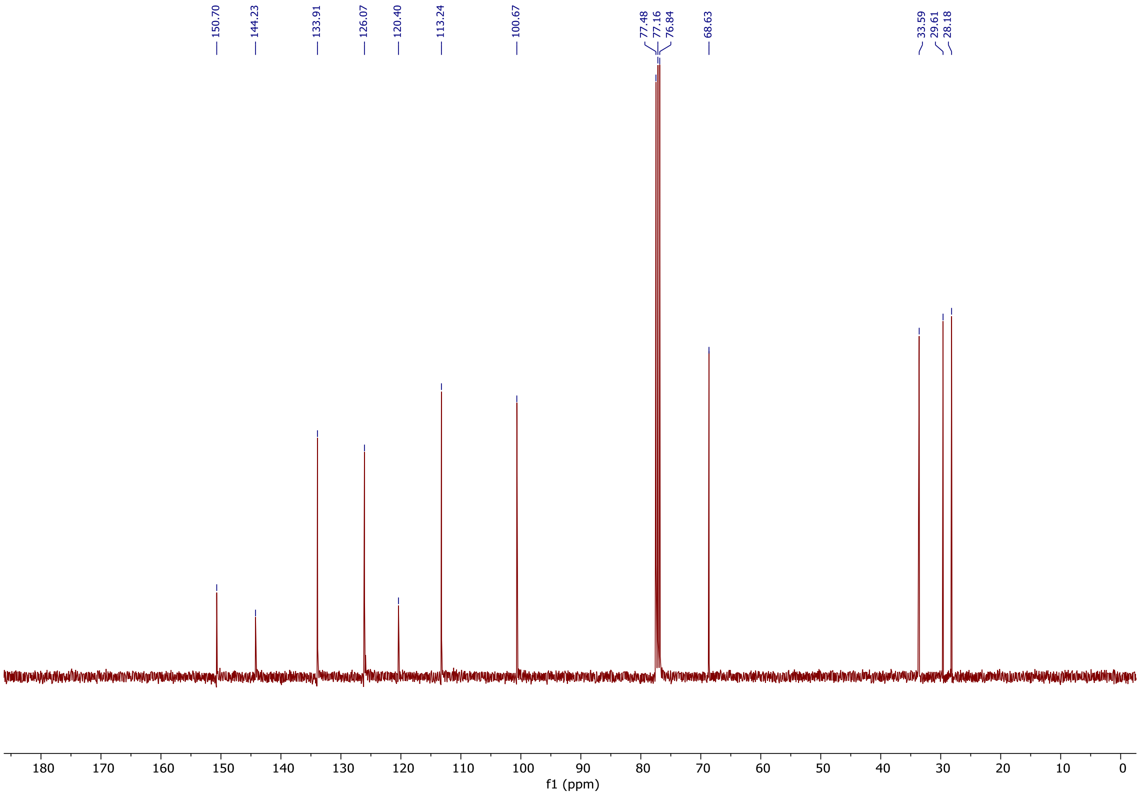

Alkyl iodide **11s** – ^{1}H NMR spectrum ($CDCl_3$)

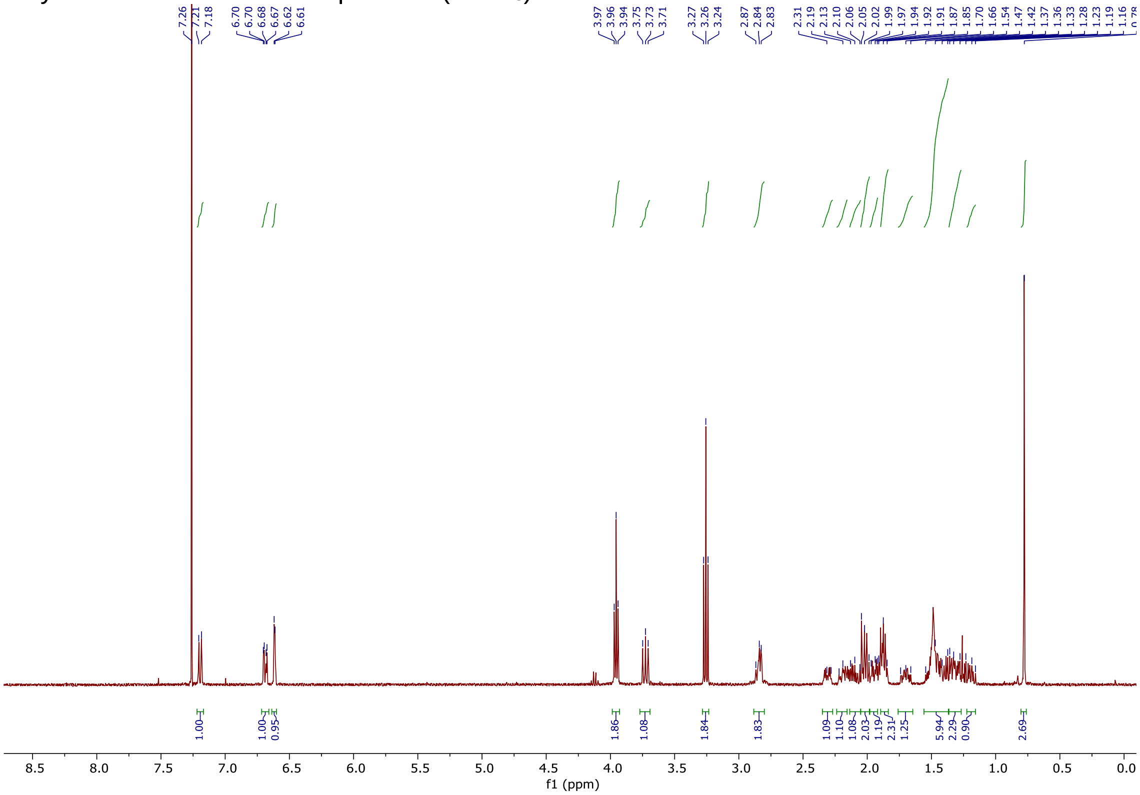

Alkyl iodide **11s** – $^{13}C\{^1H\}$ NMR spectrum ($CDCl_3$)

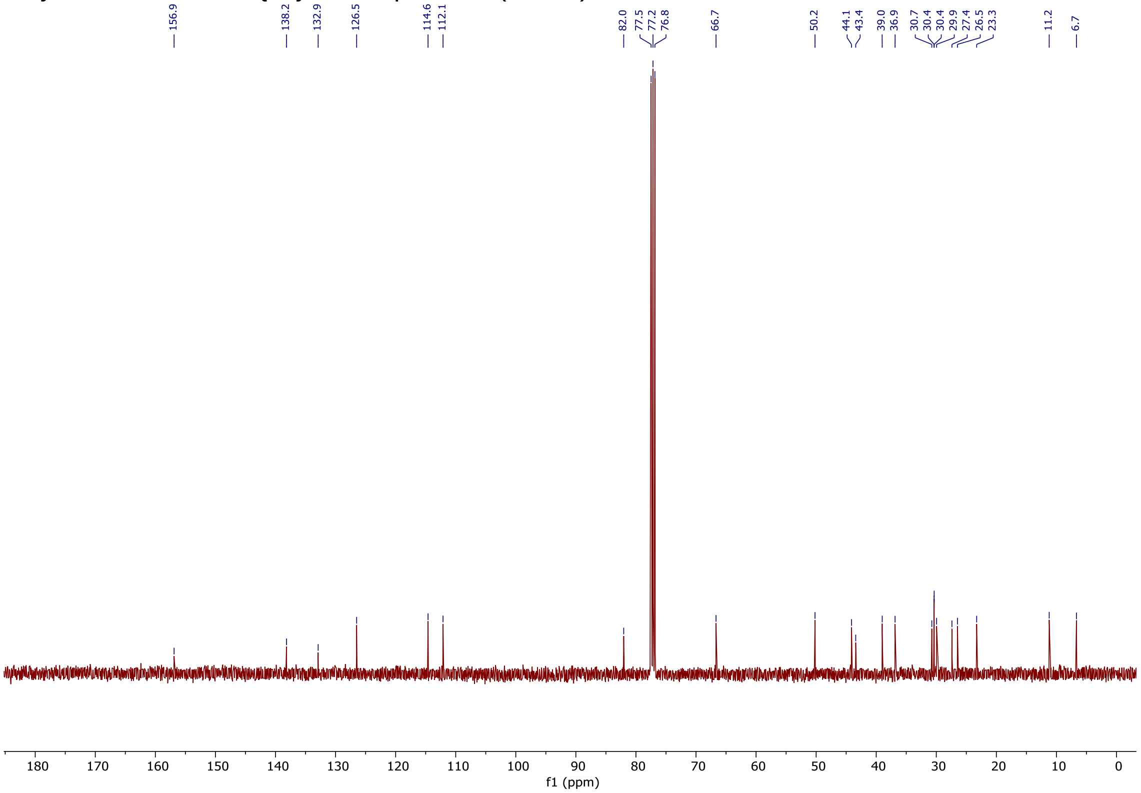

Alkyl bromide **11t** – ^1H NMR spectrum ($CDCl_3$)

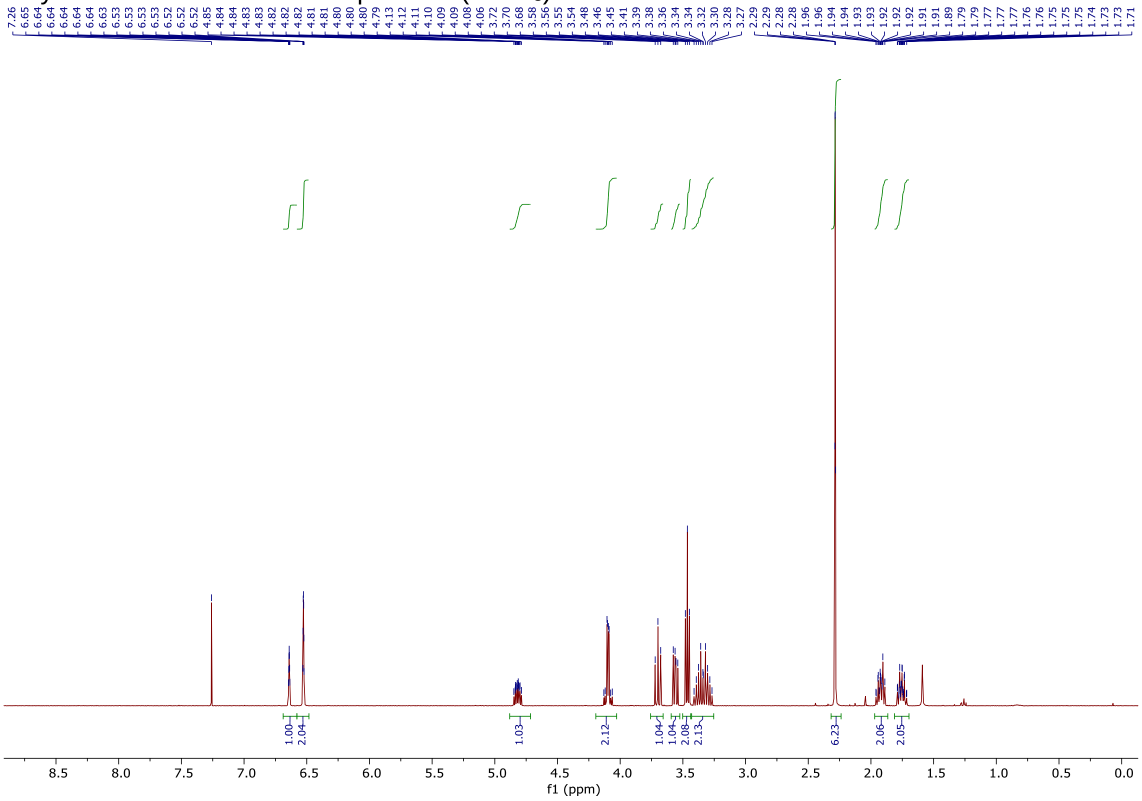

Alkyl bromide **11t** – $^{13}C\{^{1}H\}$ NMR spectrum ($CDCl_3$)

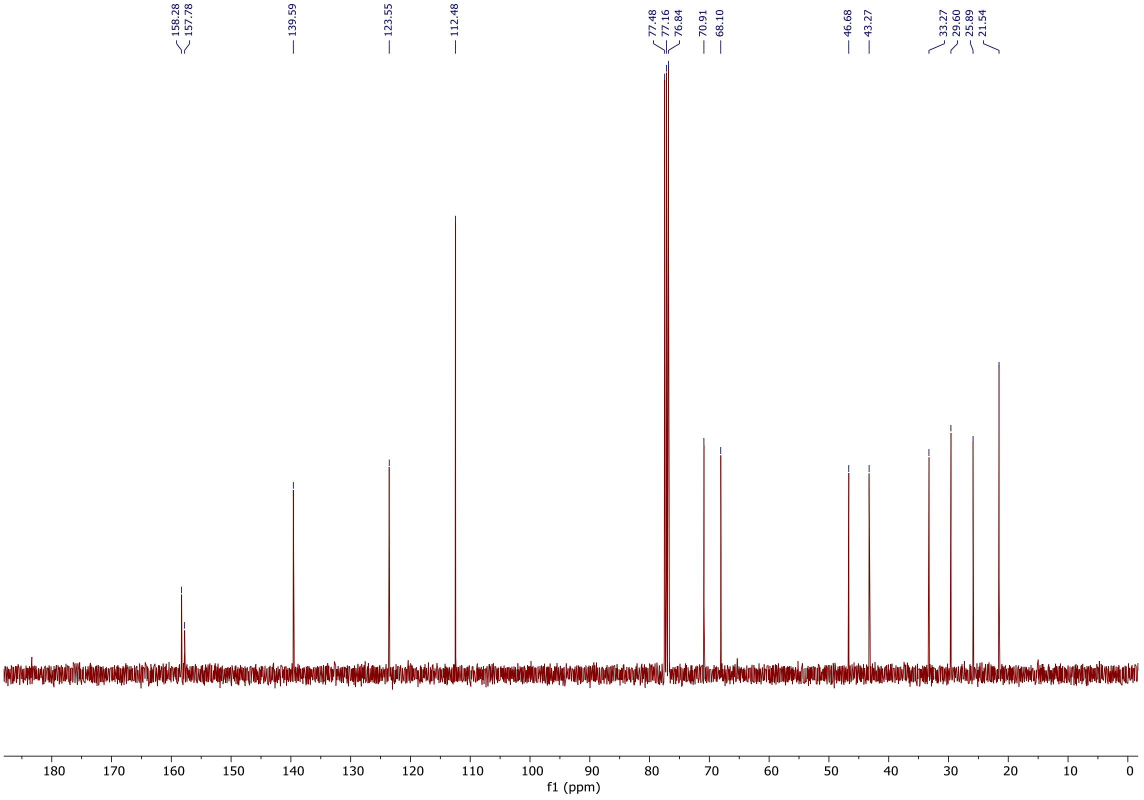

Alkyl bromide **11u** – ^{1}H NMR spectrum ($CDCl_3$)

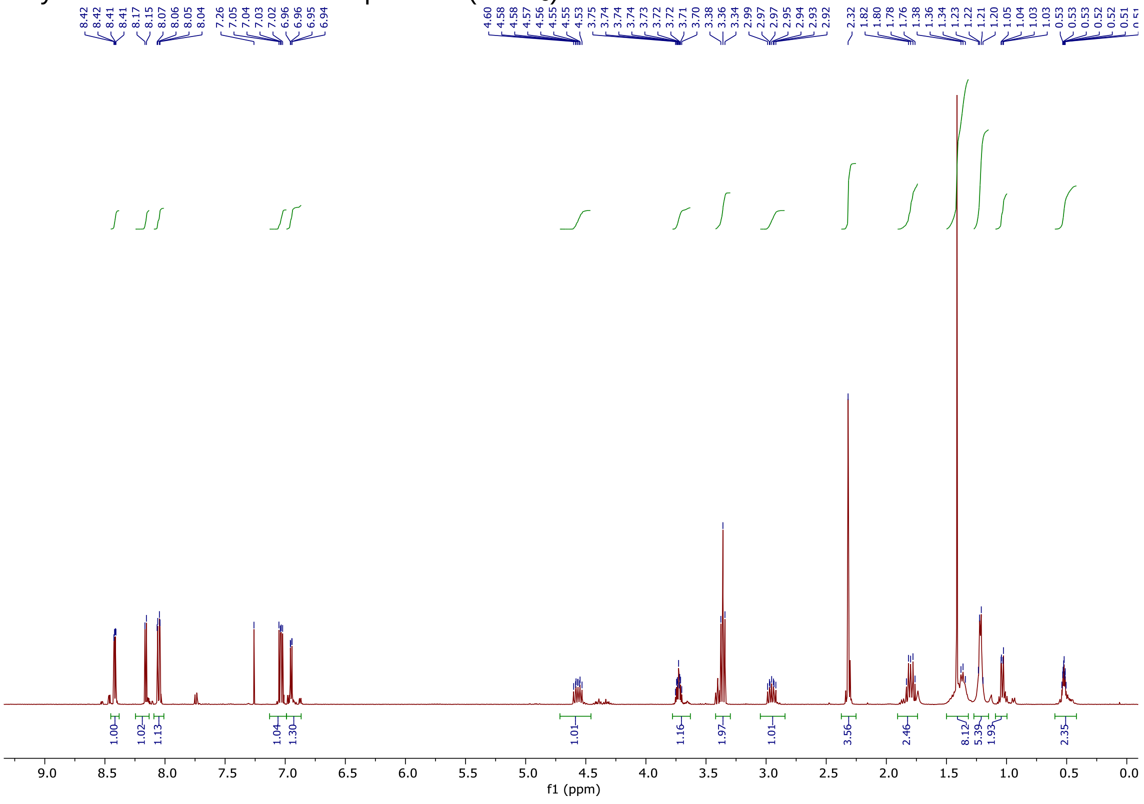

Alkyl bromide **11u** – $^{13}C\{^{1}H\}$ NMR spectrum ($CDCl_3$)

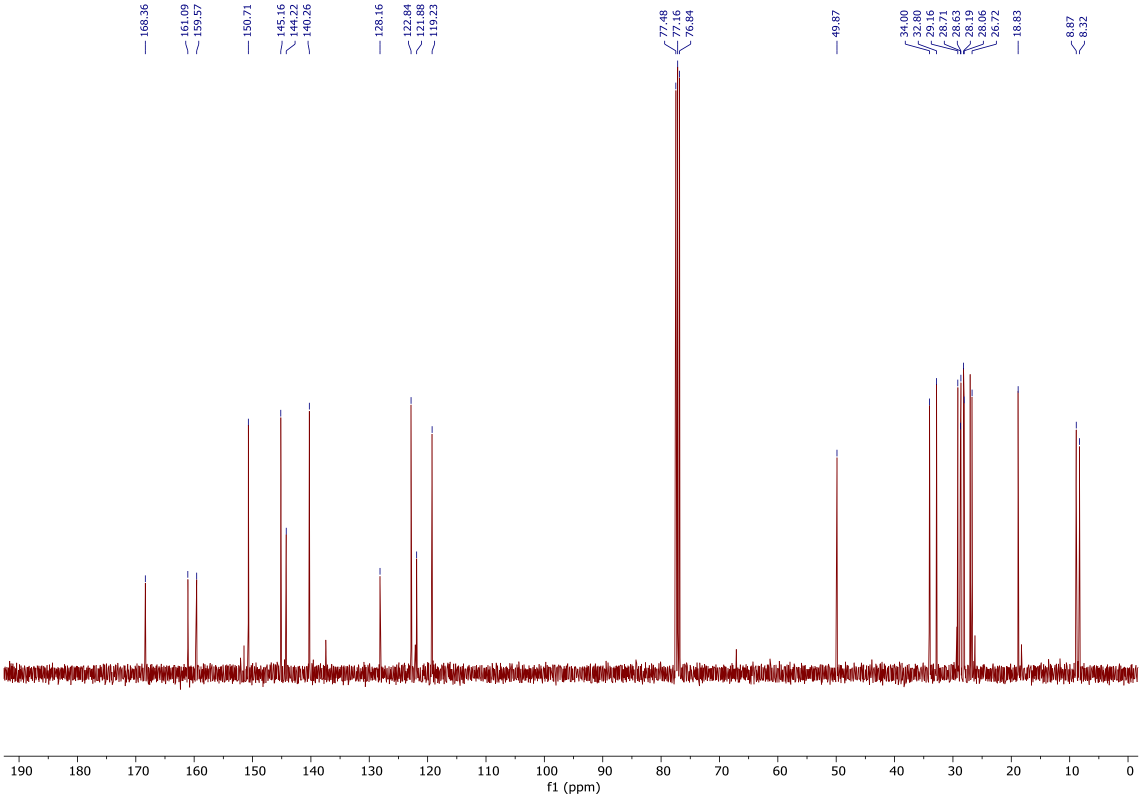

(6-Cyclopentylhexyl)benzene (**12a**) – ^{1}H NMR spectrum ($CDCl_3$)

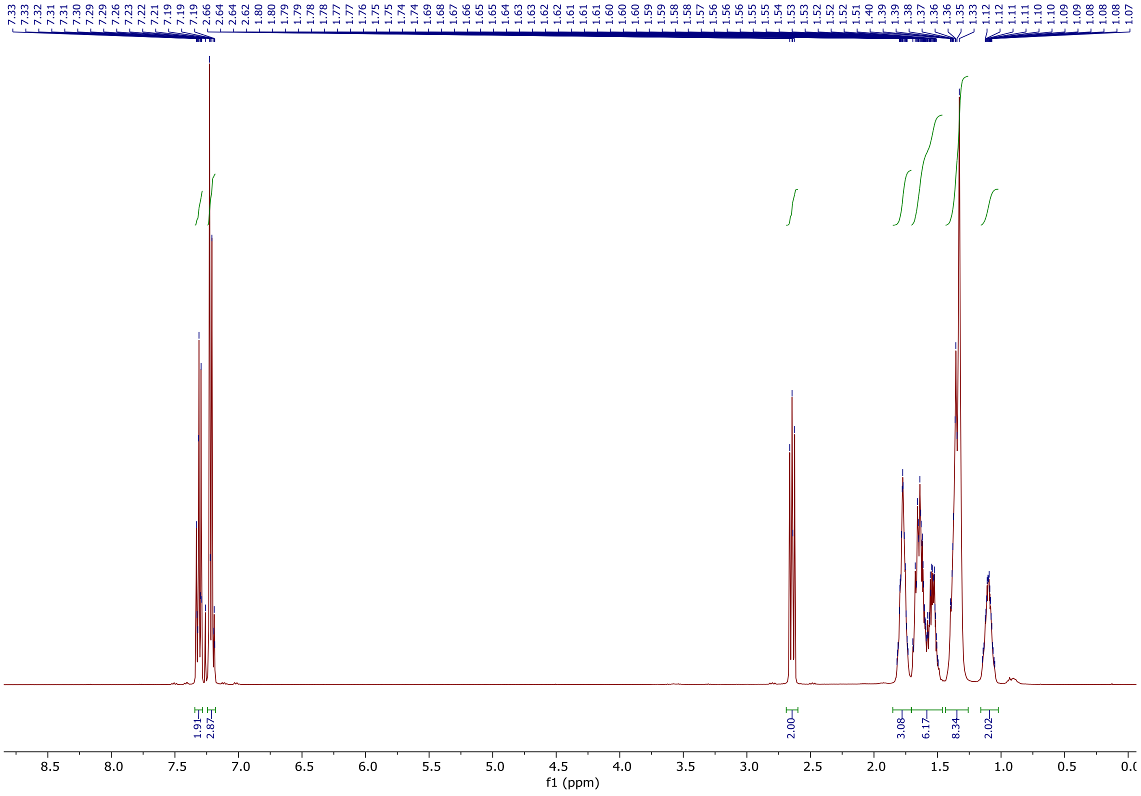

(6-Cyclopentylhexyl)benzene (**12a**) – $^{13}C\{^{1}H\}$ NMR spectrum ($CDCl_3$)

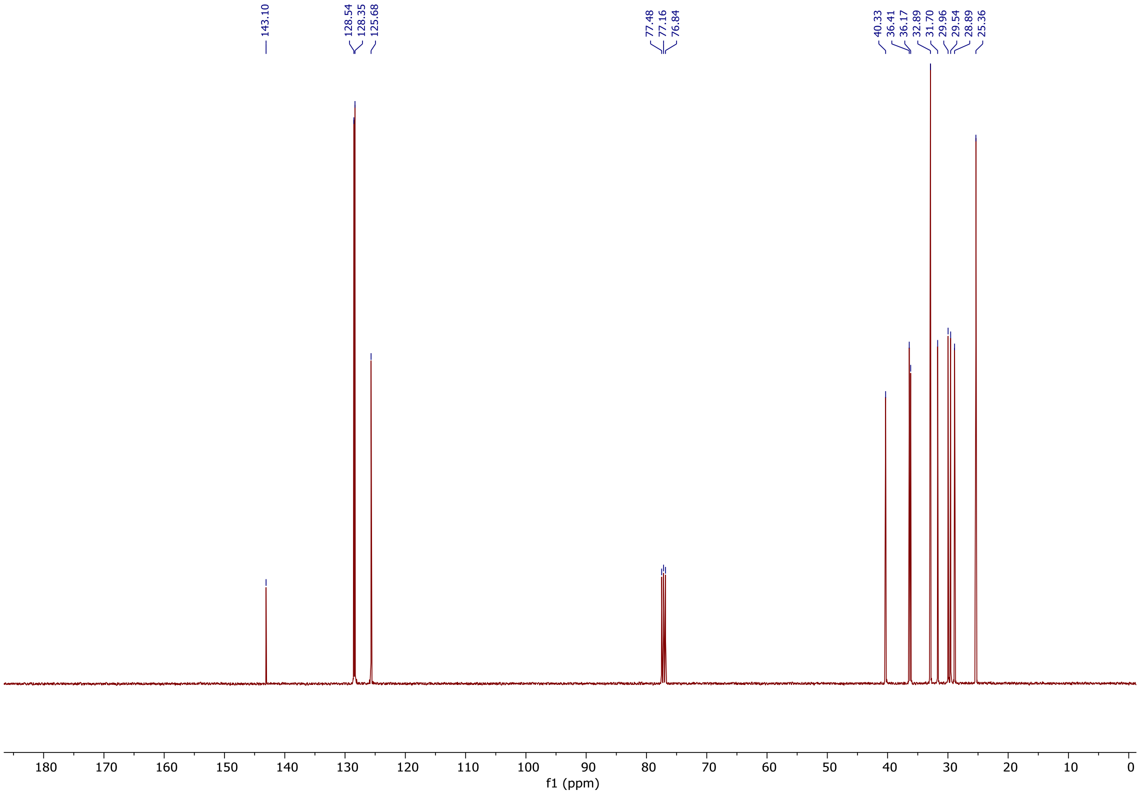

(4-Cyclopentylbutoxy)benzene (**12b**) – ^{1}H NMR spectrum ($CDCl_3$)

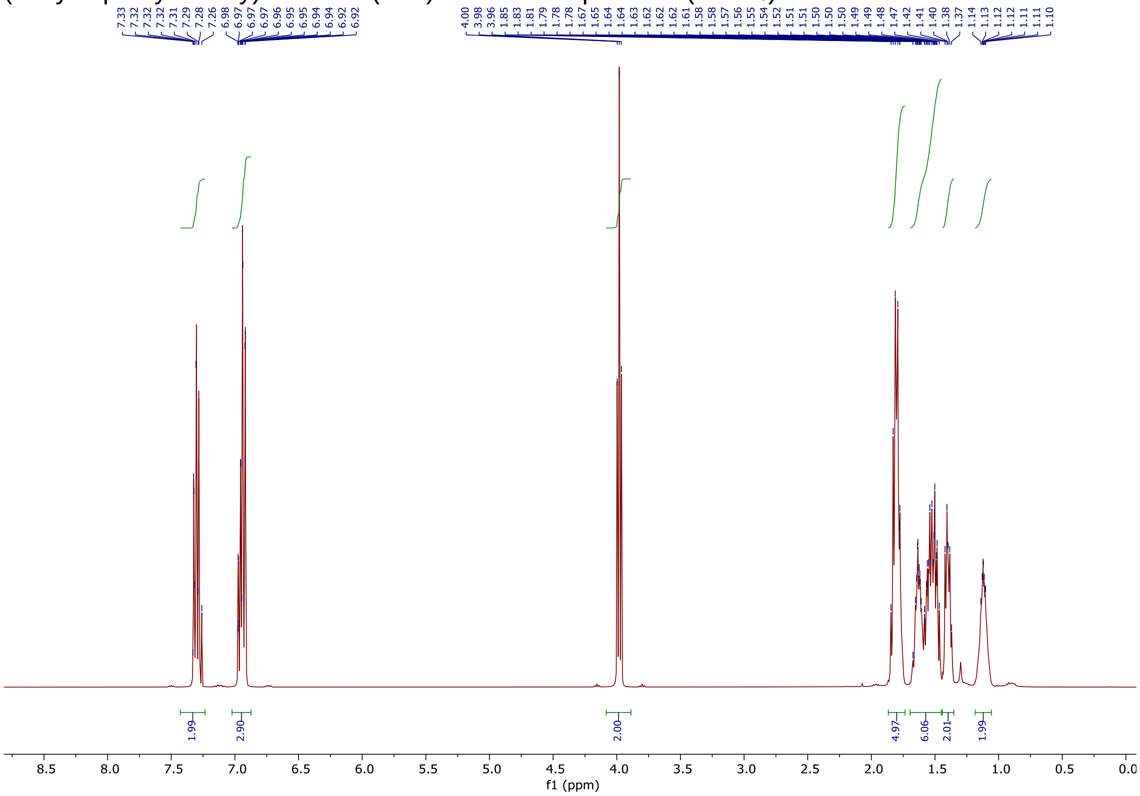

(4-Cyclopentylbutoxy)benzene (**12b**) – $^{13}C\{^{1}H\}$ NMR spectrum ($CDCl_3$)

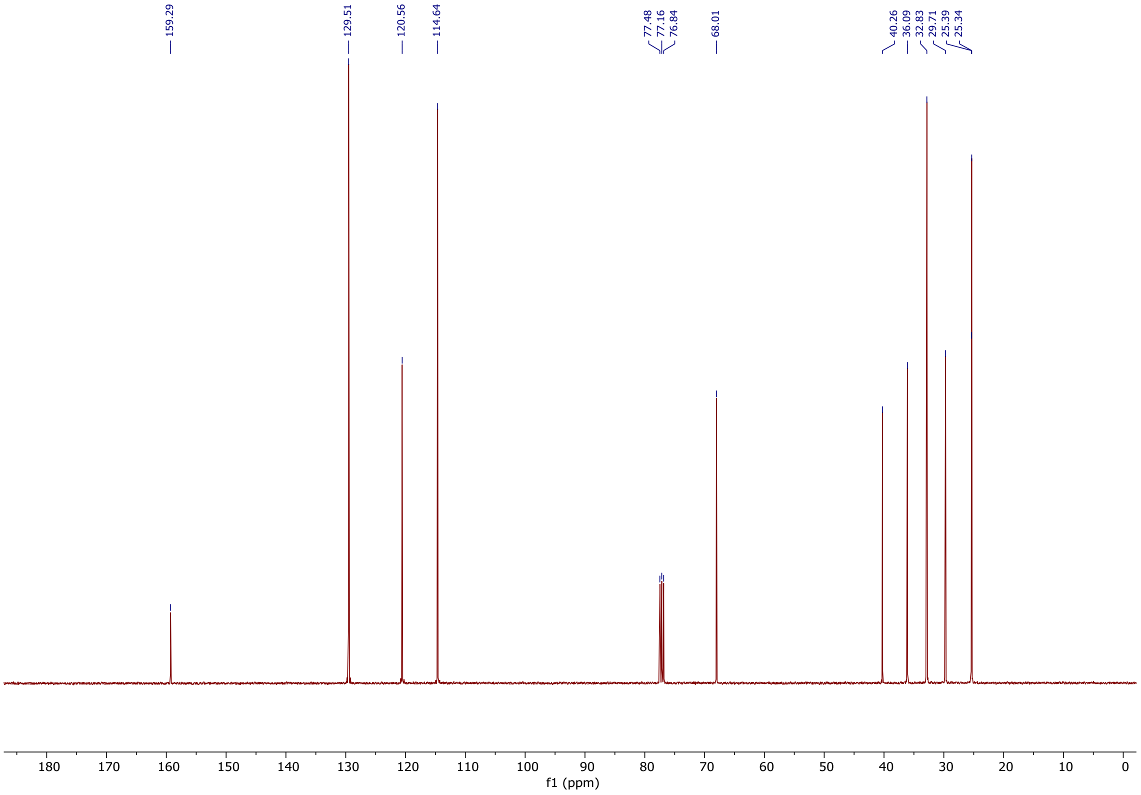

tert-Butyl(3-cyclopentylpropoxy)diphenylsilane (**12c**) – ^{1}H NMR spectrum ($CDCl_3$)

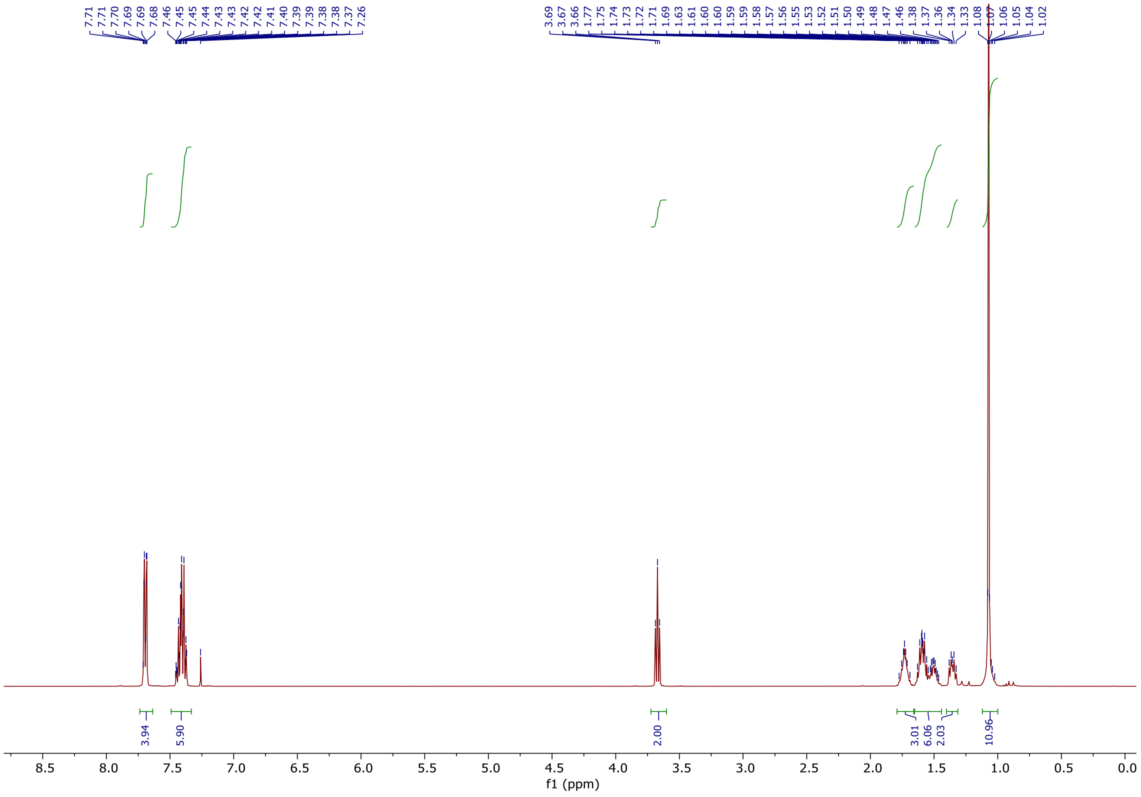

tert-Butyl(3-cyclopentylpropoxy)diphenylsilane (**12c**) – $^{13}C\{^{1}H\}$ NMR spectrum ($CDCl_3$)

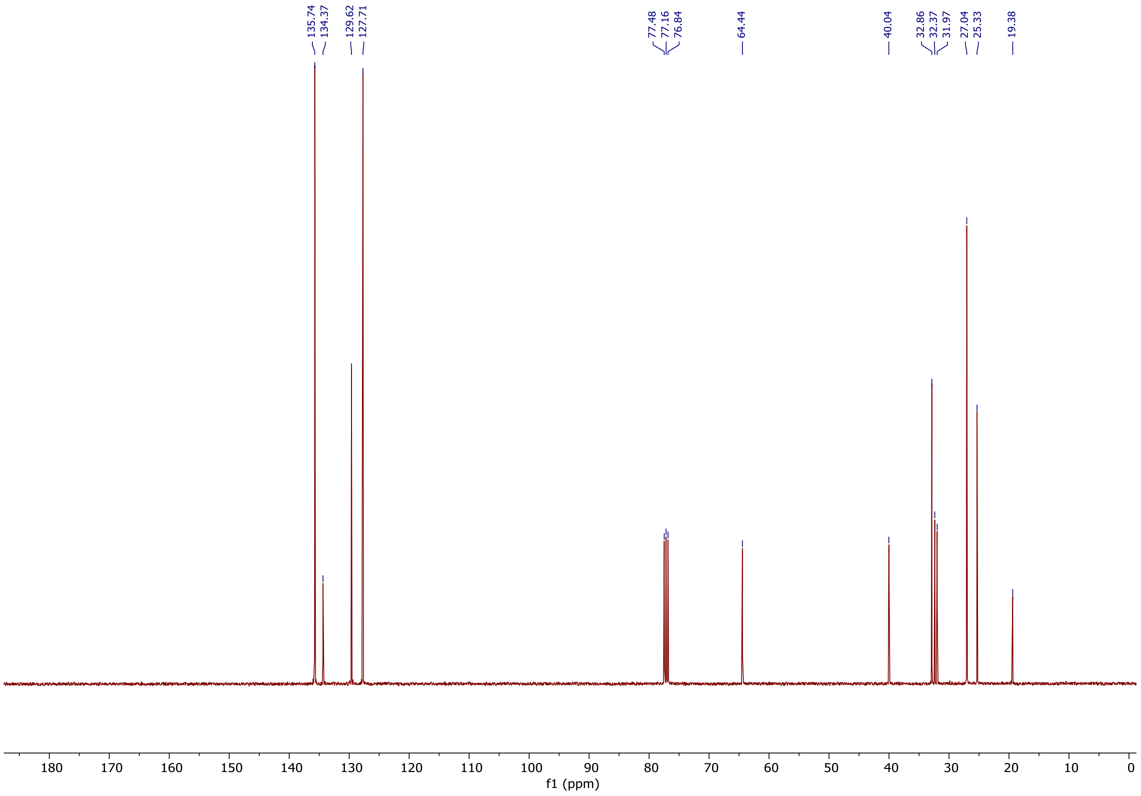

5-(3-Cyclopentylpropyl)benzo[d][1,3]dioxole (**12d**) – ^{1}H NMR spectrum ($CDCl_3$)

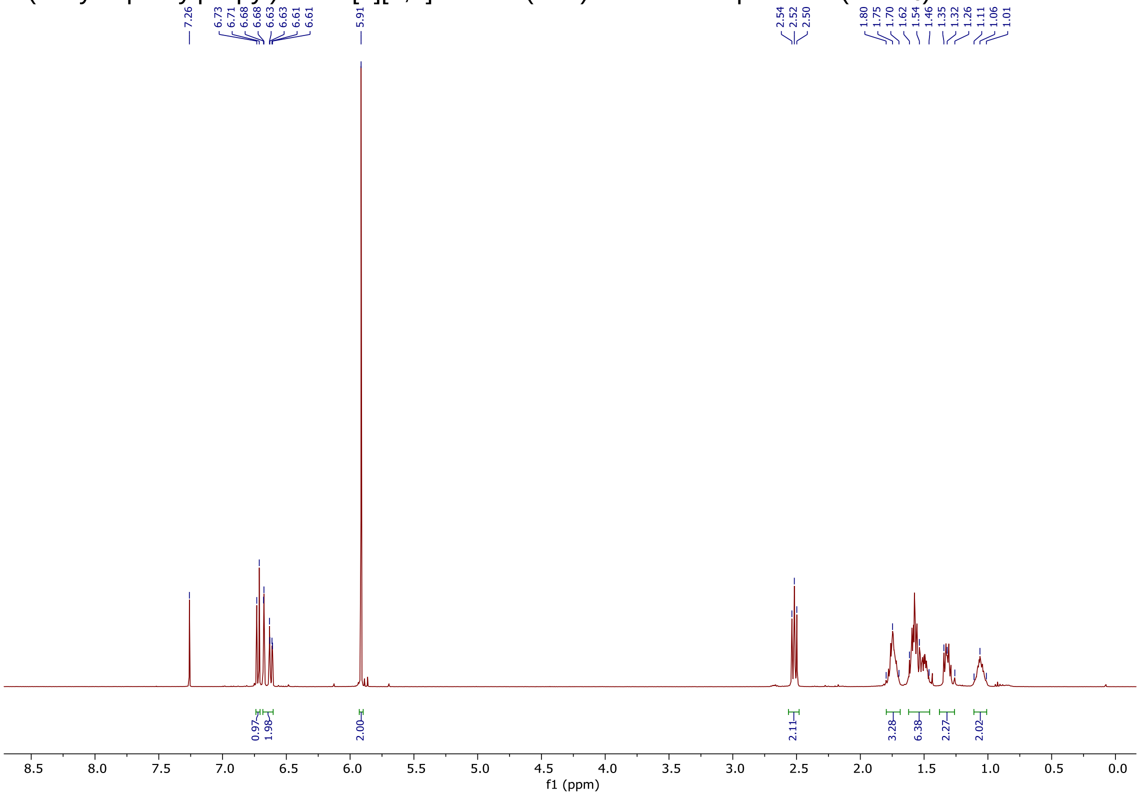

5-(3-Cyclopentylpropyl)benzo[d][1,3]dioxole (**12d**) – $^{13}C\{^1H\}$ NMR spectrum ($CDCl_3$)

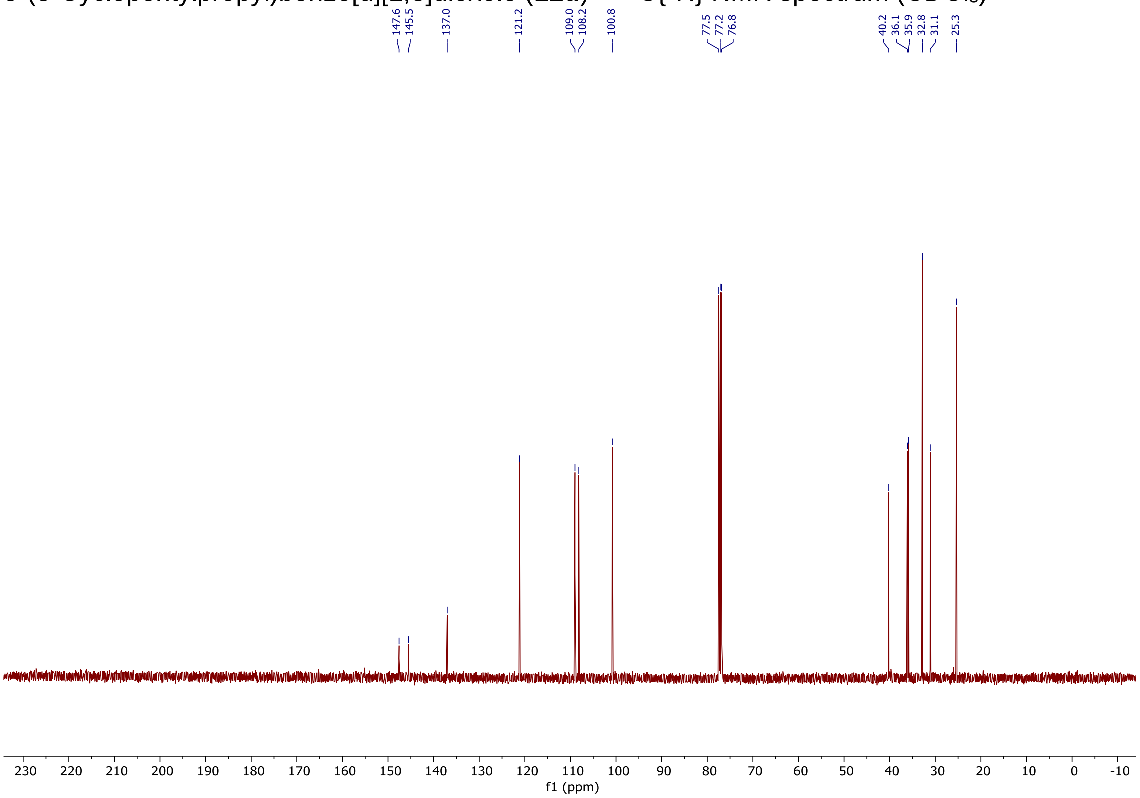

Amide **12e** – ^1H NMR spectrum ($CDCl_3$; measured at ca. 293 K)

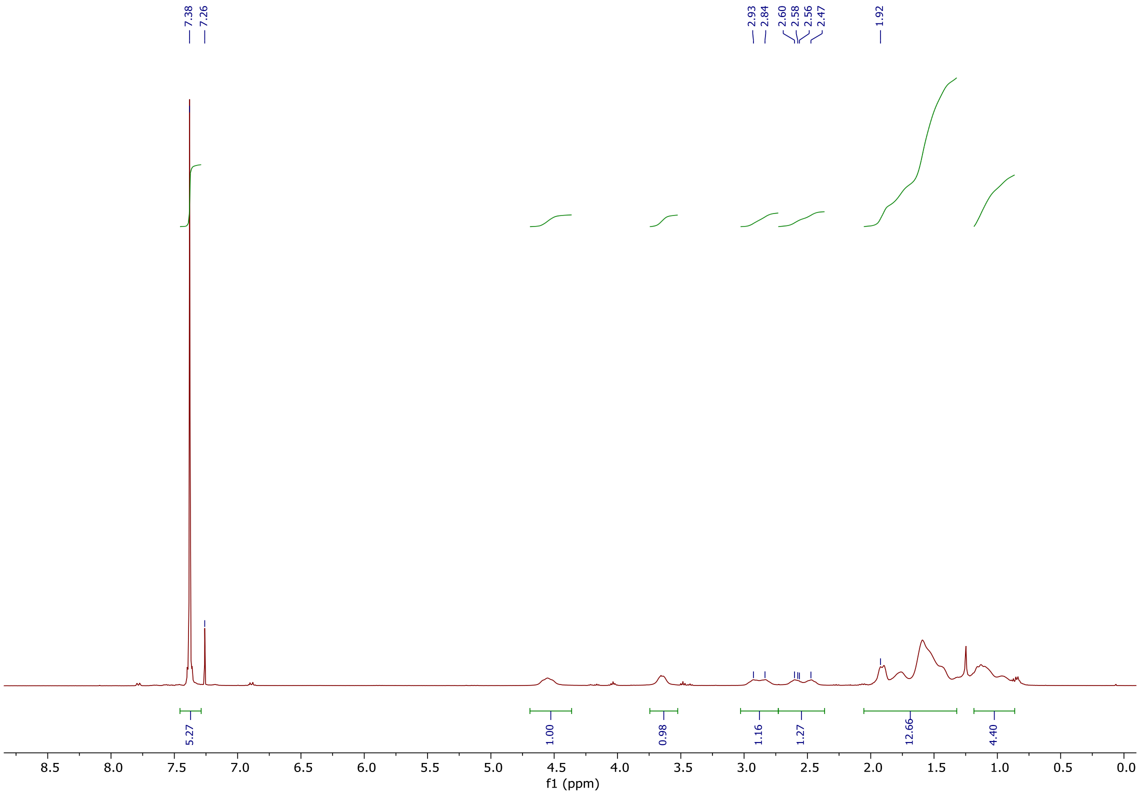

Amide **12e** – $^{13}C\{^{1}H\}$ NMR spectrum ($CDCl_3$; measured at ca. 293 K)

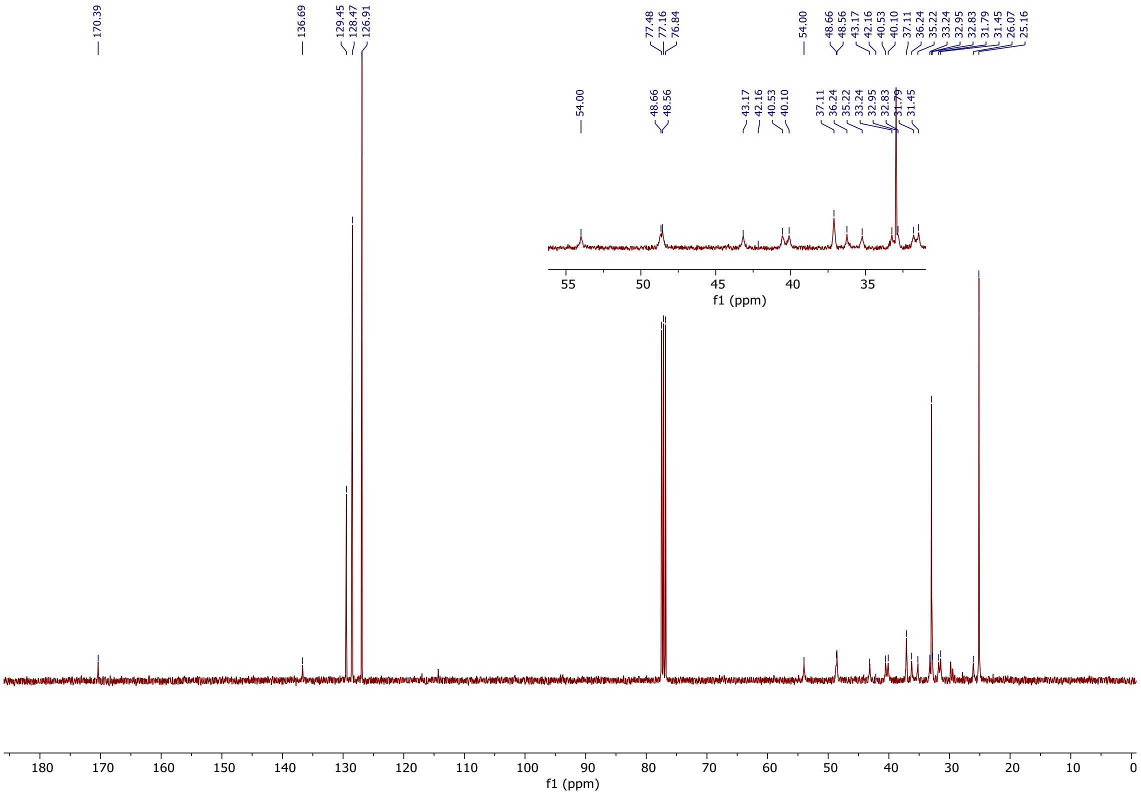

Amide **12e** – ^{1}H NMR spectrum ($CDCl_3$; measured at 328 K)

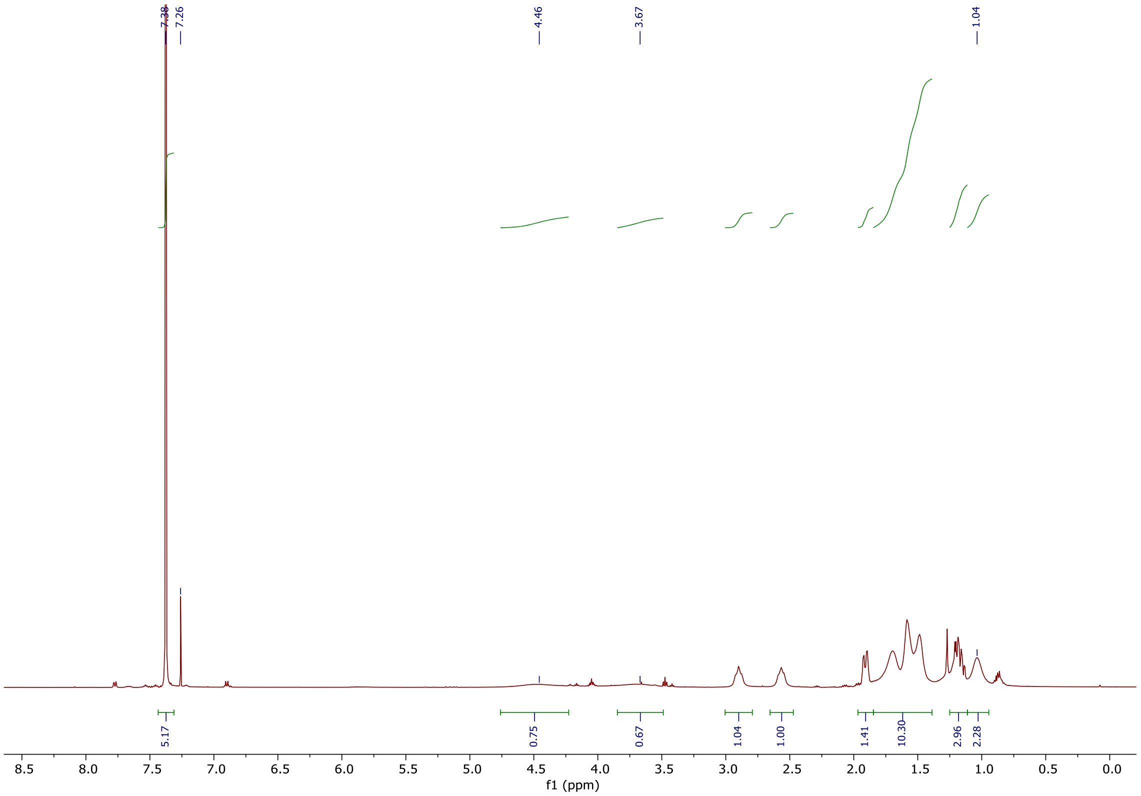

Amide **12e** – $^{13}C\{^{1}H\}$ NMR spectrum ($CDCl_3$; measured at 328 K)

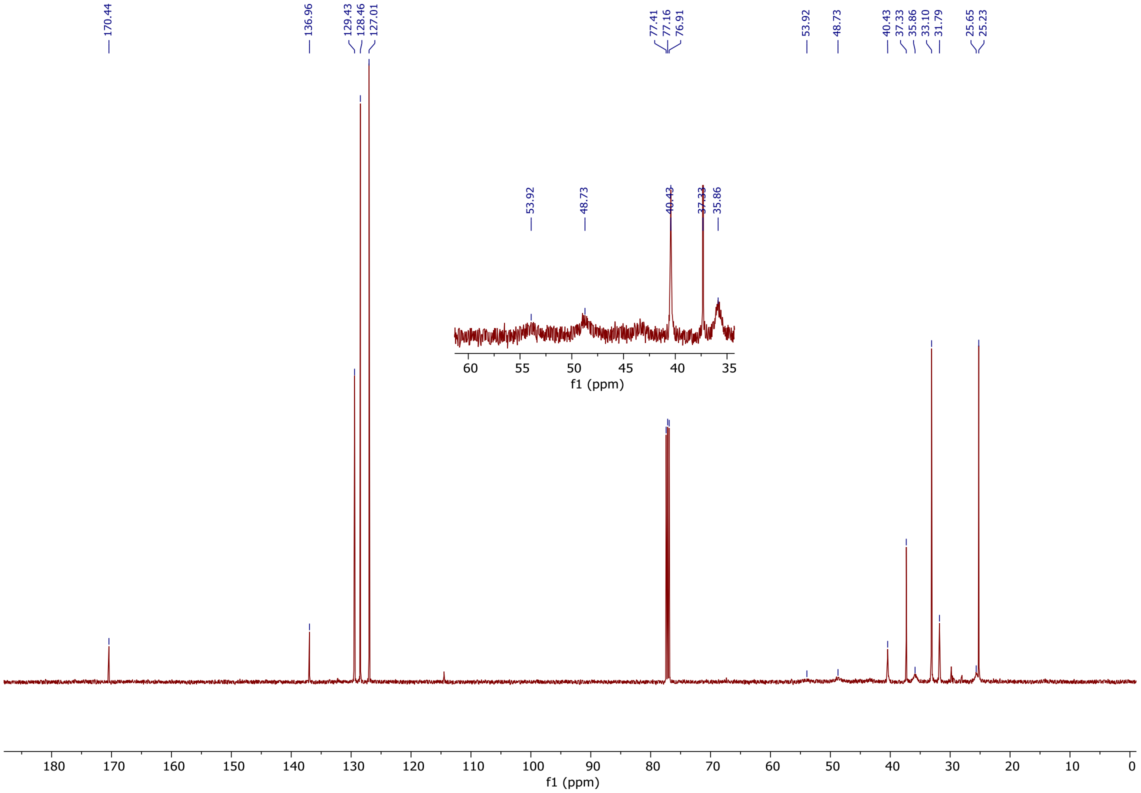

tert-Butyl 4-(cyclopentylmethyl)piperidine-1-carboxylate (**12f**) – ^{1}H NMR spectrum ($CDCl_3$)

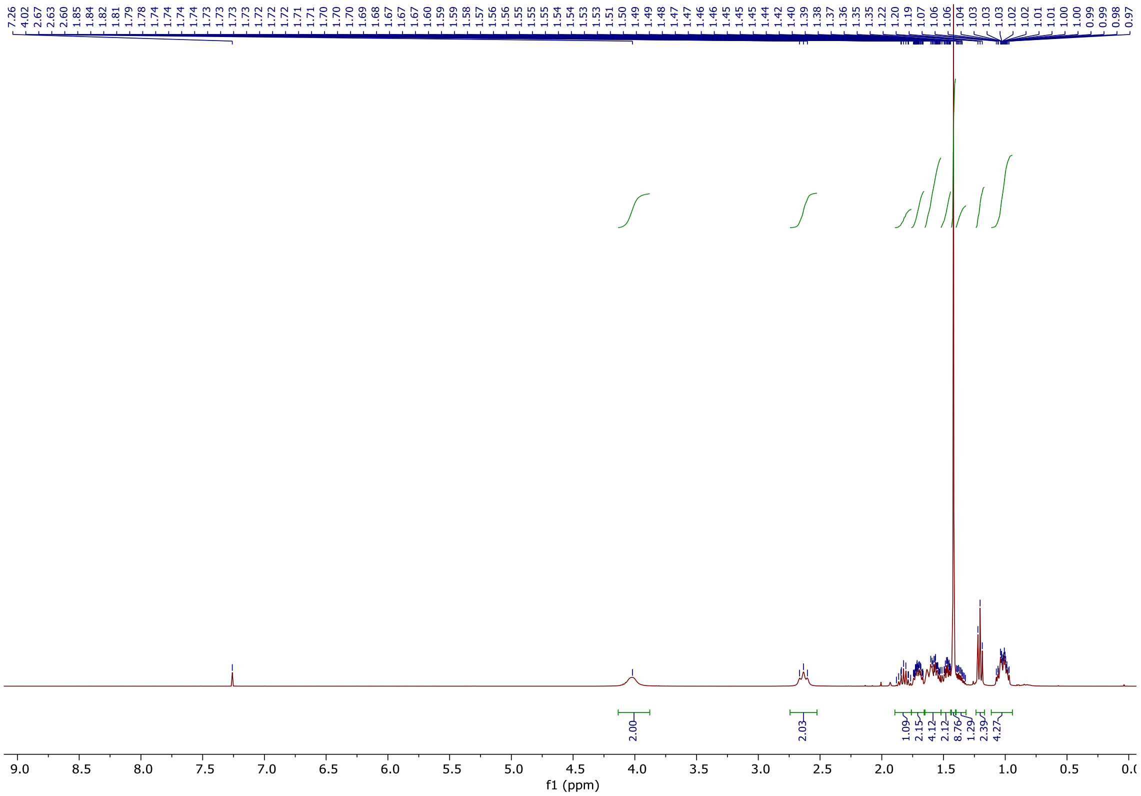

tert-Butyl 4-(cyclopentylmethyl)piperidine-1-carboxylate (**12f**) – $^{13}C\{^1H\}$ NMR spectrum ($CDCl_3$)

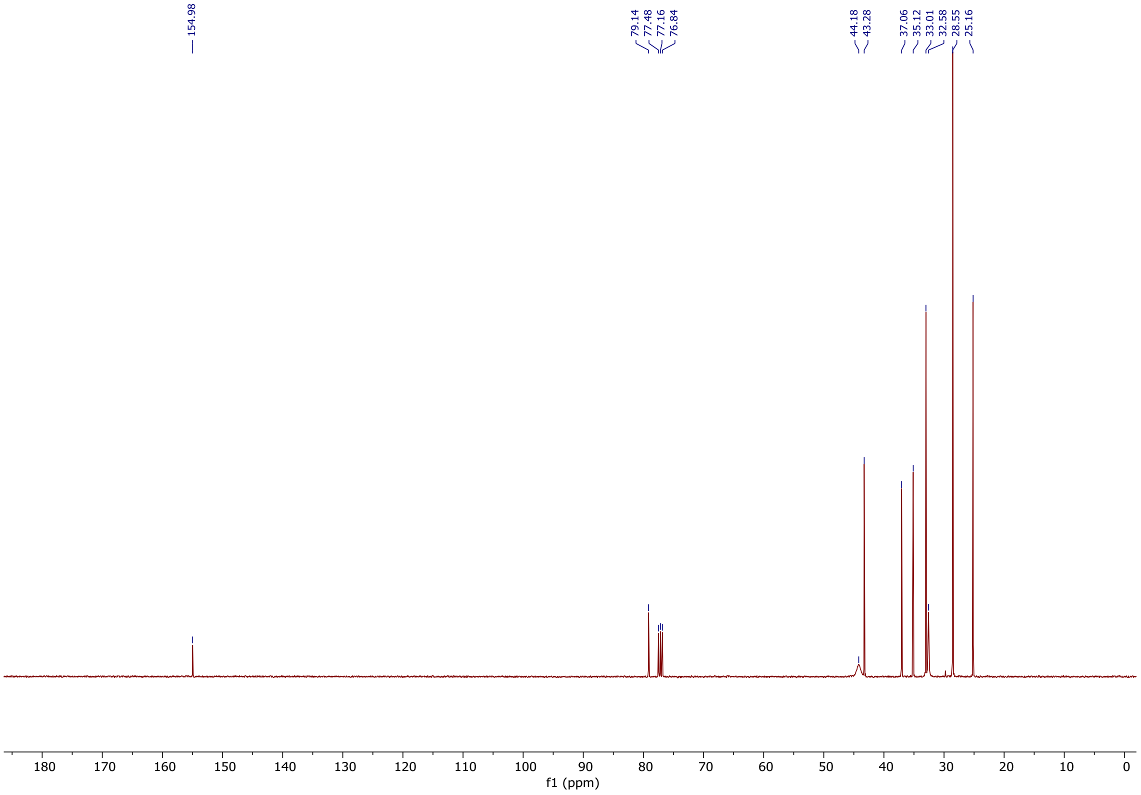

1-(3-Cyclopentylpropyl)-4-iodobenzene (**12g**) – ^1H NMR spectrum ($CDCl_3$)

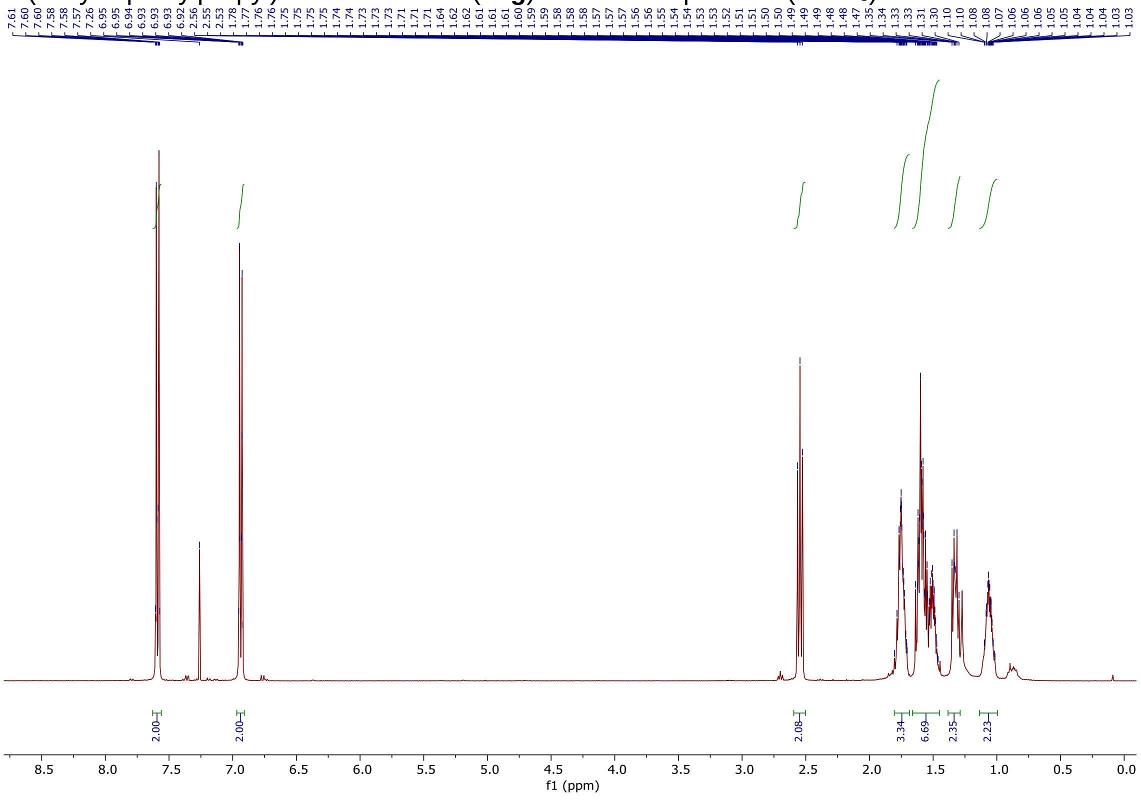

1-(3-Cyclopentylpropyl)-4-iodobenzene (**12g**) – $^{13}C\{^1H\}$ NMR spectrum ($CDCl_3$)

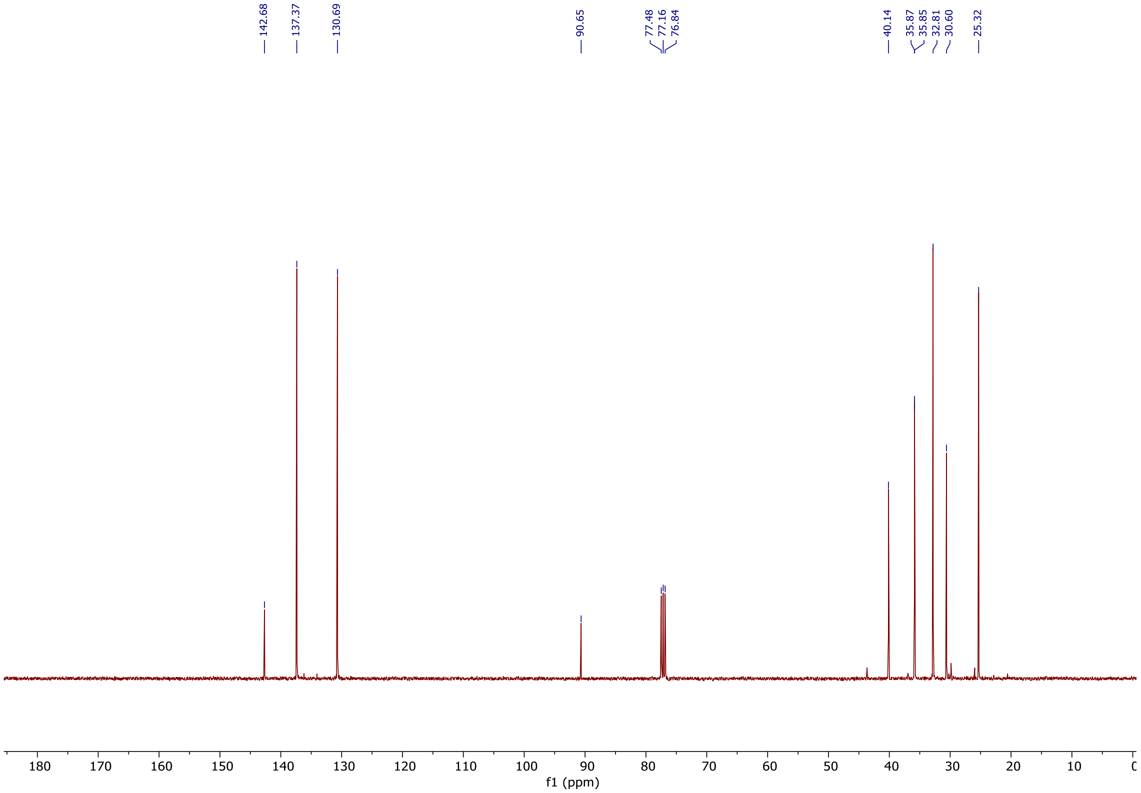

1-Bromo-4-(3-cyclopentylpropyl)benzene (**12h**) – ^1H NMR spectrum ($CDCl_3$)

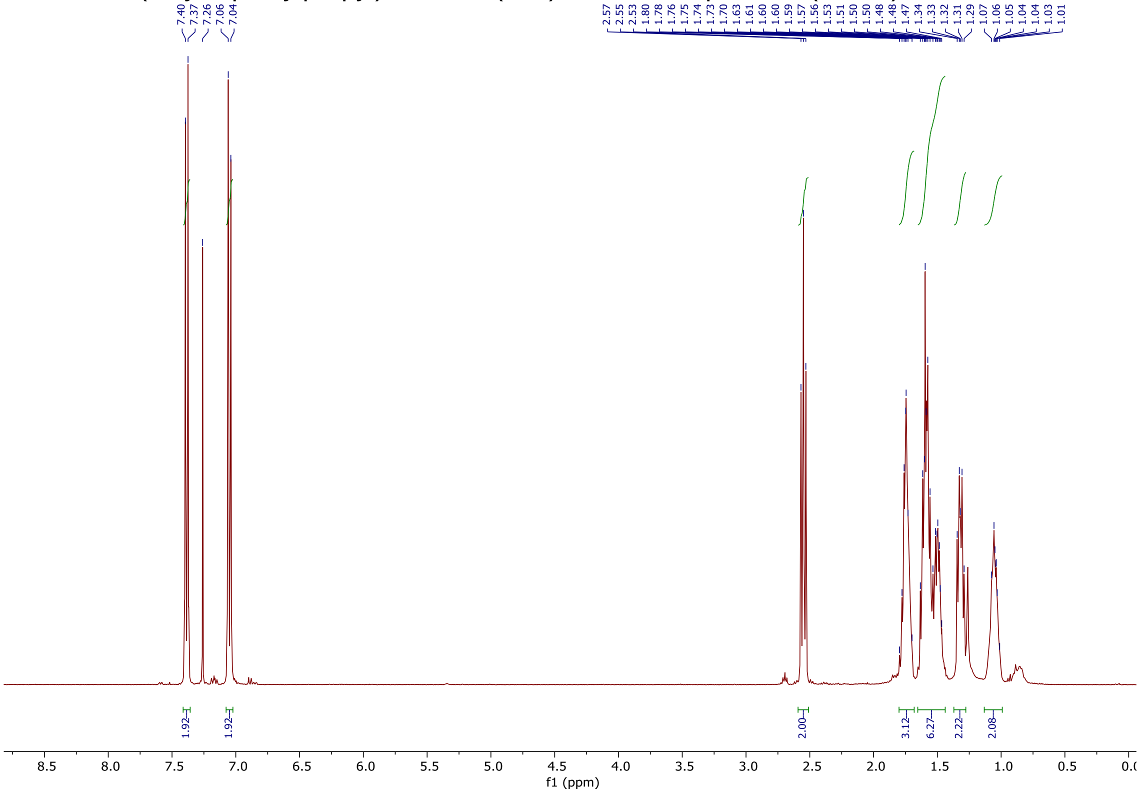

1-Bromo-4-(3-cyclopentylpropyl)benzene (**12h**) – $^{13}C\{^{1}H\}$ NMR spectrum ($CDCl_3$)

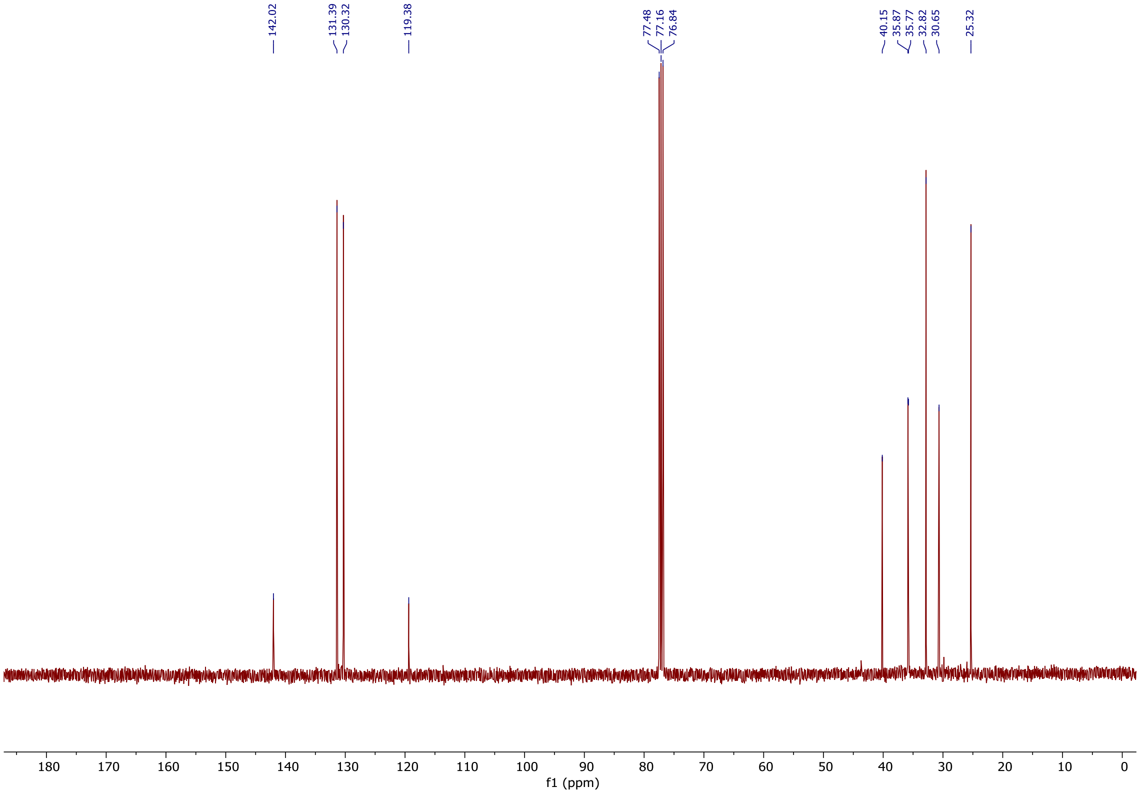

1-Chloro-4-(3-cyclopentylpropyl)benzene (**12i**) – ^{1}H NMR spectrum ($CDCl_3$)

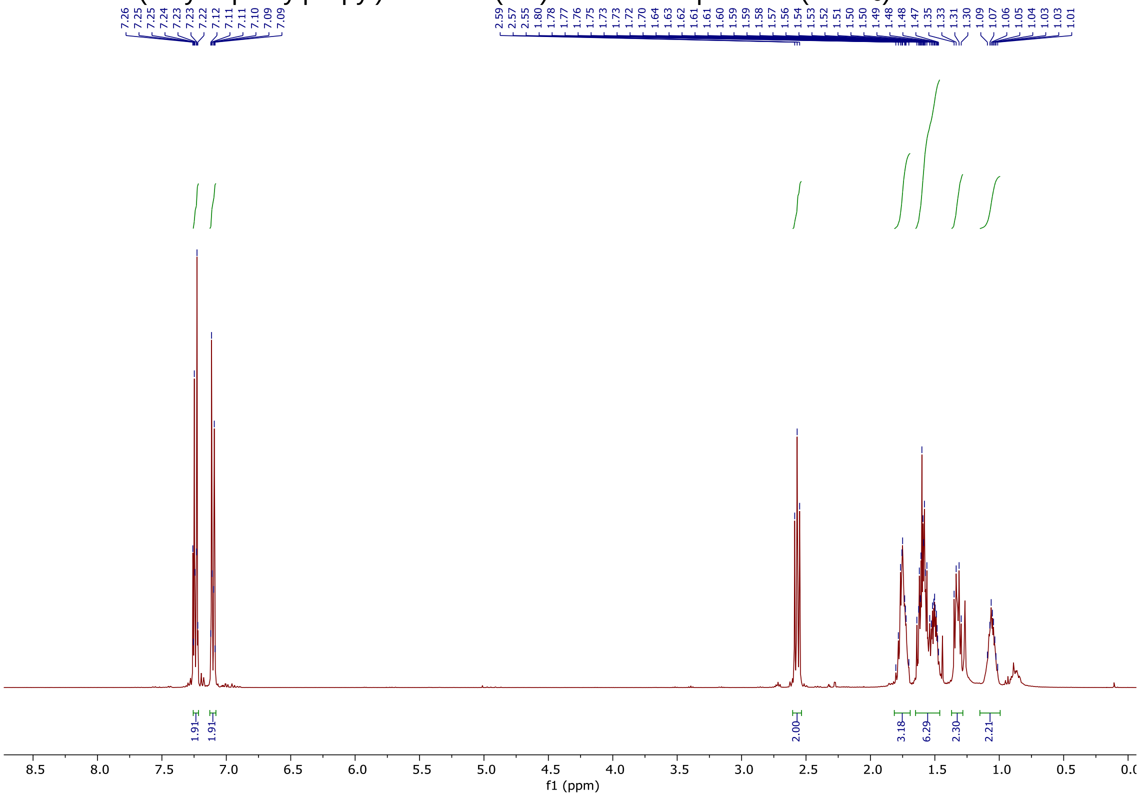

1-Chloro-4-(3-cyclopentylpropyl)benzene (**12i**) – $^{13}C\{^{1}H\}$ NMR spectrum ($CDCl_3$)

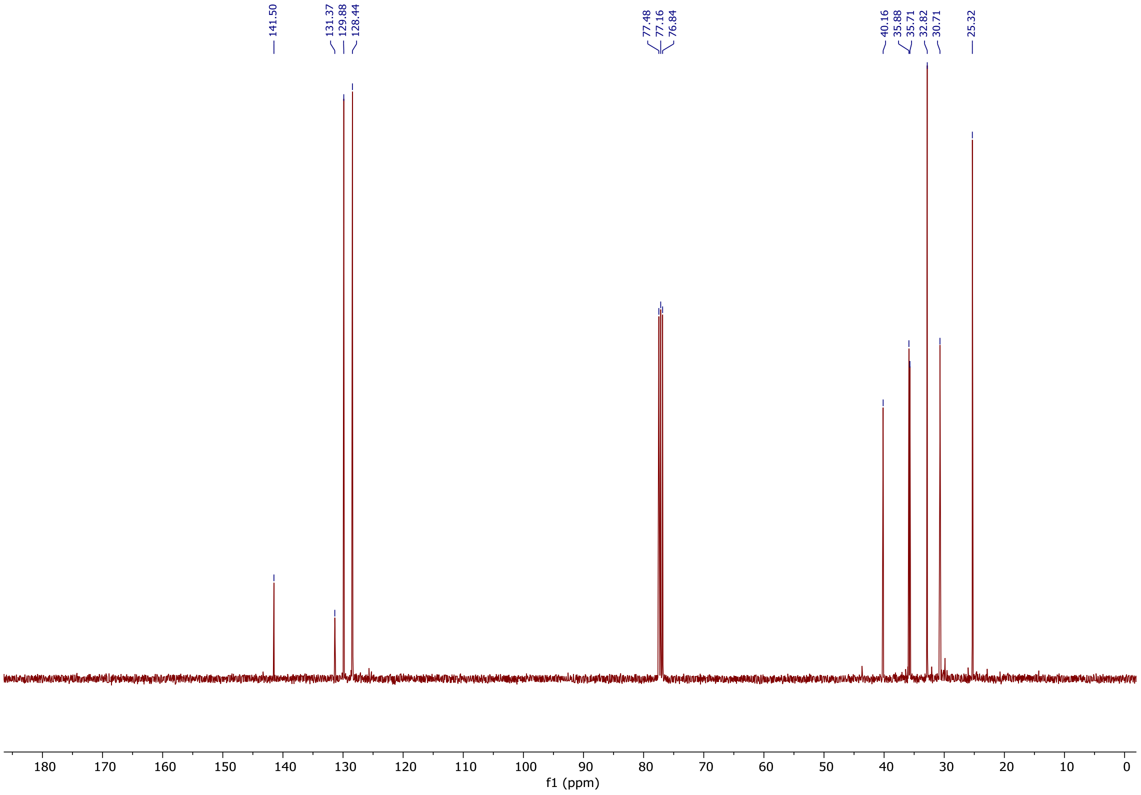

Boronic ester **12j** – ^{1}H NMR spectrum ($CDCl_3$)

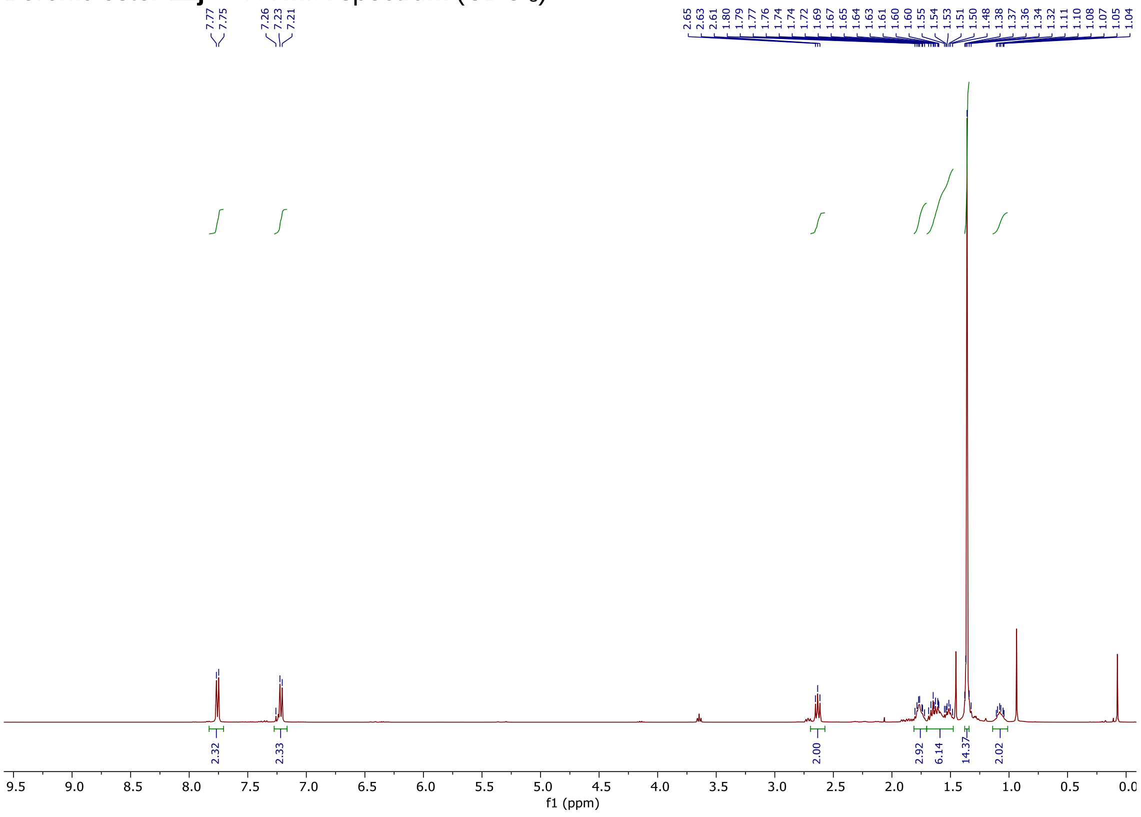

Boronic ester **12j** – ^{11}B NMR spectrum ($CDCl_3$)

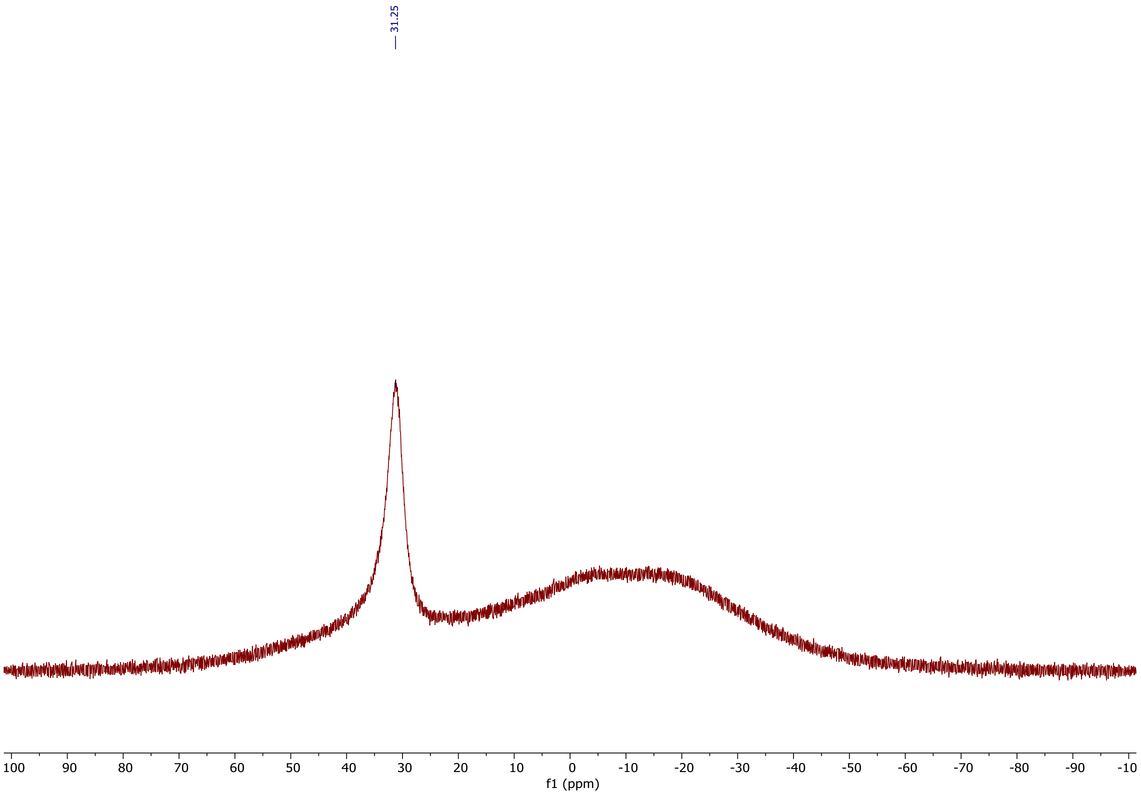

Boronic ester **12j** – ^{13}C{^{1}H} NMR spectrum ($CDCl_3$)

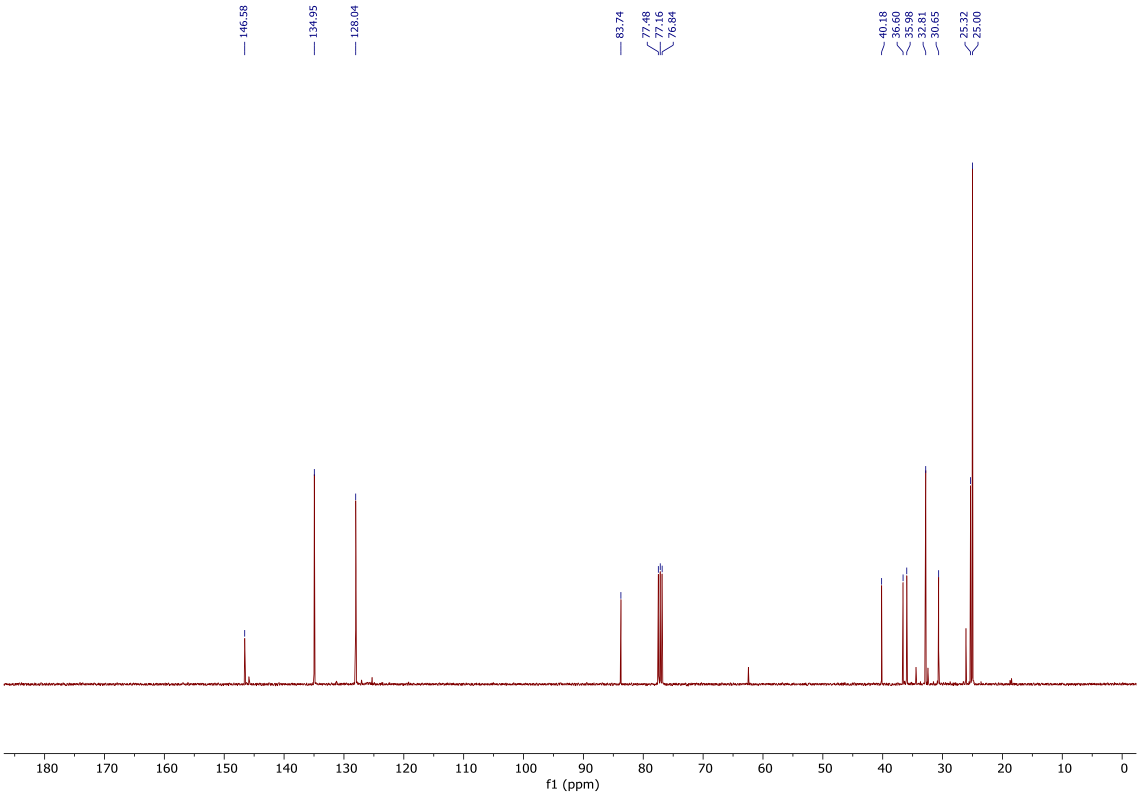

10-(4-Cyclopentylbutyl)-10H-phenoxazine (**12k**) – ^{1}H NMR spectrum ($CDCl_3$)

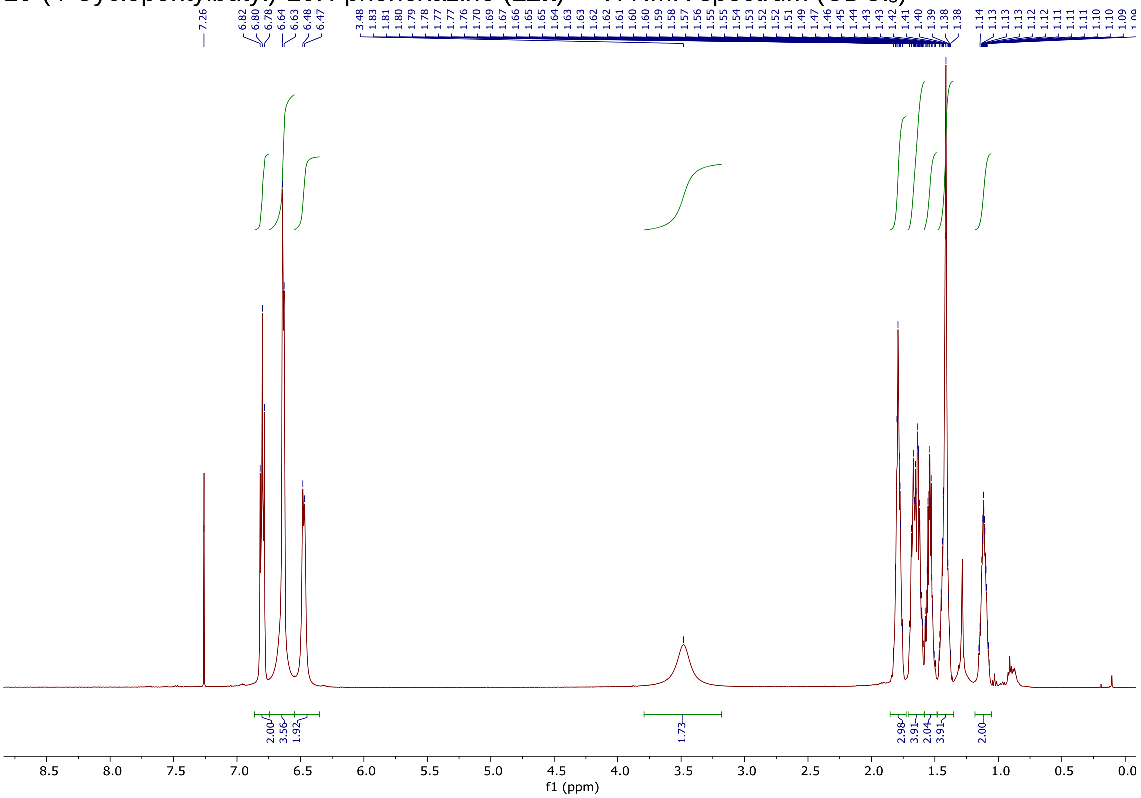

10-(4-Cyclopentylbutyl)-10H-phenoxazine (**12k**) – $^{13}C\{^{1}H\}$ NMR spectrum ($CDCl_3$)

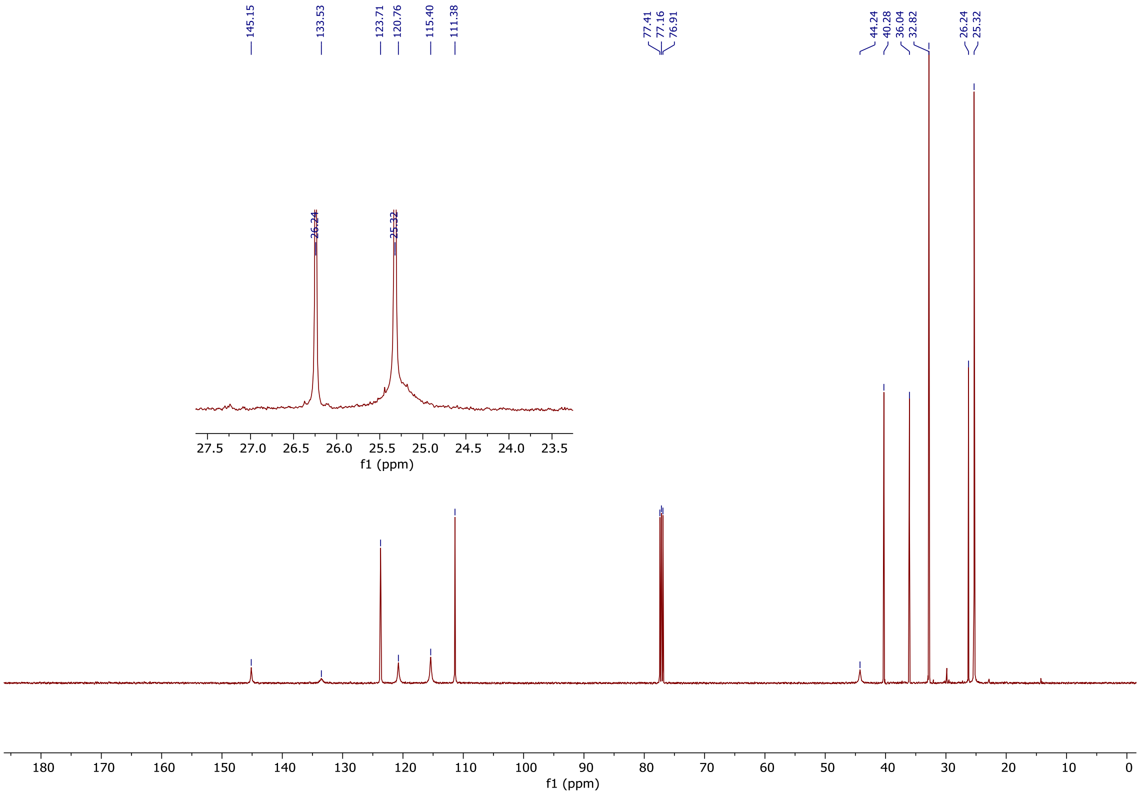

1-(4-Cyclopentylbutyl)- 2-phenyl-1H-indole (**12l**) – ^{1}H NMR spectrum ($CDCl_3$)

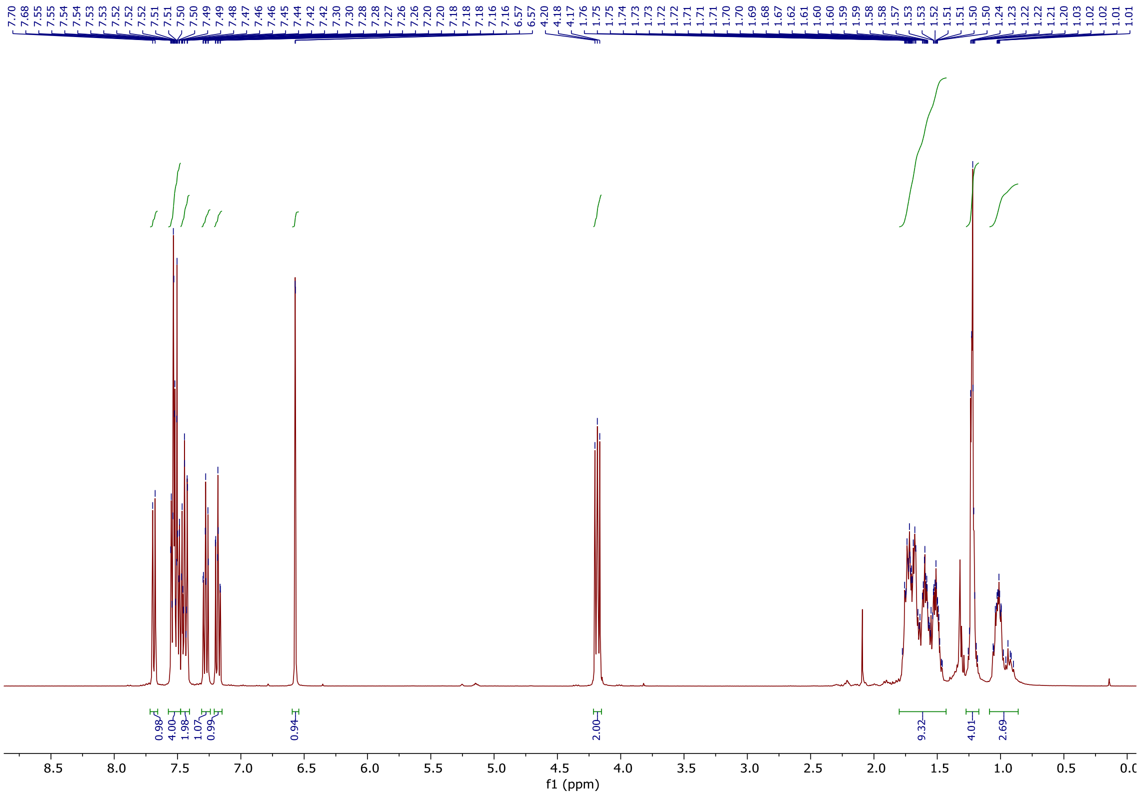

1-(4-Cyclopentylbutyl)- 2-phenyl-1H-indole (**12l**) – $^{13}C\{^{1}H\}$ NMR spectrum ($CDCl_3$)

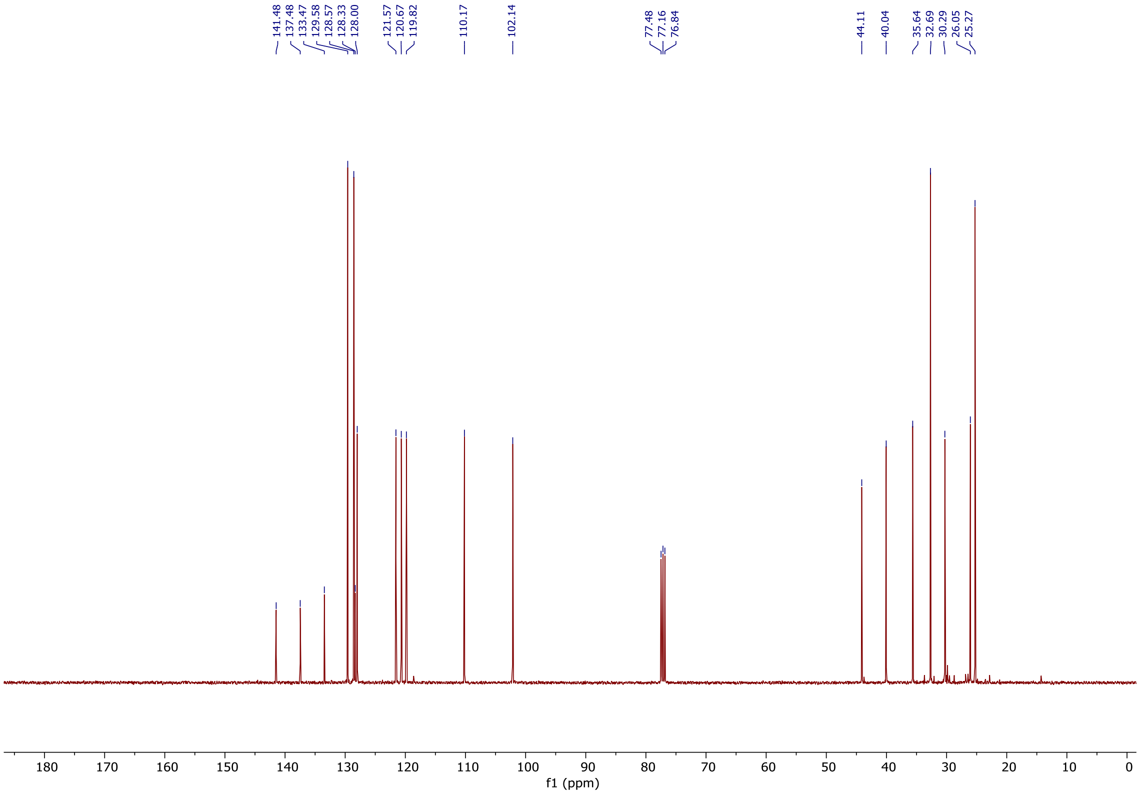

2-(4-(Cyclopentylmethyl)piperidin-1-yl)pyrimidine (**12m**) – ^{1}H NMR spectrum ($CDCl_3$)

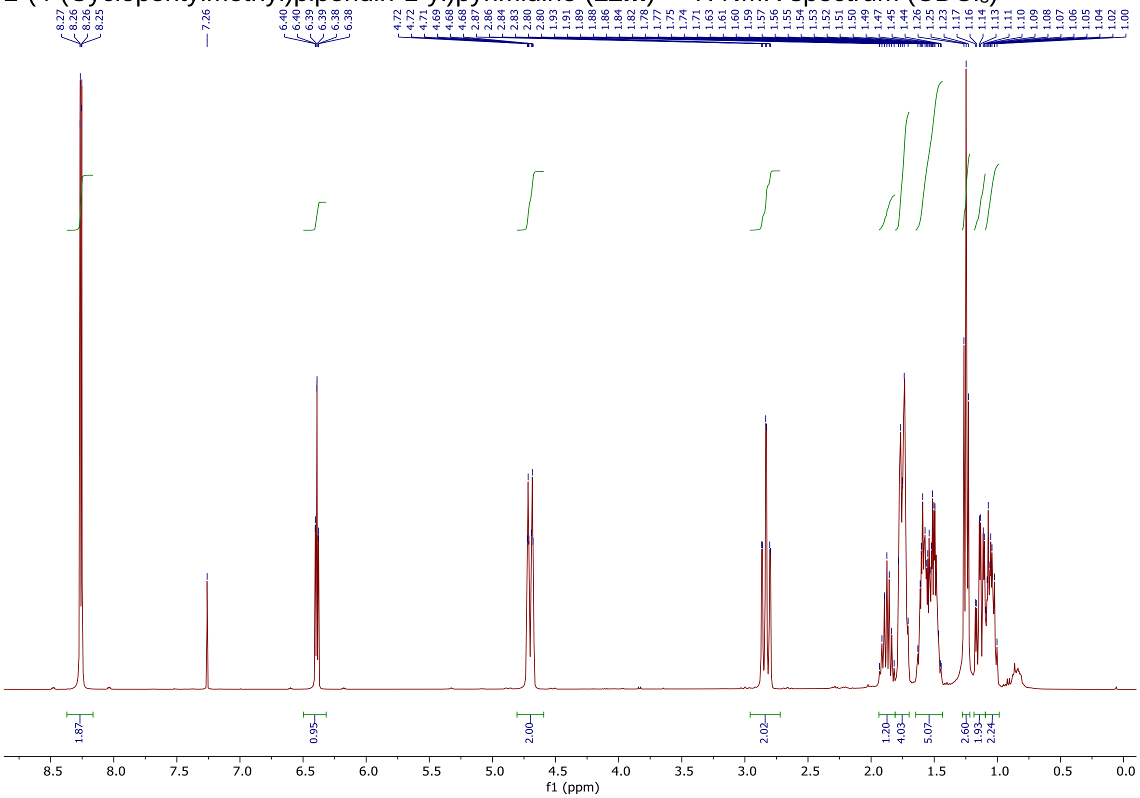

2-(4-(Cyclopentylmethyl)piperidin-1-yl)pyrimidine (**12m**) – ^{1}H qNMR spectrum ($CDCl_3$)

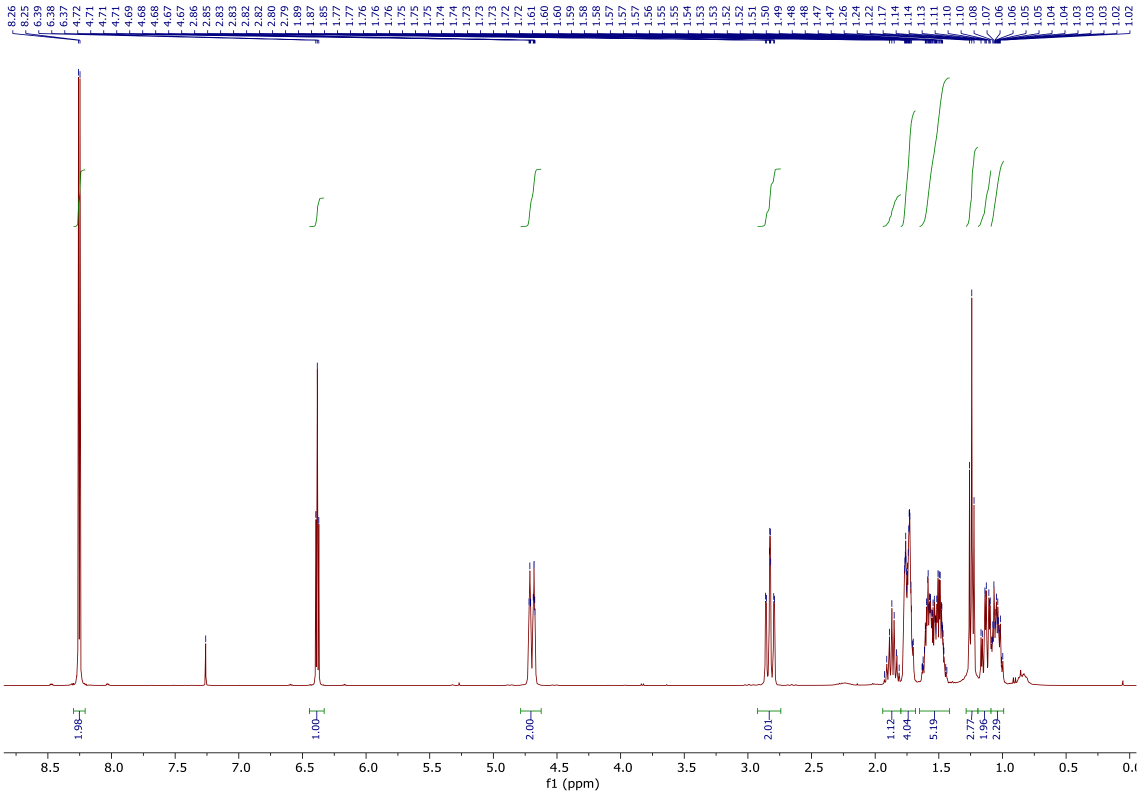

2-(4-(Cyclopentylmethyl)piperidin-1-yl)pyrimidine (**12m**) – $^{13}C\{^{1}H\}$ NMR spectrum ($CDCl_3$)

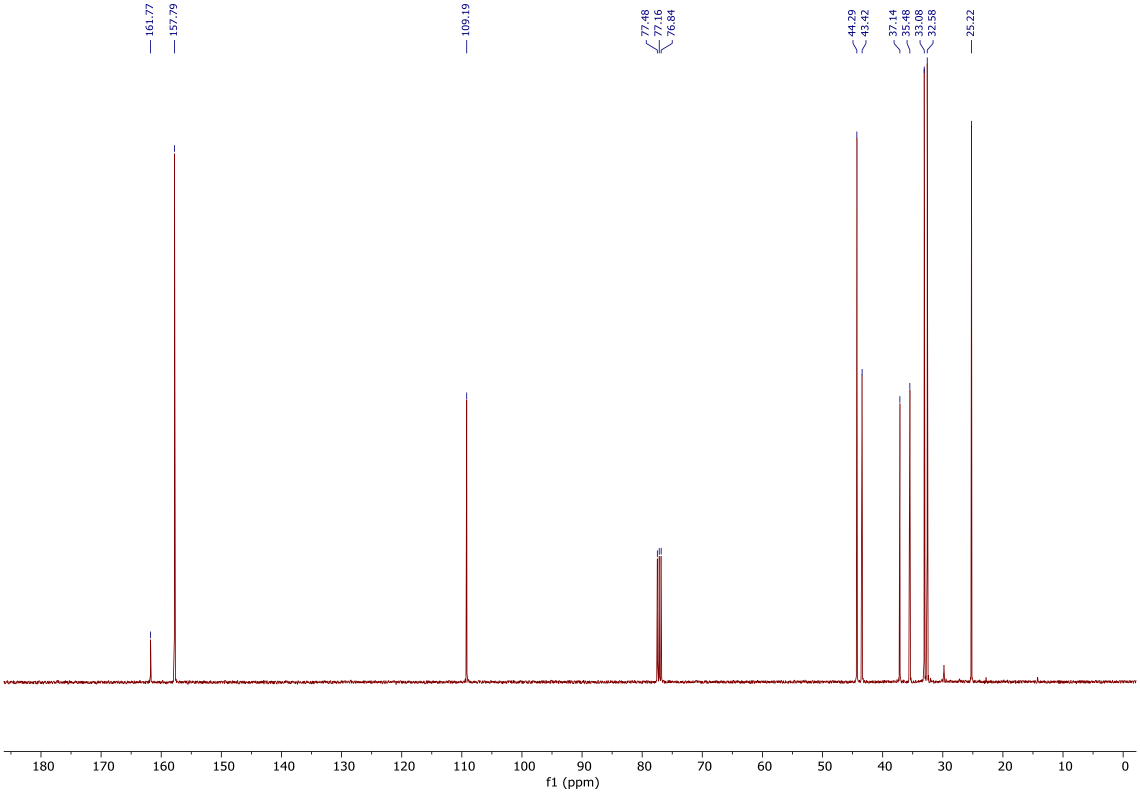

2-(4-(Cyclopentylmethyl)piperidin-1-yl)pyrimidine (**12m**) – HSQC NMR spectrum ($CDCl_3$)

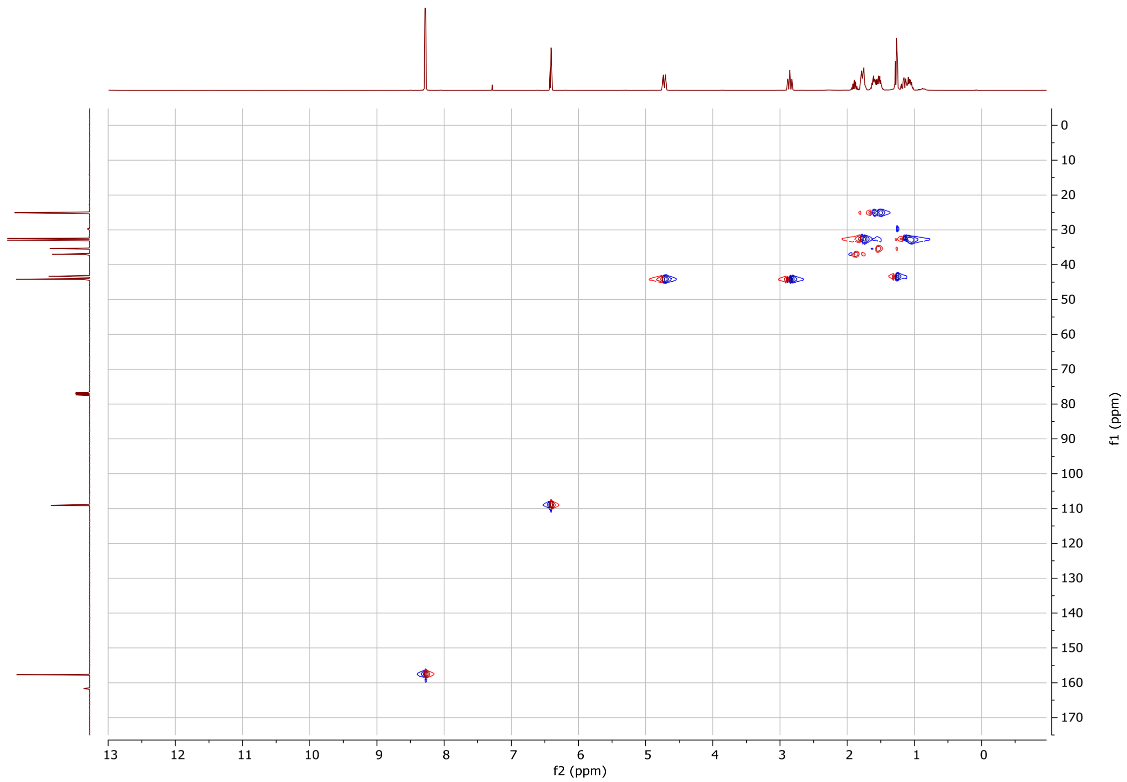

2-(4-(Cyclopentylmethyl)piperidin-1-yl)pyrimidine (**12m**) – HMBC NMR spectrum ($CDCl_3$)

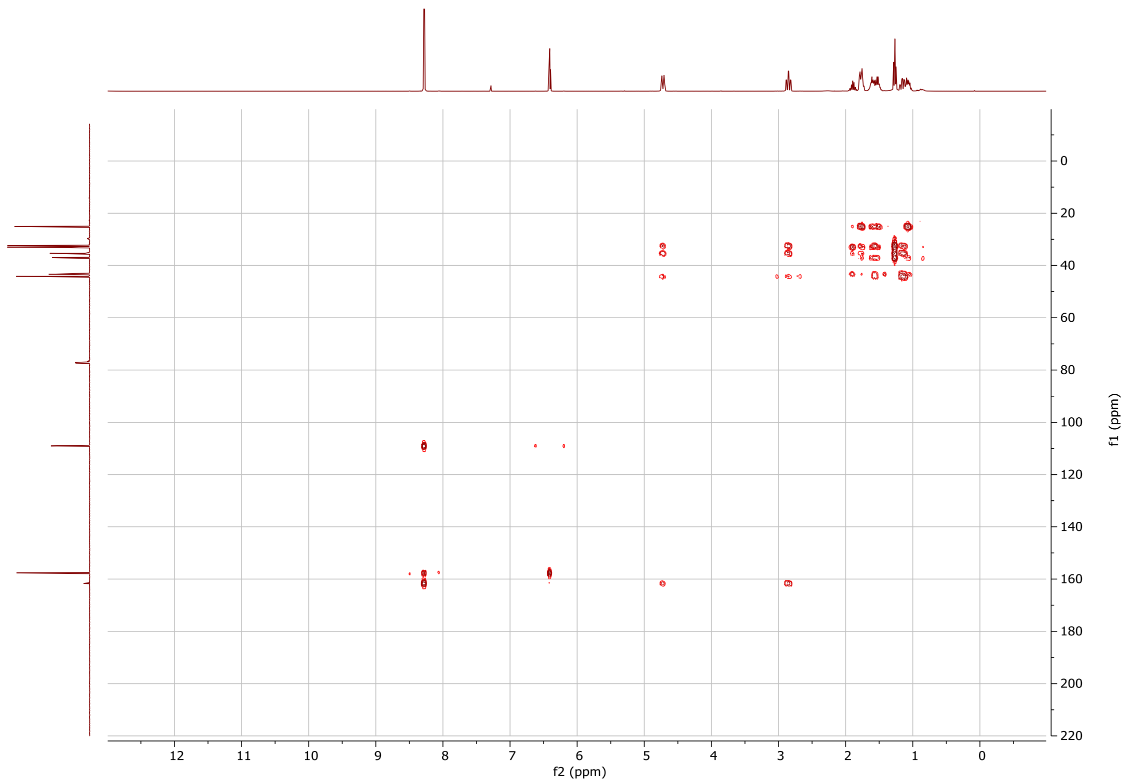

2-(4-(Cyclopentylmethyl)piperidin-1-yl)pyrimidine (**12m**) – COSY NMR spectrum ($CDCl_3$)

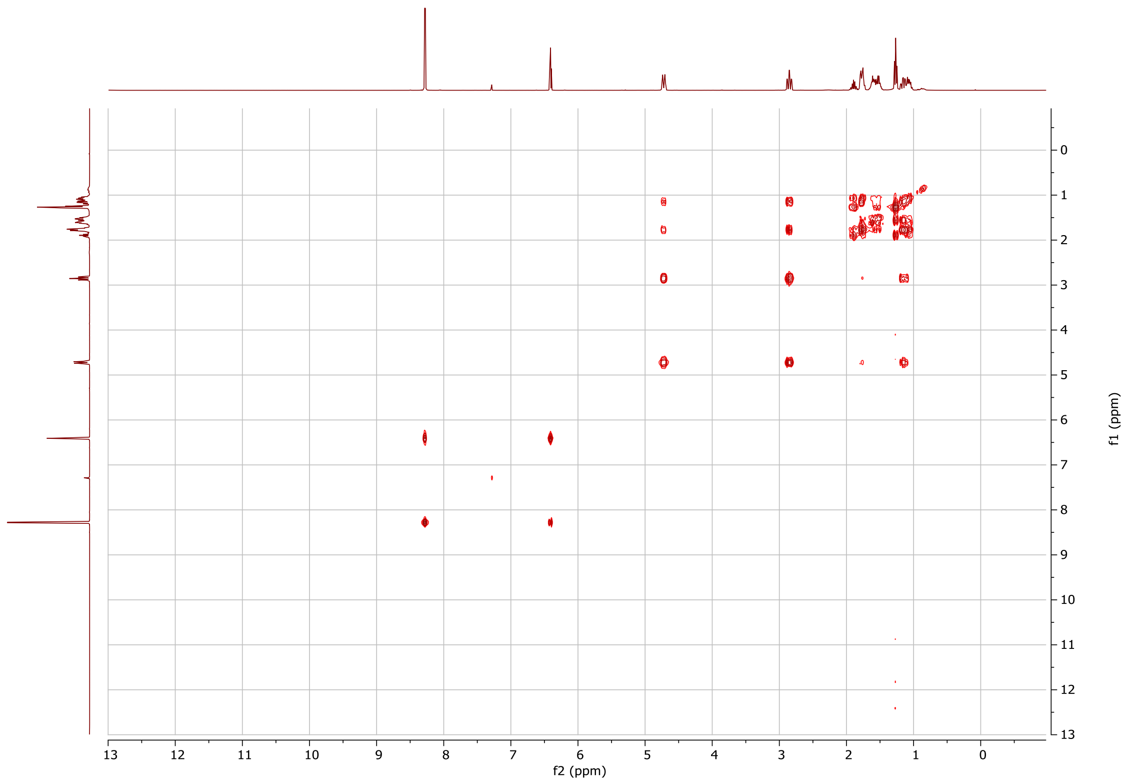

2-(4-((Cyclopentyl-1-*d*)methyl)piperidin-1-yl)pyrimidine (**12m-*d***) – ^{1}H qNMR spectrum ($CDCl_3$)

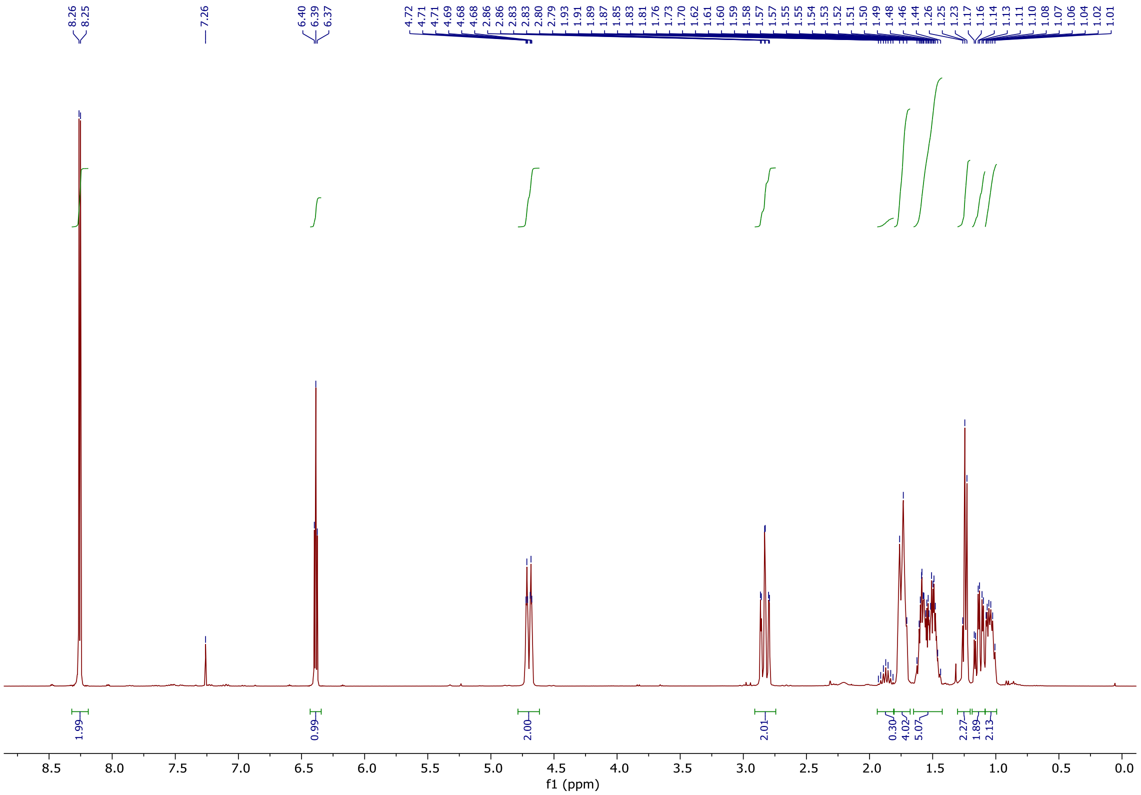

2-(4-((Cyclopentyl-1-*d*)methyl)piperidin-1-yl)pyrimidine (**12m-*d***) – ^{13}C{^{1}H} NMR spectrum ($CDCl_3$)

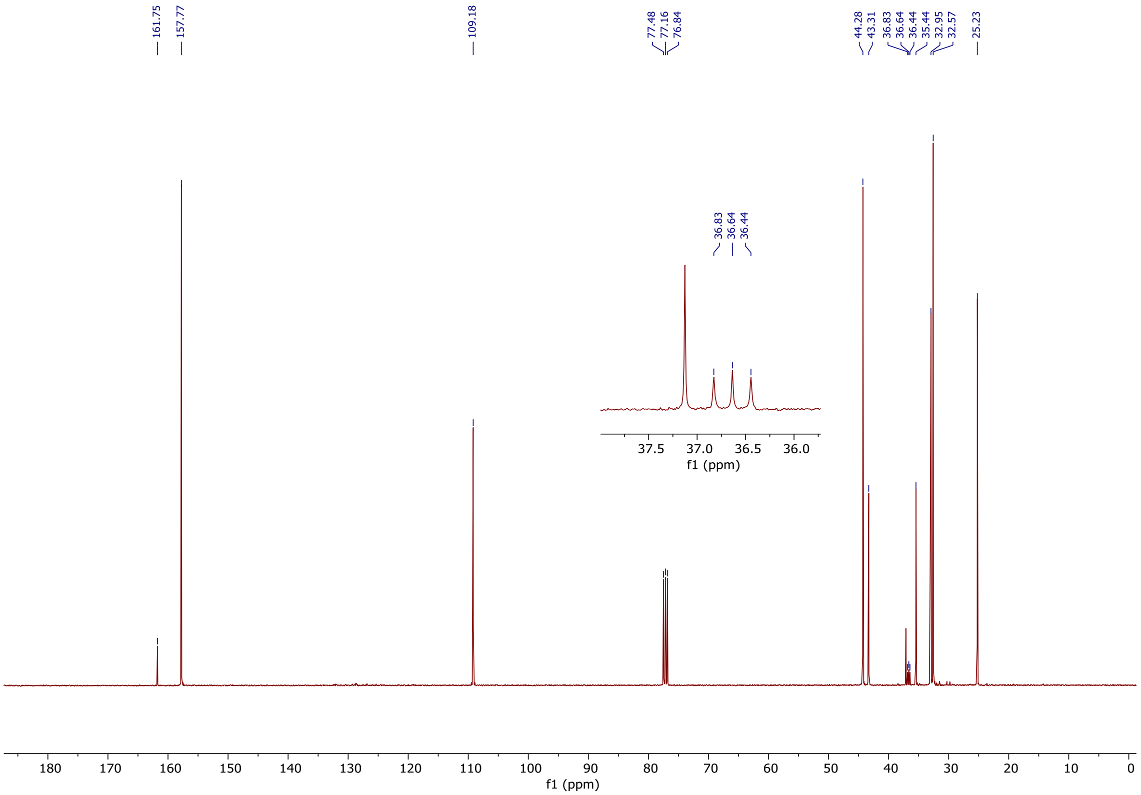

Overlay of the alkyl region of the $^{13}C\{^{1}H\}$ NMR spectra ($CDCl_3$) of **12m** (red) and **12m-*d*** (green)

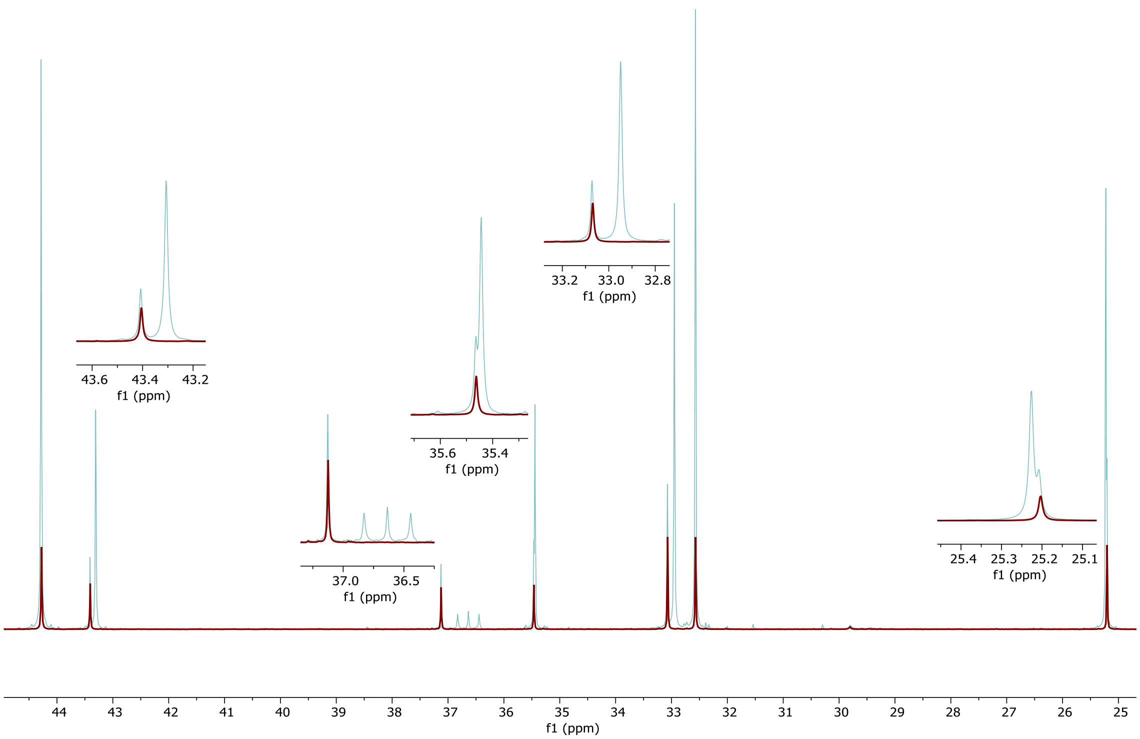

1-(4-Iodophenyl)spiro[2.4]heptane (**12n**) – ^{1}H NMR spectrum ($CDCl_3$)

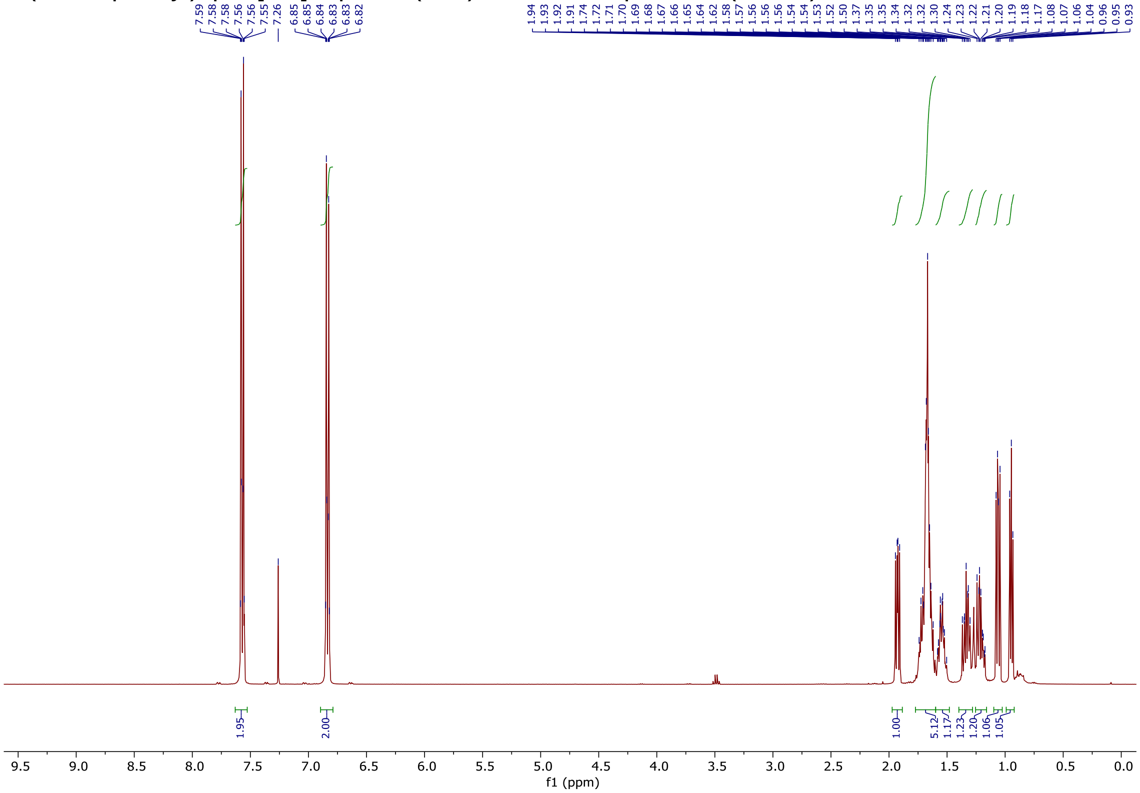

1-(4-Iodophenyl)spiro[2.4]heptane (**12n**) – $^{13}C\{^{1}H\}$ NMR spectrum ($CDCl_3$)

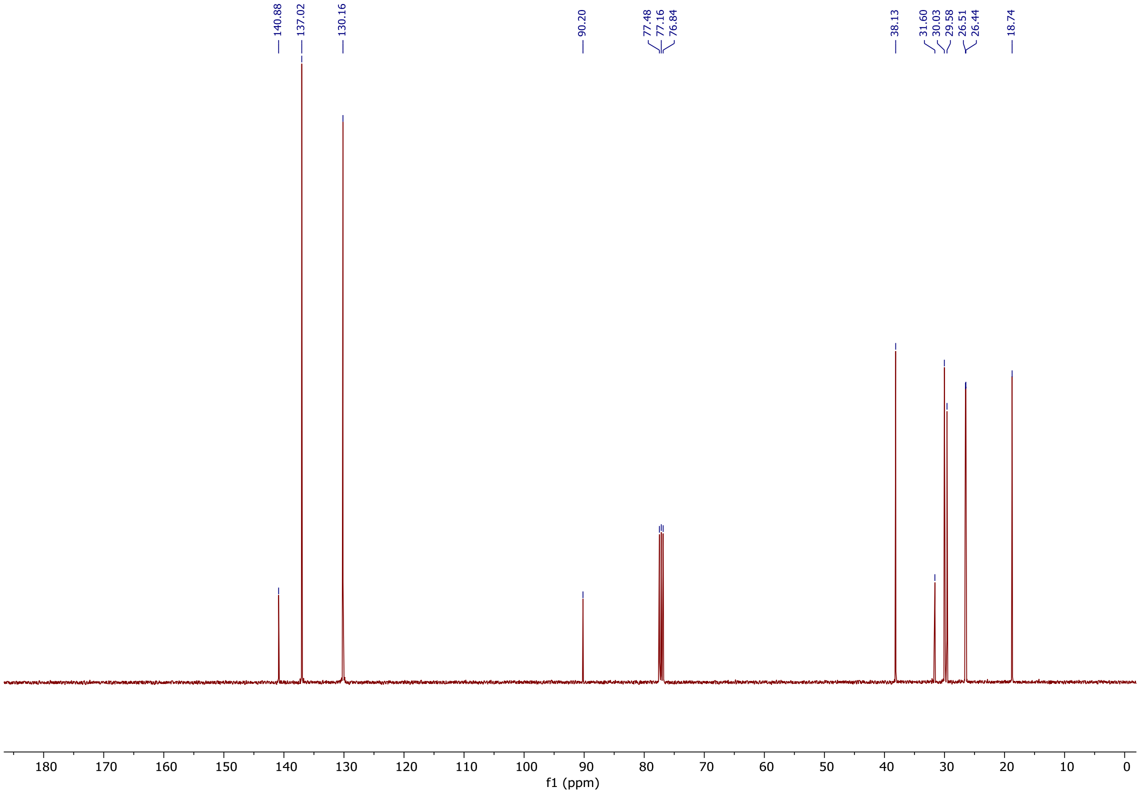

1-(Cyclopentylmethyl)-4-(hexyloxy)benzene (**12o**) – ^{1}H NMR spectrum ($CDCl_3$)

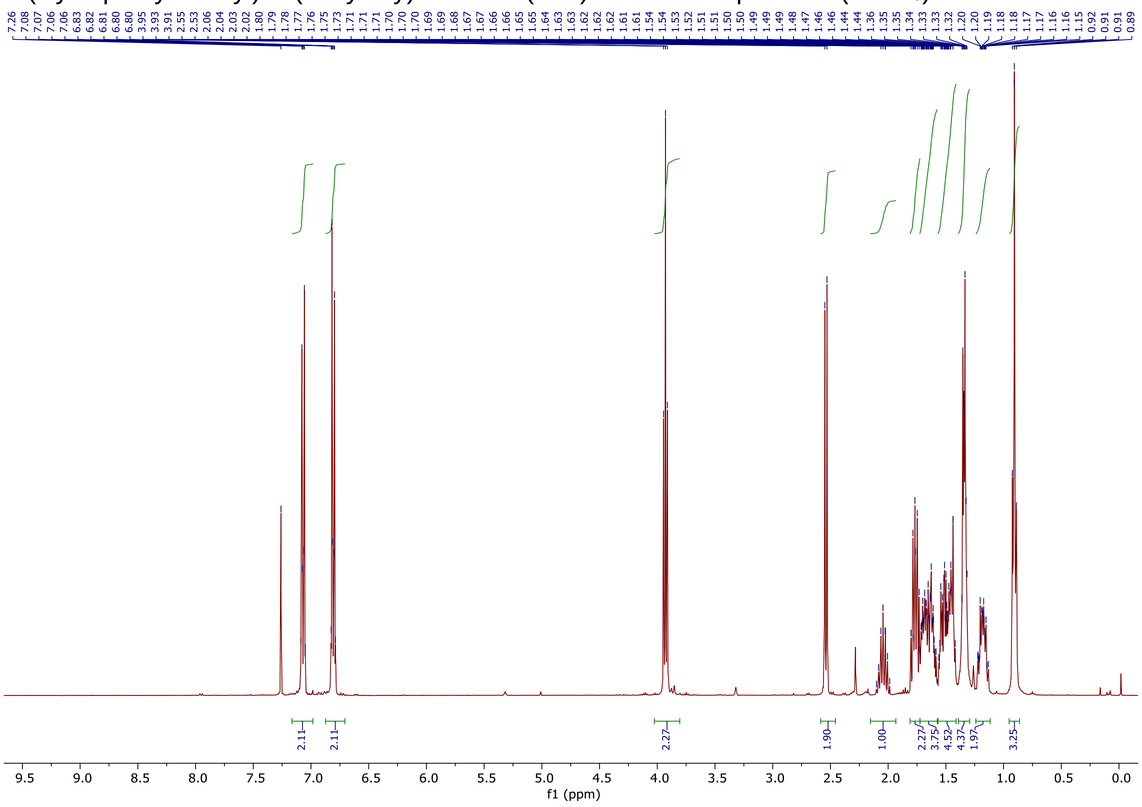

1-(Cyclopentylmethyl)-4-(hexyloxy)benzene (**12o**) – $^{13}C\{^{1}H\}$ NMR spectrum ($CDCl_3$)

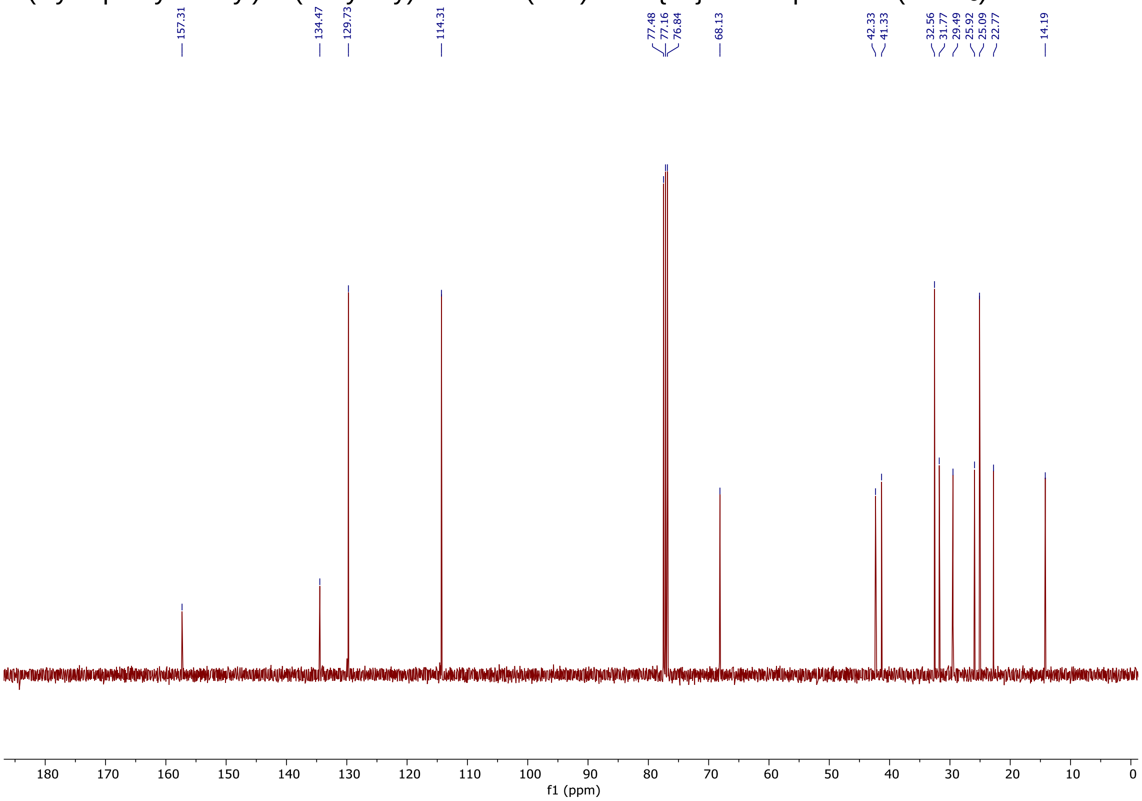

4-(3-Cyclopentylpropyl)phenol (**12p**) – ^{1}H NMR spectrum ($CDCl_3$)

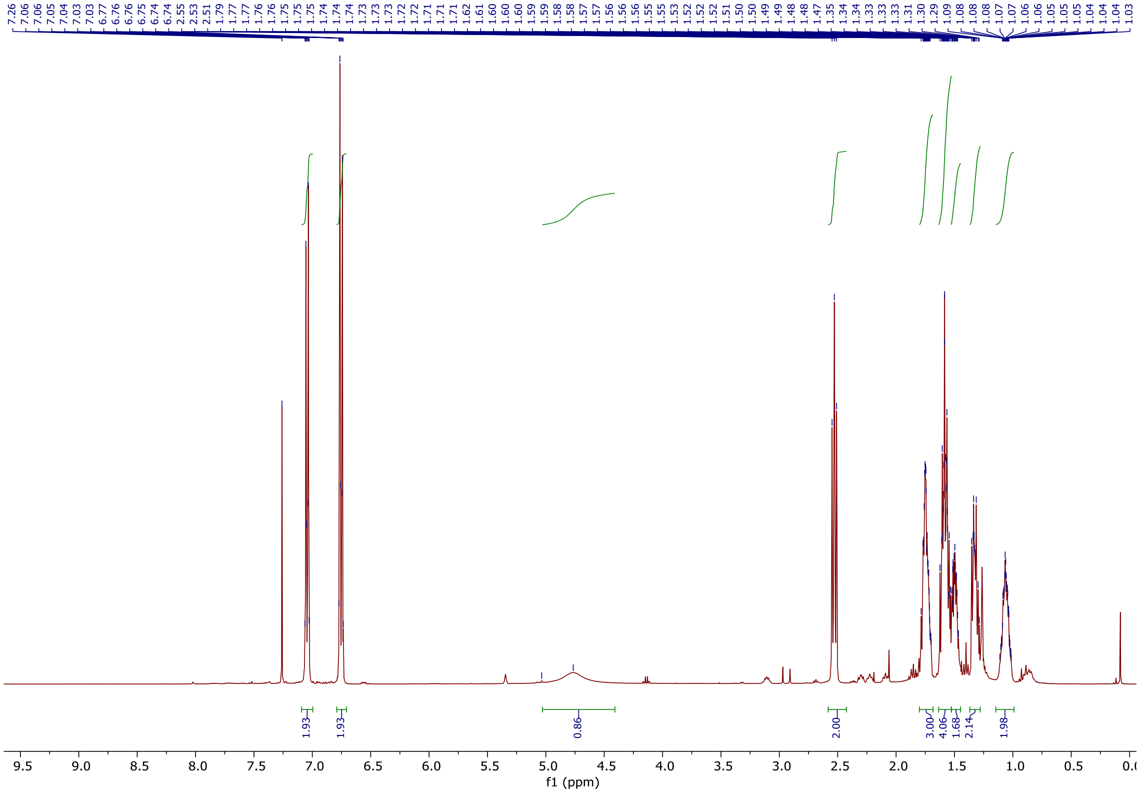

4-(3-Cyclopentylpropyl)phenol (**12p**) – $^{13}C\{^1H\}$ NMR spectrum ($CDCl_3$)

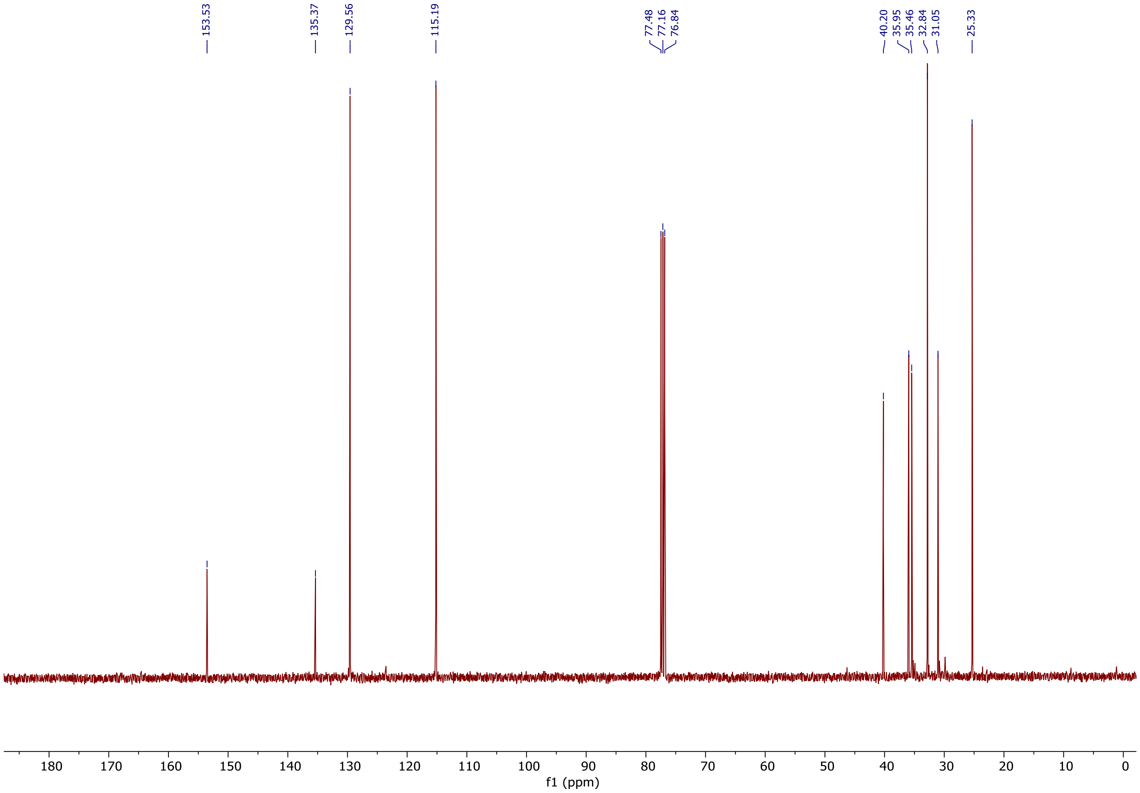

4-(4-Cyclopentylbutyl)benzoic acid (**12q**) – ^1H NMR spectrum ($CDCl_3$)

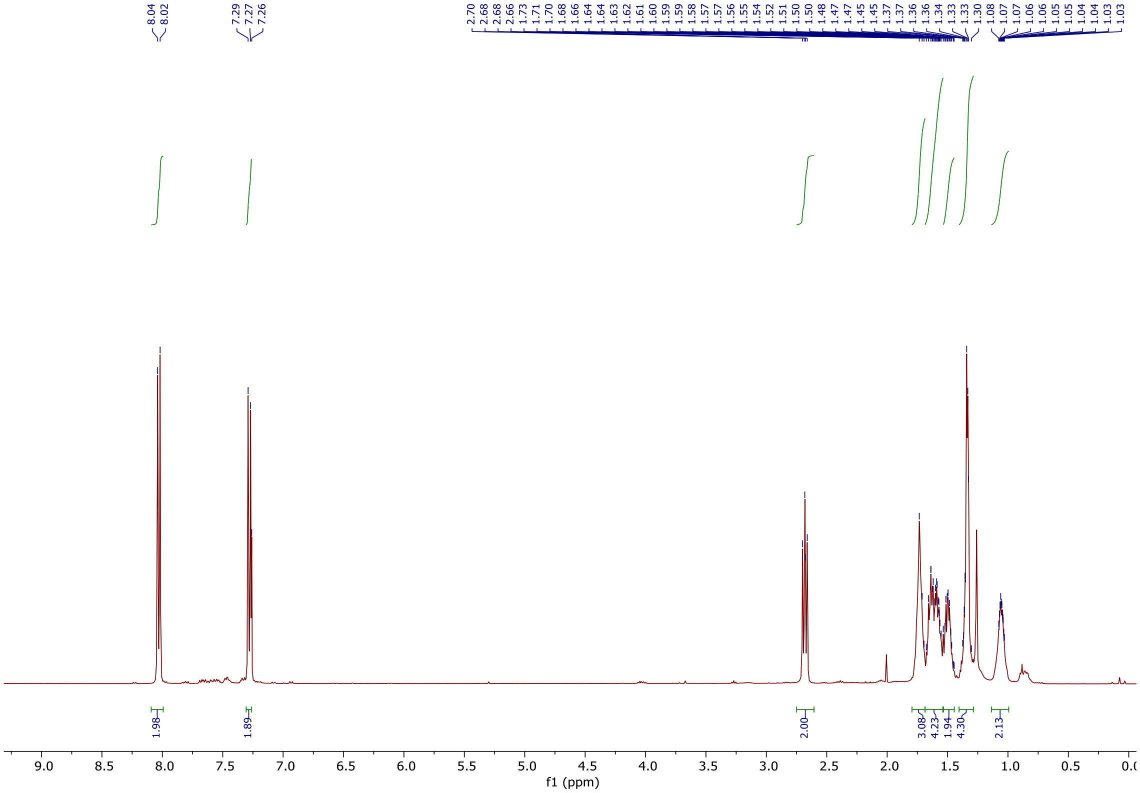

4-(4-Cyclopentylbutyl)benzoic acid (**12q**) – $^{13}C\{^1H\}$ NMR spectrum ($CDCl_3$)

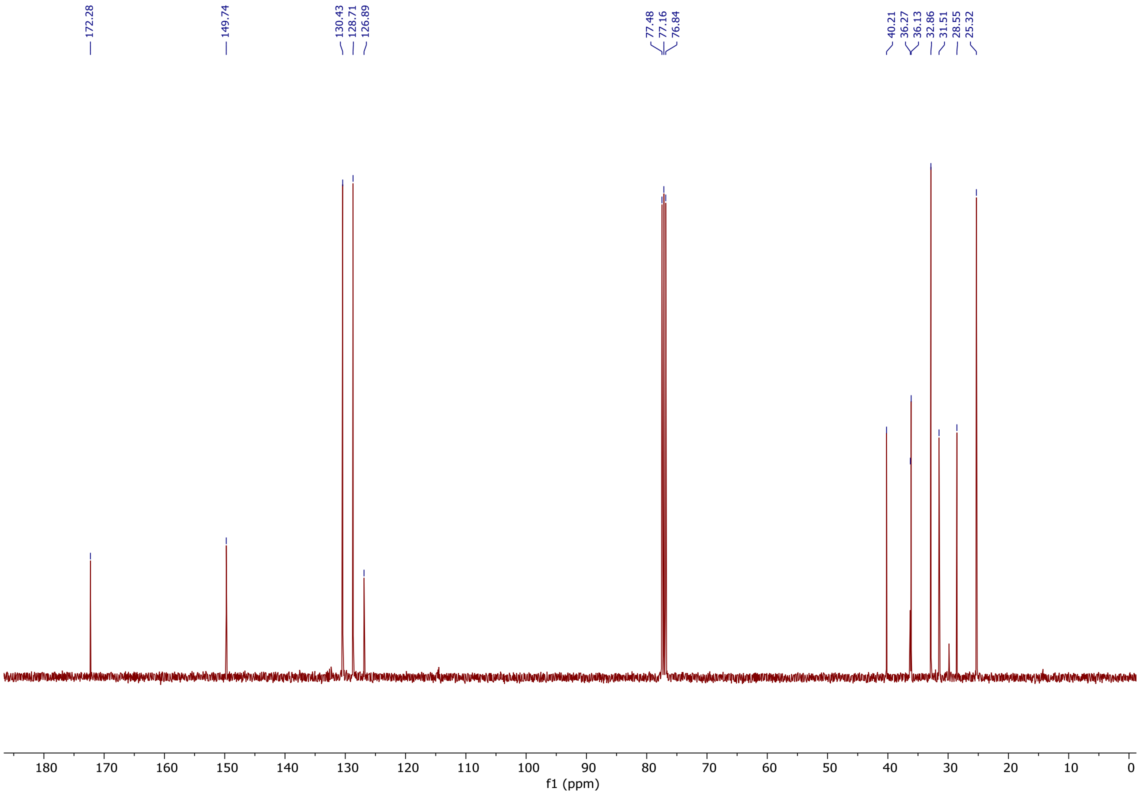

5-(4-Cyclopentylbutoxy)-1*H*-pyrrolo[2,3-b]pyridine (**12r**) – ^1H NMR spectrum ($CDCl_3$)

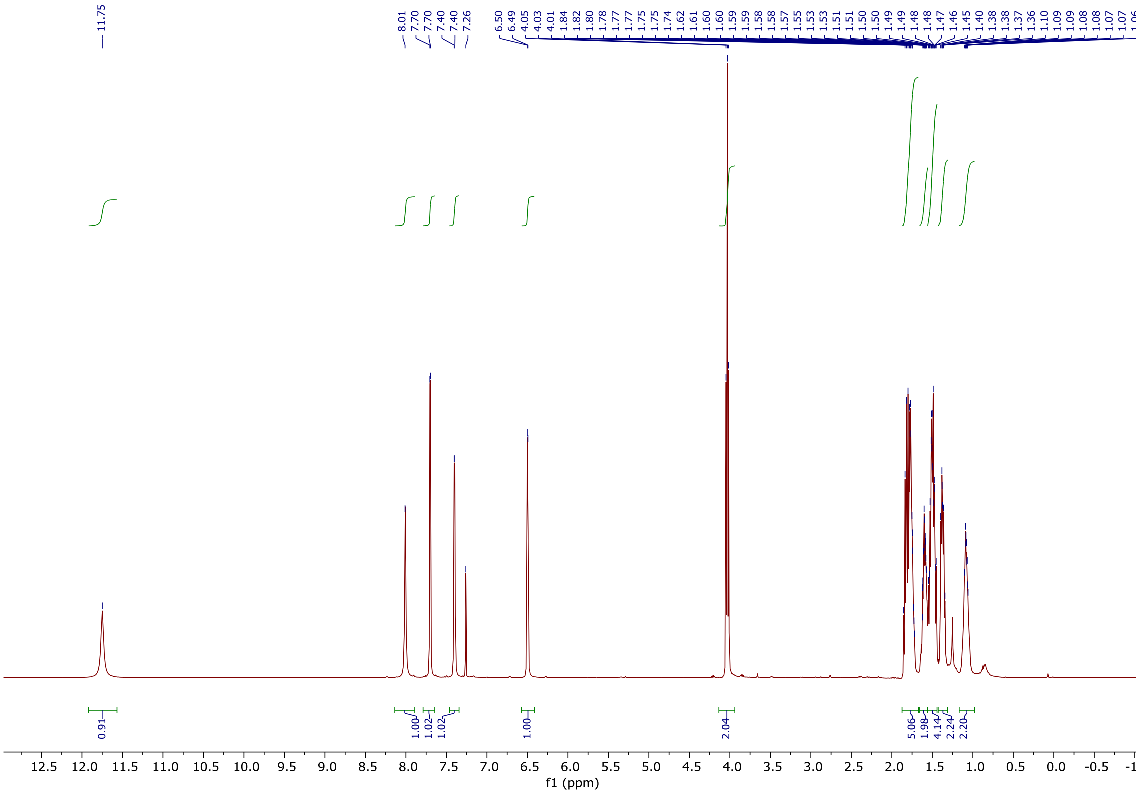

5-(4-Cyclopentylbutoxy)-1*H*-pyrrolo[2,3-b]pyridine (**12r**) – $^{13}C\{^{1}H\}$ NMR spectrum ($CDCl_3$)

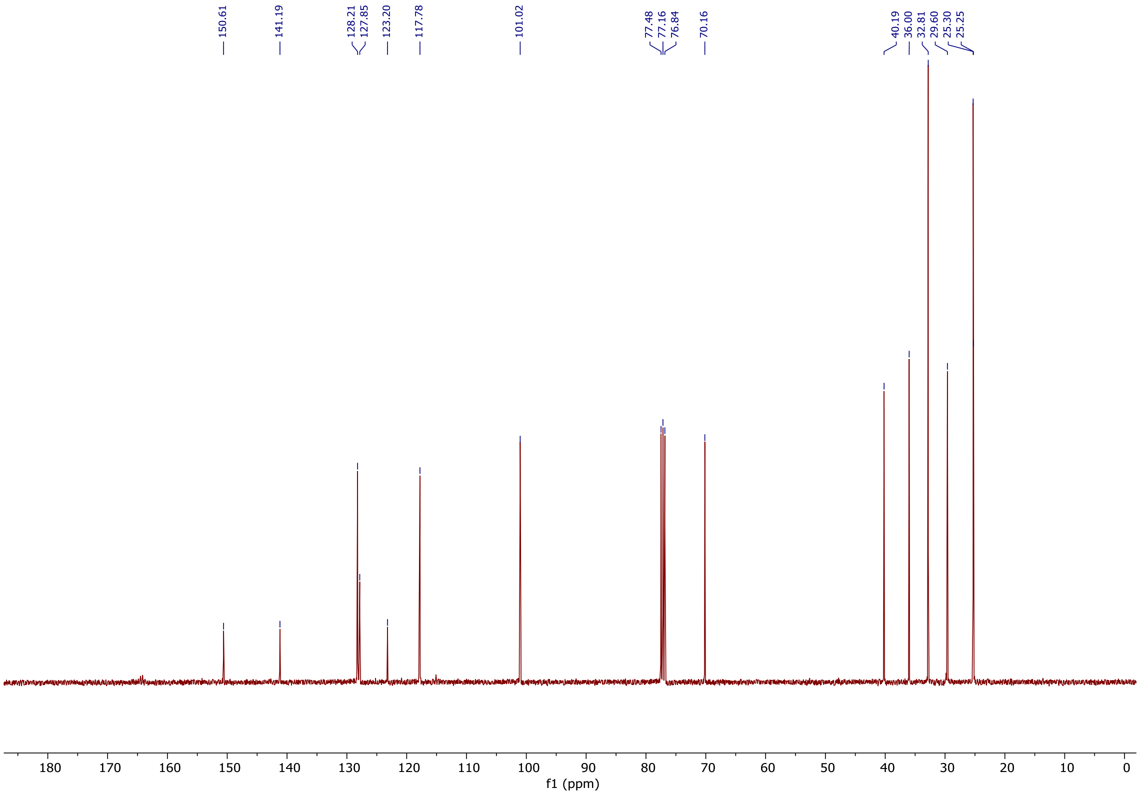

Alcohol **12s** – ^{1}H NMR spectrum ($CDCl_3$)

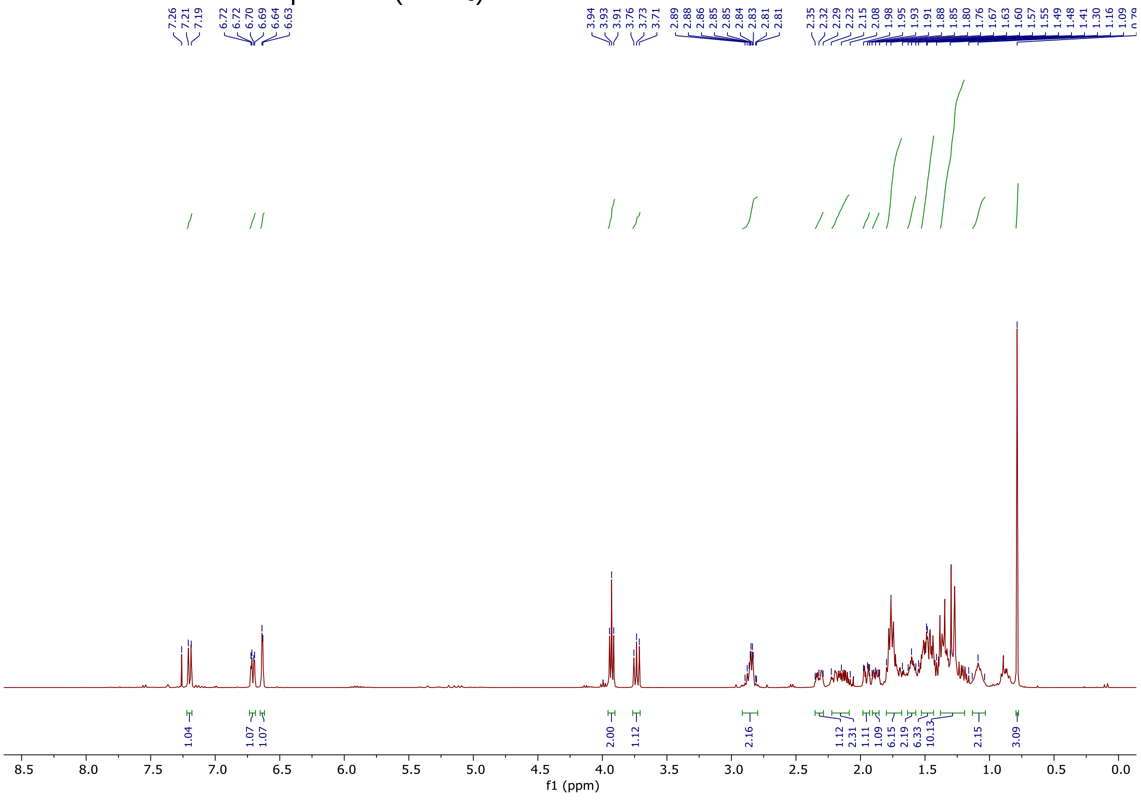

Alcohol **12s** – $^{13}C\{^1H\}$ NMR spectrum ($CDCl_3$)

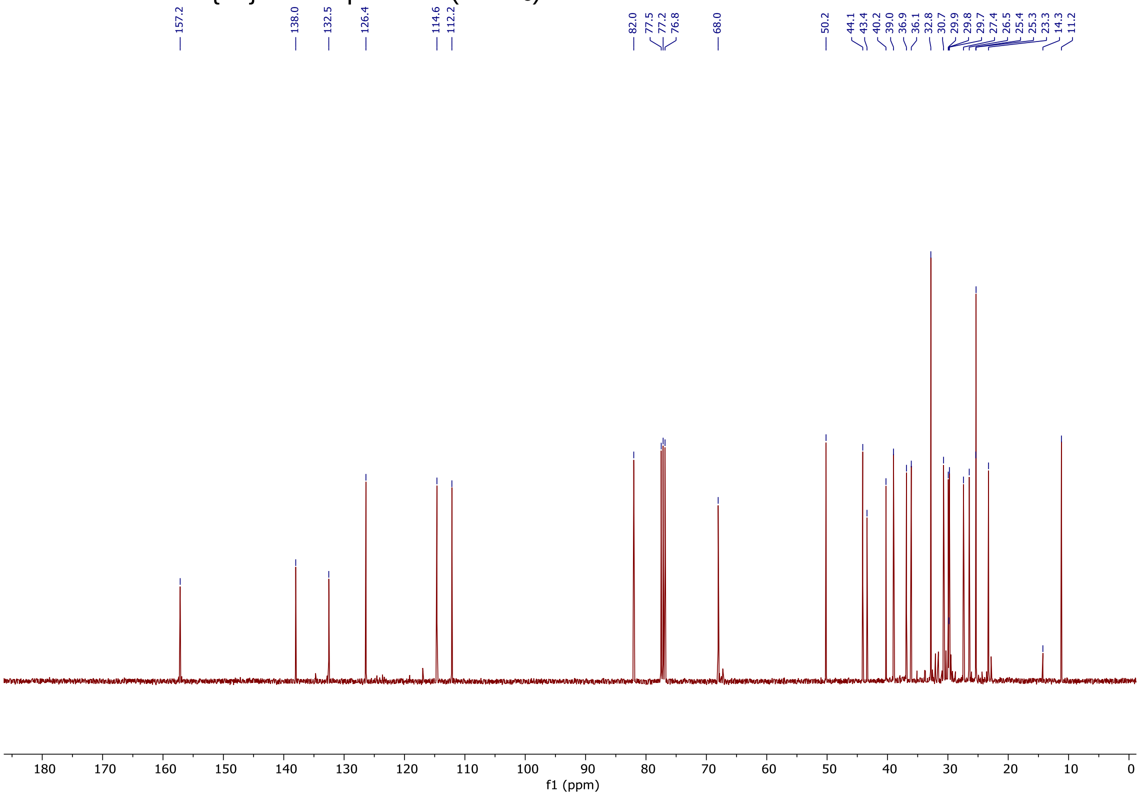

Oxazolidinone **12t** – ^1H NMR spectrum ($CDCl_3$)

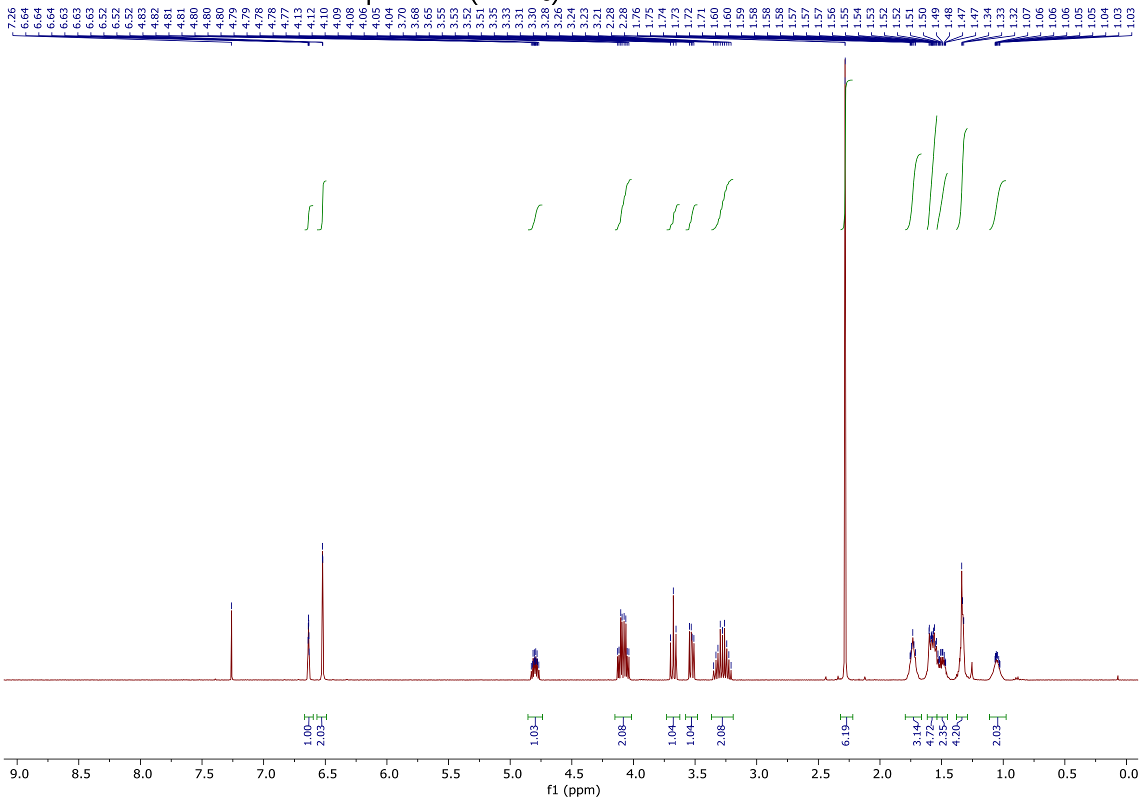

Oxazolidinone **12t** – $^{13}C\{^{1}H\}$ NMR spectrum ($CDCl_3$)

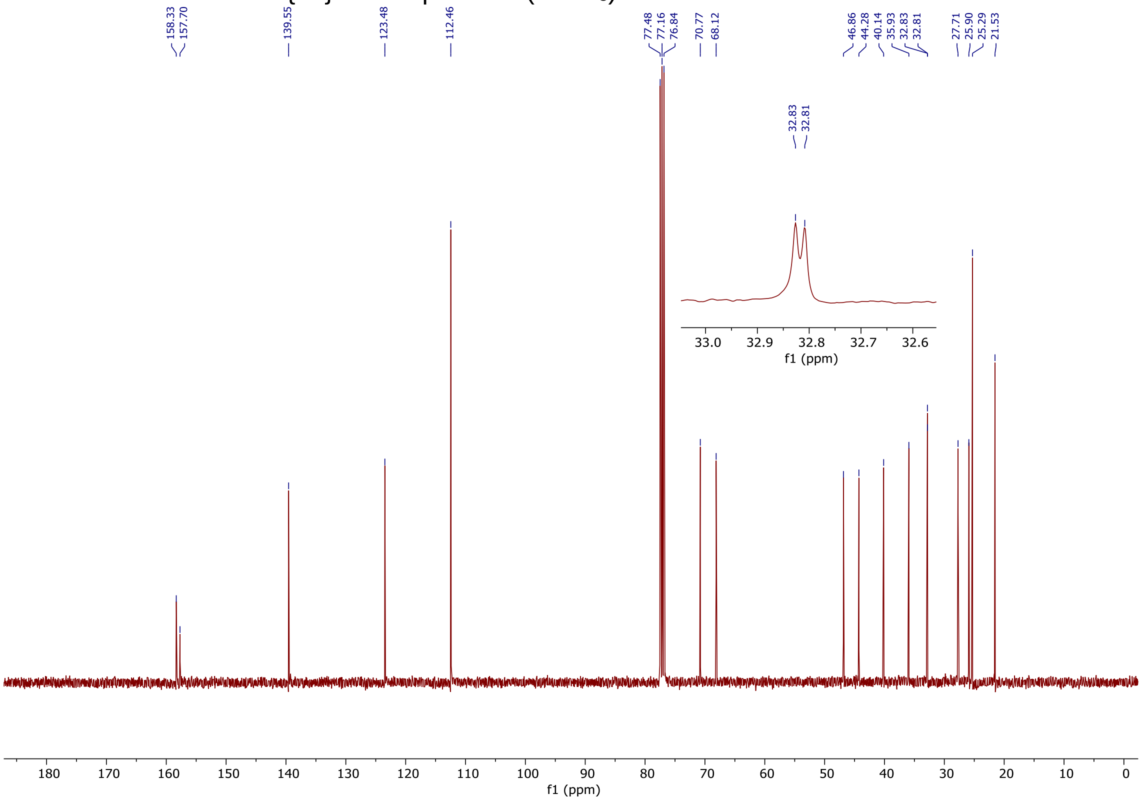

Pyridine **12u** – ^{1}H NMR spectrum ($CDCl_3$)

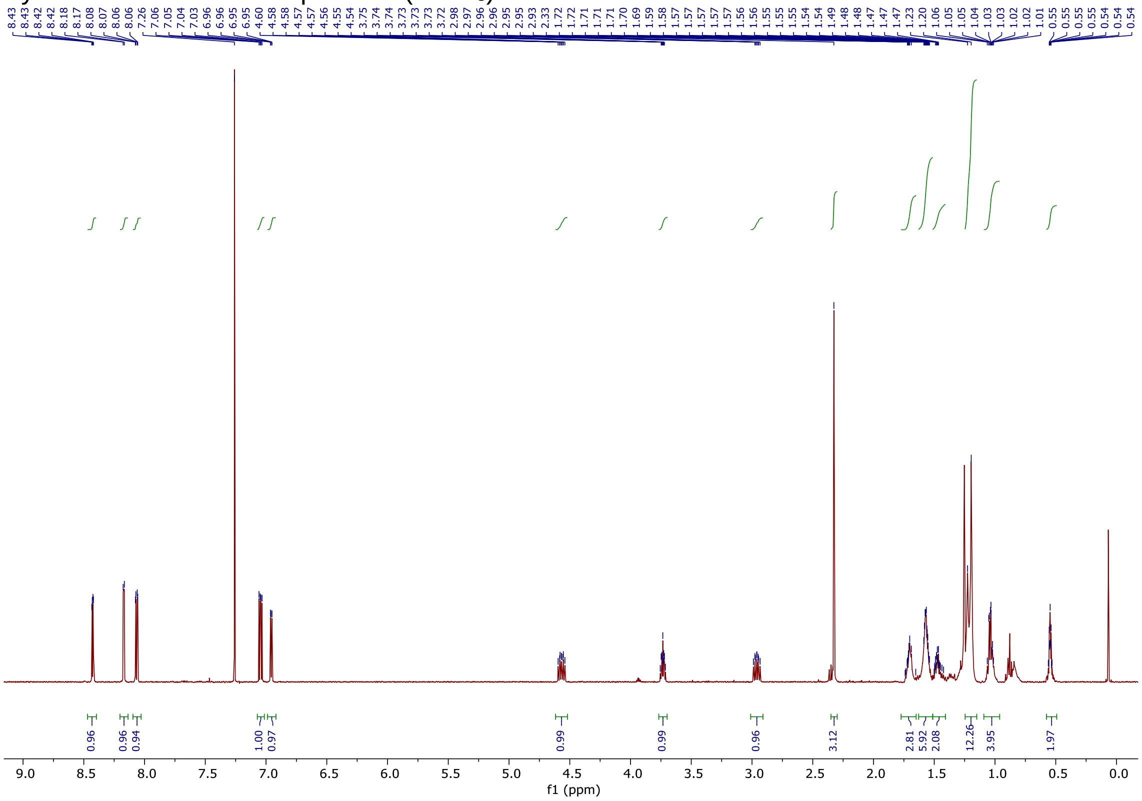

Pyridine **12u** – $^{13}C\{^{1}H\}$ NMR spectrum ($CDCl_3$)

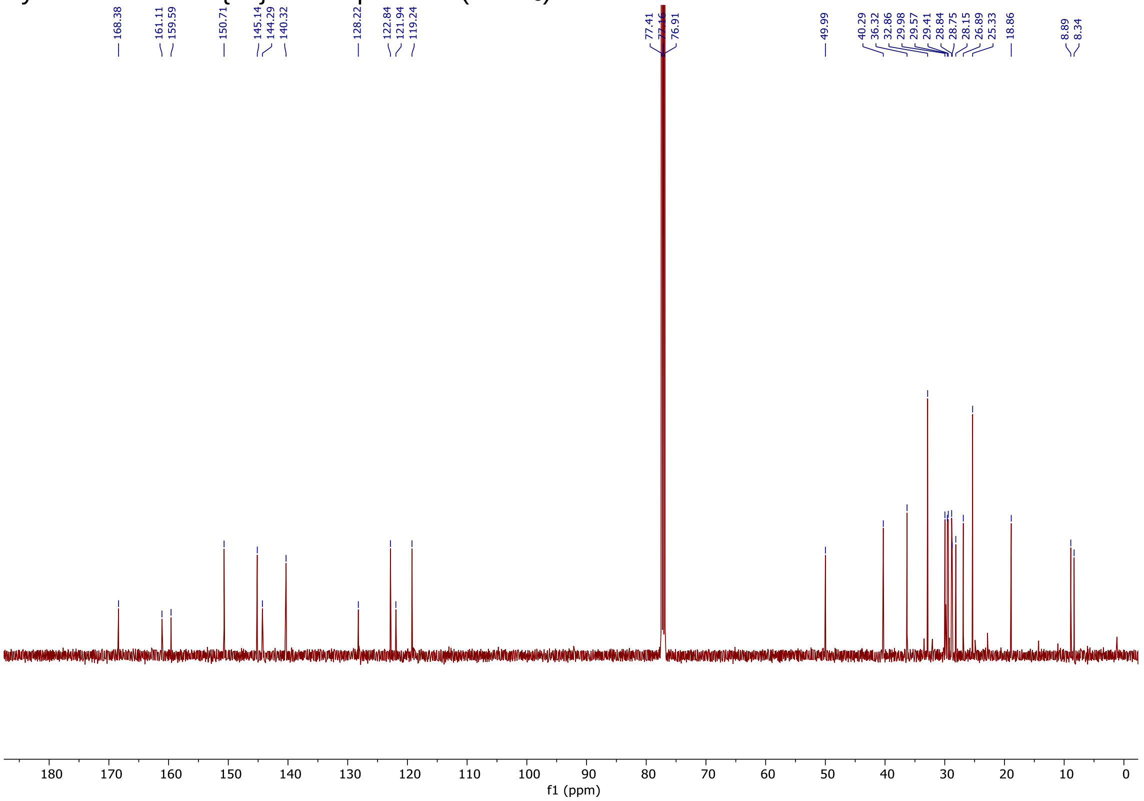

(6-Cyclobutylhexyl)benzene (**12v**) – ^{1}H NMR spectrum ($CDCl_3$)

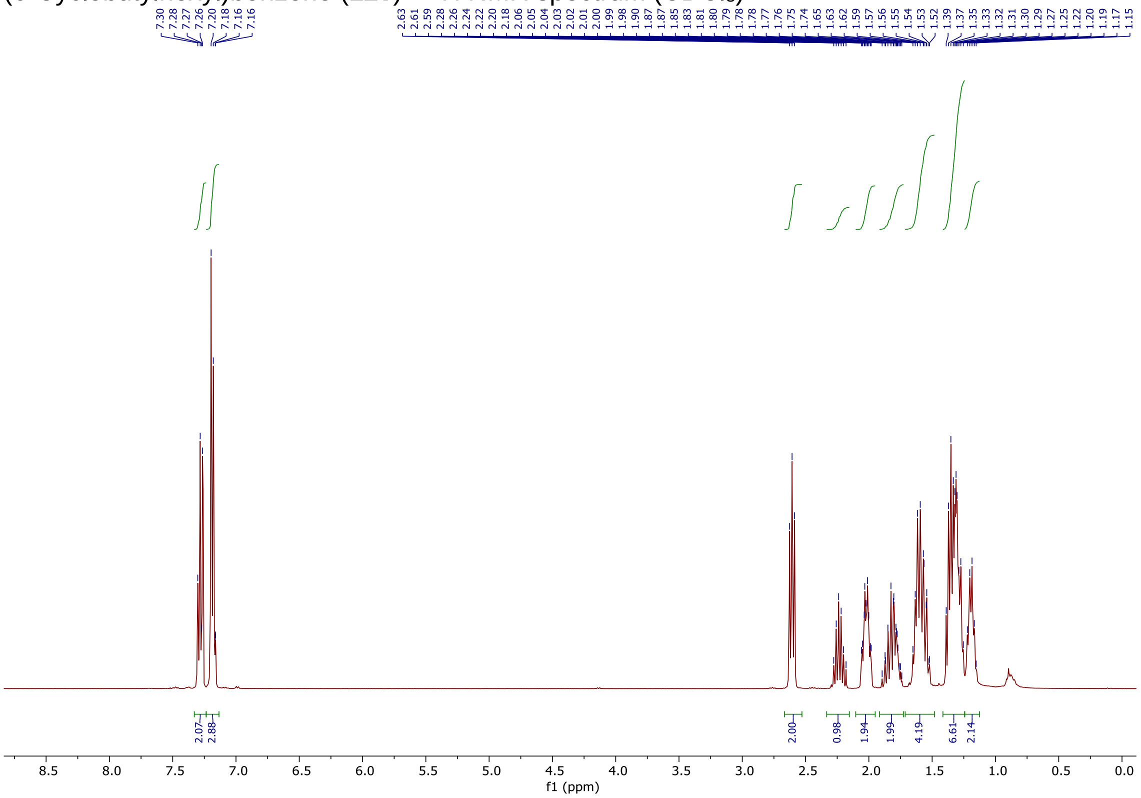

(6-Cyclobutylhexyl)benzene (**12v**) – $^{13}C\{^{1}H\}$ NMR spectrum ($CDCl_3$)

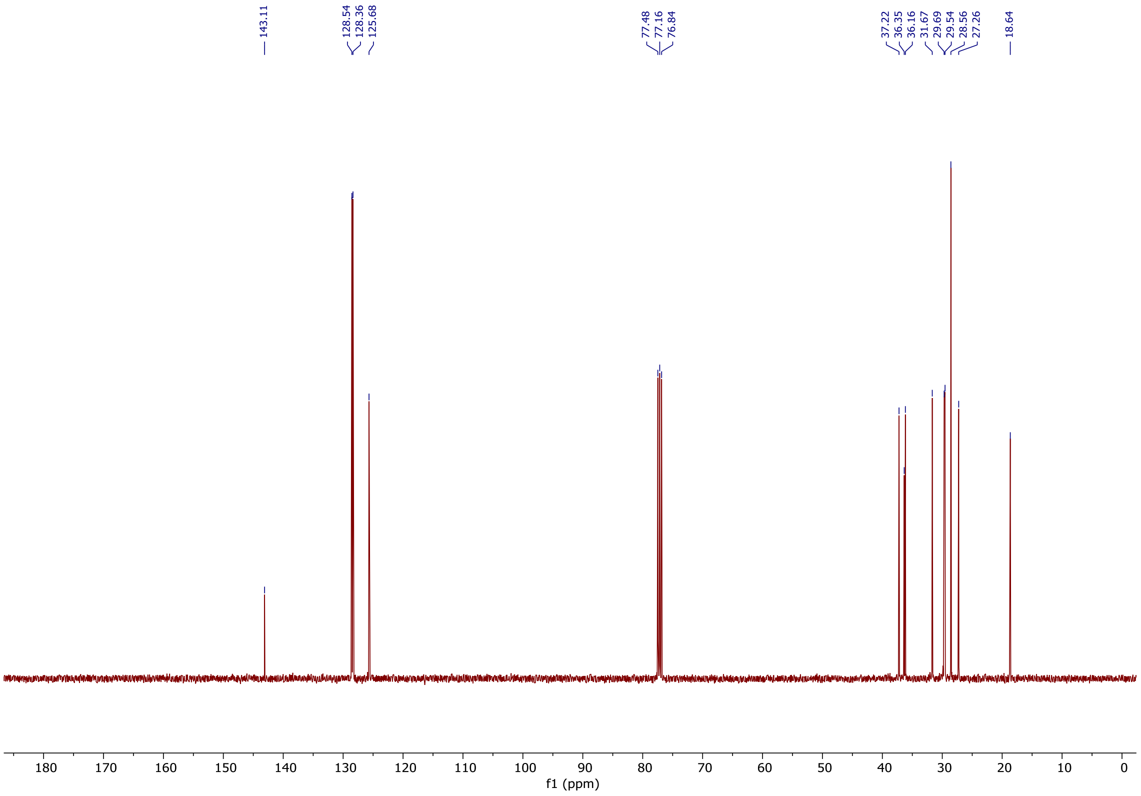

2-(6-Phenylhexyl)norbornane (**12w**) – ^{1}H NMR spectrum ($CDCl_3$)

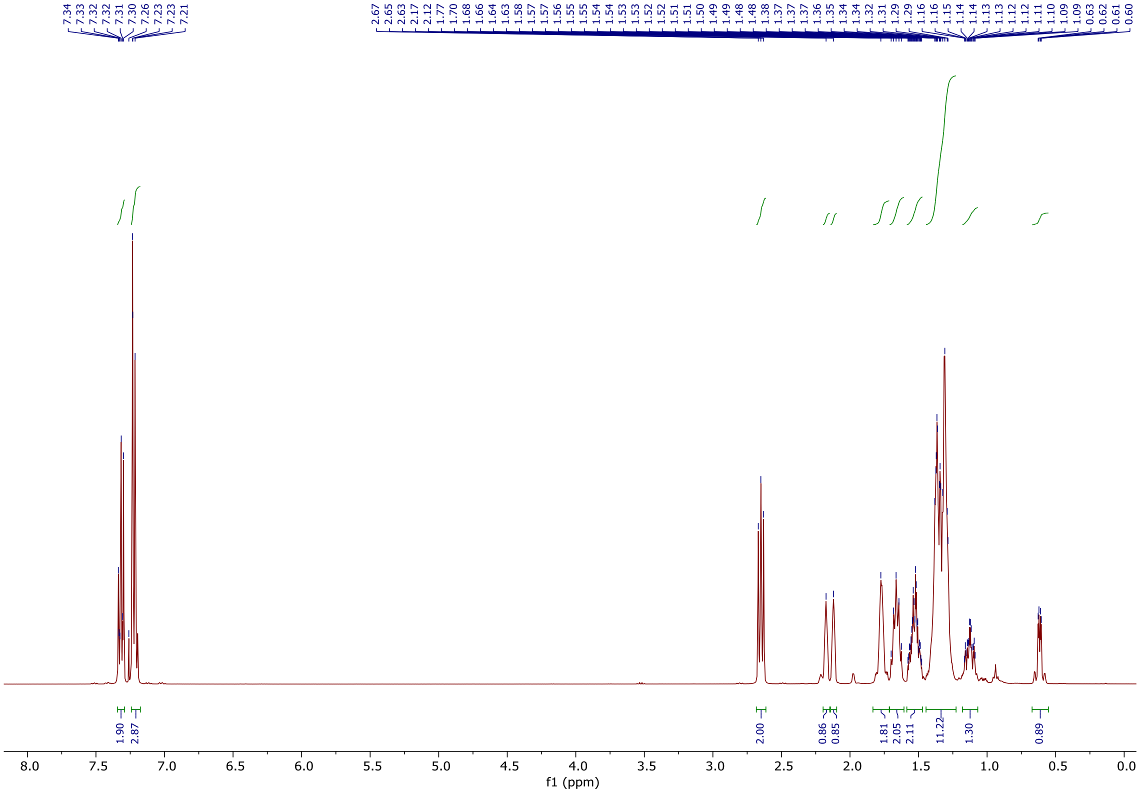

2-(6-Phenylhexyl)norbornane (**12w**) – $^{13}C\{^1H\}$ NMR spectrum ($CDCl_3$)

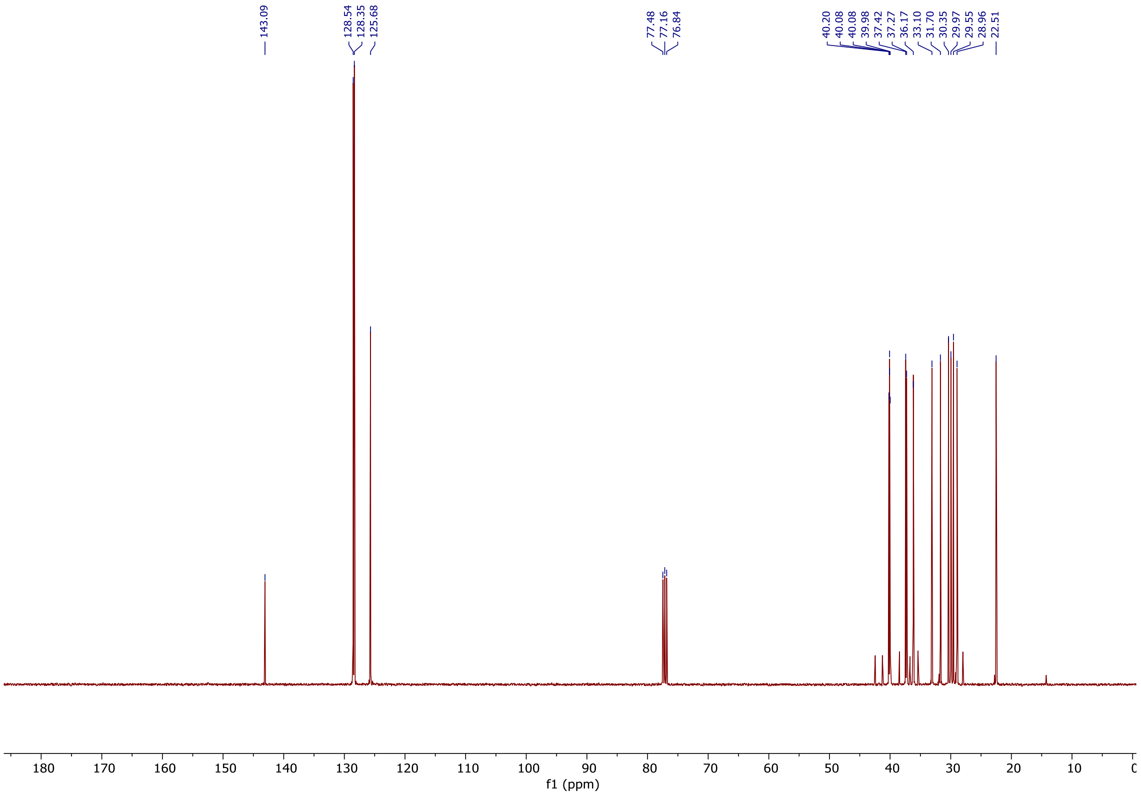

(6-Cyclohexylhexyl)benzene (**12x**) – ^1H NMR spectrum ($CDCl_3$)

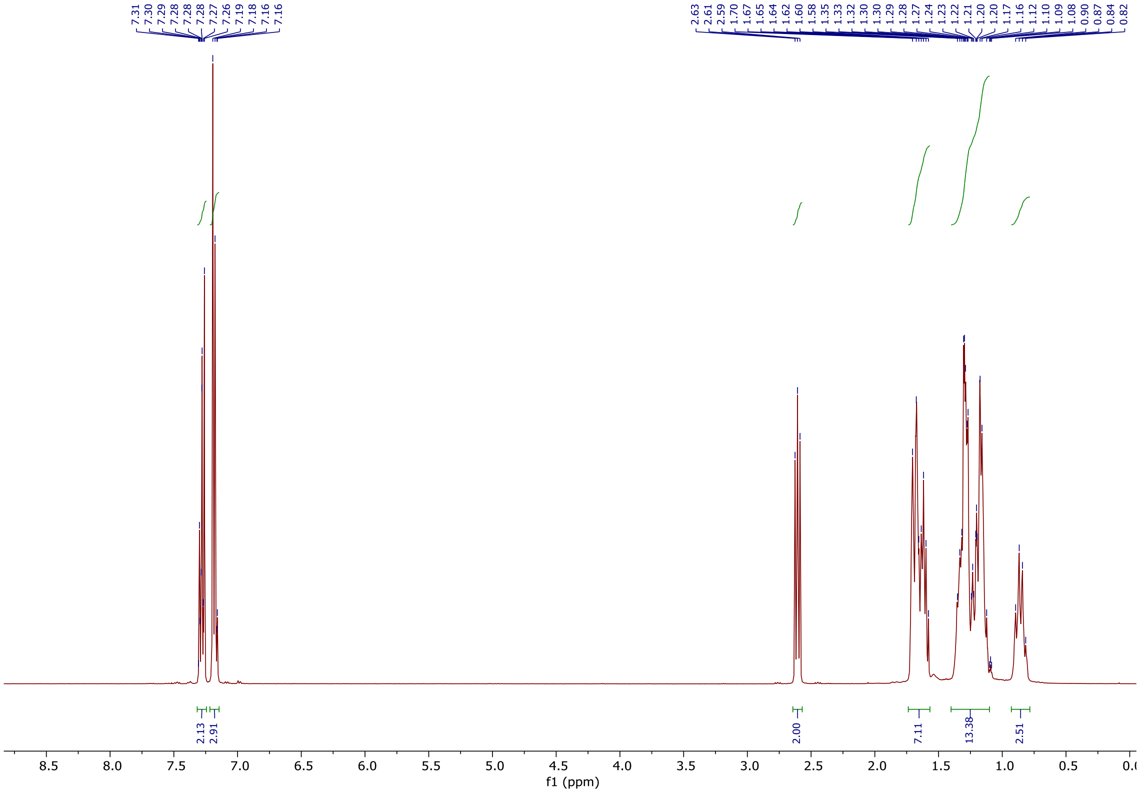

(6-Cyclohexylhexyl)benzene (**12x**) – $^{13}C\{^{1}H\}$ NMR spectrum ($CDCl_3$)

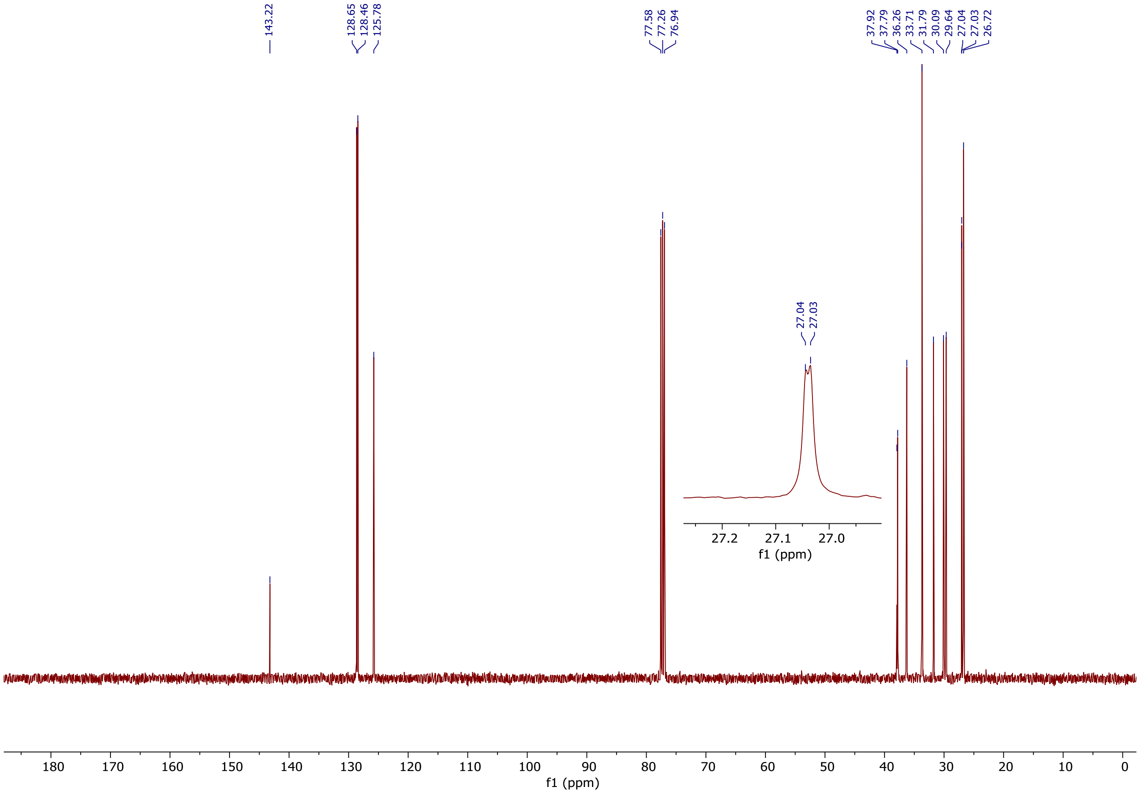

(6-Phenylhexyl)cycloheptane (**12y**) – ^{1}H NMR spectrum ($CDCl_3$)

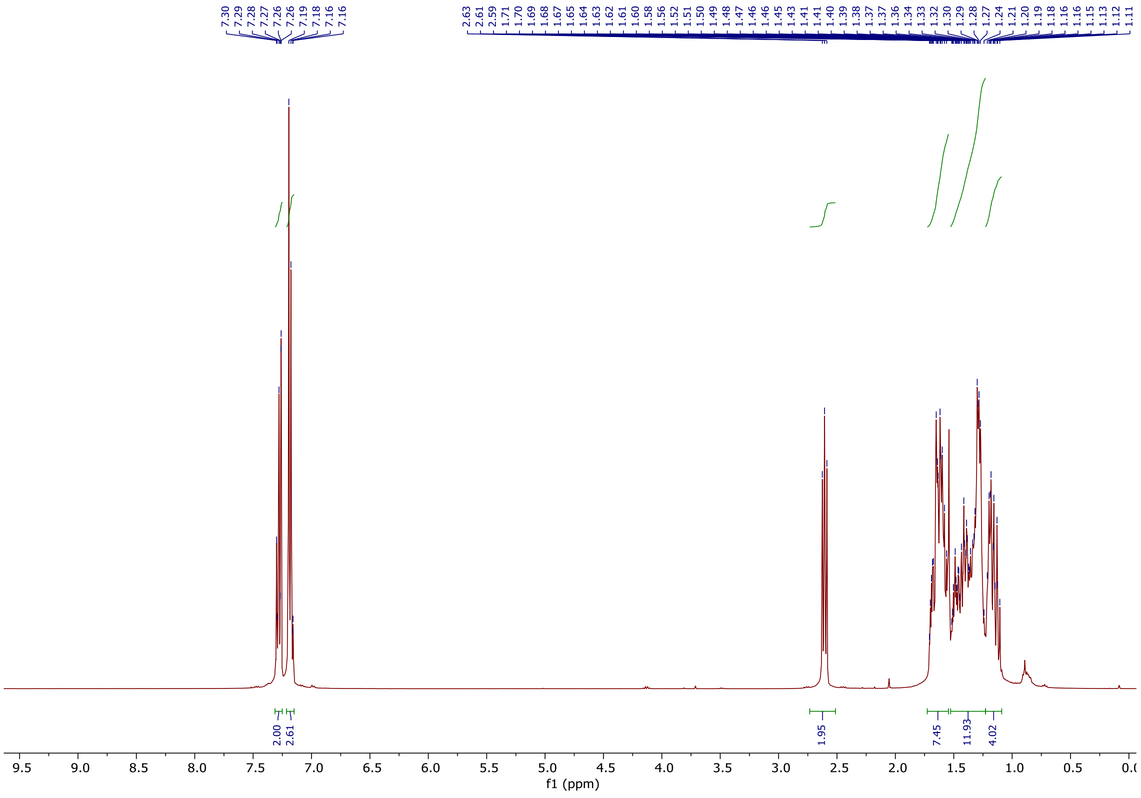

(6-Phenylhexyl)cycloheptane (**12y**) – $^{13}C\{^{1}H\}$ NMR spectrum ($CDCl_3$)

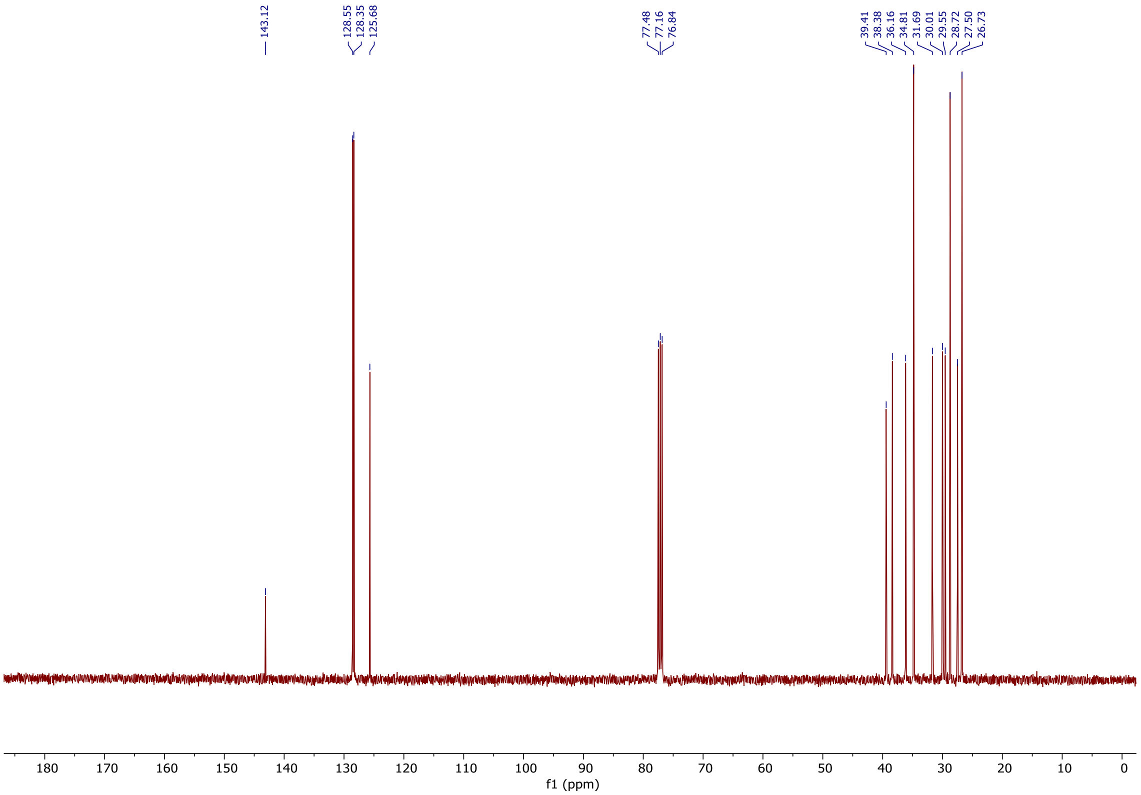

Ether **12z** – ^{1}H NMR spectrum ($CDCl_3$)

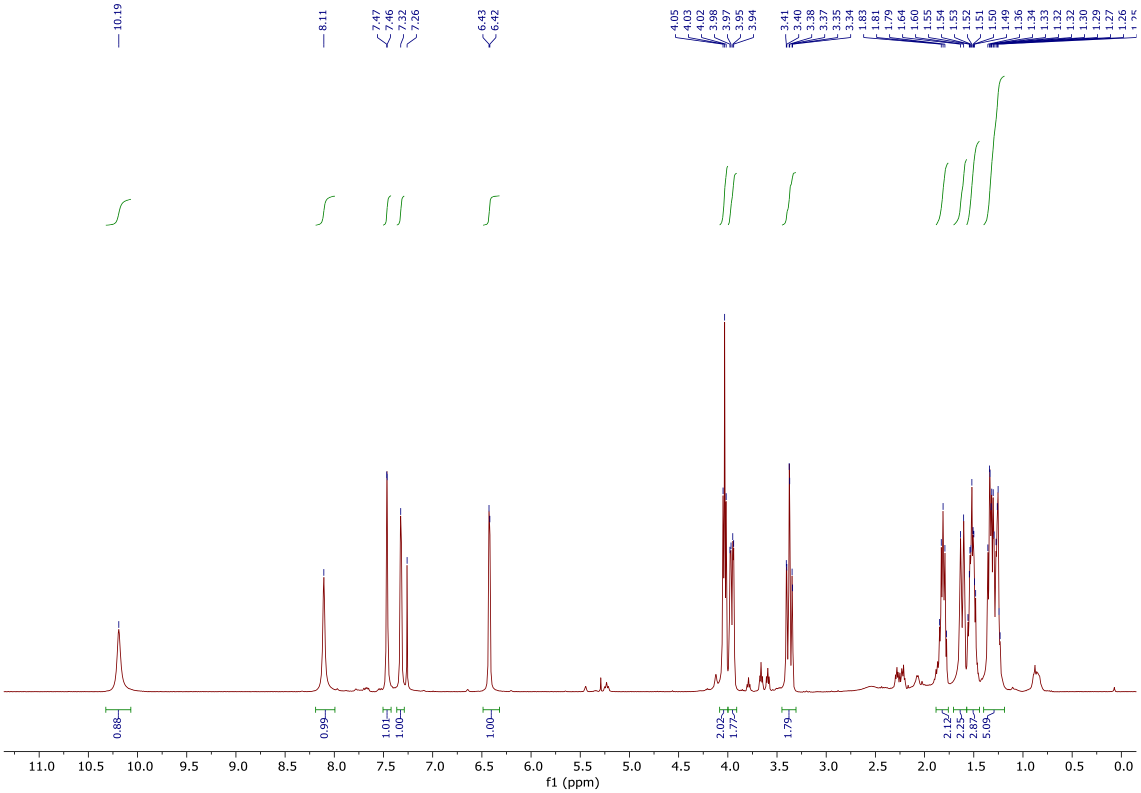

Ether **12z** – $^{13}C\{^{1}H\}$ NMR spectrum ($CDCl_3$)

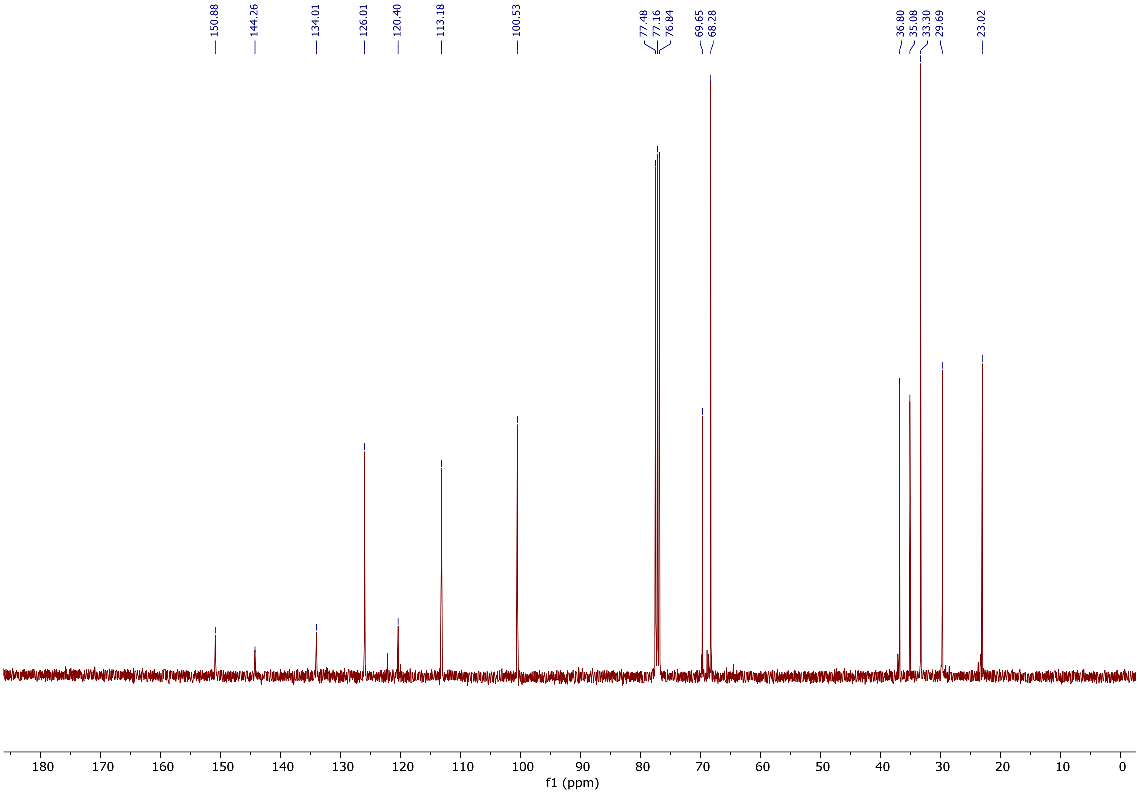

(7-Methyloctyl)benzene (**12aa**) – ^{1}H NMR spectrum ($CDCl_3$)

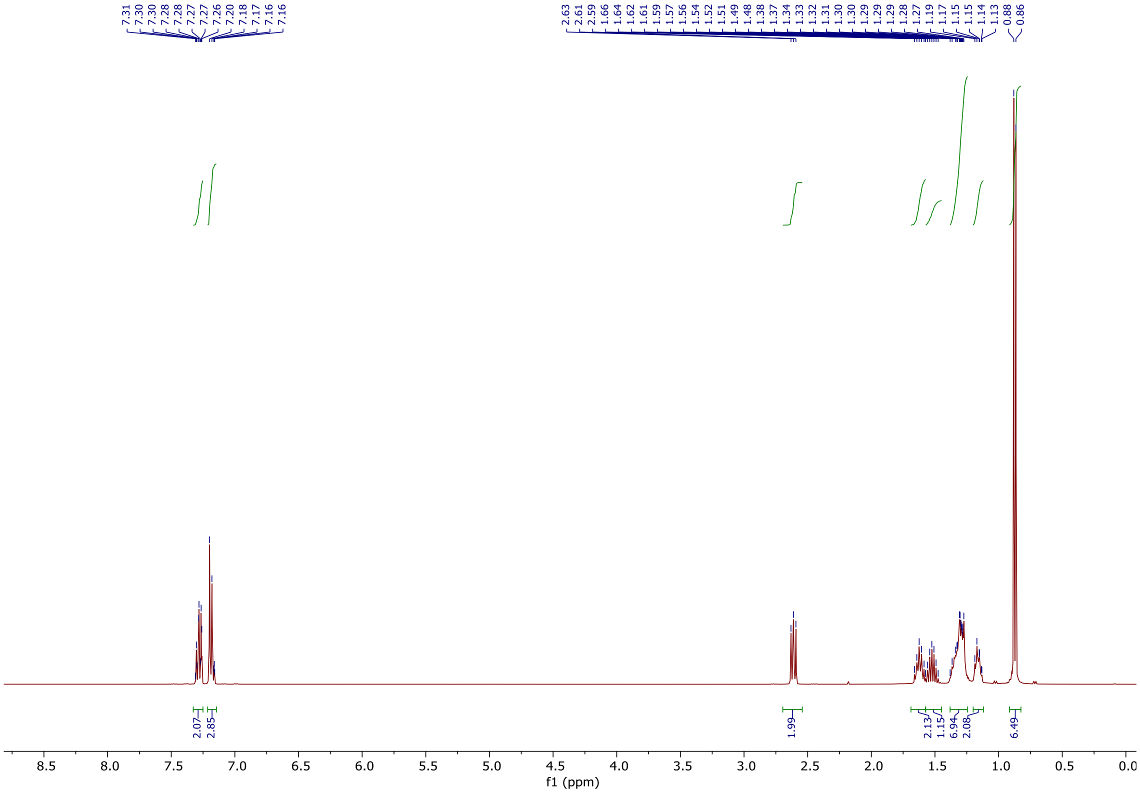

(7-Methyloctyl)benzene (**12aa**) – $^{13}C\{^1H\}$ NMR spectrum ($CDCl_3$)

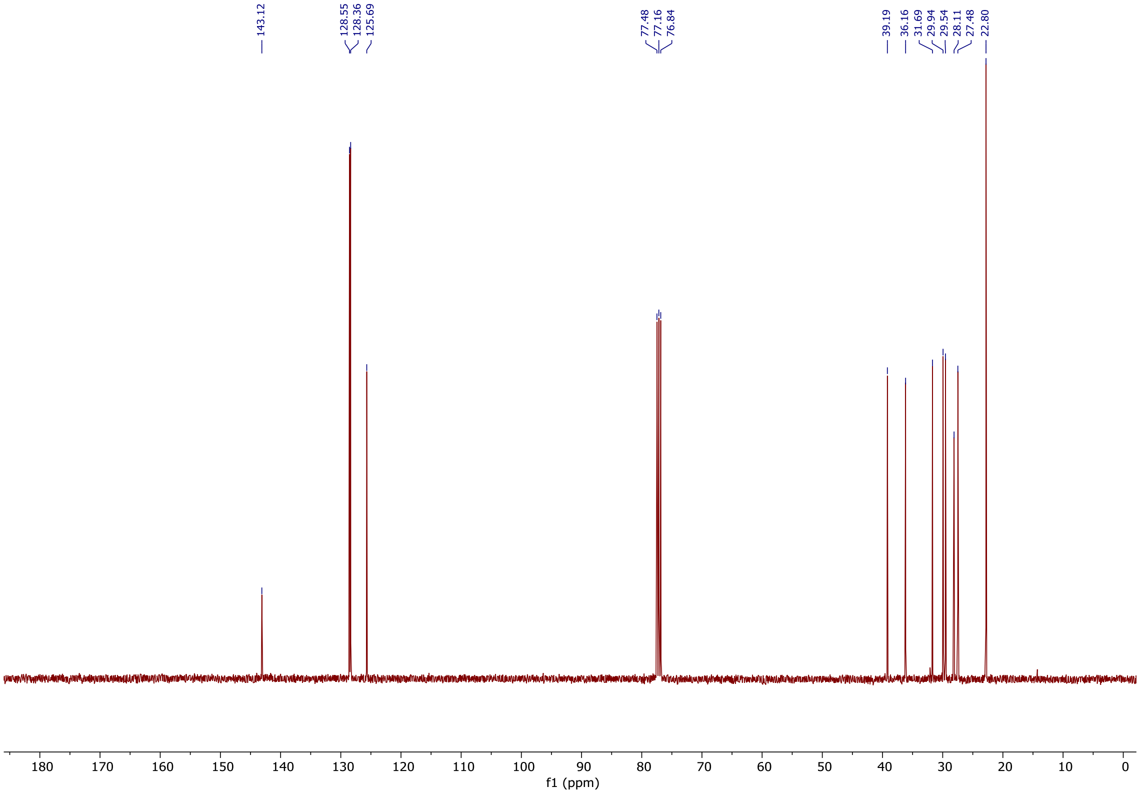

(4-Methylheptane-1,7-diyl)dibenzene (**12ab**) – ^1H NMR spectrum ($CDCl_3$)

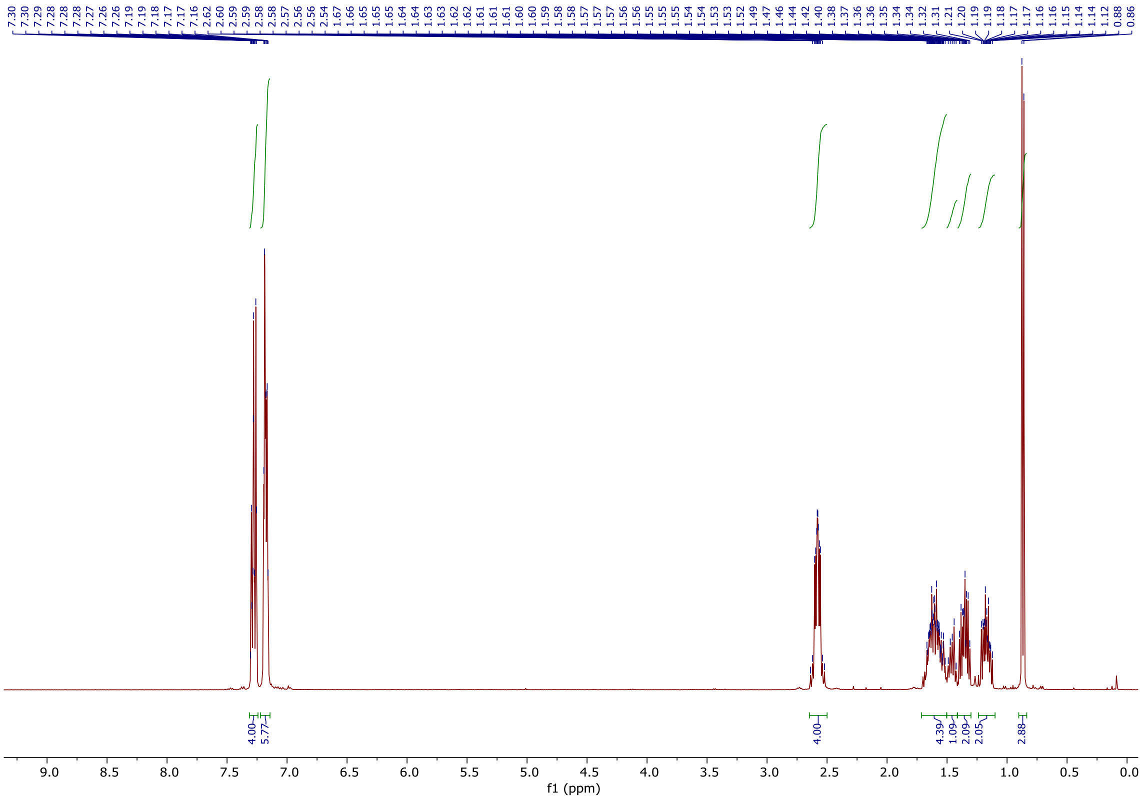

(4-Methylheptane-1,7-diyl)dibenzene (**12ab**) – $^{13}C\{^{1}H\}$ NMR spectrum ($CDCl_3$)

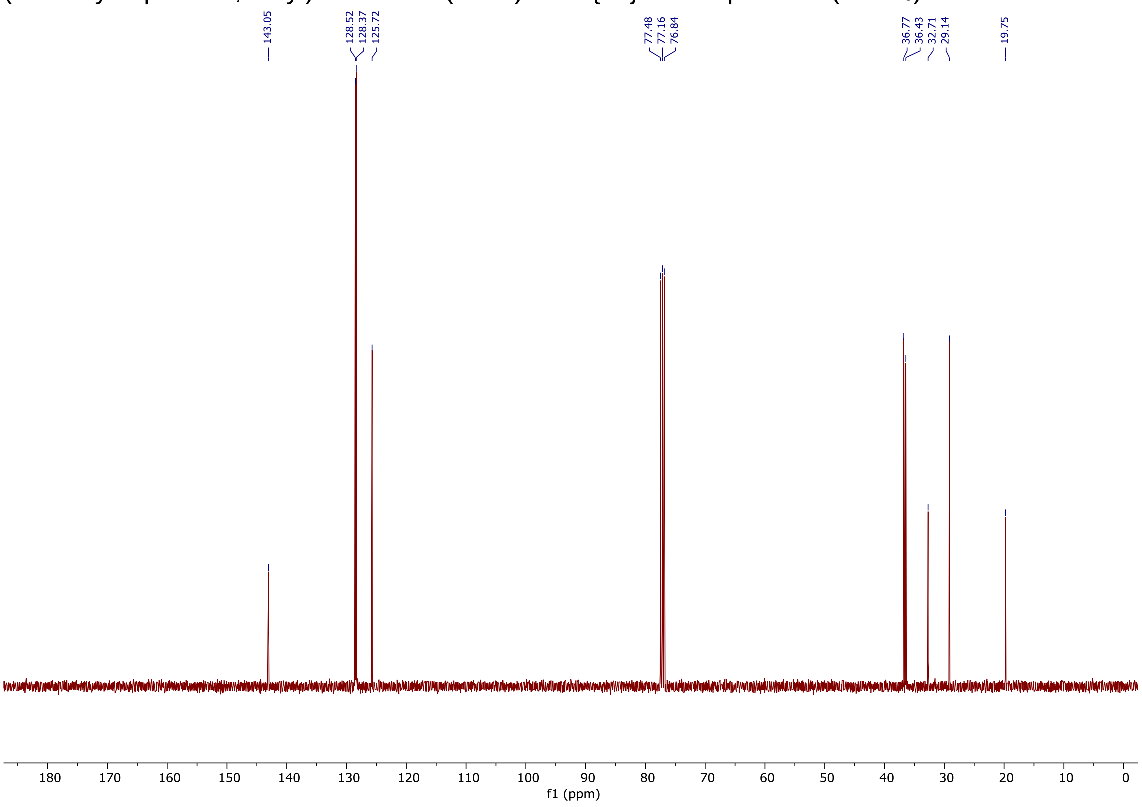

1-Bromo-4-(4-methyl-7-phenylheptyl)benzene **(12ac)** – ^{1}H NMR spectrum ($CDCl_3$)

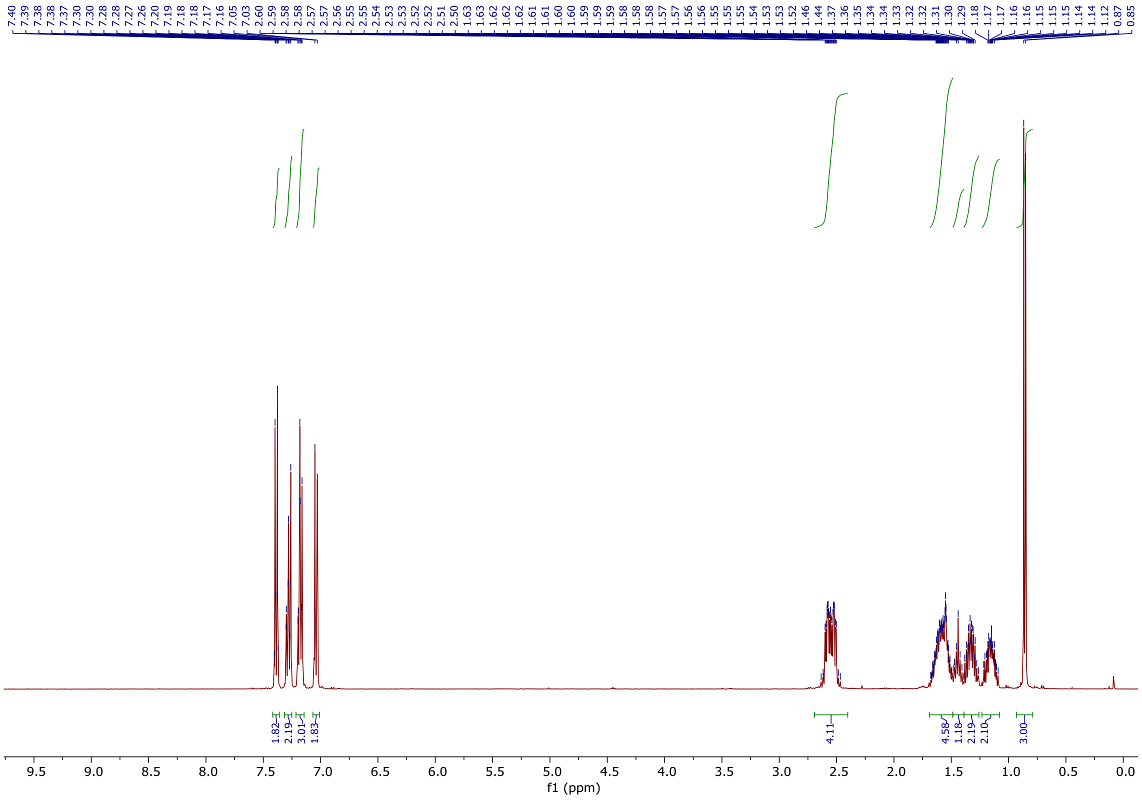

1-Bromo-4-(4-methyl-7-phenylheptyl)benzene **(12ac)** – $^{13}C\{^{1}H\}$ NMR spectrum ($CDCl_3$)

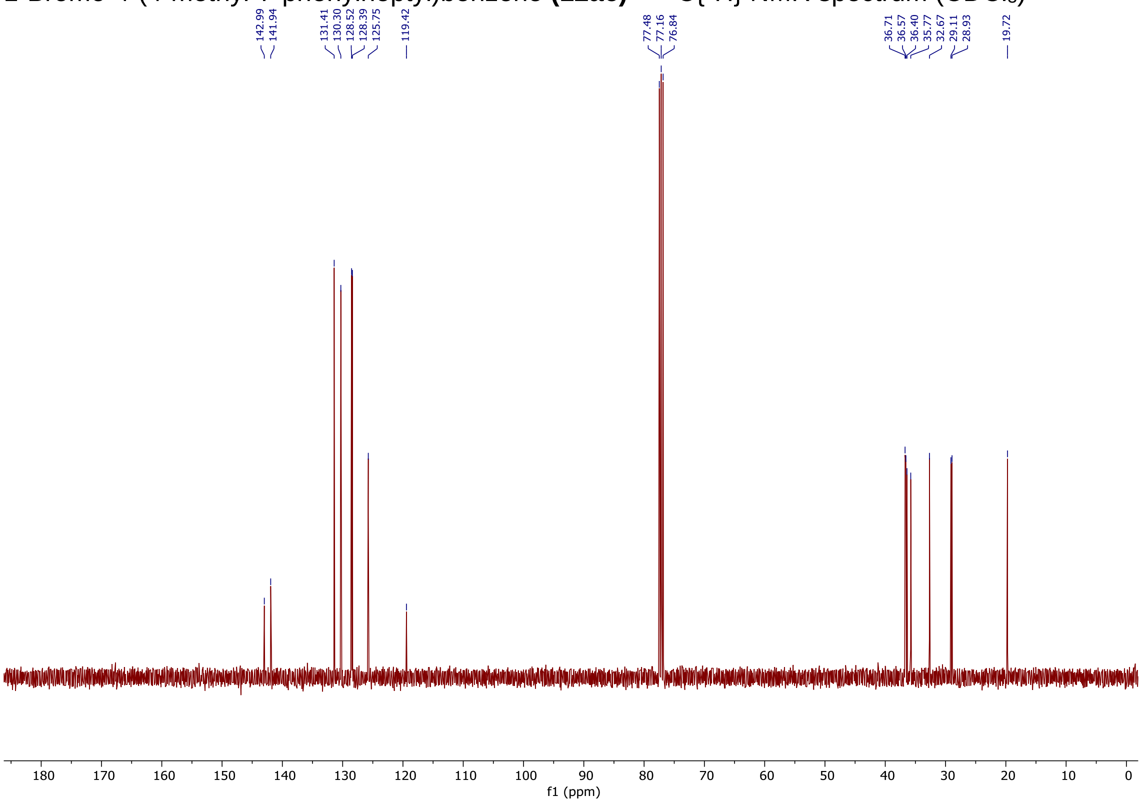

tert-Butyl((4-methyl-7-phenylheptyl)oxy)diphenylsilane (**12ad**) – ^{1}H NMR spectrum ($CDCl_3$)

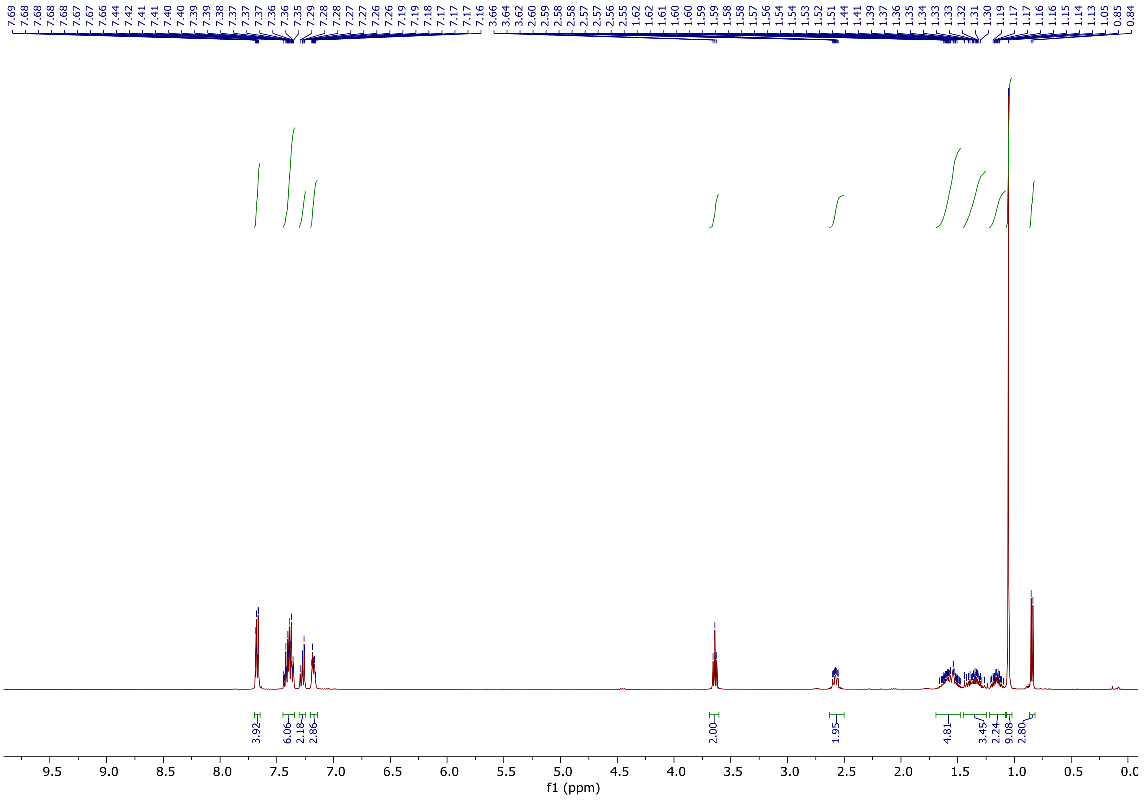

tert-Butyl((4-methyl-7-phenylheptyl)oxy)diphenylsilane (**12ad**) – $^{13}C\{^1H\}$ NMR spectrum ($CDCl_3$)

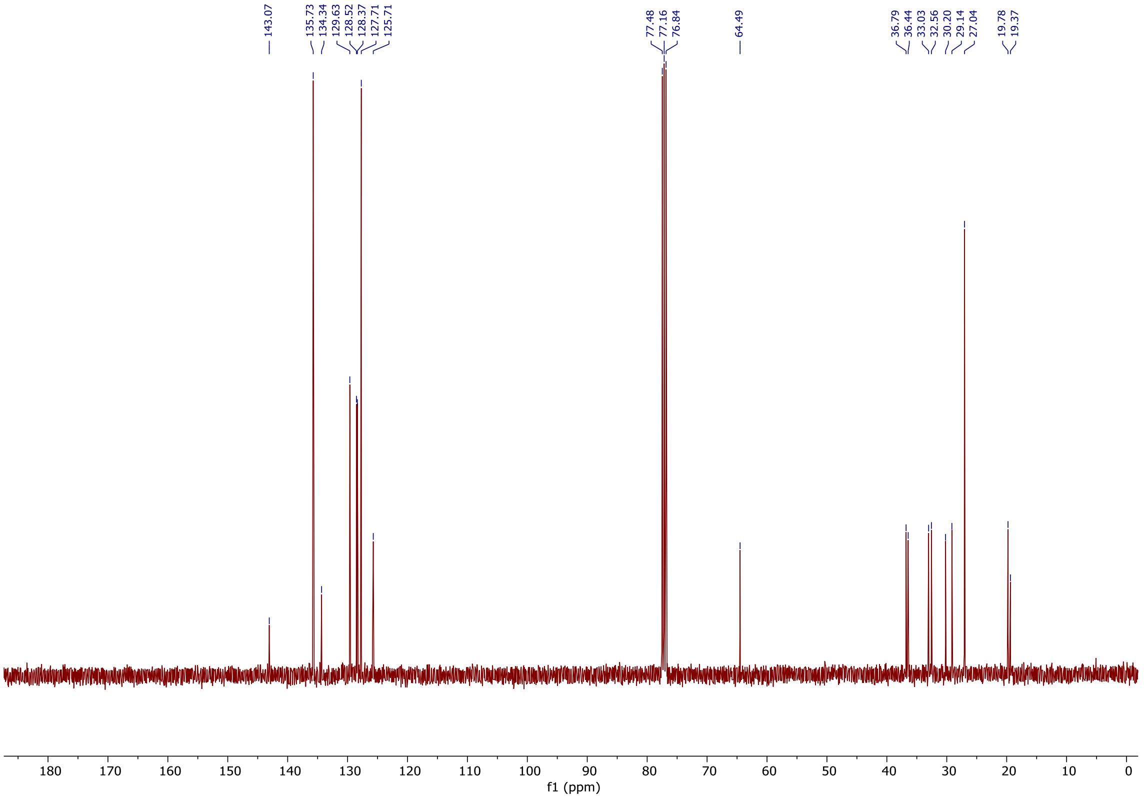

tert-Butyl((4-methylpentyl)oxy)diphenylsilane (**12ae**) – ^1H NMR spectrum ($CDCl_3$)

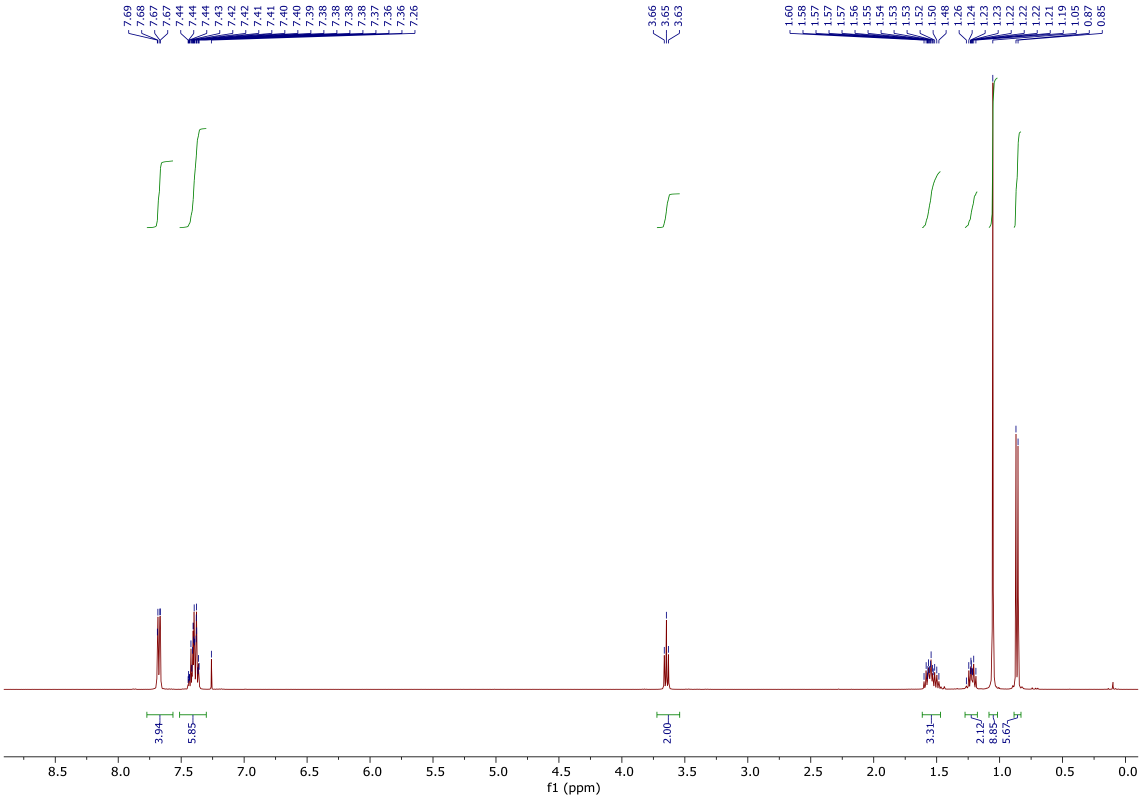

tert-Butyl((4-methylpentyl)oxy)diphenylsilane (**12ae**) – $^{13}C\{^1H\}$ NMR spectrum ($CDCl_3$)

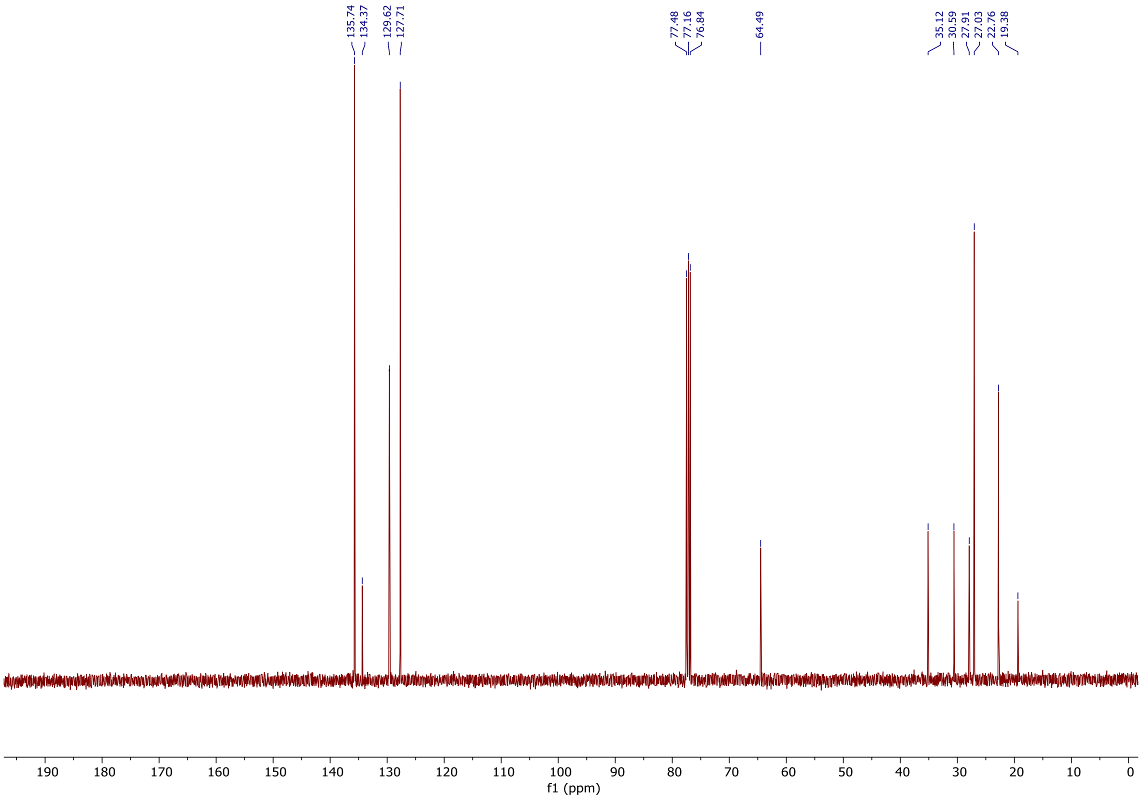

5-((5-Methyl-8-(phenylthio)octyl)oxy)-1H-pyrrolo[2,3-b]pyridine (**12af**) – ^1H NMR spectrum ($CDCl_3$)

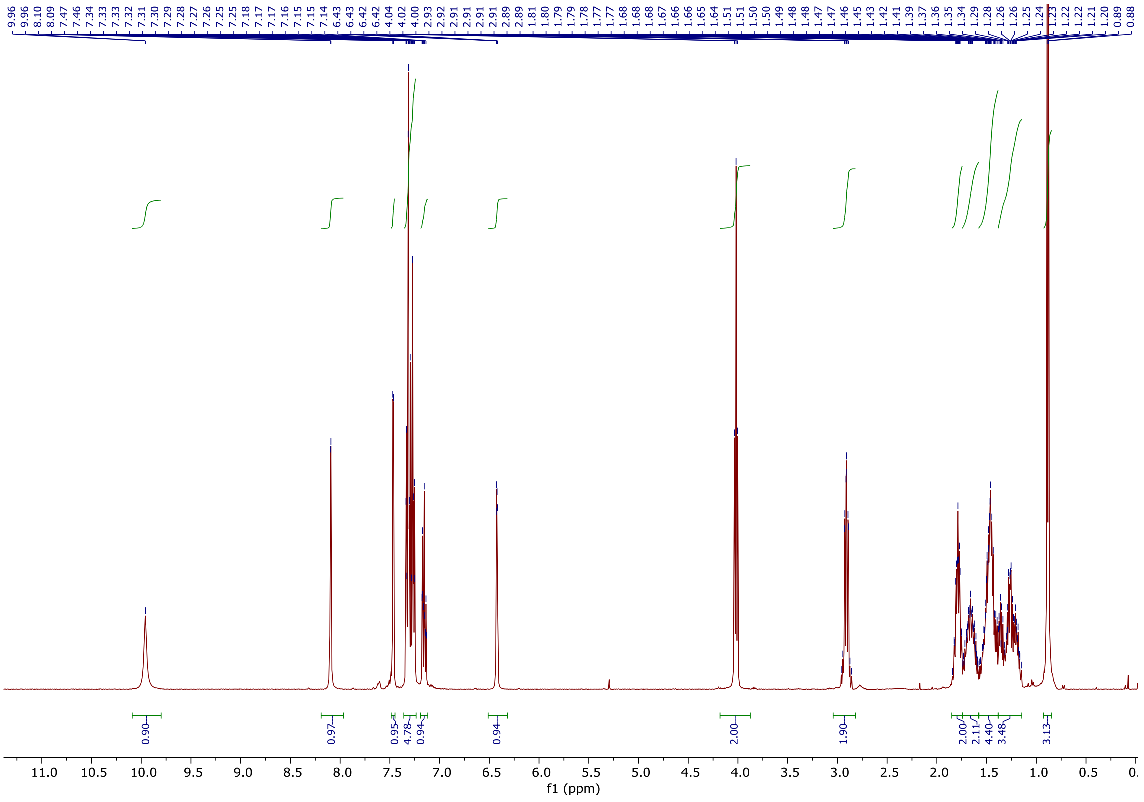

5-((5-Methyl-8-(phenylthio)octyl)oxy)-1H-pyrrolo[2,3-b]pyridine (**12af**) – $^{13}C\{^{1}H\}$ NMR spectrum ($CDCl_3$)

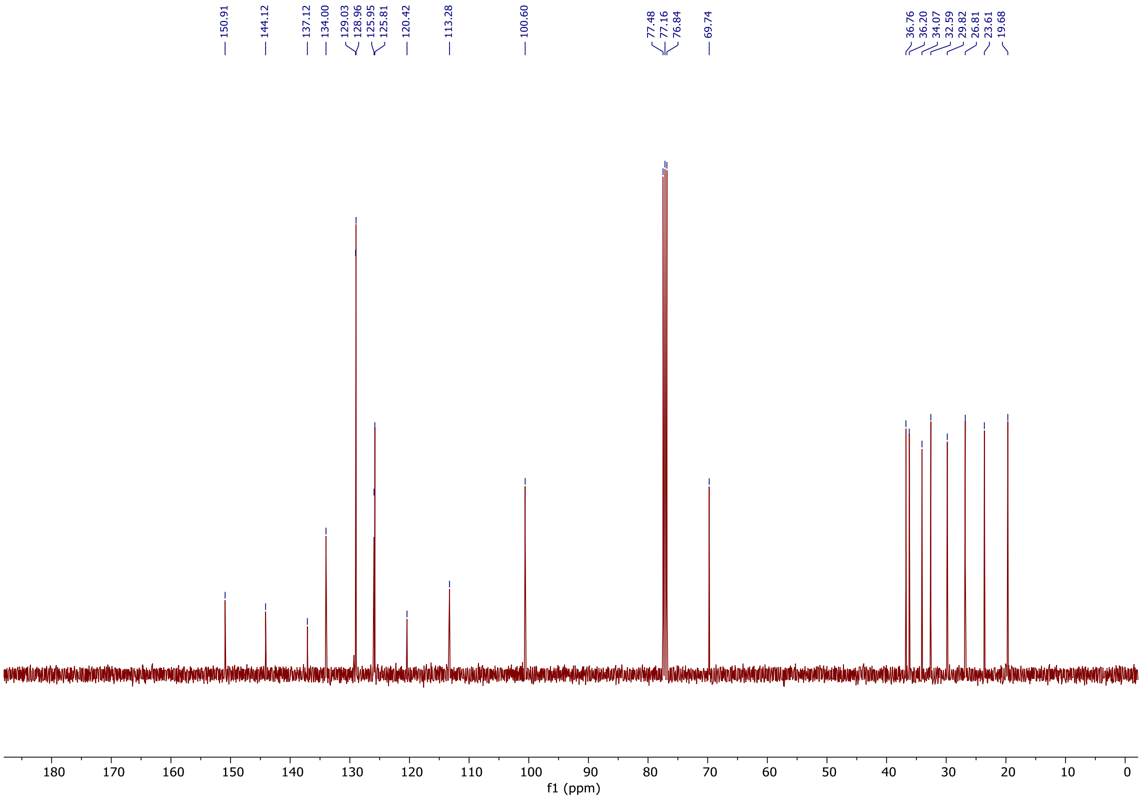

Pyrimidine **12ag** – ^{1}H NMR spectrum ($CDCl_3$)

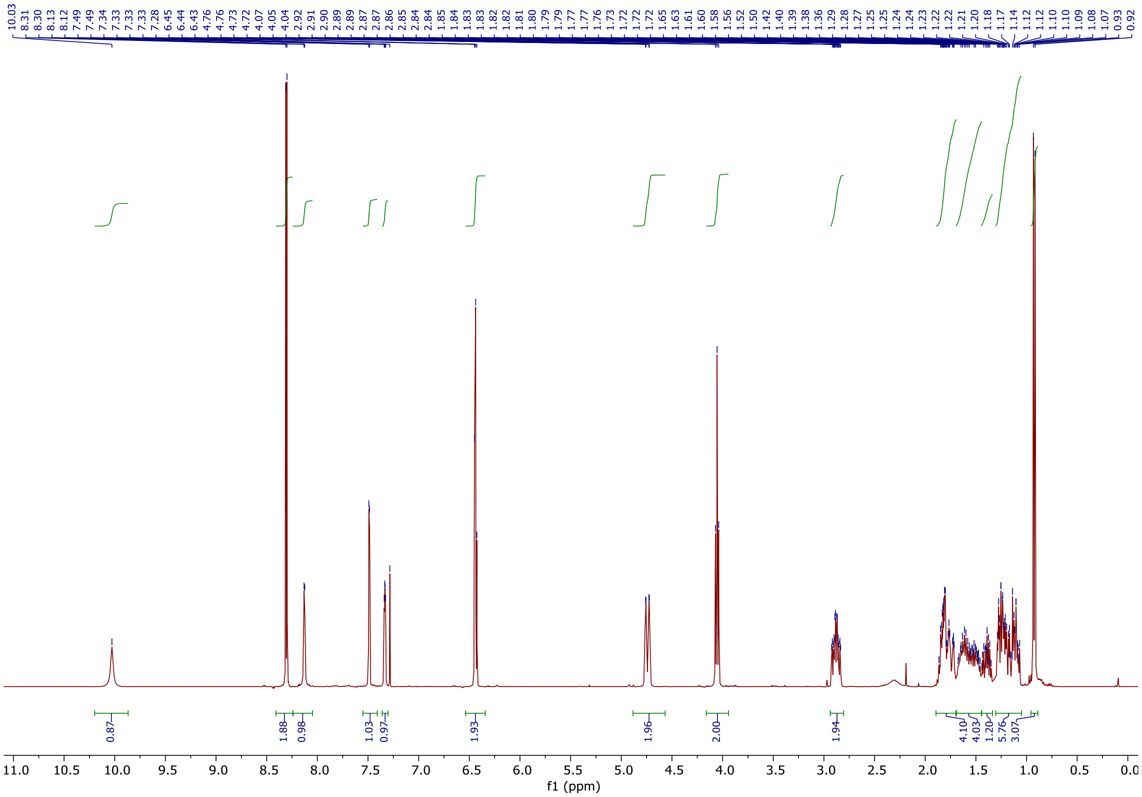

Pyrimidine **12ag** – $^{13}C\{^{1}H\}$ NMR spectrum ($CDCl_3$)

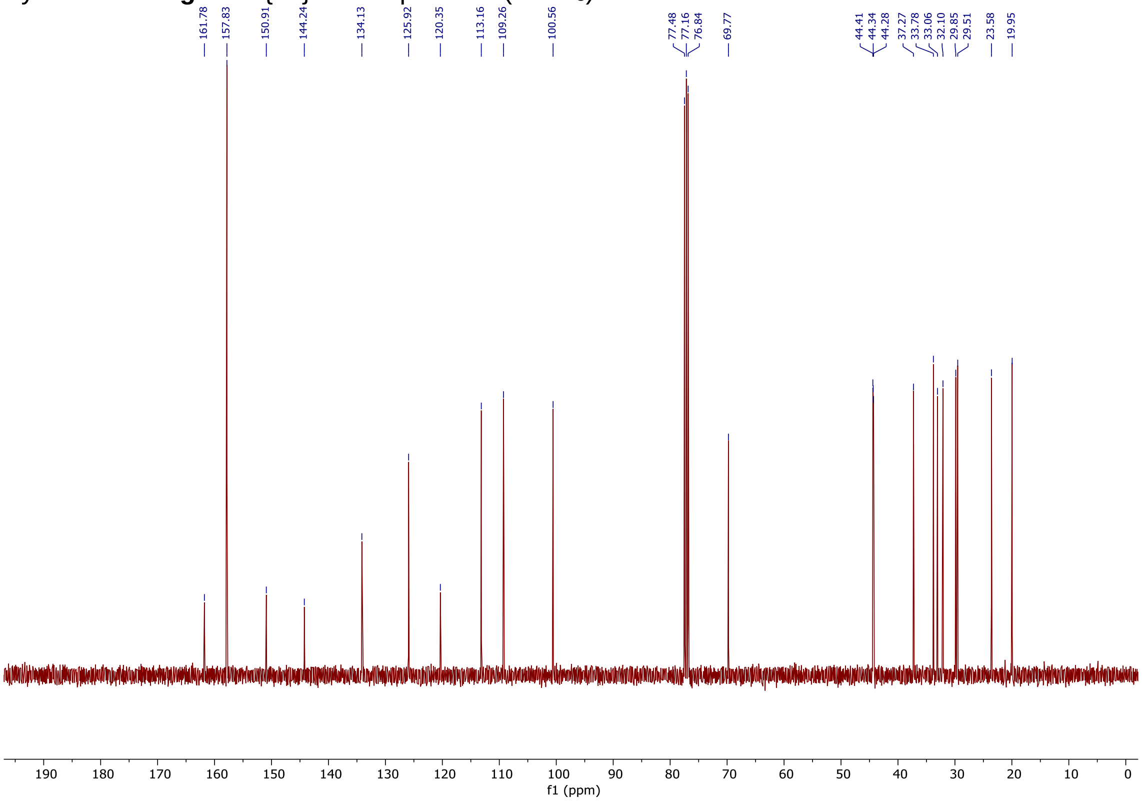

Triphenyl(4-phenylbutyl)phosphonium bromide (**13**) – ^{1}H NMR spectrum ($CDCl_3$)

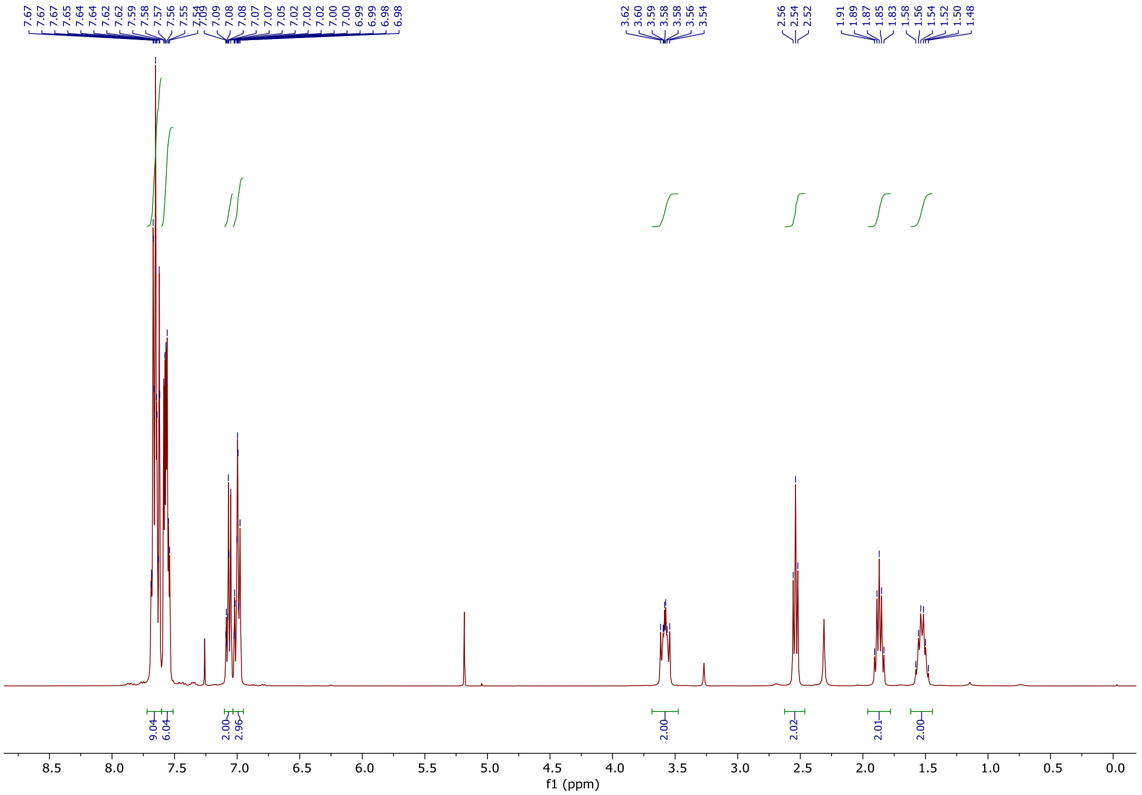

Triphenyl(4-phenylbutyl)phosphonium bromide (**13**) – $^{13}C\{^1H\}$ NMR spectrum ($CDCl_3$)

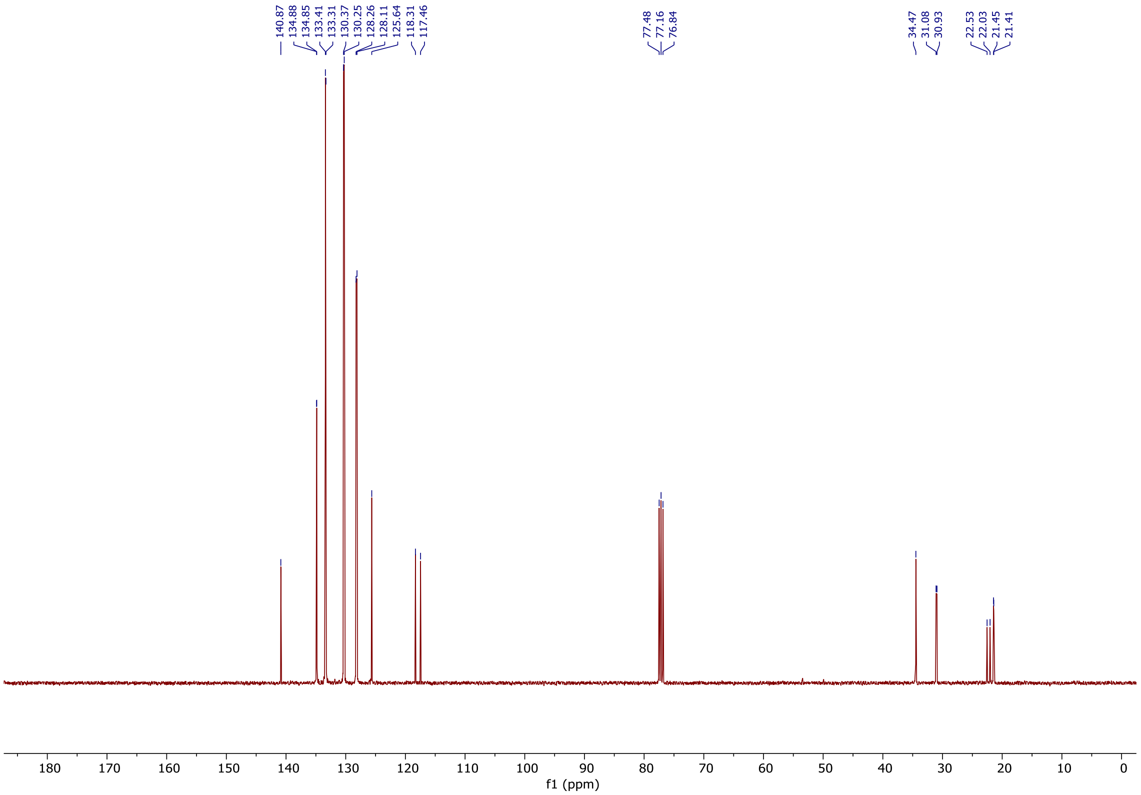

Triphenyl(4-phenylbutyl)phosphonium bromide (**13**) – $^{31}P\{^1H\}$ NMR spectrum ($CDCl_3$)

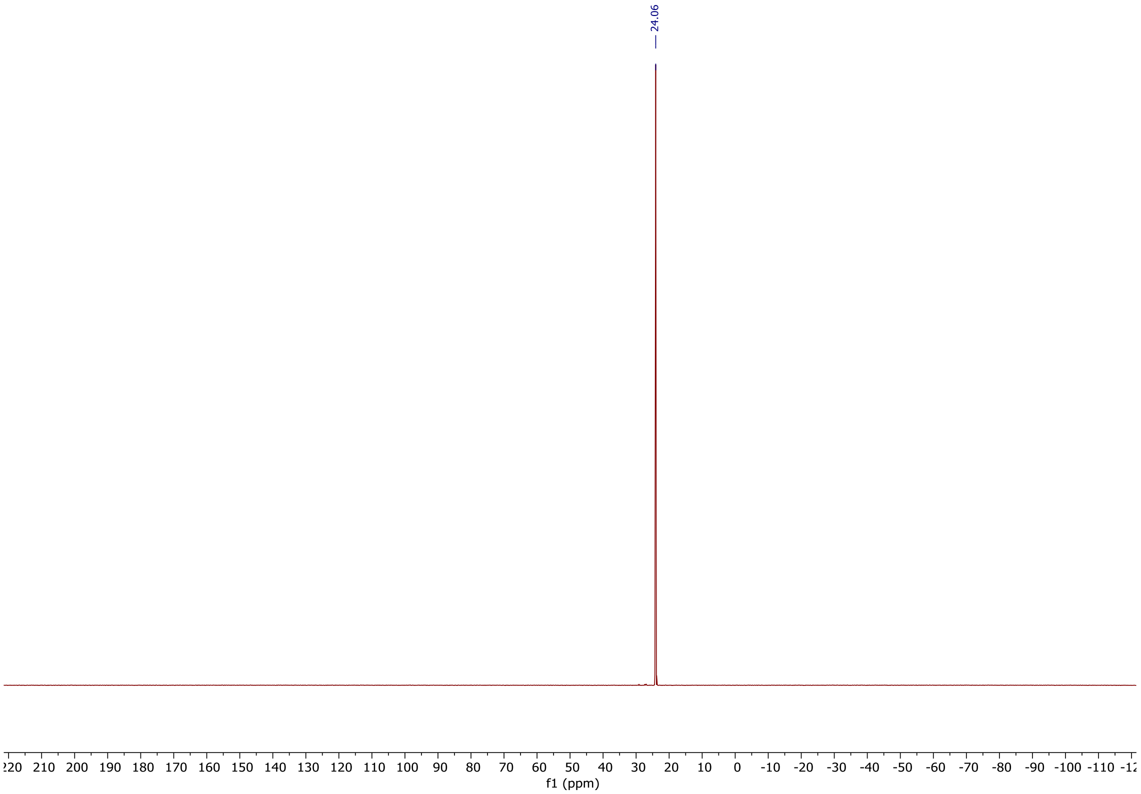

1-Methoxy-4-(2-(1-(3-phenylpropyl)cyclopentyl)ethyl)benzene (**15**) – ^{1}H NMR spectrum ($CDCl_3$)

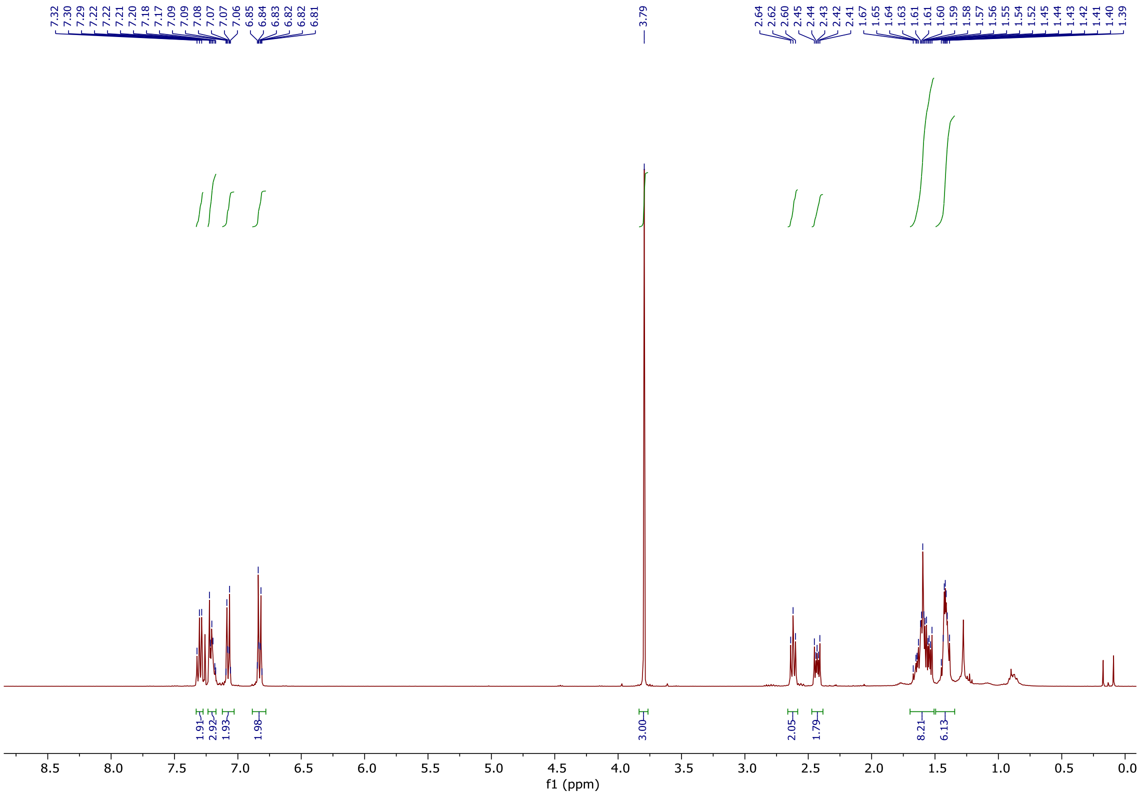

1-Methoxy-4-(2-(1-(3-phenylpropyl)cyclopentyl)ethyl)benzene (**15**) – $^{13}C\{^{1}H\}$ NMR spectrum ($CDCl_3$)

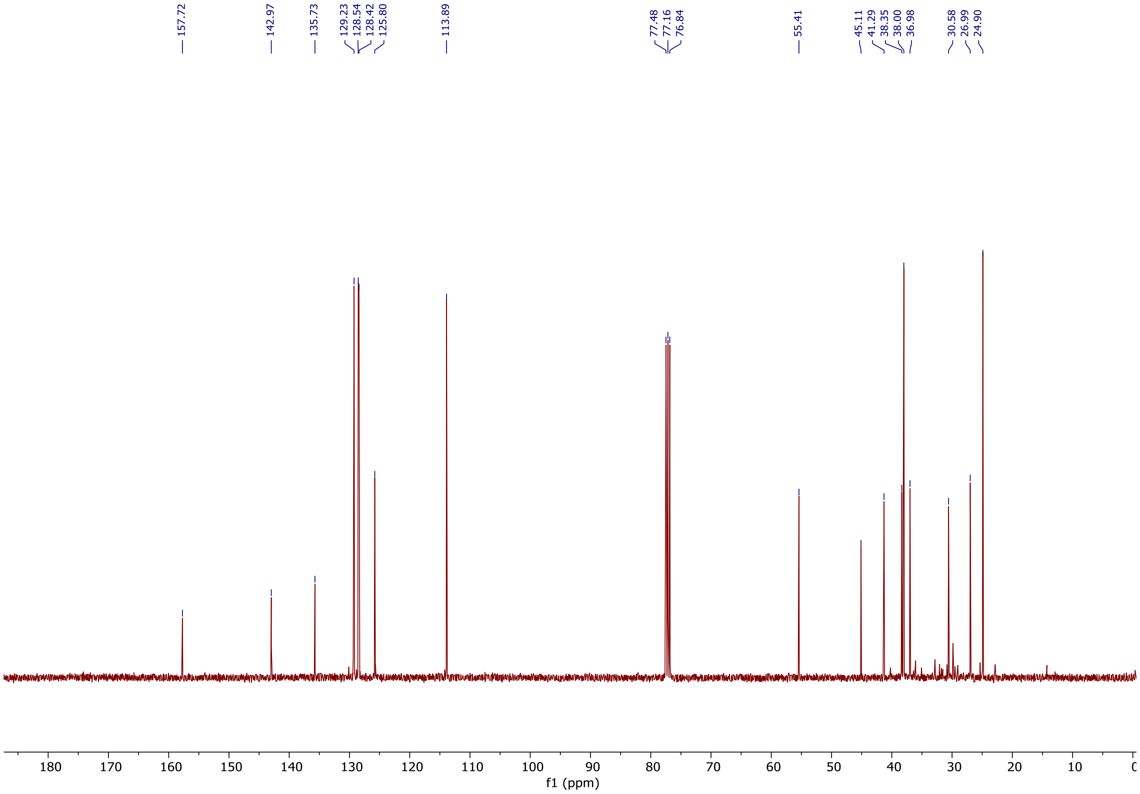

2-(1-(3-Phenylpropyl)cyclopentyl)quinoxaline (**18**) – ^{1}H NMR spectrum ($CDCl_3$)

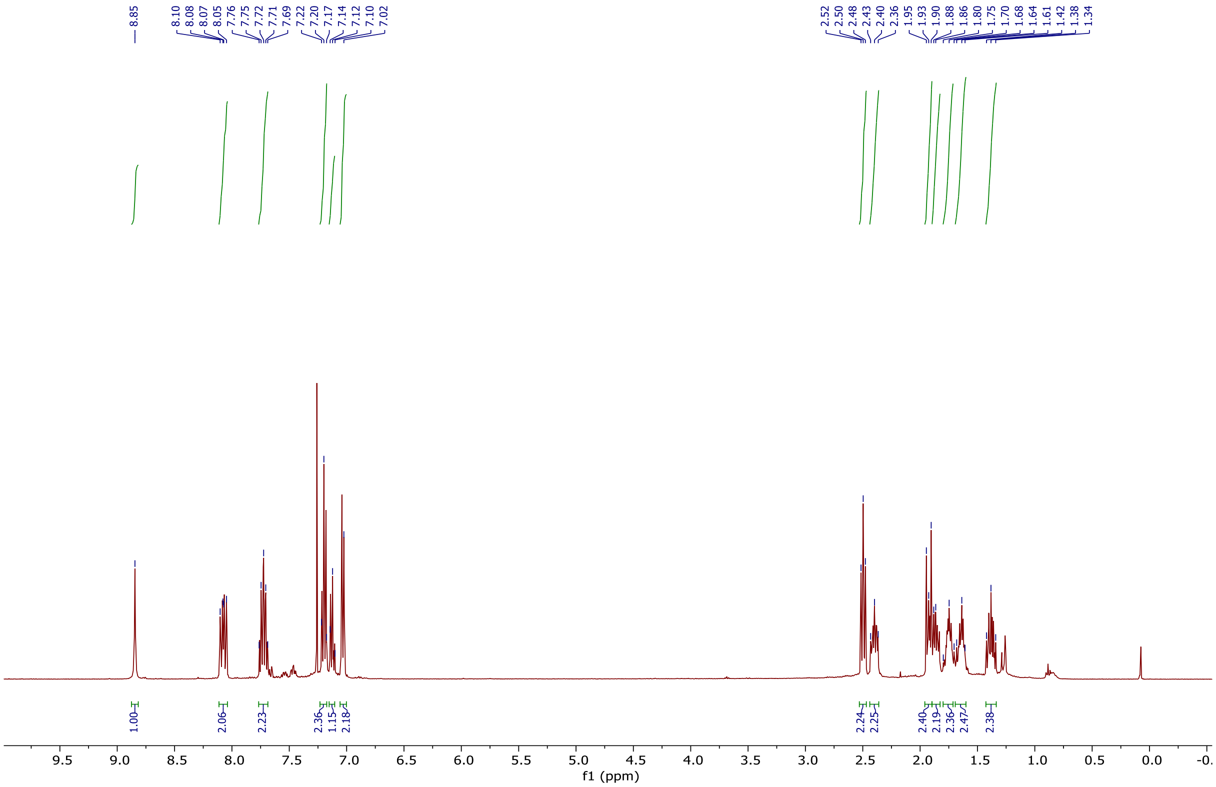

2-(1-(3-Phenylpropyl)cyclopentyl)quinoxaline (**18**) – ^{13}C{^{1}H} NMR spectrum ($CDCl_3$)

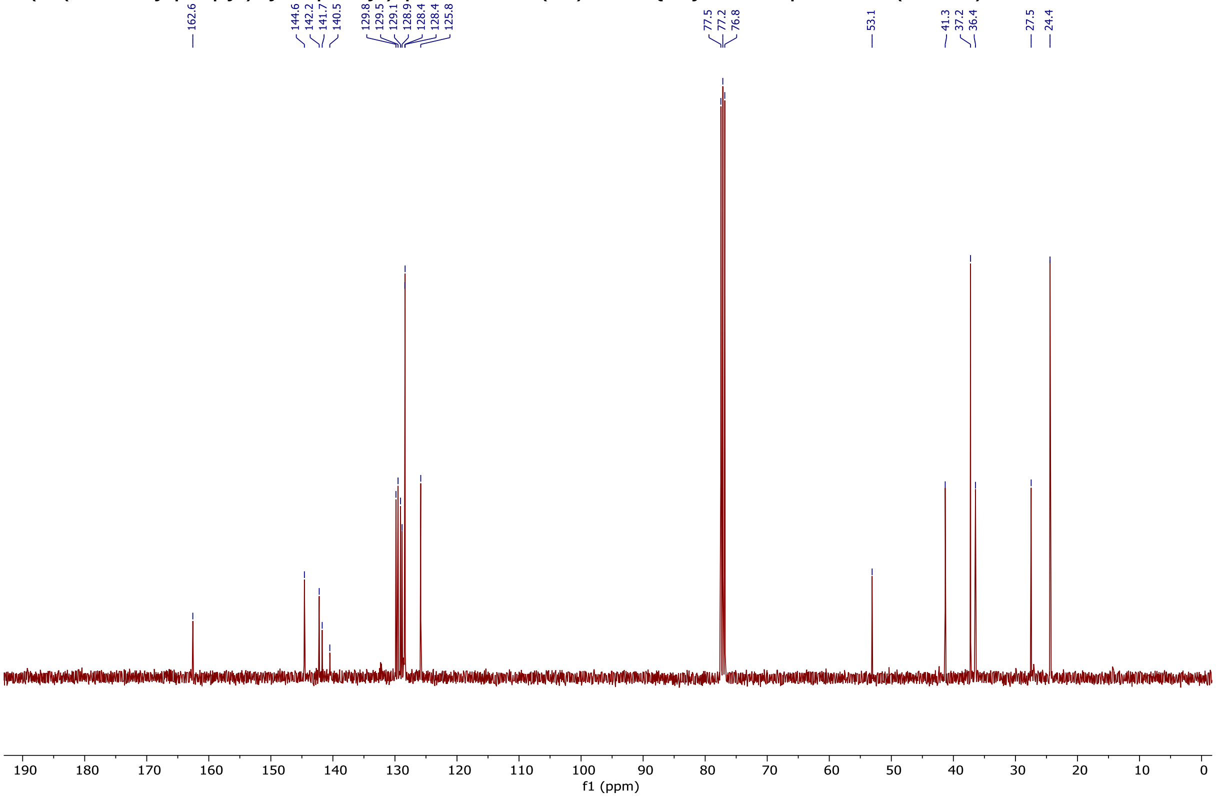

tert-Butyl 4-(2,2-diphenylethyl)piperidine-1-carboxylate (**21**) – ^{1}H NMR spectrum (CDCl$_3$)

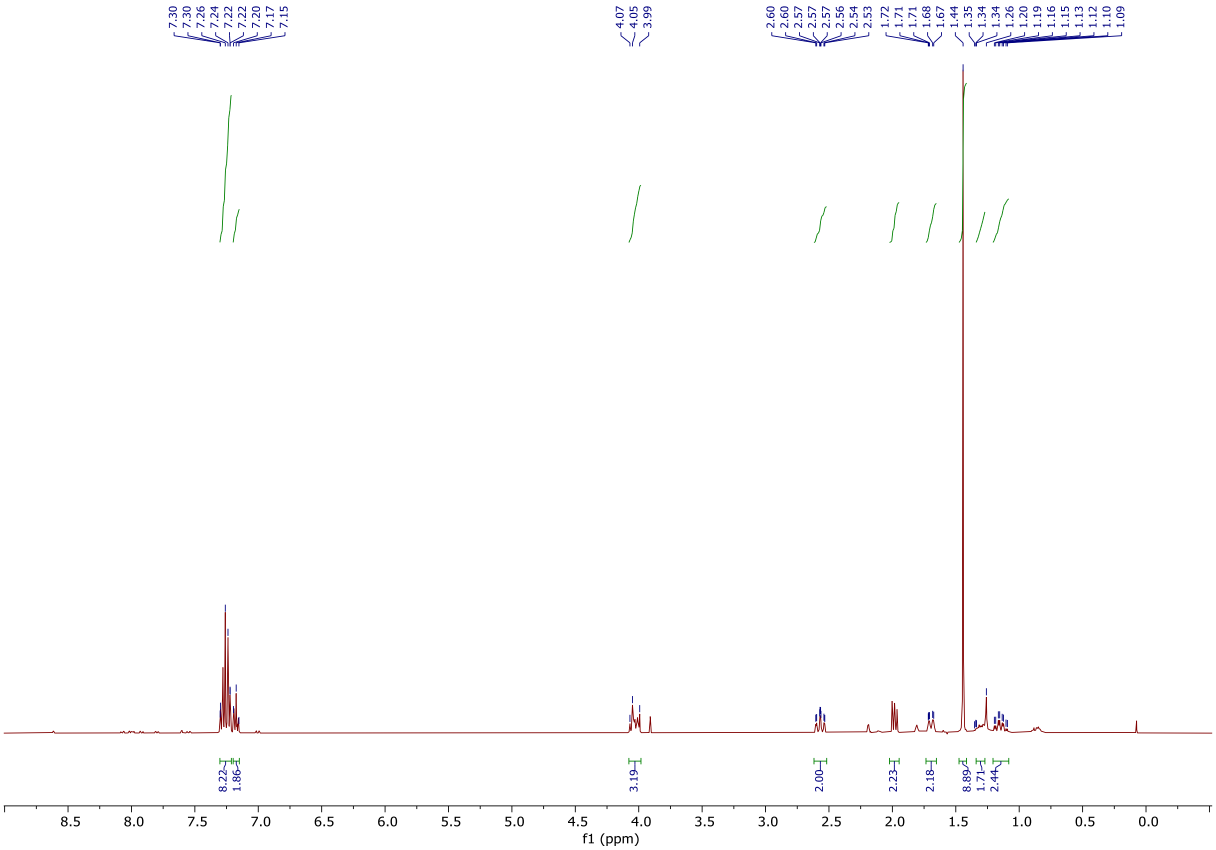

tert-Butyl 4-(2,2-diphenylethyl)piperidine-1-carboxylate (**21**) – ^{13}C{^{1}H} NMR spectrum (CDCl$_3$)

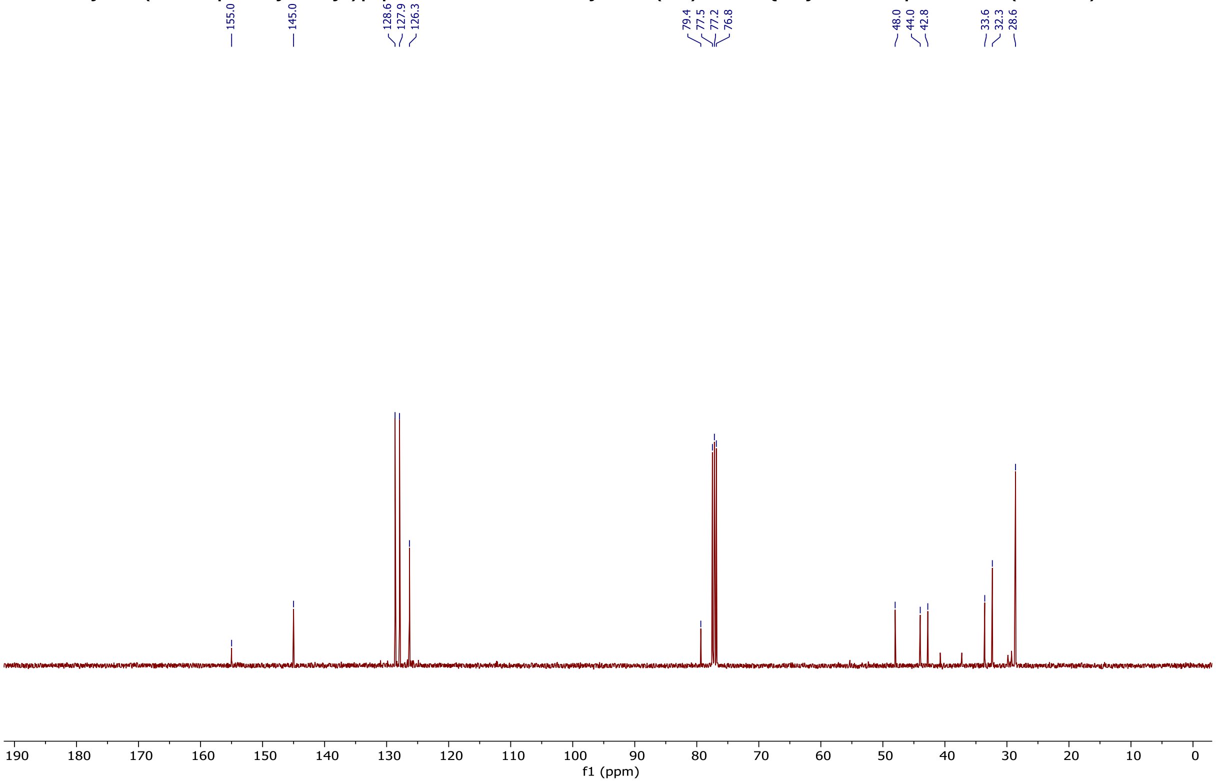

Cyclopentyltriphenylphosphonium iodide (**S1**) – ^{1}H NMR spectrum ($CDCl_3$)

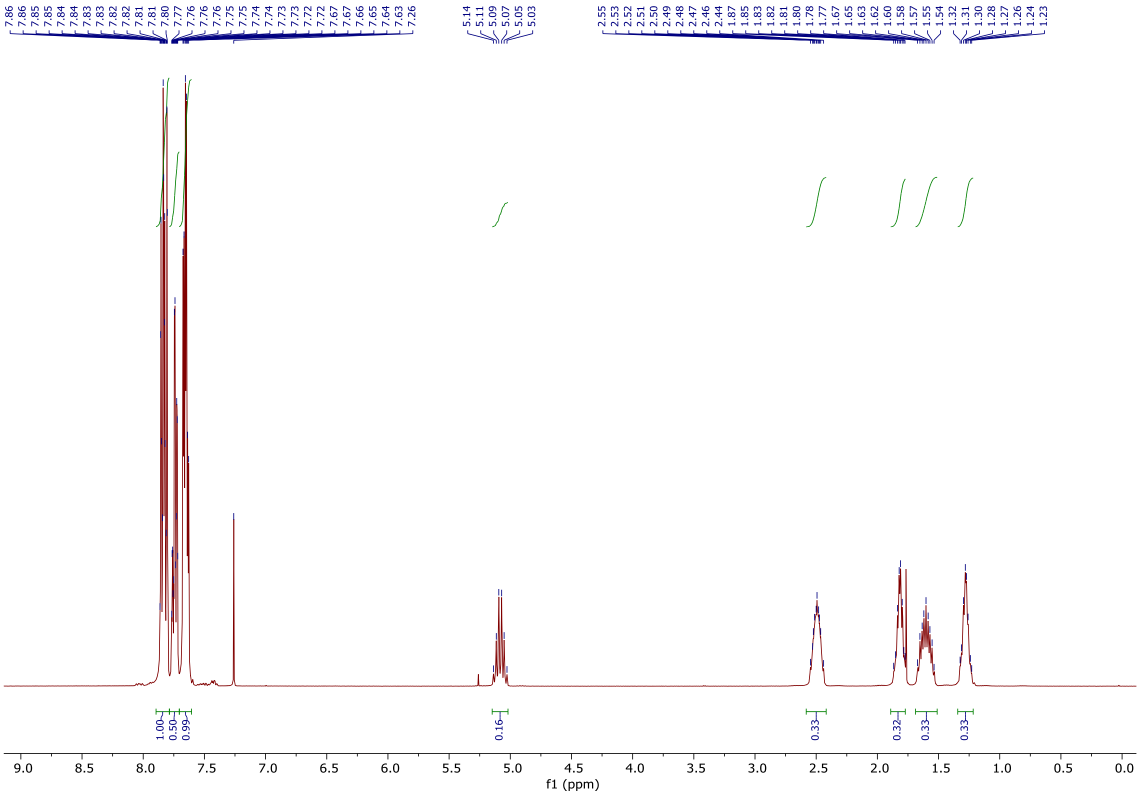

Cyclopentyltriphenylphosphonium iodide (**S1**) – $^{13}C\{^{1}H\}$ NMR spectrum ($CDCl_3$)

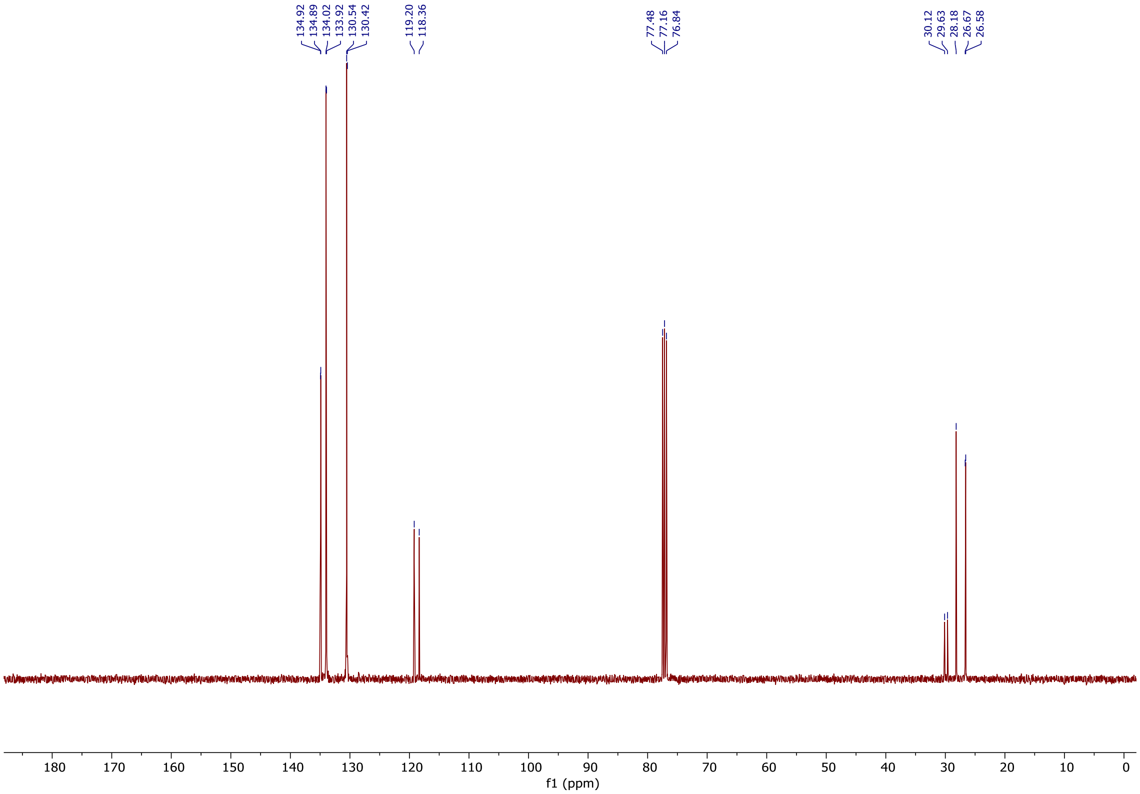

Cyclopentyltriphenylphosphonium iodide (**S1**) – $^{31}P\{^{1}H\}$ NMR spectrum ($CDCl_3$)

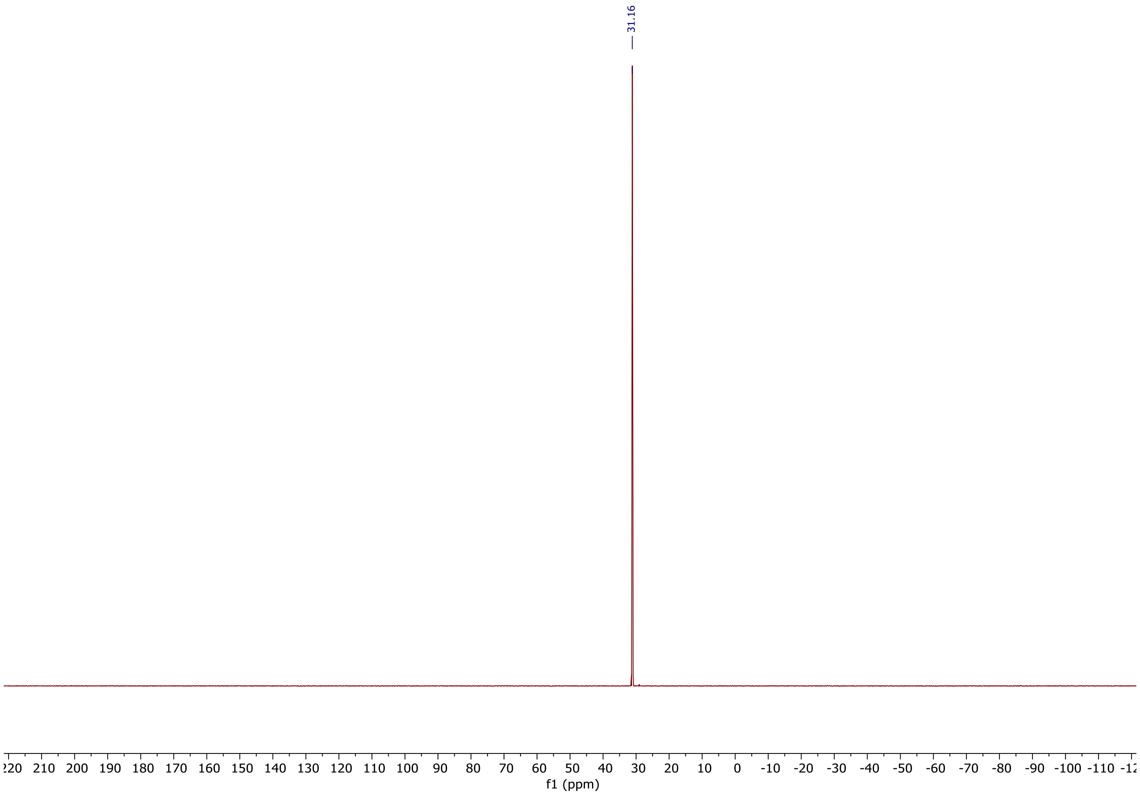

3-Phenylpropyl 4-methylbenzenesulfonate (**S3**) – ^{1}H NMR spectrum ($CDCl_3$)

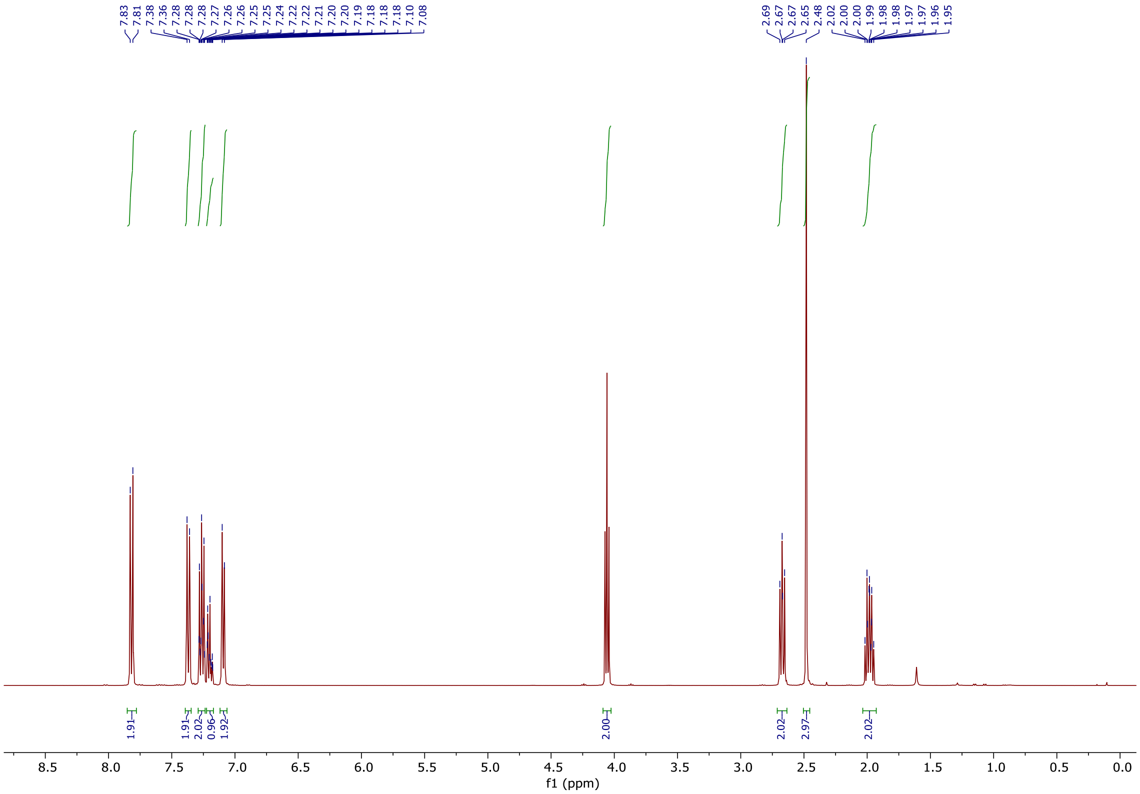

3-Phenylpropyl 4-methylbenzenesulfonate (**S3**) – $^{13}C\{^{1}H\}$ NMR spectrum ($CDCl_3$)

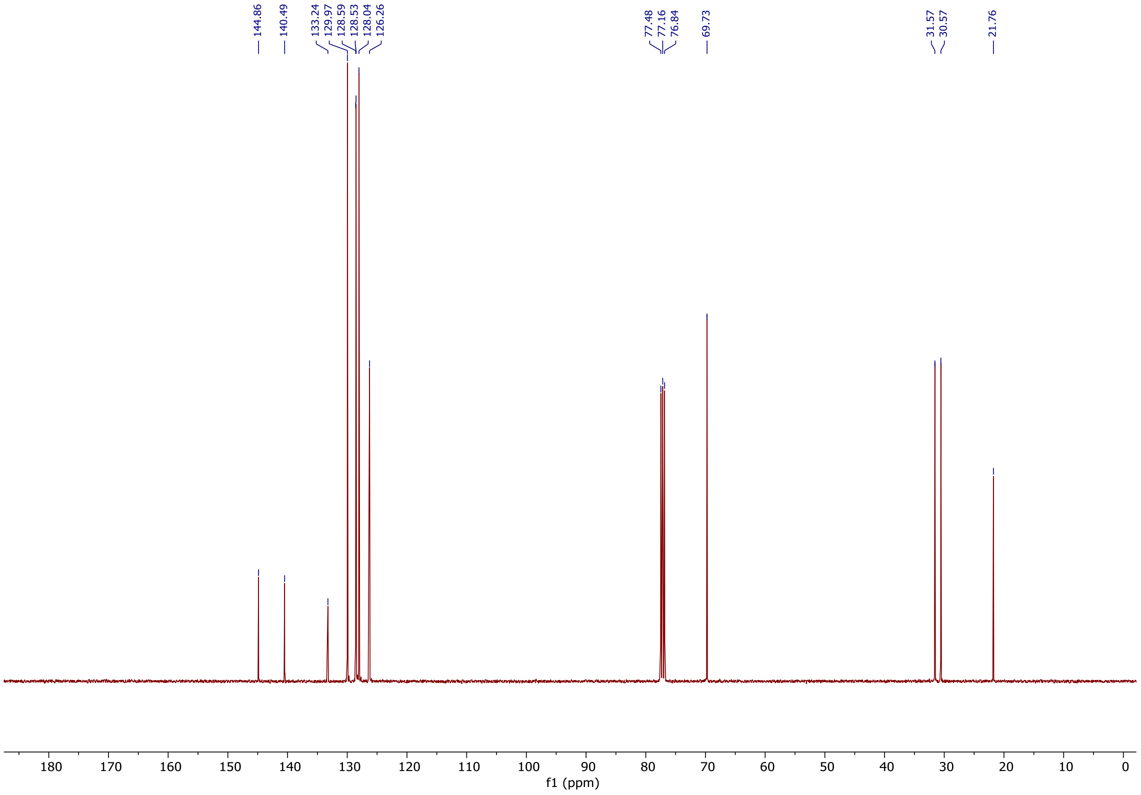

(3-Bromopropoxy)(*tert*-butyl)diphenylsilane (**S16**) – ^{1}H NMR spectrum ($CDCl_3$)

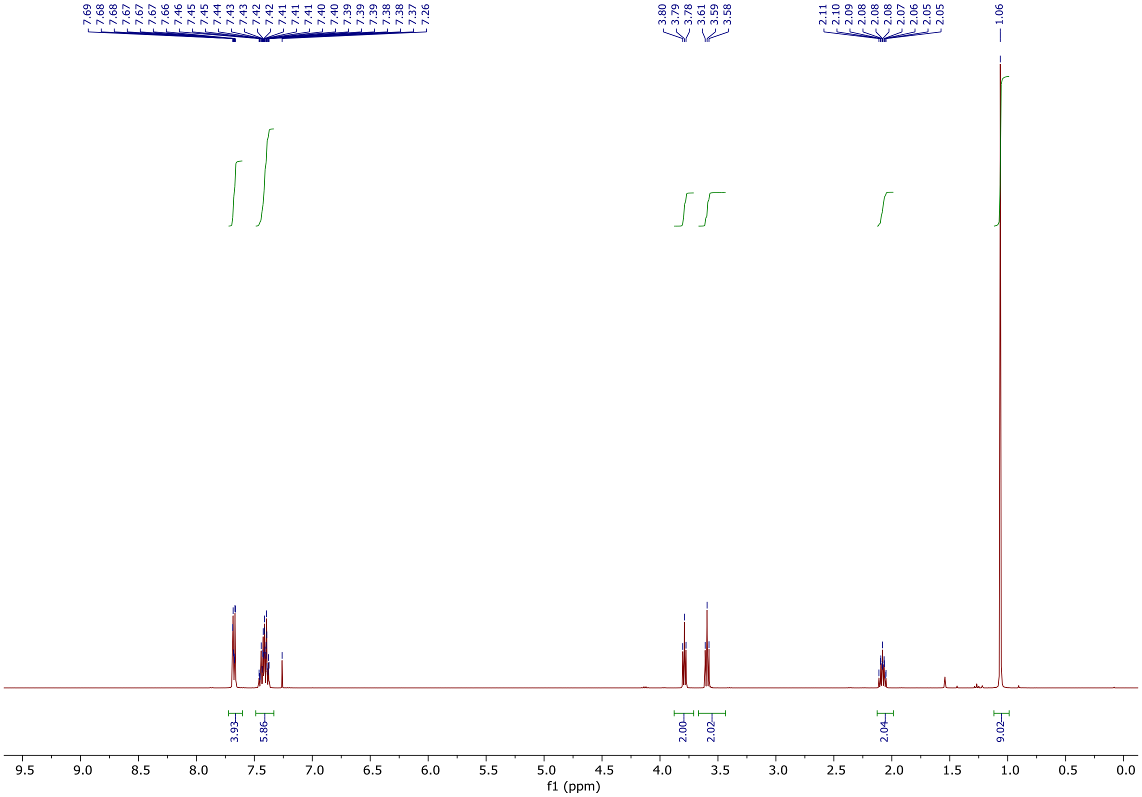

(3-Bromopropoxy)(*tert*-butyl)diphenylsilane (**S16**) – $^{13}C\{^{1}H\}$ NMR spectrum ($CDCl_3$)

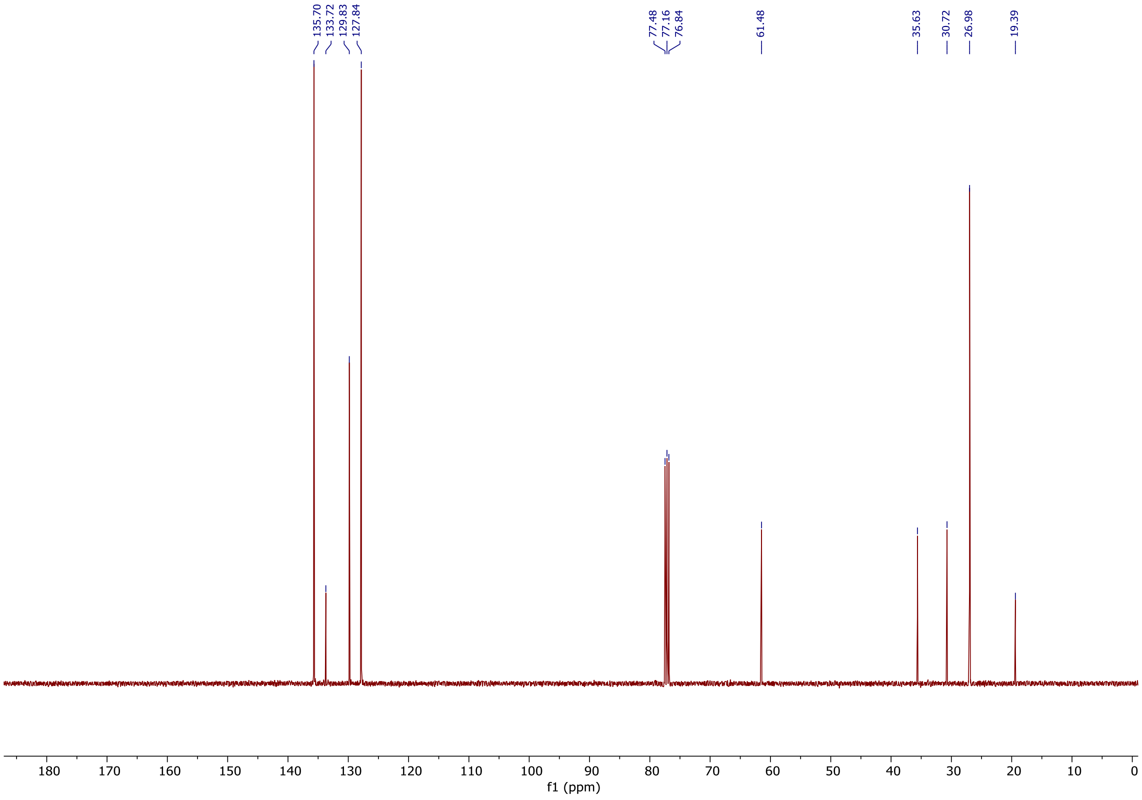

(3-Iodoropropyl)(phenyl)sulfane (**S18**) – ^{1}H NMR spectrum ($CDCl_3$)

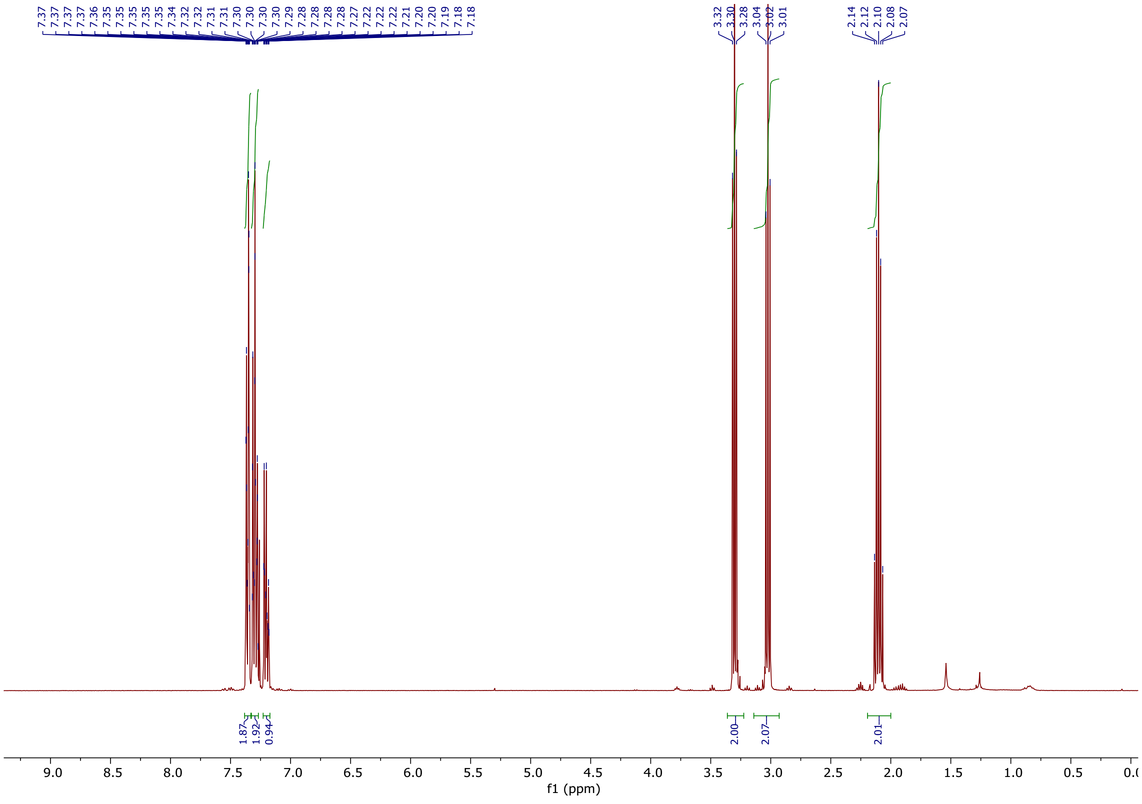

(3-Iodoropropyl)(phenyl)sulfane (**S18**) – $^{13}C\{^1H\}$ NMR spectrum ($CDCl_3$)

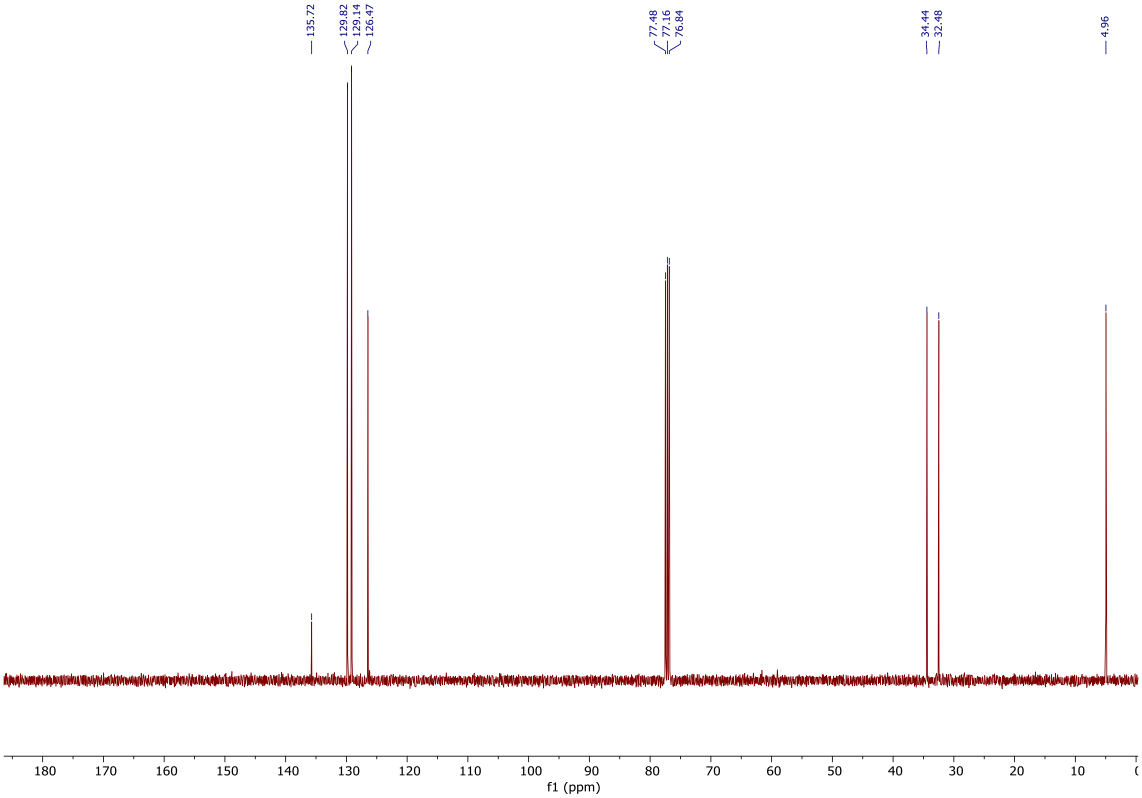

(3-(Hydroxymethyl)piperidin-1-yl)(phenyl)methanone (**S25**) – ^1H NMR spectrum ($CDCl_3$)

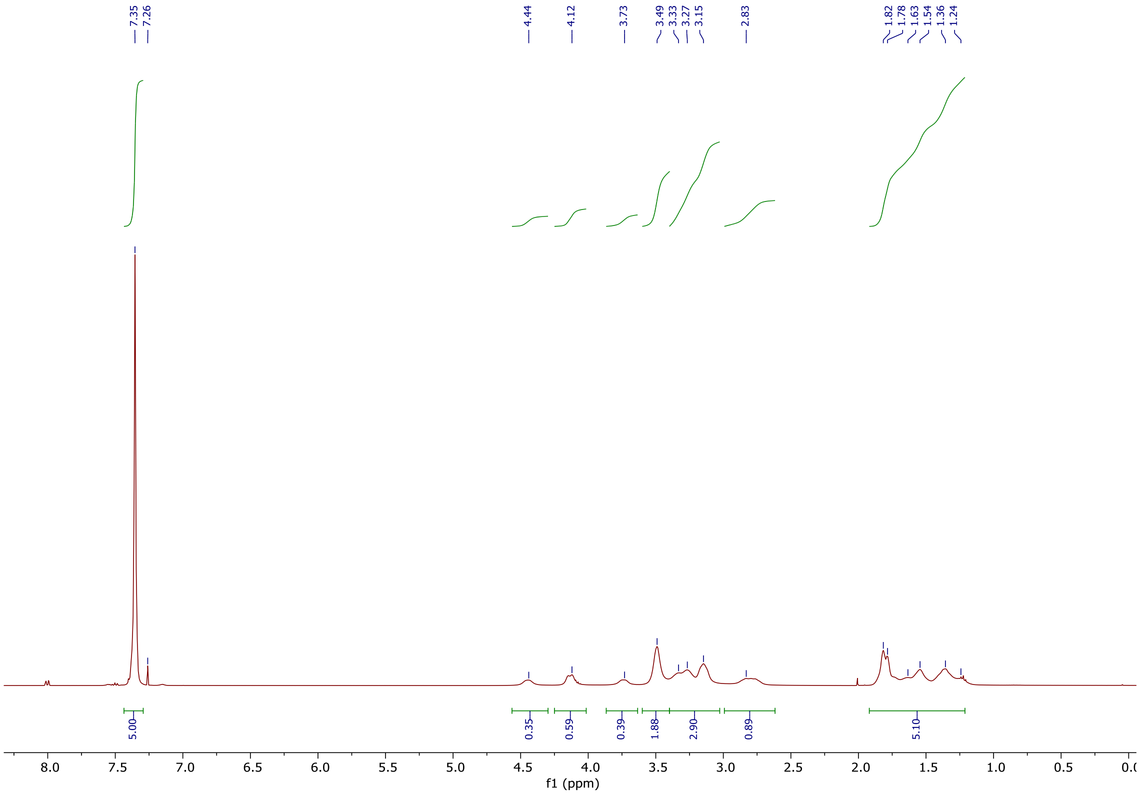

(3-(Hydroxymethyl)piperidin-1-yl)(phenyl)methanone (**S25**) – $^{13}C\{^{1}H\}$ NMR spectrum ($CDCl_3$)

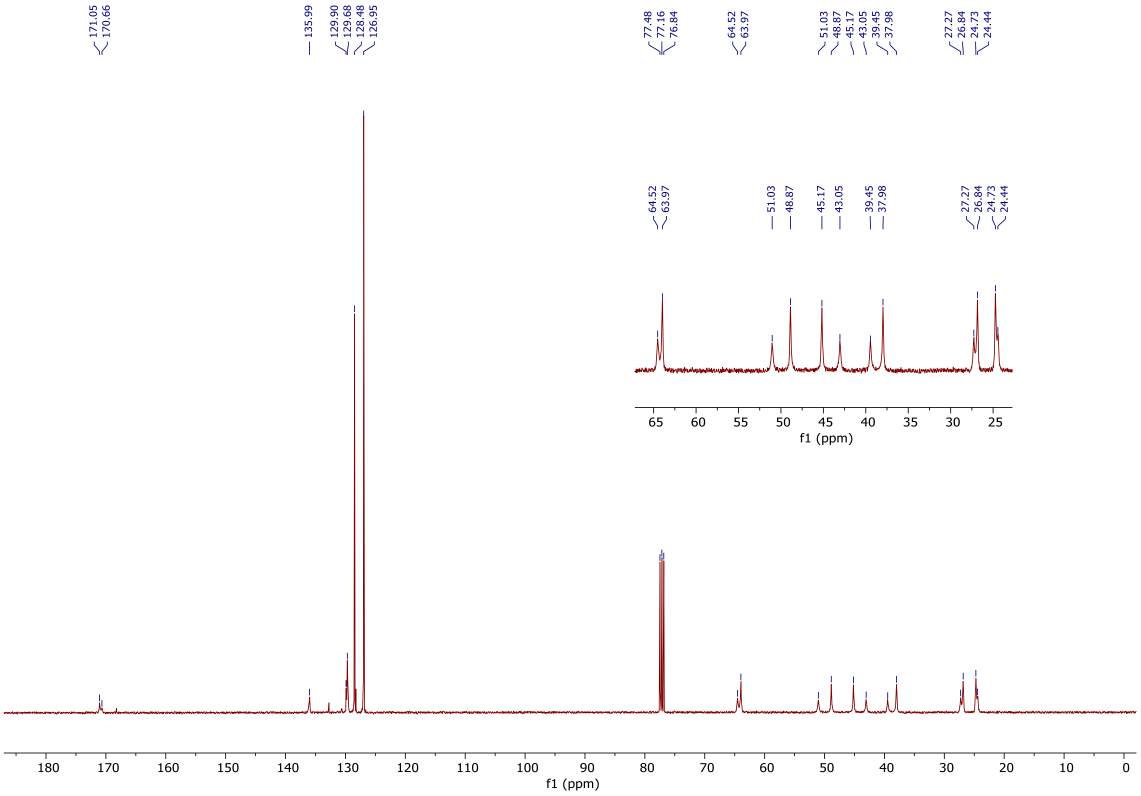

(3-(4-Bromophenyl)propoxy)(*tert*-butyl)dimethylsilane (**S30**) – ^{1}H NMR spectrum ($CDCl_3$)

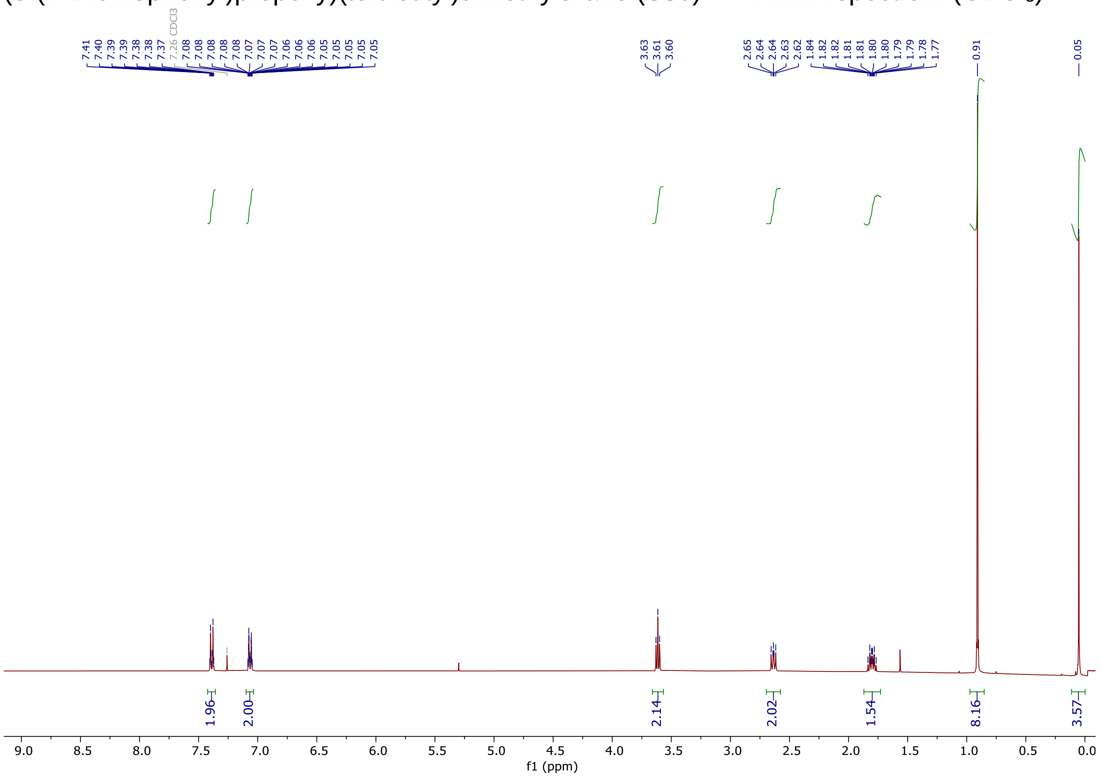

(3-(4-Bromophenyl)propoxy)(*tert*-butyl)dimethylsilane (**S30**) – $^{13}C\{^1H\}$ NMR spectrum ($CDCl_3$)

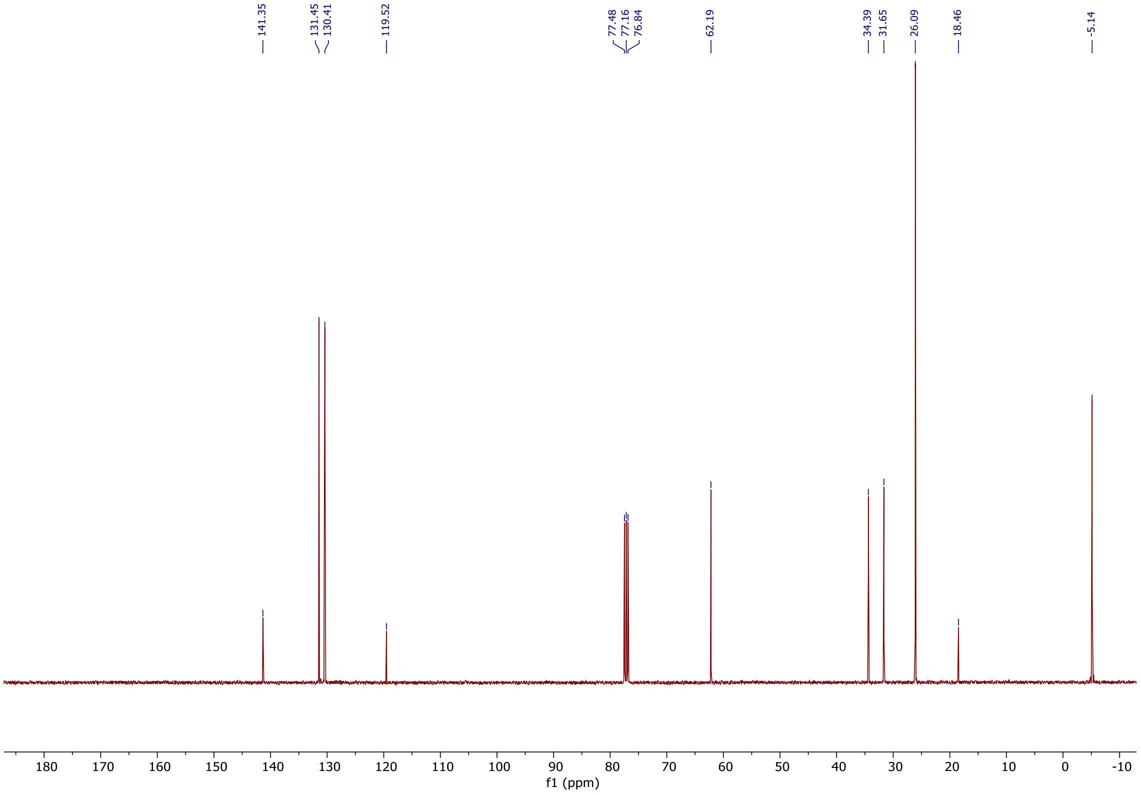

Alcohol **S32** – ^1H NMR spectrum ($CDCl_3$)

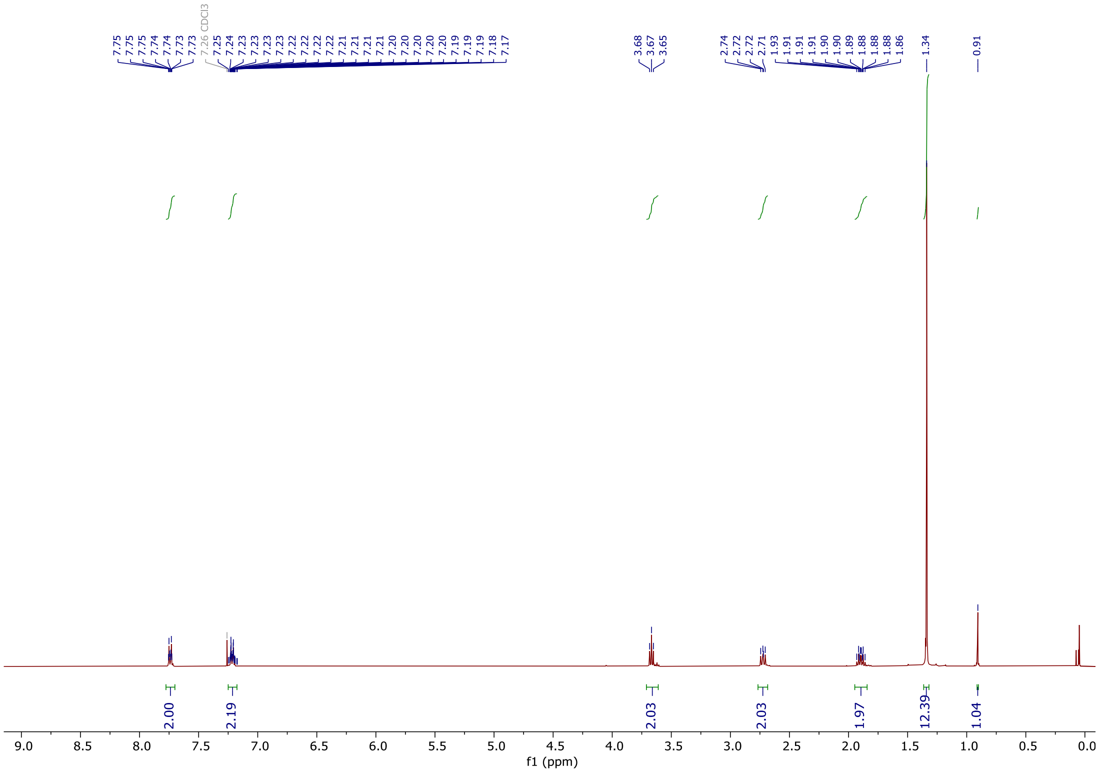

Alcohol **S32** – $^{13}C\{^{1}H\}$ NMR spectrum ($CDCl_3$)

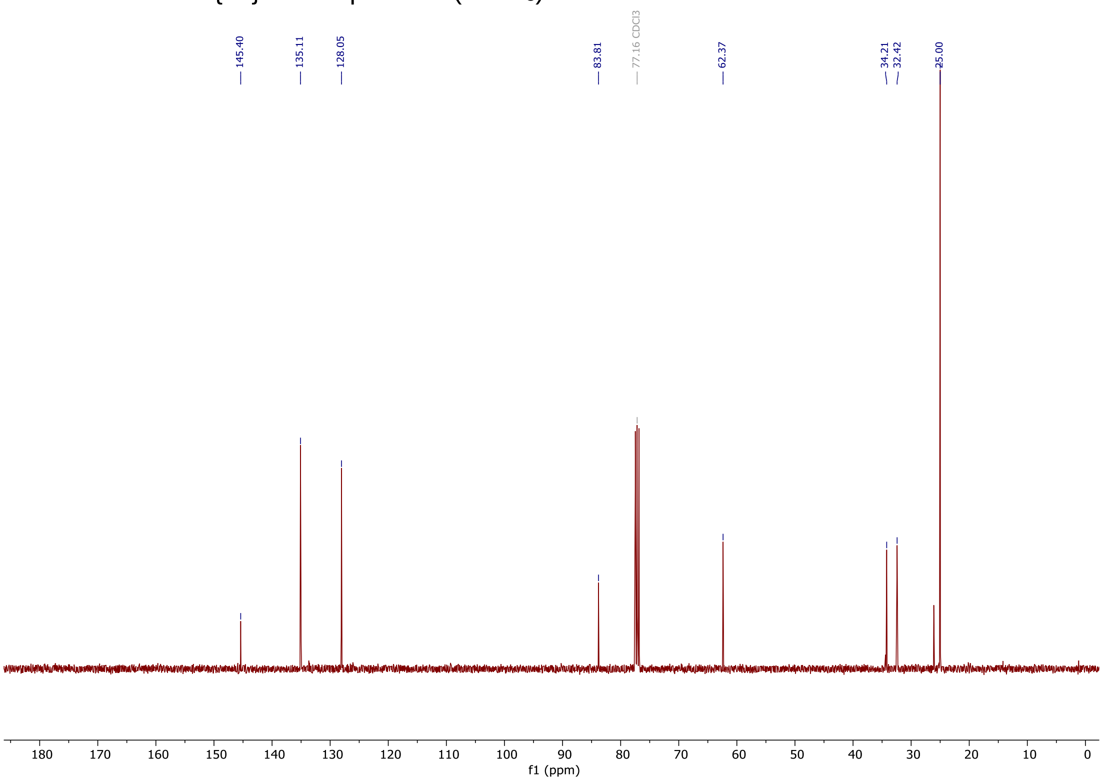

10-(4-Chlorobutyl)-10*H*-phenoxazine (**S34**) – ^{1}H NMR spectrum ($CDCl_3$)

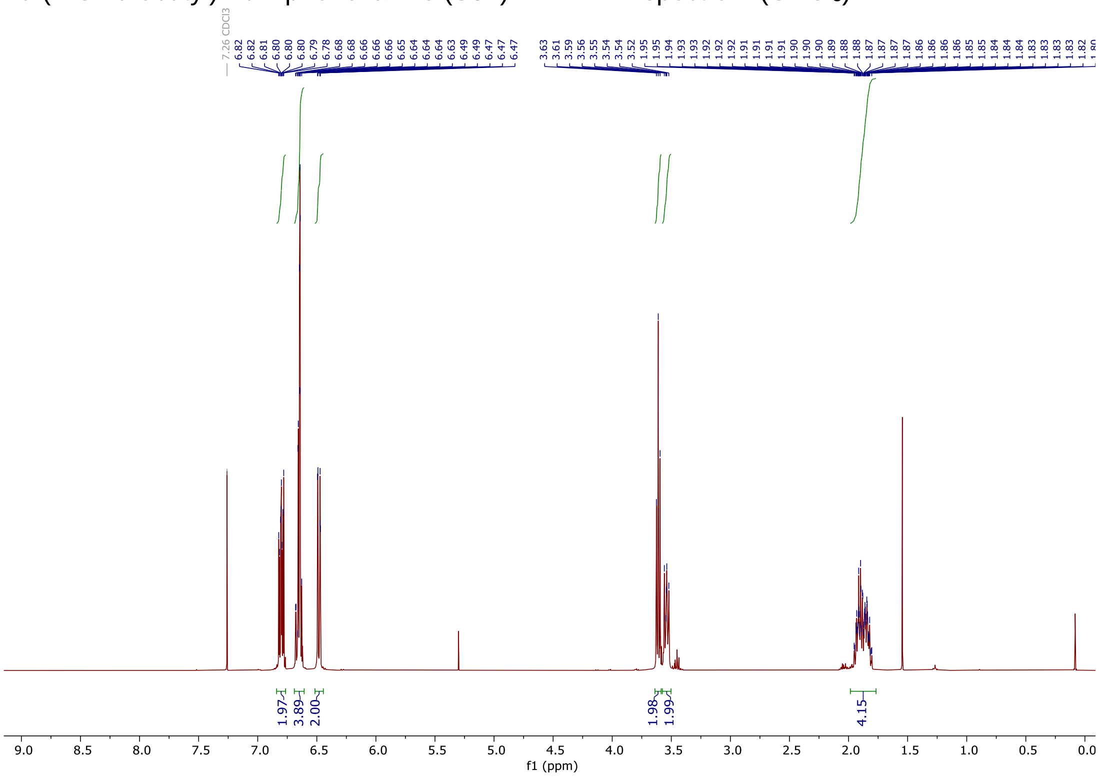

10-(4-Chlorobutyl)-10*H*-phenoxazine (**S34**) – $^{13}C\{^{1}H\}$ NMR spectrum ($CDCl_3$)

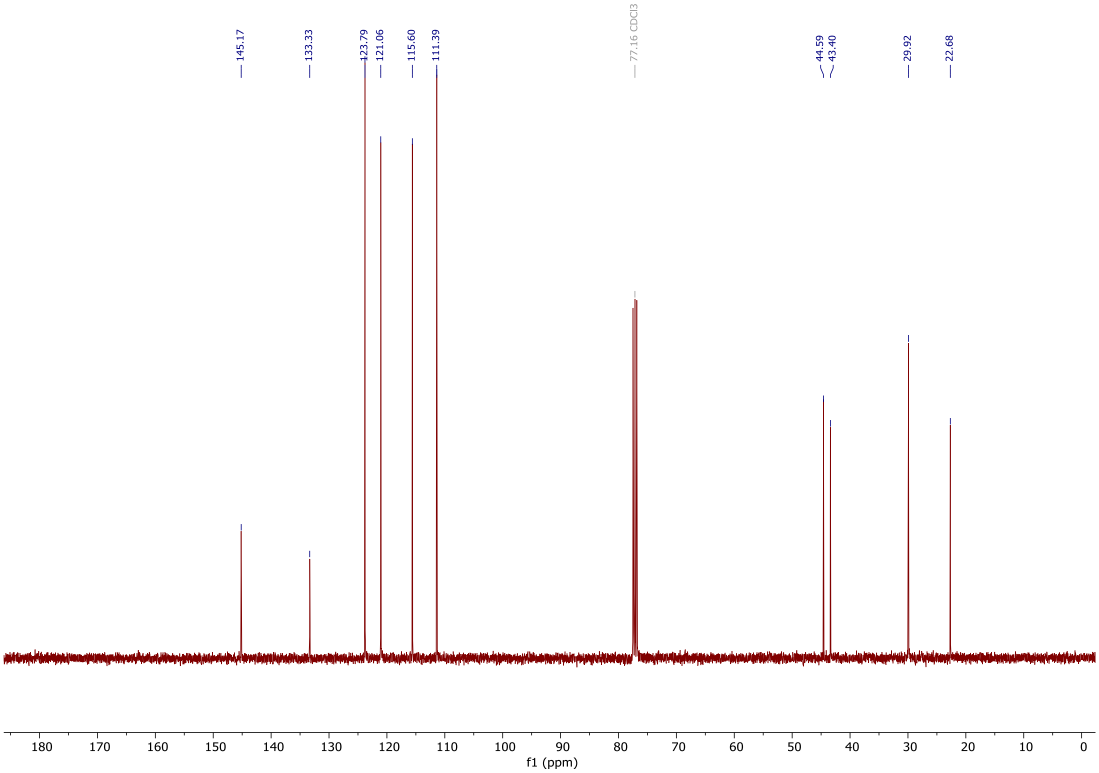

(4-(Hexyloxy)phenyl)methanol (**S39**) – ^{1}H NMR spectrum ($CDCl_3$)

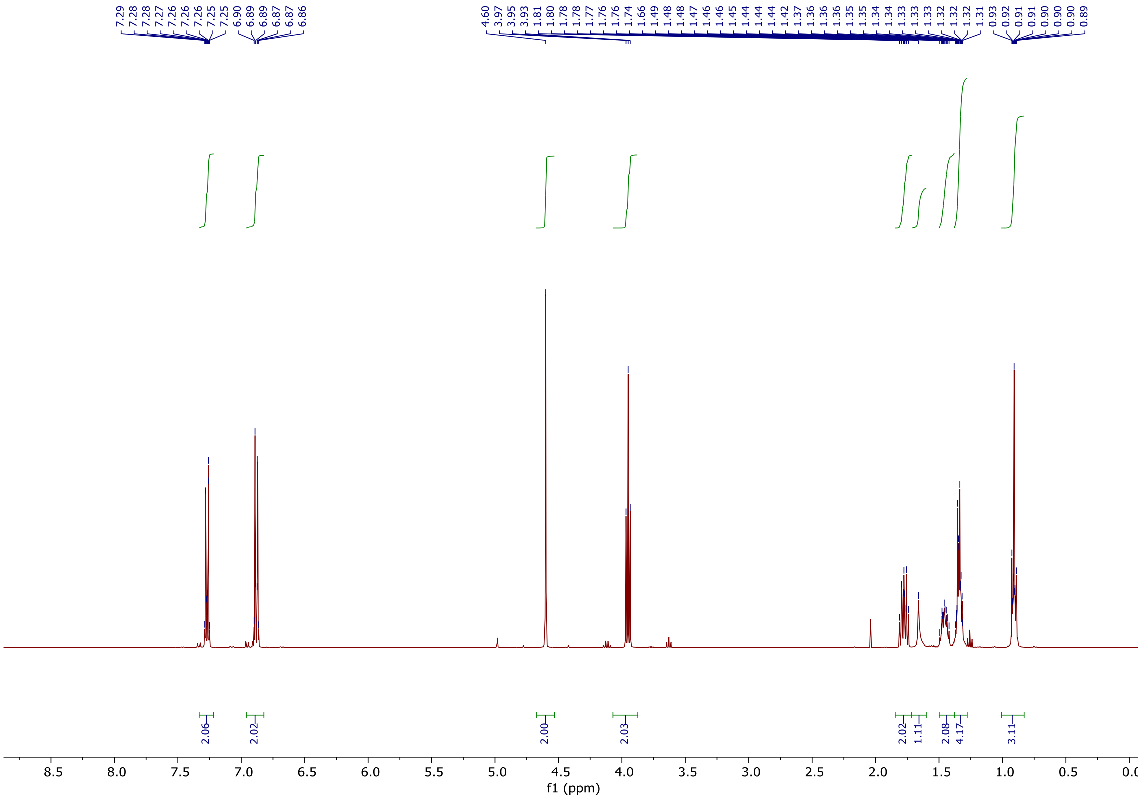

(4-(Hexyloxy)phenyl)methanol (**S39**) – $^{13}C\{^{1}H\}$ NMR spectrum ($CDCl_3$)

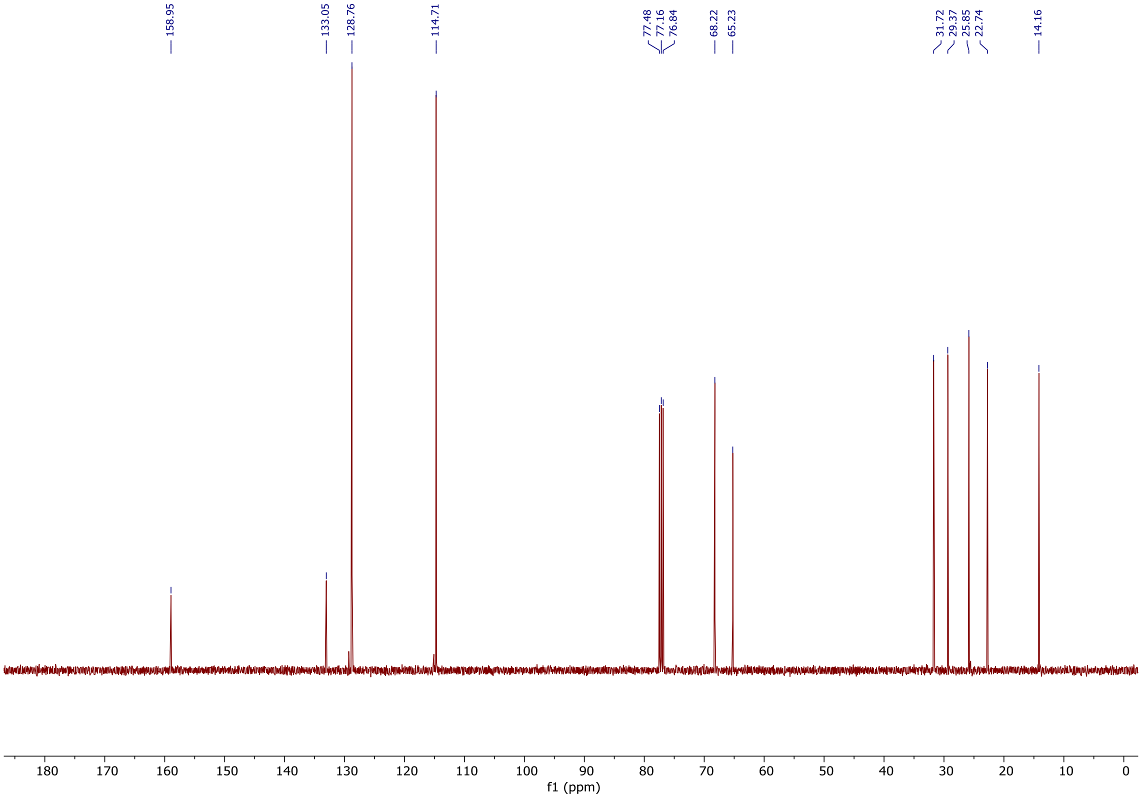

Alkyl chloride **S44** – ^{1}H NMR spectrum ($CDCl_3$)

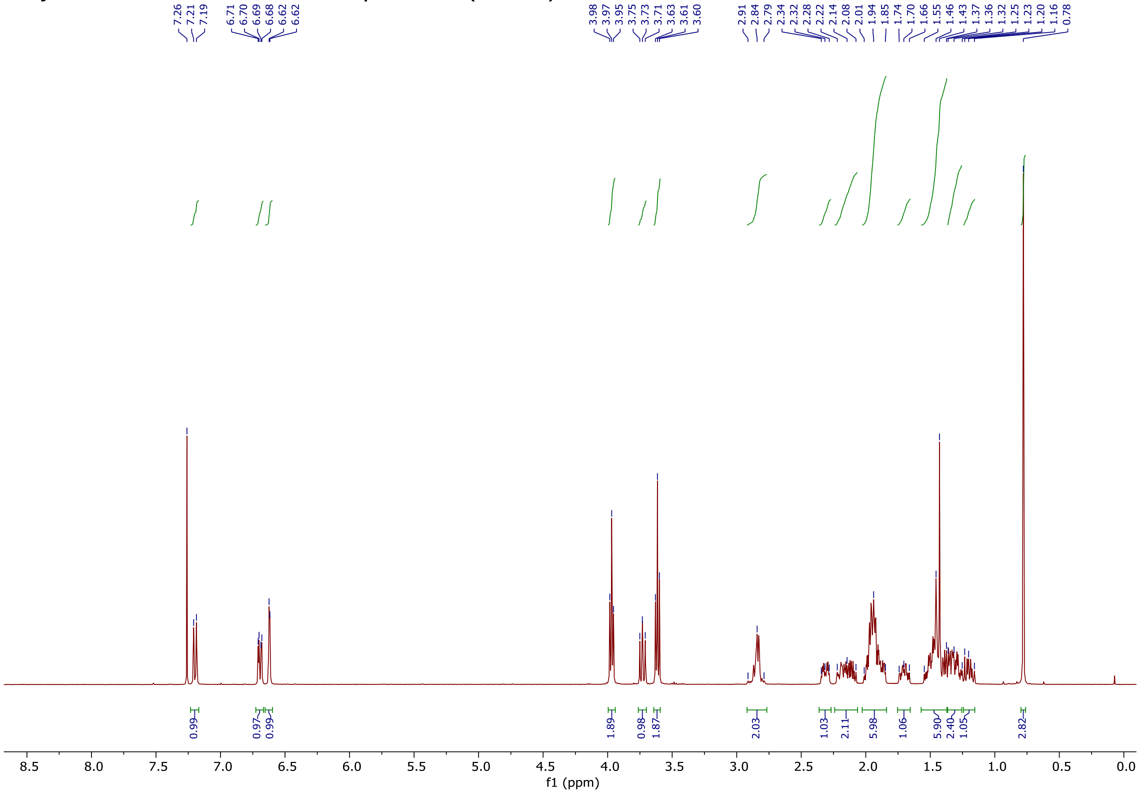

Alkyl chloride **S44** – $^{13}C\{^{1}H\}$ NMR spectrum ($CDCl_3$)

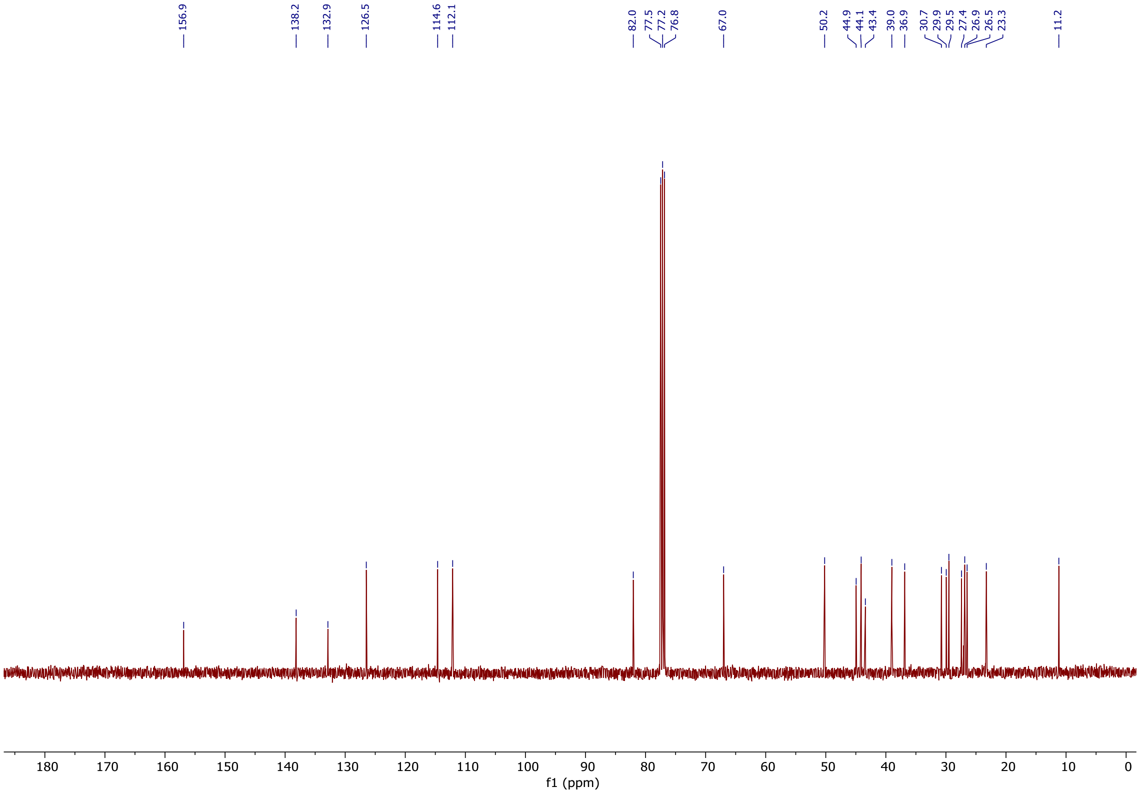

8. References

1. Sheldrick, G. M. SHELXT – Integrated space-group and crystal-structure determination. *Acta Crystallogr. Sect. A Found. Adv.* **71**, 3–8 (2015).
2. Sheldrick, G. M. A short history of SHELX. *Acta Crystallogr. Sect. A Found. Crystallogr.* **64**, 112–122 (2008).
3. Sheldrick, G. M. Crystal structure refinement with SHELXL. *Acta Crystallogr. Sect. C Struct. Chem.* **71**, 3–8 (2015).
4. Dolomanov, O. V., Bourhis, L. J., Gildea, R. J., Howard, J. A. K. & Puschmann, H. OLEX2 : a complete structure solution, refinement and analysis program. *J. Appl. Crystallogr.* **42**, 339–341 (2009).
5. Rezazadeh, S., Devannah, V. & Watson, D. A. Nickel-Catalyzed *C*-Alkylation of Nitroalkanes with Unactivated Alkyl Iodides. *J. Am. Chem. Soc.* **139**, 8110–8113 (2017).
6. Horner, L. & Hohndorf, W.-D. Phosphororganische Verbindungen 82 Darstellung und Eigenschaften einiger 1-Adamantyl-phosphoniumsalze. *Phosphorus* **6**, 71–75 (1976).
7. Hanstock, C. C. & Tebby, J. C. The Synthesis of bicyclic phosphonium salts. *Phosphorus Sulfur Relat. Elem.* **15**, 239–244 (1983).
8. Yoneda, N., Fukata, Y., Asano, K. & Matsubara, S. Asymmetric Synthesis of Spiroketals with Aminothiourea Catalysts. *Angew. Chem. Int. Ed.* **54**, 15497–15500 (2015).
9. Achyutha Rao, S., Chou, T.-S., Schipor, I. & Knochel, P. Preparation and Reactivity of Polyfunctional Zinc and Copper Organometallics Bearing Sulfur Functionalities. *Tetrahedron* **48**, 2025–2043 (1992).
10. Alapafuja, S. O. *et al.* Sulfonyl Fluoride Inhibitors of Fatty Acid Amide Hydrolase. *J. Med. Chem.* **55**, 10074–10089 (2012).
11. Gao, Y. *et al.* Visible-Light-Induced Nickel-Catalyzed Cross-Coupling with Alkylzirconocenes from Unactivated Alkenes. *Chem* **6**, 675–688 (2020).
12. Kumar, V. P., Babu, V. S., Yahata, K. & Kishi, Y. Fe/Cu-Mediated One-Pot Ketone Synthesis. *Org. Lett.* **19**, 2766–2769 (2017).
13. Kabalka, G. W. & Gooch, E. E. A mild and convenient procedure for conversion of alkenes into alkyl iodides via reaction of iodine monochloride with organoboranes. *J. Org. Chem.* **45**, 3578–3580 (1980).
14. Andersen, C. *et al.* Introduction of Cyclopropyl and Cyclobutyl Ring on Alkyl Iodides through Cobalt-Catalyzed Cross-Coupling. *Org. Lett.* **21**, 2285–2289 (2019).
15. Hirbawi, N., Lin, P. C. & Jarvo, E. R. Halogenation Reactions of Alkyl Alcohols Employing Methyl Grignard Reagents. *J. Org. Chem.* **87**, 12352–12369 (2022).
16. Bezençon, O. *et al.* Design and Preparation of Potent, Nonpeptidic, Bioavailable Renin Inhibitors. *J. Med. Chem.* **52**, 3689–3702 (2009).
17. Grimm, S. H. *et al.* Comprehensive structure-activity-relationship of azaindoles as highly potent FLT3 inhibitors. *Bioorg. Med. Chem.* **27**, 692–699 (2019).
18. Thimmaiah, K. N. *et al.* Synthesis and chemical characterization of N-substituted phenoxazines directed toward reversing vinca alkaloid resistance in multidrug-resistant cancer cells. *J. Med. Chem.* **35**, 3358–3364 (1992).
19. Zhao, S. *et al.* NIR-II Fluorescent Probe for Detecting Trimethylamine Based on Intermolecular Charge Transfer. *Chem. Eur. J.* **28**, e202200113 (2022).
20. Liu, X., Liu, B. & Liu, Q. Migratory Hydrogenation of Terminal Alkynes by Base/Cobalt Relay Catalysis. *Angew. Chem. Int. Ed.* **59**, 6750–6755 (2020).
21. Itsenko, O. & Långström, B. Radical-Mediated Carboxylation of Alkyl Iodides with [^{11}C]Carbon Monoxide in Solvent Mixtures. *J. Org. Chem.* **70**, 2244–2249 (2005).
22. Baumgartner, Y. & Baudoin, O. One-Pot Alkene Hydroboration/Palladium-Catalyzed Migratory Suzuki–Miyaura Cross-Coupling. *ACS Catal.* **10**, 10508–10515 (2020).
23. Liu, J.-H. *et al.* Copper-Catalyzed Reductive Cross-Coupling of Nonactivated Alkyl Tosylates and Mesylates with Alkyl and Aryl Bromides. *Chem. Eur. J.* **20**, 15334–15338 (2014).
24. Fisher, J. & Gradwell, M. J. Substituent Effects on ^{1}H Chemical Shifts. I—Complete ^{1}H Chemical Shift Assignments of Methyl-Substituted Cyclic Systems. *Magn. Reson. Chem.* **30**, 338–346 (1992).
25. Juliá, F., Constantin, T. & Leonori, D. Applications of Halogen-Atom Transfer (XAT) for the Generation of Carbon Radicals in Synthetic Photochemistry and Photocatalysis. *Chem. Rev.* **122**,

2292–2352 (2022).
26. Gao, L. *et al.* Organocatalytic decarboxylative alkylation of *N*-hydroxy-phthalimide esters enabled by pyridine-boryl radicals. *Chem. Commun.* **54**, 11534–11537 (2018).
27. Wu, Z. & Dong, G. Rapid Access to Multisubstituted Acrylamides from Cyclic Ketones via Palladium/Norbornene Cooperative Catalysis. *Angew. Chem. Int. Ed.* **61**, (2022).
28. Zhu, Q., Gentry, E. C. & Knowles, R. R. Catalytic Carbocation Generation Enabled by the Mesolytic Cleavage of Alkoxyamine Radical Cations. *Angew. Chem. Int. Ed.* **55**, 9969–9973 (2016).
29. Constantin, T. *et al.* Aminoalkyl radicals as halogen-atom transfer agents for activation of alkyl and aryl halides. *Science* **367**, 1021–1026 (2020).
30. Ding, Y., Luo, S., Ma, L. & An, J. Reductive Cleavage of Unactivated Carbon–Cyano Bonds under Ammonia-Free Birch Conditions. *J. Org. Chem.* **84**, 15827–15833 (2019).
31. Pracht, P., Bohle, F. & Grimme, S. Automated exploration of the low-energy chemical space with fast quantum chemical methods. *Phys. Chem. Chem. Phys.* **22**, 7169–7192 (2020).
32. Frisch, M. J. *et al.* Gaussian09. (2009).
33. Perdew, J. P., Burke, K. & Ernzerhof, M. Generalized Gradient Approximation Made Simple. *Phys. Rev. Lett.* **77**, 3865–3868 (1996).
34. Perdew, J. P., Burke, K. & Ernzerhof, M. Generalized Gradient Approximation Made Simple [Phys. Rev. Lett. 77, 3865 (1996)]. *Phys. Rev. Lett.* **78**, 1396–1396 (1997).
35. Adamo, C. & Barone, V. Toward reliable density functional methods without adjustable parameters: The PBE0 model. *J. Chem. Phys.* **110**, 6158–6170 (1999).
36. Ernzerhof, M. & Scuseria, G. E. Assessment of the Perdew–Burke–Ernzerhof exchange-correlation functional. *J. Chem. Phys.* **110**, 5029–5036 (1999).
37. Grimme, S., Antony, J., Ehrlich, S. & Krieg, H. A consistent and accurate *ab initio* parametrization of density functional dispersion correction (DFT-D) for the 94 elements H-Pu. *J. Chem. Phys.* **132**, 154104 (2010).
38. Grimme, S., Ehrlich, S. & Goerigk, L. Effect of the damping function in dispersion corrected density functional theory. *J. Comput. Chem.* **32**, 1456–1465 (2011).
39. Marenich, A. V., Cramer, C. J. & Truhlar, D. G. Universal Solvation Model Based on Solute Electron Density and on a Continuum Model of the Solvent Defined by the Bulk Dielectric Constant and Atomic Surface Tensions. *J. Phys. Chem. B* **113**, 6378–6396 (2009).
40. Weigend, F. & Ahlrichs, R. Balanced basis sets of split valence, triple zeta valence and quadruple zeta valence quality for H to Rn: Design and assessment of accuracy. *Phys. Chem. Chem. Phys.* **7**, 3297 (2005).
41. Weigend, F. Accurate Coulomb-fitting basis sets for H to Rn. *Phys. Chem. Chem. Phys.* **8**, 1057 (2006).
42. Grimme, S. Supramolecular Binding Thermodynamics by Dispersion-Corrected Density Functional Theory. *Chem. Eur. J.* **18**, 9955–9964 (2012).
43. Luchini, G., Alegre-Requena, J. V., Funes-Ardoiz, I. & Paton, R. S. GoodVibes: automated thermochemistry for heterogeneous computational chemistry data. *F1000Research* **9**, 291 (2020).
44. Neese, F. The ORCA program system. *WIREs Comput. Mol. Sci.* **2**, 73–78 (2012).
45. Neese, F. Software update: the ORCA program system, version 4.0. *WIREs Comput. Mol. Sci.* **8**, e1327 (2018).
46. Neese, F. Software update: The ORCA program system—Version 5.0. *WIREs Comput. Mol. Sci.* **12**, e1606 (2022).
47. Zhao, Y. & Truhlar, D. G. Design of Density Functionals That Are Broadly Accurate for Thermochemistry, Thermochemical Kinetics, and Nonbonded Interactions. *J. Phys. Chem. A* **109**, 5656–5667 (2005).
48. Lu, T. & Chen, F. Multiwfn: A multifunctional wavefunction analyzer. *J. Comput. Chem.* **33**, 580–592 (2012).
49. Humphrey, W., Dalke, A. & Schulten, K. VMD: Visual molecular dynamics. *J. Mol. Graph.* **14**, 33–38 (1996).
50. Yanai, T., Tew, D. P. & Handy, N. C. A new hybrid exchange–correlation functional using the Coulomb-attenuating method (CAM-B3LYP). *Chem. Phys. Lett.* **393**, 51–57 (2004).
51. Falivene, L. *et al.* Towards the online computer-aided design of catalytic pockets. *Nat. Chem.* **11**, 872–879 (2019).
52. Legault, C. Y. CYLview20. (2020).

53. Bogdos, M. K. & Morandi, B. EveRplot: A Web-Based Shiny Application for Creating Energy vs Reaction Coordinate Diagrams. *J. Chem. Educ.* **100**, 3641–3644 (2023).
54. Griffin, C. E. & Kaufman, M. L. Photolysis of benzyltriphenylphosphonium and tetraphenylphosphonium chlorides. *Tetrahedron Lett.* **6**, 773–775 (1965).
55. Lin, Q.-Y., Xu, X.-H., Zhang, K. & Qing, F.-L. Visible-Light-Induced Hydrodifluoromethylation of Alkenes with a Bromodifluoromethylphosphonium Bromide. *Angew. Chem. Int. Ed.* **55**, 1479–1483 (2016).
56. Yu, J., Lin, J.-H., Cao, Y.-C. & Xiao, J.-C. Visible-light-induced radical hydrodifluoromethylation of alkenes. *Org. Chem. Front.* **6**, 3580–3583 (2019).
57. Liu, Q. *et al.* Visible-Light-Induced Selective Photolysis of Phosphonium Iodide Salts for Monofluoromethylations. *Angew. Chem. Int. Ed.* **60**, 25477–25484 (2021).
